# GRASP55 maintains lysosome function by controlling sorting of lysosomal enzymes at the Golgi

**DOI:** 10.1038/s44319-026-00773-w

**Published:** 2026-04-16

**Authors:** Julian Nüchel, Maryam Omidi, Stephanie A Fernandes, Marina Tauber, Sandra Pohl, Markus Plomann, Constantinos Demetriades

**Affiliations:** 1 Max Planck Institute for Biology of Ageing (MPI-AGE), Cologne, Germany; 2 University of Cologne, Faculty of Medicine and University Hospital Cologne, Center for Biochemistry, Cologne, Germany; 3 University of Cologne, Center for Molecular Medicine Cologne (CMMC), Cologne, Germany; 4 Department of Osteology and Biomechanics (IOBM), University Medical Center Hamburg-Eppendorf, Hamburg, Germany; 5 University of Cologne, Cologne Excellence Cluster for Aging and Aging-Associated Diseases (CECAD), Cologne, Germany; 6 European Research Institute for the Biology of Ageing (ERIBA), University of Groningen (RUG), University Medical Center Groningen (UMCG), Groningen, The Netherlands

**Keywords:** Membranes & Trafficking, Organelles, Signal Transduction

## Abstract

Lysosomes are multifunctional organelles that play important roles in cellular recycling, signaling, and homeostasis, relying on precise trafficking and activation of lysosomal enzymes. While the Golgi apparatus plays a central role in lysosomal enzyme sorting, the mechanisms linking Golgi function to lysosomal activity remain incompletely understood. Here, we identify the Golgi-resident protein GRASP55, but not its paralog GRASP65, as necessary for lysosome function. Loss of GRASP55 expression leads to missorting and secretion of lysosomal enzymes, lysosomal dysfunction and bloating. GRASP55 deficiency also disrupts lysosomal mTORC1 signaling, reducing the phosphorylation of its lysosomal substrates TFEB/TFE3, while sparing its non-lysosomal targets. Mechanistically, GRASP55 binds and maintains the COPI adaptor GOLPH3 protein at the Golgi, thereby controlling the Golgi localization and stability of LYSET and GNPTAB that are required for mannose 6-phosphate (M6P) tagging of lysosomal enzymes. These findings reveal an essential role for GRASP55 in Golgi–lysosome communication and lysosomal enzyme trafficking and underscore the importance of Golgi-mediated protein sorting in lysosome function and lysosomal mTORC1 signaling.

## Synopsis

GRASP55 is a gatekeeper of lysosomal enzyme sorting at the Golgi, thus being necessary for proper lysosome function, morphology and lysosomal mTORC1 signaling.

- Cells lacking GRASP55 expression demonstrate lysosomal enzyme missorting and secretion, lysosomal dysfunction and bloating, and perturbed lysosomal mTORC1-TFEB/TFE3 signaling.
- GRASP55 is responsible for keeping GOLPH3, LYSET and GNPTAB at the Golgi, via interactions with the former.
- In the absence of GRASP55, the mislocalization of LYSET and GNPTAB is linked to their destabilization, in turn causing defective M6P tagging and missorting of lysosomal enzymes.

## Abstract (web summary)

GRASP55 is a gatekeeper of lysosomal enzyme sorting at the Golgi, thus being necessary for proper lysosome function, morphology and lysosomal mTORC1 signaling.

## Graphical abstract

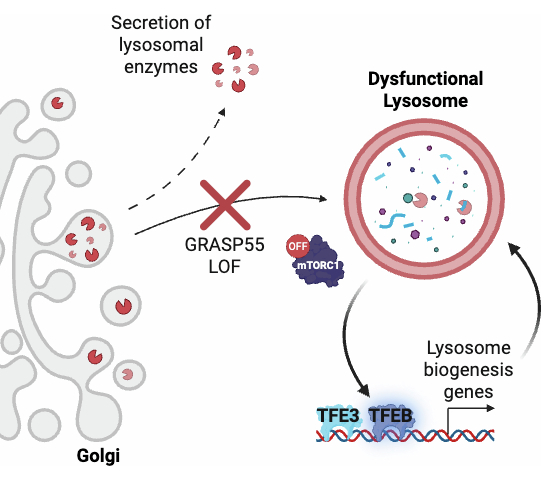

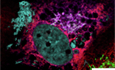

## Introduction

Lysosomes are the main degradative organelles in cells. By breaking down their content, lysosomes recycle material on a continuous basis, thereby providing cells with fresh building blocks to support anabolic functions (Ballabio and Bonifacino, 2020; Parenti et al, 2021; Perera and Zoncu, 2016; Saftig and Klumperman, 2009). Work over the last 15 years has expanded this view further, showing that the lysosomal surface also serves as a platform for a number of key signaling molecules, like the growth- and metabolism-related mTOR (mechanistic/mammalian Target of Rapamycin) kinase (Acharya and Demetriades, 2024; Fernandes et al, 2024; Gollwitzer et al, 2022; Sancak et al, 2010), or its upstream negative regulator, the tumor suppressor TSC (Tuberous Sclerosis Complex) protein complex (Demetriades et al, 2014; Demetriades et al, 2016; Fitzian et al, 2021; Plescher et al, 2015; Prentzell et al, 2021), both of which are regulated by nutrient and stress signals at this subcellular location to coordinate anabolic and catabolic processes in cells (Fernandes and Demetriades, 2021; He et al, 2024; Rabanal-Ruiz and Korolchuk, 2018). At the same time, lysosomes remove insoluble aggregates and damaged proteins or even organelles, whose accumulation can cause severe cell damage and is a hallmark of neurological disorders such as Alzheimer’s and Parkinson’s (Cuervo et al, 2004; Dehay et al, 2010; Nixon et al, 2005), thus underscoring the importance of proper lysosomal function for human disease and ageing (Ballabio and Bonifacino, 2020; Parenti et al, 2021).

Lysosomal cargo is degraded by soluble lysosomal enzymes that break down a variety of structurally diverse compounds, such as proteins, lipids, sugars, nucleic acids, and other macromolecules. The directed vesicle-mediated transport of newly synthesized lysosomal enzymes to these organelles involves a complex system of sorting signals and recognition proteins, a process that takes place in the Golgi apparatus, the main processing and distribution center for cargo proteins in cells (Bajaj et al, 2019; Honing et al, 1998; Lefkir et al, 2003; Parenti et al, 2021). One such well-described signal is the modification of specific mannose residues on lysosomal enzymes that reach the Golgi from the endoplasmic reticulum (ER) and the ER-Golgi intermediate compartment (ERGIC) by the addition of phosphate groups to generate mannose 6-phosphate (M6P) (Braulke and Bonifacino, 2009; Dahms et al, 1989; Kornfeld and Kornfeld, 1985; Li et al, 2022; Pohl et al, 2009; Qian et al, 2010). The key enzyme in the formation of M6P residues is the heterohexameric (α_2_β_2_γ_2_) GlcNAc-1-phosphotransferase (GNPT) complex. The *GNPTAB* gene encodes the transmembrane α/β precursor of GNPT, whereas *GNPTG* encodes the soluble γ subunit (Kollmann et al, 2010). After assembly of the GNPT subunits in the ER, the inactive complex is transported to the *cis*-Golgi and the α/β precursor (here referred to as GNPTAB) is proteolytically processed by site-1 protease (S1P) into the enzymatically active α and β subunits (De Pace et al, 2015; Encarnacao et al, 2011; Marschner et al, 2011) (hereafter referred to as GNPTA and GNPTB, respectively). Recent studies have identified the cis-Golgi transmembrane protein LYSET (also known as TMEM251 or GCAF) as a key factor that is necessary for the stabilization of GNPTAB at the Golgi (Pechincha et al, 2022; Richards et al, 2022; Zhang et al, 2023). In turn, the correct intra-Golgi localization of LYSET is established via the function of the COPI adaptor proteins GOLPH3/GOLPH3L that actively retrieve it from medial and trans-Golgi membranes to prevent its missorting and degradation at lysosomes (Brauer et al, 2024). The M6P residues on lysosomal proteins are recognized by M6P receptors (MPRs) in specialized *trans*-Golgi subdomains, which facilitate their transportation to late endosomes and lysosomes (Arighi et al, 2004; Bajaj et al, 2019; Braulke and Bonifacino, 2009; Dahms et al, 1989; Ghosh et al, 2003; Puertollano et al, 2001). Lysosomal enzyme activity requires the acidic environment inside the lysosomal lumen that is established by the V-ATPase (vacuolar H^+^-ATPase) and other transmembrane ion channels (Mauvezin and Neufeld, 2015). Besides ensuring that lysosomal enzymes are only active inside lysosomes, the acidic environment of the lumen plays an important role also for their delivery to these organelles, as MPRs bind M6P-tagged proteins at pH 6.7 in the *trans*-Golgi and release it in the endosomes at pH 6 due to changes in their affinity and conformation (Ghosh et al, 2003; Mullins and Bonifacino, 2001).

Pathogenic variants in many lysosomal enzymes or proteins participating in their maturation and trafficking have catastrophic effects at the cellular and organismal levels, and cause lysosomal storage disorders (LSDs) in humans. More than 70 genetically distinct types of LSDs have been reported to date (Bajaj et al, 2019; Ballabio and Gieselmann, 2009; Parenti et al, 2021; Platt et al, 2018). Although LSDs are individually rare, collectively, they are found at relatively high incidence, with more than 1 in 5000 live births affected in the general population (Boustany, 2013; Parenti et al, 2021; Platt et al, 2018). While the symptomatology varies a lot between disorders in the LSD spectrum, most diseases affect the central nervous system, while some manifest with musculoskeletal system defects, impaired growth, and reduced life span (Parenti et al, 2021; Platt et al, 2018). Whereas the vast majority of LSDs are caused by pathogenic variants in genes that code for soluble lysosomal enzymes or lysosomal membrane proteins, genetic defects in *GNPTAB*, encoding the catalytic subunits of the Golgi-resident GNPT complex, result in mistargeting of lysosomal enzymes and the severe human disease mucolipidosis type II (ML-II, formerly known as I-cell disease) (Tiede et al, 2005; Velho et al, 2019). Cells from these patients are characterized by accumulation of non-degraded macromolecules, lysosomal bloating, and missorting of lysosomal enzymes that are secreted to the extracellular space as inactive proenzymes, instead of being delivered to lysosomes. Therefore, a key diagnostic feature in ML-II is the increased levels of lysosomal enzymes in the circulation (Kollmann et al, 2010; Velho et al, 2019). Highlighting its key role in maintaining cellular physiology, perturbation of Golgi function is also linked to cancer growth, invasion and metastasis, in addition to its involvement in the etiology of LSDs (reviewed in (Bui et al, 2021)).

Unconventional protein secretion (UPS) is an alternative secretory route via which specific cargo proteins reach the cell surface or the extracellular space (Rabouille, 2017). In contrast to bulk secretory pathways, UPS is induced upon nutrient starvation or stress, and is, therefore, part of the cellular adaptation to stress. A central regulator of UPS is the Golgi-resident protein GRASP55 (Golgi re-assembly and stacking protein 55; also referred to as GORASP2) (Nüchel et al, 2018; Nüchel et al, 2021; Shorter et al, 1999). Besides controlling UPS, together with the closely-related GRASP65 protein, GRASP55 was originally described to be involved in the assembly and membrane stacking of the Golgi, although its precise role in Golgi structure and function is not fully understood yet (Barr et al, 1997; Grond et al, 2020; Jarvela and Linstedt, 2014; Zhang and Wang, 2015; Zhang and Seemann, 2021). We have recently identified GRASP55 as a direct mTOR complex 1 (mTORC1) substrate, establishing the mTORC1-GRASP55 signaling axis as a key metabolic sensory hub that responds to nutrient and stress stimuli to reshape the extracellular proteome accordingly (Demetriades et al, 2021; Fernandes et al, 2024; Nüchel et al, 2021).

Here we report that GRASP55, but not GRASP65, is required for proper lysosome function by controlling the trafficking and localization of lysosomal enzymes. Loss of GRASP55 expression leads to missorting and secretion of lysosomal enzymes to the cell culture media, causes lysosome dysfunction and bloating, blunts the lysosomal localization of mTORC1, as well as the phosphorylation of its lysosomal targets TFEB and TFE3, but not that of its non-lysosomal substrate 4E-BP1. Notably, our data place GRASP55 upstream of the previously described GOLPH3-LYSET-GNPTAB protein sorting machinery: GRASP55 interacts with GOLPH3 and is required for its presence at the Golgi. Consequently, GRASP55 knockout (KO) cells are characterized by mislocalization and decreased stability of both LYSET and GNPTAB, and thus defective M6P-tagging and trafficking of lysosomal enzymes. In sum, these results highlight the role of the Golgi protein sorting machinery in orchestrating Golgi–lysosome communication, lysosome function, and lysosomal mTORC1 signaling. Furthermore, because GRASP55 loss results in a cellular phenotype that is reminiscent of that caused by GNPTAB or LYSET inactivating mutations, our findings suggest that *GRASP55/GORASP2* may be a susceptibility gene in LSD-like conditions.

## Results

### GRASP55 is necessary for proper sorting and delivery of lysosomal enzymes

**Figure 1:**
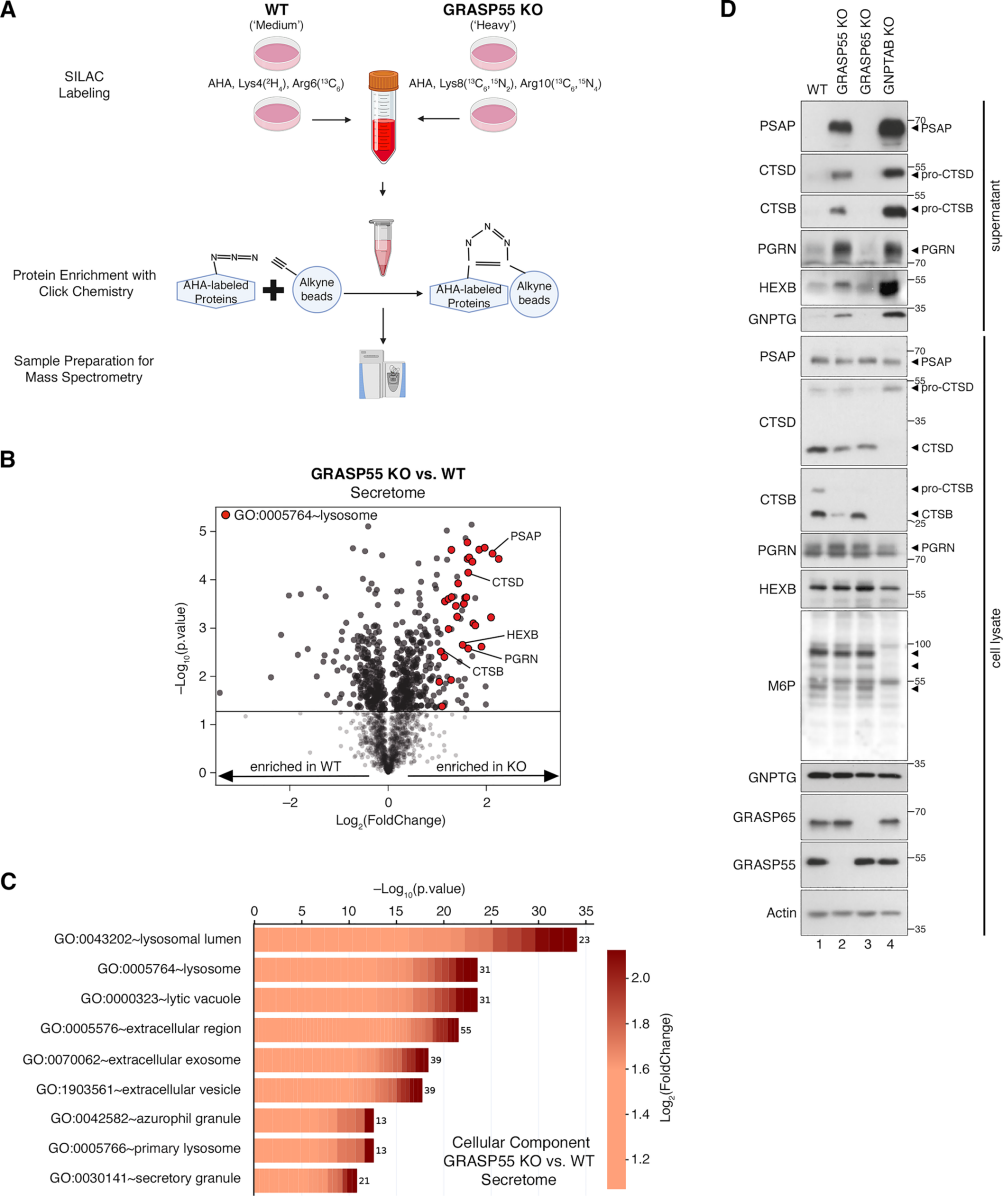
Loss of GRASP55, but not GRASP65, causes missorting of lysosomal enzymes, resembling GNPTAB loss-of-function. (**A**) Experimental outline of the SILAC labeling- and click chemistry-based assay performed to identify the GRASP55-dependent secretome in WI-26 cells (PRIDE dataset identifier: PXD020331; originally described in (Nüchel et al, 2021; Data ref: Nüchel et al, 2021); see also Methods). (**B**) Volcano plot showing all proteins identified in the GRASP55-dependent secretome (gray dots). Proteins whose secretion is strongly and significantly increased (Log_2_FC > 1, *P* < 0.05) in GRASP55 KO WI-26 cells were used for the GO analysis shown in (**C**). Proteins within this subset that belong to the cellular component (CC) GO term ‘lysosome’ are shown in red. *n* = 4 independent biological replicates, including two independent GRASP55 KO clones and swapping labeling of WT and KO cells with heavy and medium isotopes (or vice versa) to control for labeling bias. *P* values were calculated using a one-sample *t* test (Value = 0, S0 = 0.1, side = both). (**C**) CC GO term analysis using the proteins whose secretion is enhanced in GRASP55 KO cells reveals strong enrichment of lysosome-related terms. The color of each box represents log_2_-transformed fold change values for each protein in GRASP55 KO vs. WT cells. The number of proteins in the selected dataset for each term is shown on the right side of each bar. (**D**) Immunoblots with the indicated antibodies showing enhanced secretion of lysosomal enzymes in cell culture media of GRASP55 KO and GNPTAB KO, but not GRASP65 KO cells. Secretion of lysosomal proteins (PSAP, CTSD, CTSB, PGRN, HEXB) and GNPTG assayed by immunoblotting in the supernatants of WT, GRASP55 KO, GRASP65 KO, and GNPTAB KO WI-26 cells. Intracellular levels of the same proteins, and of GRASP55, GRASP65, and Actin assayed in whole-cell lysates. M6P-modification of intracellular proteins shown as control, with arrowheads indicating bands in the M6P blot that are lost in GNPTAB KO samples. *n* = 4 independent experiments. See also Fig. EV1. Source data are available online for this figure.

To gain insight into the role of GRASP55 in the reshaping of the extracellular proteome upon stress, and to reveal the GRASP55-dependent secretome, we previously conducted SILAC-labeling and mass spectrometry (MS)-based proteomics experiments using GRASP55 KO and control WI-26 human lung fibroblasts (Nüchel et al, 2021; Data ref: Nüchel et al, 2021) (Fig. 1A). Focusing on the proteins whose secretion is blunted in GRASP55 KO cells, we observed a robust enrichment of extracellular matrix (ECM)- and cell-adhesion-related factors, indicating that GRASP55 controls such cellular processes through the regulation of UPS (Demetriades et al, 2021; Nüchel et al, 2021). Intriguingly, when reanalyzing our secretome data, we noticed the presence of a group of proteins whose secretion is actually enhanced in cells lacking GRASP55 (65 significantly upregulated proteins in the GRASP55 KO cells, compared to the WT controls) (Fig. 1B; Dataset EV1). Gene ontology (GO) term analysis revealed a striking enrichment of lysosomal proteins—among other extracellular proteins—within this dataset (31 out of 65 proteins annotated within the cellular component GO term ‘lysosome’), with most of them being lysosomal enzymes, such as prosaposin (PSAP), cathepsin D (CTSD), cathepsin B (CTSB), β-hexosaminidase (HEXB), and progranulin (PGRN) (Fig. 1B,C; Dataset EV1), in line with previous work (Ahat et al, 2022; Akaaboune et al, 2025).

Because the aberrant secretion of lysosomal enzymes in GRASP55 KO cells is reminiscent of the phenotypes typically observed in ML-II, we included GNPTAB KO cells in our follow-up analyses (Velho et al, 2019). Furthermore, as GRASP65 is structurally very similar to GRASP55, and together they are involved in the maintenance of Golgi morphology (Barr et al, 1997), but is not involved in the regulation of UPS (Nüchel et al, 2021), we also generated GRASP65 KO cells to test if this abnormal secretory phenotype is specific for GRASP55 or a general characteristic of perturbed Golgi function. In line with our secretome data, immunoblotting analysis of cell culture supernatants and cell lysates from GRASP55 KO and control WI-26 cells showed strongly elevated secretion of the lysosomal enzymes PSAP, CTSD, CTSB, PGRN, and HEXB in the media of GRASP55-deficient cells, similar to that of GNPTAB KO cells (Fig. 1D). Interestingly, in addition to lysosomal enzymes, we also detected GNPTG, the soluble γ subunit of the GNPT complex, in the media of GRASP55 KO cells (and of GNPTAB KO cells) (Fig. 1D). This suggested a potential defect in the assembly and function of the GNPT complex that is necessary for M6P-tagging of lysosomal enzymes (De Pace et al, 2015; Encarnacao et al, 2011). Indeed, the levels of several M6P-modified proteins were greatly reduced in the absence of GRASP55 compared to control cells, albeit not to the extent observed in GNPTAB-deficient cells that completely lack catalytic activity (Fig. 1D). Of note, none of these effects were observed in GRASP65-deficient cells, indicating that GRASP55 controls lysosomal enzyme trafficking and GNPT activity independently of its role in Golgi morphology (Fig. 1D).

**Figure EV1:**
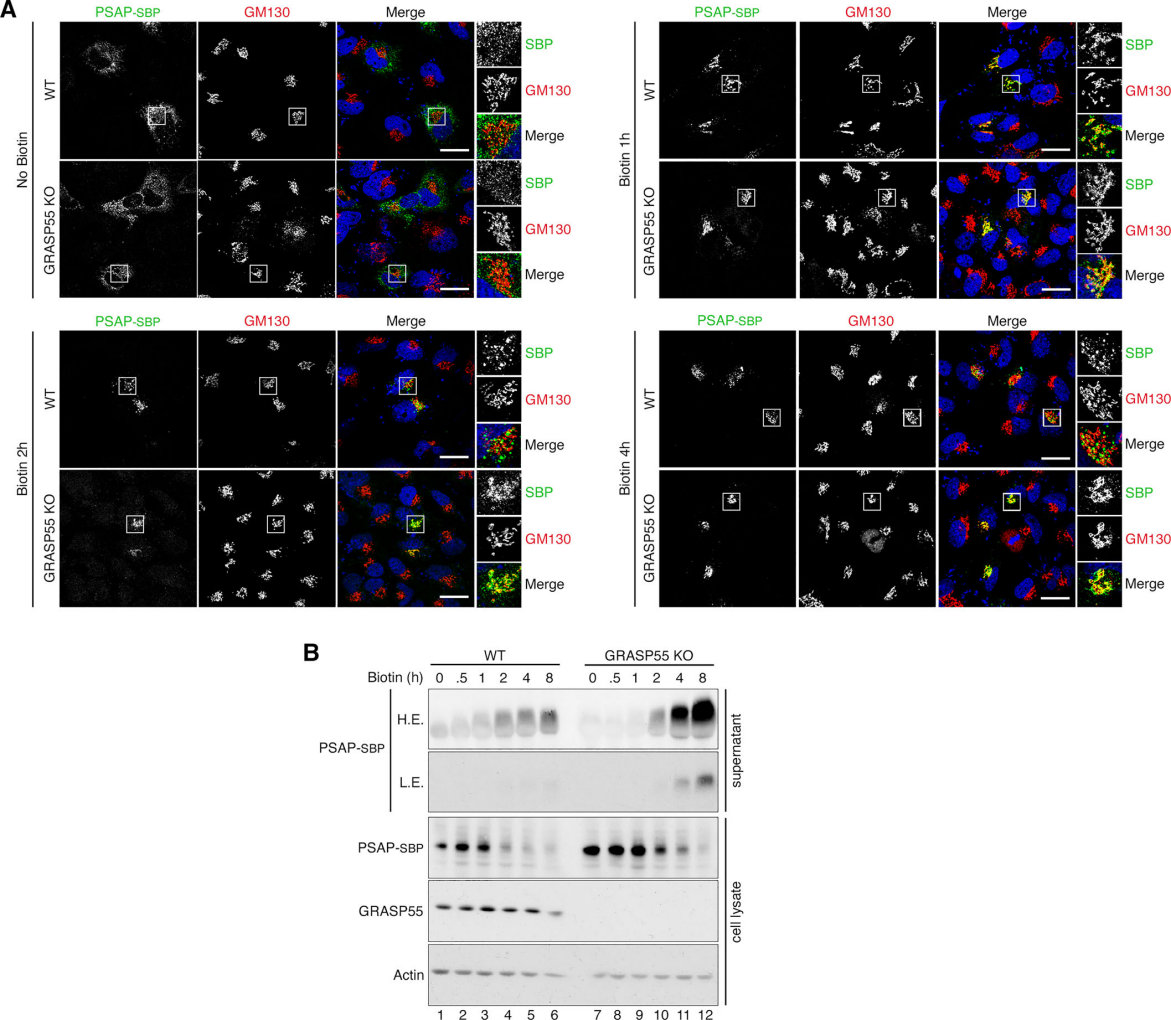
Missorting of exogenous SBP-tagged PSAP GRASP55 KO cells, detected using a RUSH assay. Related to Fig. 1. (**A**) Immunofluorescence analysis of WT or GRASP55 KO WI-26 cells transiently expressing streptavidin-binding peptide (SBP)-tagged prosaposin (PSAP) at the indicated time points following biotin addition. GM130 used as a Golgi marker. Nuclei stained with DAPI. Scale bars, 10 μm. *n* = 3 independent experiments. (**B**) Representative immunoblot analysis of cell lysates and supernatants from WT or GRASP55 KO WI-26 cells transiently expressing SBP-tagged PSAP at the indicated time points following biotin addition. GRASP55 and Actin immunoblots were used as controls. *n* = 2 independent experiments Source data are available online for this figure.

In order to investigate the dynamics of lysosomal enzyme sorting and secretion in the absence of GRASP55, we then performed synchronized trafficking experiments using the RUSH (retention using selective hooks) method (Boncompain et al, 2012), with a streptavidin-KDEL fusion protein as ER luminal hook and PSAP fused to SBP (streptavidin-binding protein). This method allows for the synchronized and rapid release of an SBP-tagged protein from the streptavidin hook by the addition of biotin in the culture medium (Boncompain et al, 2012). In both WT and GRASP55-deficient cells, PSAP-SBP localized in the ER in the absence of biotin. In WT cells, PSAP-SBP was transported to the Golgi apparatus within 1 h after biotin addition and was sorted into vesicular structures that did not show substantial overlap with the Golgi at 2–4 h following biotin addition (Fig. EV1A). In contrast, whereas the ER-Golgi trafficking of PSAP-SBP was also observed in GRASP55-deficient cells within 1 h following biotin addition, the Golgi localization of PSAP-SBP did not change further at later time-points (Fig. EV1A), indicating a potential sorting defect in GRASP55 KOs. Therefore, we performed a biotin addition time-course looking at the secretion of PSAP-SBP into the medium of GRASP55 or control cells. As with our experiments assessing the steady-state levels of endogenous PSAP in cell culture supernatants (Fig. 1D), also in RUSH assays PSAP-SBP secretion was strongly elevated in GRASP55-deficient cells, compared to controls (Fig. EV1B).

**Figure 2:**
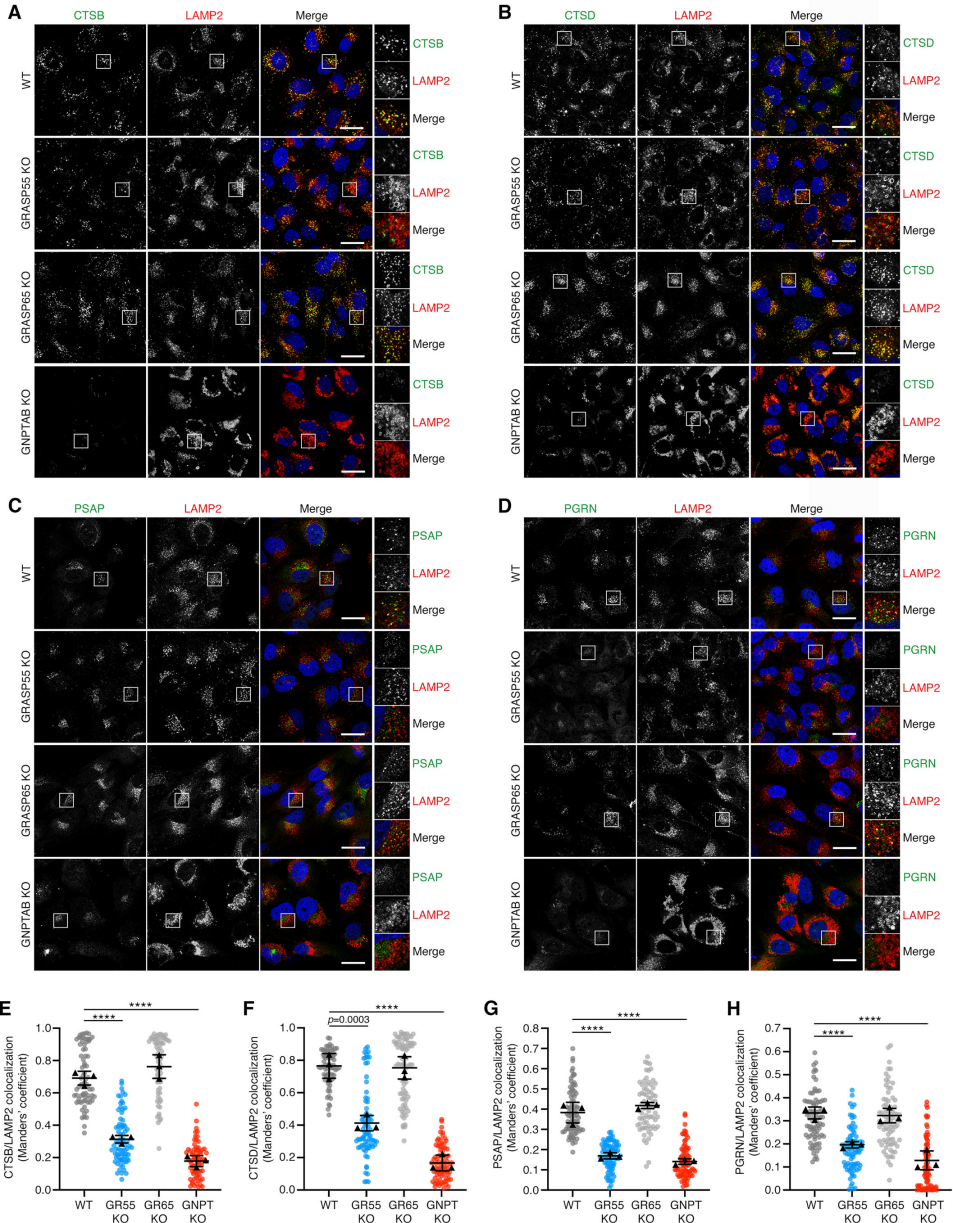
Reduced levels of lysosomal enzymes in lysosomes of GRASP55 KO cells, similar to GNPTAB KO cells. (**A**–**D**) Colocalization analysis of CTSB (**A**), CTSD (**B**), PSAP (**C**), or PGRN (**D**) with LAMP2 (lysosomal marker) in WT, GRASP55 KO, GRASP65 KO, and GNPTAB KO WI-26 cells, using confocal microscopy. Nuclei stained with DAPI (blue). Magnified insets shown to the right. Scale bars, 10 μm. (**E**–**H**) Quantification of colocalization from (**A**–**D**). Merged data from three independent experiments are shown. *n* = 70–82 individual cells from five independent fields per genotype per experiment. Data in graphs shown as mean ± SD. ****P < 0.0001; *P* values ≥ 0.0001 are shown directly in the figure (one-way ANOVA). Source data are available online for this figure.

Next, we assessed the role of GRASP55 in the subcellular localization of lysosomal enzymes using confocal microscopy and colocalization analysis of CTSB, CTSD, PSAP, and PGRN with the lysosomal membrane protein LAMP2. Whereas all four proteins colocalized strongly with LAMP2 in control cells, their lysosomal localization was strongly decreased in both GRASP55- and GNPTAB-deficient cells (Fig. 2A–H). Again, this effect was specific for GRASP55, as loss of GRASP65 had no effect in the localization of these enzymes (Fig. 2A–H).

### Cells lacking GRASP55 expression display an LSD-like lysosomal signature

**Figure 3:**
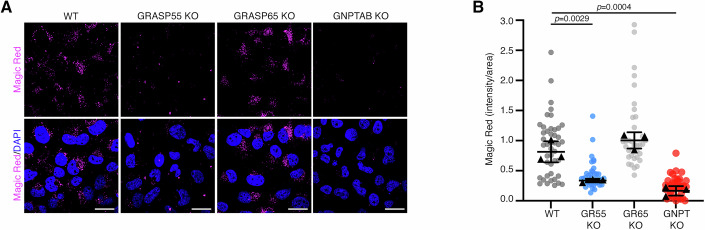
GRASP55-deficient cells contain dysfunctional lysosomes. (**A**, **B**) Lysosomal cathepsin B (CTSB) activity was monitored using a Magic Red fluorescent assay in WT, GRASP55 KO (GR55 KO), GRASP65 KO (GR65 KO), or GNPTAB KO (GNPT KO) WI-26 cells. The punctate signal is proportional to CTSB proteolytic activity inside lysosomes. Nuclei stained with DAPI. Scale bars, 10 μm (**A**). Quantification of Magic Red signal (intensity/area) in (**B**). Merged data from three independent experiments are shown. *n* = 37–45 cells from three independent fields per genotype per experiment. Data in (**B**) shown as mean ± SD. *P* values are shown directly in the figure (one-way ANOVA). See also Fig. EV2. Source data are available online for this figure.

**Figure EV2:**
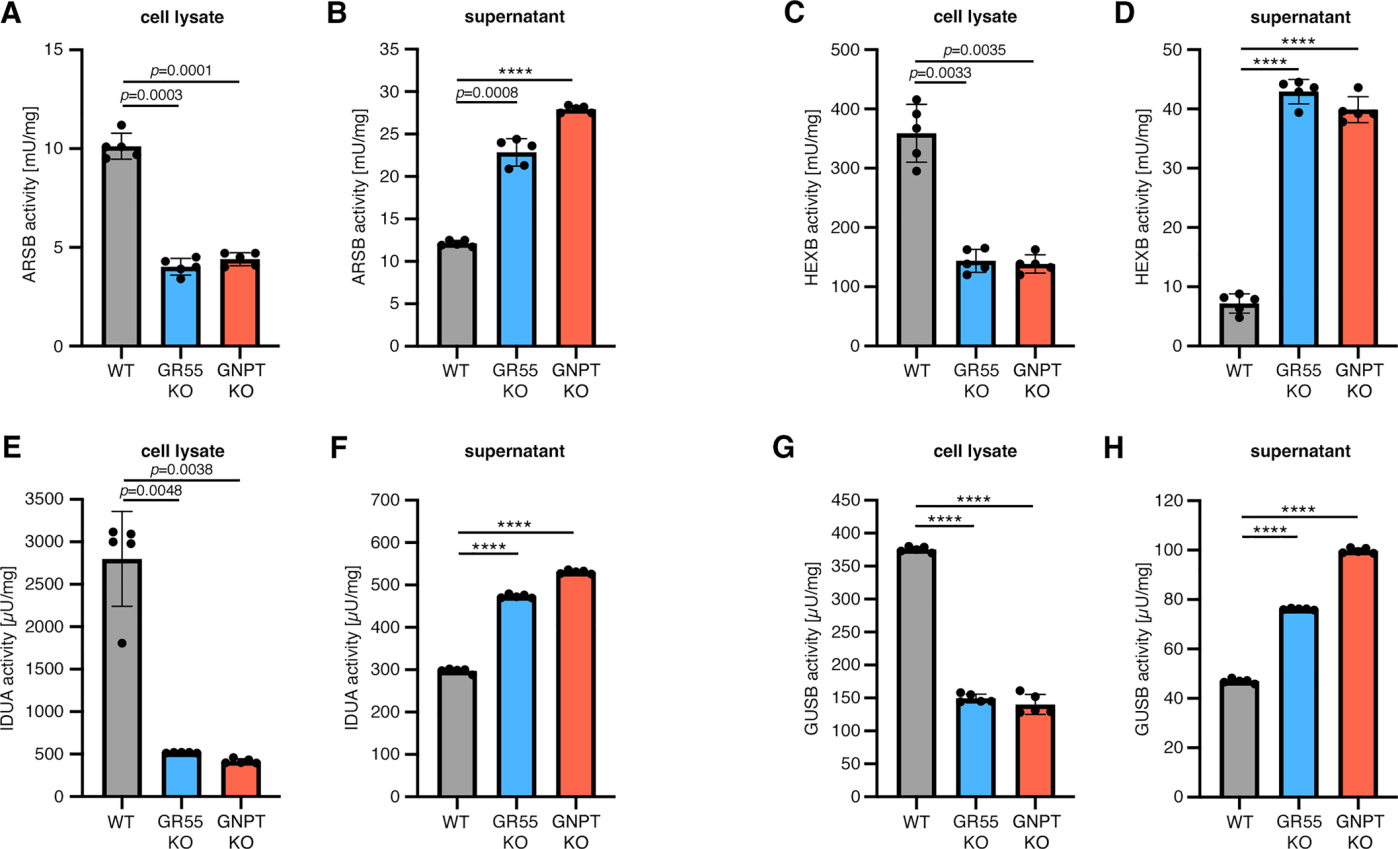
GRASP55 KO cells exhibit decreased intracellular and elevated extracellular lysosomal enzyme activity, similar to GNPTAB KO cells. Related to Fig. 3. (**A**, **B**) The enzymatic activity of arylsulfatase B (ARSB) was assayed in cell lysates (**A**) or supernatants (**B**) of wild-type (WT), GRASP55 KO (GR55 KO), or GNPTAB KO (GNPT KO) WI-26 cells (as a cellular model of mucolipidosis type II). (**C**, **D**) As in (**A**, **B**), but for β-hexosaminidase (HEXB) activity. (**E**, **F**) As in (**A**, **B**), but for α-L-iduronidase (IDUA) activity. (**G**, **H**) As in (**A**, **B**), but for β-glucuronidase (GUSB) activity. For all panels, *n* = 5 independent measurements from four biological replicates. Data in graphs shown as mean ± SD. ****P < 0.0001; *P* values ≥ 0.0001 are shown directly in the figures (one-way ANOVA) Source data are available online for this figure.

The lysosomal storage disease ML-II, caused by mutations in *GNPTAB*, is typically characterized by the presence of enlarged and defective lysosomes in the diseased cells. More specifically, the inability of lysosomal enzymes to reach their destination leads to the accumulation of non-degraded lysosomal content, which in turn causes an expansion of the lysosomal area and lysosomal bloating. In line with this, we observed diminished lysosomal activity (assayed by Magic Red, a fluorescence-based CTSB activity assay) in GRASP55- and GNPTAB-deficient cells, but not in GRASP65 KO cells (Fig. 3A,B). In addition, using enzymatic activity assays, we detected blunted arylsulfatase B (ARSB), β-hexosaminidase (HEXB), α-L-iduronidase (IDUA), and β-glucuronidase (GUSB) activities in the lysates of GRASP55 and GNPTAB KO cells, accompanied by significantly elevated activities in the respective supernatants (Fig. EV2A–H).

**Figure 4:**
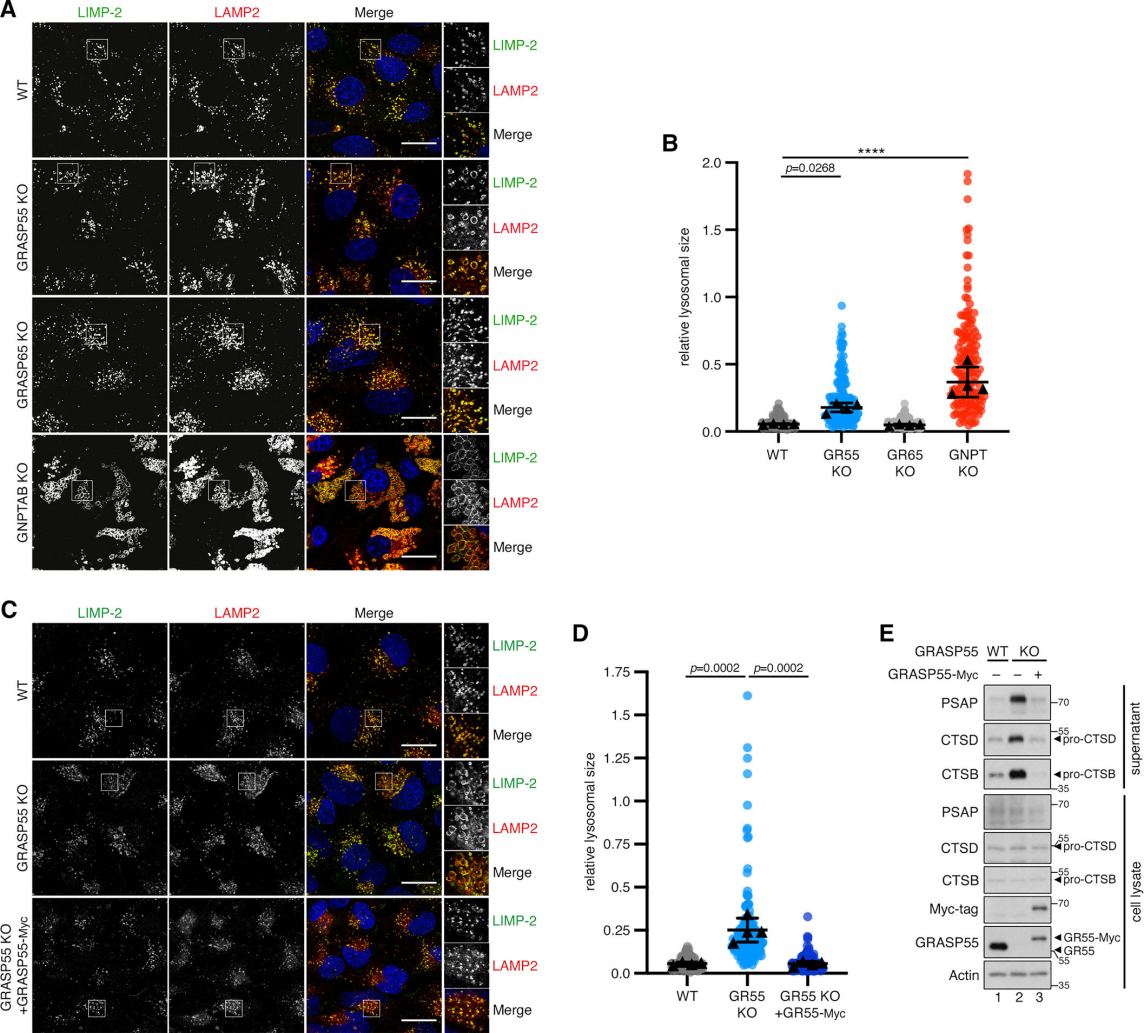
Loss of GRASP55 expression causes lysosomal bloating. (**A**, **B**) Enlarged lysosome size in GRASP55 KO and GNPTAB KO, but not in GRASP65 KO cells. Immunofluorescence analysis of LIMP-2 and LAMP2 (lysosomal membrane markers) in WT, GRASP55 KO, GRASP65 KO, and GNPTAB KO WI-26 cells, using confocal microscopy. Nuclei stained with DAPI (blue). Magnified insets shown to the right (**A**). Quantification of relative lysosomal size in (**B**). Merged data from four independent experiments are shown. *n* = 60–81 individual cells from five independent fields per genotype per experiment. (**C**, **D**) The enlarged lysosome size is reversed by re-expression of GRASP55 in GRASP55 KO cells. Immunofluorescence analysis of LIMP-2 and LAMP2 (lysosomal membrane markers) in WT, GRASP55 KO, and GRASP55 KO WI-26 cells stably expressing Myc-tagged GRASP55, using confocal microscopy. Nuclei stained with DAPI (blue). Magnified insets shown to the right (**C**). Quantification of relative lysosomal size in (**D**). Merged data from four independent experiments are shown. *n* = 50–64 individual cells from five independent fields per genotype per experiment. (**E**) The aberrant lysosomal enzyme secretion is reversed by re-expression of GRASP55 in GRASP55 KO cells. Secretion of lysosomal enzymes (PSAP, CTSD, CTSB) assayed by immunoblotting in the supernatants of WT, GRASP55 KO, and GRASP55 KO WI-26 cells stably expressing Myc-tagged GRASP55. Intracellular levels of PSAP, CTSD, CTSB, GRASP55, and Actin assayed in whole-cell lysates. *n* = 3 independent experiments. Scale bars, 10 μm. Data in graphs shown as mean ± SD. ****P < 0.0001; *P* values ≥ 0.0001 are shown directly in the figure (one-way ANOVA). See also Fig. EV3. Source data are available online for this figure.

Unlike soluble lysosomal enzymes, lysosomal transmembrane proteins do not depend on MPR-dependent pathways for their transportation to lysosomes (Ecard et al, 2024; Pols et al, 2013), therefore we next assessed lysosomal morphology using confocal microscopy and immunofluorescence-based detection of LIMP-2 (lysosomal integral membrane protein-2) and LAMP2 (lysosome-associated membrane protein 2). Indeed, both LIMP-2 and LAMP2 showed a typical punctate lysosomal-like pattern in WT cells, with near-perfect colocalization between the two proteins (Fig. 4A). Further indicating that loss of GRASP55 triggers an LSD-like phenotype, GRASP55-deficient cells contained strongly enlarged lysosomes (Fig. 4A,B), although the effect was less pronounced compared to that observed in GNPTAB KO cells (Fig. 4A,B). Of note, this lysosomal bloating was not observed in GRASP65 KO cells (Fig. 4A,B), underscoring a specific role for GRASP55 in this process.

**Figure EV3:**
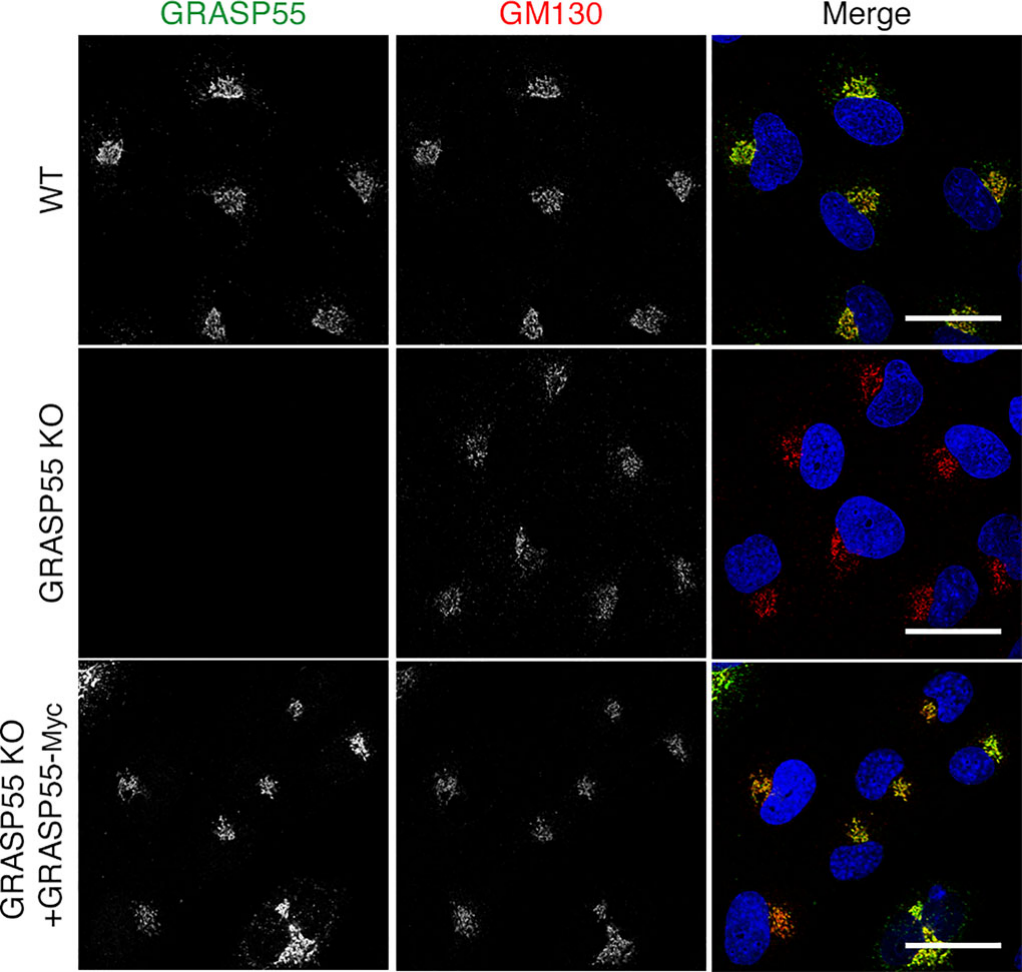
Stably-expressed Myc-tagged GRASP55 localizes at the Golgi, resembling endogenous GRASP55 localization. Related to Fig. 4. Immunofluorescence analysis of WT or GRASP55 KO WI-26 cells, or GRASP55 KO cells stably re-expressing Myc-tagged GRASP55 at near-endogenous levels. GM130 used as a Golgi marker. Nuclei stained with DAPI (blue). Scale bars, 10 μm. *n* = 2 independent experiments Source data are available online for this figure.

To ensure that the abnormal lysosomal phenotype is linked to GRASP55 expression, we next reconstituted the GRASP55 KO WI-26 cells by stably re-expressing Myc-tagged GRASP55. Exogenously expressed GRASP55 localized properly at the Golgi, similarly to endogenous GRASP55 localization in WT cells (Fig. EV3). Importantly, reconstitution of GRASP55 expression fully reversed the lysosomal bloating (Fig. 4C,D) and the lysosomal enzyme secretion phenotypes (Fig. 4E) observed in GRASP55 KO cells. In sum, these data indicate the specific role of GRASP55 in the sorting and trafficking of lysosomal enzymes and in the maintenance of proper lysosome function, while GRASP55 deletion resembles the LSD-like phenotypes that are driven by loss of GNPTAB expression.

### Lysosomal dysfunction upon loss of GRASP55 selectively perturbs lysosomal mTORC1 signaling

**Figure 5:**
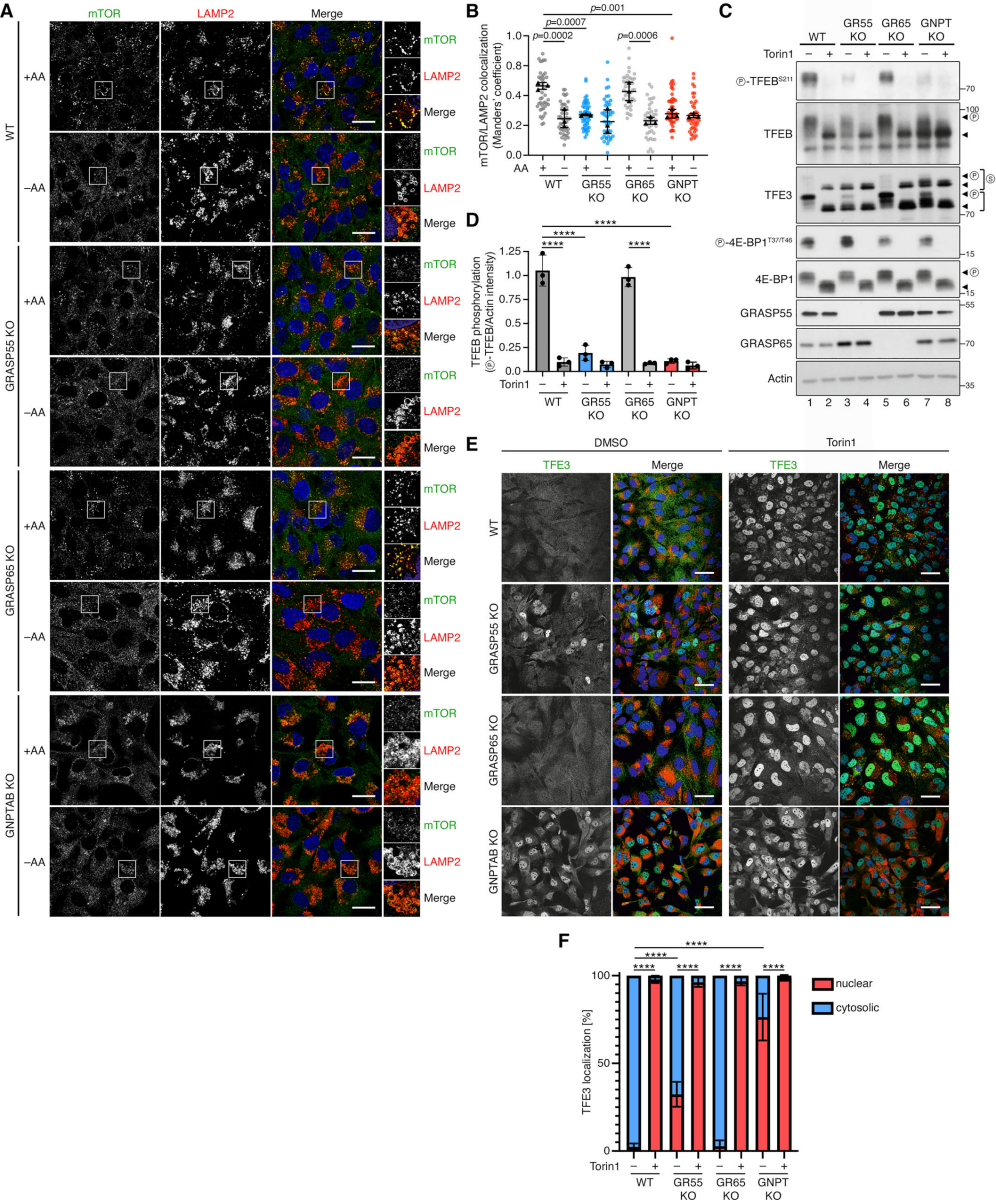
Lysosomal dysfunction in GRASP55 and GNPTAB KO cells specifically perturbs lysosomal mTORC1 signaling. (**A**, **B**) Loss of lysosomal mTOR localization in GRASP55 KO and GNPTAB KO cells. Colocalization analysis of mTOR with LAMP2 (lysosomal marker) in WT, GRASP55 KO, GRASP65 KO, and GNPTAB KO WI-26 cells, treated with media containing ( + AA) or lacking AAs (–AA) for 2 h, using confocal microscopy. Nuclei stained with DAPI (blue). Magnified insets shown to the right. Scale bars, 10 μm (**A**). Quantification of colocalization in (**B**). Merged data from three independent experiments are shown. *n* = 60 individual cells from five independent fields per genotype per experiment. (**C**, **D**) Diminished phosphorylation of TFEB/TFE3, but not 4E-BP1, in GRASP55 KO or GNPTAB KO cells. Immunoblots with lysates from WT, GRASP55 KO, GRASP65 KO, and GNPTAB KO WI-26 cells, treated with DMSO (vehicle) or Torin1 (250 nM) for 2 h, and probed with the indicated antibodies (**C**). Quantification of TFEB phosphorylation (p-TFEB/Actin ratio) in (**D**). Arrowheads indicate bands corresponding to different protein forms, when multiple bands are present. P phosphorylated form, S SUMOylated form. *n *= 3 independent experiments. (**E**, **F**) Nuclear translocation of TFE3 in GRASP55 KO or GNPTAB KO cells. TFE3 localization analysis in WT, GRASP55 KO, GRASP65 KO, and GNPTAB KO WI-26 cells, treated with DMSO (vehicle) or Torin1 (250 nM) for 2 h, using confocal microscopy. LAMP2 used as lysosomal marker (red). Nuclei stained with DAPI (blue). Magnified insets shown to the right. Scale bars, 20 μm (**E**). Quantification of nuclear/cytosolic TFE3 localization (%) in (**F**). Merged data from four independent experiments are shown. *n* = 80–118 individual cells from five independent fields per genotype per experiment. Data in graphs shown as mean ± SD. ****P < 0.0001; *P* values ≥ 0.0001 are shown directly in the figure (two-way ANOVA). Source data are available online for this figure.

Active mTORC1 controls virtually all cellular functions through the phosphorylation of multiple effector proteins that takes place at various subcellular locations (Fernandes and Demetriades, 2021; He et al, 2024). For instance, while mTORC1 regulates protein synthesis by phosphorylating S6K and 4E-BP1 in the cytoplasm, it phosphorylates TFEB and TFE3 on the lysosomal surface to prevent their nuclear translocation and block lysosome biogenesis (Fernandes et al, 2024; Settembre et al, 2012). Furthermore, we recently showed that pharmacological or genetic blockage of basal lysosomal proteolysis (e.g., by BafA1 treatment; or in GNPTAB KO cells) specifically affects the phosphorylation of lysosomal mTORC1 substrates, as it causes its relocalization away from the lysosomal surface without downregulating its activity towards its non-lysosomal substrates (Fernandes et al, 2024). Therefore, we next assessed the localization and activity of mTORC1 in GRASP55 KO cells. As observed previously in GNPTAB KO HEK293FT cells (Fernandes et al, 2024), the lysosomal localization of mTOR was diminished in GRASP55 KO and GNPTAB KO, but not in GRASP65 KO cells, resembling the pattern seen upon treatment with amino acid (AA) starvation media (Fig. 5A,B). Accordingly, loss of GRASP55 or GNPTAB expression blunted the phosphorylation of the lysosomal mTORC1 substrates TFEB and TFE3, but not that of the cytoplasmic substrate 4E-BP1 (Fig. 5C). As a control, treatment with Torin1, an ATP-competitive mTOR inhibitor, diminished the phosphorylation of all mTORC1 substrates (Fig. 5C). Consistent with the changes in its phosphorylation, we observed enhanced nuclear translocation of TFE3 in GRASP55 KO and GNPTAB KO cells (as in all Torin1-treated cells), but not in GRASP65 KOs (Fig. 5E,F). Taken together, these data show that the defect in lysosomal function that is observed in GRASP55- and GNPTAB-deficient cells selectively perturbs lysosomal mTORC1 signaling without affecting its activity towards a cytoplasmic target such as 4E-BP1.

### GRASP55 controls GNPTAB localization and protein stability

**Figure 6:**
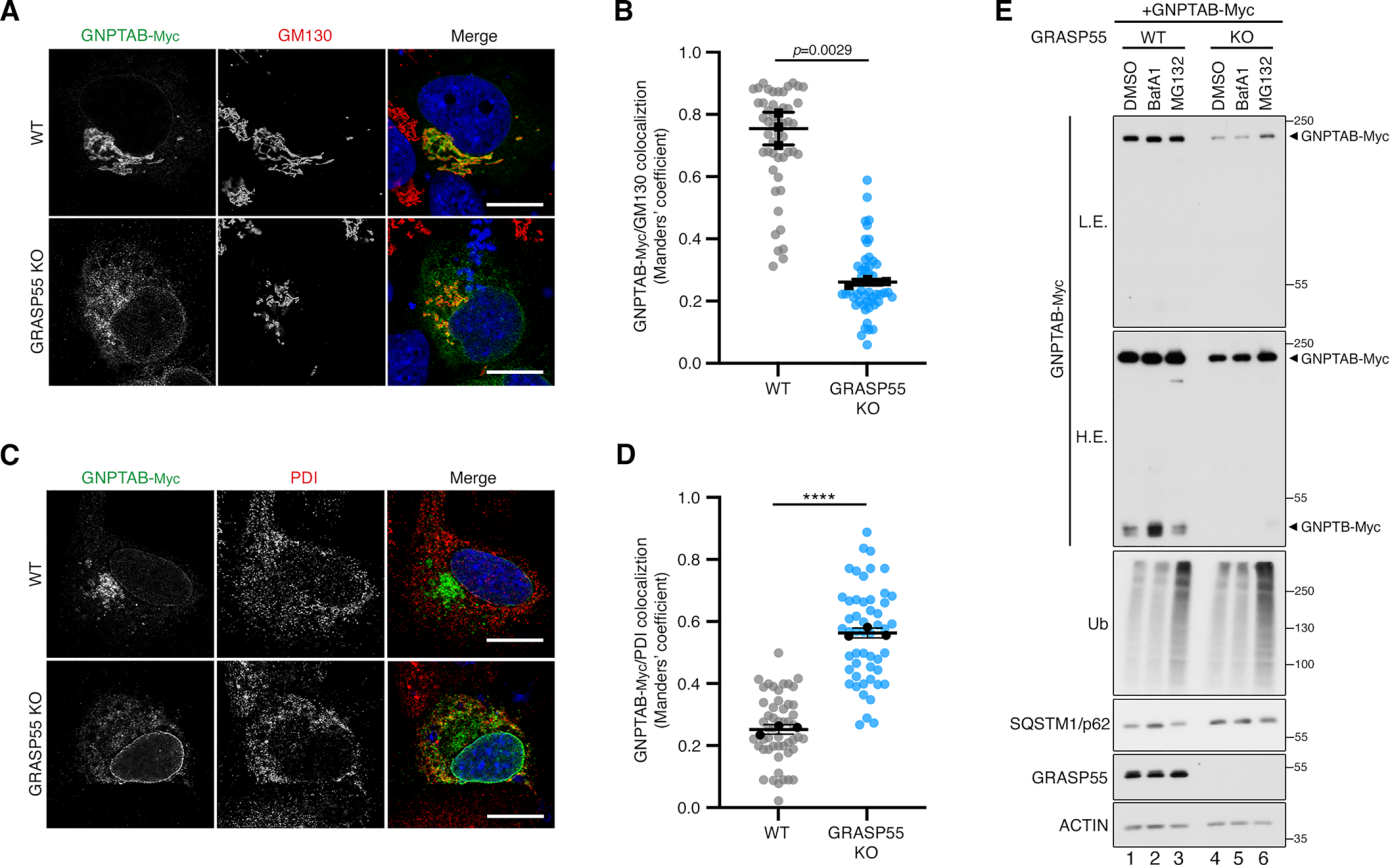
GRASP55 is required for proper GNPTAB localization and stability. (**A**, **B**) Blunted Golgi localization of GNPTAB in GRASP55 KO cells. Colocalization analysis of exogenously expressed Myc-tagged GNPTAB with GM130 (Golgi marker) in WT or GRASP55 KO WI-26 cells, using confocal microscopy. Nuclei stained with DAPI (blue) (**A**). Quantification of colocalization in (**B**). Merged data from three independent experiments are shown. *n* = 50 individual cells from five independent fields per genotype per experiment. (**C**, **D**) Aberrant ER localization of GNPTAB in GRASP55 KO cells. Experiment as in (**A**, **B**), but for GNPTAB colocalization with PDI (ER marker) (**C**). Quantification of colocalization in (**D**). Merged data from three independent experiments are shown. *n* = 51 individual cells from five independent fields per genotype per experiment. (**E**) Decreased stability of GNPTAB is rescued by proteasomal inhibition. Immunoblots with lysates from WT or GRASP55 KO WI-26 cells, transiently expressing Myc-tagged GNPTAB, treated with DMSO (vehicle), BafA1 (100 nM, 8 h), or MG132 (20 μM, 8 h), and probed with the indicated antibodies. L.E. low exposure, H.E. high exposure. *n* = 3 independent experiments. Scale bars, 10 μm. Data in graphs shown as mean ± SD. ****P < 0.0001; *P* values ≥ 0.0001 are shown directly in the figure (Student’s *t* test). Source data are available online for this figure.

We next aimed to investigate the underlying cause for the missorting of lysosomal enzymes in GRASP55-deficient cells. As we observed an overall reduction of M6P protein modification as well as abnormal secretion of GNPTG in GRASP55 KO cells (Fig. 1D), we focused our analysis on GNPTAB, the catalytic part of the GNPT complex. As expected, exogenously expressed, Myc-tagged GNPTAB showed a near-perfect colocalization with the *cis*-Golgi marker GM130 (Nakamura et al, 1995) in control cells (Fig. 6A). Interestingly, however, loss of GRASP55 led to strongly reduced Golgi localization of GNPTAB (Fig. 6A,B), accompanied by increased colocalization with PDI (Protein disulfide-isomerase), a marker of the ER lumen (Wilkinson and Gilbert, 2004) (Fig. 6C,D), indicating that GRASP55 is necessary for maintaining GNPTAB at the Golgi apparatus.

At the *cis*-Golgi, the GNPTAB precursor protein undergoes a maturation process, activated through proteolytic cleavage by S1P to the enzymatically active GNPTA and GNPTB subunits (Marschner et al, 2011). Indeed, in control WI-26 cells, a fraction of the exogenously expressed GNPTAB was processed, as shown by the presence of the cleaved Myc-tagged GNPTB (Fig. 6E). In contrast, consistent with the mislocalization and reduced Golgi presence of GNPTAB in GRASP55 KO cells, we also observed defective GNPTAB processing (Fig. 6E, lanes 1 and 4). Strikingly, these experiments also revealed reduced protein levels of Myc-tagged GNPTAB in GRASP55 KO cells (Fig. 6E, lanes 1 and 4), which was partially rescued by treatment with the proteasome inhibitor MG132, but not with the lysosome inhibitor BafA1 (Fig. 6E, lanes 5–7). In sum, these results indicate that GRASP55 is necessary for proper retention and processing of GNPTAB at the Golgi. Moreover, in GRASP55-deficient cells, GNPTAB is mislocalized to the ER where it is targeted for degradation, in part through proteasome-mediated mechanisms.

### GRASP55 controls GOLPH3-LYSET localization at the Golgi and LYSET stability

**Figure 7:**
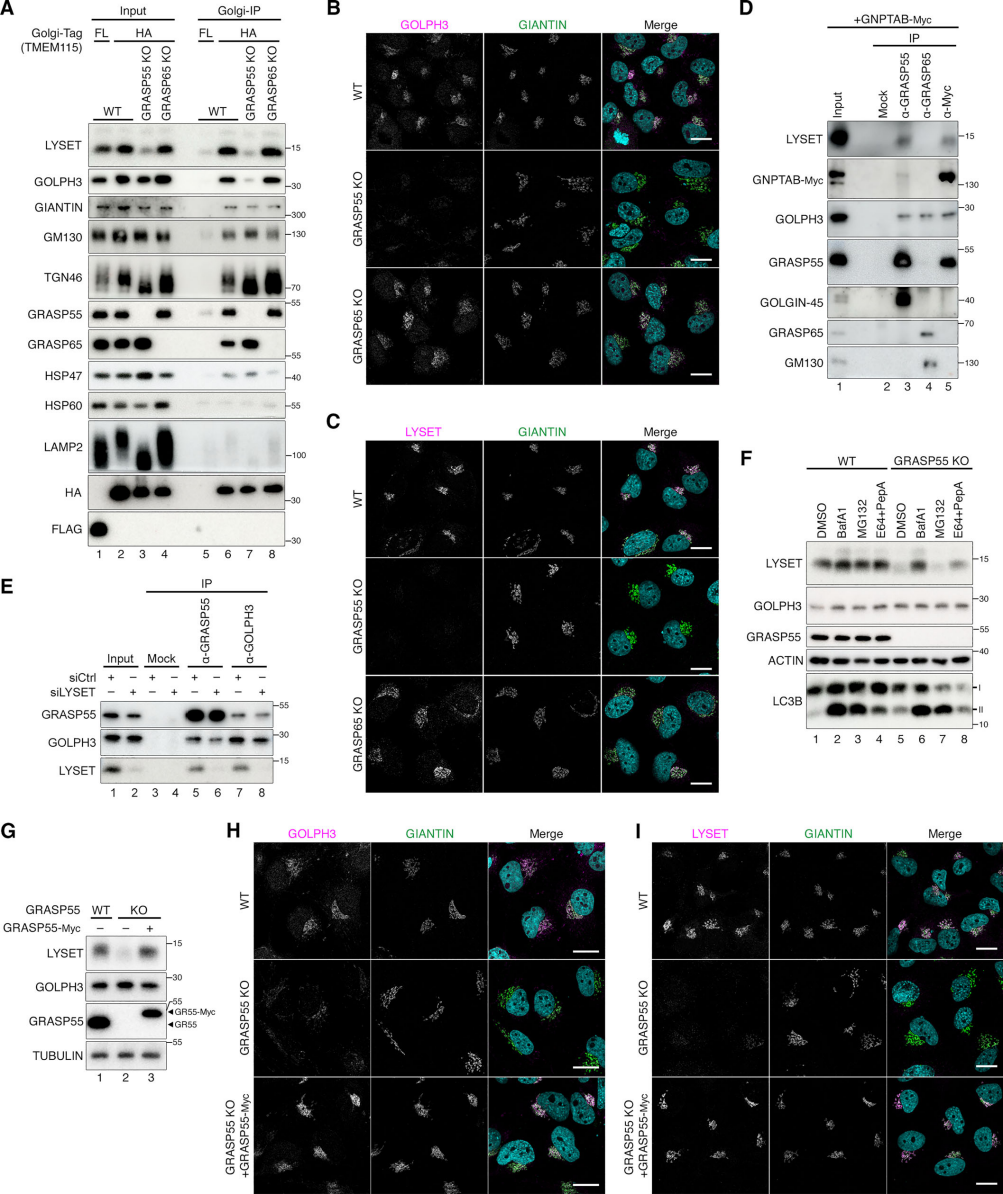
GRASP55 is required for GOLPH3-LYSET localization at the Golgi and LYSET stability. (**A**) Diminished Golgi localization of GOLPH3 and LYSET in GRASP55 KO cells. Immunoblots with Golgi-IP samples or whole-cell lysates (‘Input’) from WT, GRASP55 KO and GRASP65 KO WI-26 cells, stably expressing TMEM115-2xHA (Golgi-Tag), or TMEM115-2xFLAG (negative control; only in WT), probed with the indicated antibodies. GIANTIN, GM130, TGN46 (Golgi), HSP47 (ER-Golgi), HSP60 (mitochondria) and LAMP2 (lysosomes) used as markers for the presence of various organelles in the Golgi-IP fractions. Note also the destabilization of LYSET in the input samples of GRASP55 KO cells. *n* = 3 independent experiments. (**B**, **C**) Blunted Golgi localization of GOLPH3 and LYSET in GRASP55 KO, but not in GRASP65 KO cells. Colocalization analysis of endogenous GOLPH3 (**B**) or LYSET (**C**) with GIANTIN (Golgi marker) in WT, GRASP55 KO, or GRASP65 KO WI-26 cells, using confocal microscopy. Nuclei stained with DAPI (blue). Scale bars, 10 μm. *n* = 3 independent experiments. (**D**) GRASP55, but not GRASP65, acts in a complex with GOLPH3, GNPTAB and LYSET. Co-IP analysis of endogenous GRASP55, GRASP65, and exogenously expressed Myc-tagged GNPTAB proteins in WI-26 cells. The input and IP samples were analyzed by immunoblotting using antibodies against the indicated proteins. The GRASP55 interactor GOLGIN-45 and the GRASP65 interactor GM130 were used as positive controls. *n* = 3 independent experiments. (**E**) GRASP55 binds GOLPH3 independently of LYSET. Co-IP analysis of endogenous GRASP55 and GOLPH3 proteins in WI-26 cells transiently transfected with siRNAs against *LYSET* or a control siRNA duplex. The input and IP samples were analyzed by immunoblotting using antibodies against the indicated proteins. *n* = 2 independent experiments (**F**) Lysosome inhibition prevents LYSET degradation in GRASP55 KO cells. Immunoblots with lysates from WT or GRASP55 KO WI-26 cells treated with DMSO (vehicle), BafA1 (100 nM), MG132 (10 μM), or E64 (25 μM) + Pepstatin A (50 μM) for 8 h, probed with the indicated antibodies. *n* = 3 independent experiments. (**G**) LYSET degradation is rescued by GRASP55 re-expression in GRASP55 KO cells. Immunoblots with lysates from WT, GRASP55 KO or GRASP55 KO WI-26 cells stably reconstituted with GRASP55-Myc probed with the indicated antibodies. *n* = 2 independent experiments. (**H**, **I**) Blunted Golgi localization of GOLPH3 and LYSET is rescued by re-expression of GRASP55 in GRASP55 KO cells. Colocalization analysis of GOLPH3 (**H**) or LYSET (**I**) with GIANTIN (Golgi marker) in WT, GRASP55 KO, or GRASP55 KO WI-26 cells stably expressing Myc-tagged GRASP55, using confocal microscopy. Nuclei stained with DAPI (blue). Scale bars, 10 μm. *n* = 3 independent experiments. Source data are available online for this figure.

The stability and localization of GNPTAB at the Golgi is facilitated through the coordinated action of the GOLPH3 and LYSET proteins (Brauer et al, 2024; Pechincha et al, 2022; Richards et al, 2022). As these processes were dysregulated in GRASP55 KO cells, we hypothesized that GRASP55 may be acting upstream or in parallel to the GOLPH3-LYSET machinery. To test this, we performed Golgi-IP experiments comparing WT, GRASP55 KO and GRASP65 KO fibroblast cell lines, stably expressing a Golgi-Tag (Fasimoye et al, 2023). As expected, several Golgi-resident proteins—such as GOLPH3, LYSET, GIANTIN, GM130, TGN46, GRASP55, and GRASP65—were robustly enriched in the Golgi-IP samples from WT cells, whereas markers of other organelles were strongly de-enriched (Fig. 7A). Intriguingly, both GOLPH3 and LYSET were specifically depleted from Golgi-IP samples derived from GRASP55 KO cells (Fig. 7A). The absence of GOLPH3 and LYSET from the Golgi of GRASP55-deficient cells was also confirmed by confocal microscopy (Fig. 7B,C), suggesting that GRASP55 may be required to maintain their proper localization. Further supporting this hypothesis, endogenous GRASP55 interacted with GOLPH3, LYSET, and exogenously expressed GNPTAB in co-immunoprecipitation (co-IP) experiments (Fig. 7D). While we detected binding also between GOLPH3 and GRASP65, the latter did not interact with LYSET or GNPTAB, hinting at the existence of a separate GRASP65-GOLPH3 complex of unknown function, that is independent of the presumed GRASP55-GOLPH3-LYSET-GNPTAB complex (Fig. 7D). Furthermore, the binding between GRASP55-GOLPH3 persisted also in LYSET-depleted cells (Fig. 7E), suggesting that GRASP55 may co-IP LYSET and GNPTAB indirectly, via interactions with GOLPH3.

Interestingly, LYSET protein levels were strongly depleted, not only in the Golgi-IP samples, but also in whole-cell lysates (‘inputs’) of GRASP55 KO cells (Fig. 7A), suggesting that its mislocalization away from the Golgi may be linked to its overall destabilization. Indeed, LYSET levels were fully restored in GRASP55-deficient cells upon treatment with BafA1, and partially upon treatment with lysosomal protease inhibitors (but not upon proteasome inhibition), indicating that loss of GRASP55 leads to lysosome-dependent degradation of LYSET (Fig. 7F). These findings are in line with recent work describing the mislocalization and lysosomal degradation of LYSET in GOLPH3/GOLPH3L-deficient cells (Brauer et al, 2024). Of note, GOLPH3 levels were largely unaffected in GRASP55 KOs, or upon inhibition of lysosome or proteasome activities, showing that loss of GRASP55 specifically perturbs its Golgi localization without impacting its stability (Fig. 7F). Finally, LYSET protein levels (Fig. 7G) and the Golgi localization of GOLPH3 and LYSET (Fig. 7H,I) were fully rescued upon re-expression of GRASP55 in the GRASP55 KO cells. Collectively, our data suggest that GRASP55 controls the localization of GOLPH3 at the Golgi, which in turn is responsible for the stabilization of LYSET at this location and for preventing its missorting and lysosomal degradation.

### GRASP55 controls GNPTAB localization in a LYSET-dependent manner

**Figure 8:**
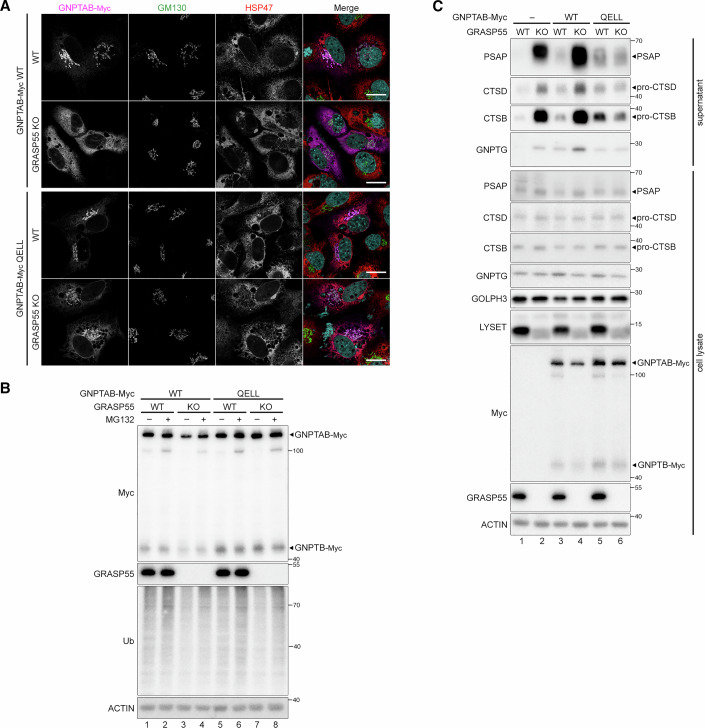
GRASP55 controls GNPTAB localization in a LYSET-dependent manner. (**A**) The GNPTAB^QELL^ mutant localizes at the Golgi independently of GRASP55. Immunofluorescence analysis of WI-26 cells transiently expressing Myc-tagged GNPTAB (WT or the LYSET-independent QELL mutant). GM130 and HSP47used as Golgi and ER markers, respectively. Nuclei stained with DAPI (cyan). Scale bars, 10 μm. *n* = 2 independent experiments. (**B**) The GNPTAB^QELL^ mutant shows enhanced stability and processing even in GRASP55 KO cells. Immunoblot analysis with lysates from WT and GRASP55 KO WI-26 cells transiently expressing Myc-tagged GNPTAB (WT or the QELL mutant), treated with DMSO (vehicle) or MG132 (10 μM, 8 h), and probed with the indicated antibodies. *n* = 2 independent experiments. (**C**) Exogenous expression of the GNPTAB^QELL^ mutant rescues trafficking of lysosomal enzymes in GRASP55 KO cells. Secretion of lysosomal proteins (PSAP, CTSD, CTSB) and GNPTG assayed by immunoblotting in the supernatants of WT and GRASP55 KO WI-26 cells transiently expressing Myc-tagged GNPTAB (WT or the QELL mutant) or transfected with an empty vector (–) as a control. Intracellular levels of the same proteins, and of GRASP55, GOLPH3, LYSET, GNPTAB-Myc, and Actin assayed in whole-cell lysates. *n* = 3 independent experiments. Source data are available online for this figure.

Our findings position GRASP55 upstream of the GOLPH3-LYSET complex in the regulation of GNPTAB localization and for the proper sorting of lysosomal enzymes at the Golgi. To test this hypothesis, we took advantage of a double GNPTAB mutant (Q36L/E39L; hereafter called ‘QELL’) that was previously described to localize at the Golgi in a LYSET-independent manner (Doray et al, 2025; Pechincha et al, 2022). Indeed, in confocal microscopy experiments, the GNPTAB^QELL^ mutant localized predominantly at the Golgi also in GRASP55 KO cells (Fig. 8A), unlike GNPTAB^WT^ that localized at the Golgi in a GRASP55-dependent fashion (Figs. 6A–D and  8A). Accordingly, also the protein levels and processing of the GNPTAB^QELL^ mutant were normal even in GRASP55-deficient cells (Fig. 8B). Finally, exogenous expression of GNPTAB^QELL^, but not of GNPTAB^WT^, robustly rescued the abnormal secretion of lysosomal enzymes and the soluble GNPTG subunit to the extracellular space of GRASP55 KO cells (Fig. 8C). Therefore, the GNPTAB^QELL^ mutant is stable, functional and localizes properly at the Golgi, even in cells lacking GRASP55 expression.

**Figure 9:**
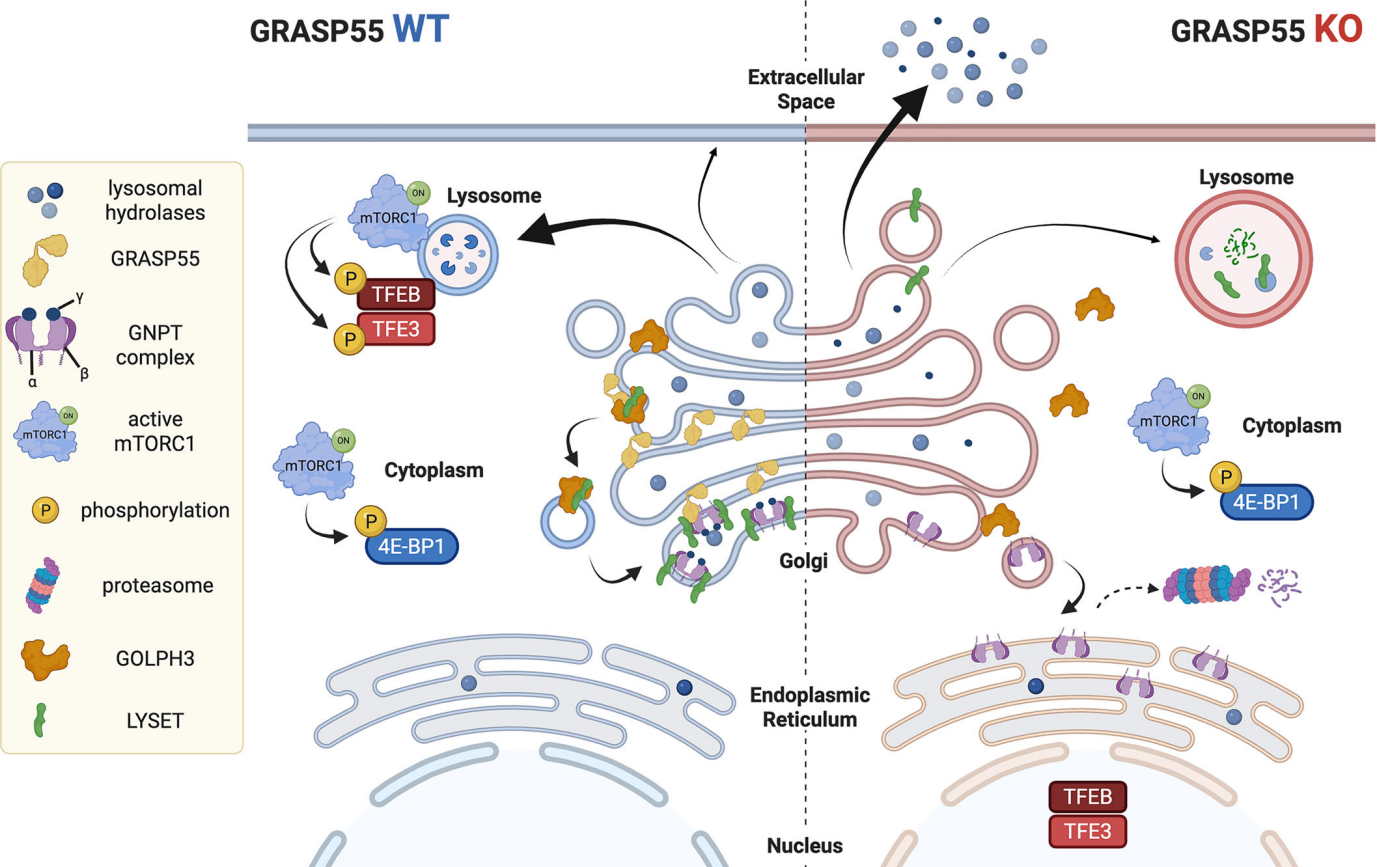
GRASP55 safeguards lysosome function via the GOLPH3-LYSET-GNPTAB axis. Proposed model for the role of GRASP55 in proper sorting of lysosomal enzymes at the Golgi by ensuring correct localization of GOLPH3, LYSET, and GNPTAB; and preventing degradation of LYSET and GNPTAB in lysosomes and at the ER, respectively. As a result, lysosomal enzymes are aberrantly secreted in GRASP55 KO cells, which exhibit dysfunctional and bloated lysosomes, resembling LSD-like phenotypes. The lysosomal dysfunction in GRASP55 KO cells specifically perturbs lysosomal mTORC1 signaling, causing nuclear translocation and activation of the TFEB/TFE3 transcription factors, without affecting cytoplasmic mTORC1 activity.

In sum, our findings draw a model wherein GRASP55 is necessary for proper retention and processing of GNPTAB at the Golgi, presumably via the recruitment of GOLPH3 to Golgi membranes that, in turn, is required for recycling LYSET from late Golgi membranes to the cis-Golgi (Brauer et al, 2024). Consequently, in GRASP55-deficient cells, GNPTAB is mislocalized to the ER where it is targeted for degradation, in part through proteasome-mediated mechanisms, while LYSET mislocalization leads to its degradation inside lysosomes, as also described previously (Brauer et al, 2024) (Fig. 9).

## Discussion

### GRASP55 as a master regulator of protein sorting at the Golgi

Initial work on the closely-related GRASP55 and GRASP65 proteins proposed their involvement in the stacking of Golgi cisternae, however their precise role in Golgi assembly and morphology has been debated by other studies (Barr et al, 1997; Grond et al, 2020; Jarvela and Linstedt, 2014; Shorter et al, 1999; Zhang and Wang, 2015; Zhang and Seemann, 2021). Whereas GRASP55 and GRASP65 share high amino acid sequence similarity in their N-terminal halves, the respective C-terminal SPR regions are substantially different between the two proteins (Shorter et al, 1999; Zhang and Wang, 2015), suggesting that they are also involved in distinct cellular functions. Indeed, we have recently demonstrated that GRASP55, independently of GRASP65, regulates UPS downstream of mTORC1 and starvation/stress signaling (Demetriades et al, 2021; Nüchel et al, 2018; Nüchel et al, 2021); and that it controls the intra-Golgi distribution and the compartmentalized localization of glycosylation enzymes that are responsible for sphingolipid biosynthesis (Pothukuchi et al, 2021). In this study, by reanalyzing our previously published GRASP55-dependent secretome dataset (Nüchel et al, 2021), we reveal a newfound important role for GRASP55 in proper sorting of lysosomal enzymes at the Golgi, with GRASP55-deficient cells showing a typical LSD-like phenotype, manifested as lysosomal enzyme missorting and secretion, lysosomal bloating, and impaired lysosome function. Of note, our data highlight a specific role for GRASP55 in this process, as GRASP65 loss-of-function cells show normal lysosomal enzyme sorting and lysosomal function. Accordingly, at the mechanistic level, while we detected binding between both GRASP55 and GRASP65 with GOLPH3 in co-IP experiments, only GRASP55 co-immunoprecipitated LYSET and GNPTAB. These data suggest that a distinct GRASP65-GOLPH3 complex of yet unknown function may also exist, however this is likely different from the complex formed by GRASP55, GOLPH3, LYSET, and GNPTAB.

### GRASP55 lies at the center of mTORC1 signaling

We previously found that mTORC1 directly phosphorylates a fraction of GRASP55 at the Golgi to control its subcellular localization and prevent the activation of UPS (Demetriades et al, 2021; Nüchel et al, 2021). Accordingly, in TSC-deficient cells, a well-described model of mTORC1 hyperactivation, GRASP55 remained phosphorylated and did not relocalize away from the Golgi even in starved or stressed cells (Demetriades et al, 2021; Nüchel et al, 2021). Besides its role in UPS, hyperactivation of mTORC1 in cellular models of LSDs (assayed by phosphorylation of its canonical substrate S6K) was suggested to be involved in the pathogenesis of the disease due to suppression of autophagy (Bartolomeo et al, 2017). In addition, mTOR signaling may also play a role in LSDs by controlling lysosome biogenesis, via the phosphorylation of its direct targets, the TFEB/TFE3 transcription factors (Bajaj et al, 2019; Martina et al, 2014; Puertollano et al, 2018; Sardiello et al, 2009; Settembre et al, 2011). In particular, TFEB and TFE3 regulate the expression of lysosomal enzymes, lysosomal membrane proteins, as well as lysosome protein receptors (Bajaj et al, 2019). Interestingly, recent studies showed that TSC-mutant cells have more and enlarged lysosomes due to aberrant activation of TFEB/TFE3 transcription factors (Alesi et al, 2021; Alesi et al, 2024), raising the plausible hypothesis that this TSC-mTORC1-TFEB/TFE3 pathway may be regulating lysosome biogenesis partly also via the hyperphosphorylation of GRASP55. Whether the GRASP55-mediated lysosomal protein sorting is also actively regulated downstream of nutrient and stress signaling will require additional work and remains to be seen in future studies.

A major site of mTORC1 activation by nutrients is the lysosomal surface, where it is recruited by the Rag GTPase heterodimers when nutrients are abundant and lysosomes are functional (Fernandes et al, 2024; Gollwitzer et al, 2022; Sancak et al, 2010). While the lysosomal localization of mTORC1 is absolutely necessary for the phosphorylation of its lysosomal substrates like TFEB and TFE3, we recently showed that, in the presence of exogenous amino acids, non-lysosomal mTORC1 is also active and can potently phosphorylate its cytoplasmic (e.g., S6K, 4E-BP1) or Golgi-localized substrates like GRASP55 (Fernandes et al, 2024). Furthermore, perturbing the sorting and delivery of lysosomal enzymes by GNPTAB knockout or knockdown caused delocalization of mTORC1 from the lysosomal surface and specifically affected the phosphorylation of its lysosomal substrates, indicating that lysosome dysfunction differentially affects substrate specificity downstream of mTORC1 (Fernandes et al, 2024). Notably, similar effects were observed in this study using GRASP55-deficient cells, which exhibited diminished lysosomal mTOR localization and enhanced dephosphorylation and activation of the TFEB/TFE3 transcription factors. This aberrant activation of lysosome biogenesis pathways is a particularly interesting—but poorly understood—aspect of LSDs, as it seemingly triggers a vicious circle in cells with dysfunctional lysosomes which promote the generation of even more dysfunctional lysosomes. This is readily evident in classical cellular models of LSDs (e.g., GNPTAB-mutant cells) or in GRASP55 KO cells, in which more and larger lysosomes occupy a large part of the cell volume. Whether blocking this feed-forward loop (e.g., by reducing the expression or blocking the activation of TFEB/TFE3) is beneficial for cell growth and for the homeostatic response of cells to nutrient starvation, two parameters that strongly rely on proper lysosomal function, is currently not clear and warrants further investigation in follow-up studies.

In sum, distinct Golgi-based factors play a central role in the activation of mTORC1 by nutrients on the lysosomal surface, thereby fine-tuning metabolic signaling and organelle biogenesis in cells. In turn, mTORC1 activity may regulate Golgi function and protein secretion, either directly via the phosphorylation of key targets like GRASP55, or indirectly via controlling lysosome/autophagosome biogenesis and trafficking. Such feedback loops are commonly found in the mTORC1 signaling network. Characteristic examples are the regulation of protein synthesis via the mTORC1-dependent phosphorylation of core components of the translation initiation machinery, with eIF4a/EIF4A functioning also as a negative upstream regulator of mTORC1 in *Drosophila* and mammalian cells (Tsokanos et al, 2016); or the direct inhibition of mTORC1 by malonyl-CoA, a metabolic intermediate in de novo lipid biosynthesis that is produced by ACC1 (Acetyl-CoA carboxylase) and is consumed by FASN (fatty acid synthase), two enzymes whose expression levels are, in turn, regulated downstream of mTORC1 and the SREBP transcription factors (Duvel et al, 2010; Nicastro et al, 2023).

### GRASP55 is required for proper trafficking of LYSET and GNPTAB

The heterohexameric GNPT complex localizes at the *cis*-Golgi and is responsible for the M6P-tagging of lysosomal enzymes, enabling their proper sorting at the *trans*-Golgi network (TGN) and their trafficking to the late endosomal compartment. Loss of GNPT expression, activity, or defective processing of the GNPTAB precursor causes ML-II in humans. Here we show that GRASP55 interacts with GOLPH3, LYSET, and GNPTAB and is necessary for their proper localization at the *cis*-Golgi, and for efficient M6P-tagging and sorting of lysosomal enzymes. More specifically, the absence of GRASP55 expression initiates a cascade of interrelated events that start with the loss of GOLPH3 localization at the Golgi. In turn, this leads to abnormal intra-Golgi localization of LYSET that, in the absence of the COPI adaptor GOLPH3 protein, is missorted and delivered to lysosomes for degradation. At the final step, absence of the GRASP55-GOLPH3-LYSET sorting machinery from the Golgi causes the mislocalization of GNPTAB to the ER, a phenomenon that is accompanied by reduced GNPTAB processing, reduced complex stability, and enhanced secretion of the soluble GNPTG subunit. As mentioned above, similar effects on GNPTAB cleavage and stability were previously observed in cells lacking expression of LYSET or GOLPH3/GOLPH3L (Ain et al, 2021; Brauer et al, 2024; Pechincha et al, 2022; Richards et al, 2022; Zhang et al, 2022), further supporting the functional relevance of the interaction between GRASP55 and GOLPH3-LYSET for the regulation of GNPTAB. Future work will be required to determine whether the interaction between GRASP55 and GOLPH3 is direct, to define the molecular interface involved, and to establish the intracellular compartment(s) to which protein GOLPH3 redistributes in GRASP55-deficient cells.

### Highlighting the Golgi–lysosome axis in lysosome biology and in LSDs

Although the role of the Golgi apparatus as the main cargo distribution center in cells is well recognized, the underlying molecular mechanisms of cargo selection remain very poorly understood. With regards to lysosomal enzyme sorting, only a handful of proteins, like GNPTAB, the MPRs, LYSET and GOLPH3/GOLPH3L, were found to participate in the tagging and trafficking of these enzymes, while even fewer have been linked to human LSD-like syndromes when mutated in patients (in addition to mutations in the lysosomal enzymes themselves) (Ain et al, 2021; Tiede et al, 2005; Velho et al, 2019). Here, we reveal a tight connection between GRASP55 and lysosomal enzyme sorting at the Golgi, underscore the importance of this Golgi–lysosome communication for health and disease, and highlight *GRASP55/GORASP2* as a putative susceptibility gene in LSD-like diseases. The central role of GRASP55 in the regulation of multiple key cellular processes at the Golgi and elsewhere, as well as its extensive protein interaction network, suggest that additional, yet unidentified proteins and molecular mechanisms at the Golgi are likely relevant for the maintenance of lysosomal function and the fine-tuning of metabolic signaling in cells.

In sum, a better characterization of the molecular interplay between nutrient-sensing signaling pathways, lysosomal protein sorting at the Golgi, and lysosomal function will be important to understand how these machineries coordinate to control multiple physiological processes in cells, and what goes wrong in associated diseases in humans.

## Methods

**Table:** Reagents and tools table

Reagent/resource	Reference or source	Identifier or catalog number
**Experimental models**		
WI-26 SV40 cells	ATCC	#CCL-95.1
WI-26 SV40 cells GRASP55 KO	Nüchel et al, 2021	N/A
WI-26 SV40 cells GRASP65 KO	This study	N/A
WI-26 SV40 cells GNPTAB KO	This study	N/A
WI-26 SV40 cells GRASP55 KO + GRASP55-Myc	Fernandes et al, 2024	N/A
**Recombinant DNA**		
pITR-TTP	Nüchel et al, 2021	N/A
pCMV-Trp	Nüchel et al, 2021	N/A
pITR-TTP-hGRASP55-Myc	Fernandes et al, 2024	N/A
pITR-TTP-hTMEM115-2xHA	This study	N/A
pITR-TTP-hTMEM115-2xFLAG	This study	N/A
pcDNA4/TO/Myc-His-A	Invitrogen	V103020
pcDNA4/TO/Myc6xHis-hGNPTAB	This study	N/A
pRRL-pUbC-GNPTAB_WT-myc-HygroR	Pechincha et al, 2022 (kind gift from Wilhelm Palm)	N/A
pRRL-pUbC-GNPTAB_Q36L,E39L-myc-HygroR	Pechincha et al, 2022 (kind gift from Wilhelm Palm)	N/A
pSpCas9(BB)-2A-Puro (pX459)	Addgene	#62988
pX459-gRNA-hGNPTAB-ex7	This study	N/A
pX459-gRNA-hGRASP65-ex2	This study	N/A
pIRES-Str-KDEL_ManII-SBP-EGFP	Addgene	#65252
pIRES-Str-KDEL-PSAP-SBP	This study	N/A
**Antibodies**		
Rabbit monoclonal anti-phospho-TFEB (Ser211) (E9S8N)	Cell Signaling Technology	#37681
Rabbit polyclonal anti-TFEB	Cell Signaling Technology	#4240
Rabbit polyclonal anti-TFE3	Cell Signaling Technology	#14779; discontinued
Rabbit polyclonal anti-phospho-4E-BP1 (Thr37/46)	Cell Signaling Technology	#9459
Rabbit polyclonal anti-4E-BP1	Cell Signaling Technology	#9452
Rabbit monoclonal anti-mTOR (7C10)	Cell Signaling Technology	#2983
Mouse monoclonal anti-LAMP2 (H4B4)	Developmental Studies Hybridoma Bank	#H4B4
Rabbit monoclonal anti-Cathepsin B (D1C7Y)	Cell Signaling Technology	#31718
Rabbit polyclonal anti-Cathepsin D	Proteintech	#21327-1-AP
Mouse monoclonal anti-Actin (C2)	BD Bioscience	#612656
Mouse monoclonal anti-GM130	BD Bioscience	#610823
Rabbit polyclonal anti-GRASP55	Proteintech	#10598-1-AP
Mouse monoclonal anti-GRASP55	Proteintech	#66627-1-Ig
Rabbit polyclonal anti-GM130	Proteintech	#11308-1-AP
Rabbit polyclonal anti-PSAP	Proteintech	#10801-1-AP
Rabbit polyclonal anti-HEXB	Proteintech	#16229-1-AP
Rabbit polyclonal anti-PGRN	Proteintech	#18410-1-AP
Rabbit polyclonal anti-Myc	Proteintech	#16286-1-AP
Mouse monoclonal anti-Myc (9E10)	Santa Cruz	#sc-40
Mouse monoclonal anti-Ubiquitin	Santa Cruz	#sc-8017
Mouse monoclonal anti-SBP	Milipore	#MAB10764
Rabbit polyclonal anti-BLZF1/GOLGIN-45	Genetex	#GTX116434
Mouse monoclonal anti-GRASP65	Novus Biologicals	#NBP2-02665
Rabbit polyclonal anti-GNPTG	Novus Biologicals	#H00084572-B01P
Rabbit polyclonal anti-M6P	ABCD Antibodies	#ABCD_AG949
Rabbit polyclonal anti-SQSTM1/p62	Enzo	#BML-PW9860
Rabbit polyclonal anti-LIMP-2	Novus Biologicals	#NB400-129
Rabbit polyclonal anti-LYSET/TMEM251 (IF)	Thermo Fisher	#PA5-61769
Rabbit polyclonal anti-LYSET/TMEM251 (WB)	Atlas Antibodies	#HPA048559
Rabbit polyclonal anti-LC3B	Sigma-Aldrich	#L7543
Rabbit polyclonal anti-TGN46	Novus Biologicals	#NBP1-49643
Rabbit polyclonal anti-FLAG	Proteintech	#20543-1-AP
Mouse monoclonal anti-GIANTIN	Enzo	#ALX-804-600-C100
Mouse monoclonal anti-HSP60	BD Bioscience	#611563
Rat monoclonal anti-HA (3F10)	Roche	#11867423001
Mouse monoclonal anti-HSP47	Enzo	#ADI-SPA-470
Rabbit polyclonal anti-GOLPH3	Proteintech	#19112-1-AP
Peroxidase AffiniPure Donkey Anti-Rabbit IgG (H + L)	Jackson ImmunoResearch	#711-035-152
Peroxidase AffiniPure Donkey Anti-Rat IgG (H + L)	Jackson ImmunoResearch	#712-035-150
Peroxidase AffiniPure Donkey Anti-Mouse IgG (H + L)	Jackson ImmunoResearch	#715-035-150
Alexa Fluor 488 Goat Anti-Rabbit IgG (H + L)	Thermo Fisher	#A-11034
Alexa Fluor 555 Goat-Anti-Rabbit IgG (H + L)	Thermo Fisher	#A-21428
Alexa Fluor 555 Goat Anti-Mouse IgG1	Thermo Fisher	#A-21127
Alexa Fluor 647 Goat Anti-Mouse IgG2b	Thermo Fisher	#A-21242
Alexa Fluor 488 Goat Anti-Mouse IgG (H + L)	Thermo Fisher	#A-11001
Alexa Fluor 555 Goat Anti-Mouse IgG (H + L)	Thermo Fisher	#A-21424
**Oligonucleotides and other sequence-based reagents**		
hGNPTAB-Kozak-s-BamHI	Sigma-Aldrich	5’-tataggatccgccaccATGCTGTTCAAGCTCCTGCAG-3'
hGNPTAB-as-NotI	Sigma-Aldrich	5’-atatgcggccgccgTACTCTGATTCGATTGGGAC-3'
hPSAP-Kozak-s-AscI	Sigma-Aldrich	5’-tataggcgcgccgccaccATGTACGCCCTCTTCCTCCTG-3’
hPSAP-as-XhoI	Sigma-Aldrich	5’-atatctcgagGTTCCACACATGGCGTTTGC-3'
SBP-s-XhoI	Sigma-Aldrich	5’-tatactcgagATGGACGAGAAGACCACTGGTTG-3'
SBP-as-Stop-PacI	Sigma-Aldrich	5’-atatatatttaattaaTGGTTCACGTTGACCTTGTGGGTG-3'
hGORASP1-gRNA-ex2-s	Sigma-Aldrich	5’-caccgTCATCACCATTGGGCACTCG-3'
hGORASP1-gRNA-ex2-as	Sigma-Aldrich	5’-aaacCGAGTGCCCAATGGTGATGAc-3'
hGNPTAB-gRNA-ex7-s	Sigma-Aldrich	5’-caccgCTTGCATTAGCACTAATCCA-3'
hGNPTAB-gRNA-ex7-as	Sigma-Aldrich	5’-aaacTGGATTAGTGCTAATGCAAGc-3'
*LYSET/TMEM251* siGENOME siRNA (Set of 4)	Horizon Discovery	1: 5’-UACAAGAGCUGAUCCCAAA-3’ 2: 5’-GUACCUUACUUACAGAUGU-3’ 3: 5’-GGAUUGGAGUGGGAUUGUA-3’ 4: 5’-ACAACUGAUUGACACGUAA-3’
**Chemicals, enzymes and other reagents**		
X-tremeGENE HP	Roche	#06366236001
Puromycin	Gibco	#A1113803
Cloning cylinders	Sigma-Aldrich	#CLS31668
Complete EDTA-free protease inhibitor	Roche	#11873580001
PhosSTOP	Roche	#4906837001
3 kDa cut-off concentrator tubes	VWR	#516-0227 P
PVDF membranes	Milipore	IPVH00010
Nitrocellulose membrane	Amersham	#10600002
Bovine Serum Albumin	Carl Roth	#8076
anti-HA magnetic beads	Thermo Fisher	#88837
Formaldehyde	Sigma-Aldrich	#252549
Fetal Bovine Serum (FBS)	Bio&Sell	FBS.HP.0500
DAPI	Sigma-Aldrich	#D9542
Fluoromount-G	Invitrogen	#00-4958-02
Magic Red Cathepsin B protease activity Kit	Bio-Rad	#ICT937
Biotin	Sigma-Aldrich	#Β4639
DMEM/F12 GlutaMAX medium	Thermo Fisher	#31331093
Penicillin-Streptomycin solution	Sigma-Aldrich	P4333-100ML
AA-free DMEM/F12	US Biologicals	D9811-01
E64	Carl Roth	#2935.1
Pepstatin A	Carl Roth	#2936.1
MG132	Selleckchem	#S2619
DMSO	Carl Roth	#4720.1
Trizol	Thermo Fisher Scientific	#15596018
Superscript II	Thermo Fisher Scientific	#18064014
Torin1	Cayman Chemicals	#Cay10997-10
Bafilomycin A1	Enzo Life Sciences	#BML-CM110-0100
Protein G agarose	Roche	#11719416001
Protein A agarose	Roche	#11719408001
HiPerfect siRNA transfection reagent	Qiagen	#301705
**Software**		
Fiji	www.imagej.net/software/fiji/downloads	N/A
Graphpad Prism	www.graphpad.com	N/A
Adobe Photoshop	Adobe	N/A
**Other**		
X-ray films	Fujifilm	#4741019289
SP8 Leica confocal microscope	TCS SP8 X	TCS SP8 X
ImageQuant 800	Amersham	N/A

### Cell culture and treatments

All cell lines were grown at 37 °C, 5% CO_2_. Human male diploid lung WI-26 SV40 fibroblasts (WI-26 cells; #CCL-95.1, ATCC; RRID: CVCL_2758) were cultured in DMEM/F12 GlutaMAX medium (#31331093, Thermo Fisher Scientific), containing 10% FBS (FBS.HP.0500, Bio&SELL) and 1% Pen/Strep (P4333-100ML, Sigma-Aldrich). The identity of the WI-26 cells was validated using the Short Tandem Repeat (STR) profiling service, provided by Multiplexion GmbH. No commonly misidentified cell lines were used in this study. All cell lines were regularly tested for *Mycoplasma* contamination, using a PCR-based approach and were confirmed to be *Mycoplasma*-free.

For amino acid (AA) starvation experiments, culture media were replaced with AA-free DMEM/F12 (D9811-01, US Biologicals) containing 10% dialyzed FBS (F0392, Sigma-Aldrich) for 2 h. To inhibit mTOR kinase activity, cells were treated with 250 nM Torin1 (Cay10997-10, Cayman Chemical) for 2 h. For Bafilomycin A1 (#BML-CM110-0100, Enzo Life Sciences) treatment, the drug was added to a final concentration of 100 nM in the media for 8 h before lysis. Treatment with E64 (#2935.1, Roth) and Pepstatin A (#2936.1, Roth) to block lysosomal protease activity was performed by adding a combination of E64 (25 μM) and PepA (50 μM) in the media for 8 h before lysis. For proteasome inhibition, cells were treated with 20 μM MG132 (S2619, Selleckchem) for 8 h before lysis. For all drug treatments, DMSO (#4720.1, Roth) was used as vehicle control. Treatments were performed by replacing the culture media with drug-containing media.

### Antibodies

A list of all primary antibodies used in this study is found in Table EV1. The H4B4 antibody against LAMP2 was obtained from the Developmental Studies Hybridoma Bank, created by the NICHD of the NIH and maintained at The University of Iowa, Department of Biology. H4B4 was deposited to the DSHB by August, J.T. / Hildreth, J.E.K. (DSHB Hybridoma Product H4B4) (Mane et al, 1989).

### Plasmids and molecular cloning

Plasmid expression vectors were generated by cloning PCR-amplified cDNAs, using appropriate primers. All primer sequences are listed in Table EV2. For cDNA generation, total RNA was isolated from human WI-26 fibroblasts using a standard Trizol/chloroform-based extraction (15596018, Thermo Fisher Scientific), and converted to cDNA using Superscript II (#18064014, Thermo Fisher Scientific). For the construction of the GNPTAB expression vector pcDNA4/TO/hGNPTAB-Myc-His, human GNPTAB (NM_024312.5) was amplified from WI-26 cDNA and cloned into the BamHI/NotI restriction sites of the pcDNA4/TO/Myc-His-A plasmid (V103020, Invitrogen). The respective vectors expressing Myc-tagged WT or Q36L/E39L (QELL) GNPTAB were kindly provided by Wilhelm Palm (Pechincha et al, 2022). The RUSH construct for PSAP (pIRES-Str-KDEL-hPSAP-SBP) was generated by amplification of human PSAP (NM_002778.4) from WI-26 cDNA and cloning of the cDNA into the AscI and XhoI restriction sites of the Str-KDEL_ManII-SBP-EGFP vector (Addgene plasmid #65252, described in (Boncompain et al, 2012)). Next, the SBP (Streptavidin-Binding Peptide) tag was amplified from Str-KDEL_ManII-SBP-EGFP and inserted in-frame with PSAP using the XhoI and PacI restriction sites.

The Golgi-Tag expression vectors were generated using GeneArt Strings DNA Fragments (Thermo Fisher) encoding human TMEM115-2xHA or TMEM115-2xFLAG cDNA (NM_007024) flanked by SfiI and NotI restriction sites and cloned into the pITR-TTP vector as described previously (Nüchel et al, 2021).

The pSpCas9(BB)-2A-Puro (PX459) V2.0 plasmid (Addgene plasmid #62988, described in (Ran et al, 2013)) was used for the generation of GRASP65/GORASP1- and GNPTAB-deficient WI-26 cells. In brief, double-stranded DNA oligos that encode guide RNAs (gRNAs) against target genes were cloned into the BbsI restriction sites of the PX459 vector. The oligo sequences used for the sgRNA expression plasmids to generate the GRASP65 and GNPTAB KO lines are provided in Table EV2.

All restriction enzymes were purchased from New England Biolabs. The integrity of all constructs was verified by sequencing.

### Plasmid DNA transfections

Plasmid DNA transfections were performed using the X-tremeGENE HP DNA transfection reagent (#06366236001, Roche) in a 2:1 DNA/transfection reagent ratio when the cells reached ~70% confluency, according to the manufacturer’s instructions. Twenty-four hours post-transfection, cells were either lysed for immunoblotting or fixed for immunofluorescence.

### Gene silencing experiments

Transient knockdown of *LYSET/TMEM251* was performed using siGENOME (pool of 4) gene-specific siRNAs (Horizon Discoveries). An siRNA duplex targeting the *Renilla reniformis* luciferase gene (RLuc) (#P-002070-01-50, Horizon Discoveries) was used as control. Transfections were performed using 20 nM siRNA and the HiPerfect transfection reagent (#301705, Qiagen), according to the manufacturer’s instructions. Cells were collected 72 h post transfection for co-immunoprecipitation experiments, and knockdown efficiency was verified by immunoblotting.

### Generation of knockout cell lines

The GRASP55 KO WI-26 cells were described previously (Nüchel et al, 2021). The GRASP65 and GNPTAB knockout cell lines were generated using the PX459-based CRISPR/Cas9 method, as described elsewhere (Ran et al, 2013). In brief, cells were transfected with sgRNA-expressing vectors and 36 h post-transfection they were selected with puromycin (2 μg/ml) (#A1113803, Gibco) for 5 days. Single cell clones were picked using cloning cylinders (#CLS31668, Sigma-Aldrich) and knockout clones were validated by genomic DNA sequencing and immunoblotting. An empty PX459 vector was used to generate matching control cell lines.

### Generation of stable cell lines

A reconstituted GRASP55 KO WI-26 cell line, stably expressing Myc-tagged human GRASP55 was described previously (Fernandes et al, 2024). The Golgi-IP WI-26 cell lines stably expressing TMEM115-2xHA or 2xFLAG were generated using a sleeping beauty-based transposon system, as described previously (Nüchel et al, 2021).

### Cell lysis, preparation of supernatant samples and immunoblotting

For standard SDS-PAGE and immunoblotting experiments, cells from one well of a six-well plate, at approximately 90% confluence, were lysed in-well with 300 μl ice-cold Triton lysis buffer (50 mM Tris-HCl pH 7.5, 0.5% Triton X-100, 150 mM NaCl, 0.1% SDS), supplemented with 1× EDTA-free cOmplete protease inhibitors (#11873580001, Roche) and 1× PhosSTOP phosphatase inhibitors (#4906837001, Roche). Lysates were clarified by centrifugation (12,000×*g*, 15 min, 4 °C) to remove debris and supernatants transferred to a new tube. Samples were boiled in 1× SDS sample buffer for 5 min at 95 °C (6× SDS sample buffer: 350 mM Tris-HCl pH 6.8, 30% glycerol, 600 mM DTT, 12.8% SDS, 0.12% bromophenol blue).

For protein secretion experiments, serum-free supernatants were centrifuged (2000×*g*, 5 min, 4 °C) to remove dead cells and debris. Cleared culture supernatants were concentrated using 3 kDa cut-off concentrator tubes (#516-0227 P, VWR) according to the manufacturer’s instructions, and 1× SDS sample buffer for 5 min at 95 °C (6× SDS sample buffer: 350 mM Tris-HCl pH 6.8, 30% glycerol, 600 mM DTT, 12.8% SDS, 0.12% bromophenol blue) was added to the concentrated supernatants.

Protein samples were subjected to electrophoretic separation on SDS-PAGE and analysed by standard Western blotting techniques. In brief, proteins were transferred to PVDF membranes (IPVH00010, Millipore), except for M6P and phospho-TFEB^S211^ blots for which nitrocellulose membranes (#10600002, Amersham) were used instead. Membranes were blocked with 5% skim milk powder (#42590, Serva) in TBS-T buffer [50 mM Tris-HCl pH 7.4, 150 mM NaCl, 0.1% Tween-20 (#A1389, AppliChem)] or with 5% BSA (#10735086001, Roche; #8076, Carl Roth) in TBS-T for phosphoprotein immunoblots, and incubated with primary antibodies diluted in TBS-T, for 1 h at room temperature or overnight at 4 °C, followed by incubation with appropriate HRP-conjugated secondary antibodies for 1 h at room temperature. For the phospho-TFEB blots, the primary antibody was incubated in TBS-T also containing 5% BSA. Signals were detected by enhanced chemiluminescence (ECL) and immunoblot images were captured on films (#28906835, GE Healthcare; #4741019289, Fujifilm) or using a digital imager (ImageQuant 800, Amersham). Quantification of immunoblots was performed by densitometric analysis of the band intensities, using the Gel analysis tool of the ImageJ software (Schneider et al, 2012). A list of all primary and secondary antibodies used in this study is provided in Table EV1.

### Golgi purification (Golgi-IP) assays

For Golgi-IP experiments, one 15-cm dish of cells stably expressing Golgi-TAG constructs (TMEM115-2xHA of TMEM115-2xFLAG as a negative control) were grown to 90% confluency and washed twice with ice-cold PBS. Unless otherwise indicated, all subsequent steps were performed at 4 °C. Cells were then scraped in 1 ml PBS supplemented with complete protease inhibitor cocktail (PBS-PI) as described above, centrifuged at 1000×*g* for 1 min, and the cell pellet was resuspended in 1 ml PBS-PI. For input samples, 80 μl of the cells suspension were transferred into a new tube, centrifuged at 1000×*g* for 1 min, and the cell pellet was lysed in 100 μl Triton lysis buffer (50 mM Tris-HCl pH 7.5, 0.5% Triton X-100, 150 mM NaCl, 0.1% SDS) supplemented with 1× EDTA-free cOmplete protease inhibitors (#11873580001, Roche) on ice for 10 min. Lysed input samples were cleared by centrifugation (12,000×*g*, 10 min) and the supernatants were transferred into new tube and boiled in 1× SDS sample buffer for 5 min at 95 °C (6× SDS sample buffer: 350 mM Tris-HCl pH 6.8, 30% glycerol, 600 mM DTT, 12.8% SDS, 0.12% bromophenol blue).

For the Golgi-IP fractions, the remaining cell suspension was homogenized with 20 strokes in pre-chilled hand dounce homogenizers kept on ice. The homogenate was cleared by centrifugation to remove unbroken cells (1000×*g*, 1 min, 4 °C) and the supernatant was incubated with 100 μl pre-washed Pierce anti-HA magnetic beads (#88837, Thermo Fisher Scientific) on a nutating mixer for 5 min at room temperature. Next, the tubes were placed in a magnetic rack on ice, beads were allowed to settle for 1 min, and were washed 4× with 1 ml PBS-PI. After a final wash with 1 ml PBS, Golgi apparatuses were eluted from the beads by addition of 100 μl Triton lysis buffer and incubation for 10 min on ice. The tubes were returned on the magnetic rack, supernatants were transferred into new tubes, and samples were boiled in 1× SDS sample buffer for 5 min at 95 °C.

### Co-immunoprecipitation (co-IP)

For GRASP55-GOLPH3-LYSET-GNPTAB co-IP experiments (Fig. 7D), WI-26 cells (1×10 cm dish per condition) were transfected with a GNPTAB-Myc expression vector and lysed in 500 μl Triton lysis buffer per dish 24 h post-transfection. For GRASP55-GOLPH3 co-IP experiments (Fig. 7E), one full 6-well plate per condition was transfected with *siLYSET/TMEM251* or control siRNAs and lysed in 500 μl Triton lysis buffer per well. Cells were lysed in Triton lysis buffer (50 mM Tris-HCl pH 7.5, 1% Triton X-100, 150 mM NaCl, 1× EDTA-free cOmplete protease inhibitors, 1× PhosSTOP phosphatase inhibitors) using a glass Dounce homogenizer and incubated for 30 min on ice. Samples were clarified by centrifugation (12,000×*g*, 10 min, 4 °C) and incubated with 2 μg of mouse monoclonal anti-GRASP55, 2 μg of mouse monoclonal anti-GRASP65, or 5 μl of mouse monoclonal anti-Myc-tag antibodies for 3–4 h at 4 °C under constant agitation. For *siLYSET* co-IP experiments, samples were incubated with 1 μg of rabbit polyclonal anti-GOLPH3 or 1 μg of rabbit polyclonal anti-GRASP55 antibodies. Complexes were incubated overnight with 40 μl protein G agarose beads (for Fig. 7D) or protein A agarose beads (for Fig. 7E), washed 4× with lysis buffer, boiled in 1× Laemmli sample buffer (5 min, 95 °C), and separated by SDS-PAGE. For all co-IP experiments, input samples (50 μl) were collected before pre-clearing, SDS sample buffer (1× final concentration) was added to the lysates, samples were boiled and analyzed by immunoblotting.

### Immunofluorescence and confocal microscopy

Immunofluorescence/confocal microscopy experiments were performed as described previously (Demetriades et al, 2014; Demetriades et al, 2016; Nüchel et al, 2021) with minor modifications. In brief, cells were grown on glass coverslips and treated as described in the figure legends. Samples were fixed for 10 min at room temperature with 4% formaldehyde (#252549, Sigma-Aldrich) in PBS (for the experiments shown in Figs. 2C,D, 3, 6, EV1, and EV3) or for 5 min at –20 °C with 100% ice-cold methanol (for the experiments shown in Figs. 2A,B, 4, 5, and 7), permeabilized with 0.5% NP-40 (I8896, Sigma-Aldrich) in PBS for 10 min, blocked with 1% FBS (FBS.HP.0500, Bio&SELL) in PBS for 30 min, incubated with primary antibodies diluted in blocking buffer (1% FBS in PBS) for 1 h at room temperature, washed 3x with blocking buffer, and incubated with appropriate highly cross-adsorbed secondary antibodies conjugated to Alexa Fluor 488 or 555 (Thermo Fisher Scientific) for 1 h at room temperature. For immunofluorescence of endogenous LYSET, cells were permeabilized and blocked with 0.2% Saponin, 1% FBS in PBS for 30 min. For these experiments, primary and secondary antibodies were diluted in saponin blocking buffer. Nuclei were stained with 0.1 μg/ml DAPI (#D9542, Sigma-Aldrich). Samples were mounted with Fluoromount-G mounting medium (#00-4958-02, Invitrogen). All images were acquired on an SP8 Leica confocal microscope (TCS SP8 X or TCS SP8 DLS, Leica Microsystems) using 63x or 100x objective lenses. Image acquisition was performed using the LAS X software (Leica Microsystems). Images from single channels are shown in grayscale, whereas in merged images, Alexa Fluor 488 is shown in green and Alexa Fluor 555 in red. In Fig. 7, in merged images, Alexa Fluor 488 is shown in magenta and Alexa Fluor 555 in green. In Fig. 8, in merged images, Alexa Fluor 488 is shown in magenta, Alexa Fluor 555 in green, and Alexa Fluor 647 in red. Brightness and contrast were adjusted for visualization purposes using Fiji (https://imagej.net/software/fiji/downloads) (Schindelin et al, 2012). Alterations were applied to the entire image, keeping the parameters identical between all images of the same channel in each panel.

### Quantification of colocalization

Colocalization analysis in confocal microscopy experiments was performed as in (Demetriades et al, 2016; Fitzian et al, 2021), using the Coloc2 plugin of the Fiji software (Schindelin et al, 2012). At least 50 individual cells from five independent representative images per condition per replicate (3–4 replicates per experiment) were used to calculate Manders’ colocalization coefficient (MCC) with automatic Costes thresholding (Costes et al, 2004; Dunn et al, 2011; Manders et al, 1993). Outlines of individual cells were traced, excluding the area corresponding to the cell nucleus, to generate the region of interest (ROI) used for calculating the MCC to prevent false-positive colocalization due to automatic signal adjustments. MCC is defined as a part of the signal of interest (lysosomal enzymes, mTOR, or GNPTAB-myc), which overlaps with a second signal (LAMP2, GM130, or PDI).

### Enzymatic activity measurements

The enzymatic activities of the lysosomal enzymes arylsulfatase B (ARSB), β-hexosaminidase (HEXB), α-iduronidase (IDUA) and β-glucuronidase (GUSB) in cell lysates and corresponding serum-free supernatants of cultured cells were assayed by estimation of 4-nitrophenol or 4-methylumbelliferone liberated from the enzyme-specific substrate (Di Lorenzo et al, 2018; Kollmann et al, 2012; Pohl et al, 2018).

### Magic Red lysosomal protease activity assay

Intracellular lysosomal protease activity was measured in whole living cells using the Magic Red Cathepsin B protease activity Kit (#ICT937, Bio-Rad) according to manufacturer’s instructions. In brief, cells were labelled with the Magic Red substrate and Hoechst (1 μg/ml) for 1 h at 37 °C, and then fixed with 4% formaldehyde in PBS for 10 min at room temperature and mounted on glass slides with Fluoromount-G mounting medium (#00-4958-02, Invitrogen). Samples were imaged using a Leica SP8 inverted laser-scanning confocal microscope using a ×100 objective lens. Image analysis and quantification was performed using the Fiji software (Schindelin et al, 2012), measuring the relative fluorescence intensity per area of at least 50 individual cells per condition per replicate.

### Retention using selective hooks (RUSH) assay

Synchronized trafficking of proteins in the secretory pathway (Boncompain et al, 2012) was achieved by transfecting WT and GRASP55 KO WI-26 cells with the Str-KDEL-containing PSAP-SBP reporter construct (see also Plasmids). Twenty-four hours post-transfection, cells were treated with 40 μM biotin (#Β4639, Sigma-Aldrich) for different time points as indicated in the figure legends. Next, cells were lysed, supernatants collected and concentrated as described above, and subjected to SDS-PAGE and immunoblotting, or fixed and subjected to indirect immunofluorescence.

### Gene Ontology analysis

Gene Ontology (GO) and pathway enrichment analysis were performed using the Database for Annotation, Visualization and Integrated Discovery (DAVID) tool (Huang da et al, 2009a, 2009b), as described previously (Nüchel et al, 2021). In brief, for the reanalysis of the GRASP55-dependent secretome (PRIDE dataset identifier: PXD020331), originally described in (Nüchel et al, 2021; Data ref: Nüchel et al, 2021), proteins whose intensity increases robustly and significantly (log_2_FC > 1, *P* < 0.05) in the supernatants of GRASP55 KO versus control WI-26 cells were used for GOTERM_CC_FAT, GOTERM_BP_FAT, KEGG_PATHWAY, GOTERM_MF_FAT, and GOTERM_PFAM analyses (Dataset EV1). The human proteome was used as reference list. Data were visualized in a cell plot, generated using DAVID and the associated Flaski apps (https://flaski.age.mpg.de/about/, developed and provided by the MPI-AGE Bioinformatics core facility (Iqbal et al, 2021)) using 9 selected non-redundant significant CC_GO terms for the secretome analysis, or 10 selected non-redundant significant BP_GO terms for the proximome analysis.

The full list of proteins detected in the secretome experiment (Nüchel et al, 2021) was used for generating the Volcano plot (gray dots). Proteins whose secretion changes significantly between GRASP55 KO and control cells (*P* < 0.05) are represented by dark gray dots. Proteins corresponding to the GO term ‘GO:0005764~lysosome’ are shown as red dots.

### Statistical analysis

Statistical analysis and presentation of quantification data was performed using GraphPad Prism (versions 9 and 10). Information on quantifications for each method is also provided in the respective Methods section. Data in all graphs are shown as mean ± SD. For the volcano plot in Fig. 1B, *P* values were calculated using a one-sample *t* test (Value = 0, S0 = 0.1, side = both). For graphs with only two conditions shown (Fig. 6B,D), significance was calculated using Student’s *t* test (unpaired, two-tailed). For all other graphs, significance for the indicated pairwise comparisons was calculated using one-way (Figs. 2–4, and EV2) or two-way (Fig. 5) ANOVA with *post hoc* Tukey’s multiple comparisons test. Sample sizes (*n*) and significance values are indicated in figure legends (****P < 0.0001; *P* values ≥ 0.0001 are shown directly in the figures).

All findings were reproducible over multiple independent experiments, within a reasonable degree of variability between replicates. For most experiments, at least three independent replicates were performed. The sample size for microscopy experiments (number of individual cells used in quantifications) is provided in the respective figure legends. No statistical method was used to predetermine sample size, which was determined in accordance with standard practices in the field. No data were excluded from the analyses. The experiments were not randomized, and the investigators were not blinded to allocation during experiments and outcome assessment.

### Graphics

Models in Figs. 1A and 9, and the summary figure were created with BioRender.com.

## Supplementary information

Table EV1

Table EV2

Peer Review File

Dataset EV1

Source data Fig. 1

Source data Fig. 2

Source data Fig. 2C-H

Source data Fig. 3

Source data Fig. 4

Source data Fig. 5

Source data Fig. 6

Source data Fig. 7

Source data Fig. 8

Figure EV1 Source Data

Figure EV2 Source Data

Figure EV3 Source Data

Expanded View Figures

## Data Availability

This study includes no new data deposited in external repositories. The source data of this paper are collected in the following database record: biostudies:S-SCDT-10_1038-S44319-026-00773-w.
