# Supplementary figures and images for: GRASP55 maintains lysosome function by controlling sorting of lysosomal enzymes at the Golgi (part 1 of 5)
Source: EMBO Rep. 2026 Apr 16;27(11):2947–72. doi: 10.1038/s44319-026-00773-w (PMC13261057; doi:10.1038/s44319-026-00773-w)

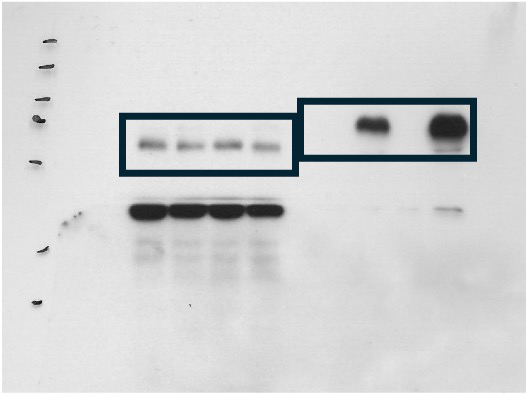

Supplement: Supplementary file 5 — Source data Fig. 1 [file 44319_2026_773_MOESM5_ESM.zip › Figure 1/Figure 1D/Western PSAP.tif]

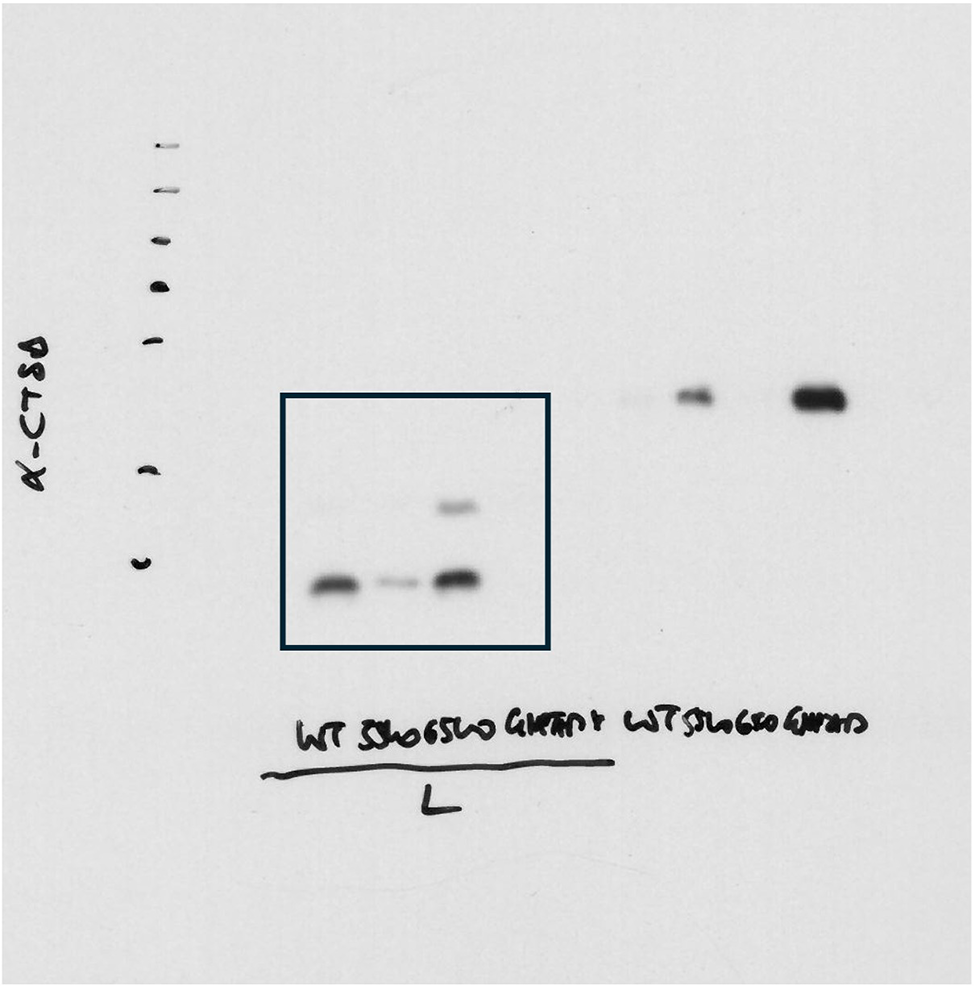

Supplement: Supplementary file 5 — Source data Fig. 1 [file 44319_2026_773_MOESM5_ESM.zip › Figure 1/Figure 1D/Western CTSB Lysate.tif]

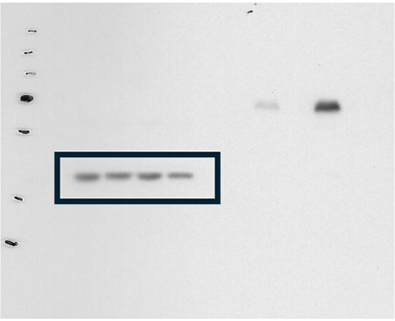

Supplement: Supplementary file 5 — Source data Fig. 1 [file 44319_2026_773_MOESM5_ESM.zip › Figure 1/Figure 1D/Western Actin.tif]

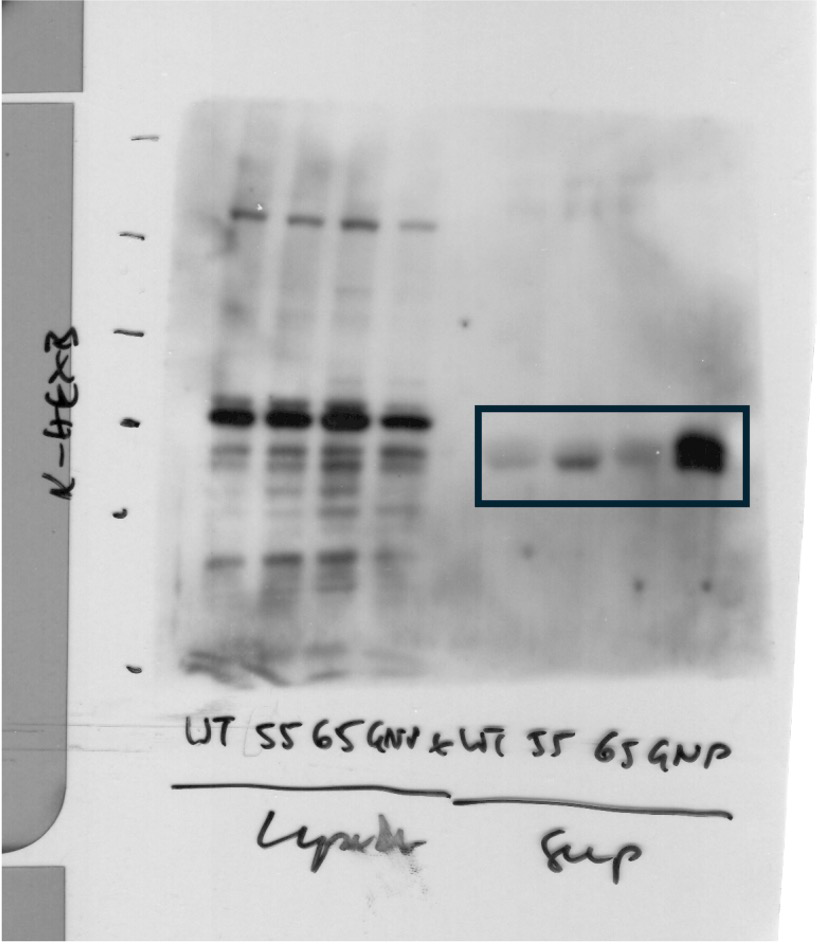

Supplement: Supplementary file 5 — Source data Fig. 1 [file 44319_2026_773_MOESM5_ESM.zip › Figure 1/Figure 1D/Western HEXB culture medium.tif]

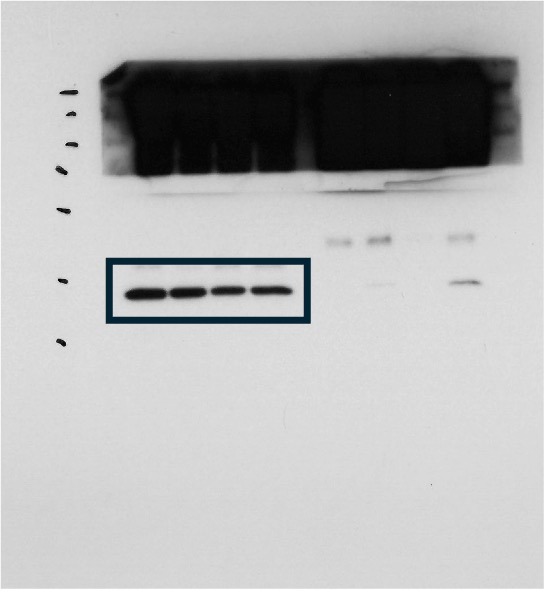

Supplement: Supplementary file 5 — Source data Fig. 1 [file 44319_2026_773_MOESM5_ESM.zip › Figure 1/Figure 1D/Western GNPTG Lysate.tif]

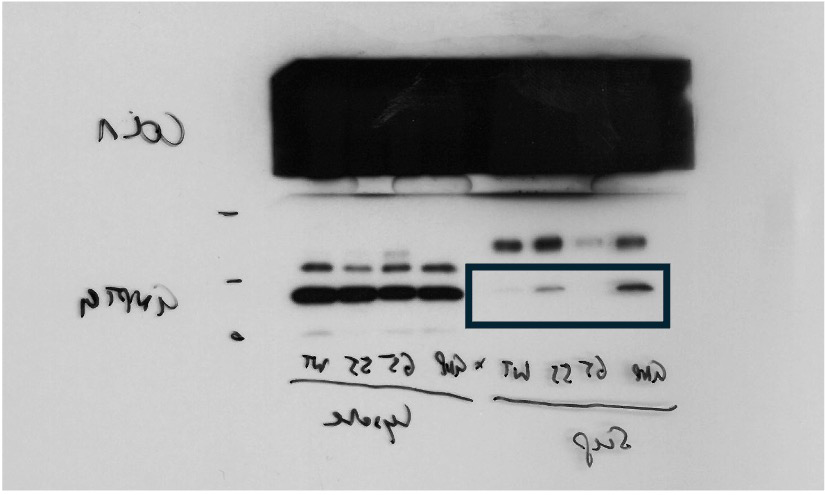

Supplement: Supplementary file 5 — Source data Fig. 1 [file 44319_2026_773_MOESM5_ESM.zip › Figure 1/Figure 1D/Western GNPTG culture medium.tif]

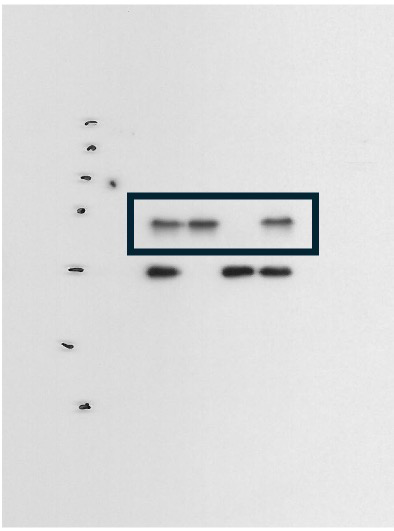

Supplement: Supplementary file 5 — Source data Fig. 1 [file 44319_2026_773_MOESM5_ESM.zip › Figure 1/Figure 1D/Western GRASP65.tif]

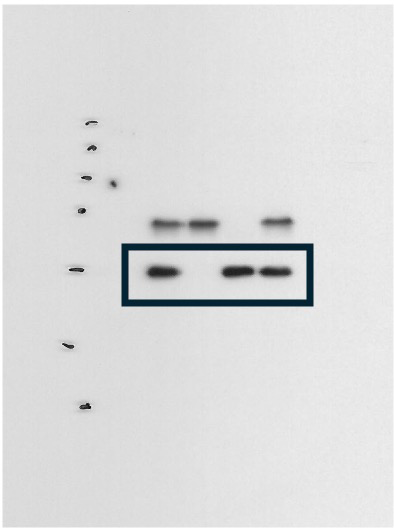

Supplement: Supplementary file 5 — Source data Fig. 1 [file 44319_2026_773_MOESM5_ESM.zip › Figure 1/Figure 1D/Western GRASP55.tif]

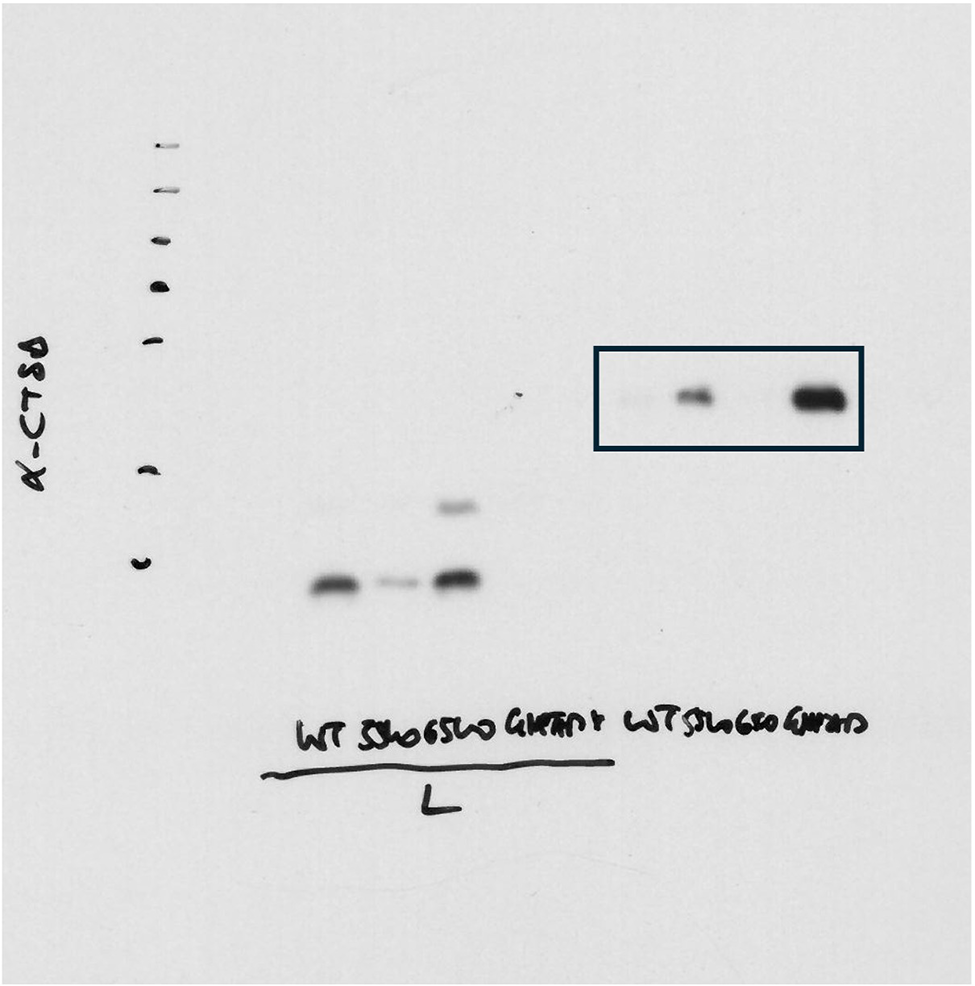

Supplement: Supplementary file 5 — Source data Fig. 1 [file 44319_2026_773_MOESM5_ESM.zip › Figure 1/Figure 1D/Western CTSB culture medium.tif]

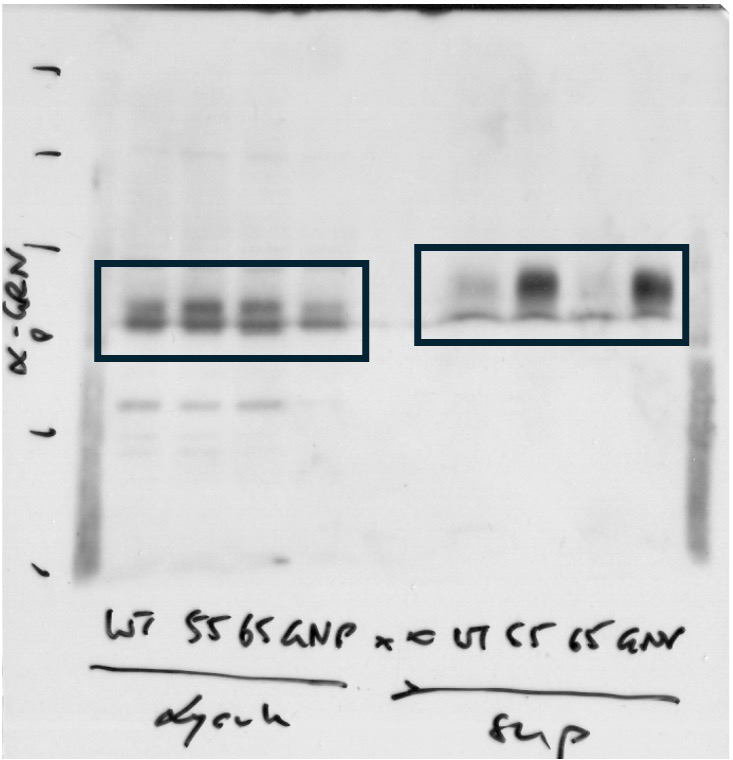

Supplement: Supplementary file 5 — Source data Fig. 1 [file 44319_2026_773_MOESM5_ESM.zip › Figure 1/Figure 1D/Western GRN.tif]

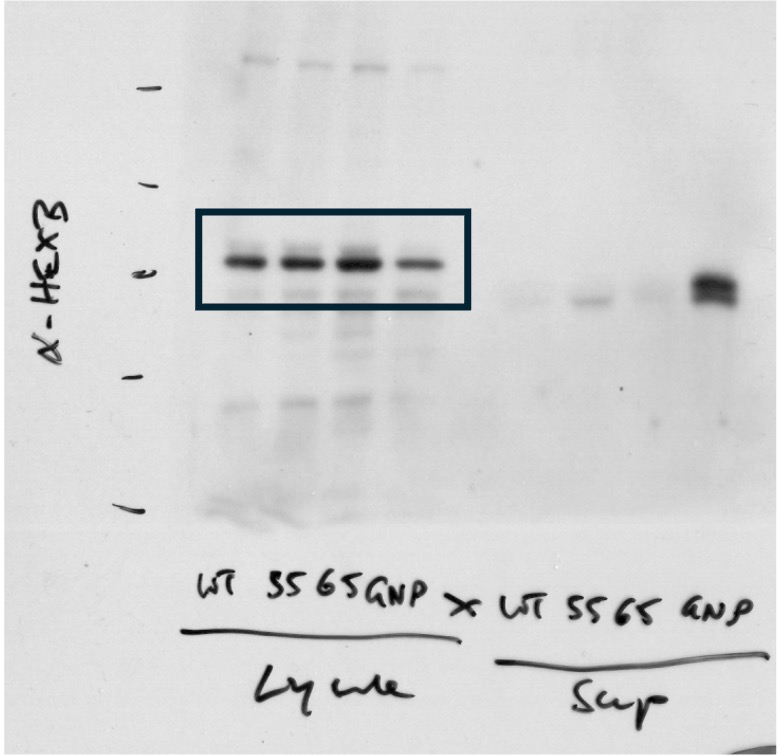

Supplement: Supplementary file 5 — Source data Fig. 1 [file 44319_2026_773_MOESM5_ESM.zip › Figure 1/Figure 1D/Western HEXB Lysate.tif]

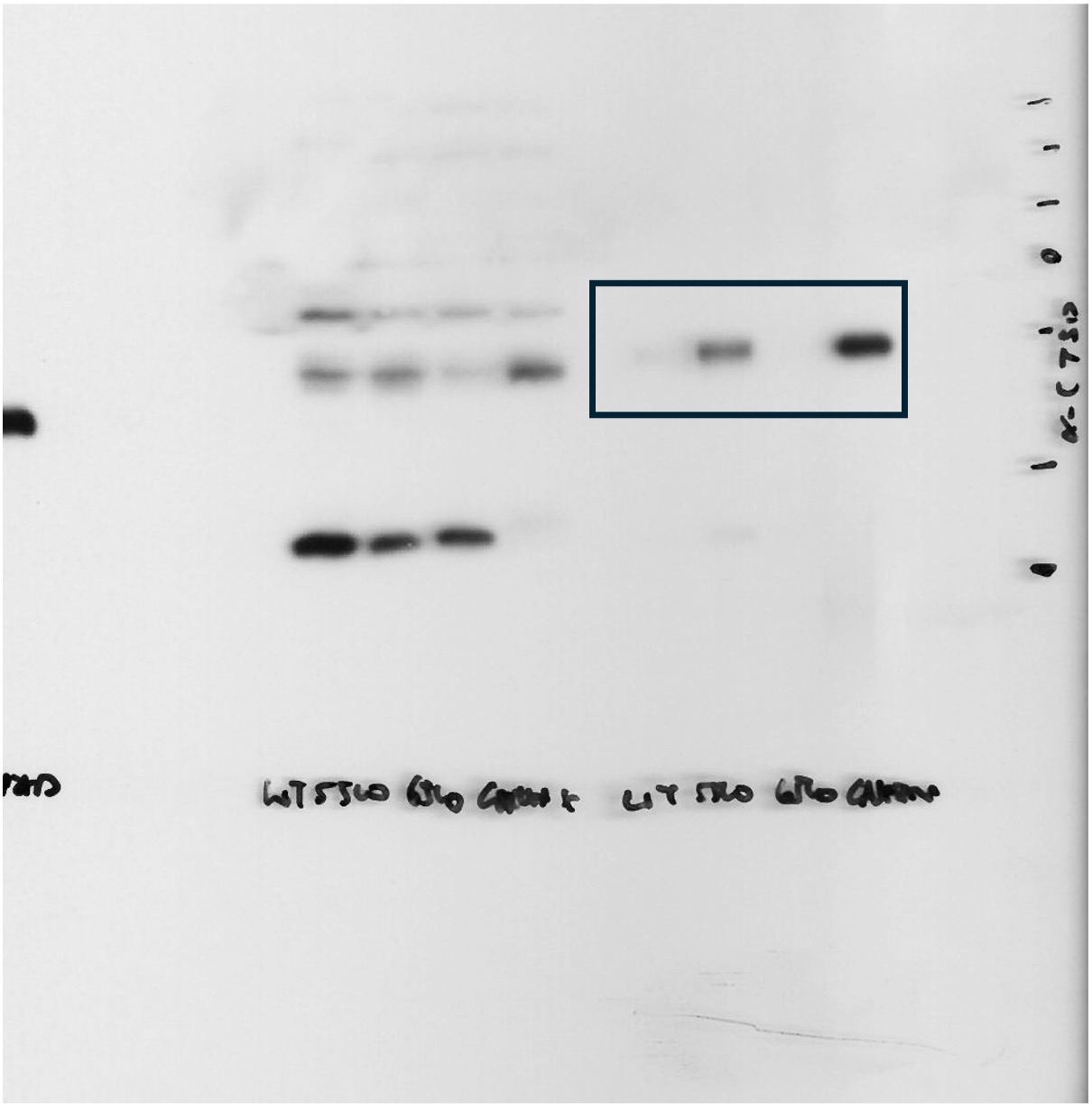

Supplement: Supplementary file 5 — Source data Fig. 1 [file 44319_2026_773_MOESM5_ESM.zip › Figure 1/Figure 1D/Western CTSD culture medium.tif]

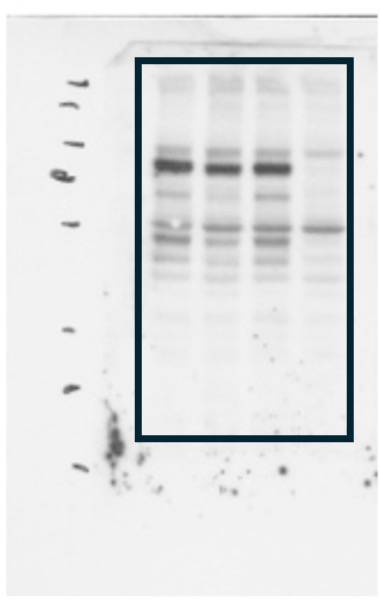

Supplement: Supplementary file 5 — Source data Fig. 1 [file 44319_2026_773_MOESM5_ESM.zip › Figure 1/Figure 1D/Western M6P.tif]

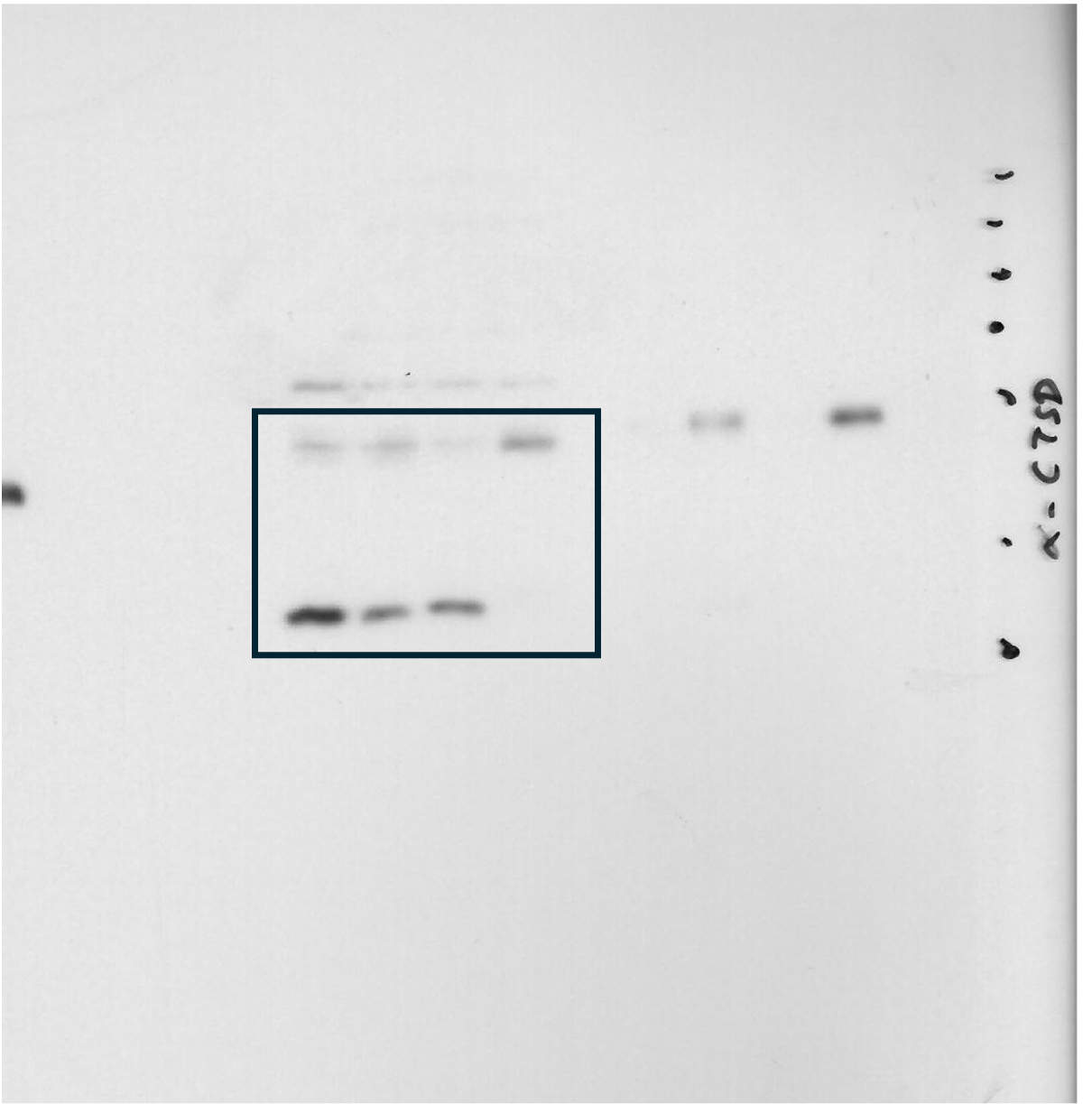

Supplement: Supplementary file 5 — Source data Fig. 1 [file 44319_2026_773_MOESM5_ESM.zip › Figure 1/Figure 1D/Western CTSD Lysate.tif]

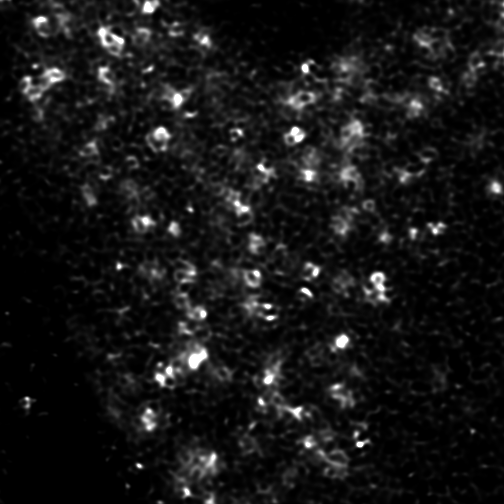

Supplement: Supplementary file 6 — Source data Fig. 2 [file 44319_2026_773_MOESM6_ESM.zip › Figure 2A/IF GRASP65KO LAMP2 inset.tif]

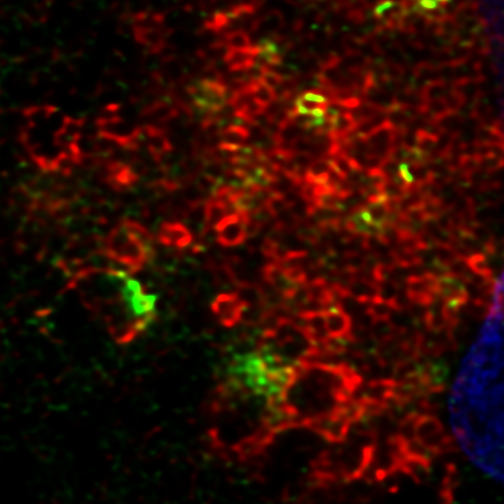

Supplement: Supplementary file 6 — Source data Fig. 2 [file 44319_2026_773_MOESM6_ESM.zip › Figure 2A/IF GRASP55KO CTSB_LAMP2 MERGE inset.tif]

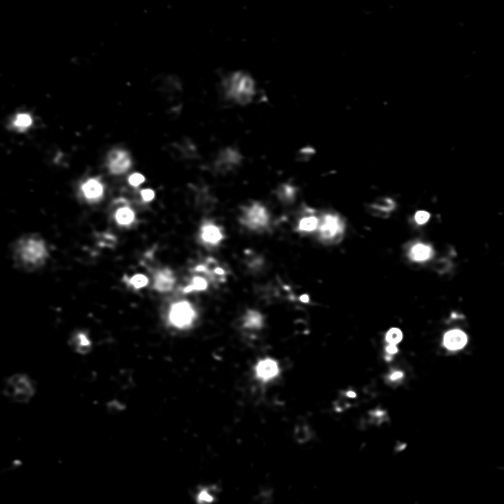

Supplement: Supplementary file 6 — Source data Fig. 2 [file 44319_2026_773_MOESM6_ESM.zip › Figure 2A/IF WT CTSB inset.tif]

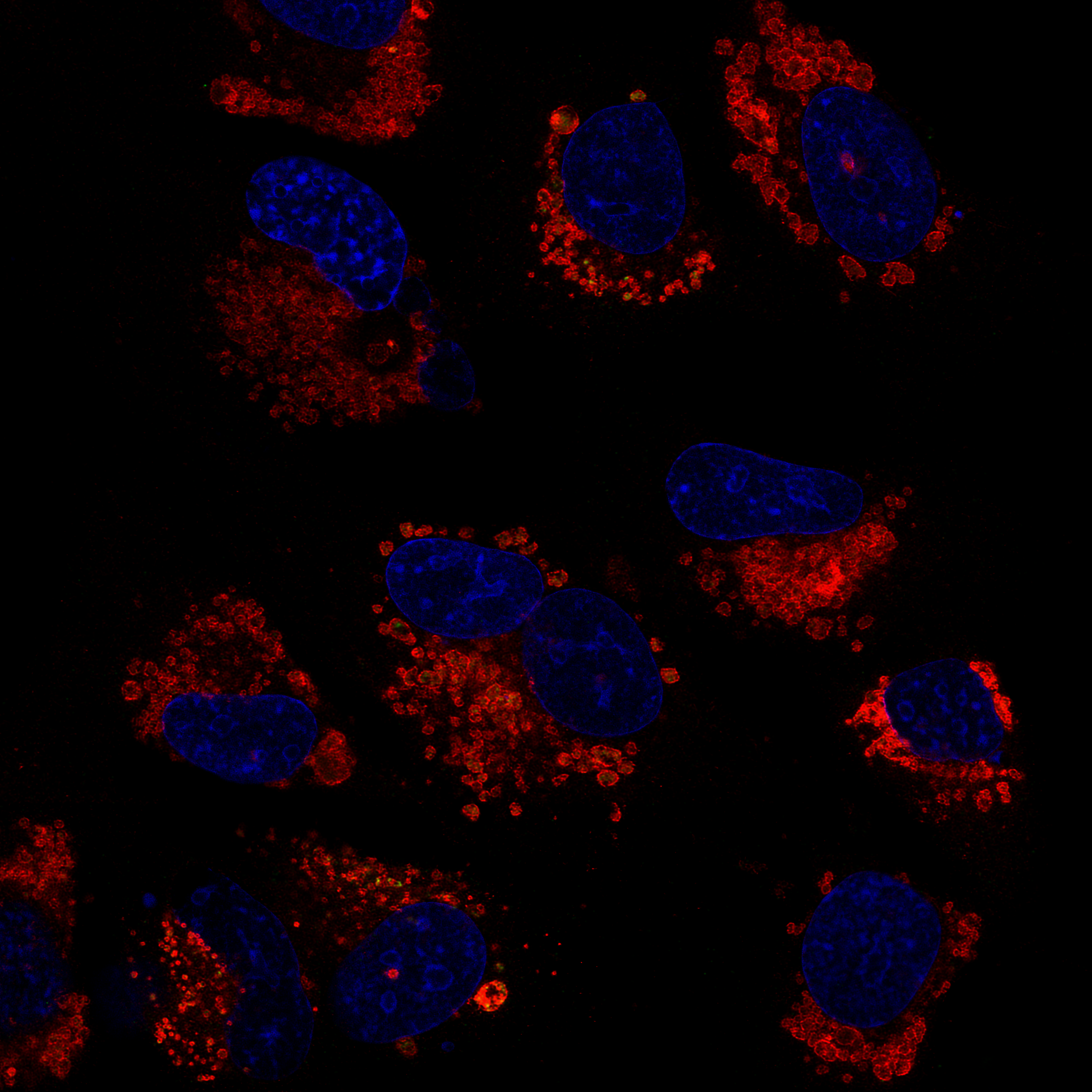

Supplement: Supplementary file 6 — Source data Fig. 2 [file 44319_2026_773_MOESM6_ESM.zip › Figure 2A/IF GNPTABKO CTSB_LAMP2 MERGE.tif]

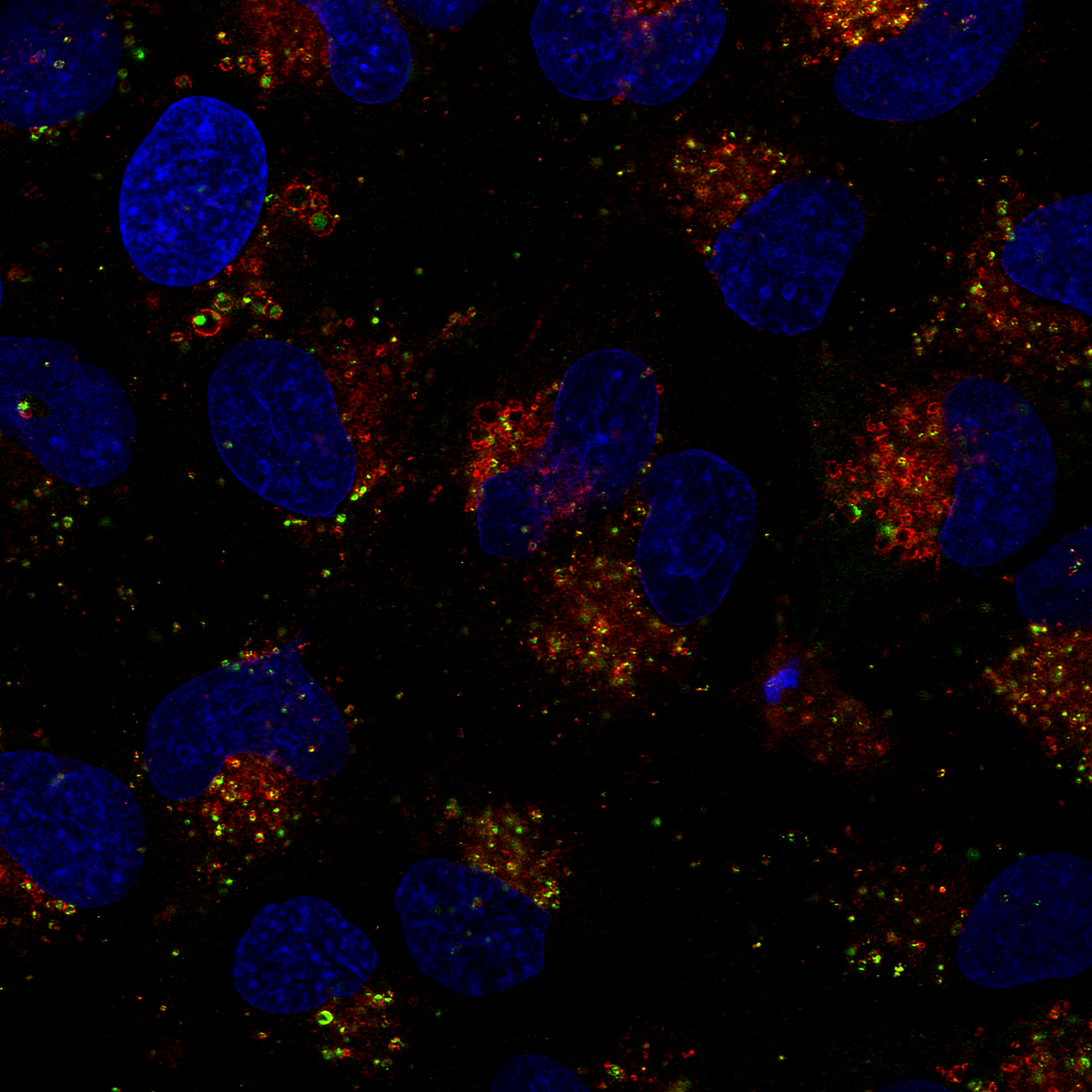

Supplement: Supplementary file 6 — Source data Fig. 2 [file 44319_2026_773_MOESM6_ESM.zip › Figure 2A/IF GRASP55KO CTSB_LAMP2 MERGE.tif]

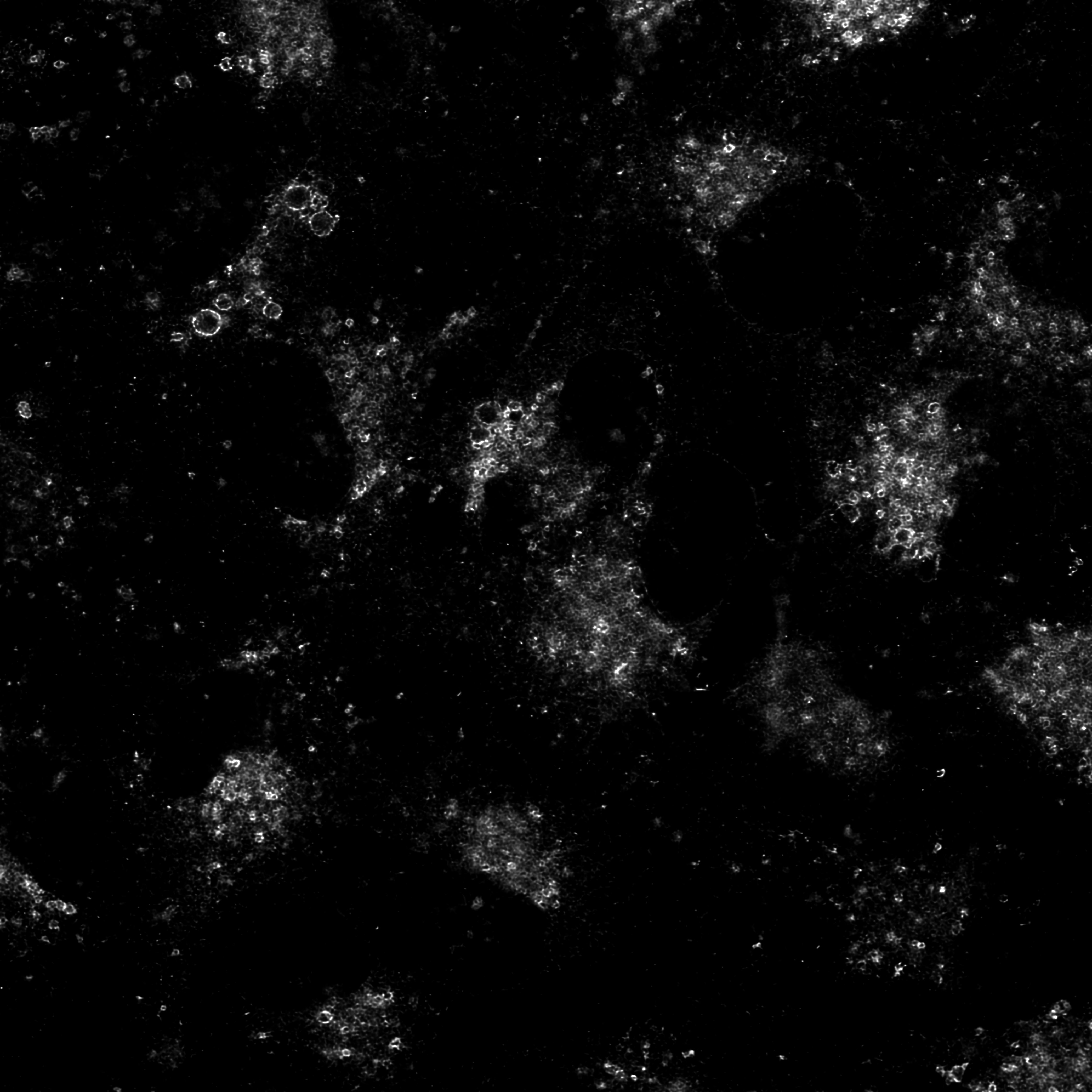

Supplement: Supplementary file 6 — Source data Fig. 2 [file 44319_2026_773_MOESM6_ESM.zip › Figure 2A/IF GRASP55KO LAMP2.tif]

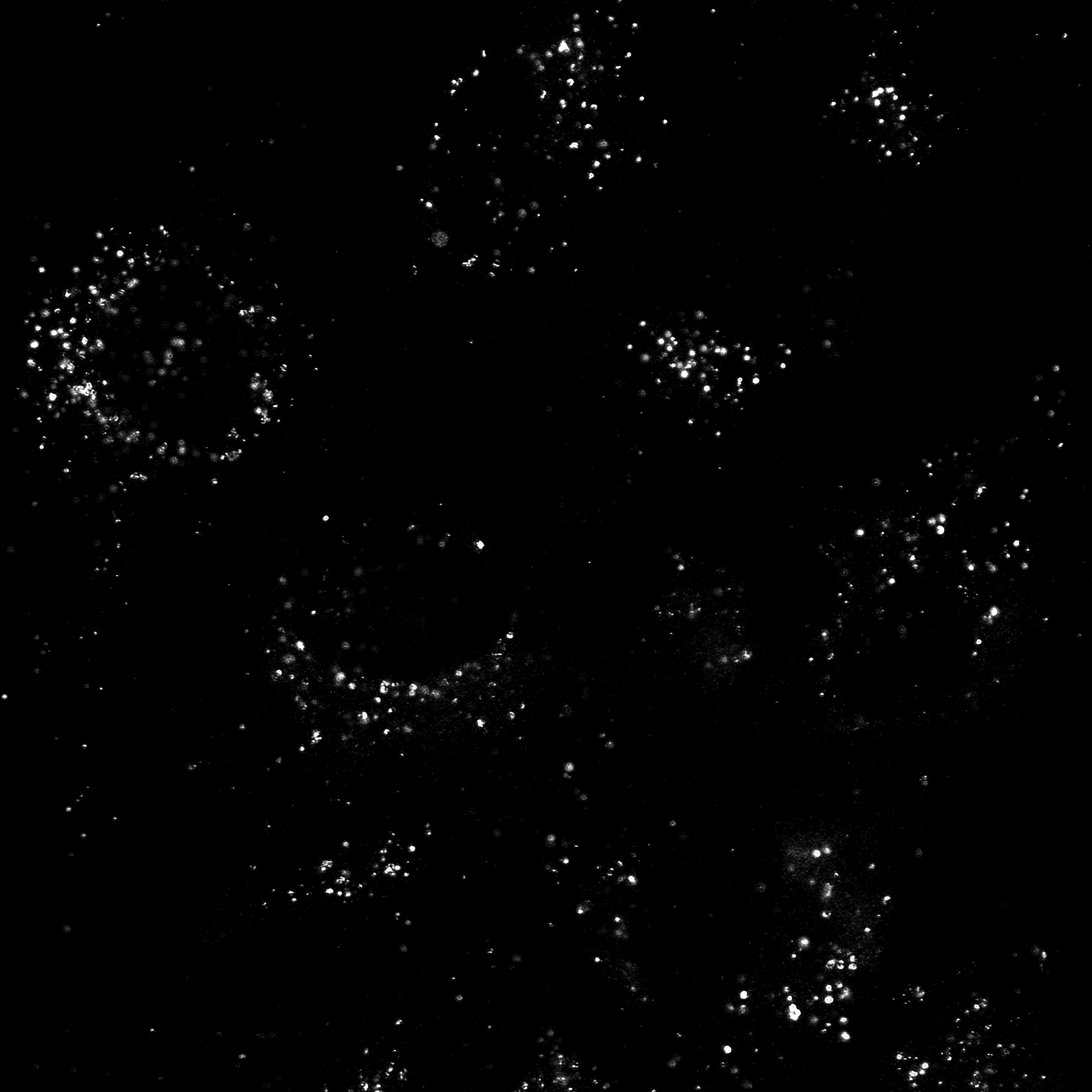

Supplement: Supplementary file 6 — Source data Fig. 2 [file 44319_2026_773_MOESM6_ESM.zip › Figure 2A/IF WT CTSB.tif]

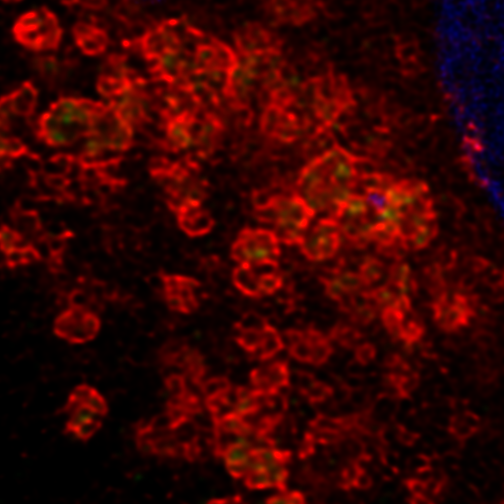

Supplement: Supplementary file 6 — Source data Fig. 2 [file 44319_2026_773_MOESM6_ESM.zip › Figure 2A/IF GNPTABKO CTSB_LAMP2 MERGE Inset.tif]

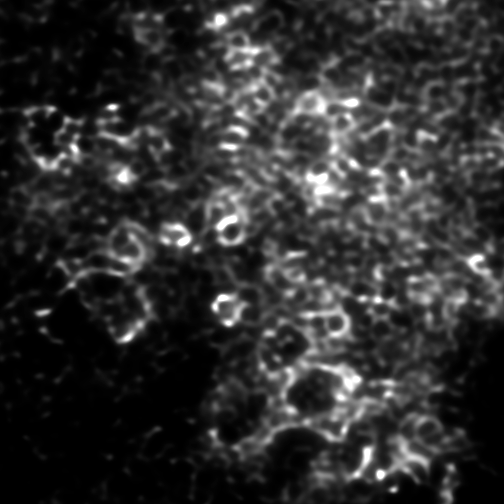

Supplement: Supplementary file 6 — Source data Fig. 2 [file 44319_2026_773_MOESM6_ESM.zip › Figure 2A/IF GRASP55KO LAMP2 inset.tif]

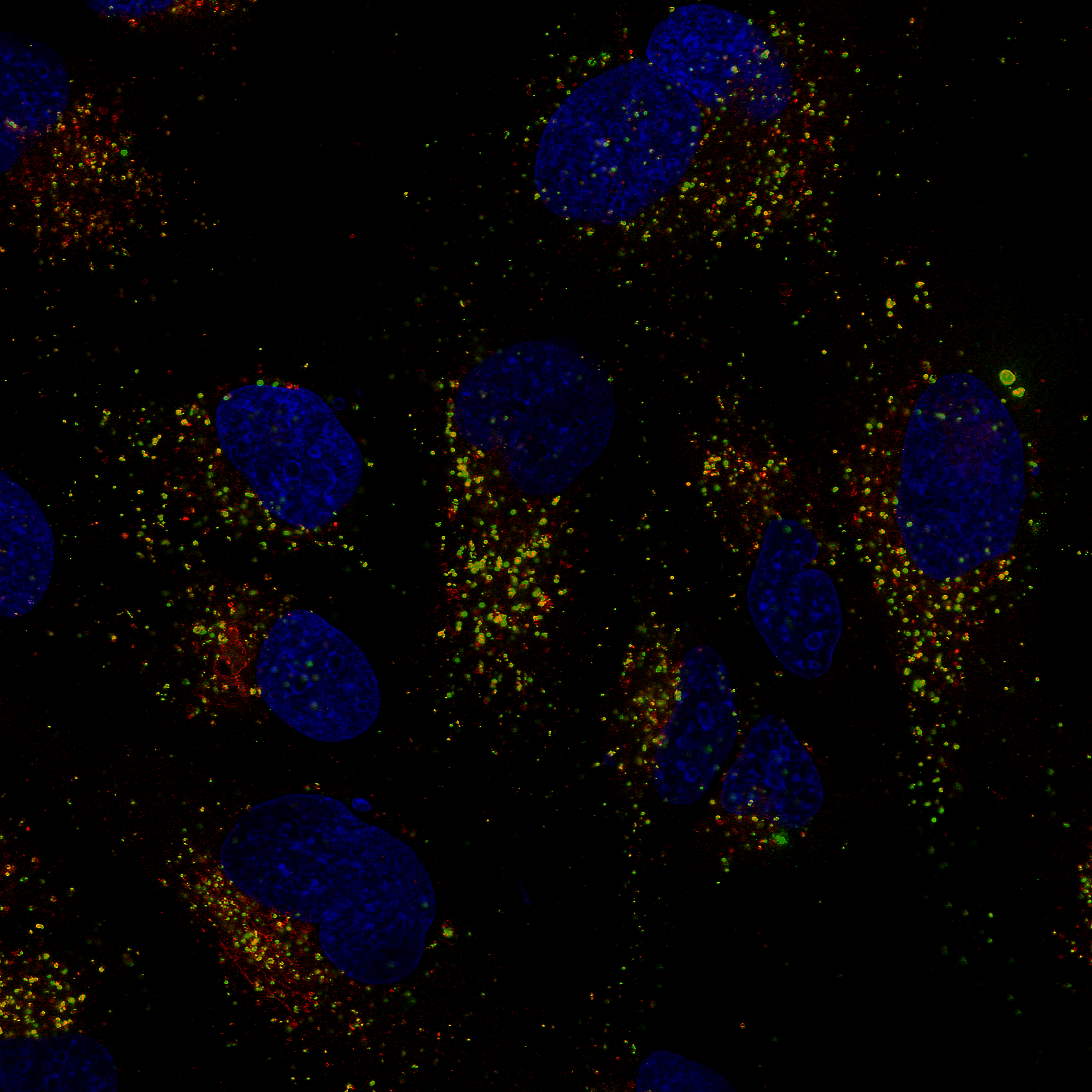

Supplement: Supplementary file 6 — Source data Fig. 2 [file 44319_2026_773_MOESM6_ESM.zip › Figure 2A/IF GRASP65KO CTSB_LAMP2 MERGE.tif]

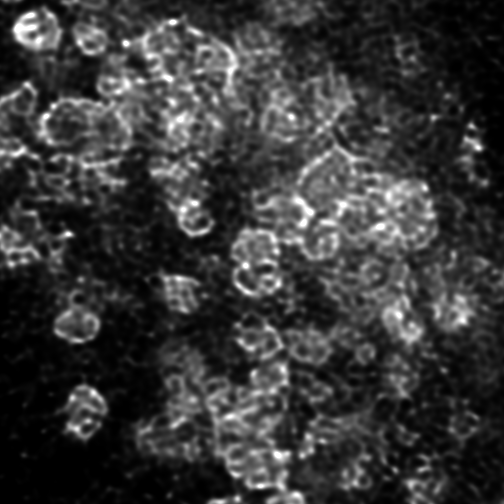

Supplement: Supplementary file 6 — Source data Fig. 2 [file 44319_2026_773_MOESM6_ESM.zip › Figure 2A/IF GNPTABKO LAMP2 inset.tif]

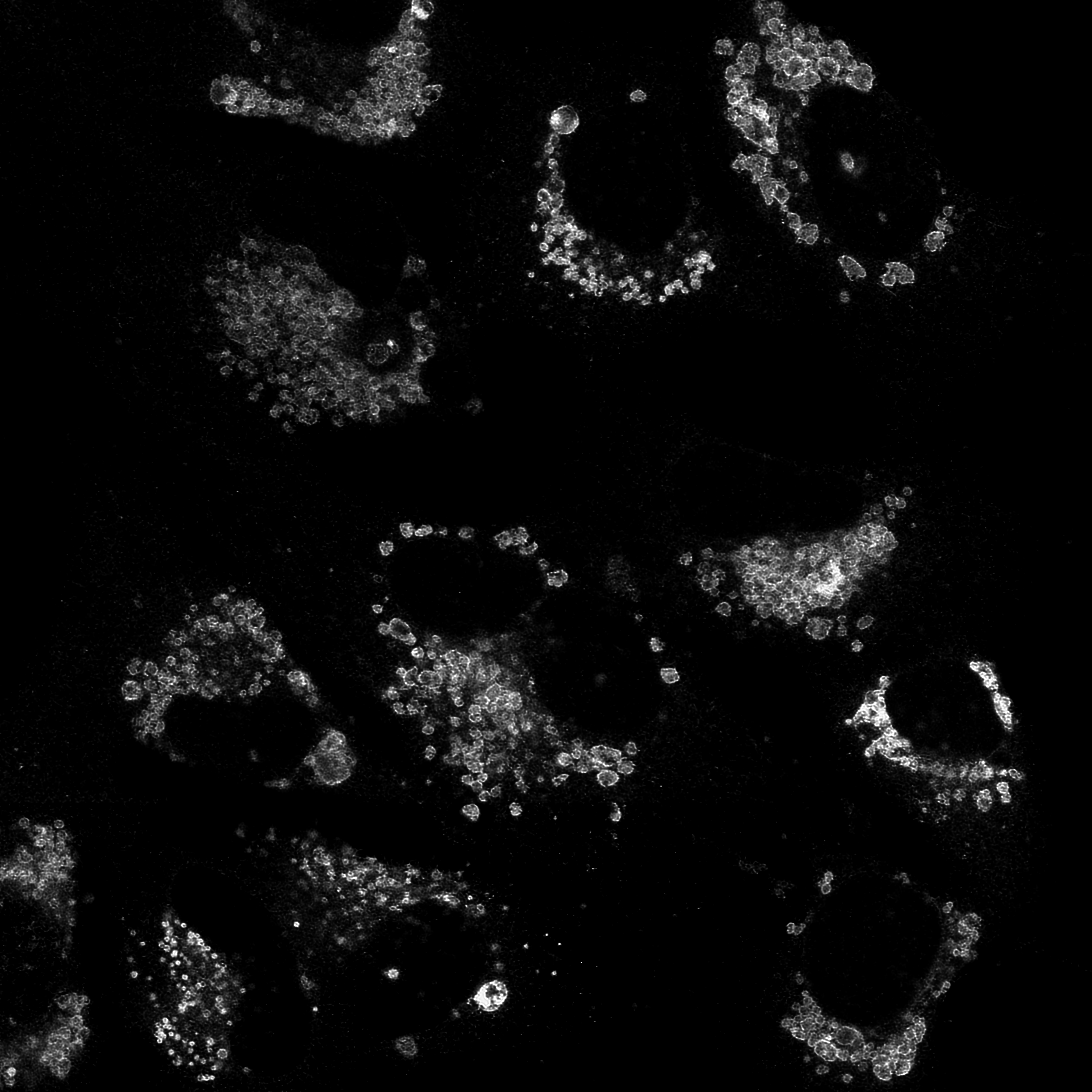

Supplement: Supplementary file 6 — Source data Fig. 2 [file 44319_2026_773_MOESM6_ESM.zip › Figure 2A/IF GNPTABKO LAMP2.tif]

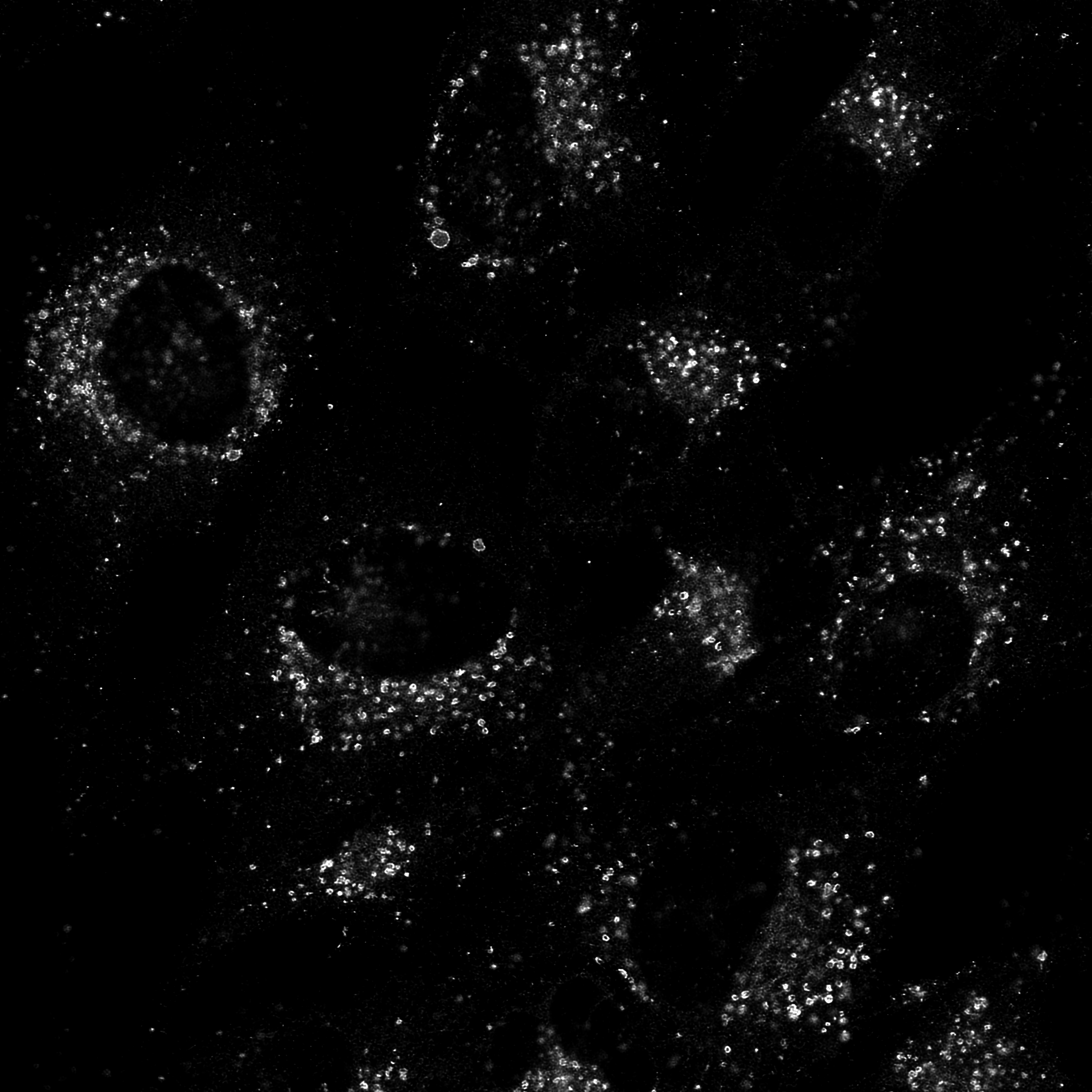

Supplement: Supplementary file 6 — Source data Fig. 2 [file 44319_2026_773_MOESM6_ESM.zip › Figure 2A/IF WT LAMP2.tif]

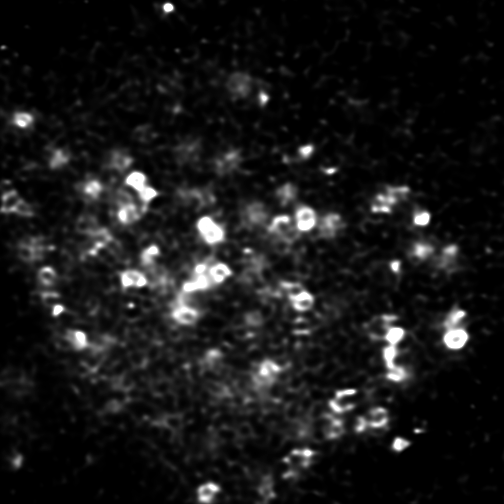

Supplement: Supplementary file 6 — Source data Fig. 2 [file 44319_2026_773_MOESM6_ESM.zip › Figure 2A/IF WT LAMP2 inset.tif]

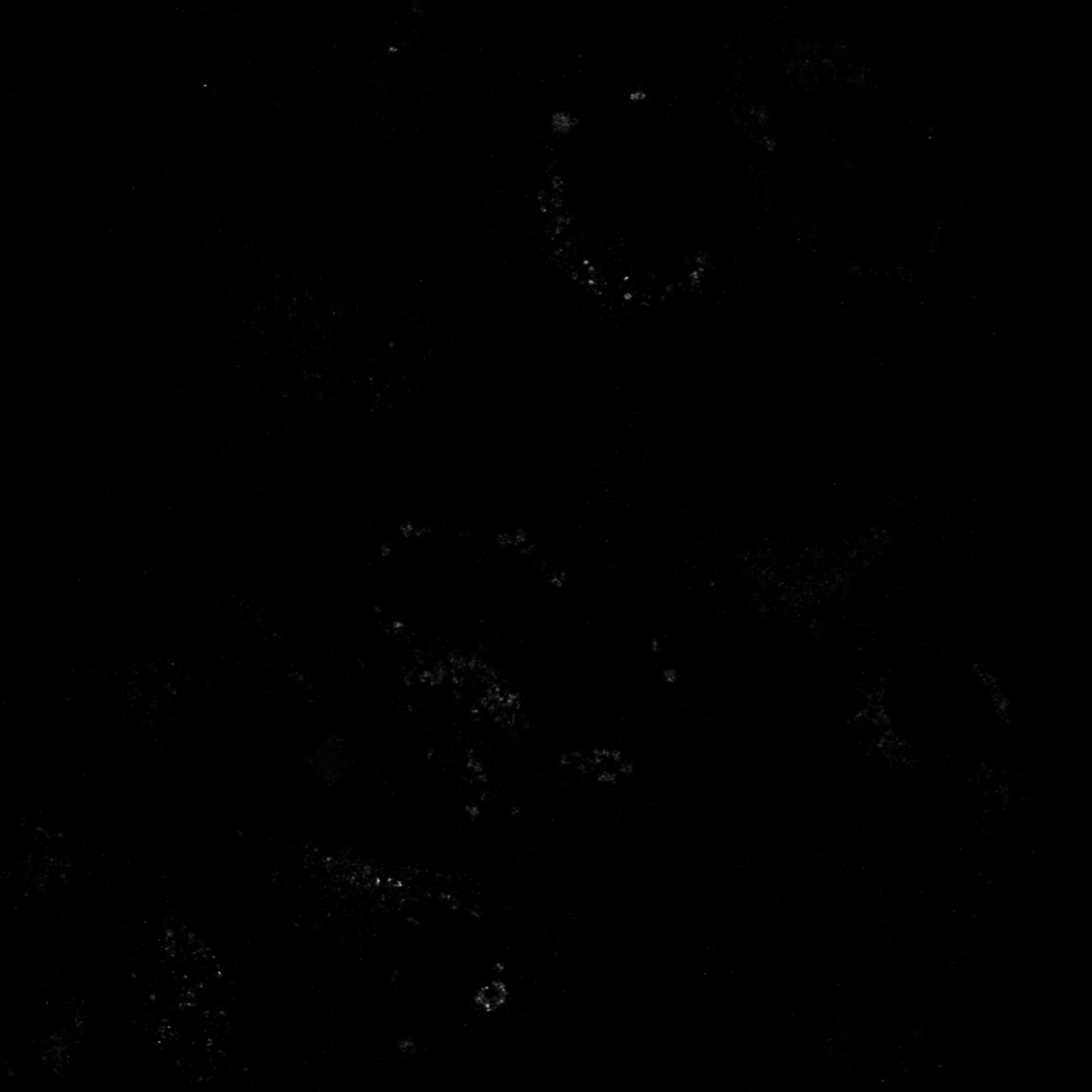

Supplement: Supplementary file 6 — Source data Fig. 2 [file 44319_2026_773_MOESM6_ESM.zip › Figure 2A/IF GNPTABKO CTSB.tif]

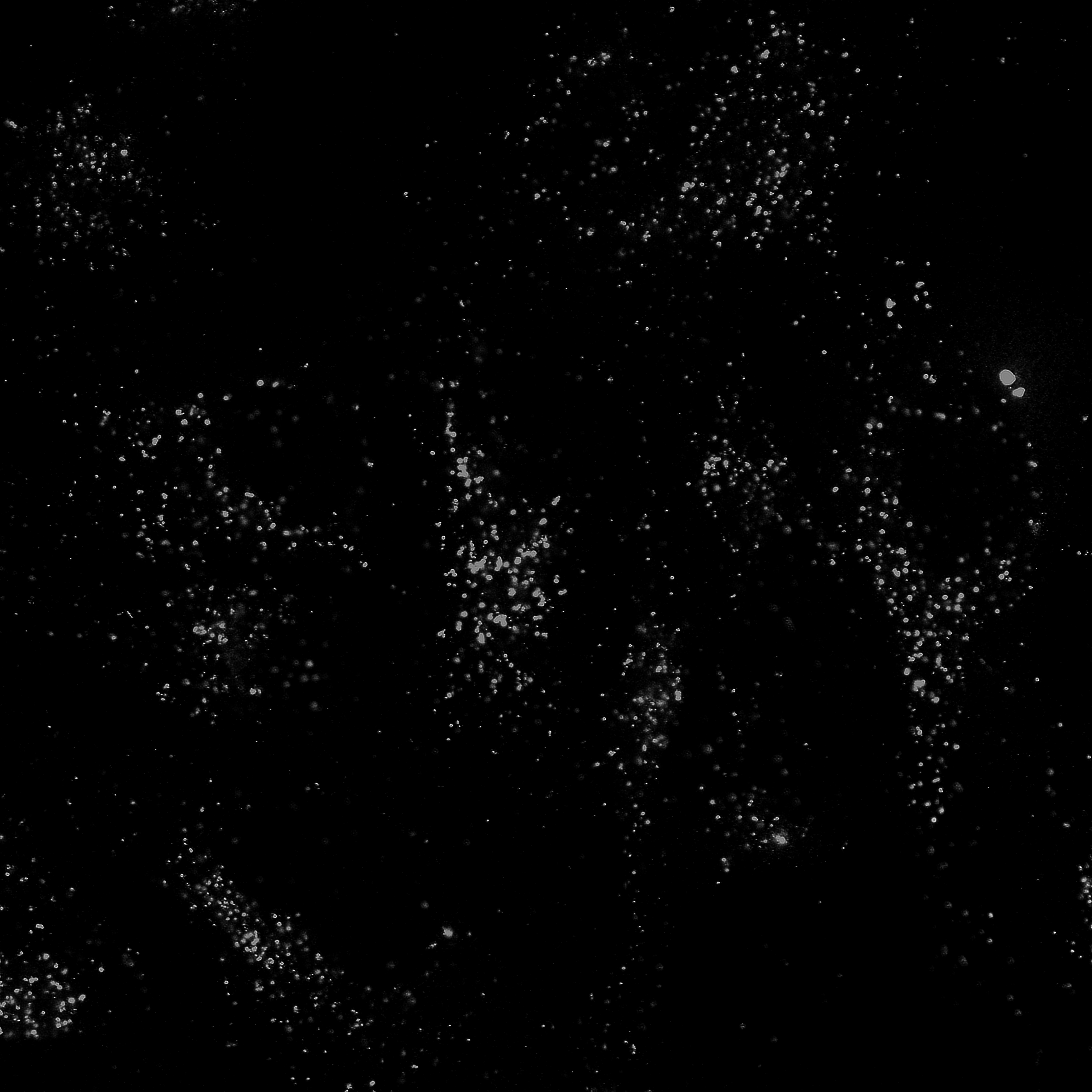

Supplement: Supplementary file 6 — Source data Fig. 2 [file 44319_2026_773_MOESM6_ESM.zip › Figure 2A/IF GRASP65KO CTSB.tif]

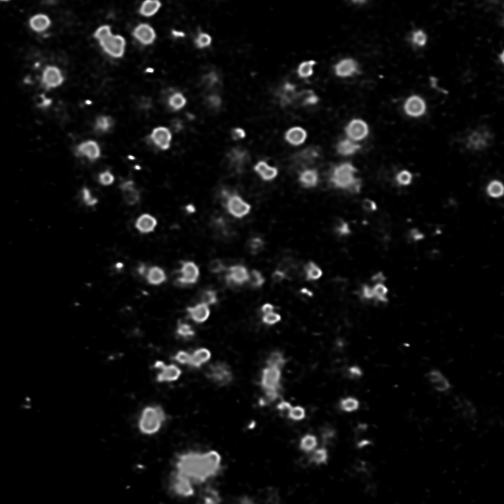

Supplement: Supplementary file 6 — Source data Fig. 2 [file 44319_2026_773_MOESM6_ESM.zip › Figure 2A/IF GRASP65KO CTSB inset.tif]

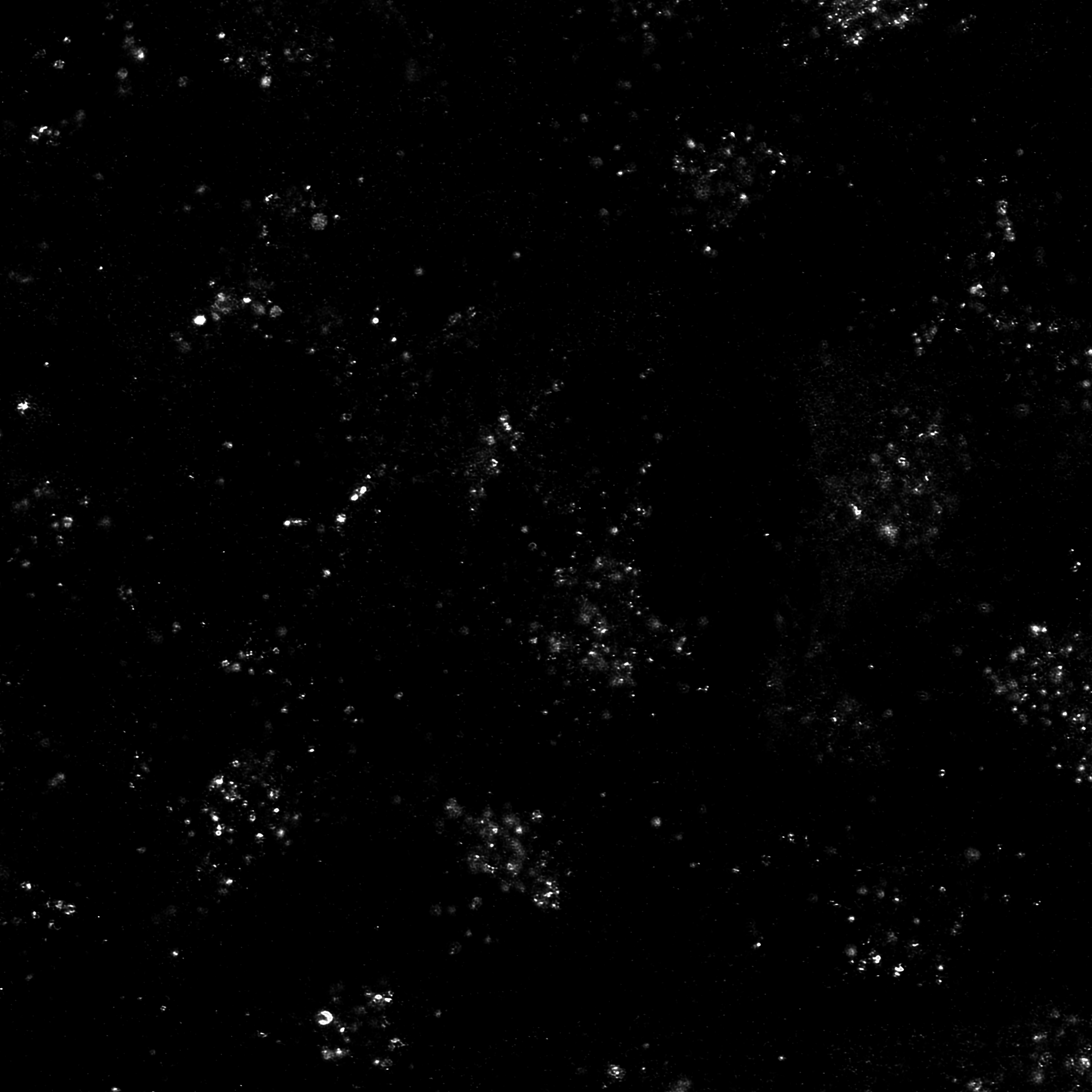

Supplement: Supplementary file 6 — Source data Fig. 2 [file 44319_2026_773_MOESM6_ESM.zip › Figure 2A/IF GRASP55KO CTSB.tif]

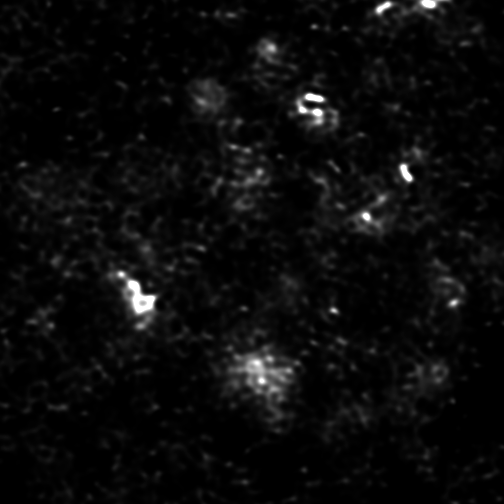

Supplement: Supplementary file 6 — Source data Fig. 2 [file 44319_2026_773_MOESM6_ESM.zip › Figure 2A/IF GRASP55KO CTSB inset.tif]

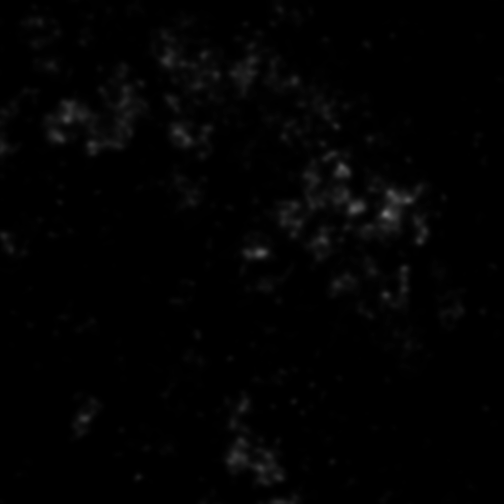

Supplement: Supplementary file 6 — Source data Fig. 2 [file 44319_2026_773_MOESM6_ESM.zip › Figure 2A/IF GNPTABKO CTSB Inset.tif]

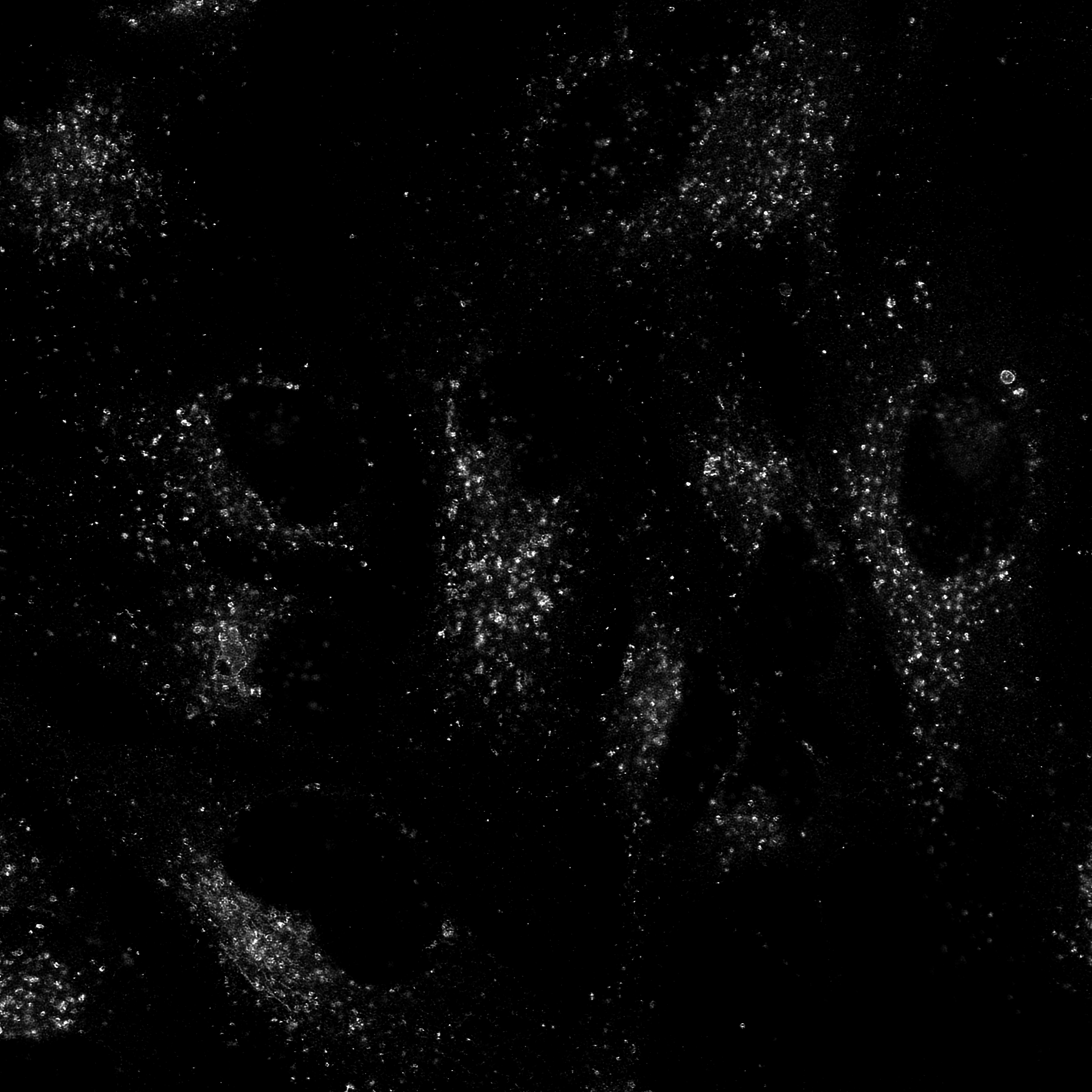

Supplement: Supplementary file 6 — Source data Fig. 2 [file 44319_2026_773_MOESM6_ESM.zip › Figure 2A/IF GRASP65KO LAMP2.tif]

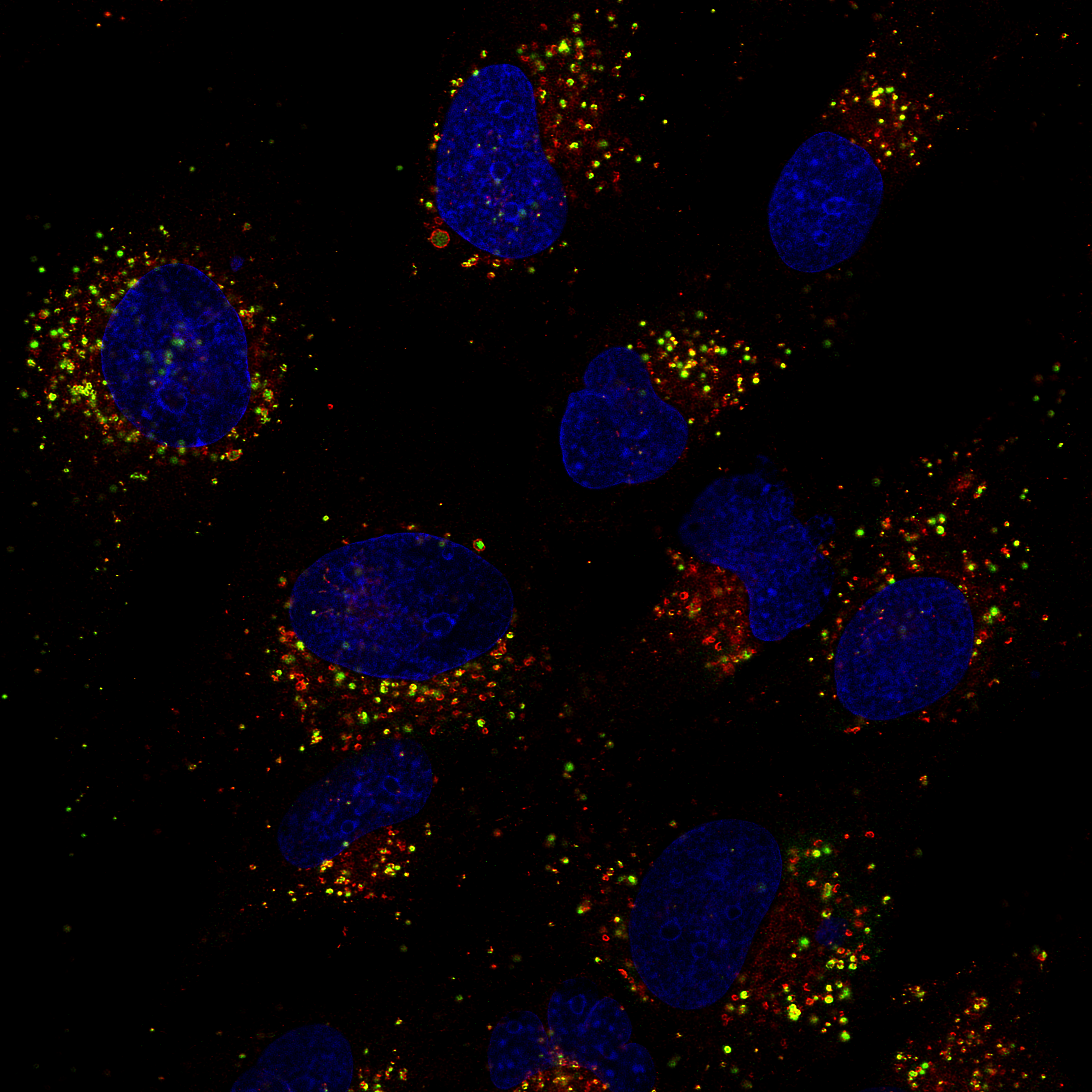

Supplement: Supplementary file 6 — Source data Fig. 2 [file 44319_2026_773_MOESM6_ESM.zip › Figure 2A/IF WT CTSB_LAMP2 MERGE.tif]

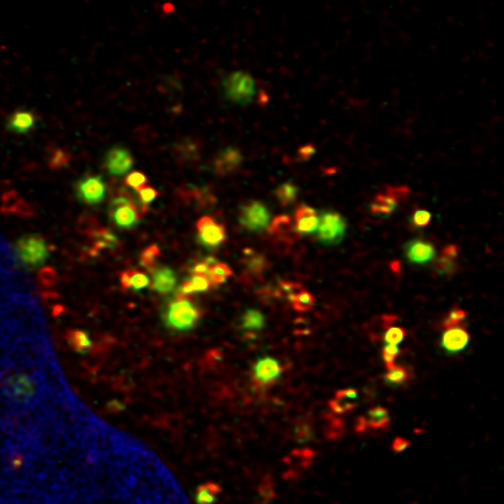

Supplement: Supplementary file 6 — Source data Fig. 2 [file 44319_2026_773_MOESM6_ESM.zip › Figure 2A/IF WT CTSB_LAMP2 MERGE inset.tif]

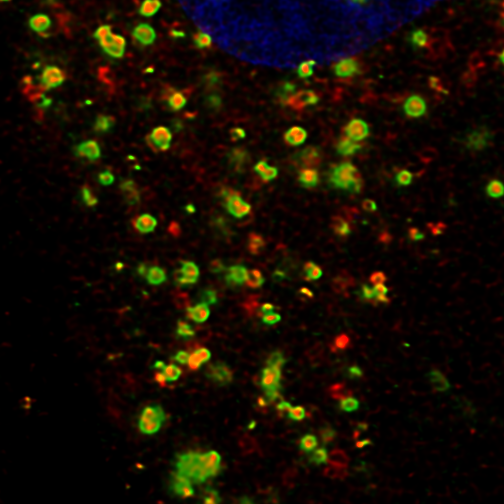

Supplement: Supplementary file 6 — Source data Fig. 2 [file 44319_2026_773_MOESM6_ESM.zip › Figure 2A/IF GRASP65KO CTSB_LAMP2 MERGE inset.tif]

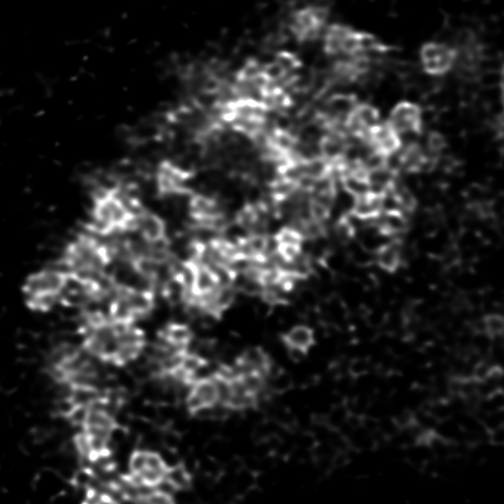

Supplement: Supplementary file 6 — Source data Fig. 2 [file 44319_2026_773_MOESM6_ESM.zip › Figure 2B/IF GNPTABKOLAMP2 Inset.tif]

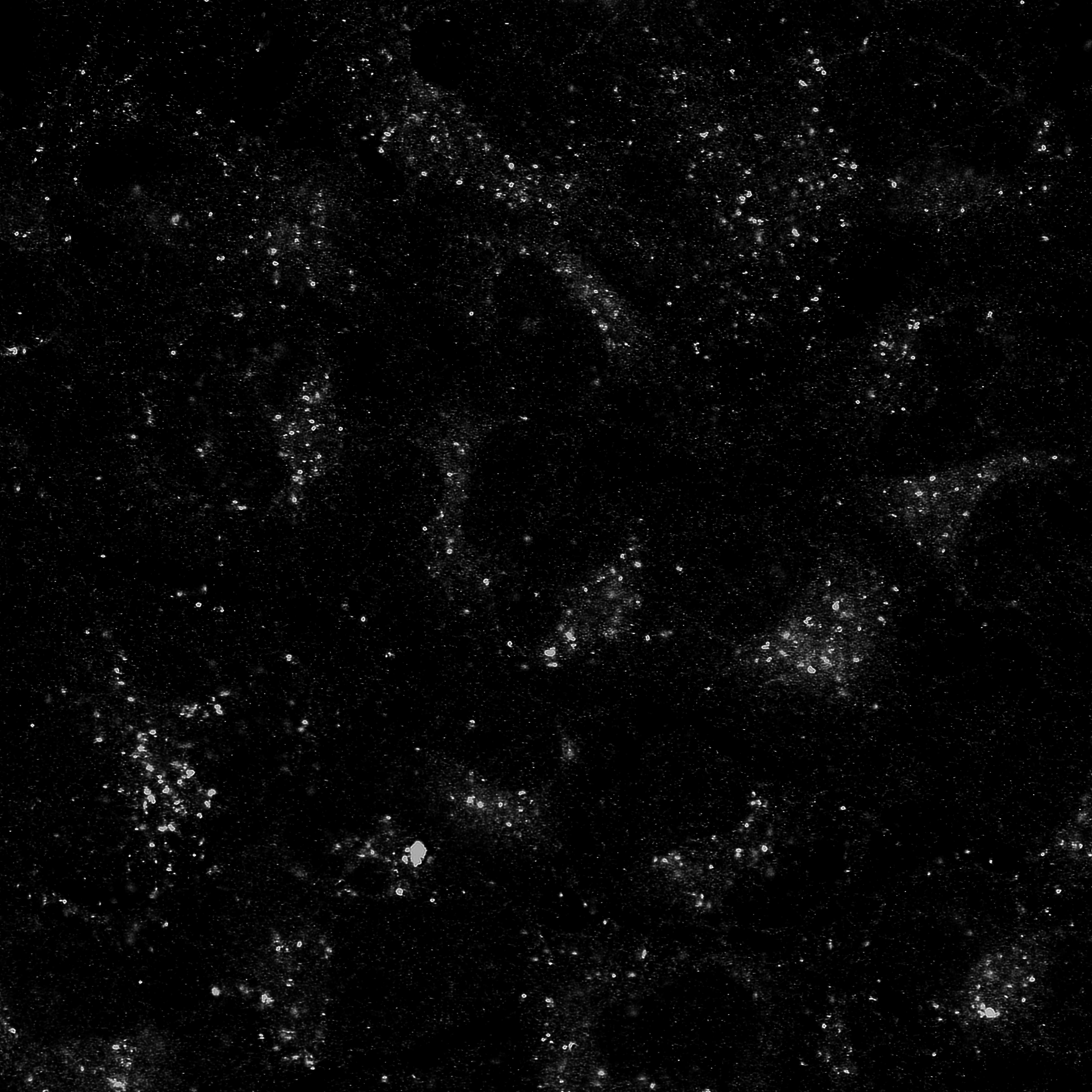

Supplement: Supplementary file 6 — Source data Fig. 2 [file 44319_2026_773_MOESM6_ESM.zip › Figure 2B/IF WT CTSD.tif]

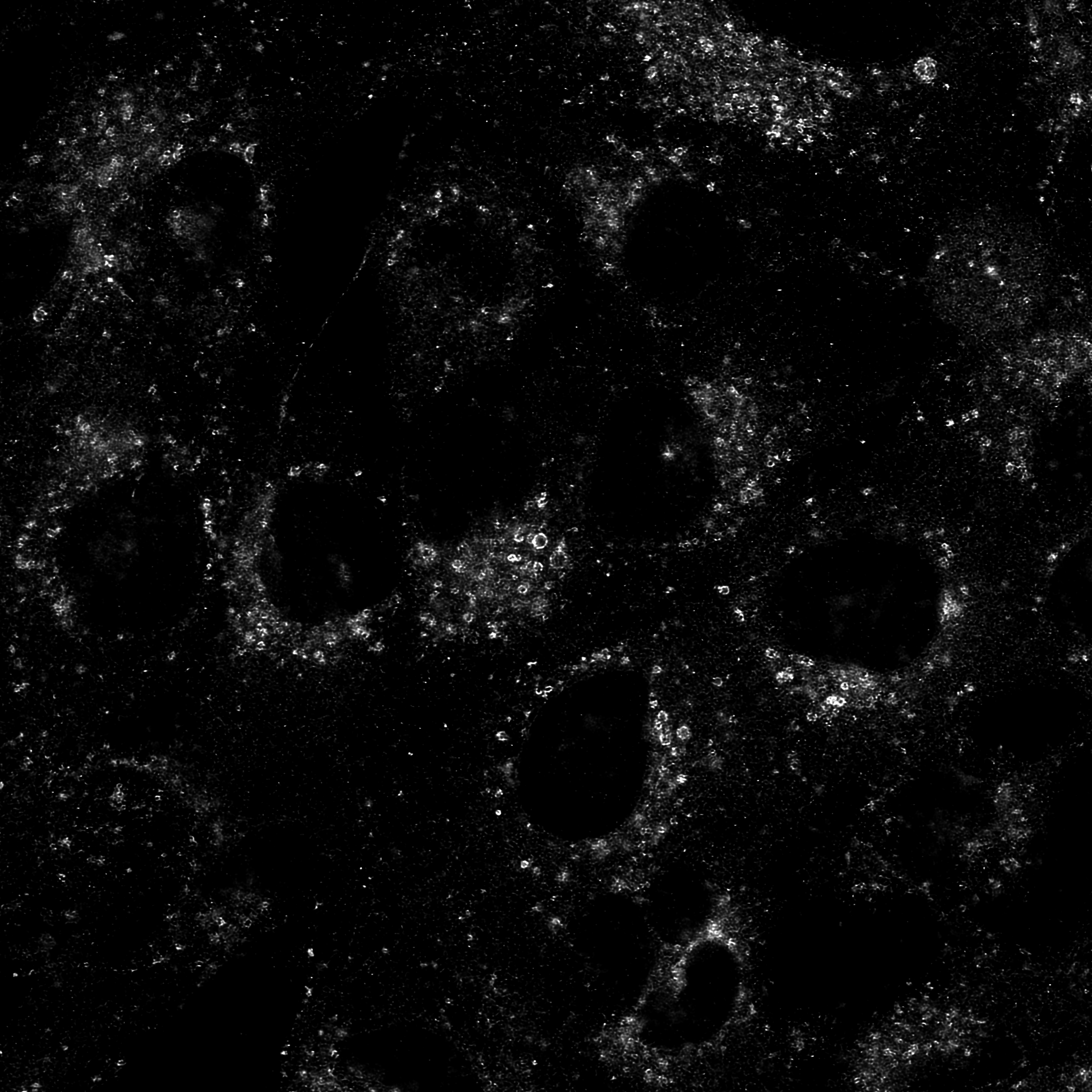

Supplement: Supplementary file 6 — Source data Fig. 2 [file 44319_2026_773_MOESM6_ESM.zip › Figure 2B/IF GRASP55KO LAMP2.tif]

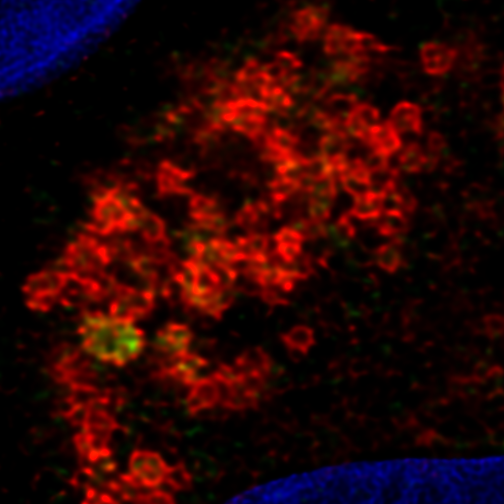

Supplement: Supplementary file 6 — Source data Fig. 2 [file 44319_2026_773_MOESM6_ESM.zip › Figure 2B/IF GNPTABKO CTSD_LAMP2 MERGE Inset.tif]

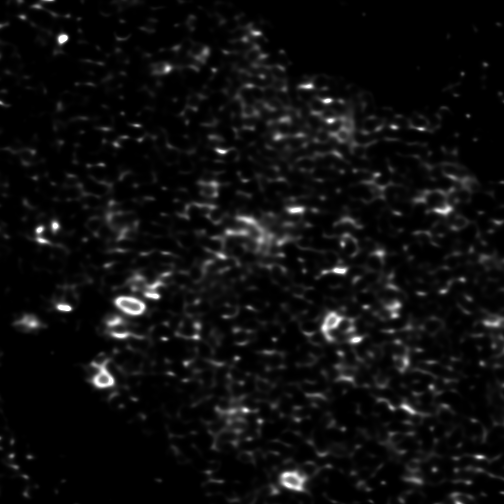

Supplement: Supplementary file 6 — Source data Fig. 2 [file 44319_2026_773_MOESM6_ESM.zip › Figure 2B/IF WT CTSD Inset.tif]

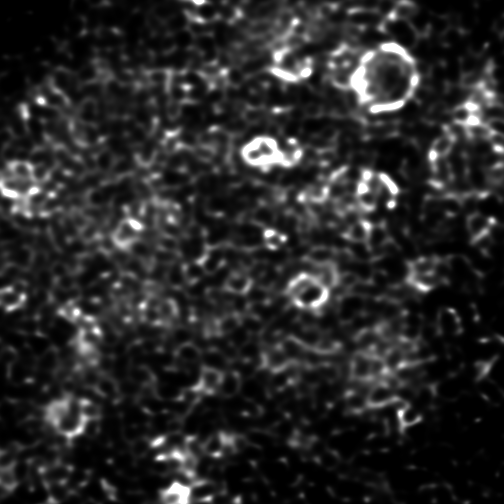

Supplement: Supplementary file 6 — Source data Fig. 2 [file 44319_2026_773_MOESM6_ESM.zip › Figure 2B/IF GRASP55KO LAMP2 inset.tif]

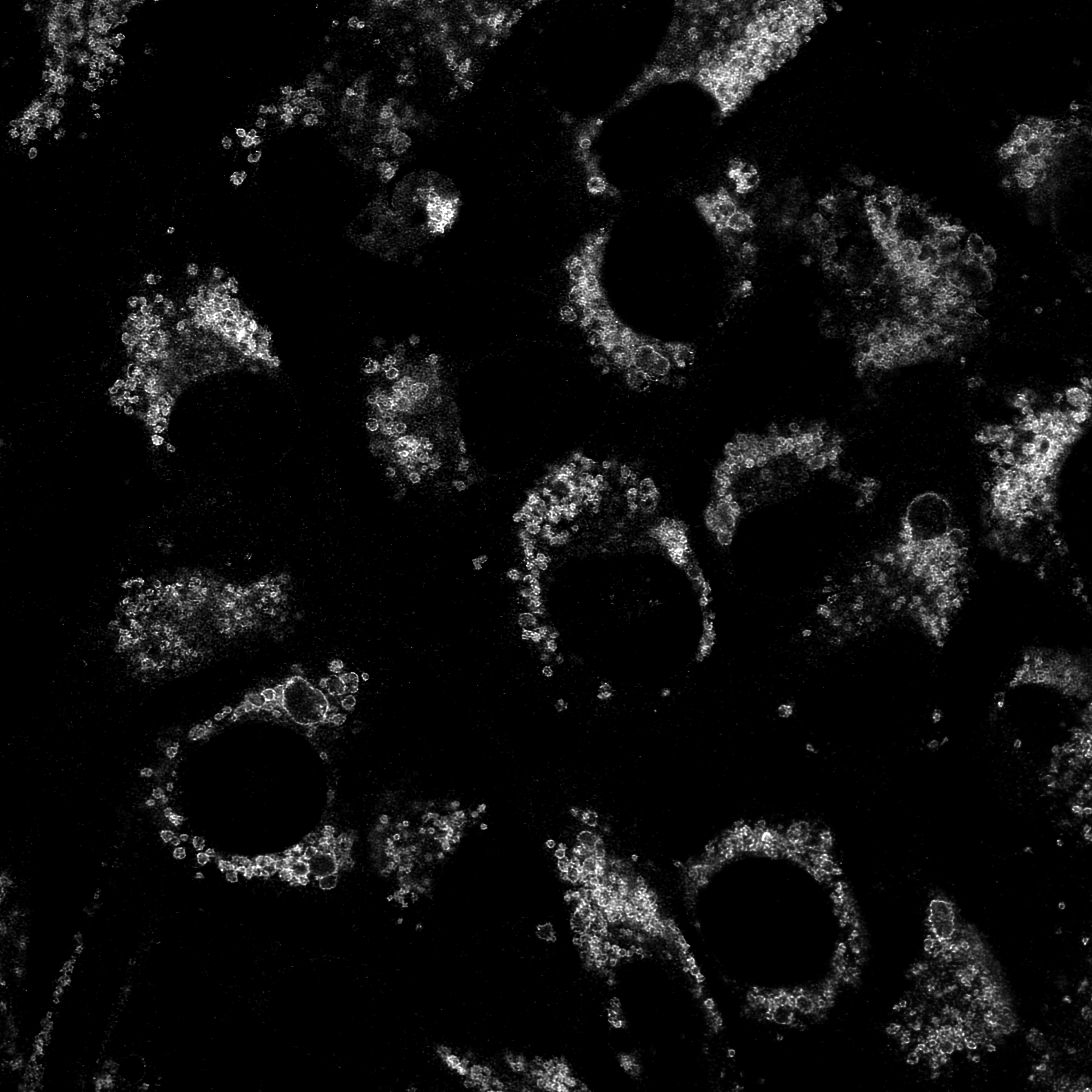

Supplement: Supplementary file 6 — Source data Fig. 2 [file 44319_2026_773_MOESM6_ESM.zip › Figure 2B/IF GNPTABKO LAMP2.tif]

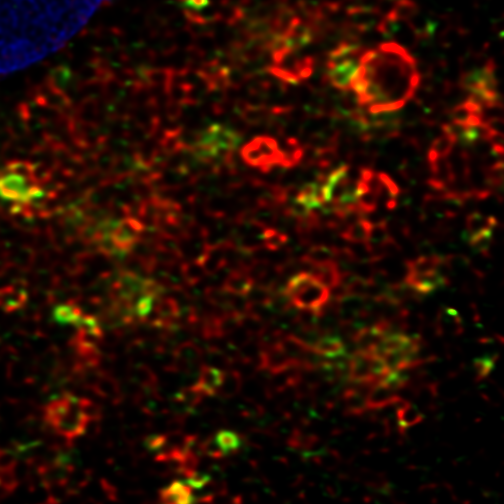

Supplement: Supplementary file 6 — Source data Fig. 2 [file 44319_2026_773_MOESM6_ESM.zip › Figure 2B/IF GRASP55KO CTSD_LAMP2 MERGE inset.tif]

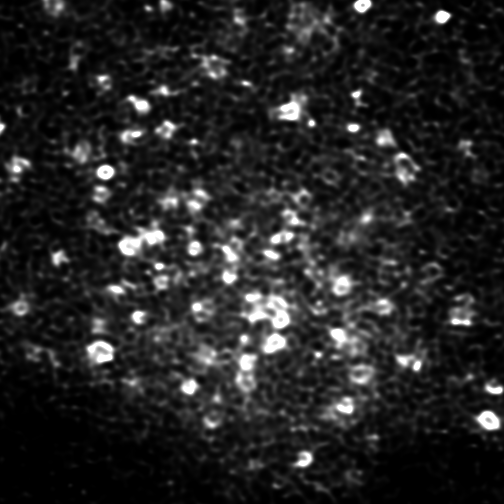

Supplement: Supplementary file 6 — Source data Fig. 2 [file 44319_2026_773_MOESM6_ESM.zip › Figure 2B/IF GRASP65KO LAMP2 inset.tif]

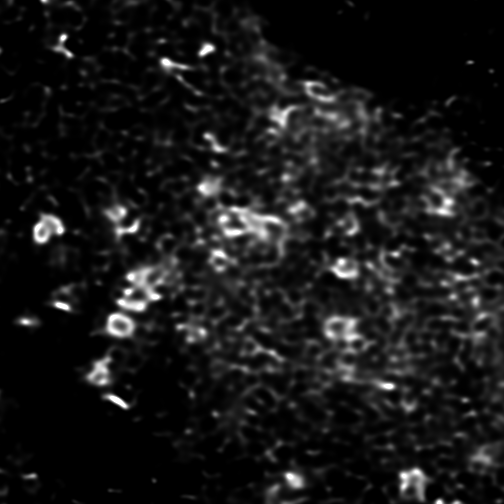

Supplement: Supplementary file 6 — Source data Fig. 2 [file 44319_2026_773_MOESM6_ESM.zip › Figure 2B/IF WT LAMP2 Inset .tif]

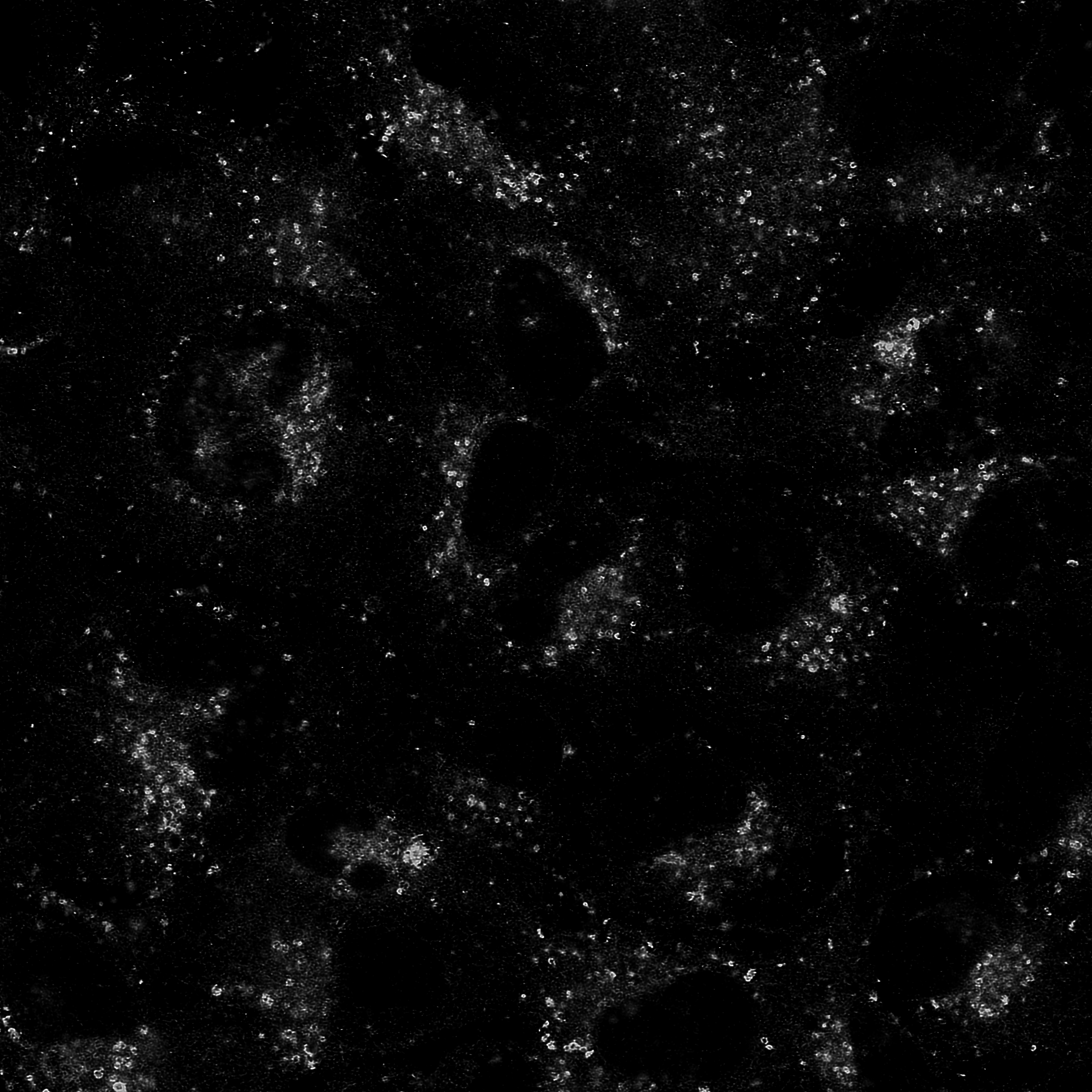

Supplement: Supplementary file 6 — Source data Fig. 2 [file 44319_2026_773_MOESM6_ESM.zip › Figure 2B/IF WT LAMP2.tif]

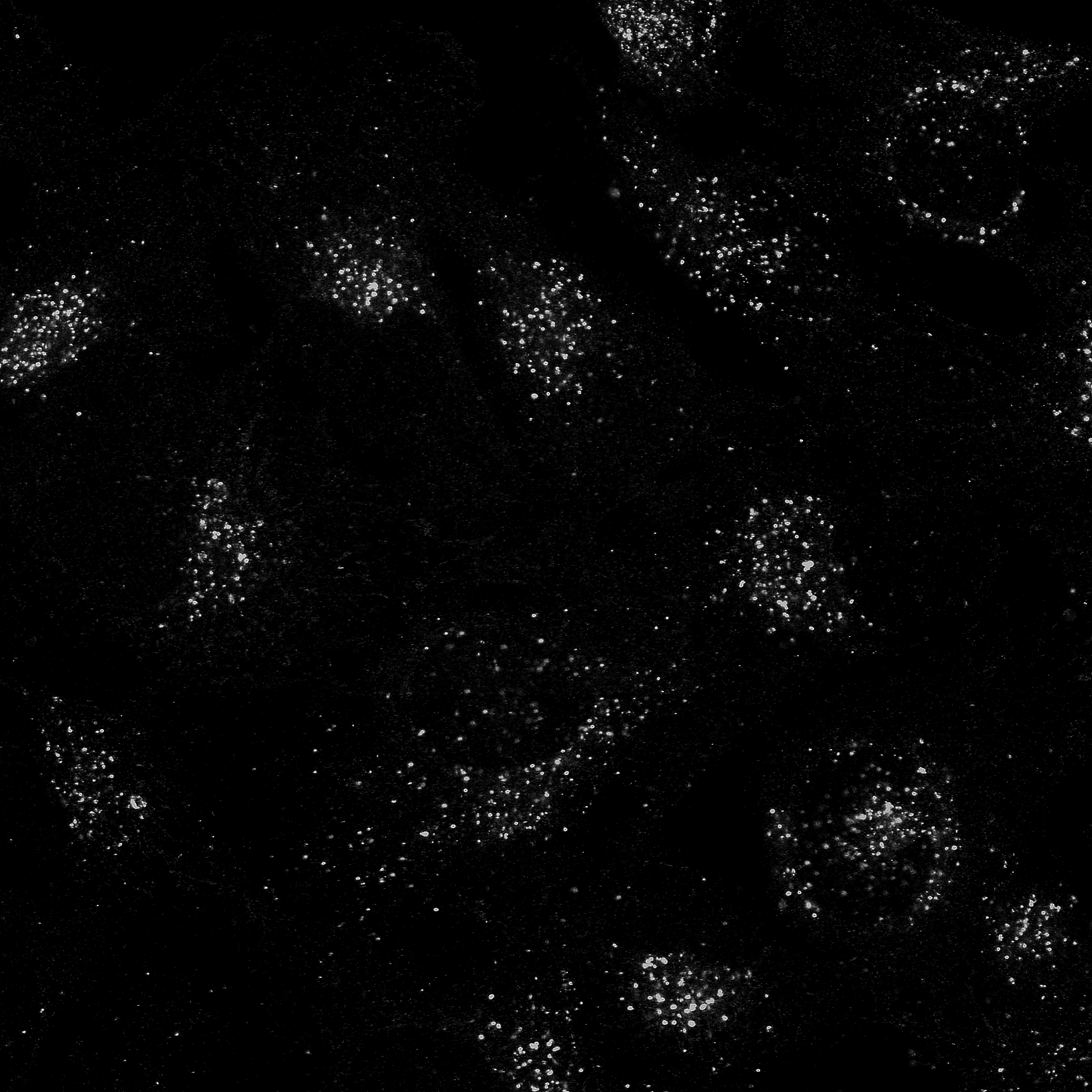

Supplement: Supplementary file 6 — Source data Fig. 2 [file 44319_2026_773_MOESM6_ESM.zip › Figure 2B/IF GRASP65KO CTSD.tif]

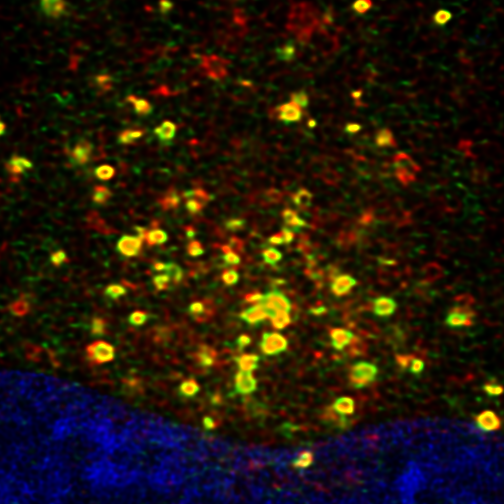

Supplement: Supplementary file 6 — Source data Fig. 2 [file 44319_2026_773_MOESM6_ESM.zip › Figure 2B/IF GRASP65KO CTSD_LAMP2 MERGE inset.tif]

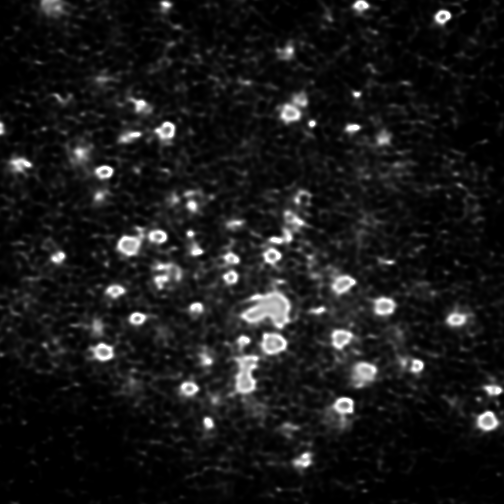

Supplement: Supplementary file 6 — Source data Fig. 2 [file 44319_2026_773_MOESM6_ESM.zip › Figure 2B/IF GRASP65KO CTSD inset.tif]

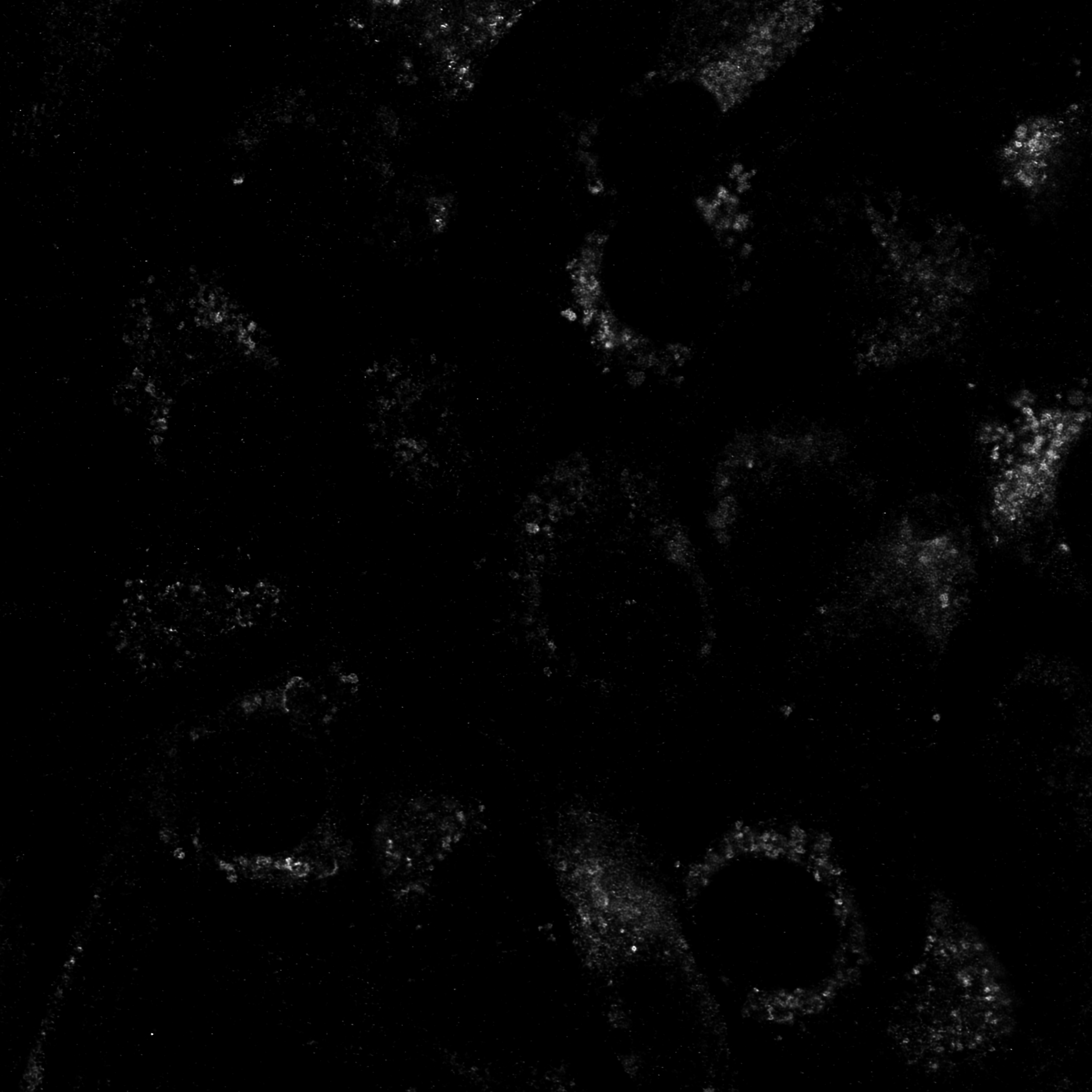

Supplement: Supplementary file 6 — Source data Fig. 2 [file 44319_2026_773_MOESM6_ESM.zip › Figure 2B/IF GNPTABKO CTSD.tif]

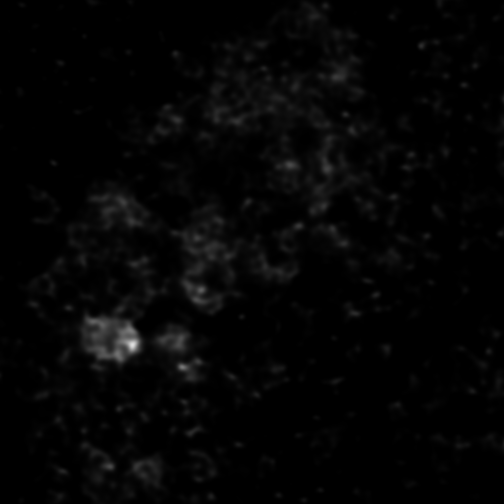

Supplement: Supplementary file 6 — Source data Fig. 2 [file 44319_2026_773_MOESM6_ESM.zip › Figure 2B/IF GNPTABKO CTSD Inset.tif]

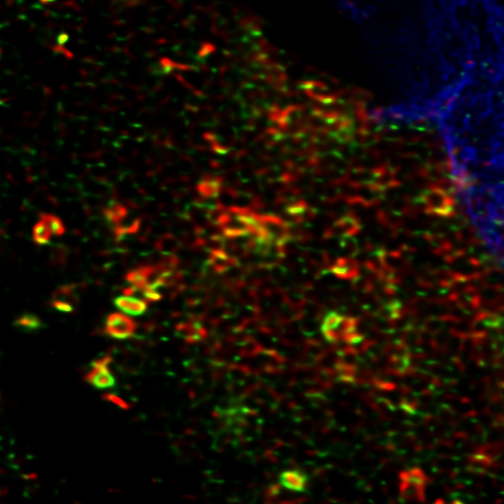

Supplement: Supplementary file 6 — Source data Fig. 2 [file 44319_2026_773_MOESM6_ESM.zip › Figure 2B/IF WT CTSD_LAMP2 MERGE Inset.tiff]

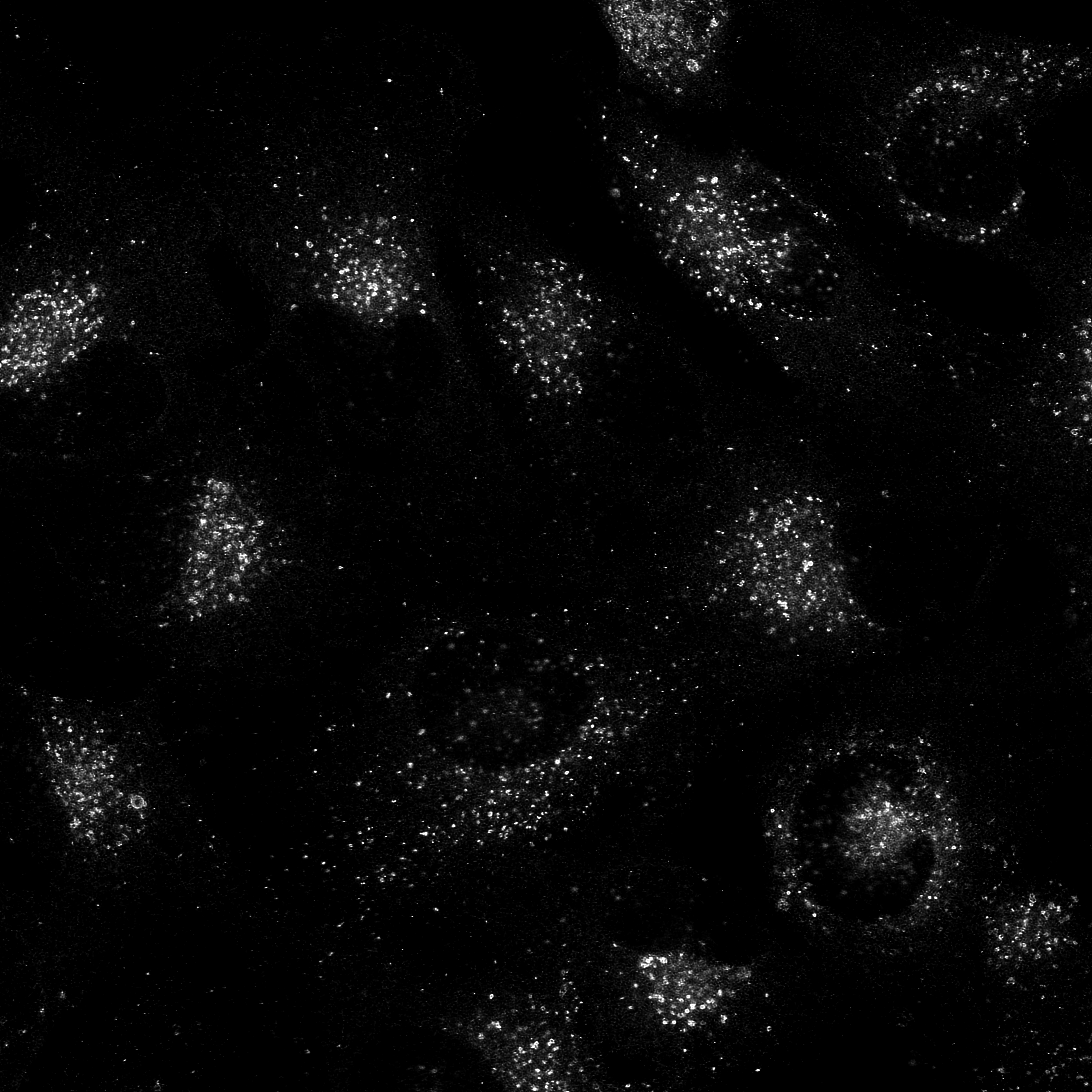

Supplement: Supplementary file 6 — Source data Fig. 2 [file 44319_2026_773_MOESM6_ESM.zip › Figure 2B/IF GRASP65KO LAMP2.tif]

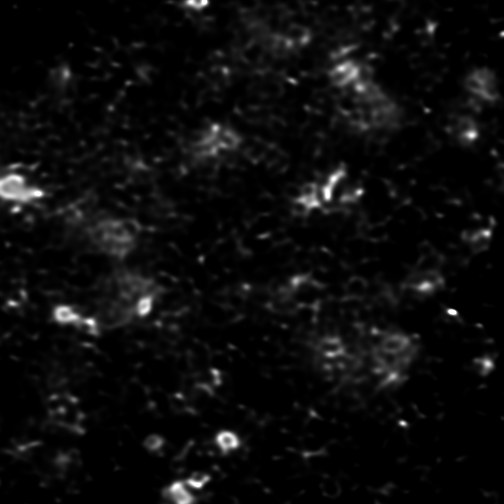

Supplement: Supplementary file 6 — Source data Fig. 2 [file 44319_2026_773_MOESM6_ESM.zip › Figure 2B/IF GRASP55KO CTSD inset.tif]

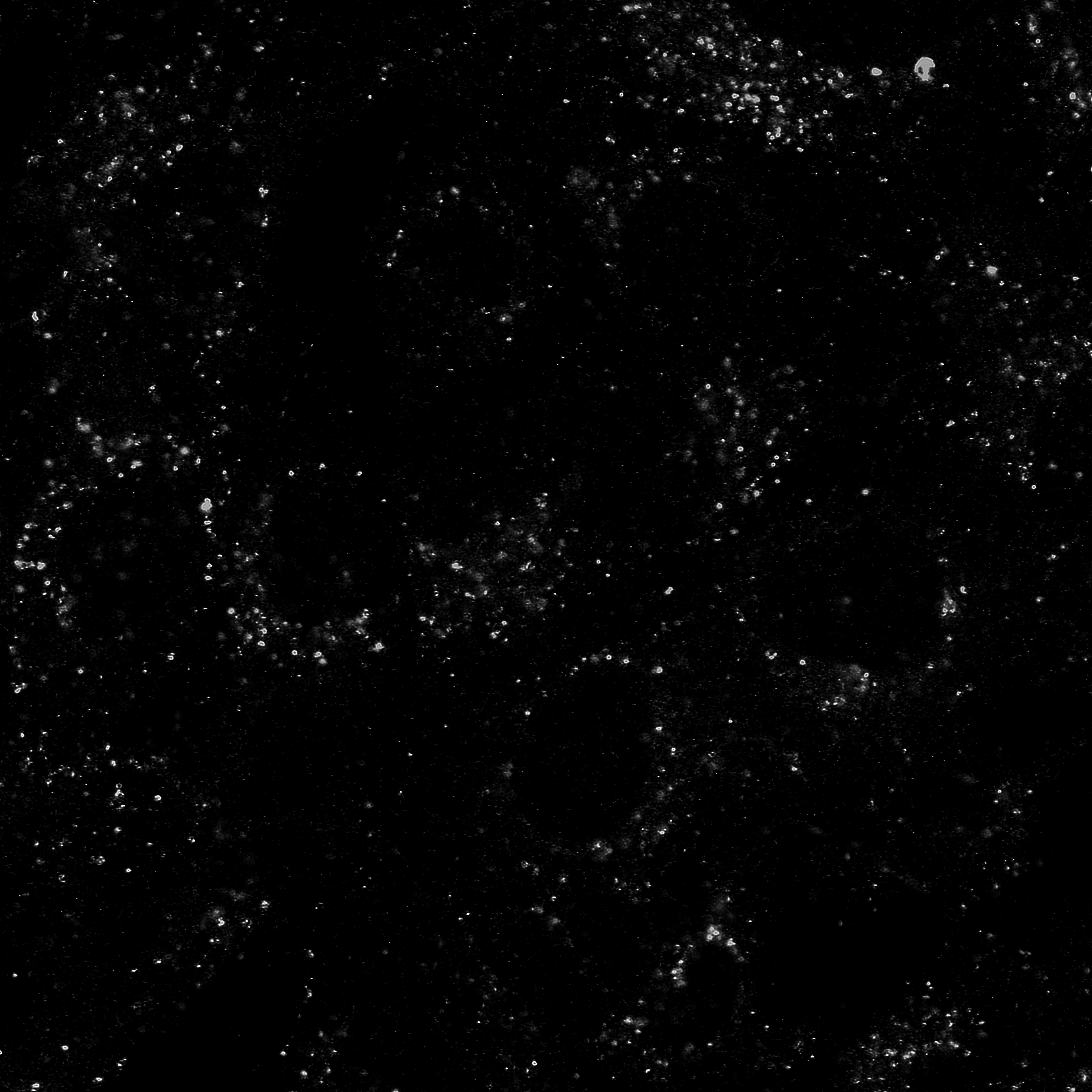

Supplement: Supplementary file 6 — Source data Fig. 2 [file 44319_2026_773_MOESM6_ESM.zip › Figure 2B/IF GRASP55KO CTSD.tif]

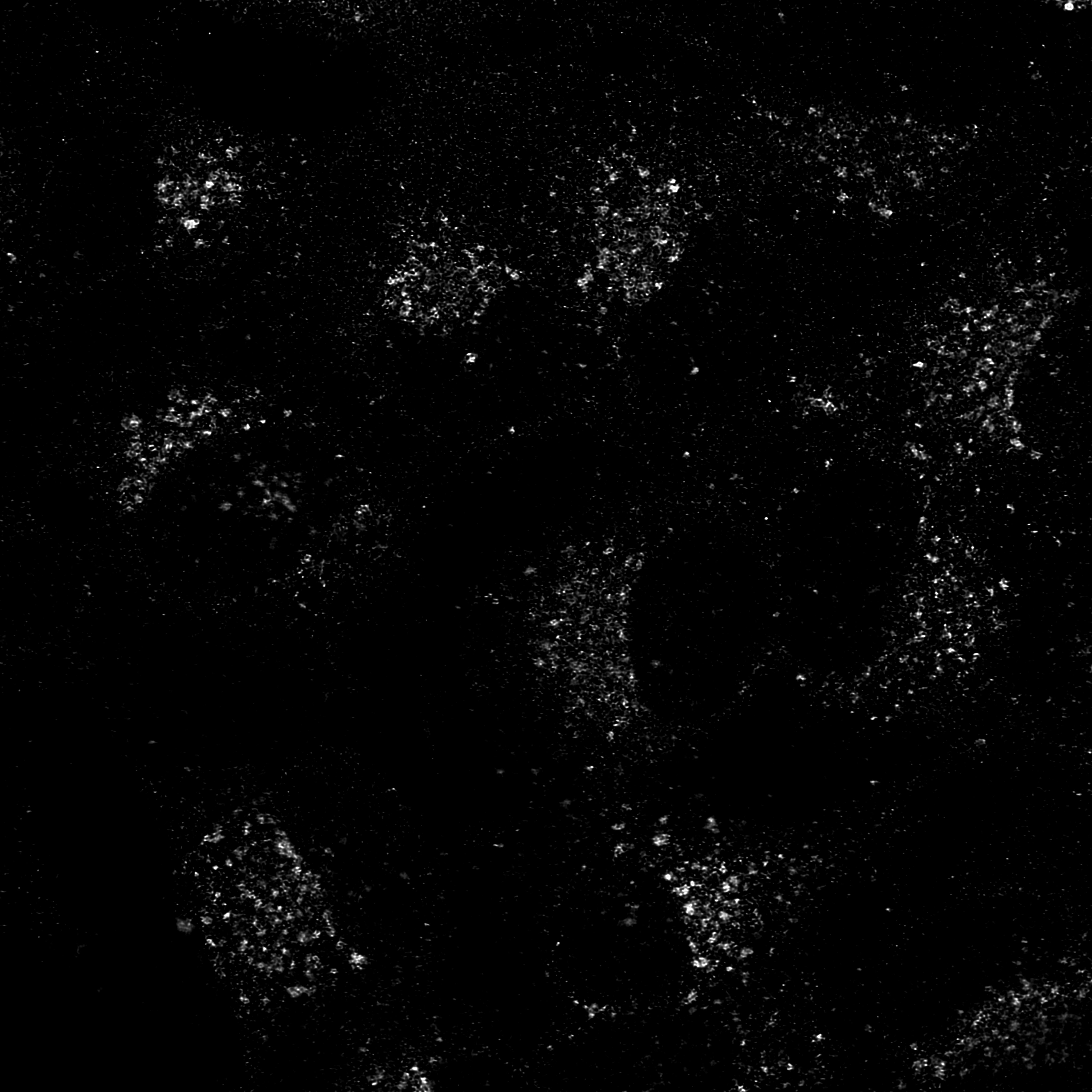

Supplement: Supplementary file 7 — Source data Fig. 2C-H [file 44319_2026_773_MOESM7_ESM.zip › Figure 2C/IF GRASP55KO LAMP2.tif]

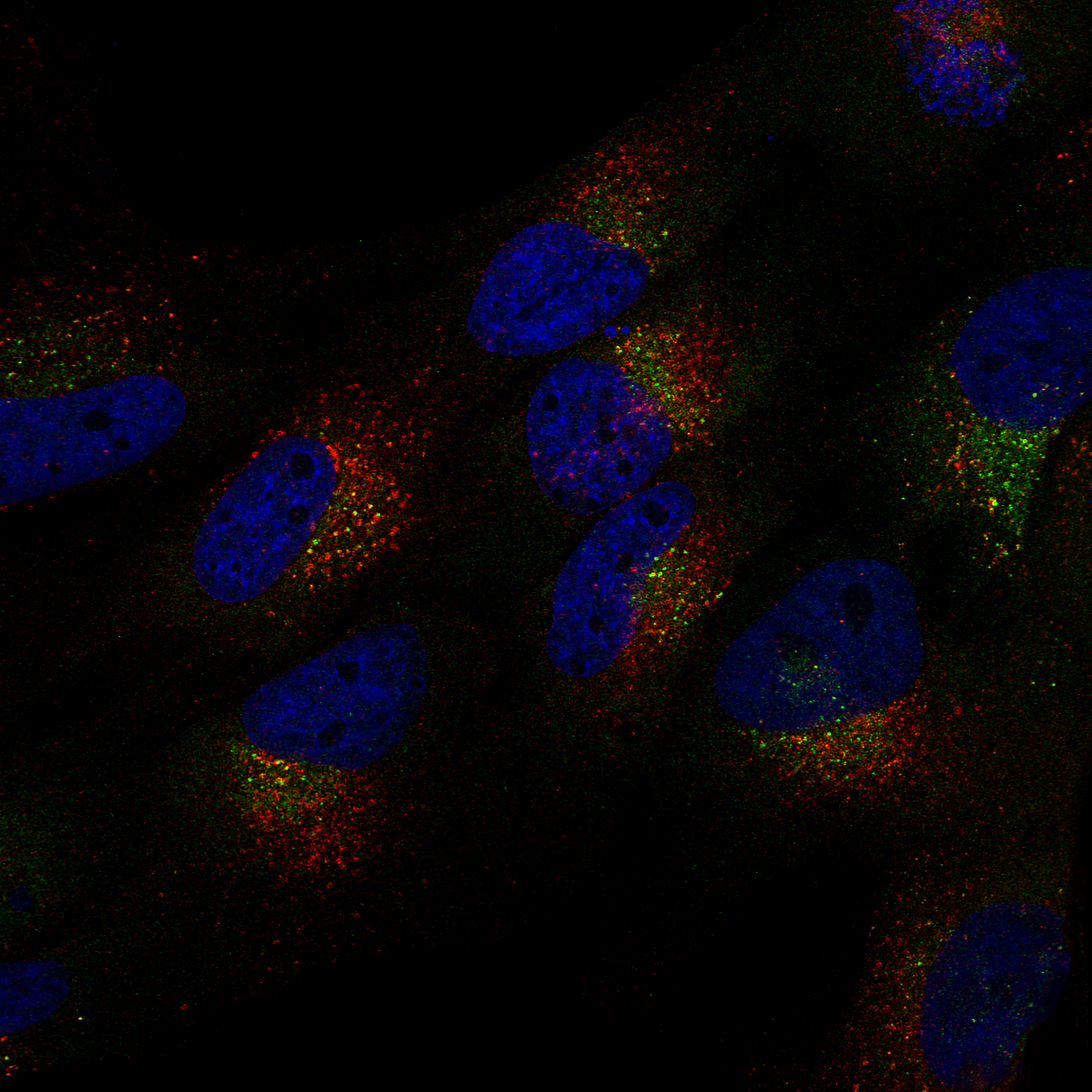

Supplement: Supplementary file 7 — Source data Fig. 2C-H [file 44319_2026_773_MOESM7_ESM.zip › Figure 2C/IF GRASP65KO PSAP_LAMP2 MERGE.tif]

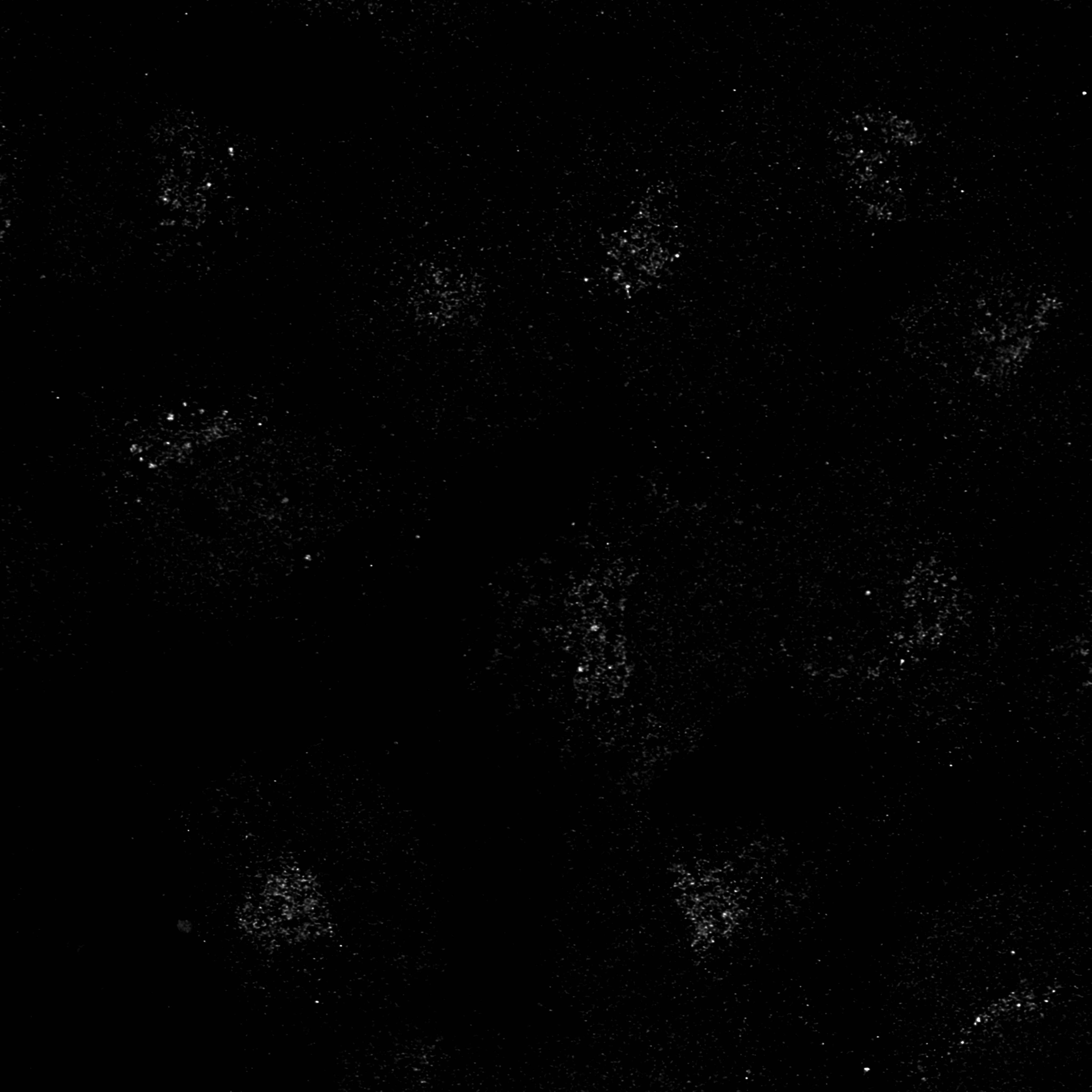

Supplement: Supplementary file 7 — Source data Fig. 2C-H [file 44319_2026_773_MOESM7_ESM.zip › Figure 2C/IF GRASP55KO PSAP.tif]

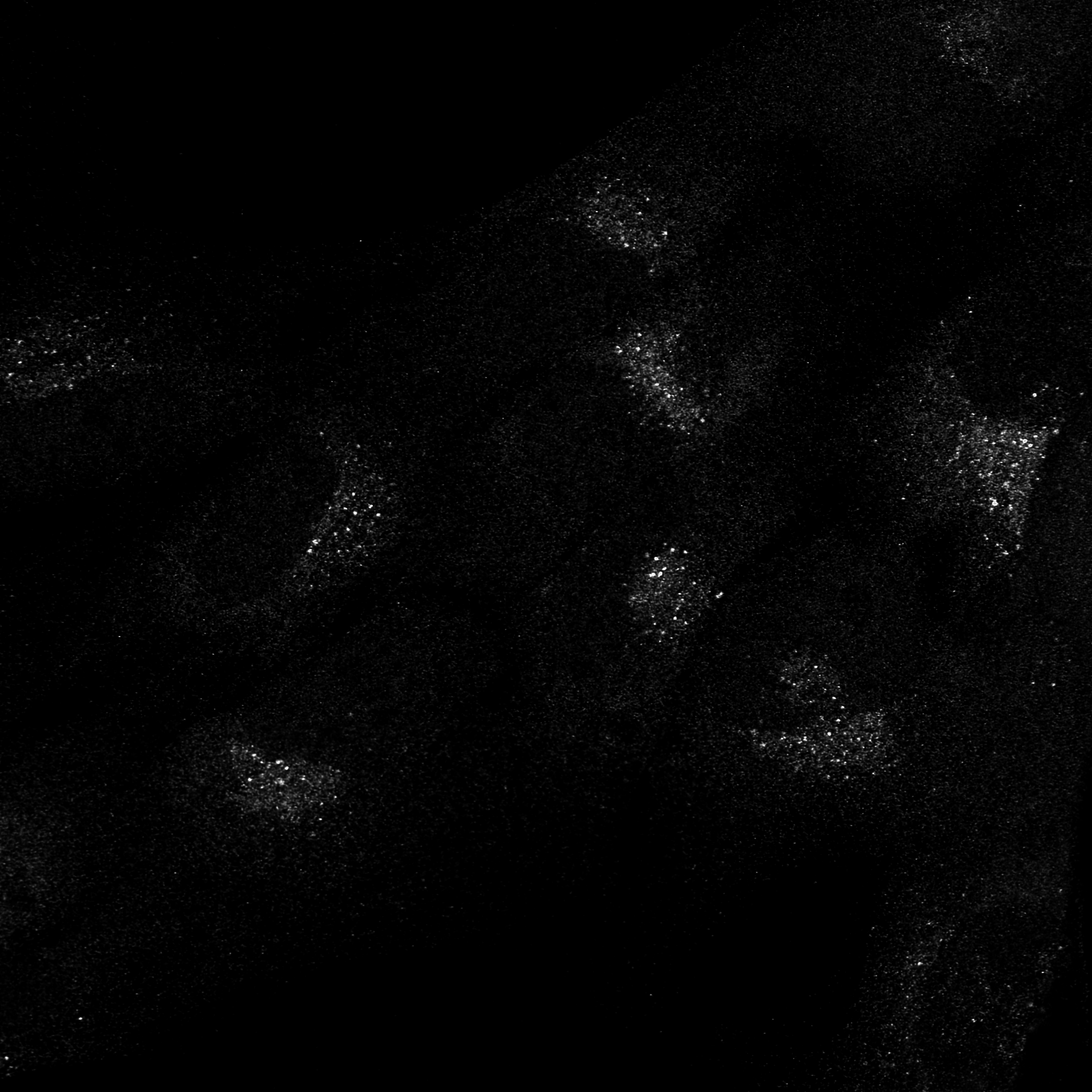

Supplement: Supplementary file 7 — Source data Fig. 2C-H [file 44319_2026_773_MOESM7_ESM.zip › Figure 2C/IF GRASP65KO PSAP.tif]

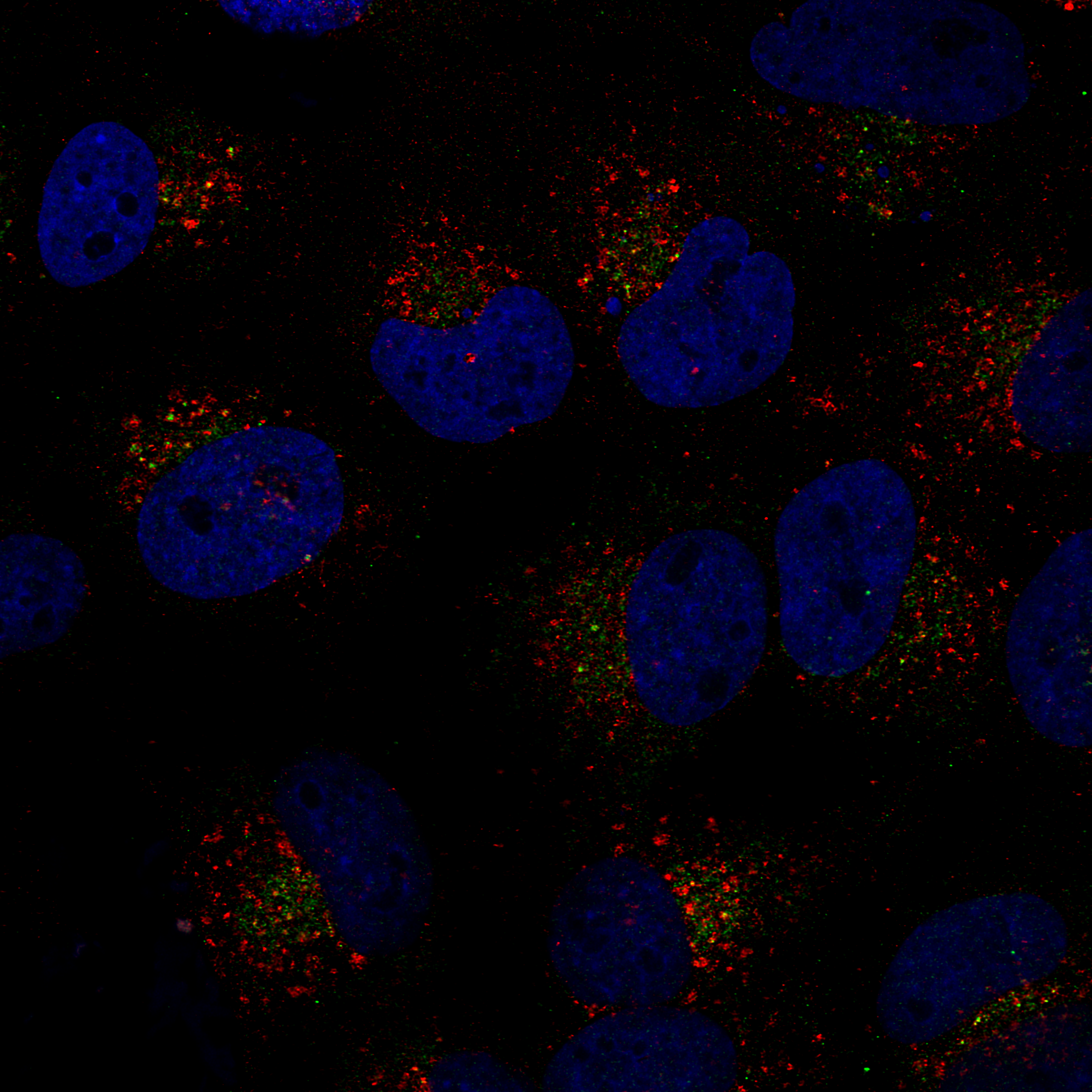

Supplement: Supplementary file 7 — Source data Fig. 2C-H [file 44319_2026_773_MOESM7_ESM.zip › Figure 2C/IF GRASP55KO PSAP_LAMP2 MERGE.tif]

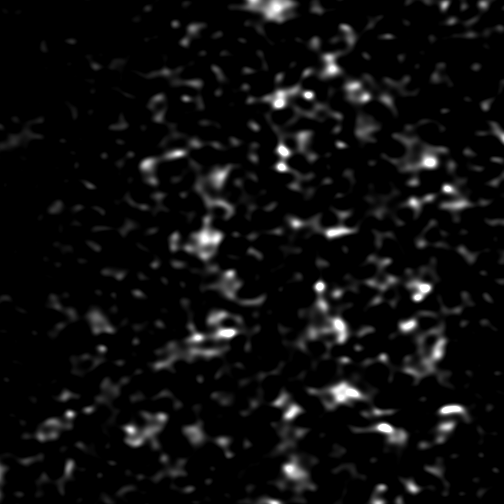

Supplement: Supplementary file 7 — Source data Fig. 2C-H [file 44319_2026_773_MOESM7_ESM.zip › Figure 2C/IF GRASP55KO LAMP2 inset.tif]

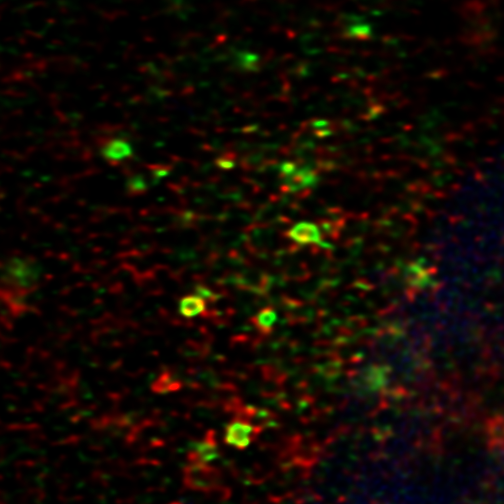

Supplement: Supplementary file 7 — Source data Fig. 2C-H [file 44319_2026_773_MOESM7_ESM.zip › Figure 2C/IF WT PSAP_LAMP2 MERGE inset.tif]

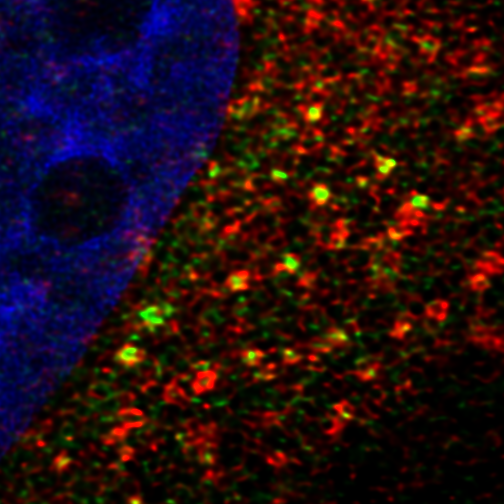

Supplement: Supplementary file 7 — Source data Fig. 2C-H [file 44319_2026_773_MOESM7_ESM.zip › Figure 2C/IF GRASP65KO PSAP_LAMP2 MERGE inset.tif]

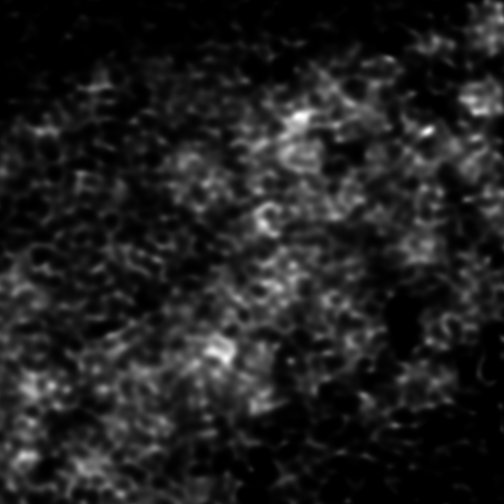

Supplement: Supplementary file 7 — Source data Fig. 2C-H [file 44319_2026_773_MOESM7_ESM.zip › Figure 2C/IF GNPTABKO LAMP2 inset.tif]

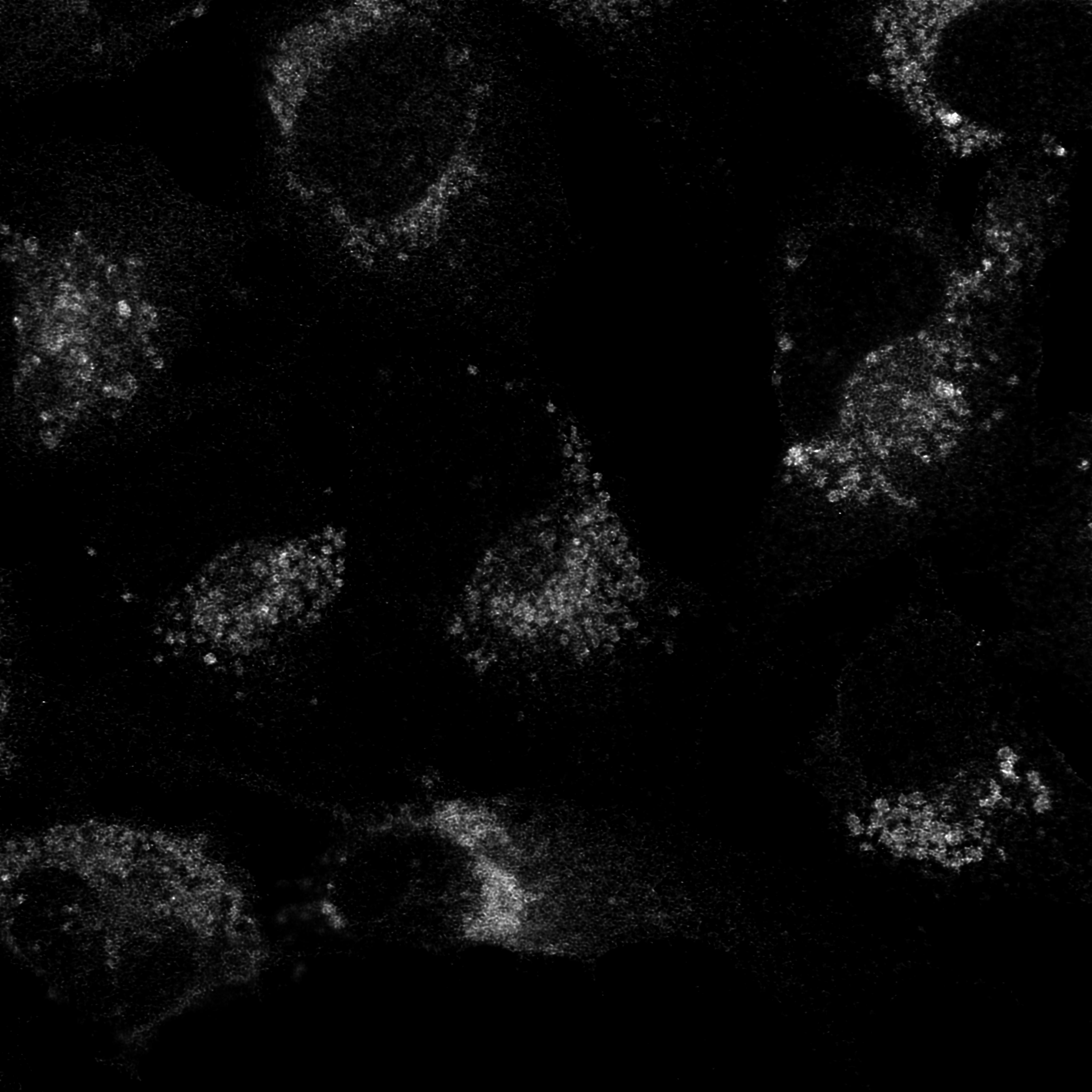

Supplement: Supplementary file 7 — Source data Fig. 2C-H [file 44319_2026_773_MOESM7_ESM.zip › Figure 2C/IF GNPTABKO LAMP2.tif]

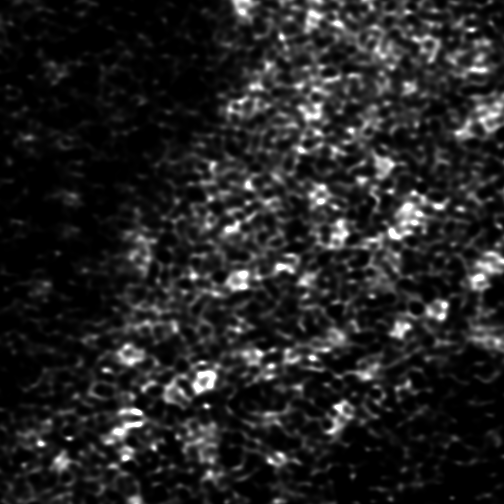

Supplement: Supplementary file 7 — Source data Fig. 2C-H [file 44319_2026_773_MOESM7_ESM.zip › Figure 2C/IF GRASP65KO LAMP2 inset.tif]

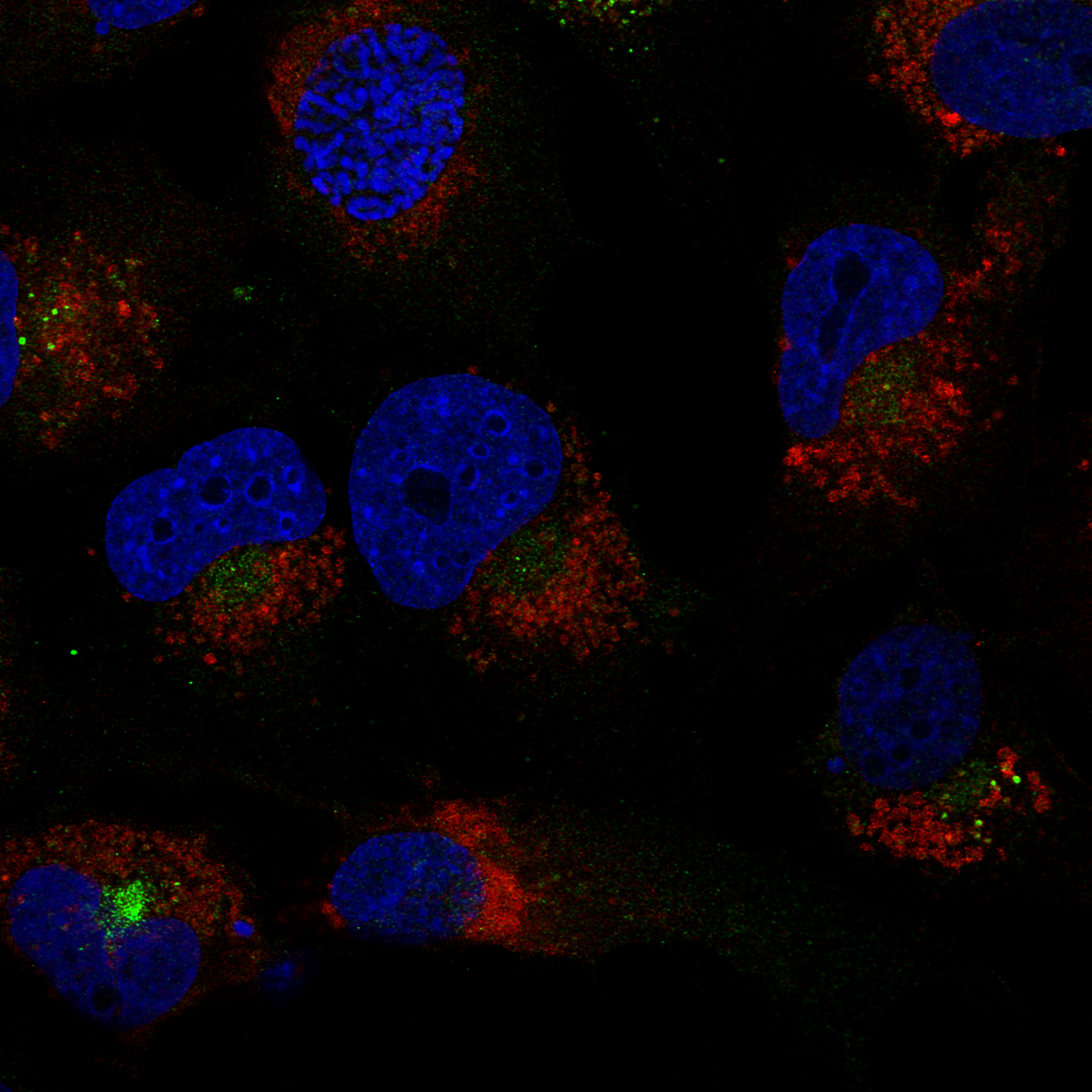

Supplement: Supplementary file 7 — Source data Fig. 2C-H [file 44319_2026_773_MOESM7_ESM.zip › Figure 2C/IF GNPTABKO PSAP_LAMP2 MERGE.tif]

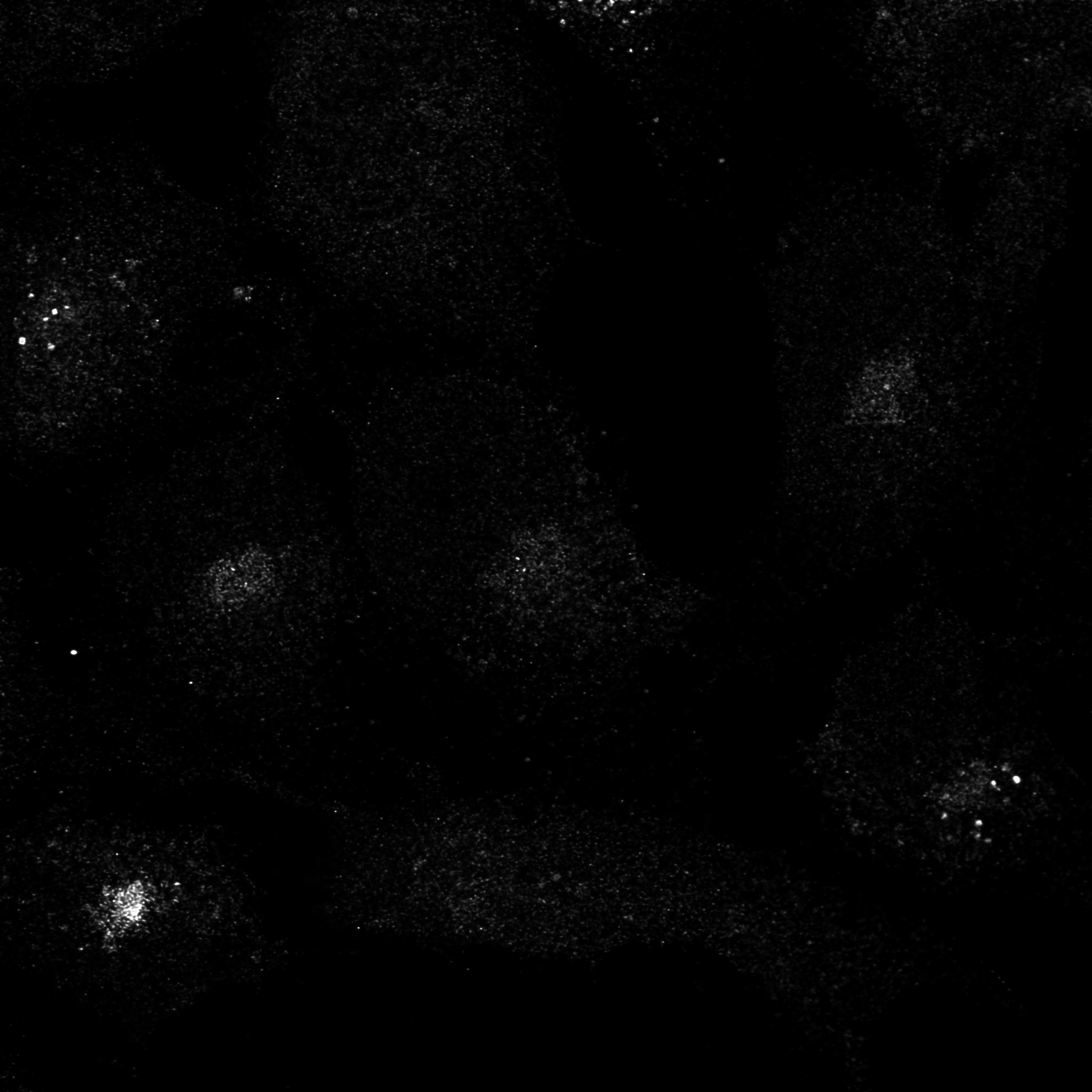

Supplement: Supplementary file 7 — Source data Fig. 2C-H [file 44319_2026_773_MOESM7_ESM.zip › Figure 2C/IF GNPTABKO PSAP.tif]

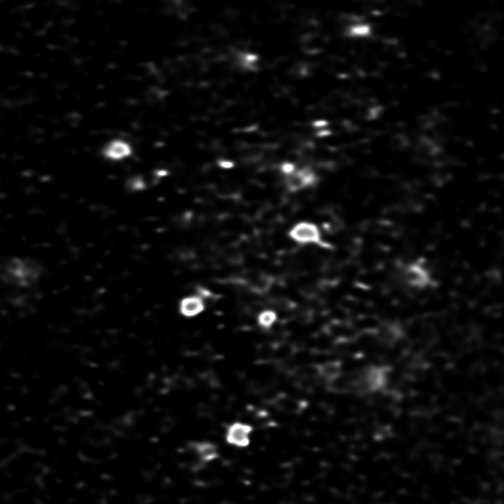

Supplement: Supplementary file 7 — Source data Fig. 2C-H [file 44319_2026_773_MOESM7_ESM.zip › Figure 2C/IF WT PSAP inset.tif]

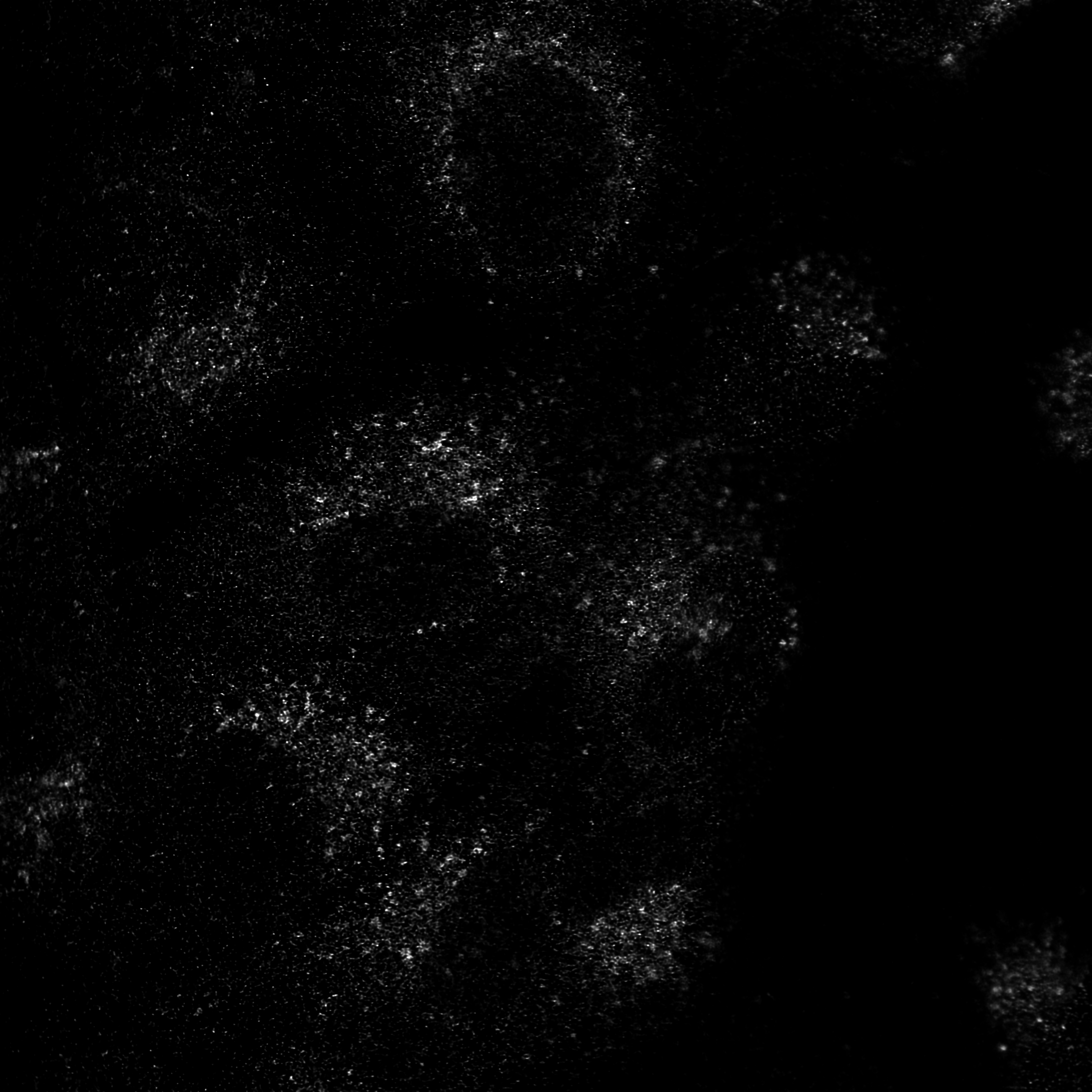

Supplement: Supplementary file 7 — Source data Fig. 2C-H [file 44319_2026_773_MOESM7_ESM.zip › Figure 2C/IF WT LAMP2.tif]

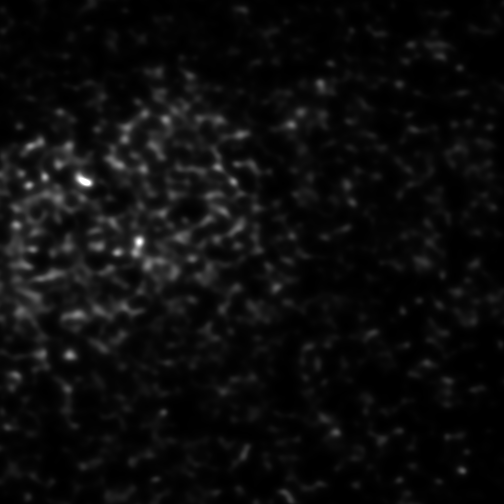

Supplement: Supplementary file 7 — Source data Fig. 2C-H [file 44319_2026_773_MOESM7_ESM.zip › Figure 2C/IF GNPTABKO PSAP inset.tif]

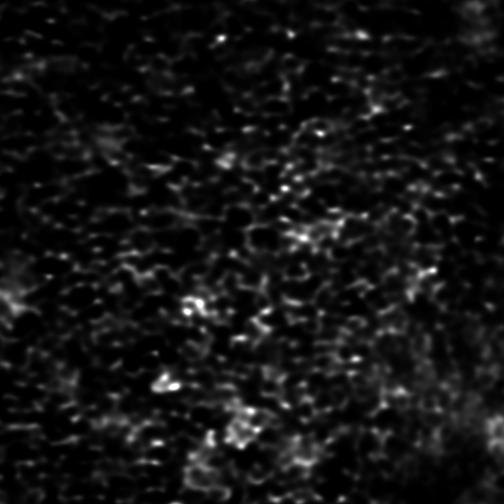

Supplement: Supplementary file 7 — Source data Fig. 2C-H [file 44319_2026_773_MOESM7_ESM.zip › Figure 2C/IF WT LAMP2 inset.tif]

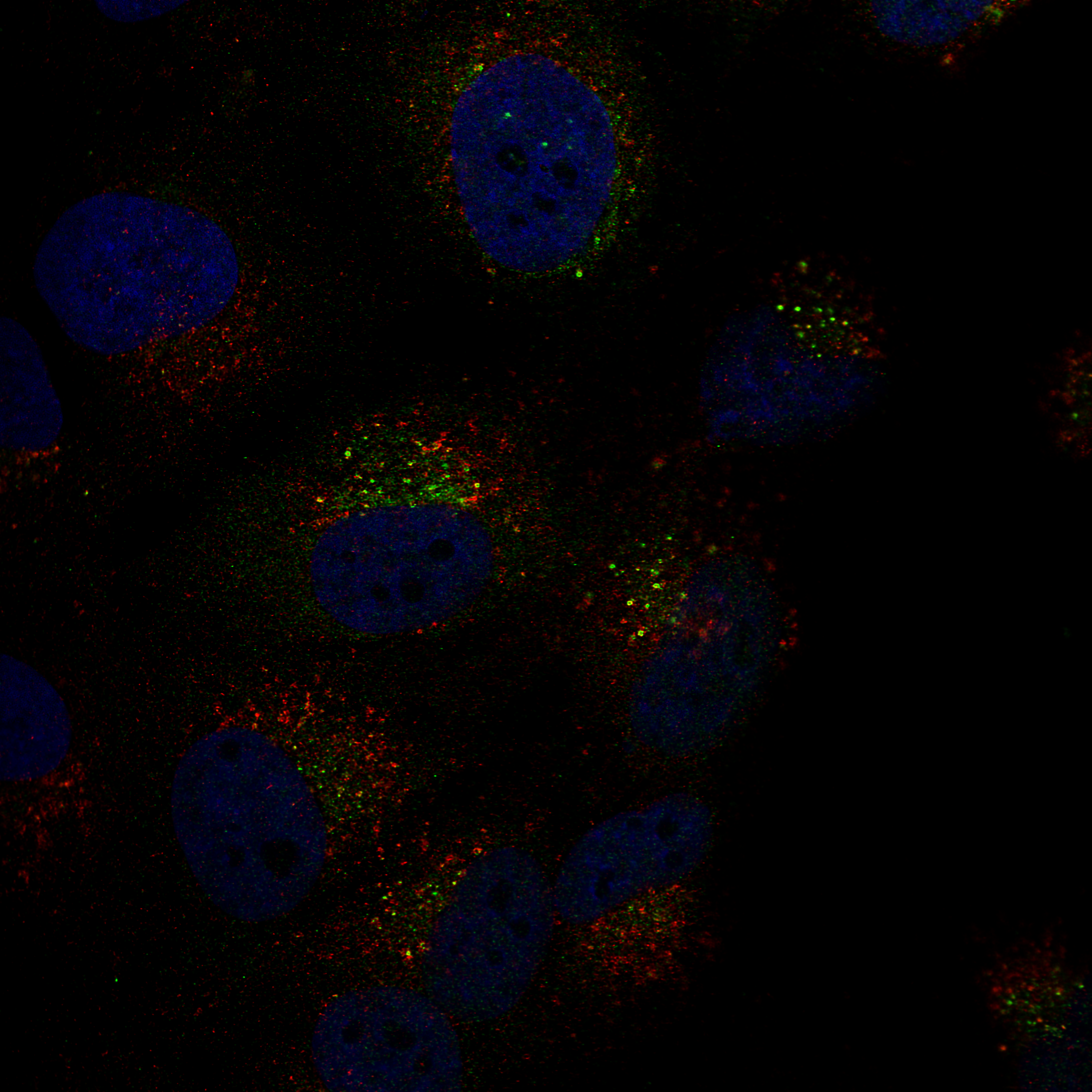

Supplement: Supplementary file 7 — Source data Fig. 2C-H [file 44319_2026_773_MOESM7_ESM.zip › Figure 2C/IF WT PSAP_LAMP2 MERGE.tif]

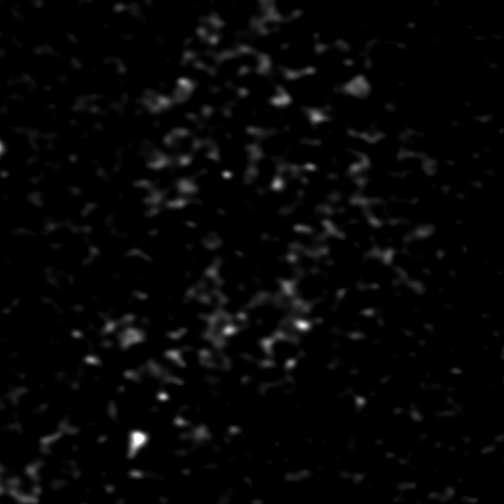

Supplement: Supplementary file 7 — Source data Fig. 2C-H [file 44319_2026_773_MOESM7_ESM.zip › Figure 2C/IF GRASP55KO PSAP inset.tif]

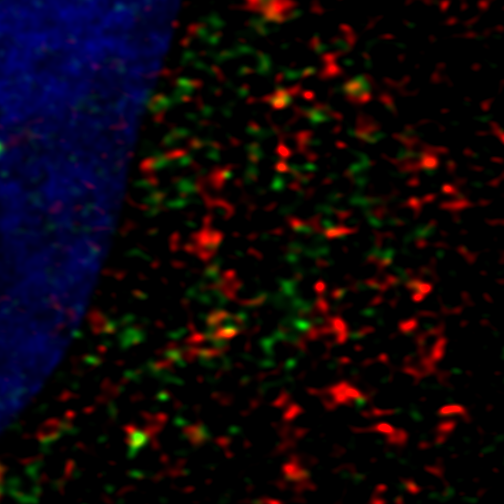

Supplement: Supplementary file 7 — Source data Fig. 2C-H [file 44319_2026_773_MOESM7_ESM.zip › Figure 2C/IF GRASP55KO PSAP_LAMP2 MERGE inset.tif]

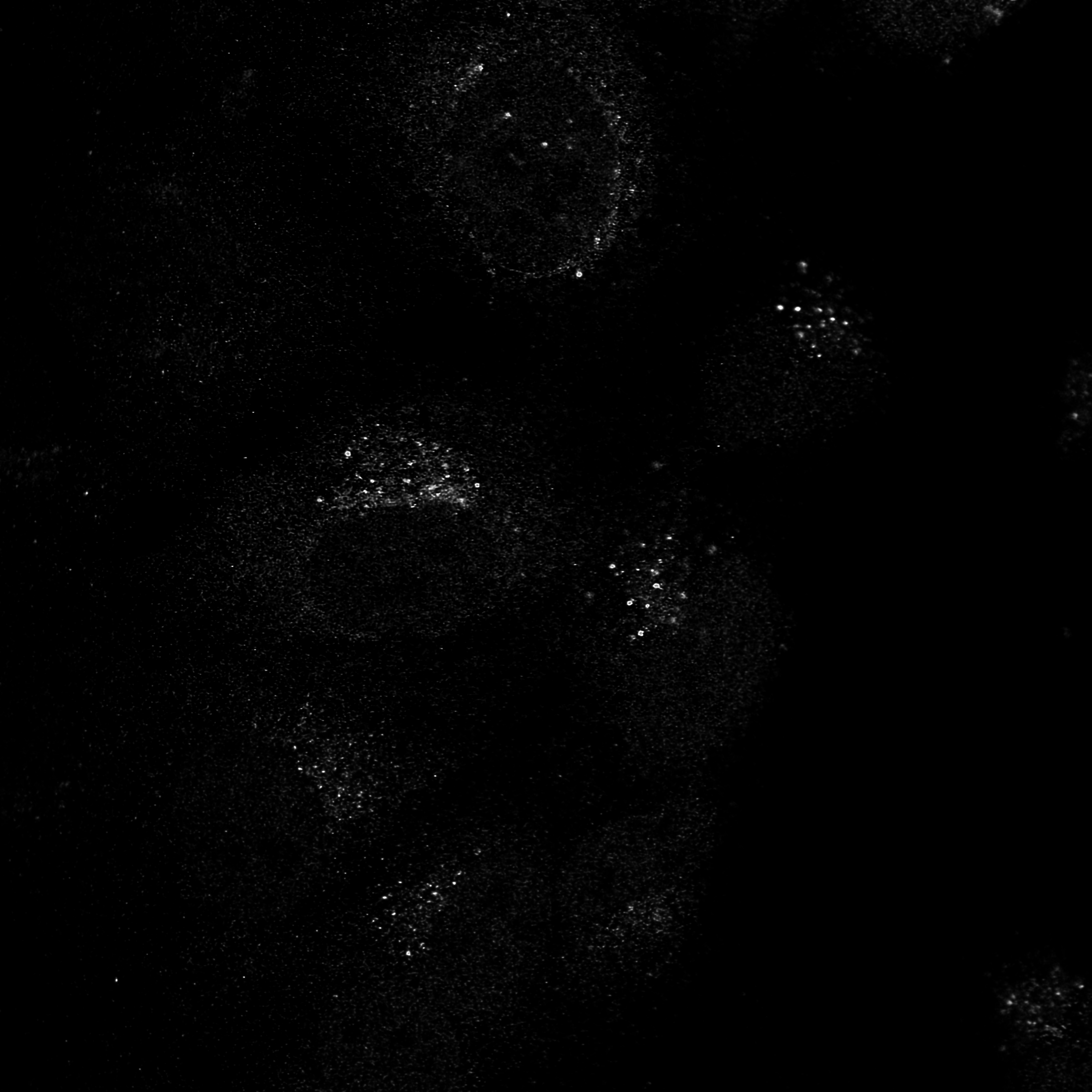

Supplement: Supplementary file 7 — Source data Fig. 2C-H [file 44319_2026_773_MOESM7_ESM.zip › Figure 2C/IF WT PSAP.tif]

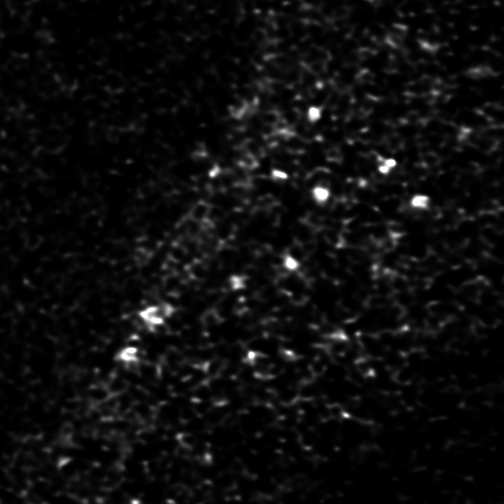

Supplement: Supplementary file 7 — Source data Fig. 2C-H [file 44319_2026_773_MOESM7_ESM.zip › Figure 2C/IF GRASP65KO PSAP inset.tif]

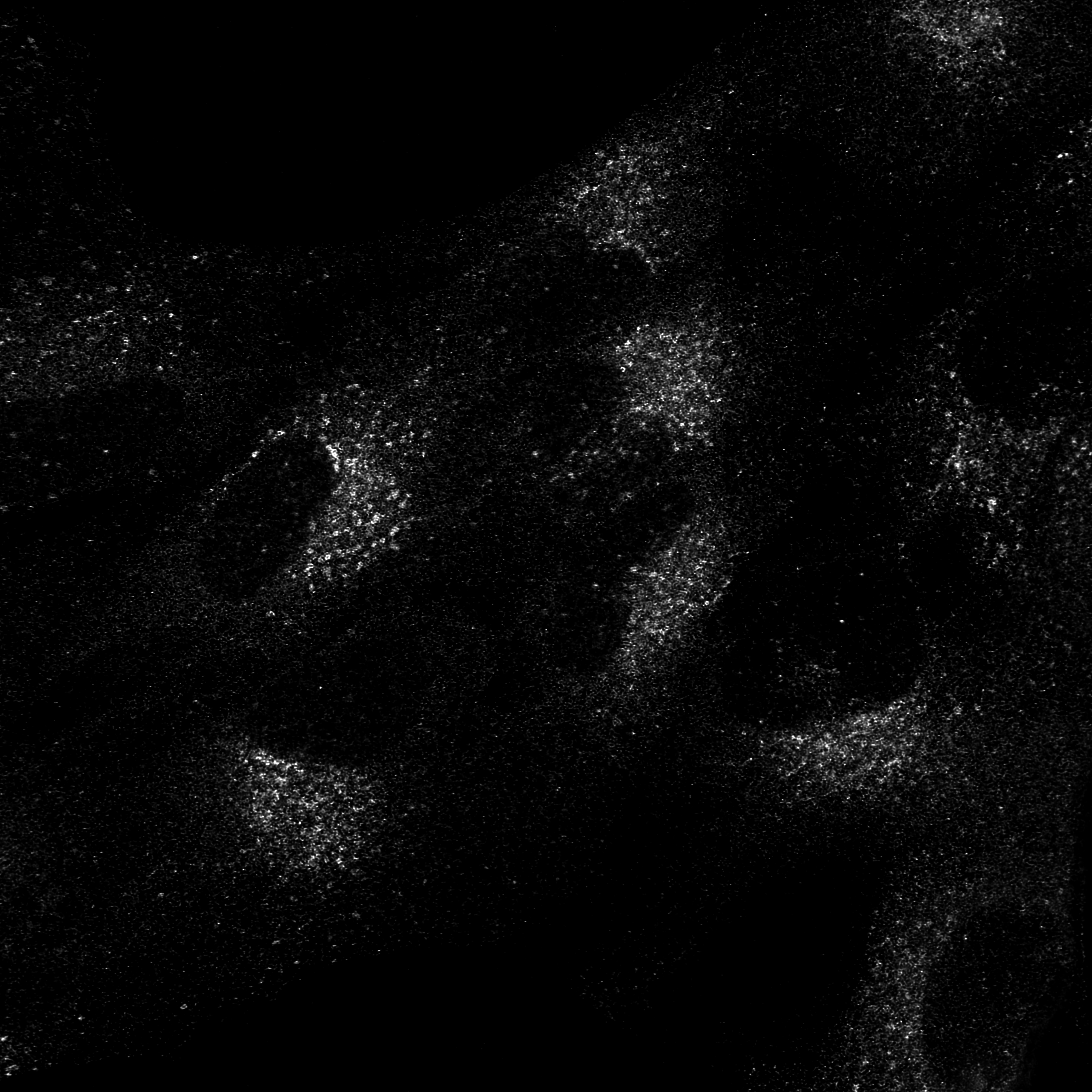

Supplement: Supplementary file 7 — Source data Fig. 2C-H [file 44319_2026_773_MOESM7_ESM.zip › Figure 2C/IF GRASP65KO LAMP2.tif]

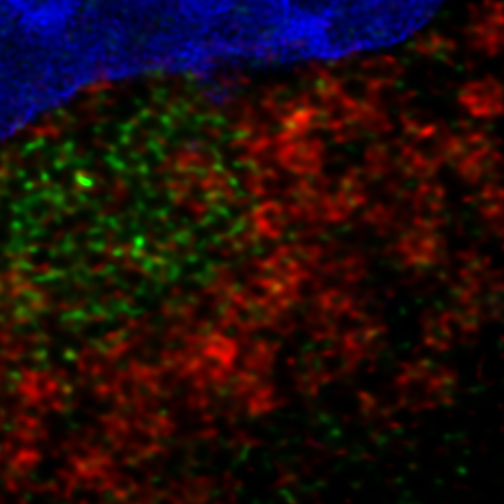

Supplement: Supplementary file 7 — Source data Fig. 2C-H [file 44319_2026_773_MOESM7_ESM.zip › Figure 2C/IF GNPTABKO PSAP_LAMP2 MERGE inset.tif]

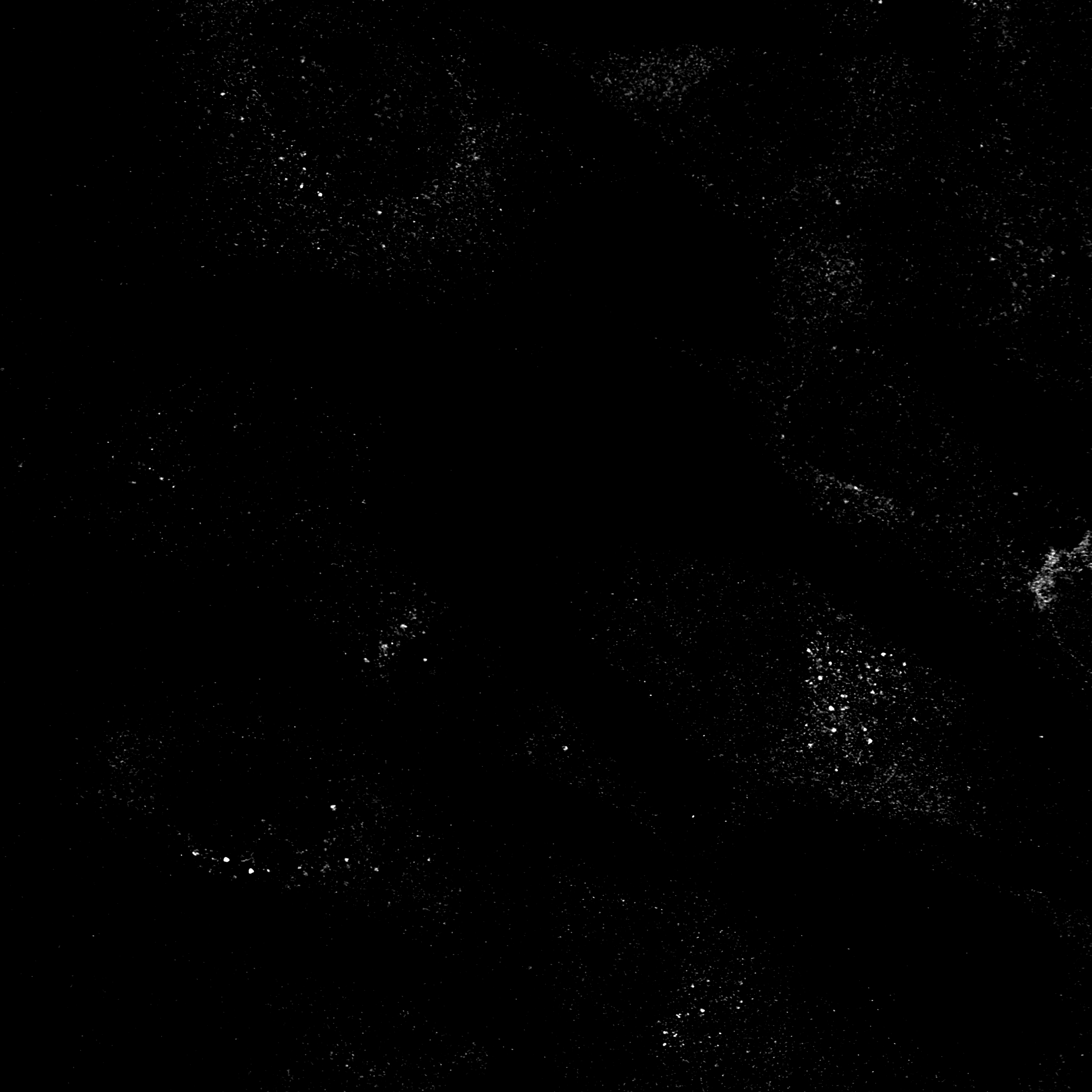

Supplement: Supplementary file 7 — Source data Fig. 2C-H [file 44319_2026_773_MOESM7_ESM.zip › Figure 2D/IF GRASP65KO GRN.tif]

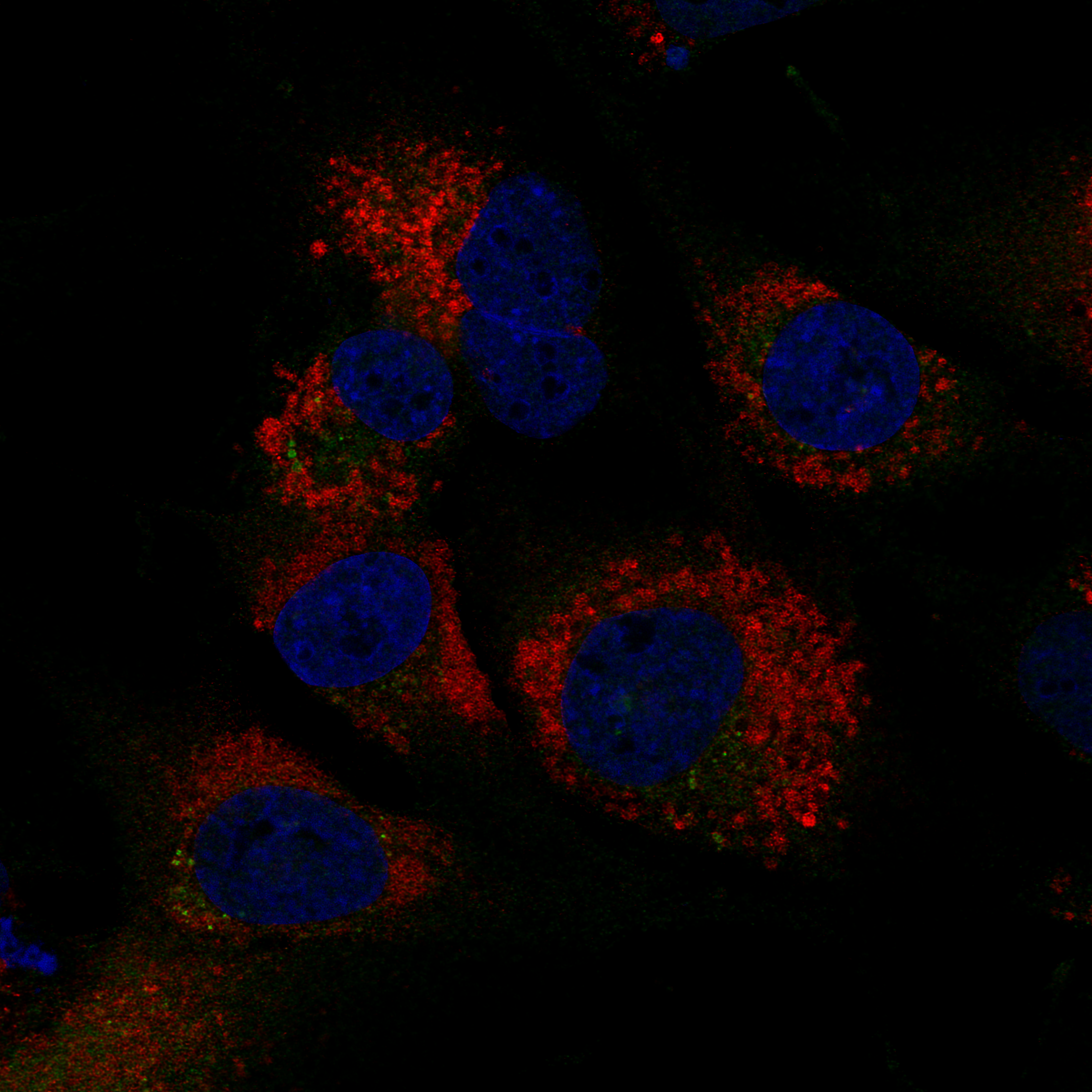

Supplement: Supplementary file 7 — Source data Fig. 2C-H [file 44319_2026_773_MOESM7_ESM.zip › Figure 2D/IF GNPTABKO GRN_LAMP2 MERGE.tif]

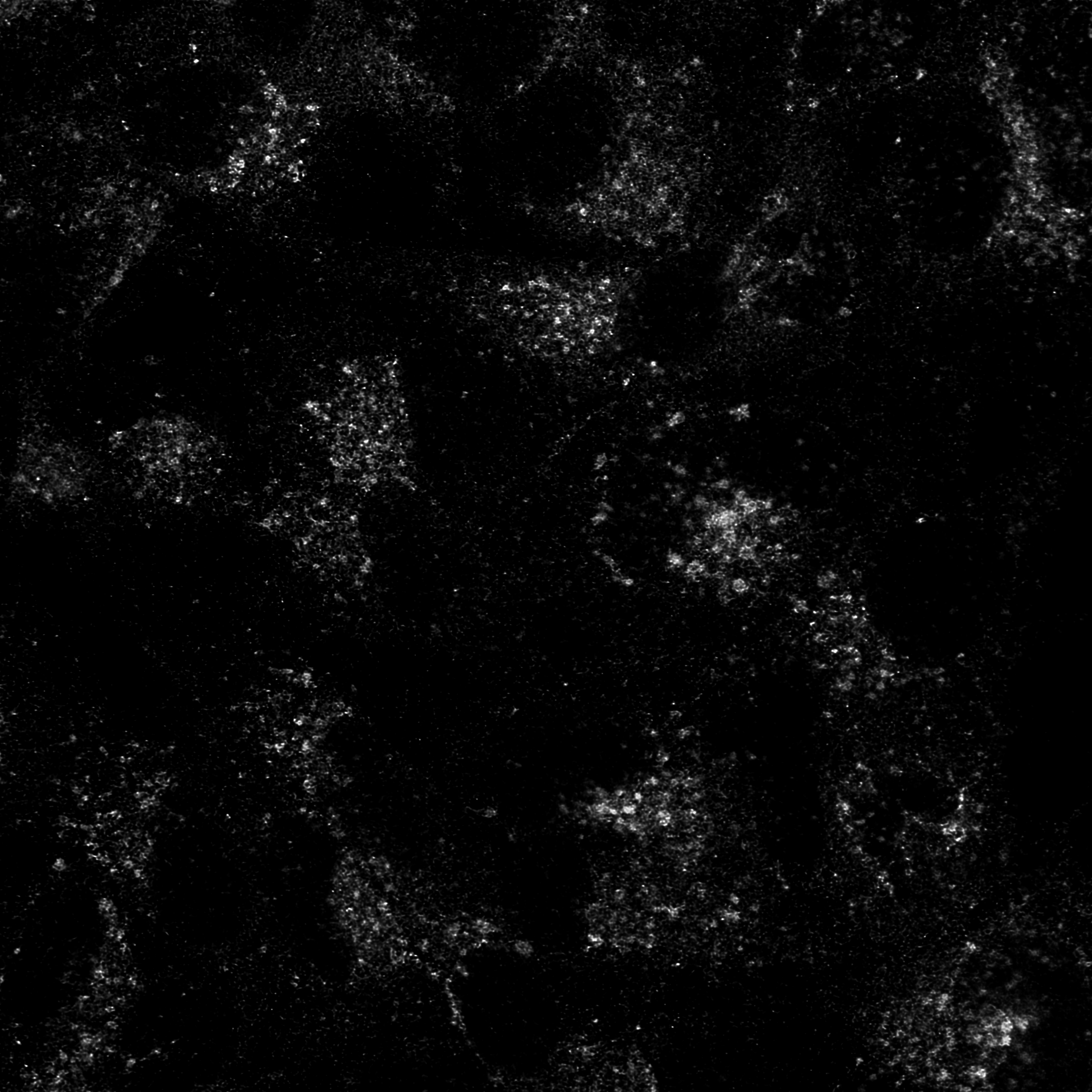

Supplement: Supplementary file 7 — Source data Fig. 2C-H [file 44319_2026_773_MOESM7_ESM.zip › Figure 2D/IF GRASP55KO LAMP2.tif]

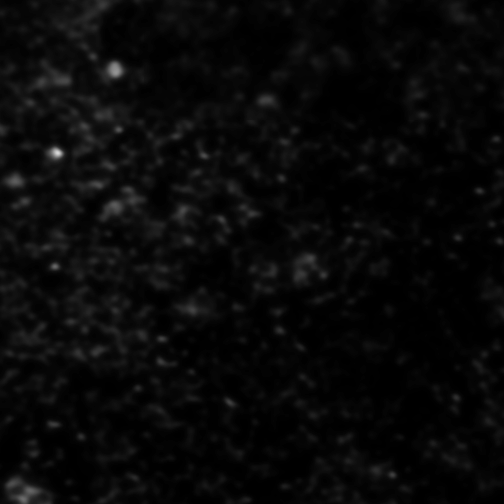

Supplement: Supplementary file 7 — Source data Fig. 2C-H [file 44319_2026_773_MOESM7_ESM.zip › Figure 2D/IF GNPTABKO GRN Inset.tif]

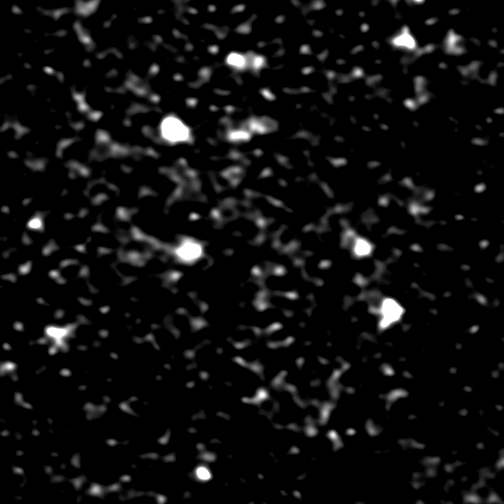

Supplement: Supplementary file 7 — Source data Fig. 2C-H [file 44319_2026_773_MOESM7_ESM.zip › Figure 2D/IF GRASP65KO GRN inset.tif]

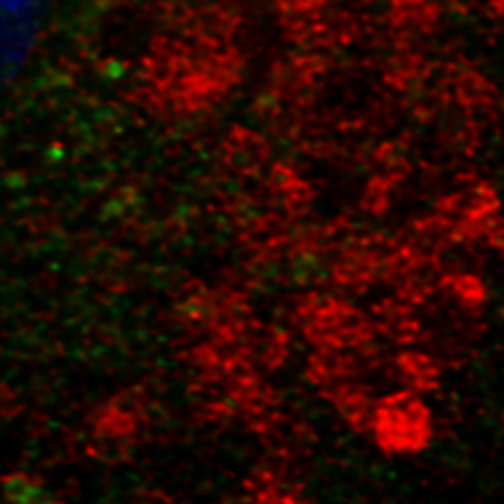

Supplement: Supplementary file 7 — Source data Fig. 2C-H [file 44319_2026_773_MOESM7_ESM.zip › Figure 2D/IF GNPTABKO GRN_LAMP2 MERGE Inset.tif]

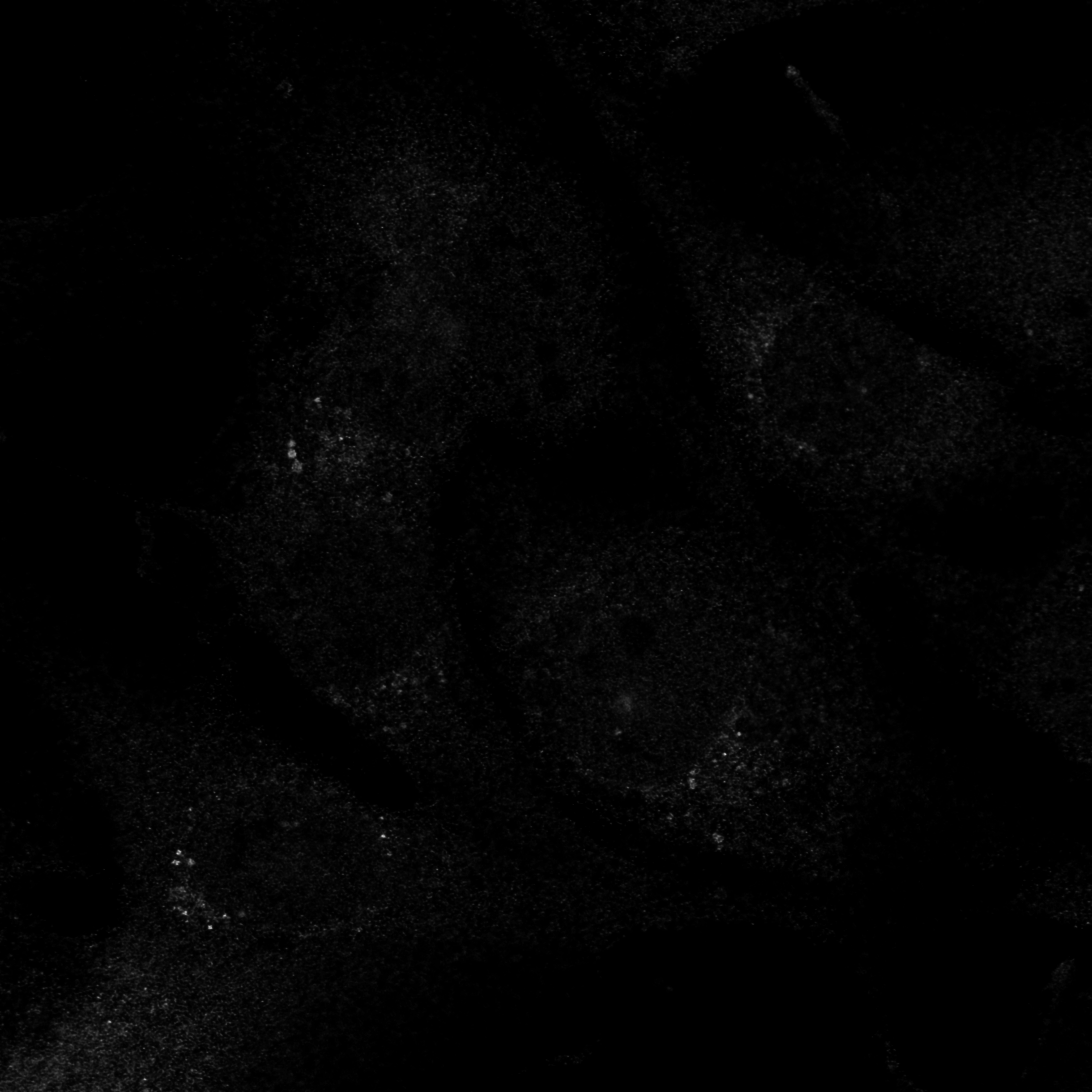

Supplement: Supplementary file 7 — Source data Fig. 2C-H [file 44319_2026_773_MOESM7_ESM.zip › Figure 2D/IF GNPTABKO GRN.tif]

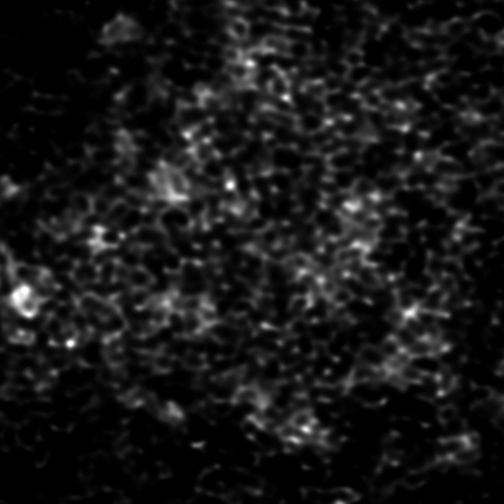

Supplement: Supplementary file 7 — Source data Fig. 2C-H [file 44319_2026_773_MOESM7_ESM.zip › Figure 2D/IF GRASP55KO LAMP2 inset.tif]

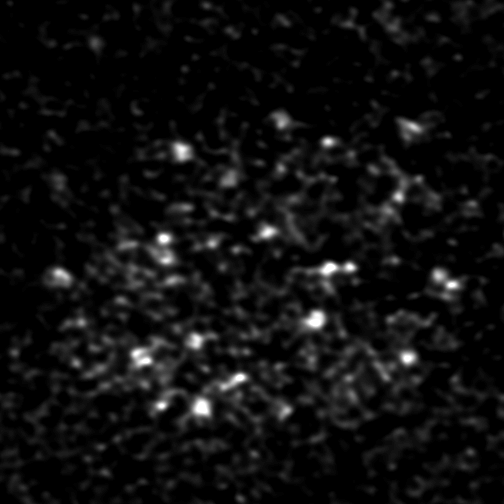

Supplement: Supplementary file 7 — Source data Fig. 2C-H [file 44319_2026_773_MOESM7_ESM.zip › Figure 2D/IF WT GRN Inset.tif]

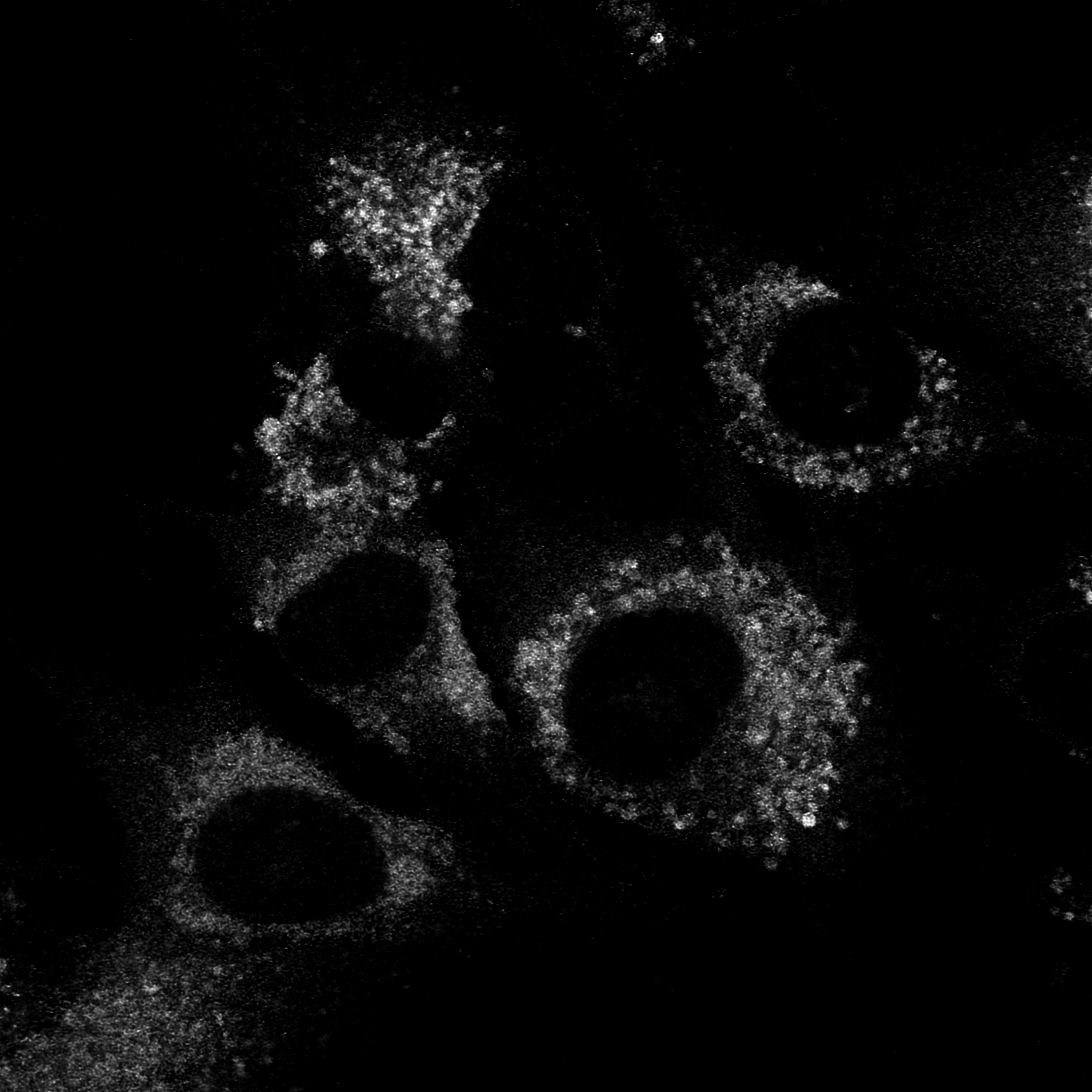

Supplement: Supplementary file 7 — Source data Fig. 2C-H [file 44319_2026_773_MOESM7_ESM.zip › Figure 2D/IF GNPTABKO LAMP2.tif]

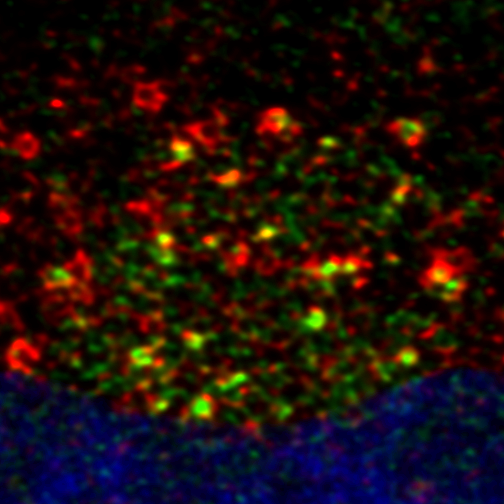

Supplement: Supplementary file 7 — Source data Fig. 2C-H [file 44319_2026_773_MOESM7_ESM.zip › Figure 2D/IF WT GRN_LAMP2 MERGE Inset.tif]

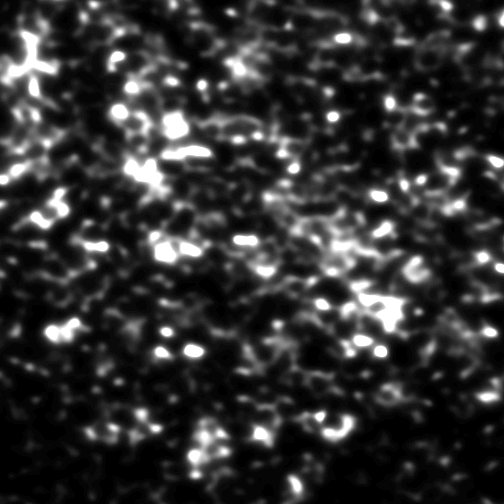

Supplement: Supplementary file 7 — Source data Fig. 2C-H [file 44319_2026_773_MOESM7_ESM.zip › Figure 2D/IF GRASP65KO LAMP2 inset.tif]

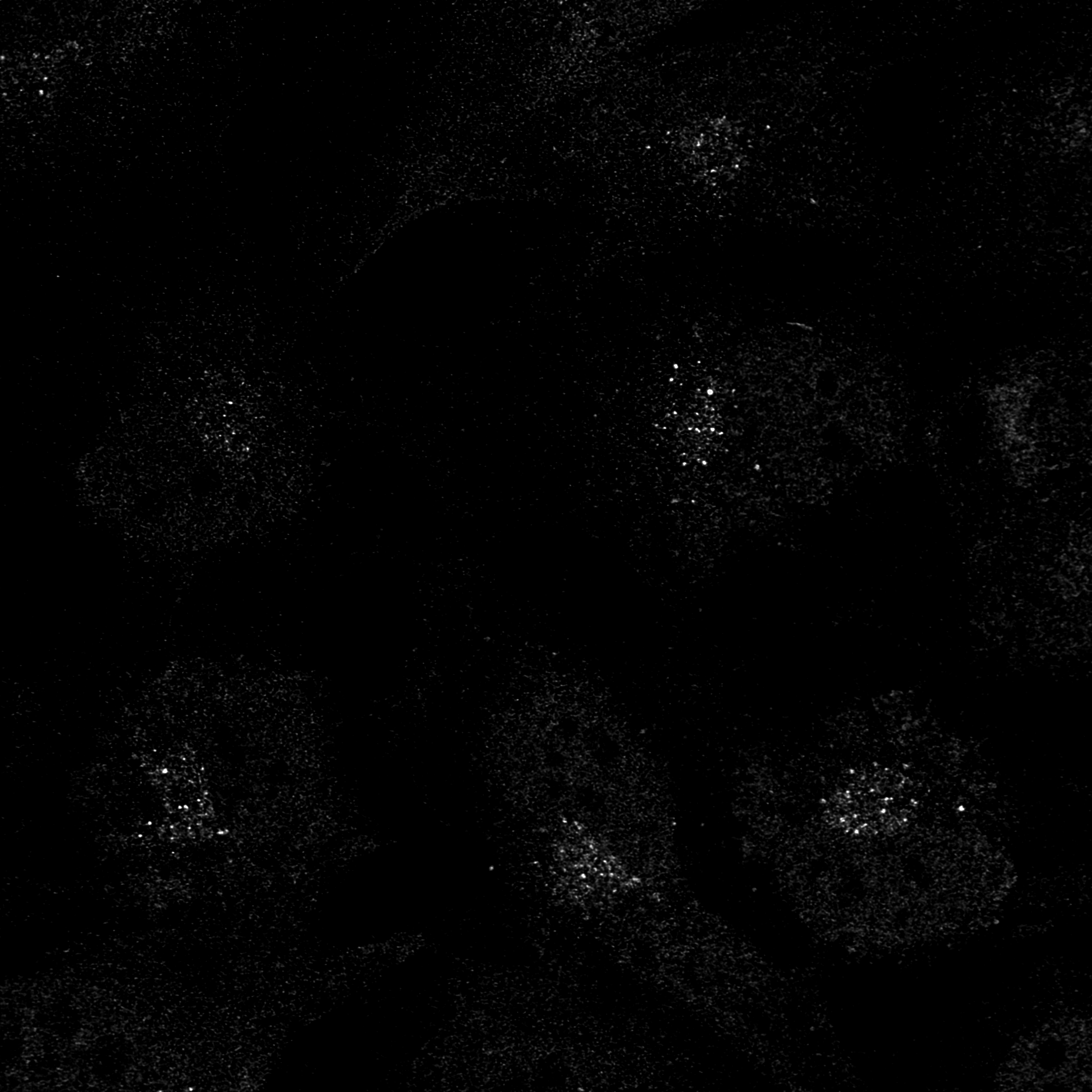

Supplement: Supplementary file 7 — Source data Fig. 2C-H [file 44319_2026_773_MOESM7_ESM.zip › Figure 2D/IF WT GRN.tif]

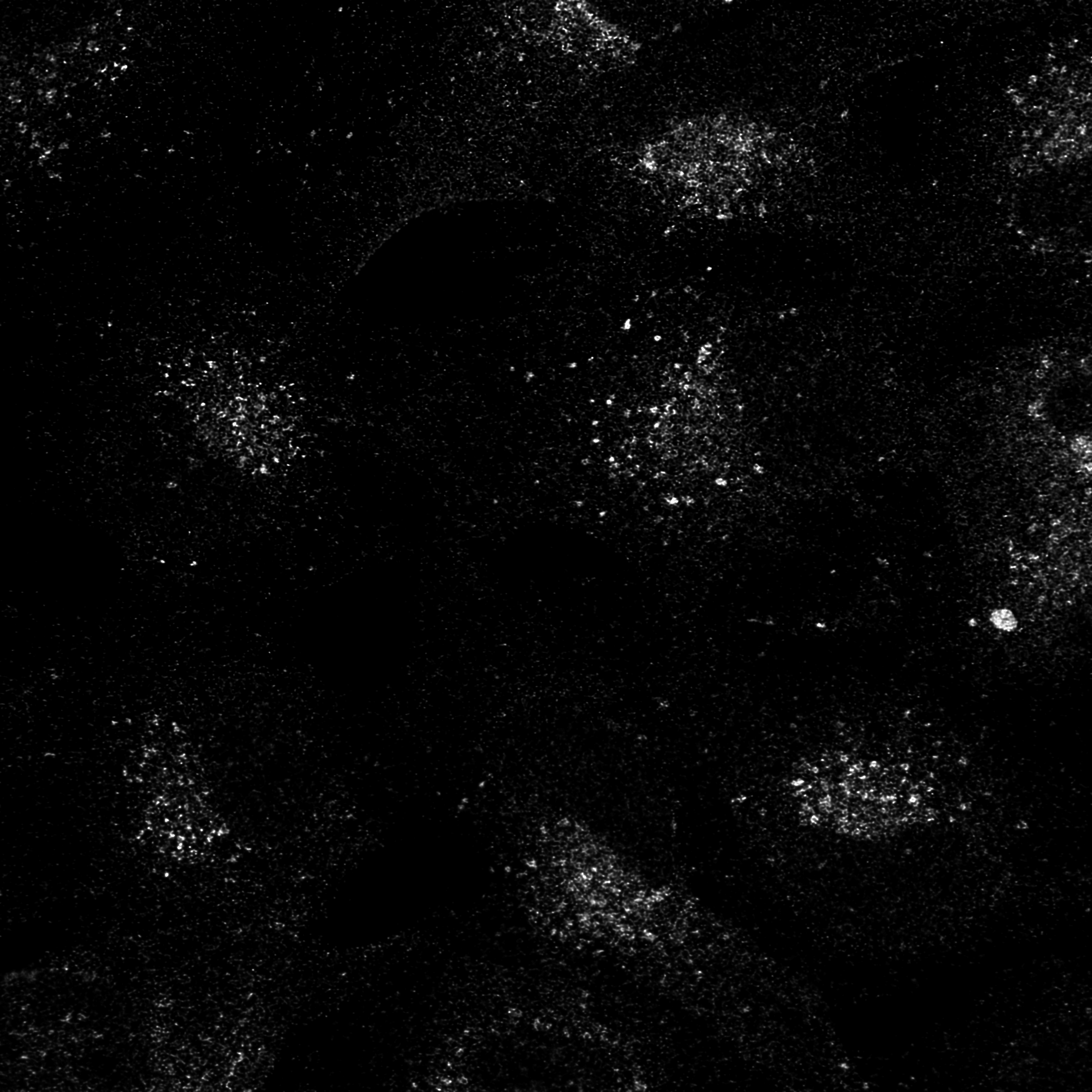

Supplement: Supplementary file 7 — Source data Fig. 2C-H [file 44319_2026_773_MOESM7_ESM.zip › Figure 2D/IF WT LAMP2.tif]

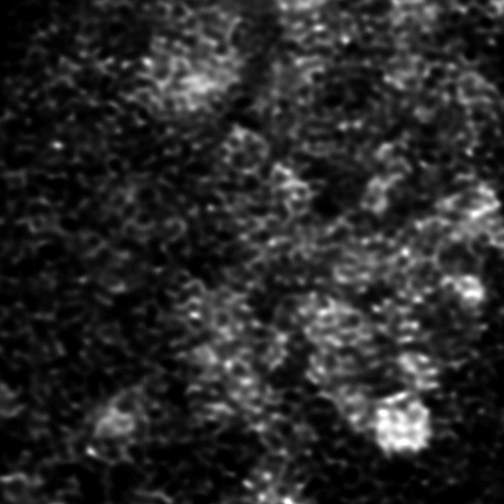

Supplement: Supplementary file 7 — Source data Fig. 2C-H [file 44319_2026_773_MOESM7_ESM.zip › Figure 2D/IF GNPTABKO LAMP2 Inset tif.tif]

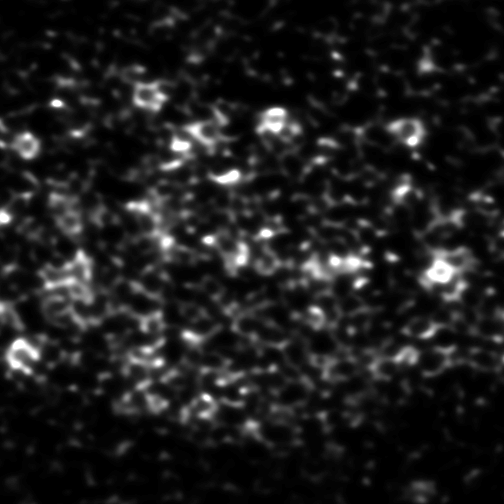

Supplement: Supplementary file 7 — Source data Fig. 2C-H [file 44319_2026_773_MOESM7_ESM.zip › Figure 2D/IF WT LAMP2 Inset.tif]

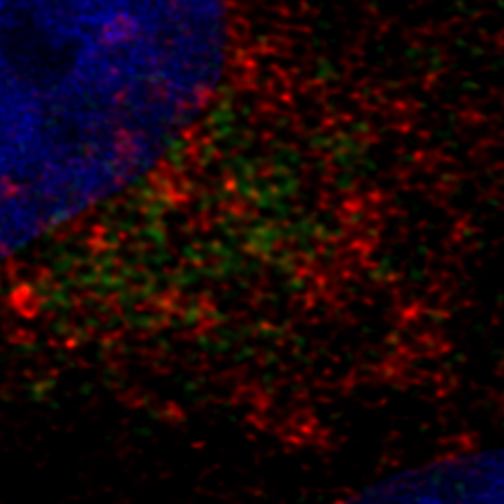

Supplement: Supplementary file 7 — Source data Fig. 2C-H [file 44319_2026_773_MOESM7_ESM.zip › Figure 2D/IF GRASP55KO GRN_LAMP2 MERGE Inset.tif]

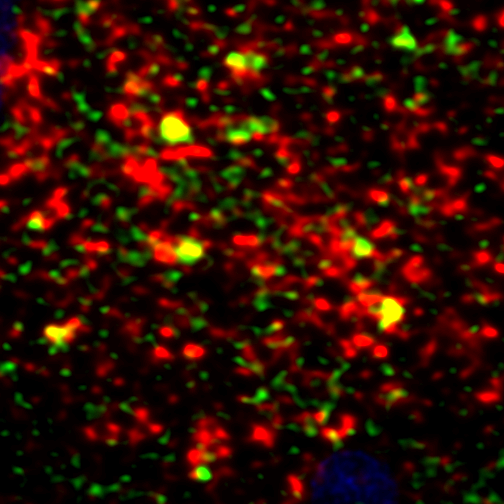

Supplement: Supplementary file 7 — Source data Fig. 2C-H [file 44319_2026_773_MOESM7_ESM.zip › Figure 2D/IF GRASP65KO GRN_LAMP2 MERGE inset.tif]
