# Supplementary figures and images for: GRASP55 maintains lysosome function by controlling sorting of lysosomal enzymes at the Golgi (part 2 of 5)
Source: EMBO Rep. 2026 Apr 16;27(11):2947–72. doi: 10.1038/s44319-026-00773-w (PMC13261057; doi:10.1038/s44319-026-00773-w)

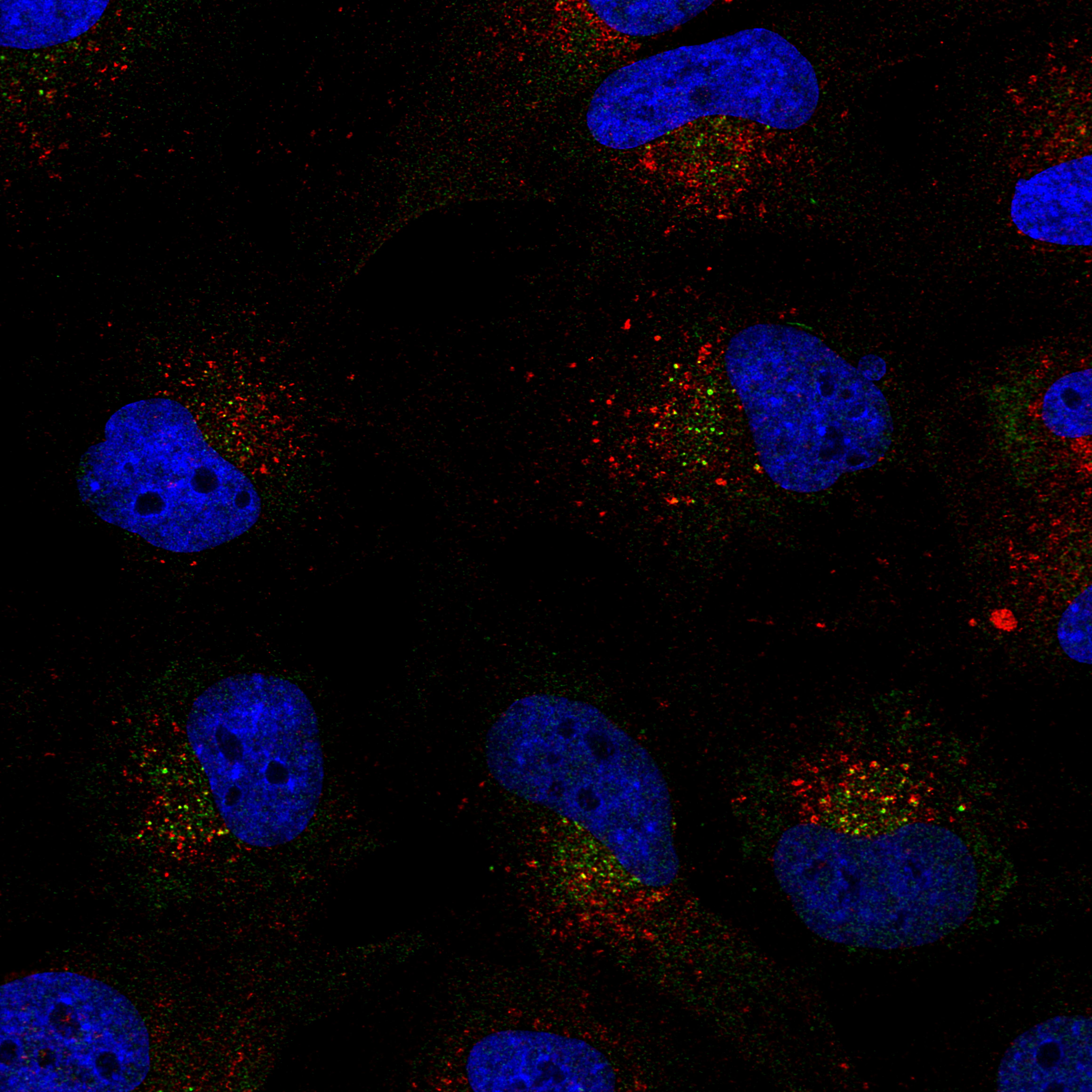

Supplement: Supplementary file 7 — Source data Fig. 2C-H [file 44319_2026_773_MOESM7_ESM.zip › Figure 2D/IF WT GRN_LAMP2 MERGE.tif]

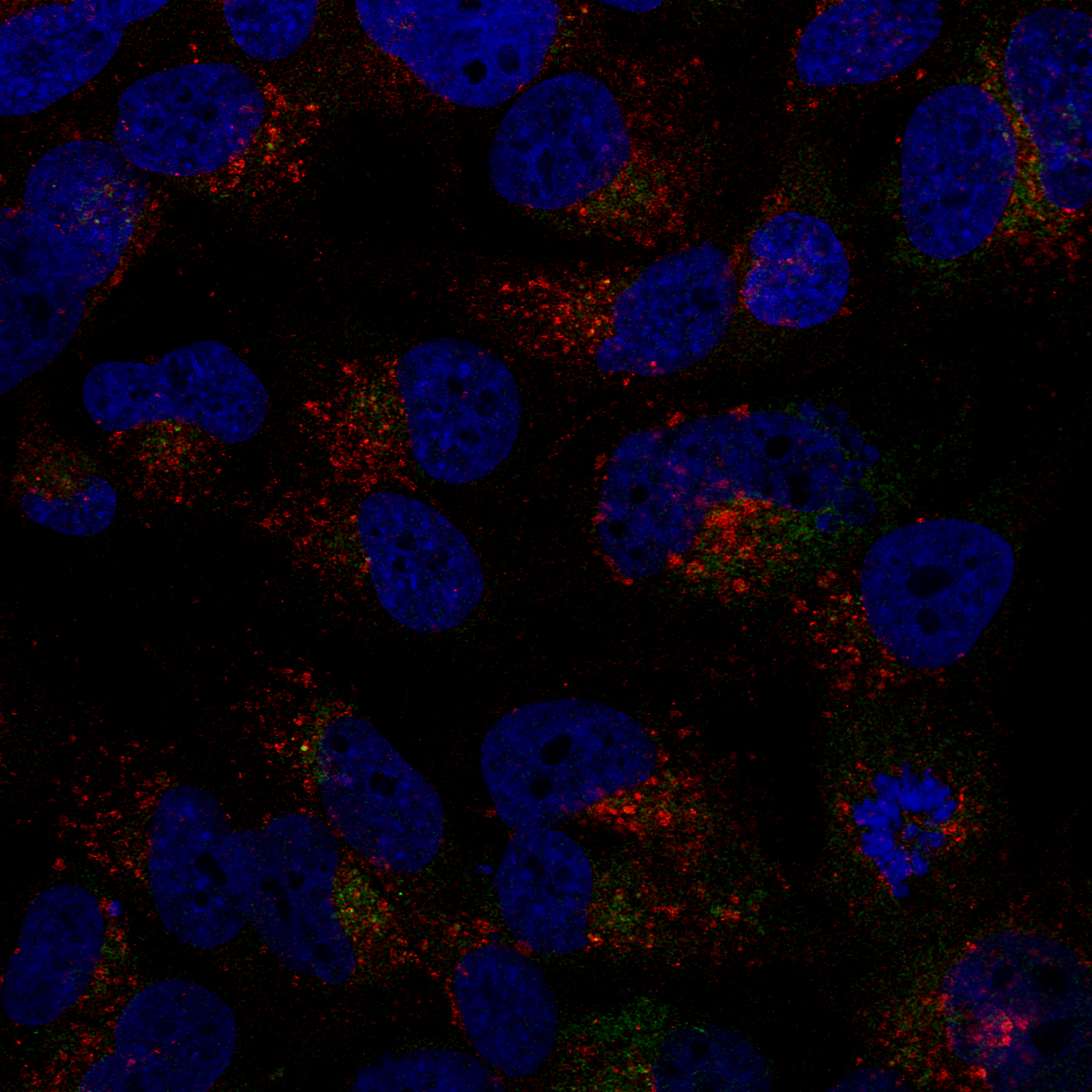

Supplement: Supplementary file 7 — Source data Fig. 2C-H [file 44319_2026_773_MOESM7_ESM.zip › Figure 2D/IF GRASP55KO GRN_LAMP2 MERGE.tif]

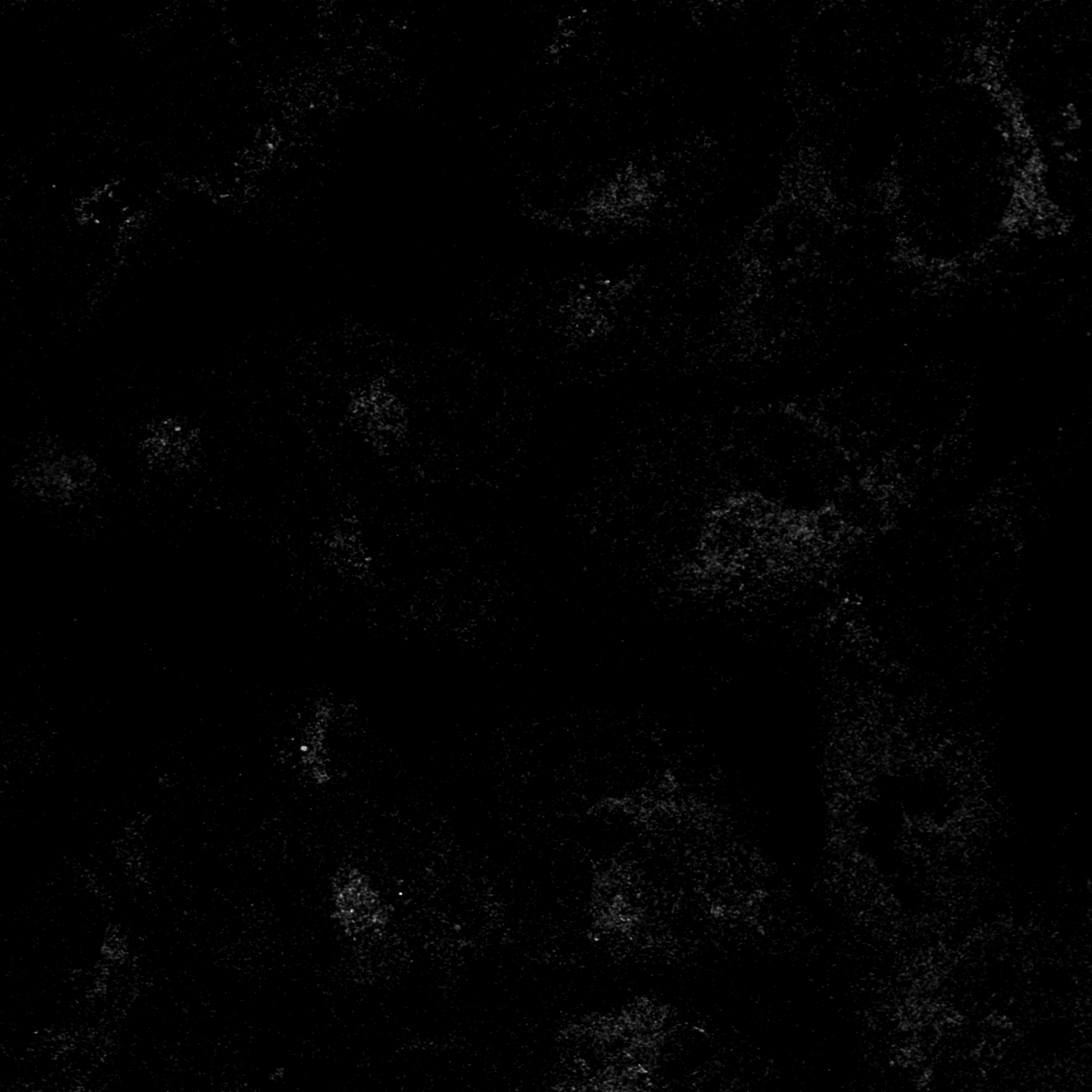

Supplement: Supplementary file 7 — Source data Fig. 2C-H [file 44319_2026_773_MOESM7_ESM.zip › Figure 2D/IF GRASP55KO GRN.tif]

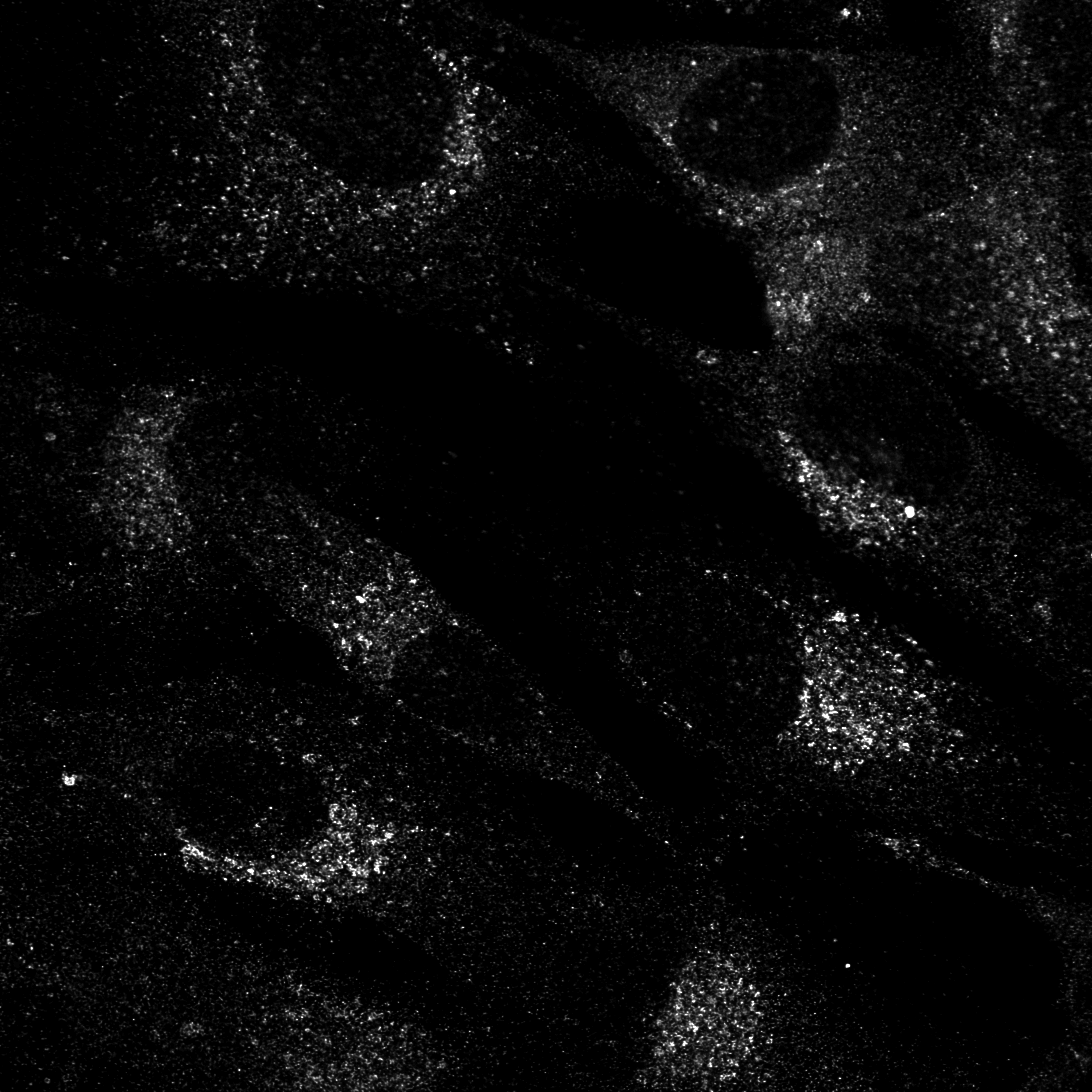

Supplement: Supplementary file 7 — Source data Fig. 2C-H [file 44319_2026_773_MOESM7_ESM.zip › Figure 2D/IF GRASP65KO LAMP2.tif]

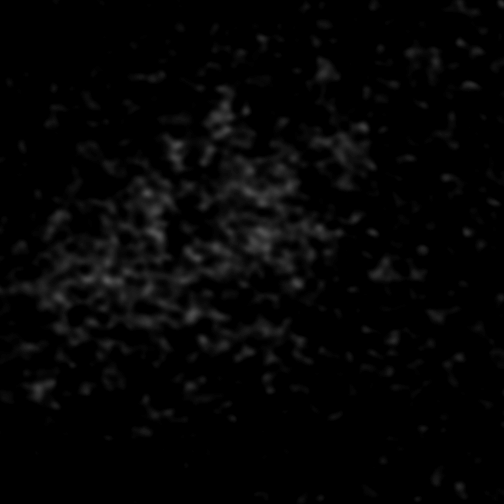

Supplement: Supplementary file 7 — Source data Fig. 2C-H [file 44319_2026_773_MOESM7_ESM.zip › Figure 2D/IF GRASP55KO GRN inset.tif]

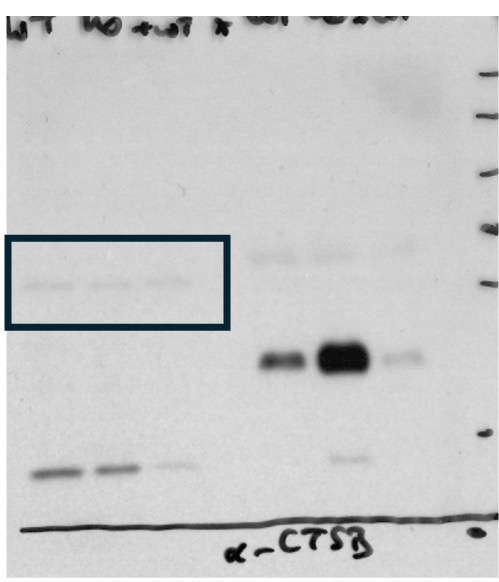

Supplement: Supplementary file 9 — Source data Fig. 4 [file 44319_2026_773_MOESM9_ESM.zip › Figure 4/Figure 4E/Western CTSB lysate.tif]

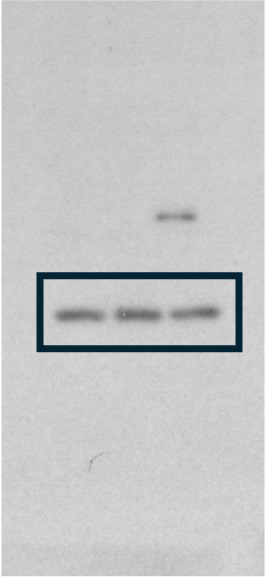

Supplement: Supplementary file 9 — Source data Fig. 4 [file 44319_2026_773_MOESM9_ESM.zip › Figure 4/Figure 4E/Western ACTIN.tif]

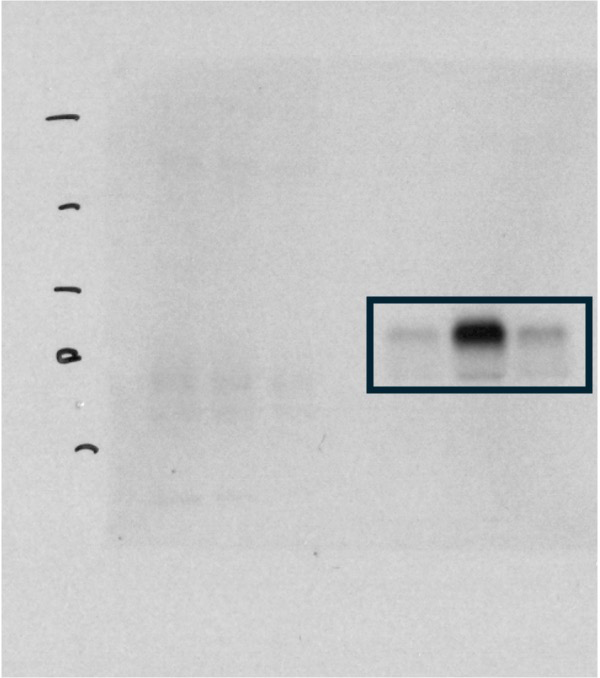

Supplement: Supplementary file 9 — Source data Fig. 4 [file 44319_2026_773_MOESM9_ESM.zip › Figure 4/Figure 4E/Western PSAP culture medium.tif]

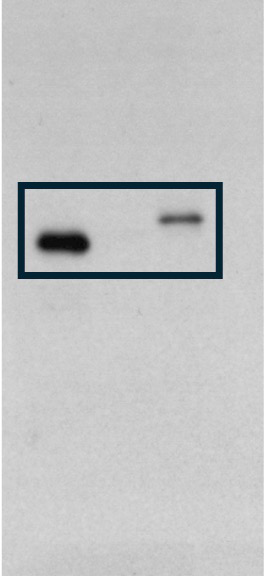

Supplement: Supplementary file 9 — Source data Fig. 4 [file 44319_2026_773_MOESM9_ESM.zip › Figure 4/Figure 4E/Western GRASP55.tif]

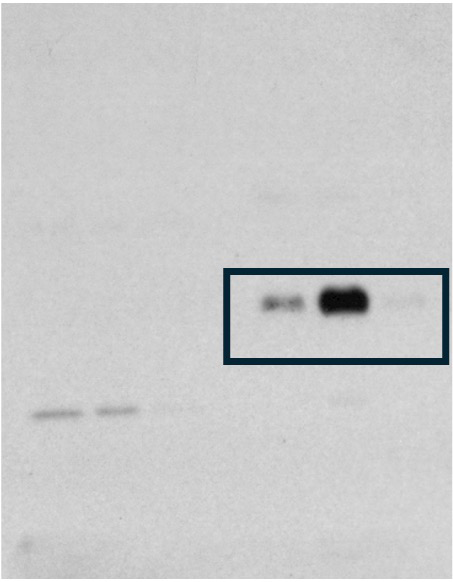

Supplement: Supplementary file 9 — Source data Fig. 4 [file 44319_2026_773_MOESM9_ESM.zip › Figure 4/Figure 4E/Western CTSB culture medium.tif]

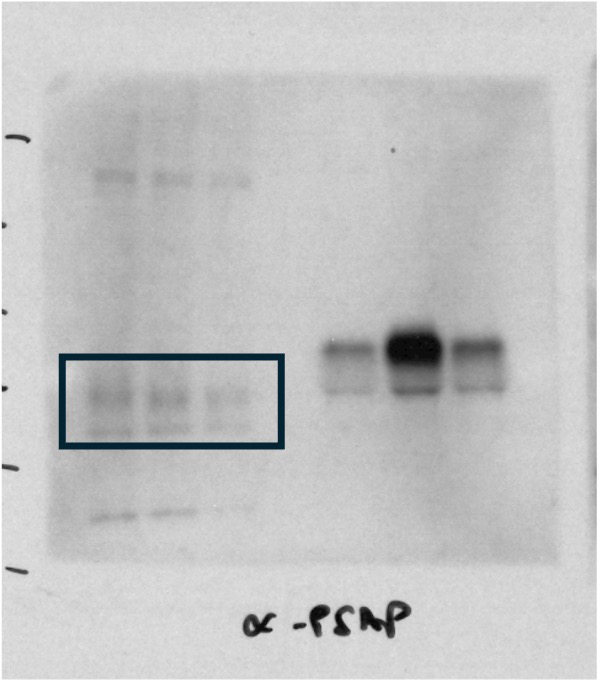

Supplement: Supplementary file 9 — Source data Fig. 4 [file 44319_2026_773_MOESM9_ESM.zip › Figure 4/Figure 4E/Western PSAP lysate.tif]

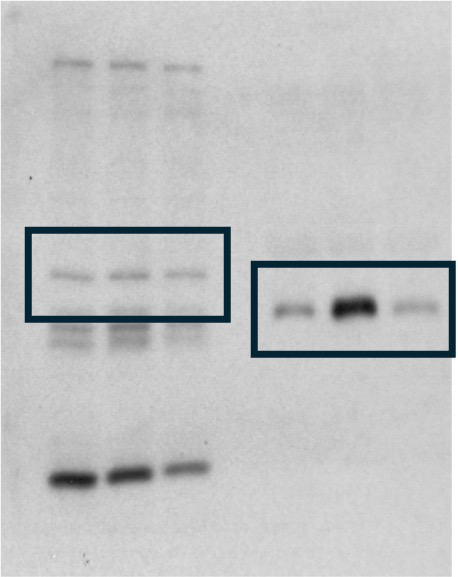

Supplement: Supplementary file 9 — Source data Fig. 4 [file 44319_2026_773_MOESM9_ESM.zip › Figure 4/Figure 4E/Western CTSD.tif]

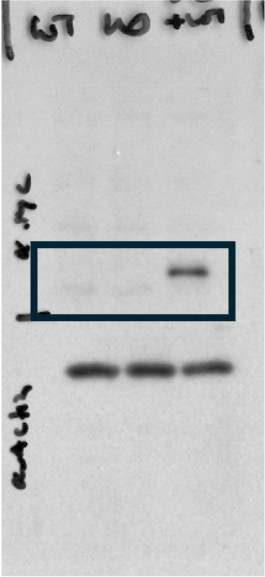

Supplement: Supplementary file 9 — Source data Fig. 4 [file 44319_2026_773_MOESM9_ESM.zip › Figure 4/Figure 4E/Western MYC.tif]

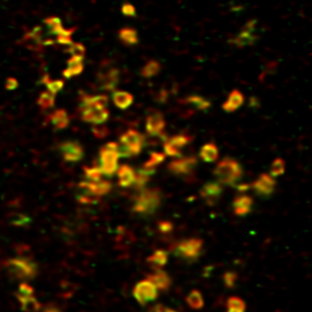

Supplement: Supplementary file 9 — Source data Fig. 4 [file 44319_2026_773_MOESM9_ESM.zip › Figure 4/Figure 4C/IF WT LIMP2 LAMP2 MERGE inset.tif]

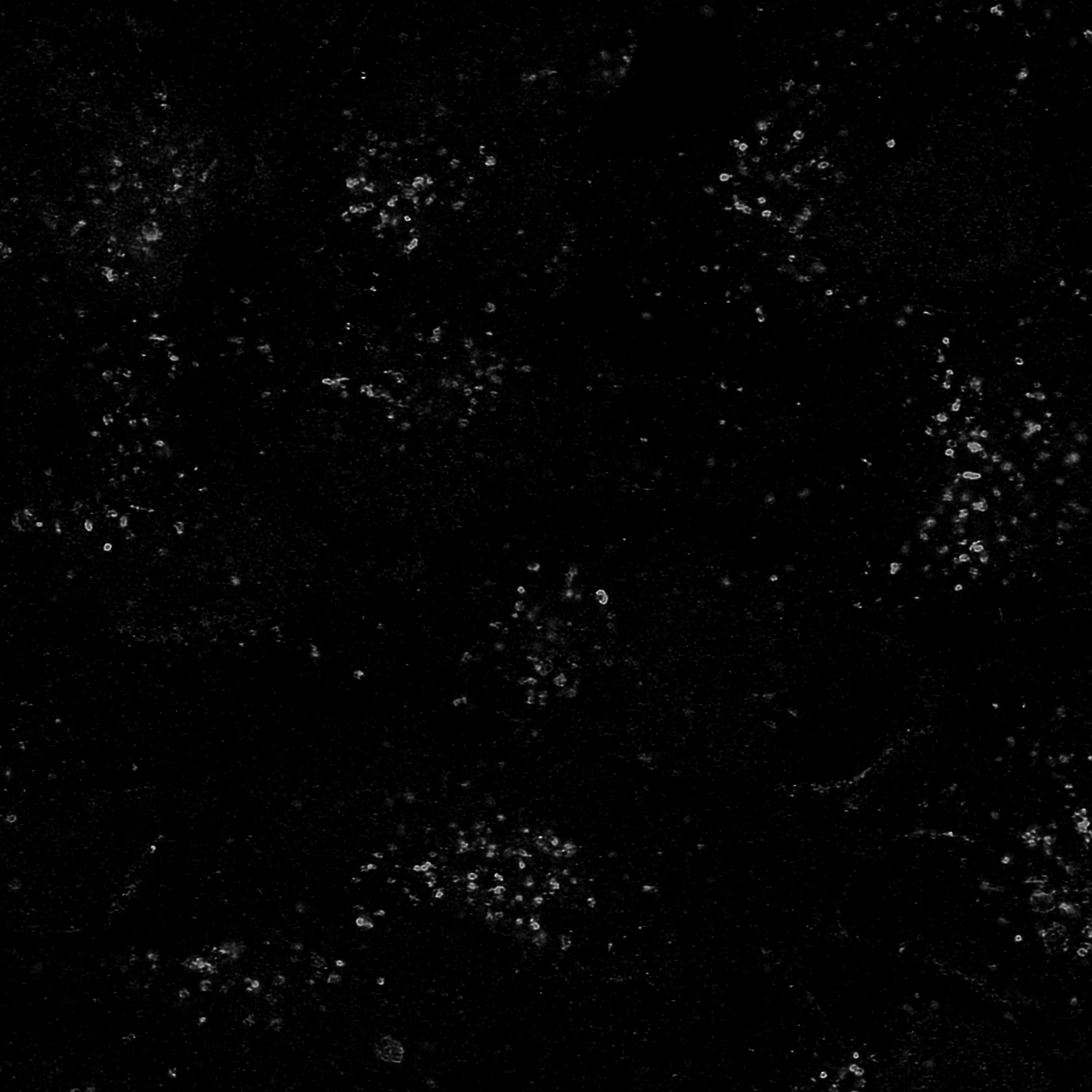

Supplement: Supplementary file 9 — Source data Fig. 4 [file 44319_2026_773_MOESM9_ESM.zip › Figure 4/Figure 4C/IF GRASP55KO+WT LIMP2.tif]

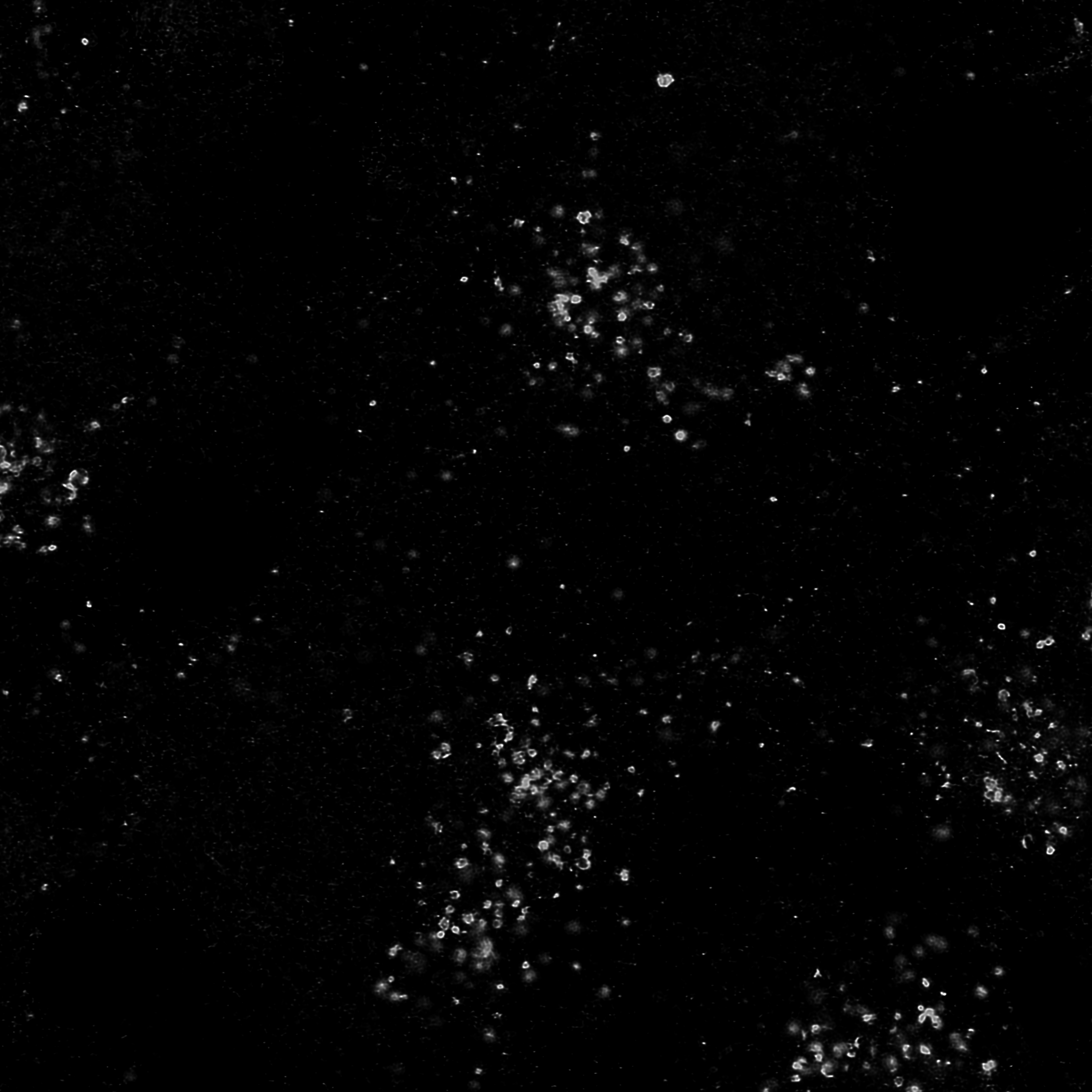

Supplement: Supplementary file 9 — Source data Fig. 4 [file 44319_2026_773_MOESM9_ESM.zip › Figure 4/Figure 4C/IF WT LIMP2.tif]

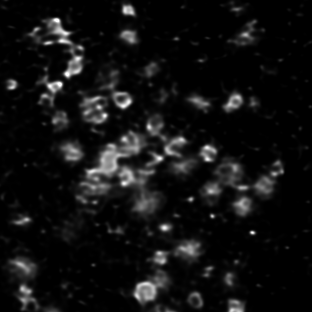

Supplement: Supplementary file 9 — Source data Fig. 4 [file 44319_2026_773_MOESM9_ESM.zip › Figure 4/Figure 4C/IF WT LIMP2 inset.tif]

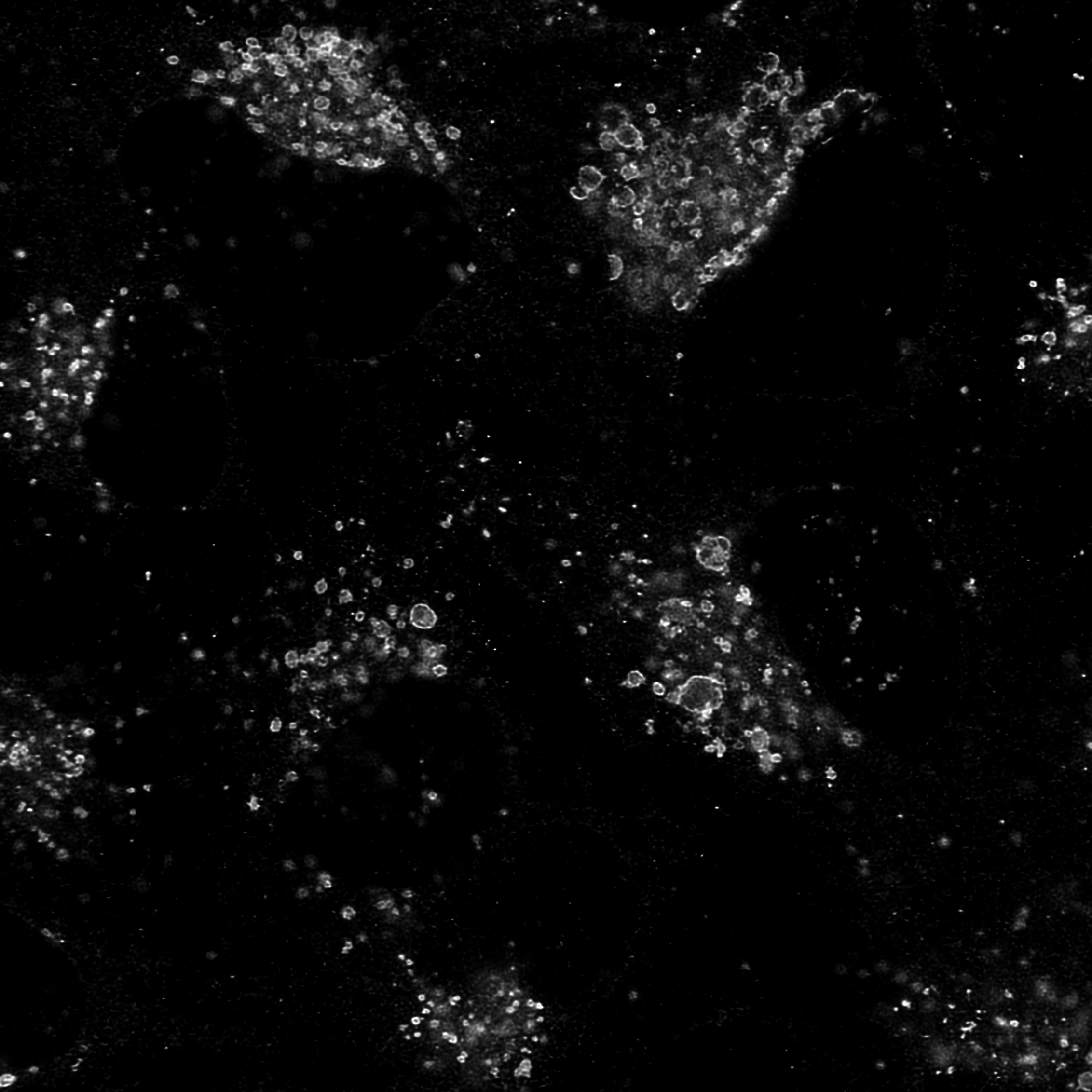

Supplement: Supplementary file 9 — Source data Fig. 4 [file 44319_2026_773_MOESM9_ESM.zip › Figure 4/Figure 4C/IF GRASP55KO LAMP2.tif]

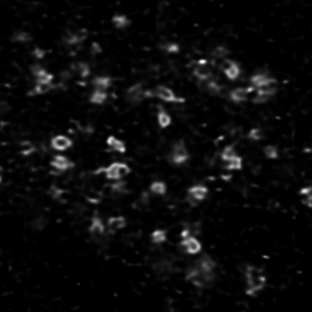

Supplement: Supplementary file 9 — Source data Fig. 4 [file 44319_2026_773_MOESM9_ESM.zip › Figure 4/Figure 4C/IF GRASP55KO+WT LIMP2 inset.tif]

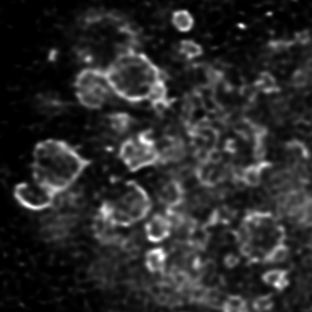

Supplement: Supplementary file 9 — Source data Fig. 4 [file 44319_2026_773_MOESM9_ESM.zip › Figure 4/Figure 4C/IF GRASP55KO LAMP2 inset.tif]

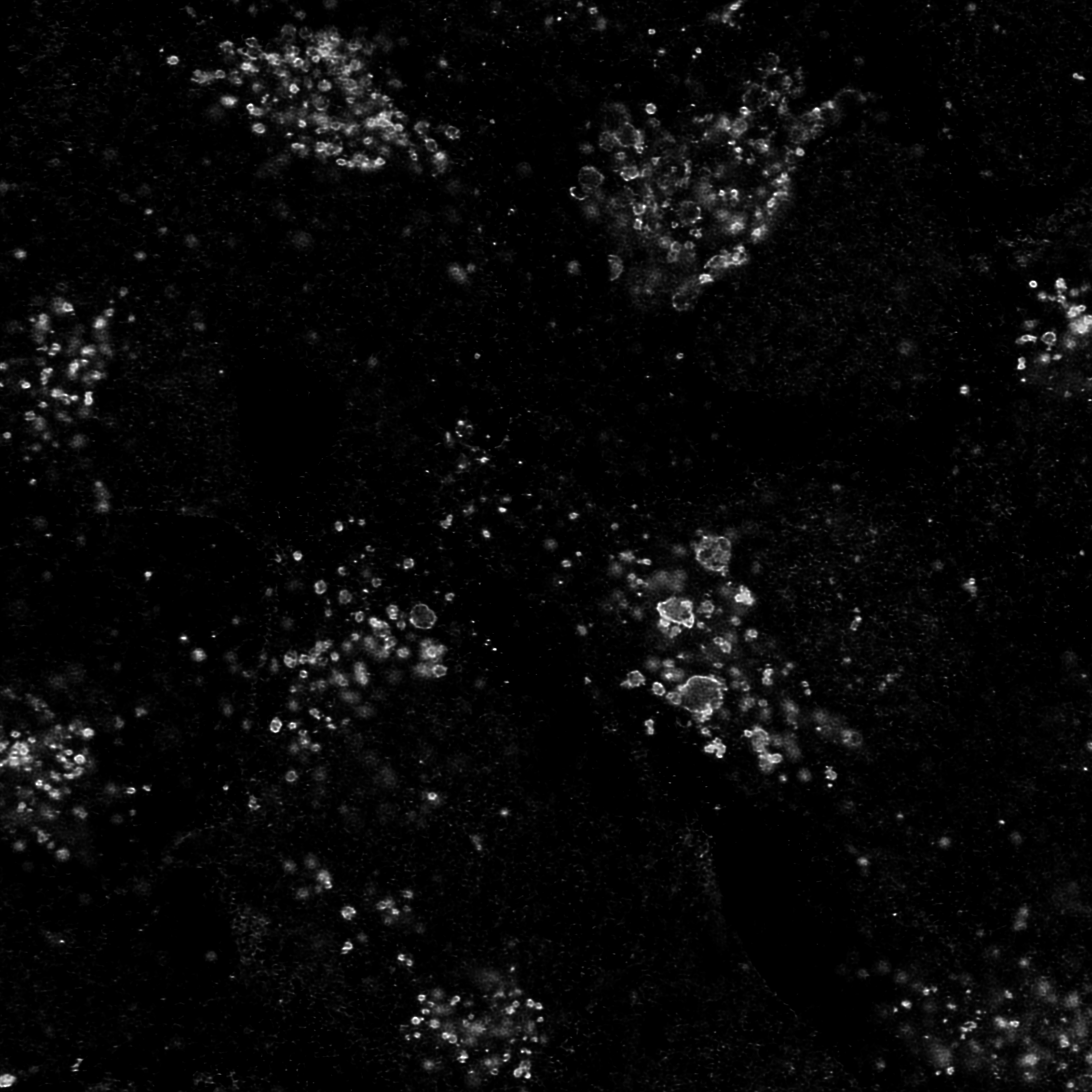

Supplement: Supplementary file 9 — Source data Fig. 4 [file 44319_2026_773_MOESM9_ESM.zip › Figure 4/Figure 4C/IF GRASP55KO LIMP2.tif]

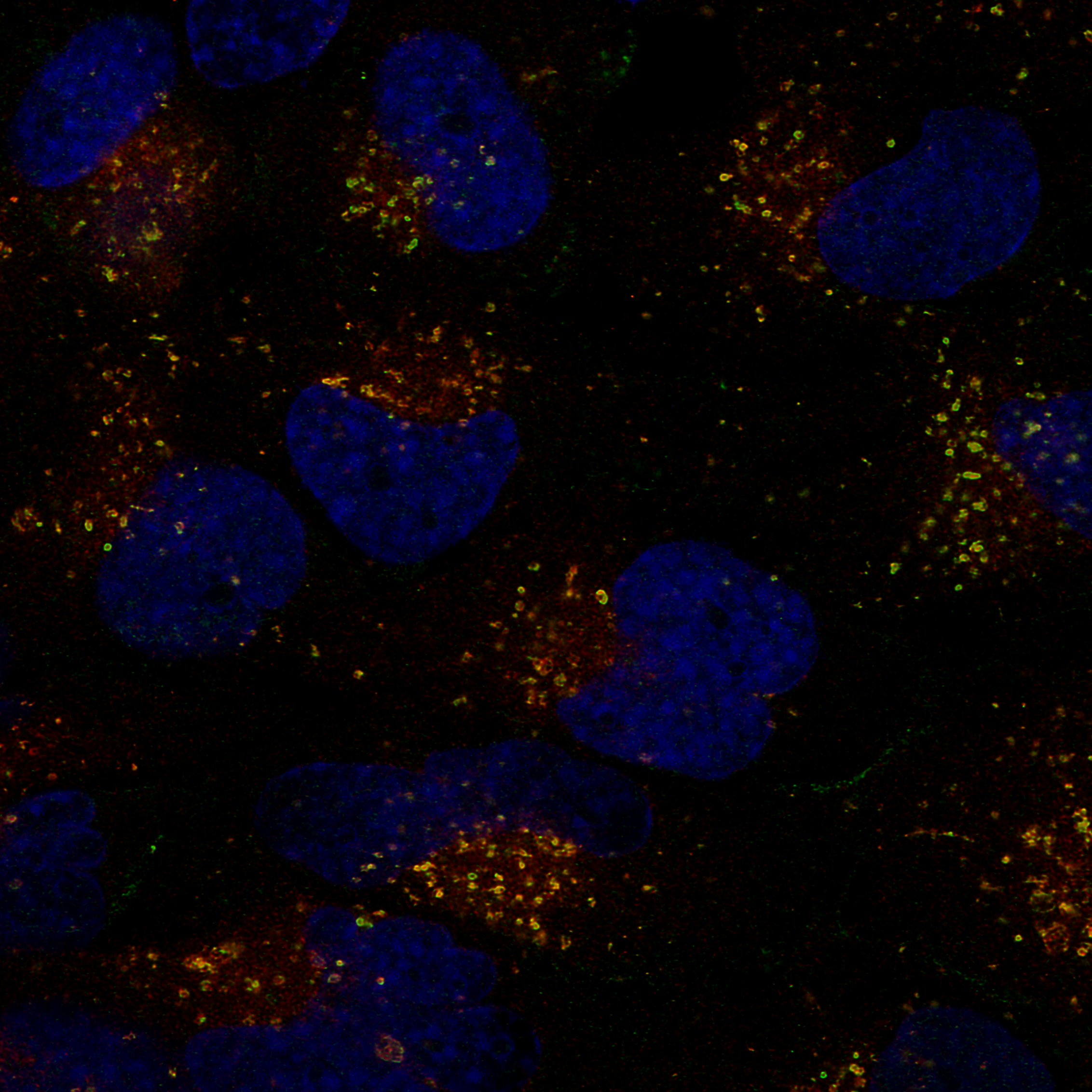

Supplement: Supplementary file 9 — Source data Fig. 4 [file 44319_2026_773_MOESM9_ESM.zip › Figure 4/Figure 4C/IF GRASP55KO+WT LIMP2 LAMP2 MERGE .tif]

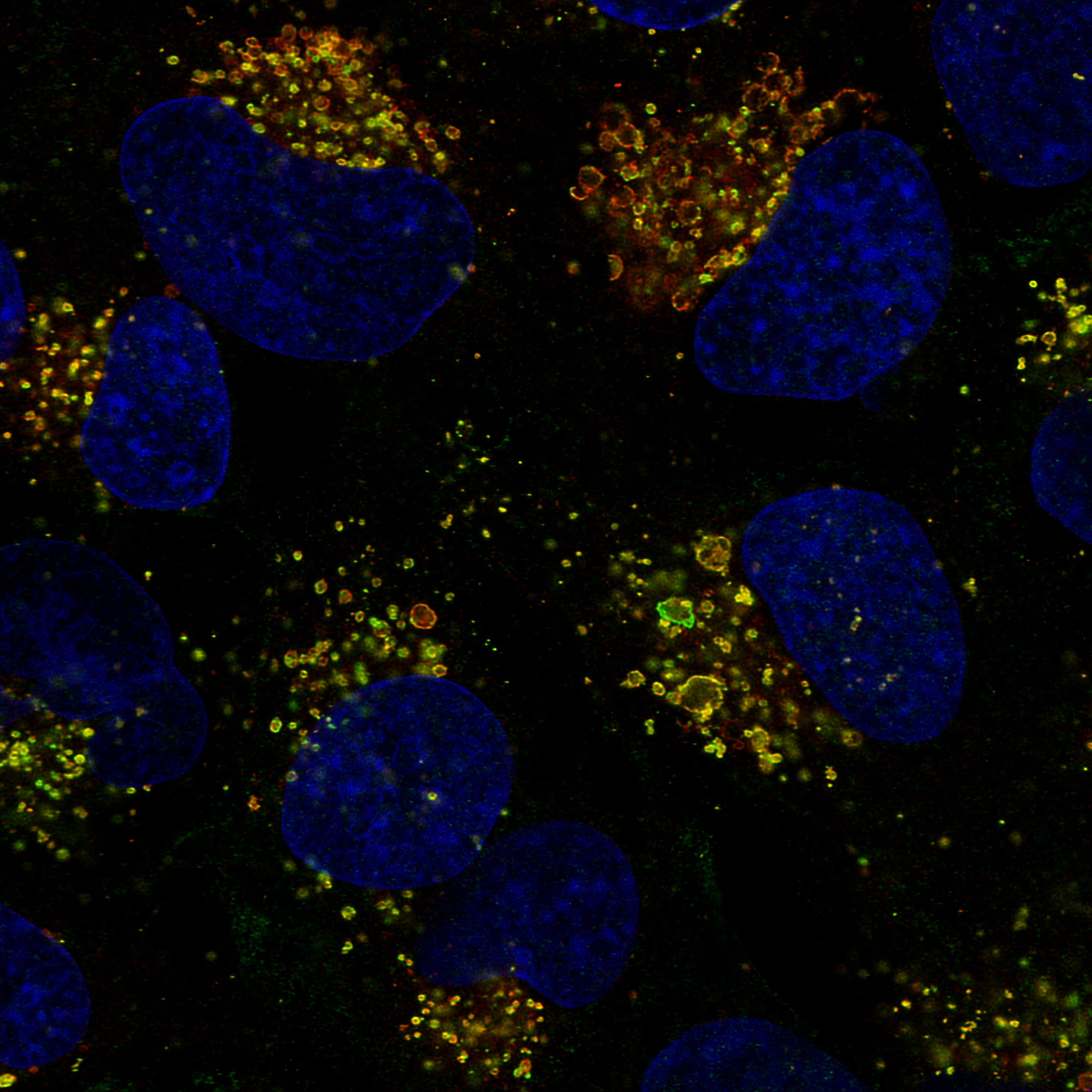

Supplement: Supplementary file 9 — Source data Fig. 4 [file 44319_2026_773_MOESM9_ESM.zip › Figure 4/Figure 4C/IF GRASP55KO LIMP2 LAMP2 MERGE.tif]

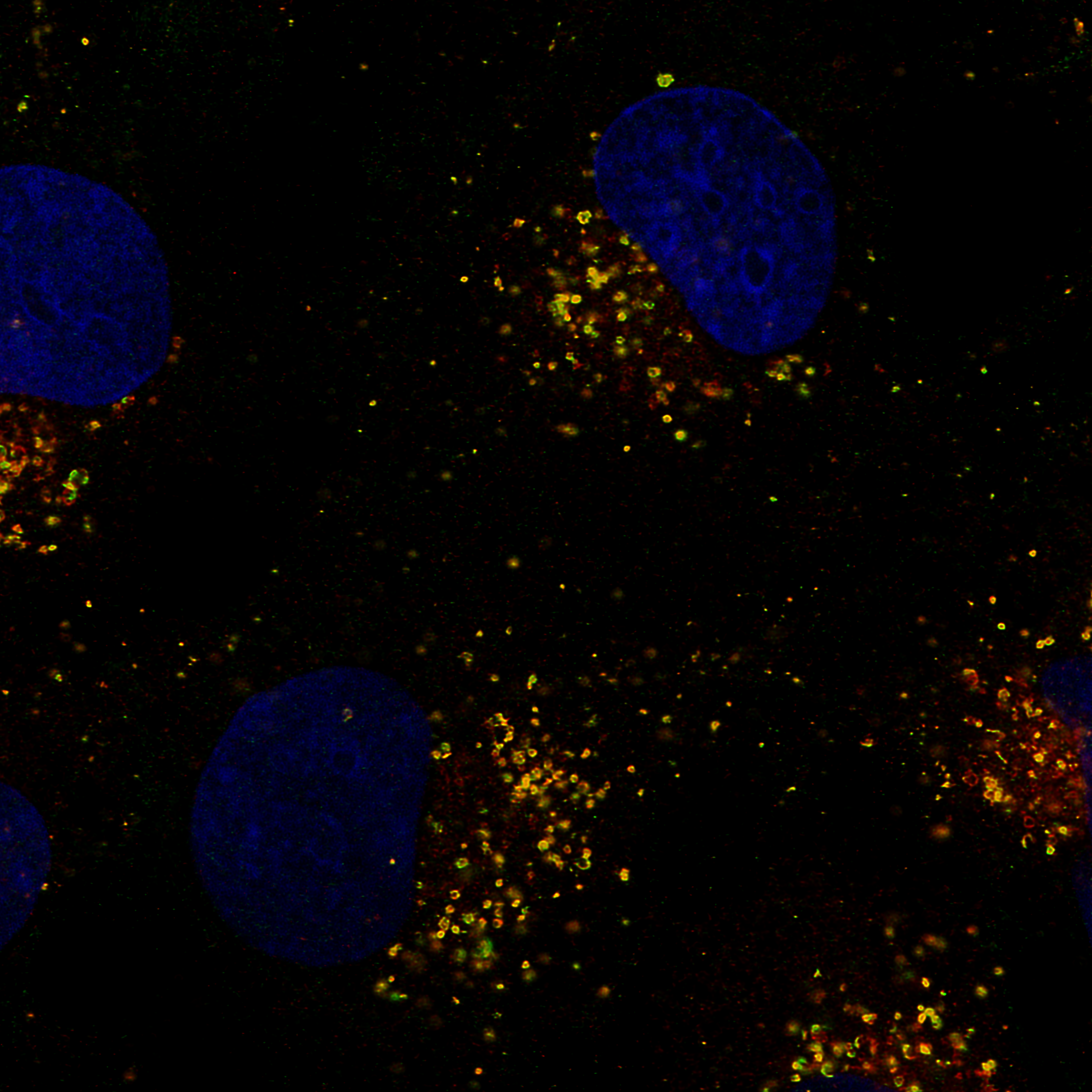

Supplement: Supplementary file 9 — Source data Fig. 4 [file 44319_2026_773_MOESM9_ESM.zip › Figure 4/Figure 4C/IF WT LIMP2 LAMP2 MERGE.tif]

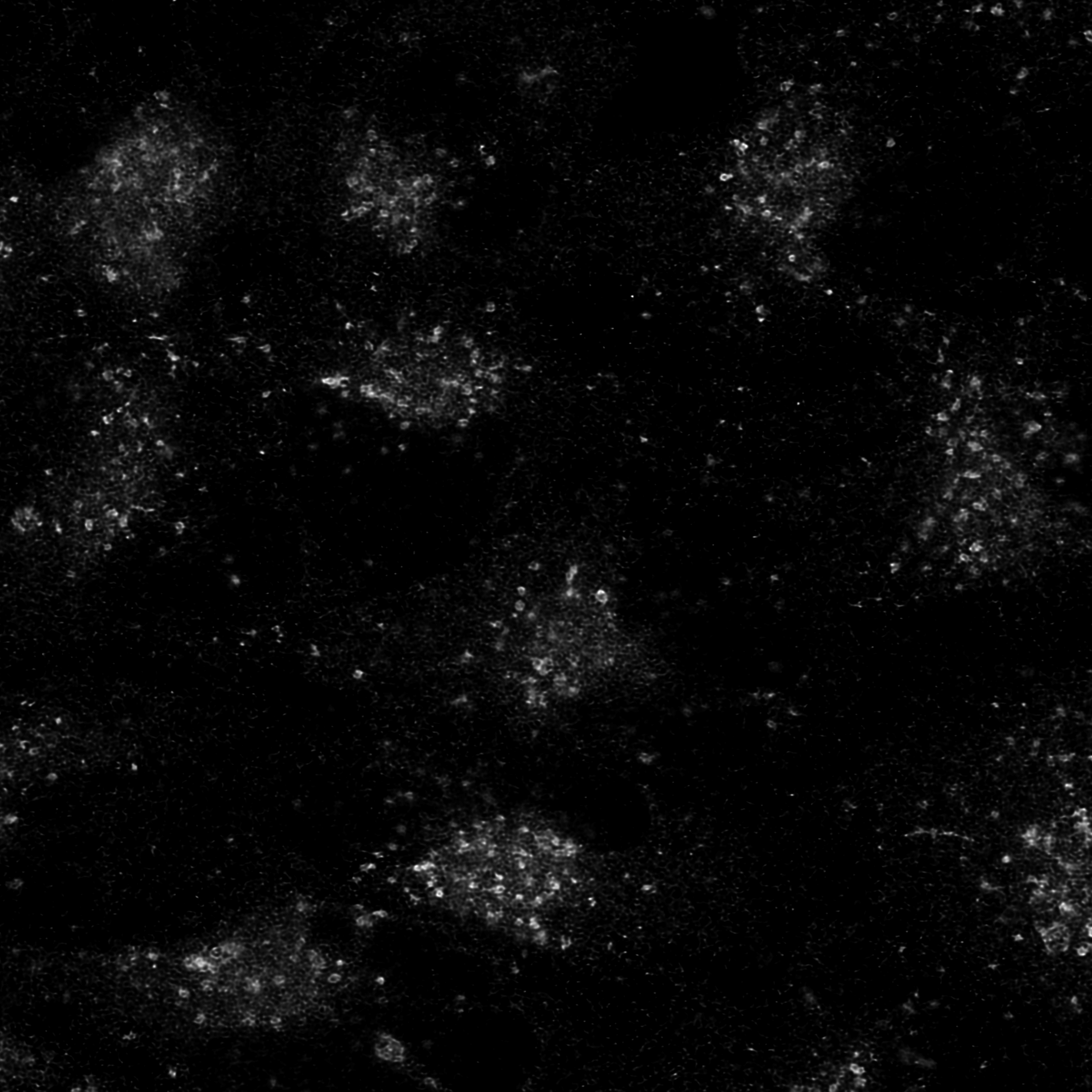

Supplement: Supplementary file 9 — Source data Fig. 4 [file 44319_2026_773_MOESM9_ESM.zip › Figure 4/Figure 4C/IF GRASP55KO+WT LAMP2.tif]

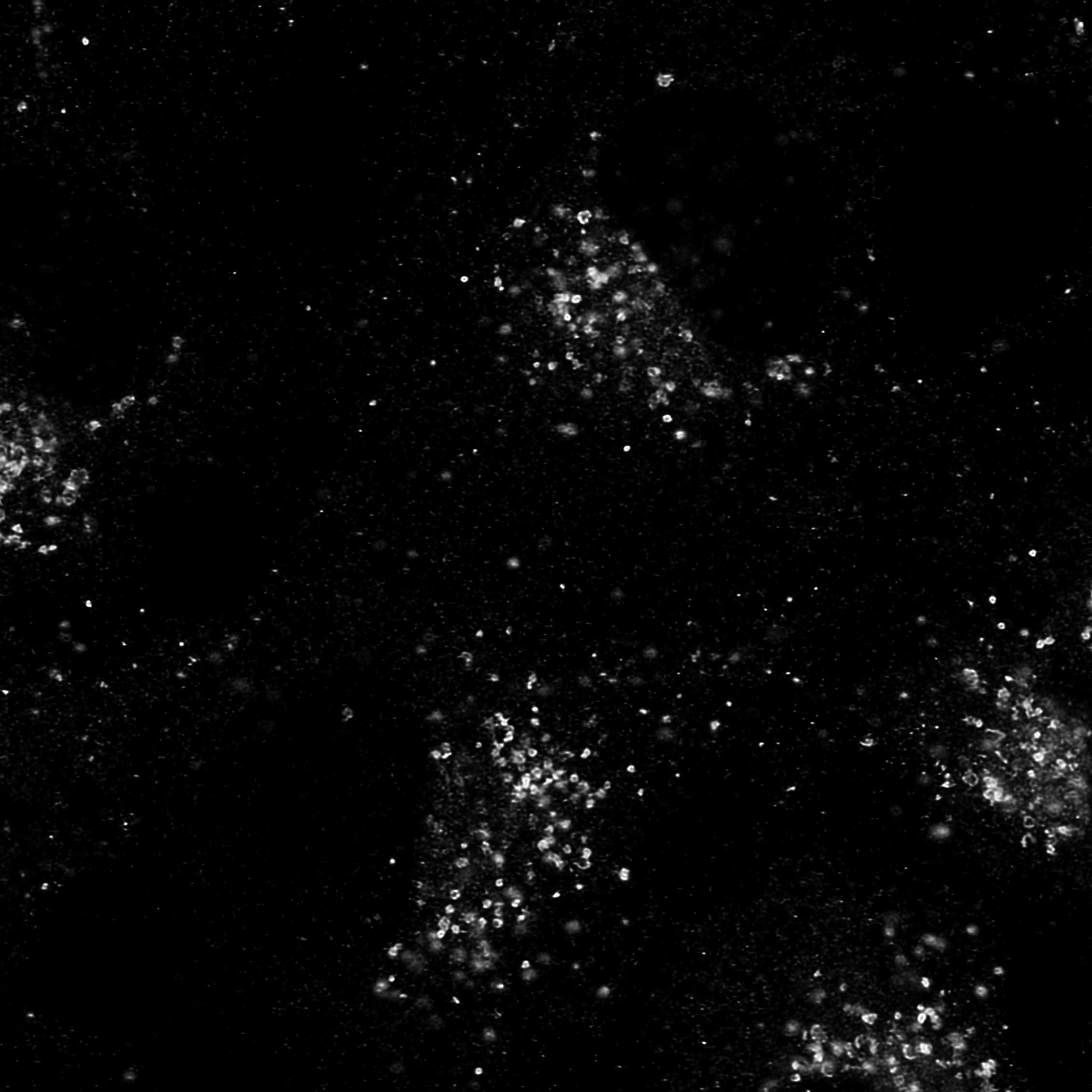

Supplement: Supplementary file 9 — Source data Fig. 4 [file 44319_2026_773_MOESM9_ESM.zip › Figure 4/Figure 4C/IF WT LAMP2.tif]

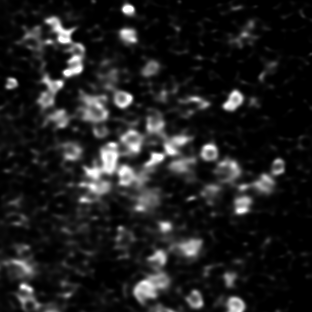

Supplement: Supplementary file 9 — Source data Fig. 4 [file 44319_2026_773_MOESM9_ESM.zip › Figure 4/Figure 4C/IF WT LAMP2 inset.tif]

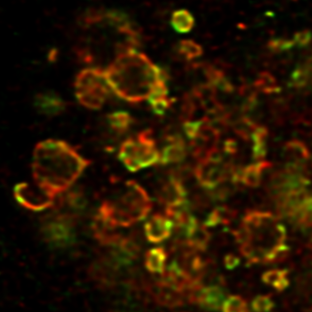

Supplement: Supplementary file 9 — Source data Fig. 4 [file 44319_2026_773_MOESM9_ESM.zip › Figure 4/Figure 4C/IF GRASP55KO LIMP2 LAMP2 MERGE inset.tif]

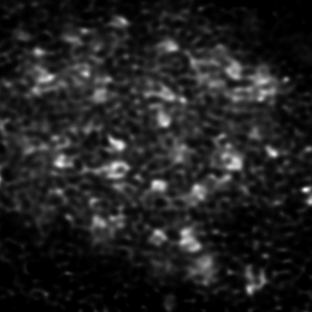

Supplement: Supplementary file 9 — Source data Fig. 4 [file 44319_2026_773_MOESM9_ESM.zip › Figure 4/Figure 4C/IF GRASP55KO+WT LAMP2 inset.tif]

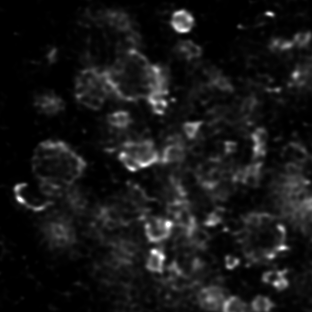

Supplement: Supplementary file 9 — Source data Fig. 4 [file 44319_2026_773_MOESM9_ESM.zip › Figure 4/Figure 4C/IF GRASP55KO LIMP2 inset.tif]

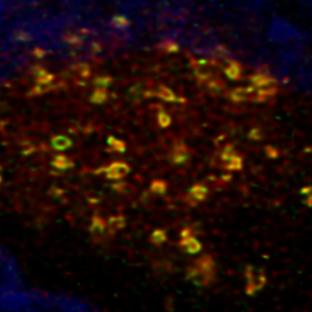

Supplement: Supplementary file 9 — Source data Fig. 4 [file 44319_2026_773_MOESM9_ESM.zip › Figure 4/Figure 4C/IF GRASP55KO+WT LIMP2 LAMP2 MERGE inset.tif]

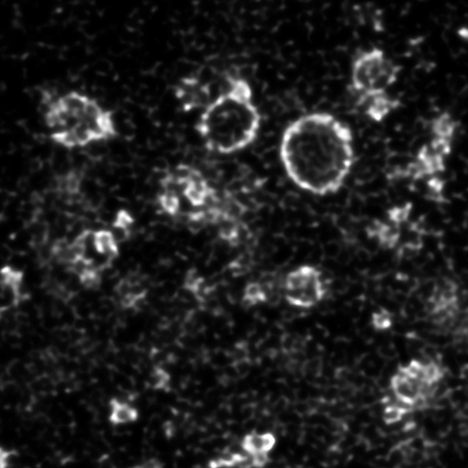

Supplement: Supplementary file 9 — Source data Fig. 4 [file 44319_2026_773_MOESM9_ESM.zip › Figure 4/Figure 4A/IF GRASP55KO LAMP2inset.tif]

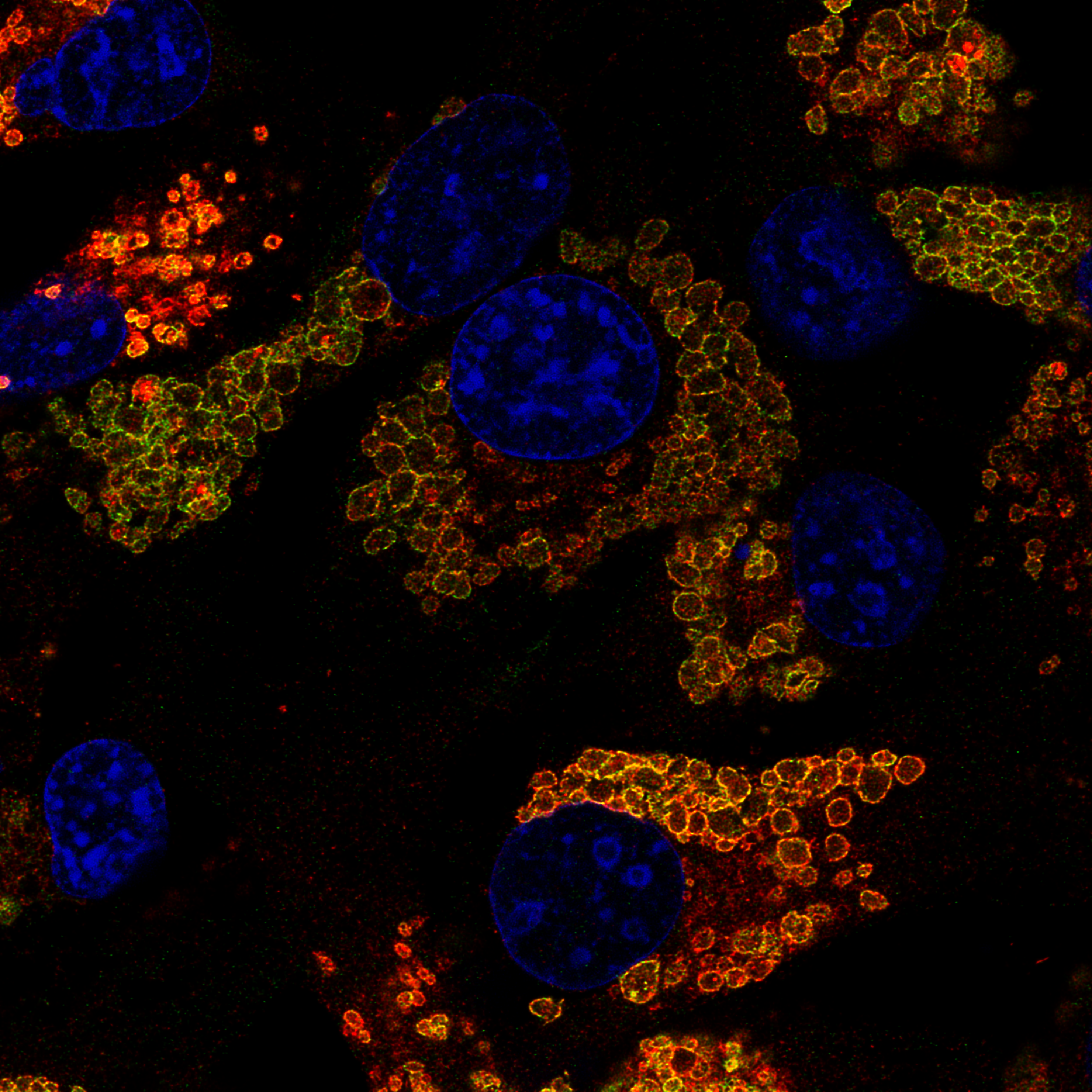

Supplement: Supplementary file 9 — Source data Fig. 4 [file 44319_2026_773_MOESM9_ESM.zip › Figure 4/Figure 4A/IF GNPTABKO LIMP2 LAMP2 MERGE .tif]

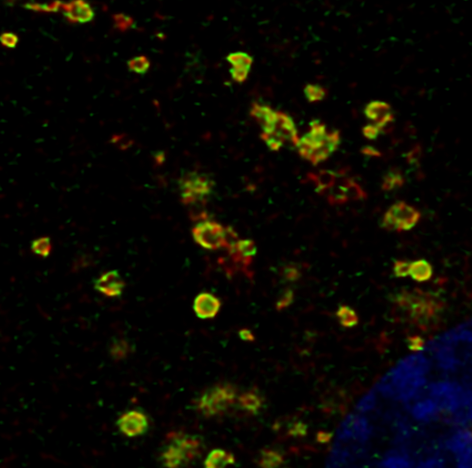

Supplement: Supplementary file 9 — Source data Fig. 4 [file 44319_2026_773_MOESM9_ESM.zip › Figure 4/Figure 4A/IF WT LIMP2 LAMP2 MERGE inset.tif]

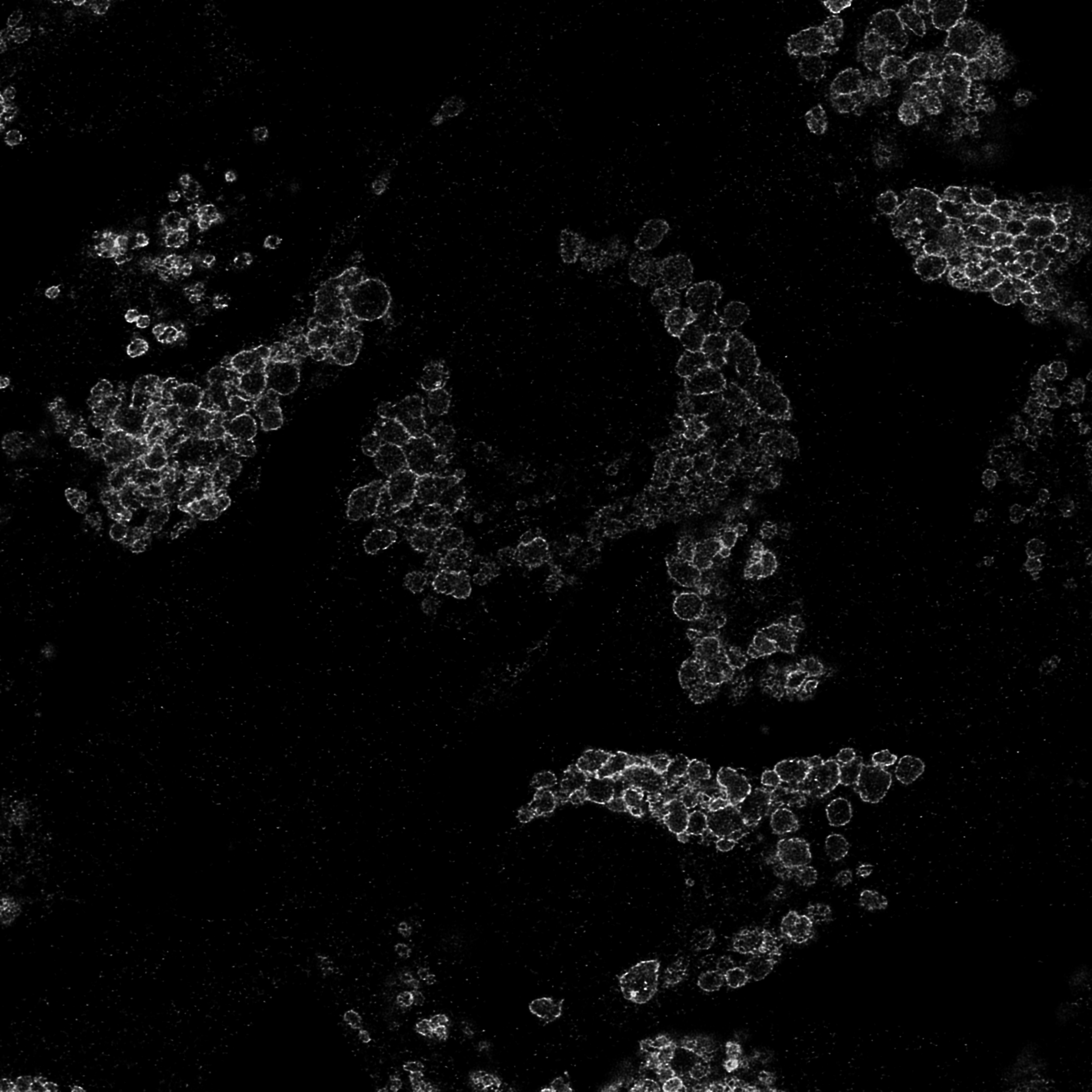

Supplement: Supplementary file 9 — Source data Fig. 4 [file 44319_2026_773_MOESM9_ESM.zip › Figure 4/Figure 4A/IF GNPTABKO LIMP2.tif]

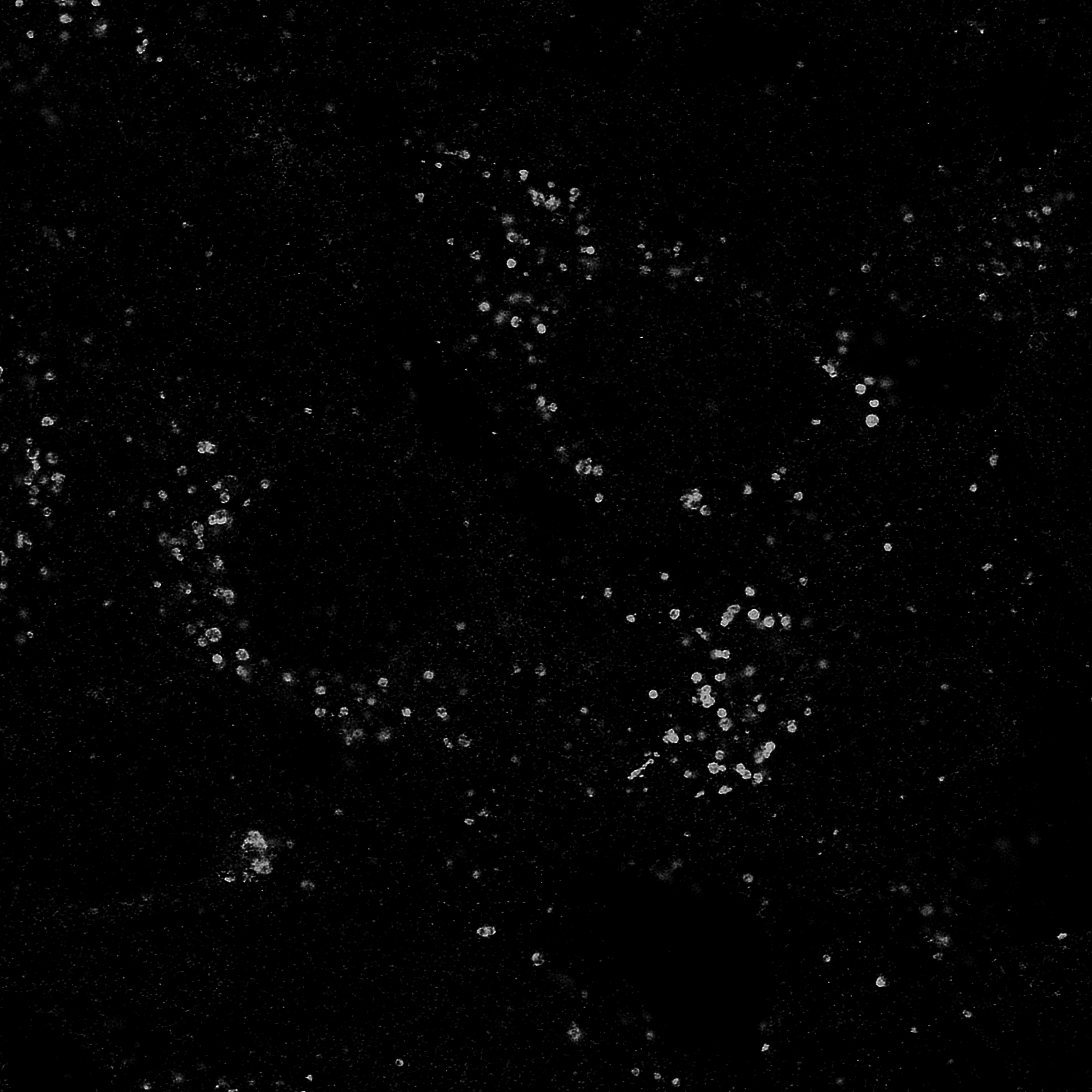

Supplement: Supplementary file 9 — Source data Fig. 4 [file 44319_2026_773_MOESM9_ESM.zip › Figure 4/Figure 4A/IF WT LIMP2.tif]

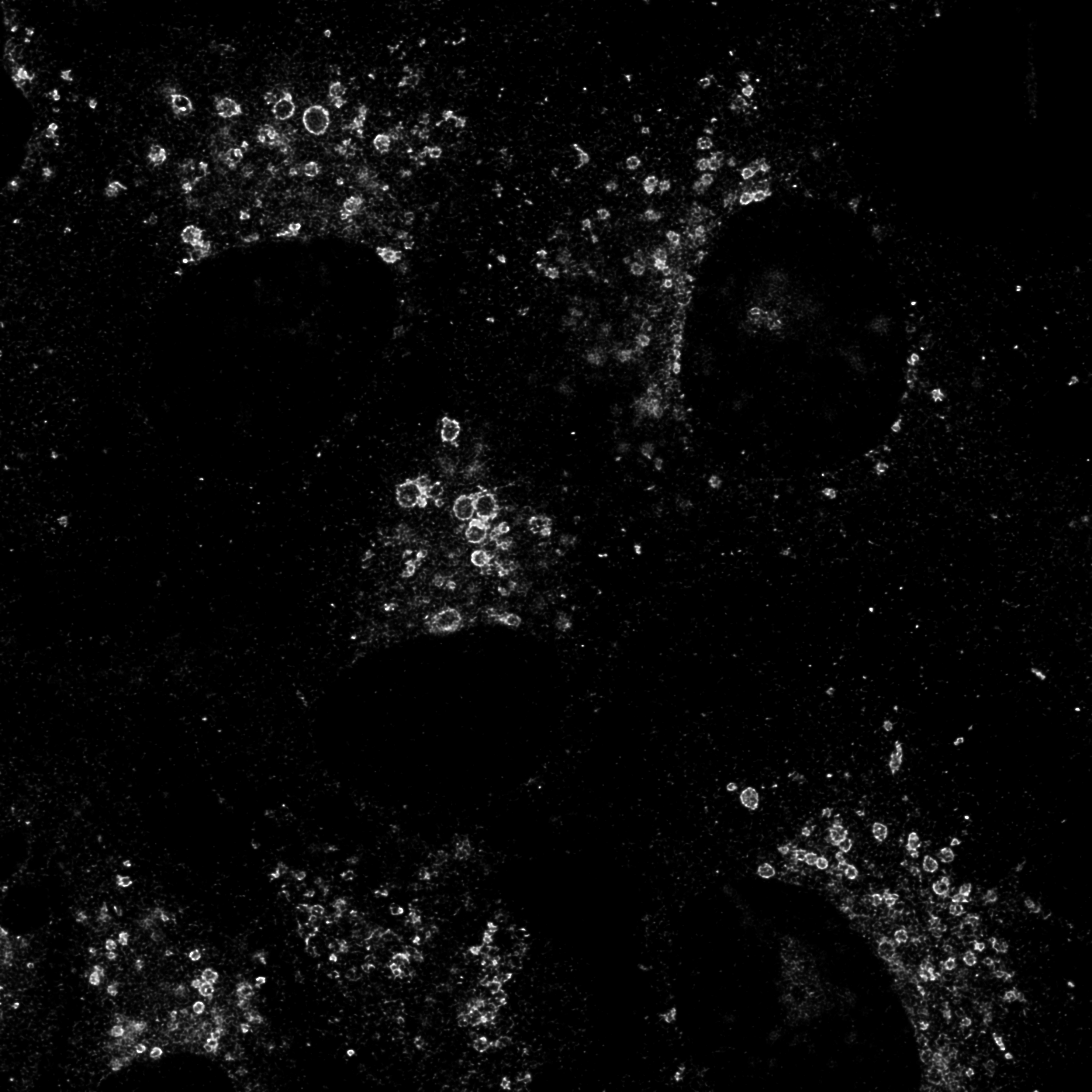

Supplement: Supplementary file 9 — Source data Fig. 4 [file 44319_2026_773_MOESM9_ESM.zip › Figure 4/Figure 4A/IF GRASP55KO LAMP2.tif]

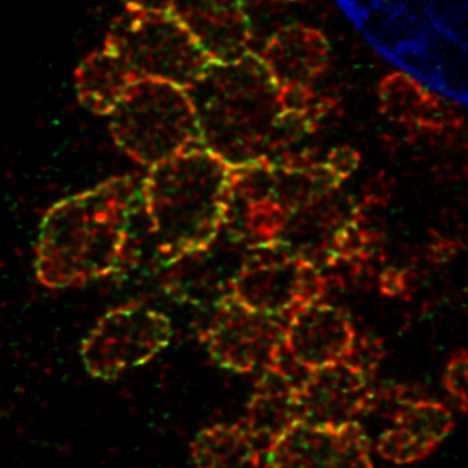

Supplement: Supplementary file 9 — Source data Fig. 4 [file 44319_2026_773_MOESM9_ESM.zip › Figure 4/Figure 4A/IF GNPTABKO LIMP2 LAMP2 MERGE inset.tif]

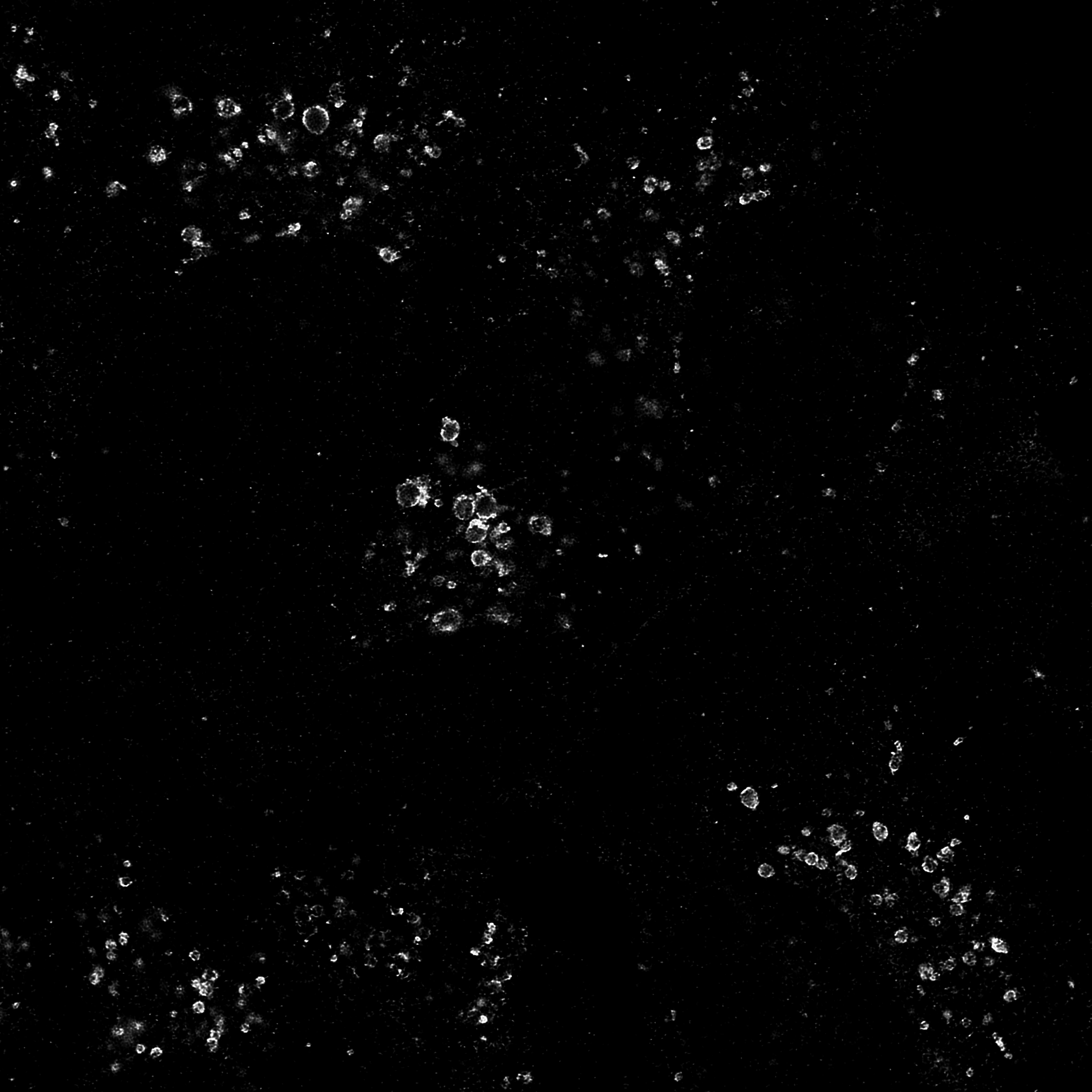

Supplement: Supplementary file 9 — Source data Fig. 4 [file 44319_2026_773_MOESM9_ESM.zip › Figure 4/Figure 4A/IF GRASP55KO LIMP2.tif]

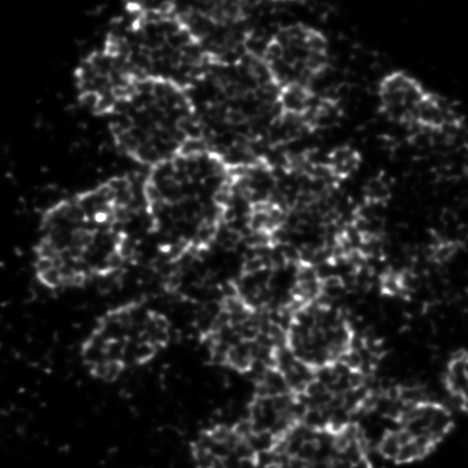

Supplement: Supplementary file 9 — Source data Fig. 4 [file 44319_2026_773_MOESM9_ESM.zip › Figure 4/Figure 4A/IF GNPTABKO LAMP2 inset.tif]

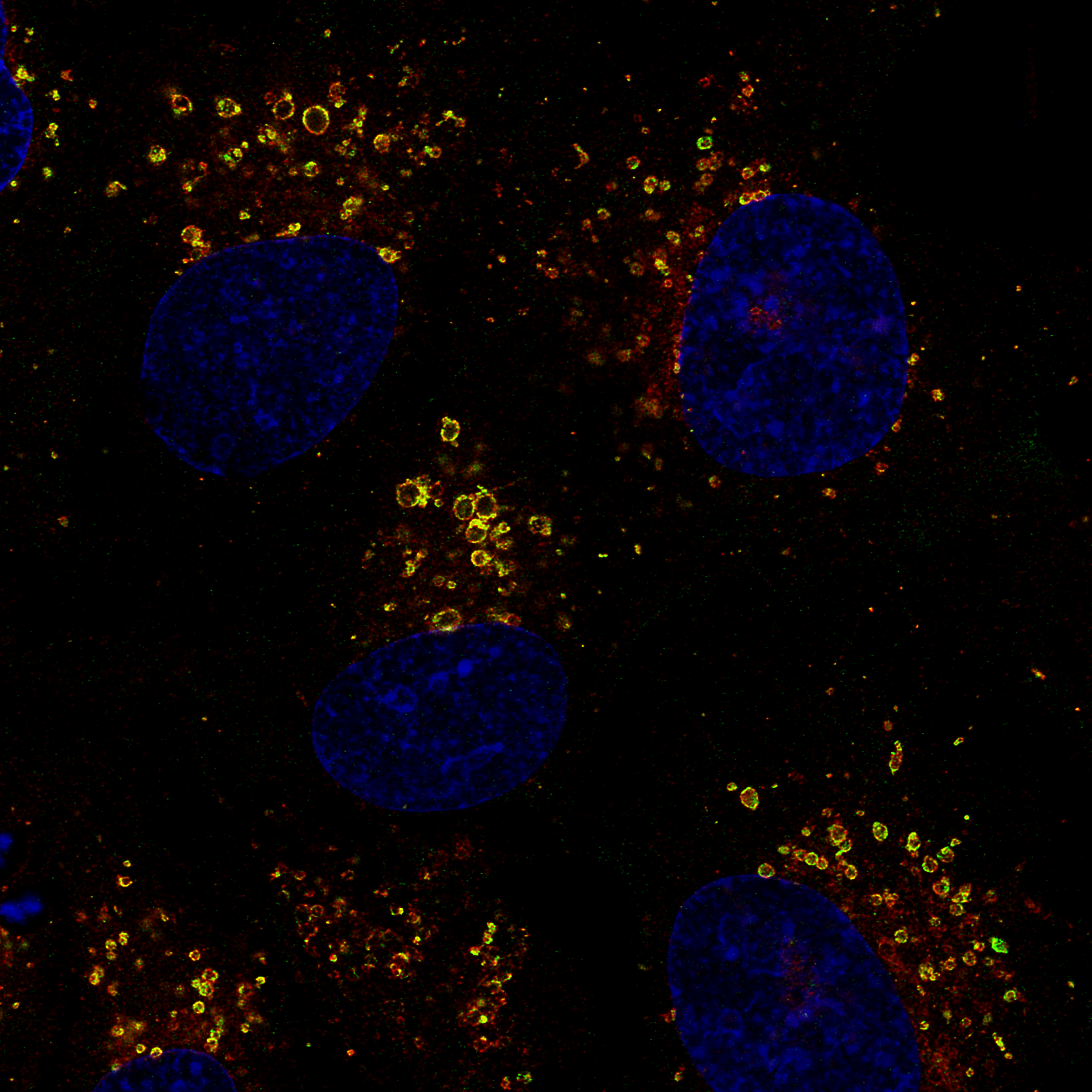

Supplement: Supplementary file 9 — Source data Fig. 4 [file 44319_2026_773_MOESM9_ESM.zip › Figure 4/Figure 4A/IF GRASP55KO LIMP2 LAMP2 MERGE.tif]

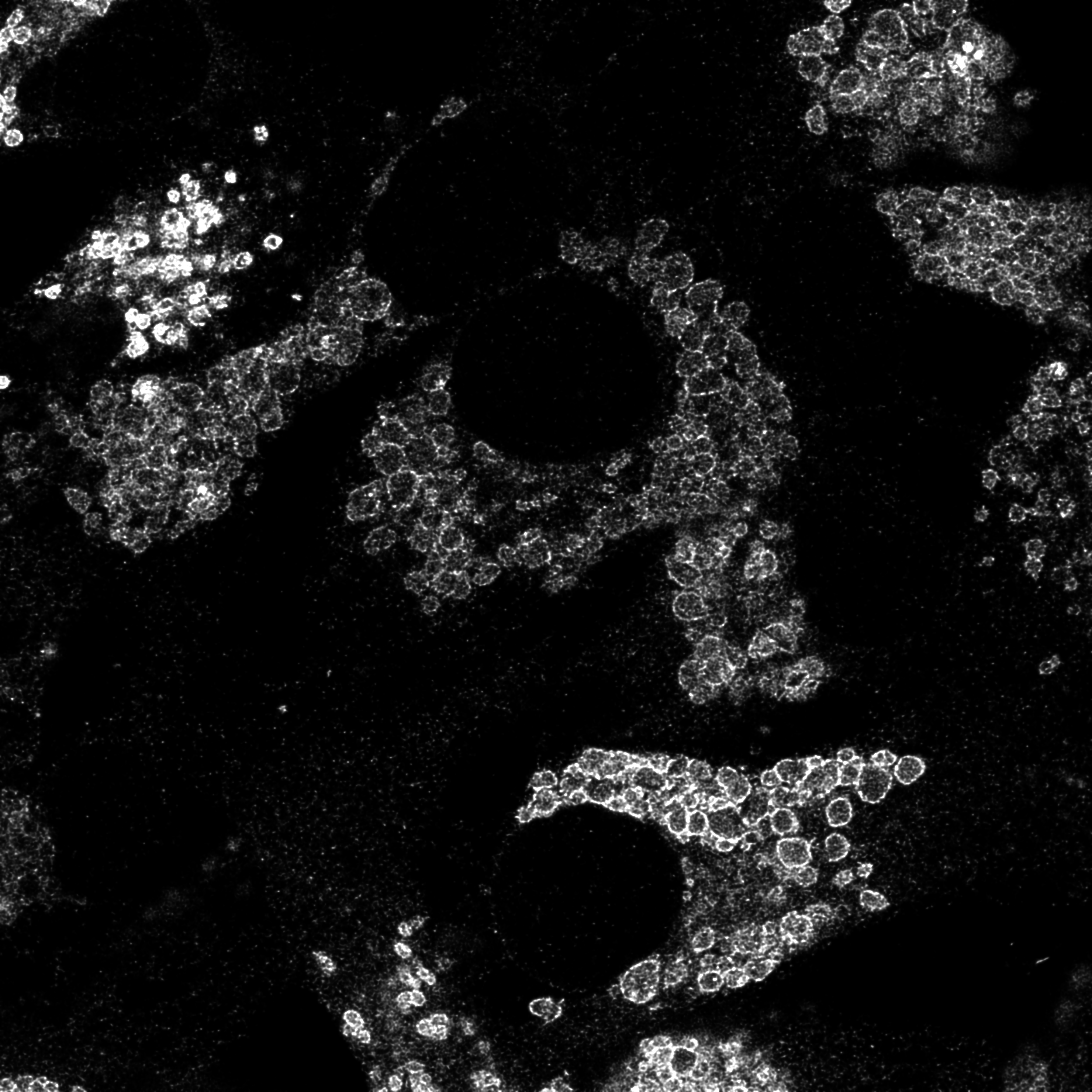

Supplement: Supplementary file 9 — Source data Fig. 4 [file 44319_2026_773_MOESM9_ESM.zip › Figure 4/Figure 4A/IF GNPTABKO LAMP2.tif]

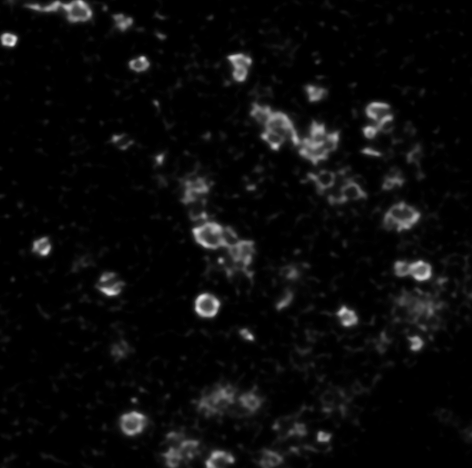

Supplement: Supplementary file 9 — Source data Fig. 4 [file 44319_2026_773_MOESM9_ESM.zip › Figure 4/Figure 4A/IF WT LAMP2 inset.tif.tif]

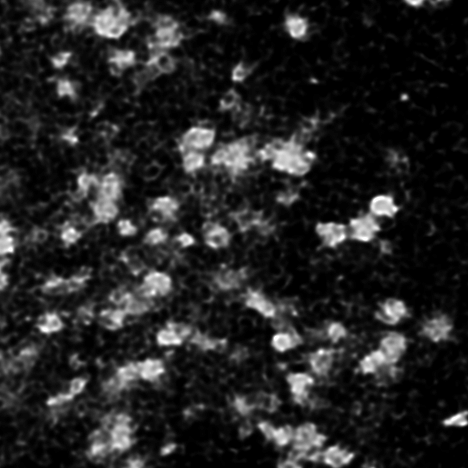

Supplement: Supplementary file 9 — Source data Fig. 4 [file 44319_2026_773_MOESM9_ESM.zip › Figure 4/Figure 4A/IF GRASP65KO LAMP2 inset.tif]

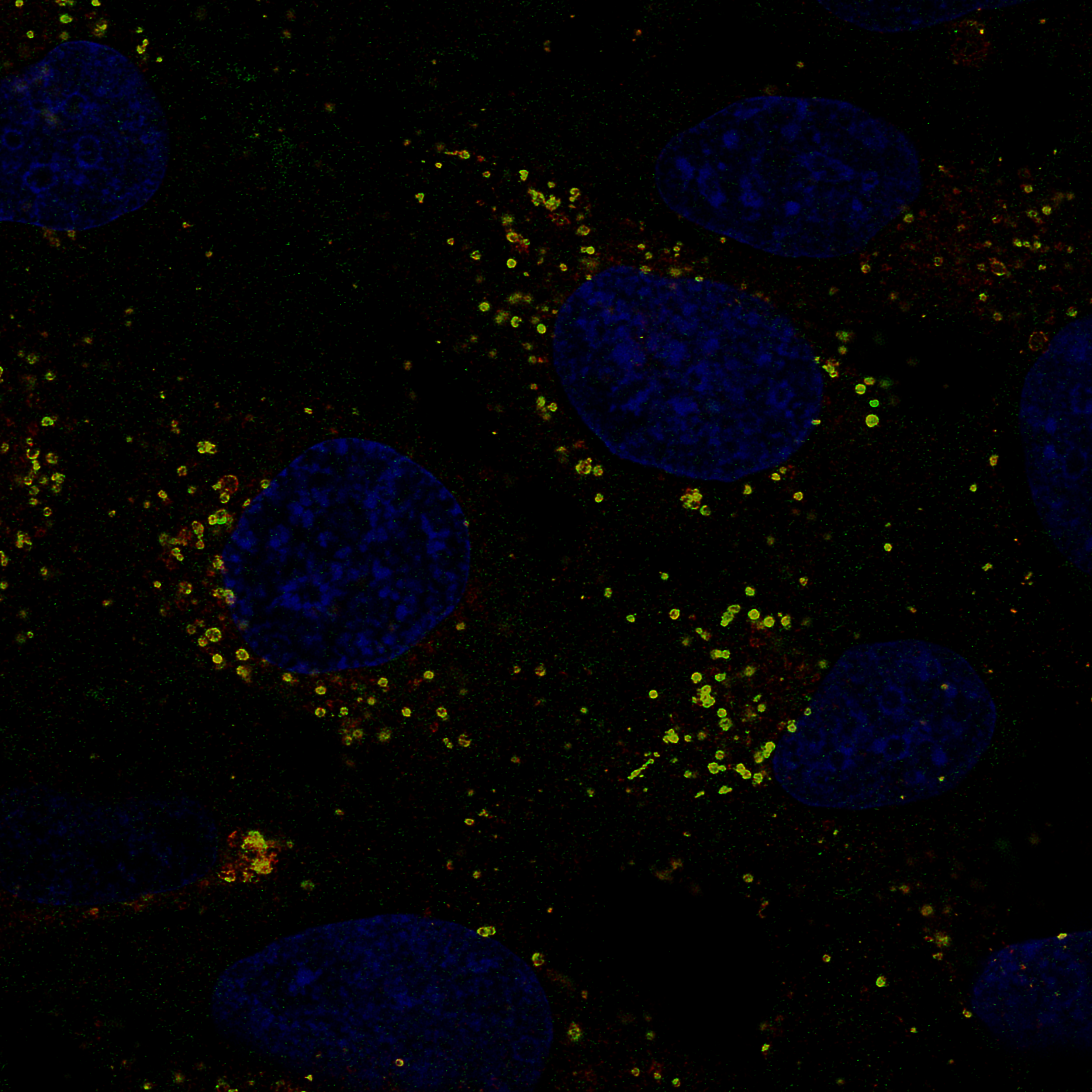

Supplement: Supplementary file 9 — Source data Fig. 4 [file 44319_2026_773_MOESM9_ESM.zip › Figure 4/Figure 4A/IF WT LIMP2 LAMP2 MERGE.tif]

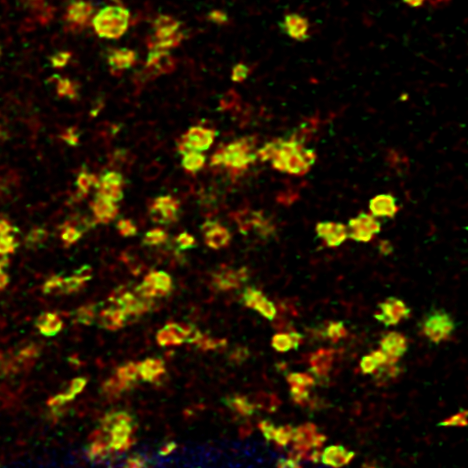

Supplement: Supplementary file 9 — Source data Fig. 4 [file 44319_2026_773_MOESM9_ESM.zip › Figure 4/Figure 4A/IF GRASP65KO LIMP2 LAMP2 MERGE inset.tif]

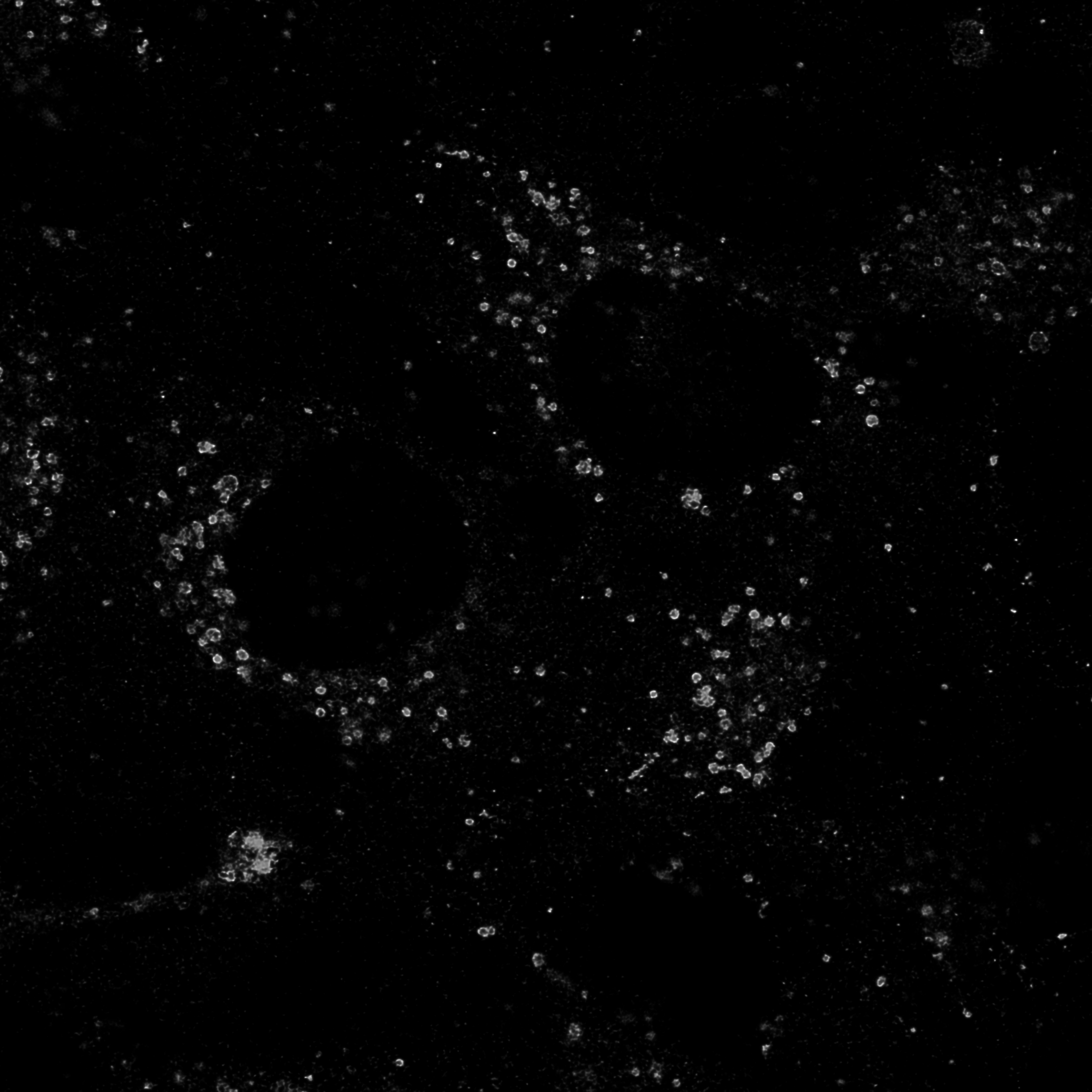

Supplement: Supplementary file 9 — Source data Fig. 4 [file 44319_2026_773_MOESM9_ESM.zip › Figure 4/Figure 4A/IF WT LAMP2.tif]

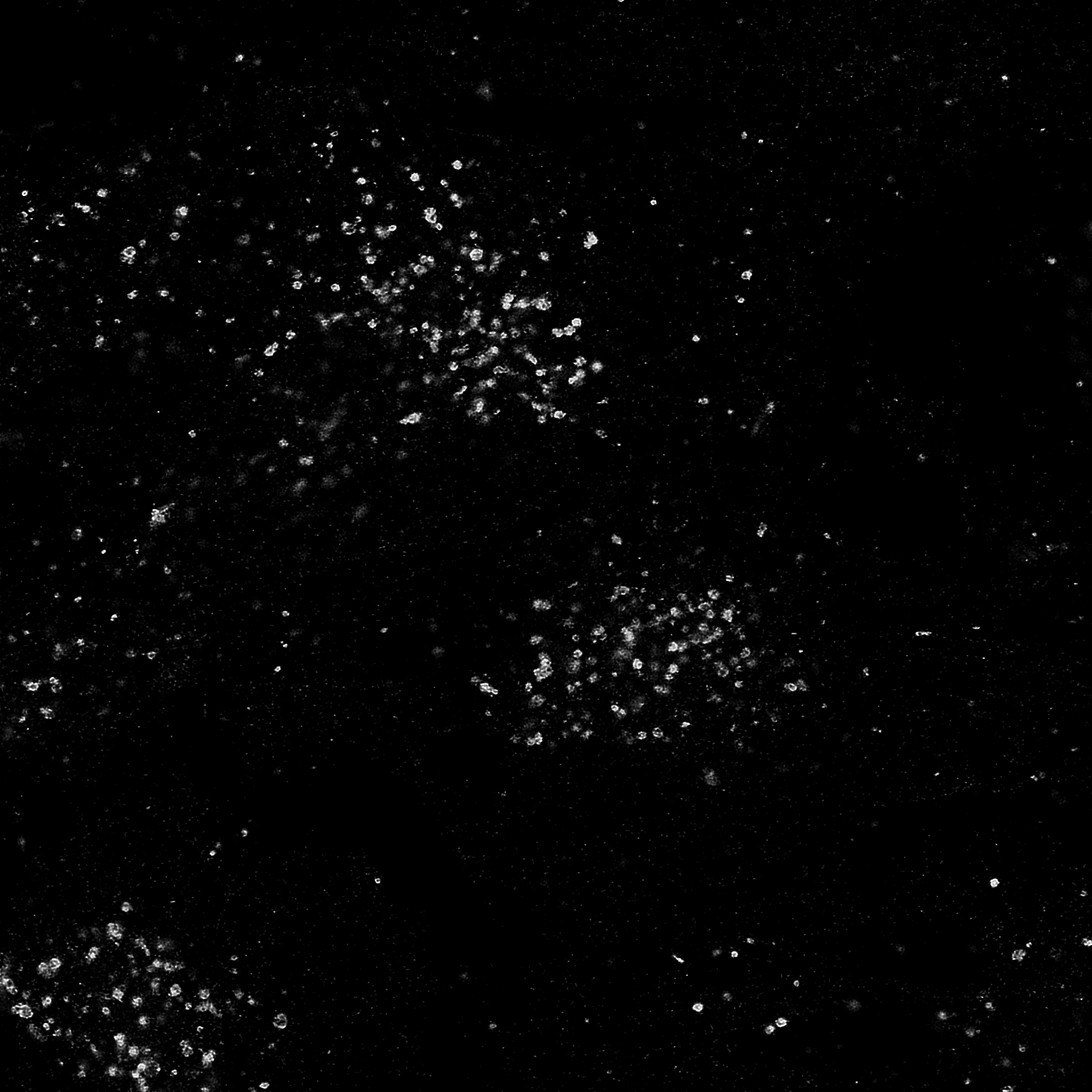

Supplement: Supplementary file 9 — Source data Fig. 4 [file 44319_2026_773_MOESM9_ESM.zip › Figure 4/Figure 4A/IF GRASP65KO LIMP2.tif]

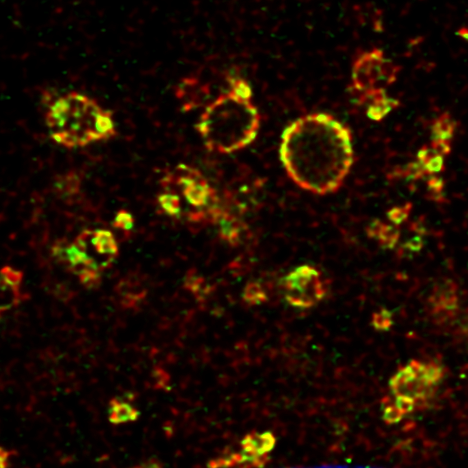

Supplement: Supplementary file 9 — Source data Fig. 4 [file 44319_2026_773_MOESM9_ESM.zip › Figure 4/Figure 4A/IF GRASP55KO LIMP2 LAMP2 MERGE inset.tif]

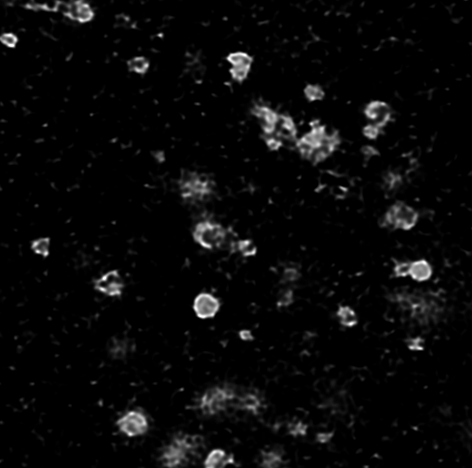

Supplement: Supplementary file 9 — Source data Fig. 4 [file 44319_2026_773_MOESM9_ESM.zip › Figure 4/Figure 4A/IF WT LIMP2 inset.tif.tif]

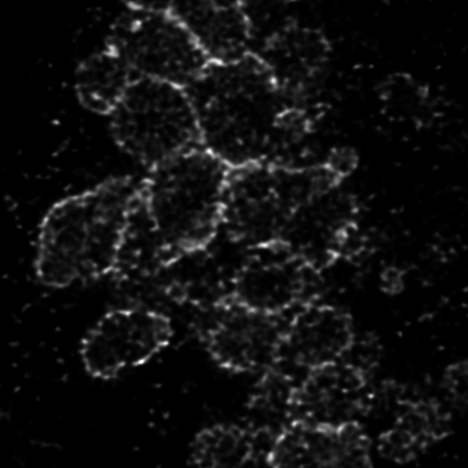

Supplement: Supplementary file 9 — Source data Fig. 4 [file 44319_2026_773_MOESM9_ESM.zip › Figure 4/Figure 4A/IF GNPTABKO LIMP2 inset.tif]

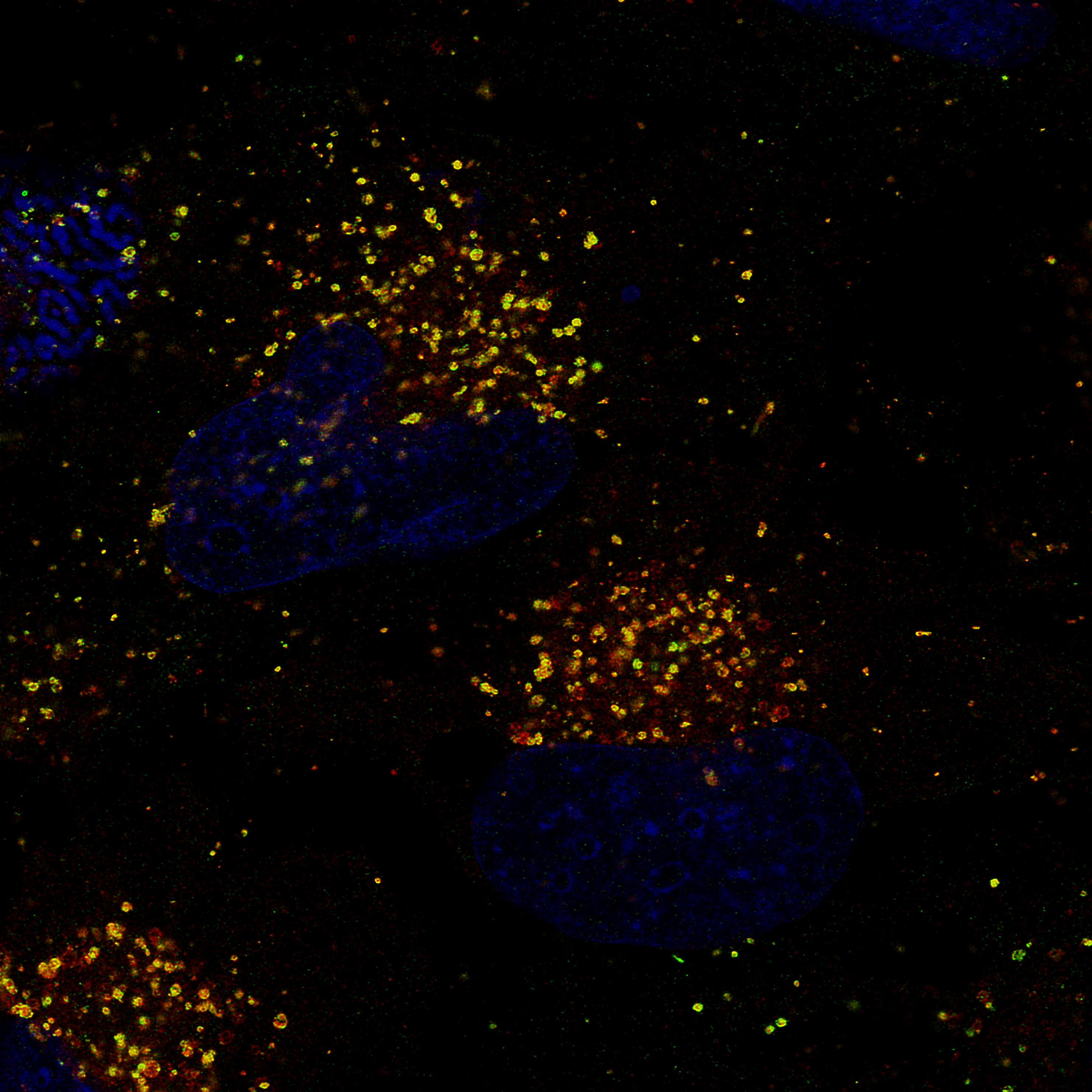

Supplement: Supplementary file 9 — Source data Fig. 4 [file 44319_2026_773_MOESM9_ESM.zip › Figure 4/Figure 4A/IF GRASP65KO LIMP2 LAMP2 MERGE.tif]

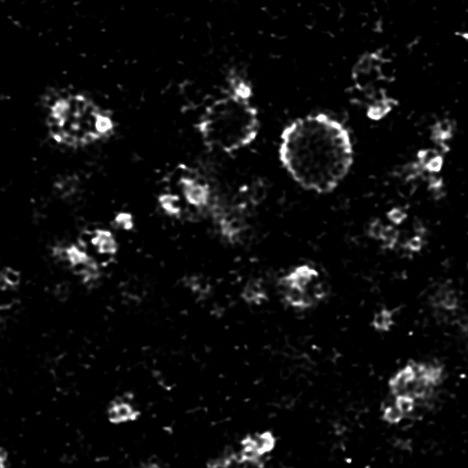

Supplement: Supplementary file 9 — Source data Fig. 4 [file 44319_2026_773_MOESM9_ESM.zip › Figure 4/Figure 4A/IF GRASP55KO LIMP2 inset.tif]

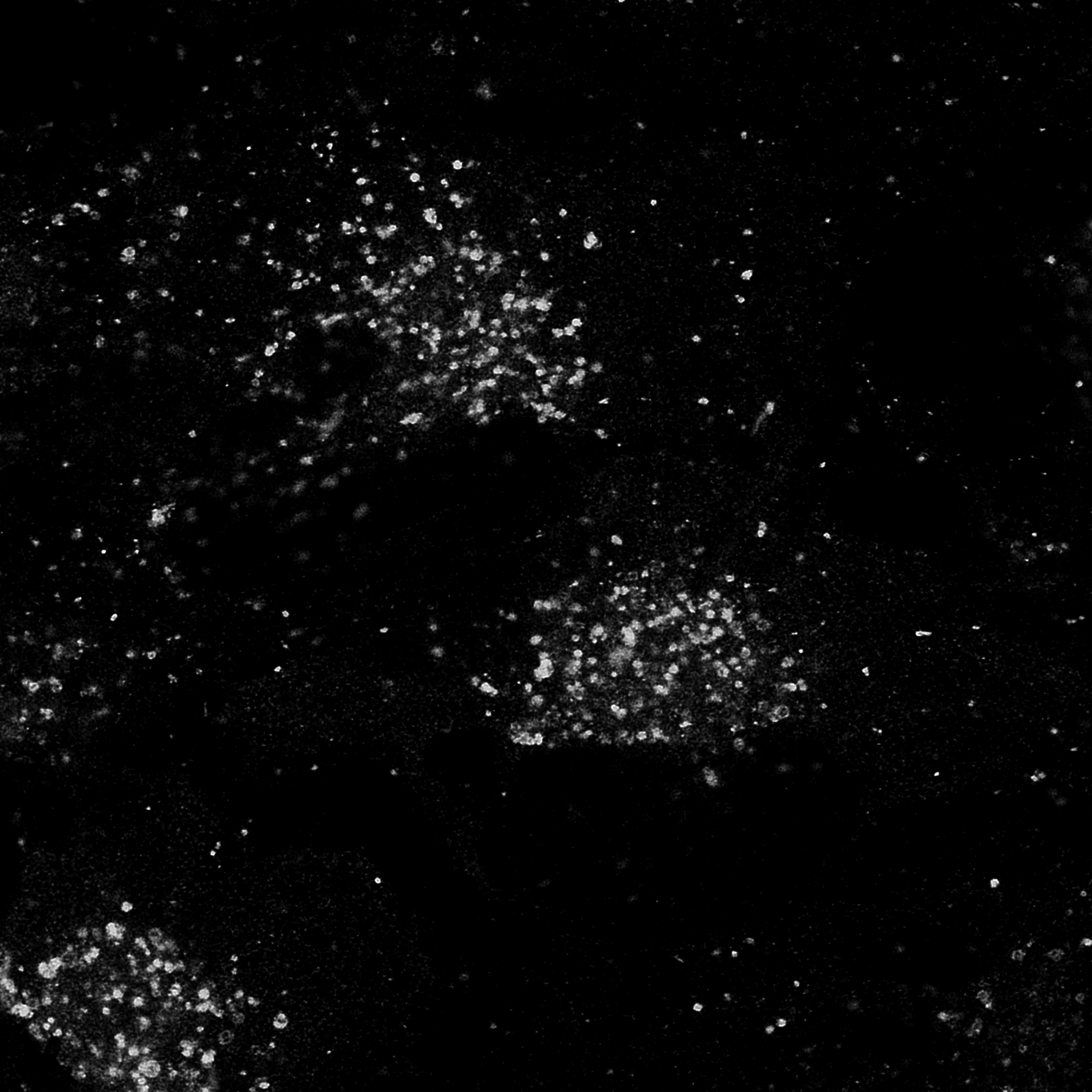

Supplement: Supplementary file 9 — Source data Fig. 4 [file 44319_2026_773_MOESM9_ESM.zip › Figure 4/Figure 4A/IF GRASP65KO LAMP2.tif]

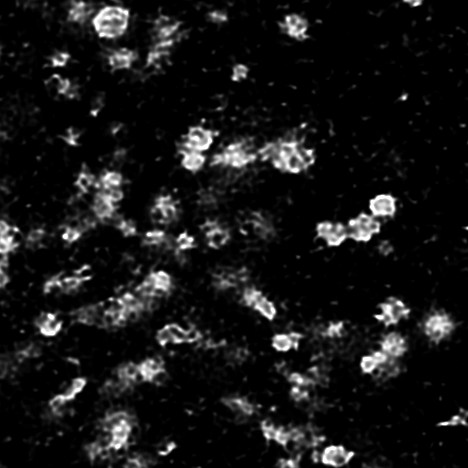

Supplement: Supplementary file 9 — Source data Fig. 4 [file 44319_2026_773_MOESM9_ESM.zip › Figure 4/Figure 4A/IF GRASP65KO LIMP2 inset.tif]

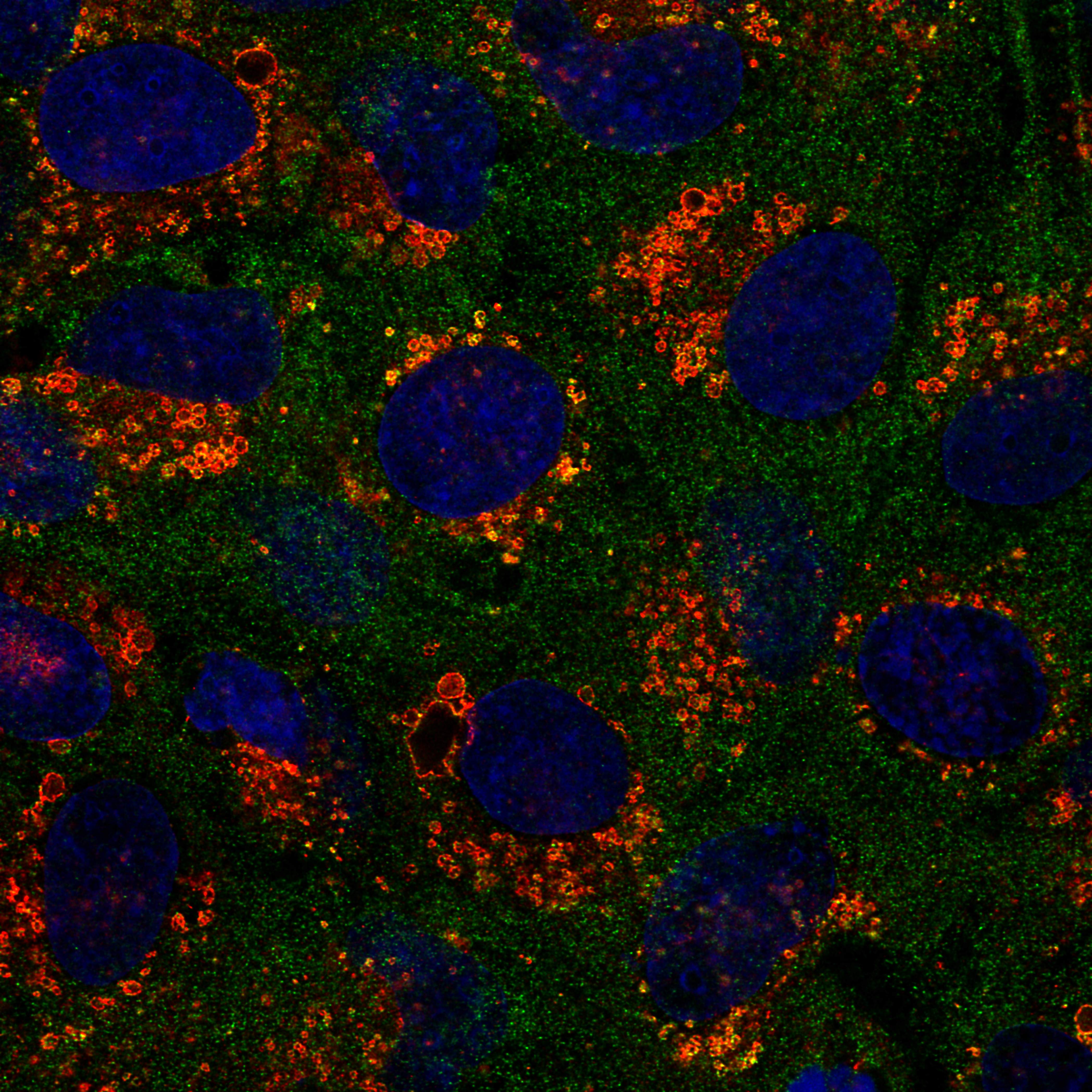

Supplement: Supplementary file 10 — Source data Fig. 5 [file 44319_2026_773_MOESM10_ESM.zip › Figure 5/Figure 5A/IF GRASP55Ko mTOR LAMP2 -AA Merge.tif]

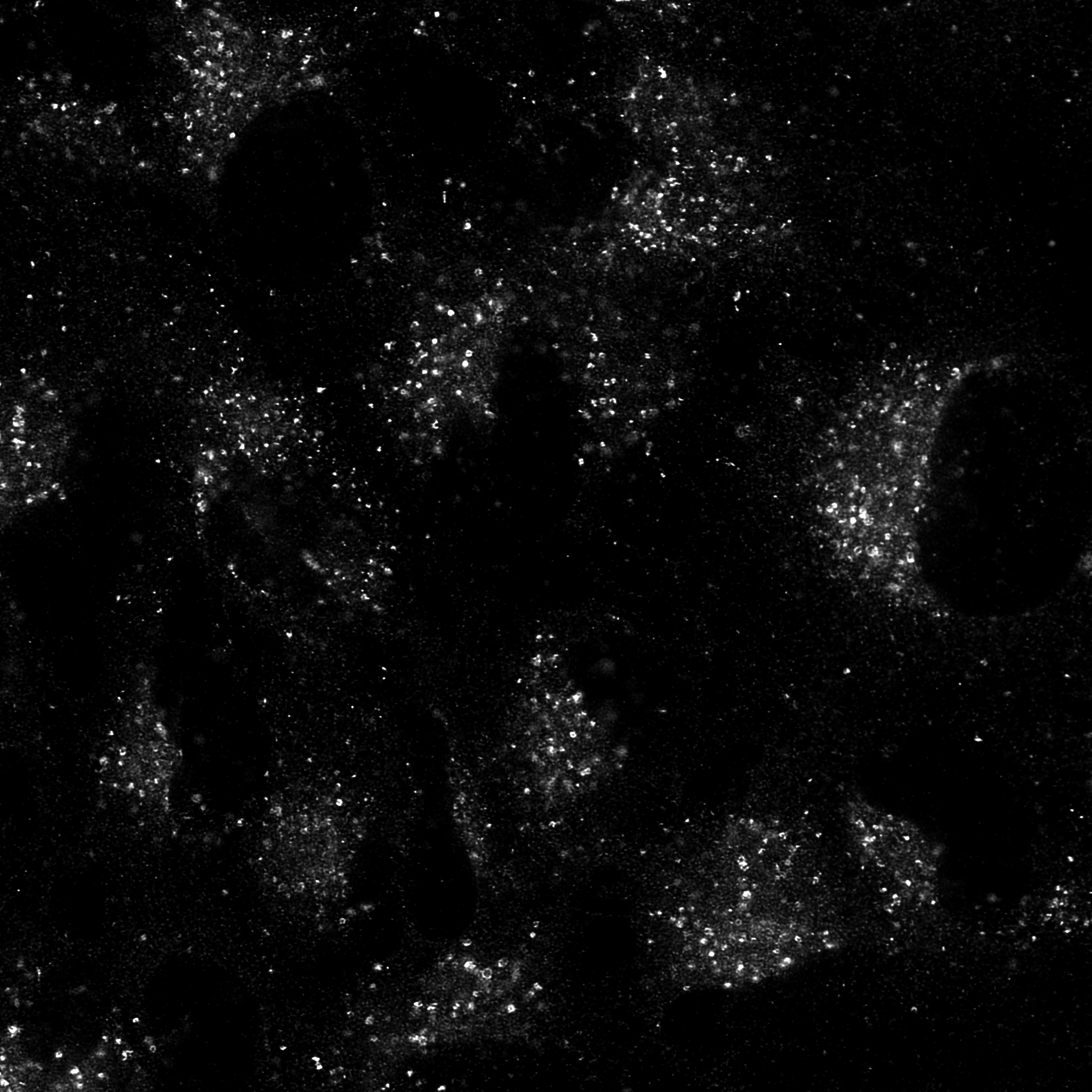

Supplement: Supplementary file 10 — Source data Fig. 5 [file 44319_2026_773_MOESM10_ESM.zip › Figure 5/Figure 5A/IF GRASP65KO LAMP2 +AA.tif]

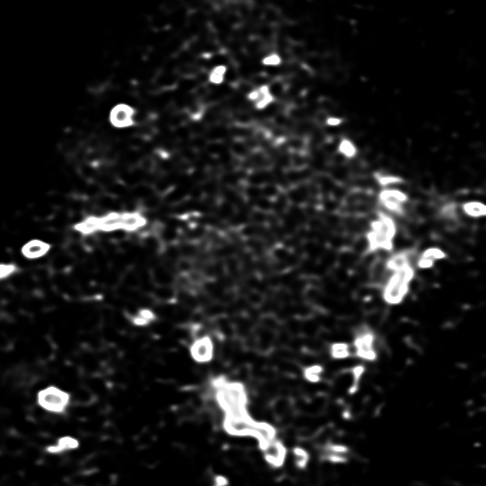

Supplement: Supplementary file 10 — Source data Fig. 5 [file 44319_2026_773_MOESM10_ESM.zip › Figure 5/Figure 5A/IF WT LAMP2 +AA inset.tif]

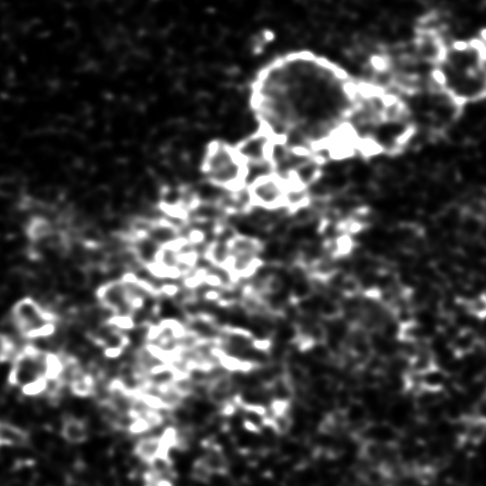

Supplement: Supplementary file 10 — Source data Fig. 5 [file 44319_2026_773_MOESM10_ESM.zip › Figure 5/Figure 5A/IF GRASP55Ko LAMP2 -AA .tif]

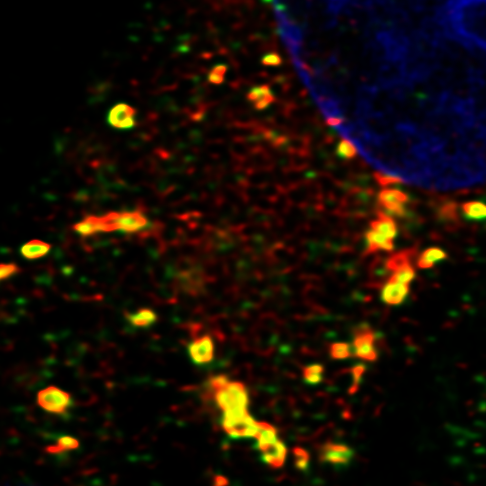

Supplement: Supplementary file 10 — Source data Fig. 5 [file 44319_2026_773_MOESM10_ESM.zip › Figure 5/Figure 5A/IF WT mTOR LAMP2 +AA MERGE inset.tif]

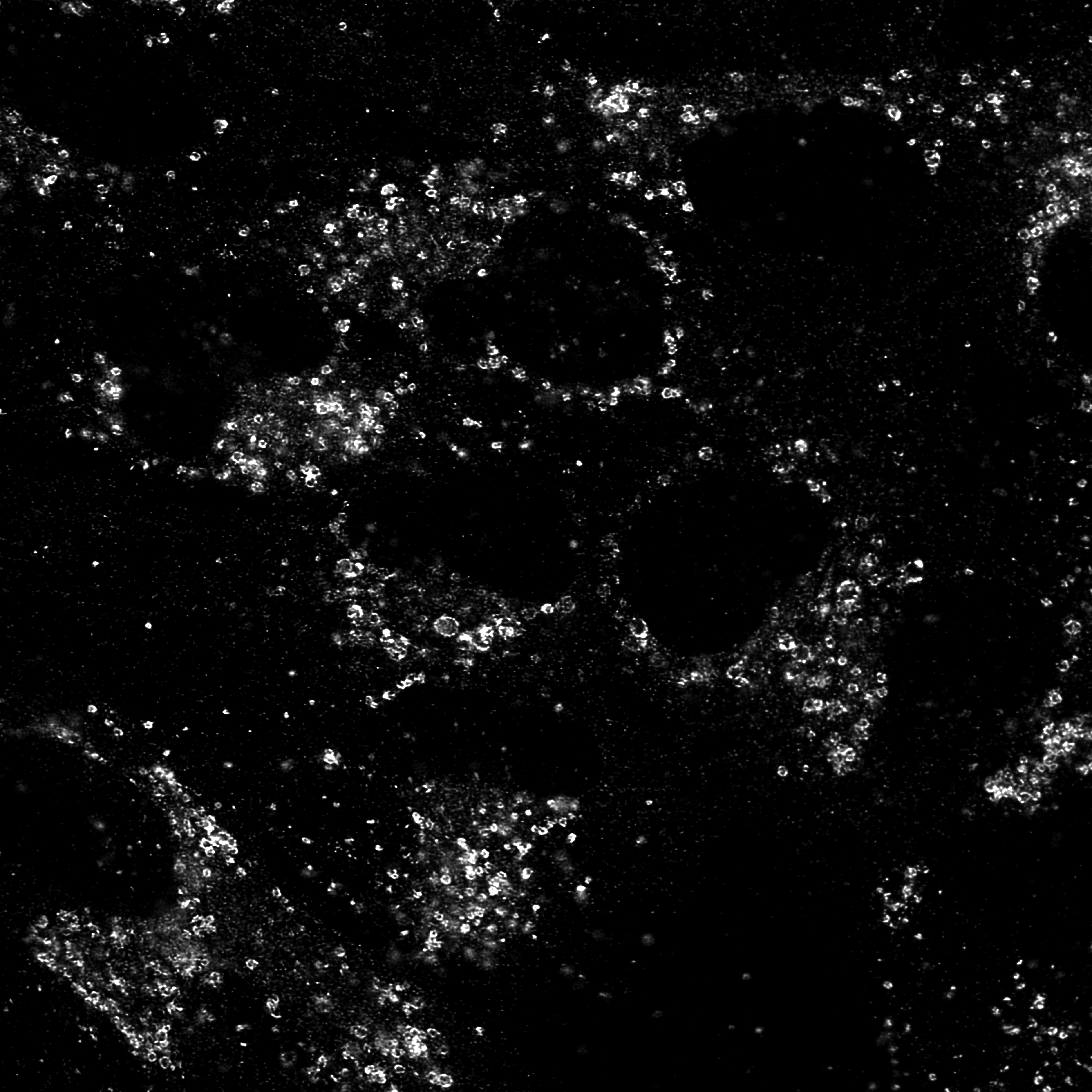

Supplement: Supplementary file 10 — Source data Fig. 5 [file 44319_2026_773_MOESM10_ESM.zip › Figure 5/Figure 5A/IF GRASP65KO LAMP2 -AA.tif]

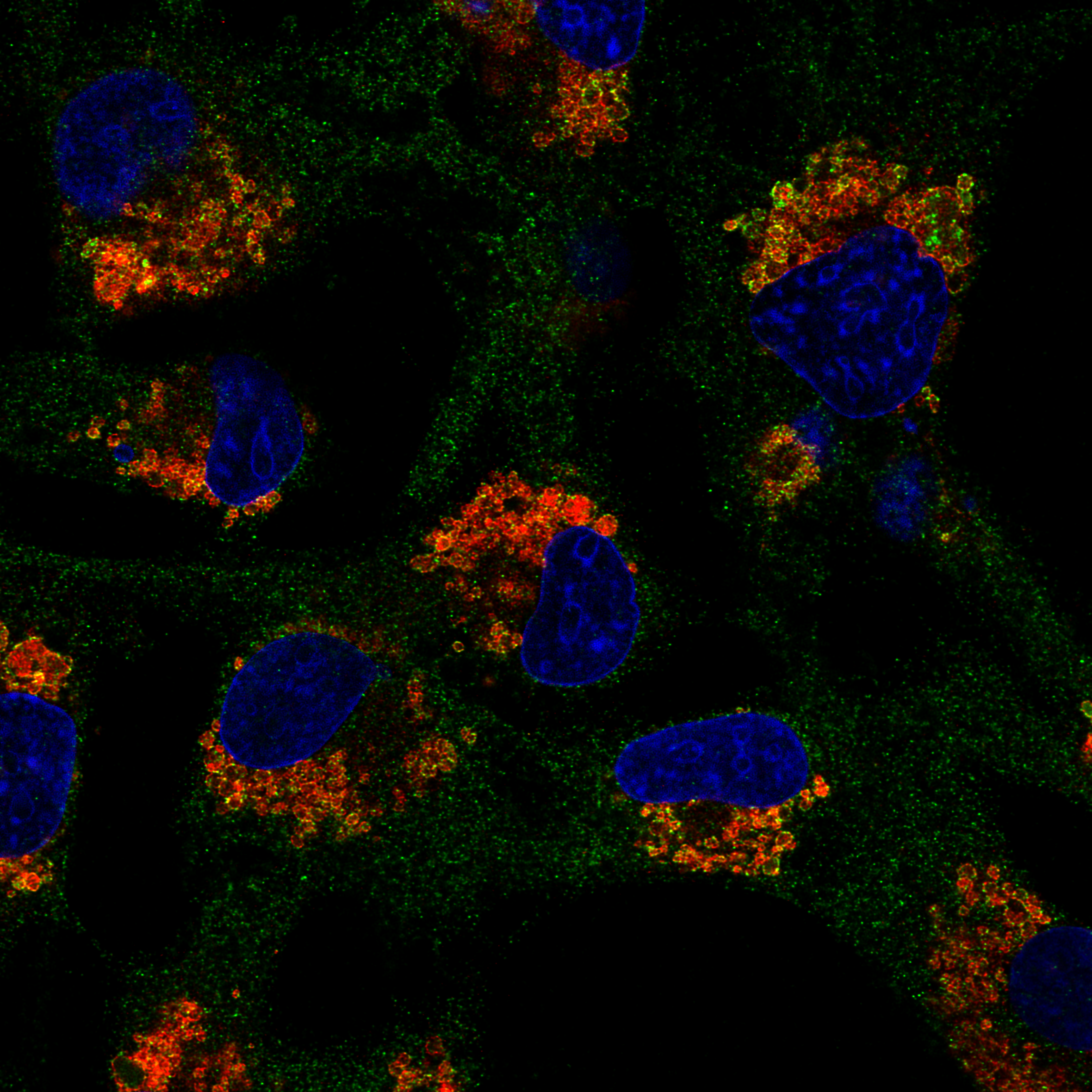

Supplement: Supplementary file 10 — Source data Fig. 5 [file 44319_2026_773_MOESM10_ESM.zip › Figure 5/Figure 5A/IF GNPTABKO mTOR LAMP2 +AA Merge.tif]

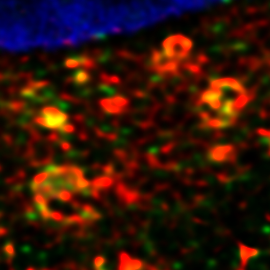

Supplement: Supplementary file 10 — Source data Fig. 5 [file 44319_2026_773_MOESM10_ESM.zip › Figure 5/Figure 5A/IF GRASP55KO mTOR LAMP2 +AA Merge inset.tif]

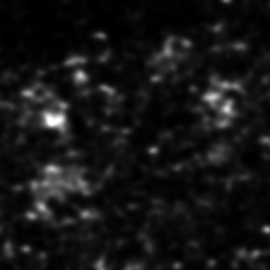

Supplement: Supplementary file 10 — Source data Fig. 5 [file 44319_2026_773_MOESM10_ESM.zip › Figure 5/Figure 5A/IF GRASP55KO mTOR +AA inset.tif]

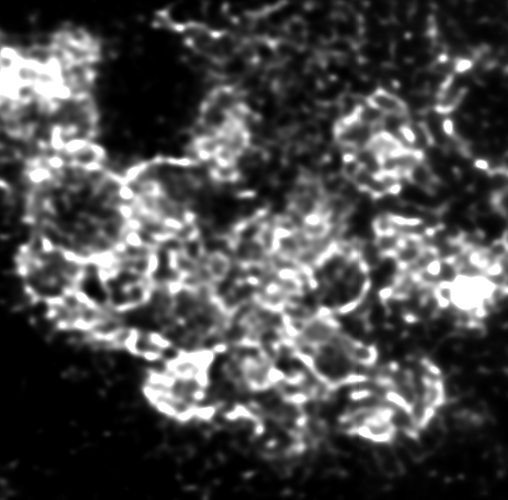

Supplement: Supplementary file 10 — Source data Fig. 5 [file 44319_2026_773_MOESM10_ESM.zip › Figure 5/Figure 5A/IF GNPTABKO LAMP2 -AA inset.tif]

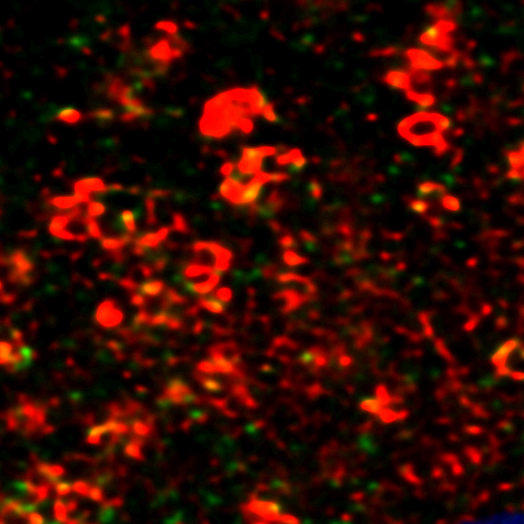

Supplement: Supplementary file 10 — Source data Fig. 5 [file 44319_2026_773_MOESM10_ESM.zip › Figure 5/Figure 5A/IF GRASP65KO mTOR LAMP2 -AA Merge inset.tif]

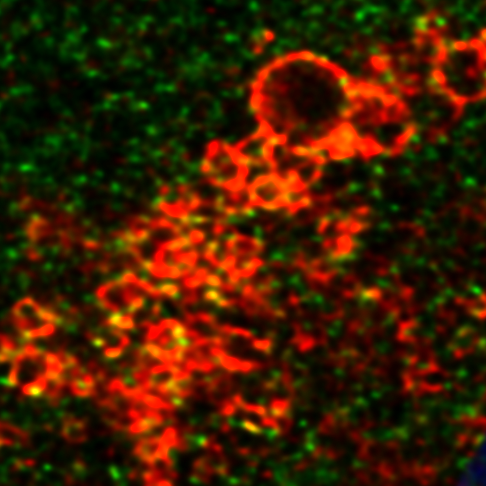

Supplement: Supplementary file 10 — Source data Fig. 5 [file 44319_2026_773_MOESM10_ESM.zip › Figure 5/Figure 5A/IF GRASP55Ko mTOR LAMP2 -AA Merge inset.tif]

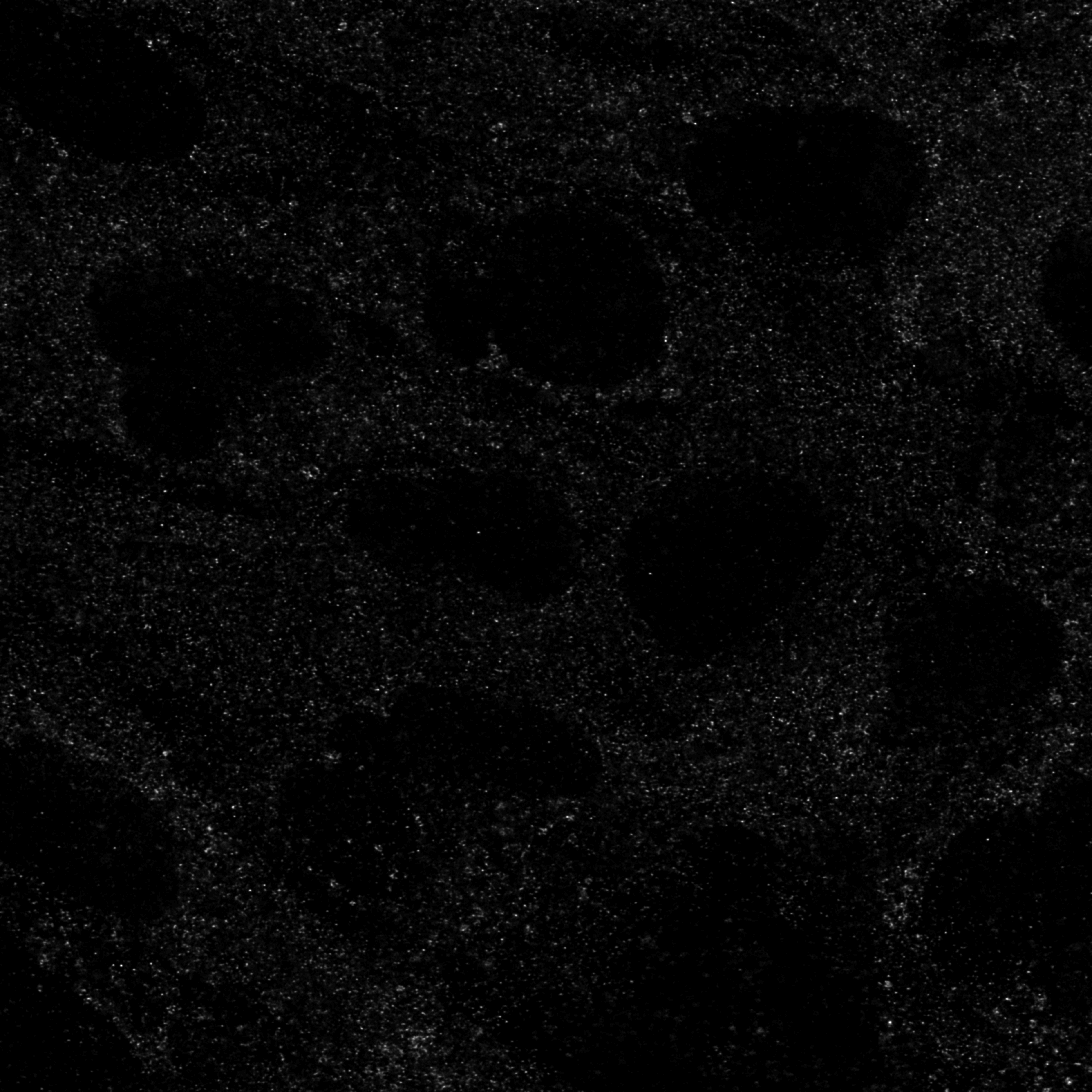

Supplement: Supplementary file 10 — Source data Fig. 5 [file 44319_2026_773_MOESM10_ESM.zip › Figure 5/Figure 5A/IF GRASP65KO mTOR -AA.tif]

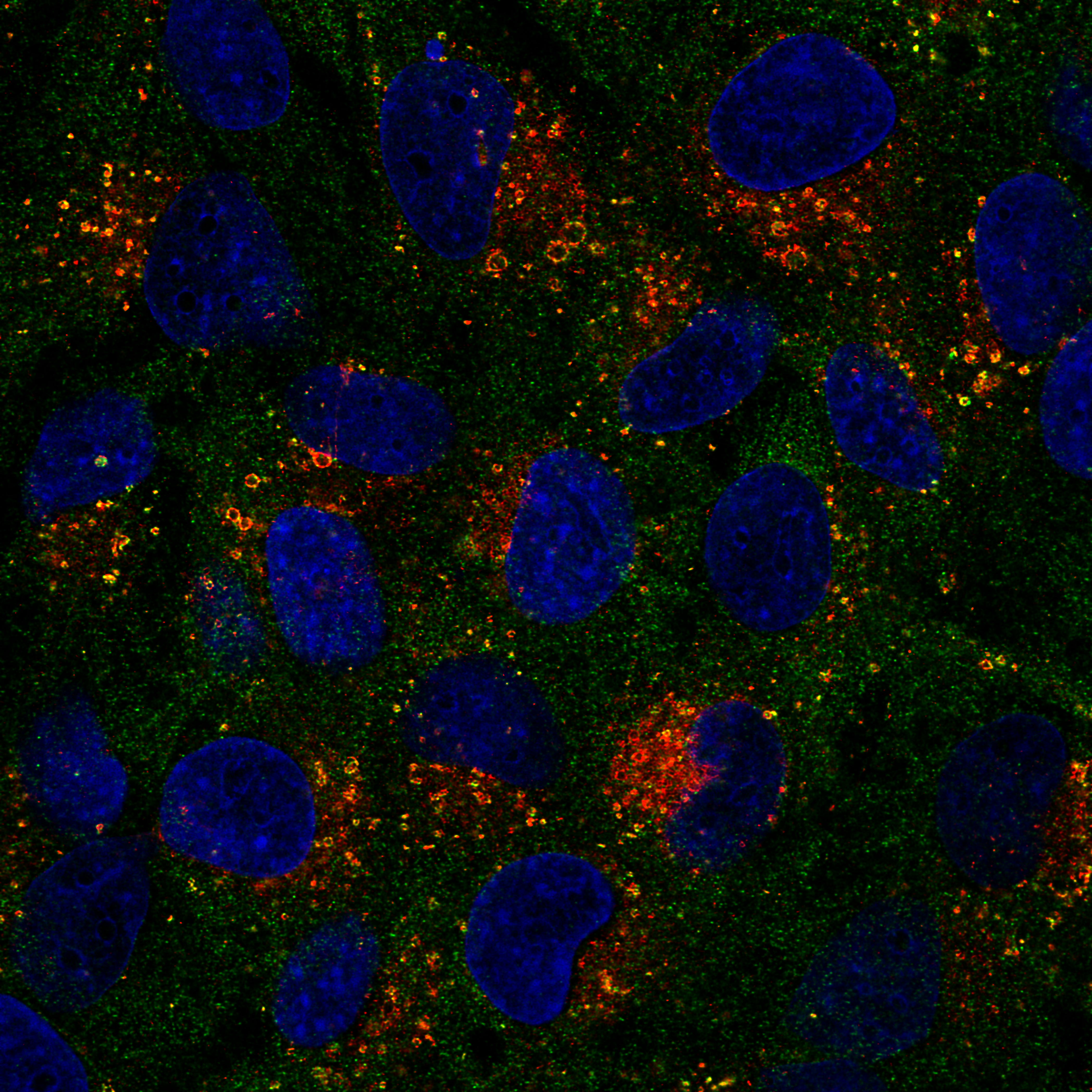

Supplement: Supplementary file 10 — Source data Fig. 5 [file 44319_2026_773_MOESM10_ESM.zip › Figure 5/Figure 5A/IF GRASP55KO mTOR LAMP2 +AA Merge.tif]

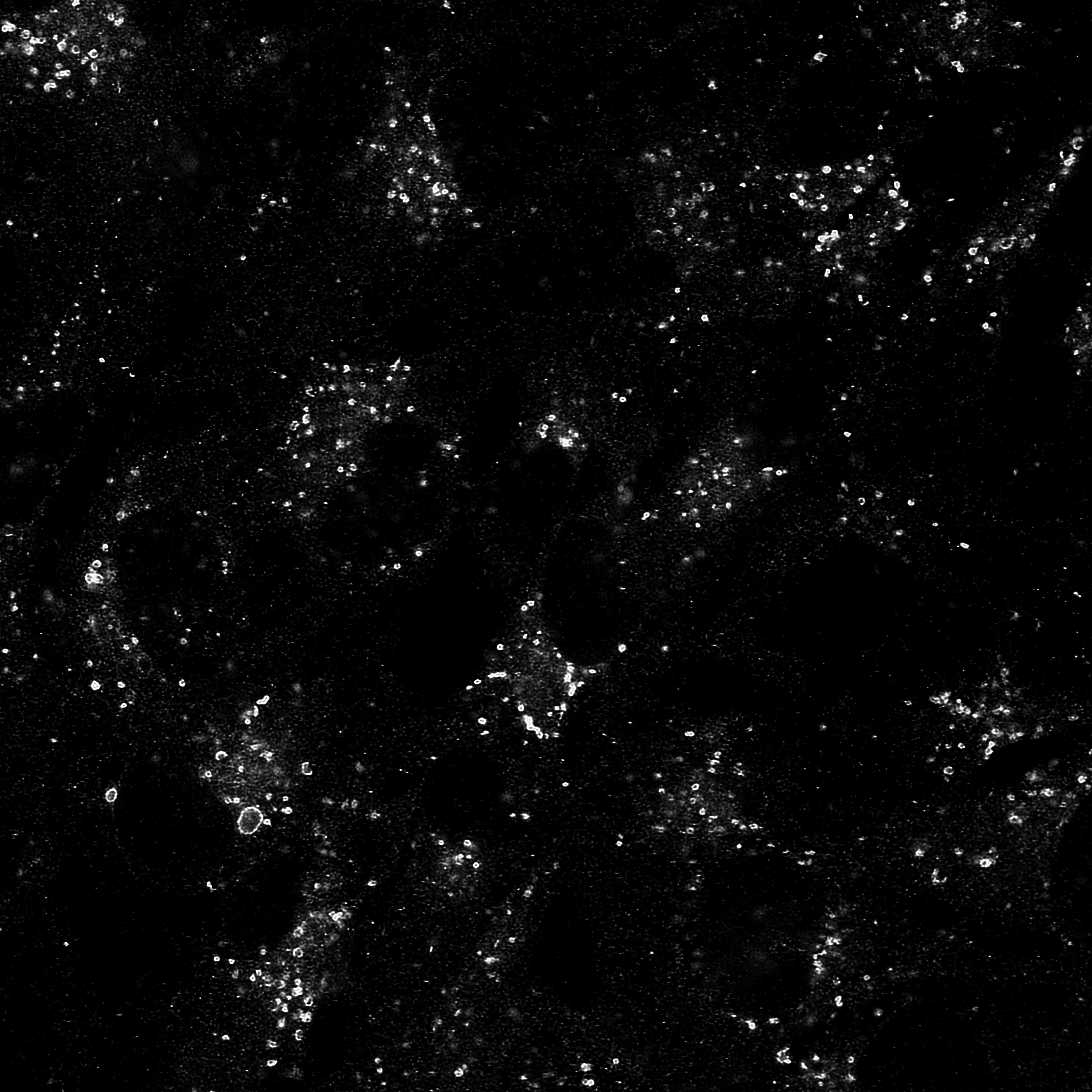

Supplement: Supplementary file 10 — Source data Fig. 5 [file 44319_2026_773_MOESM10_ESM.zip › Figure 5/Figure 5A/IF WT LAMP2 +AA.tif]

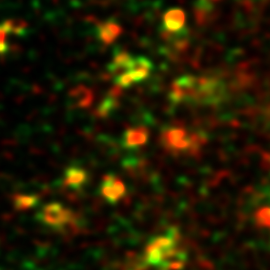

Supplement: Supplementary file 10 — Source data Fig. 5 [file 44319_2026_773_MOESM10_ESM.zip › Figure 5/Figure 5A/IF GRASP65KO mTOR LAMP2 +AA Merge inset.tif]

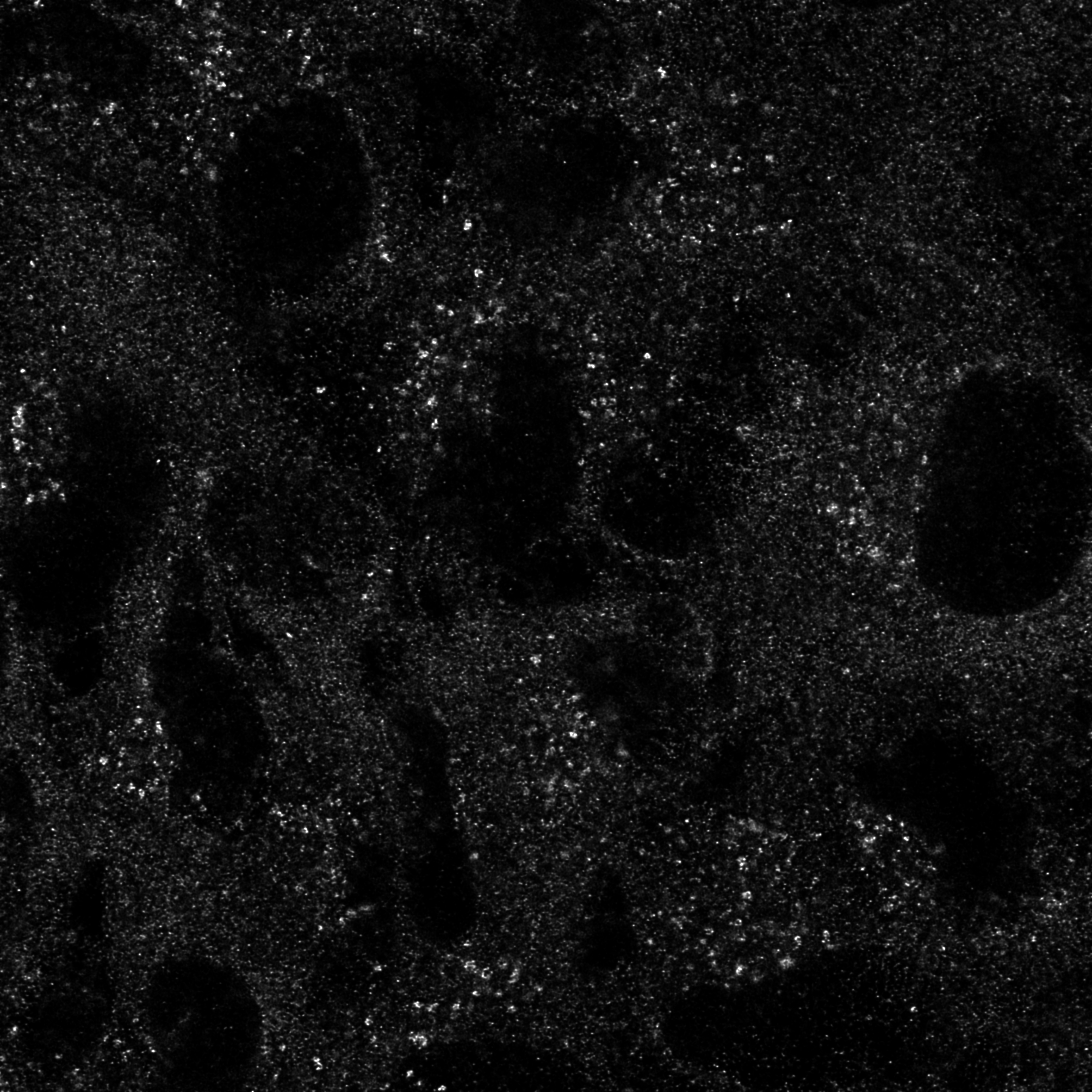

Supplement: Supplementary file 10 — Source data Fig. 5 [file 44319_2026_773_MOESM10_ESM.zip › Figure 5/Figure 5A/IF GRASP65KO mTOR +AA.tif]

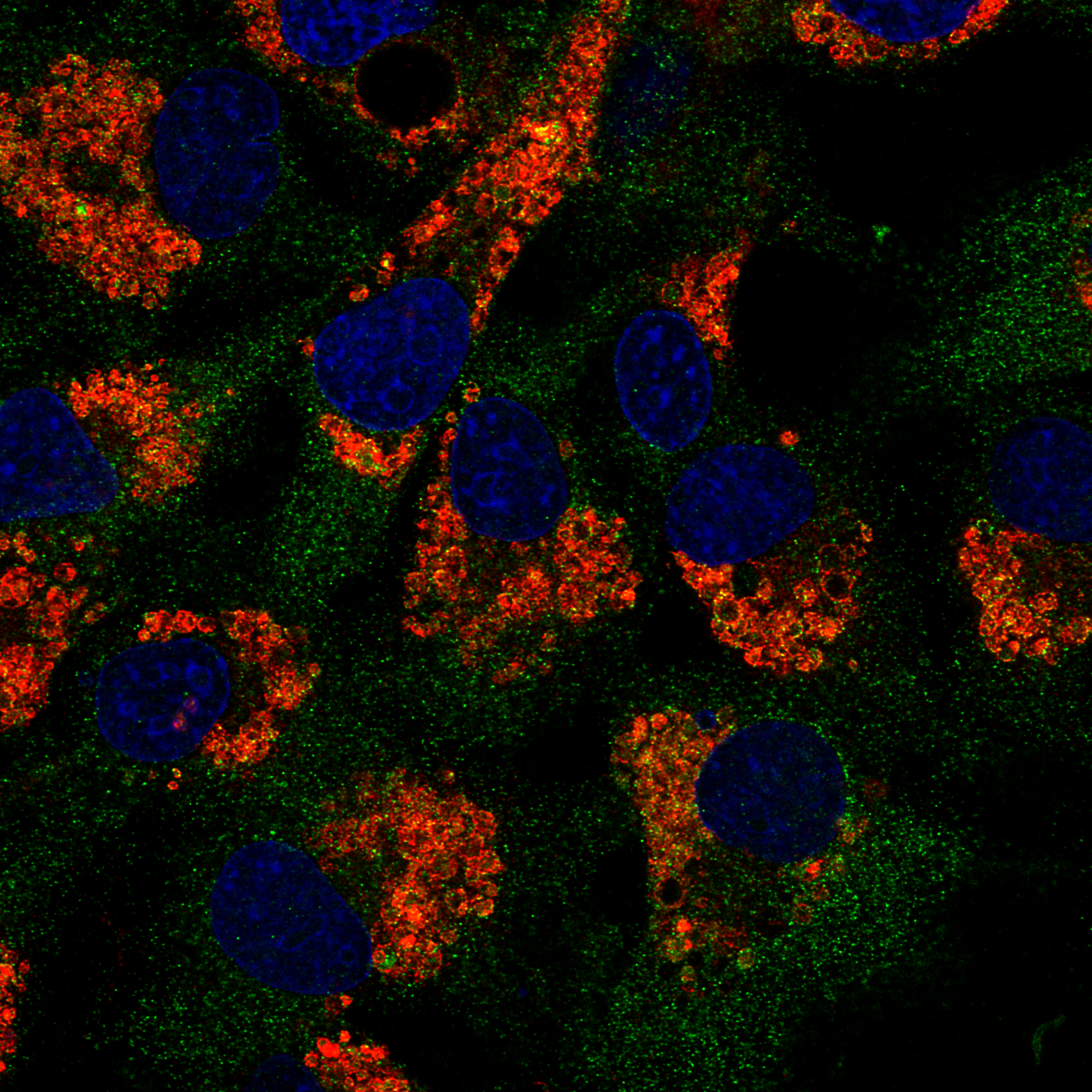

Supplement: Supplementary file 10 — Source data Fig. 5 [file 44319_2026_773_MOESM10_ESM.zip › Figure 5/Figure 5A/IF GNPTABKO mTOR LAMP2 -AA Merge.tif]

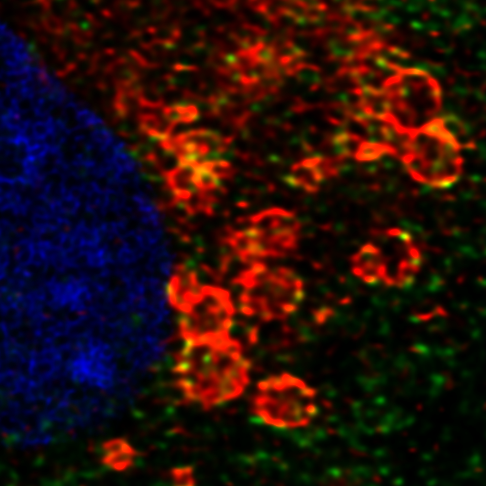

Supplement: Supplementary file 10 — Source data Fig. 5 [file 44319_2026_773_MOESM10_ESM.zip › Figure 5/Figure 5A/IF WT mTOR LAMP2 -AA MERGE inset.tif]

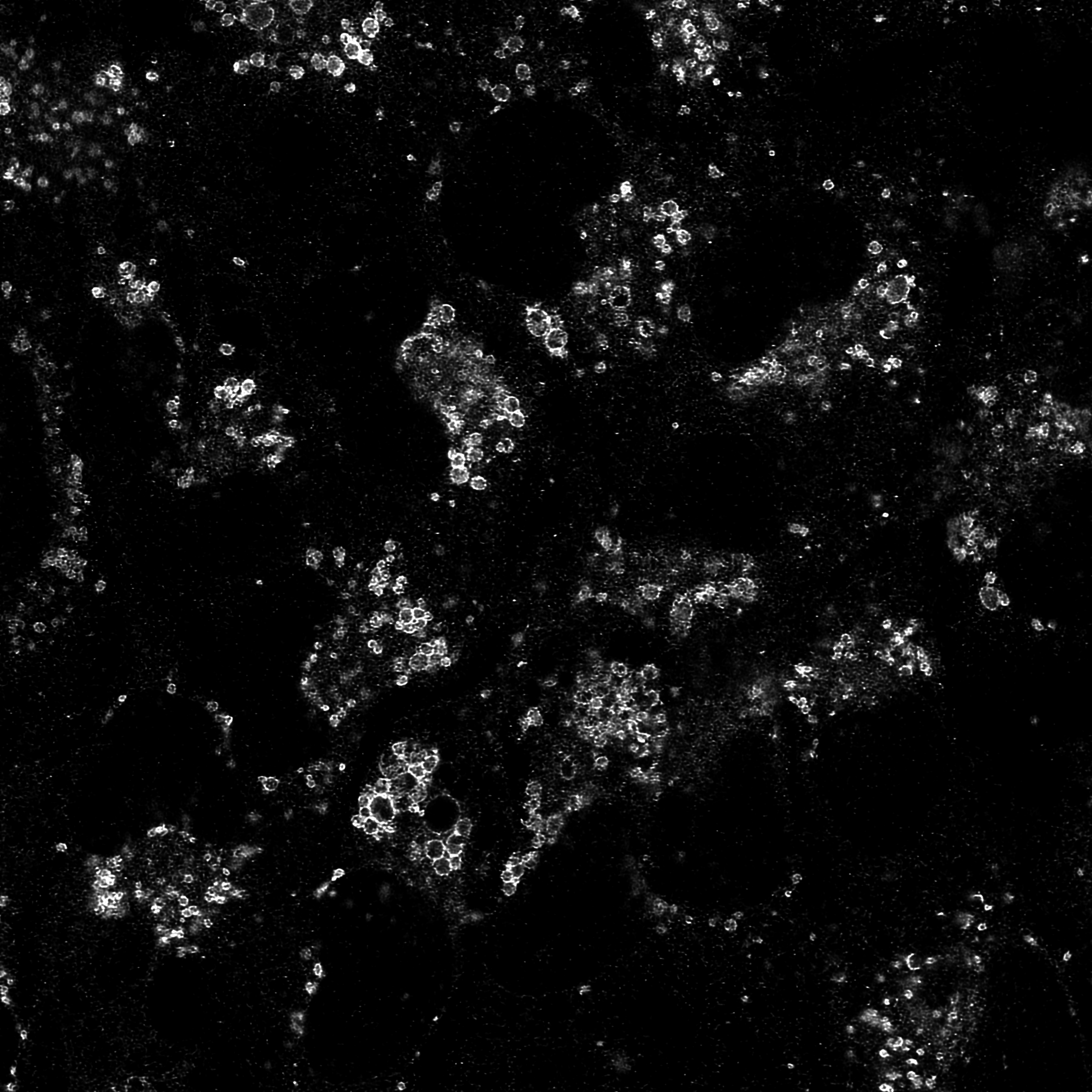

Supplement: Supplementary file 10 — Source data Fig. 5 [file 44319_2026_773_MOESM10_ESM.zip › Figure 5/Figure 5A/IF WT LAMP2 -AA.tif]

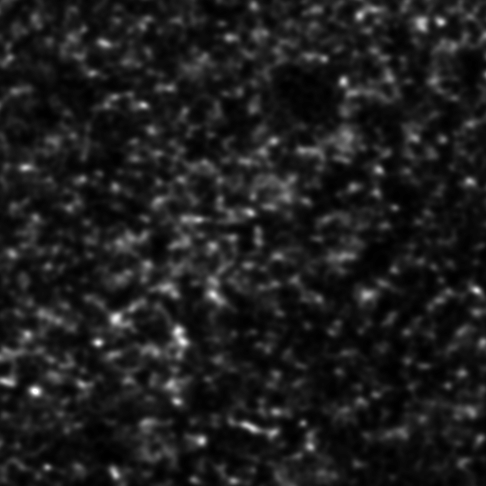

Supplement: Supplementary file 10 — Source data Fig. 5 [file 44319_2026_773_MOESM10_ESM.zip › Figure 5/Figure 5A/IF GRASP55Ko mTOR -AA inset.tif]

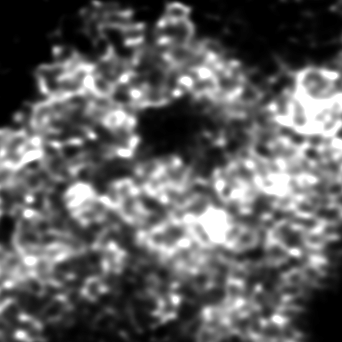

Supplement: Supplementary file 10 — Source data Fig. 5 [file 44319_2026_773_MOESM10_ESM.zip › Figure 5/Figure 5A/IF GNPTABKO LAMP2 +AA inset.tif]

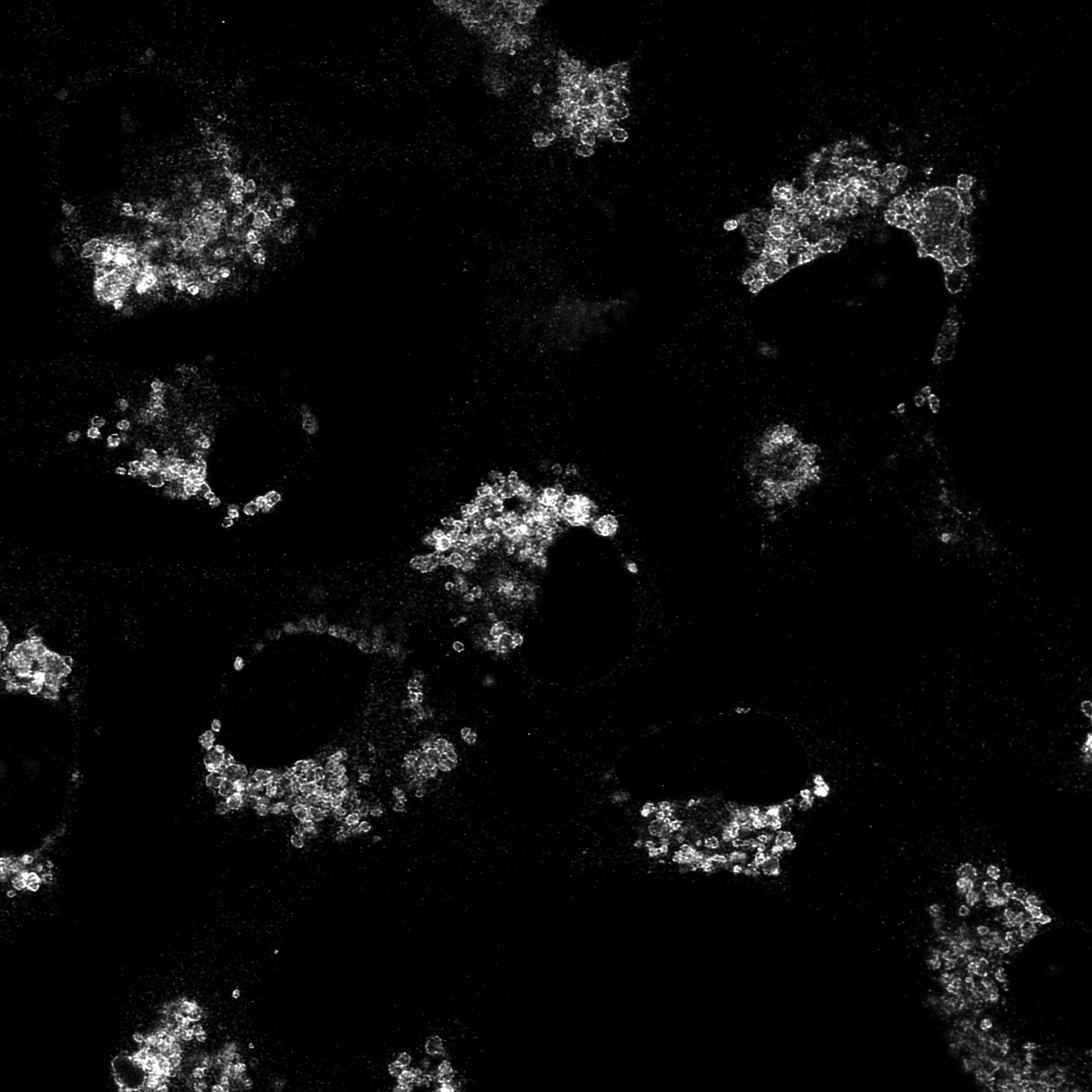

Supplement: Supplementary file 10 — Source data Fig. 5 [file 44319_2026_773_MOESM10_ESM.zip › Figure 5/Figure 5A/IF GNPTABKO LAMP2 +AA .tif]

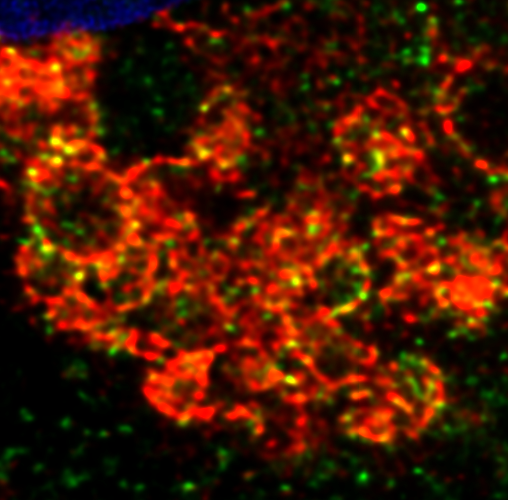

Supplement: Supplementary file 10 — Source data Fig. 5 [file 44319_2026_773_MOESM10_ESM.zip › Figure 5/Figure 5A/IF GNPTABKO mTOR LAMP2 -AA Merge inset.tif]

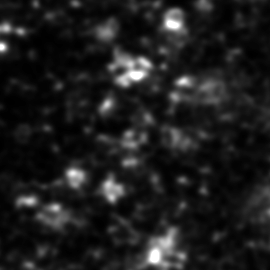

Supplement: Supplementary file 10 — Source data Fig. 5 [file 44319_2026_773_MOESM10_ESM.zip › Figure 5/Figure 5A/IF GRASP65KO mTOR +AA inset.tif]

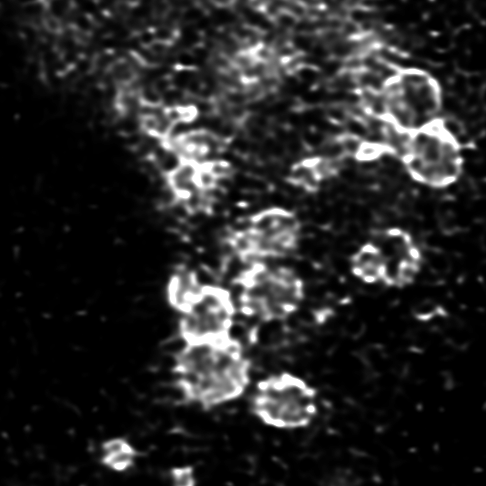

Supplement: Supplementary file 10 — Source data Fig. 5 [file 44319_2026_773_MOESM10_ESM.zip › Figure 5/Figure 5A/IF WT LAMP2 -AA MERGE inset.tif]

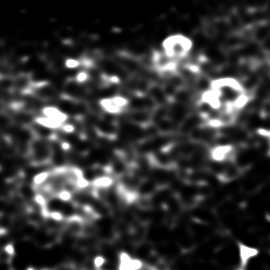

Supplement: Supplementary file 10 — Source data Fig. 5 [file 44319_2026_773_MOESM10_ESM.zip › Figure 5/Figure 5A/IF GRASP55KO LAMP2 +AA inset.tif]

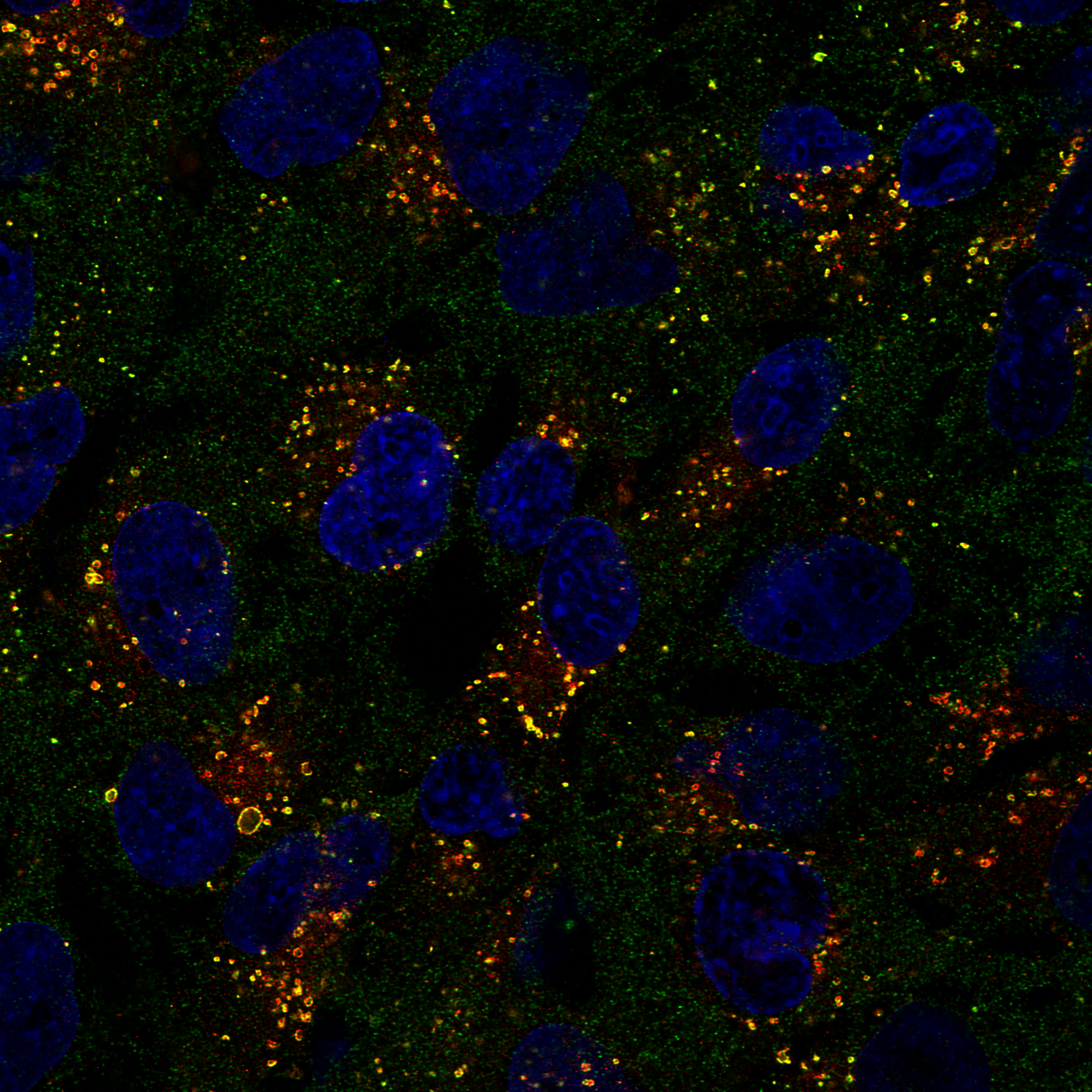

Supplement: Supplementary file 10 — Source data Fig. 5 [file 44319_2026_773_MOESM10_ESM.zip › Figure 5/Figure 5A/IF WT mTOR LAMP2 +AA MERGE.tif]

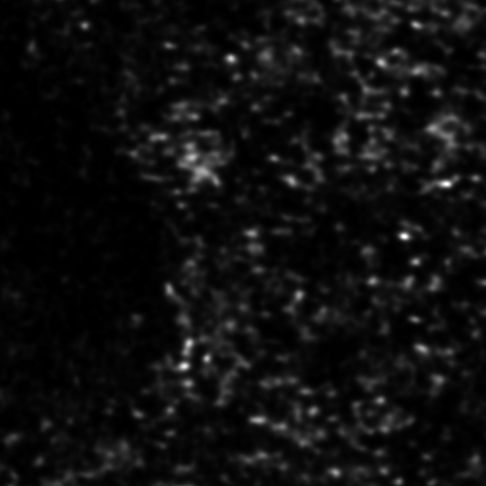

Supplement: Supplementary file 10 — Source data Fig. 5 [file 44319_2026_773_MOESM10_ESM.zip › Figure 5/Figure 5A/IF WT mTOR -AA MERGE inset.tif]

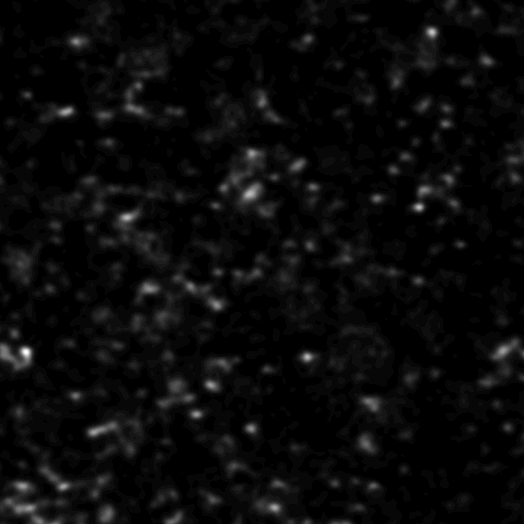

Supplement: Supplementary file 10 — Source data Fig. 5 [file 44319_2026_773_MOESM10_ESM.zip › Figure 5/Figure 5A/IF GRASP65KO mTOR -AA inset .tif]

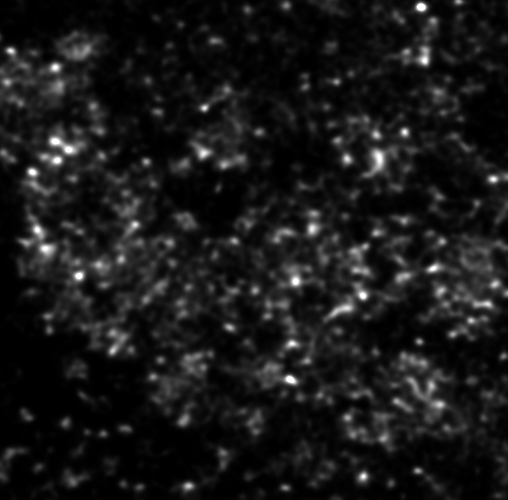

Supplement: Supplementary file 10 — Source data Fig. 5 [file 44319_2026_773_MOESM10_ESM.zip › Figure 5/Figure 5A/IF GNPTABKO mTOR -AA inset.tif]

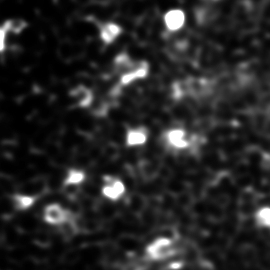

Supplement: Supplementary file 10 — Source data Fig. 5 [file 44319_2026_773_MOESM10_ESM.zip › Figure 5/Figure 5A/IF GRASP65KO LAMP2 +AA inset.tif]

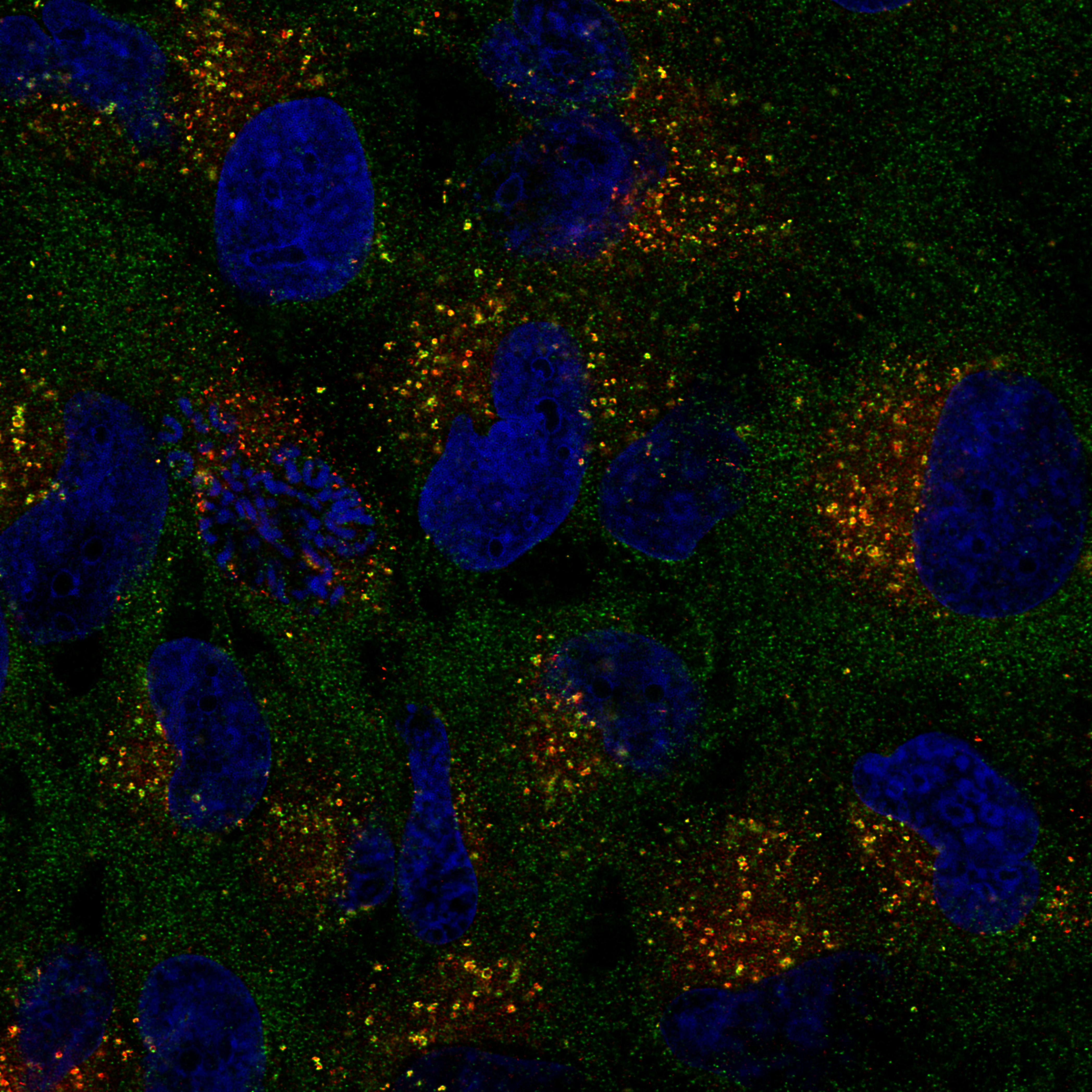

Supplement: Supplementary file 10 — Source data Fig. 5 [file 44319_2026_773_MOESM10_ESM.zip › Figure 5/Figure 5A/IF GRASP65KO mTOR LAMP2 +AA Merge.tif]

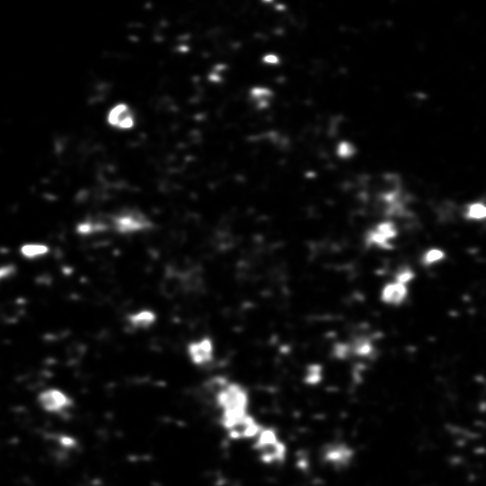

Supplement: Supplementary file 10 — Source data Fig. 5 [file 44319_2026_773_MOESM10_ESM.zip › Figure 5/Figure 5A/IF WT mTOR +AA inset.tif]

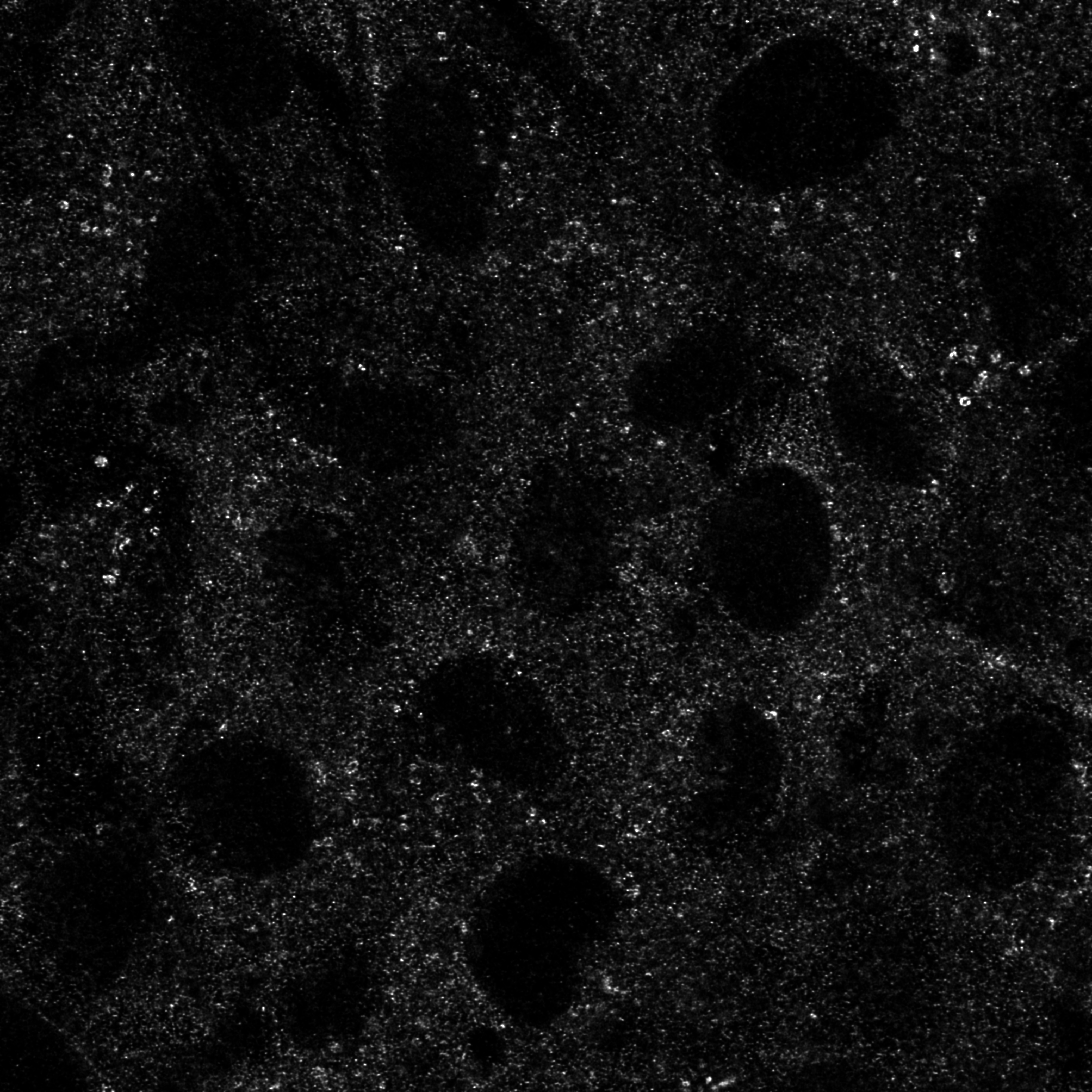

Supplement: Supplementary file 10 — Source data Fig. 5 [file 44319_2026_773_MOESM10_ESM.zip › Figure 5/Figure 5A/IF GRASP55KO mTOR +AA.tif]

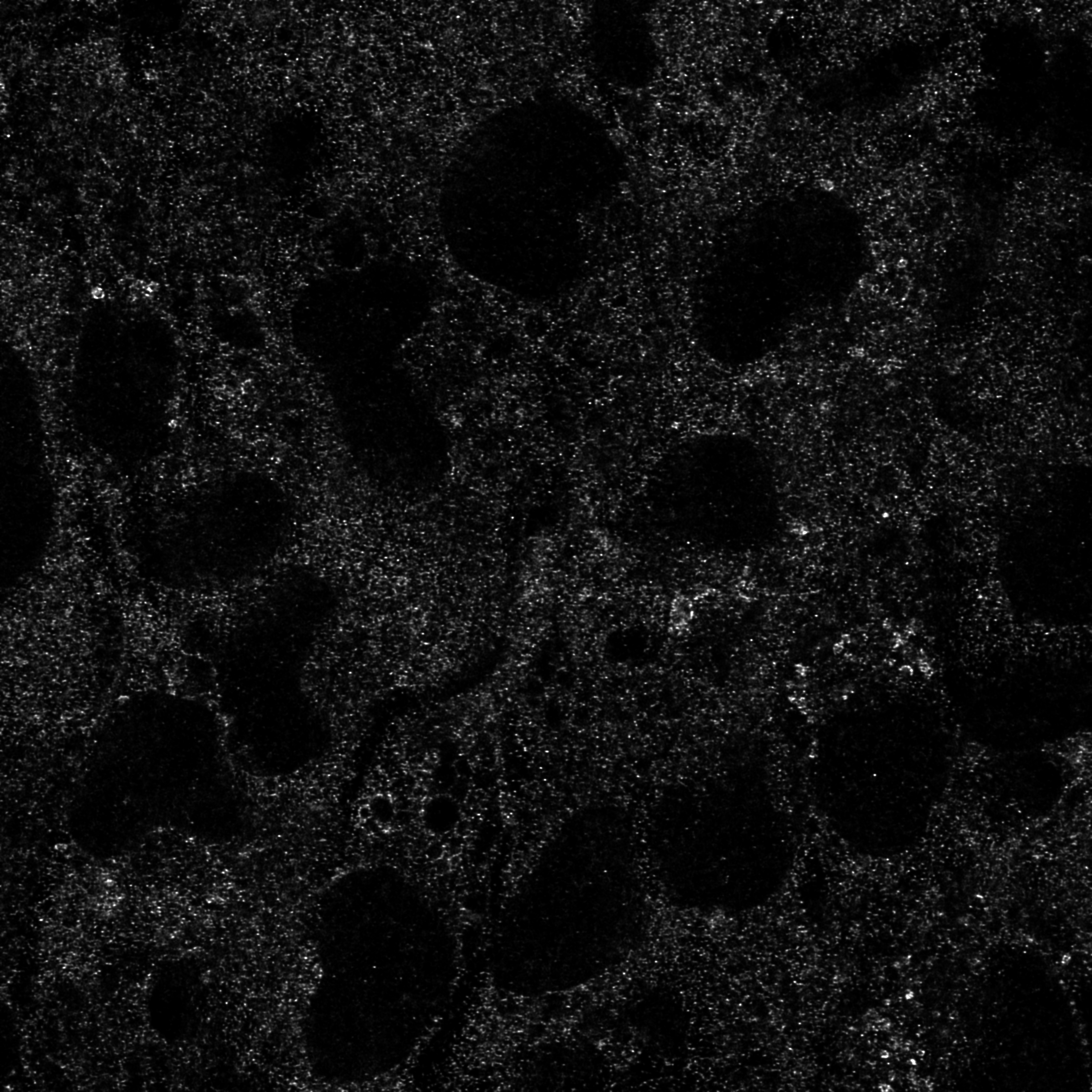

Supplement: Supplementary file 10 — Source data Fig. 5 [file 44319_2026_773_MOESM10_ESM.zip › Figure 5/Figure 5A/IF WT mTOR -AA.tif]

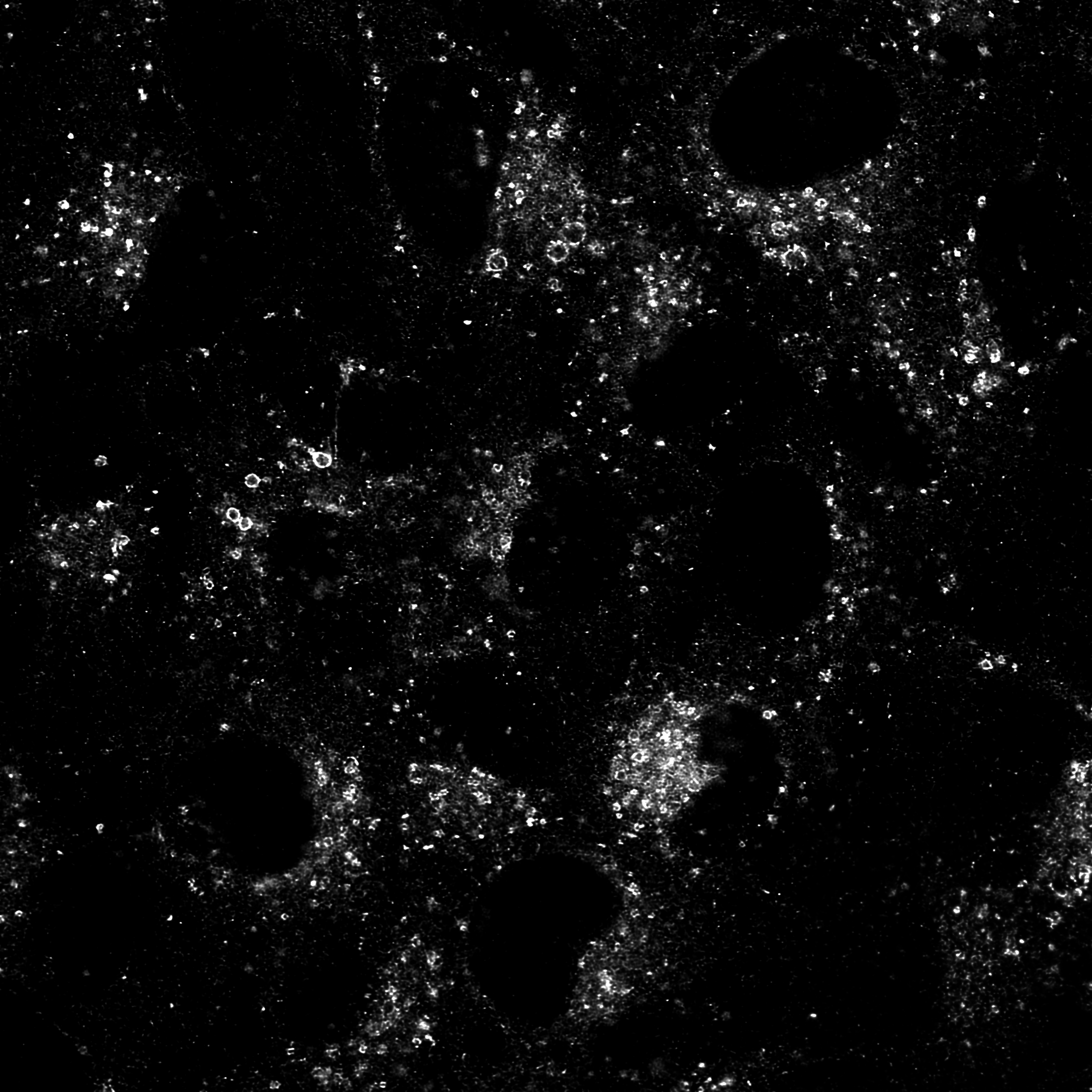

Supplement: Supplementary file 10 — Source data Fig. 5 [file 44319_2026_773_MOESM10_ESM.zip › Figure 5/Figure 5A/IF GRASP55KO LAMP2 +AA.tif]

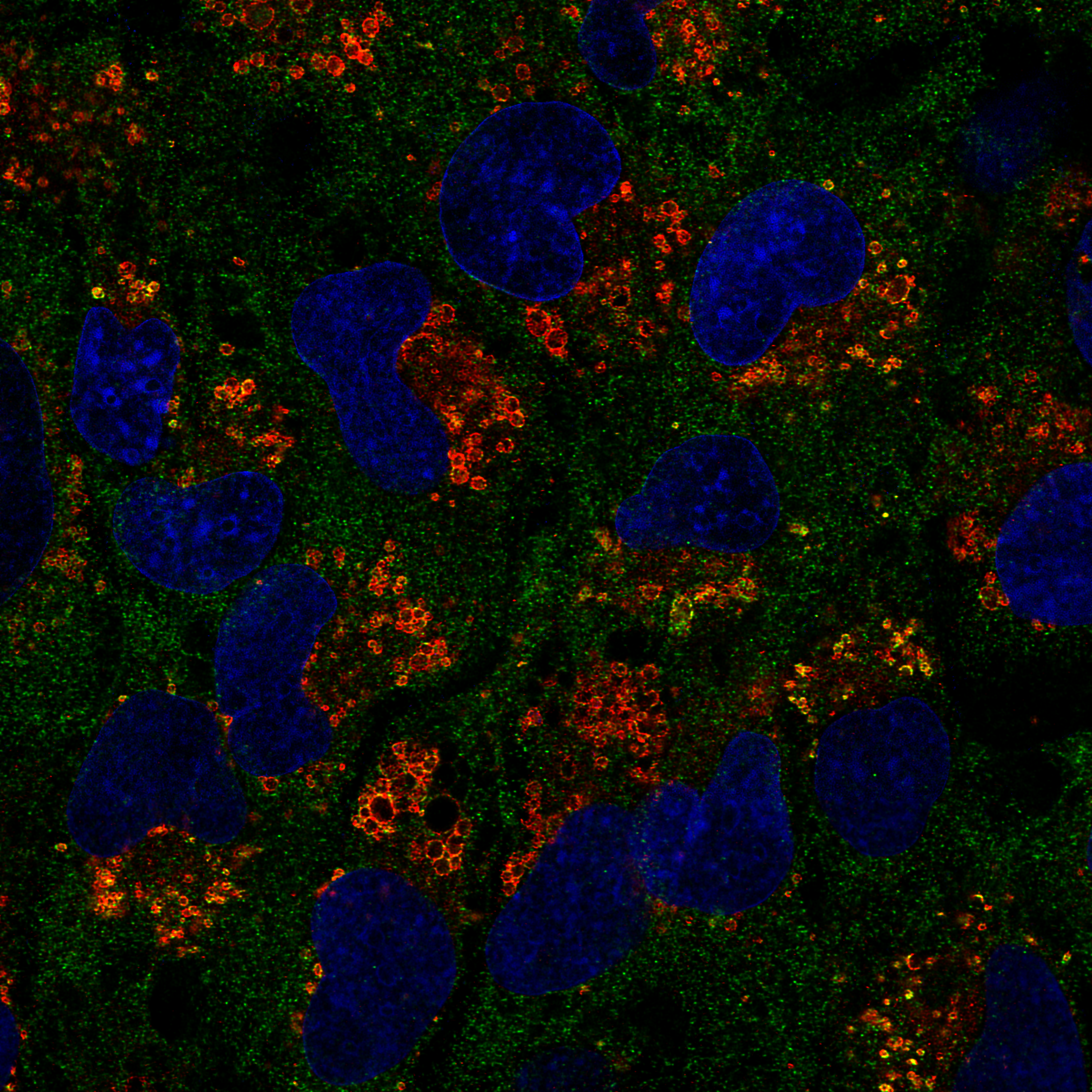

Supplement: Supplementary file 10 — Source data Fig. 5 [file 44319_2026_773_MOESM10_ESM.zip › Figure 5/Figure 5A/IF WT mTOR LAMP2 -AA MERGE.tif]

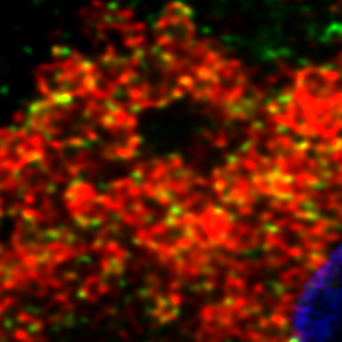

Supplement: Supplementary file 10 — Source data Fig. 5 [file 44319_2026_773_MOESM10_ESM.zip › Figure 5/Figure 5A/IF GNPTABKO mTOR LAMP2 +AA Merge inset.tif]

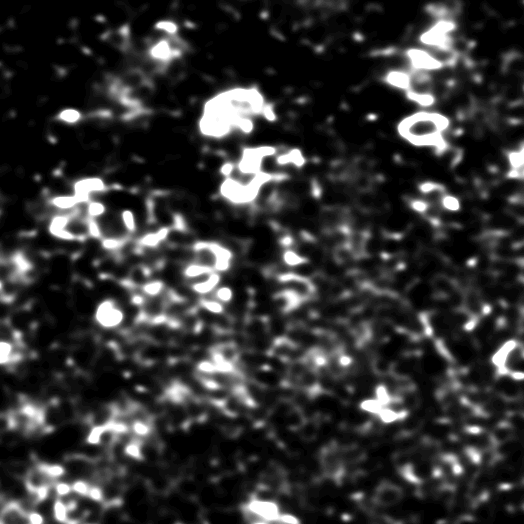

Supplement: Supplementary file 10 — Source data Fig. 5 [file 44319_2026_773_MOESM10_ESM.zip › Figure 5/Figure 5A/IF GRASP65KO LAMP2 -AA inset.tif]

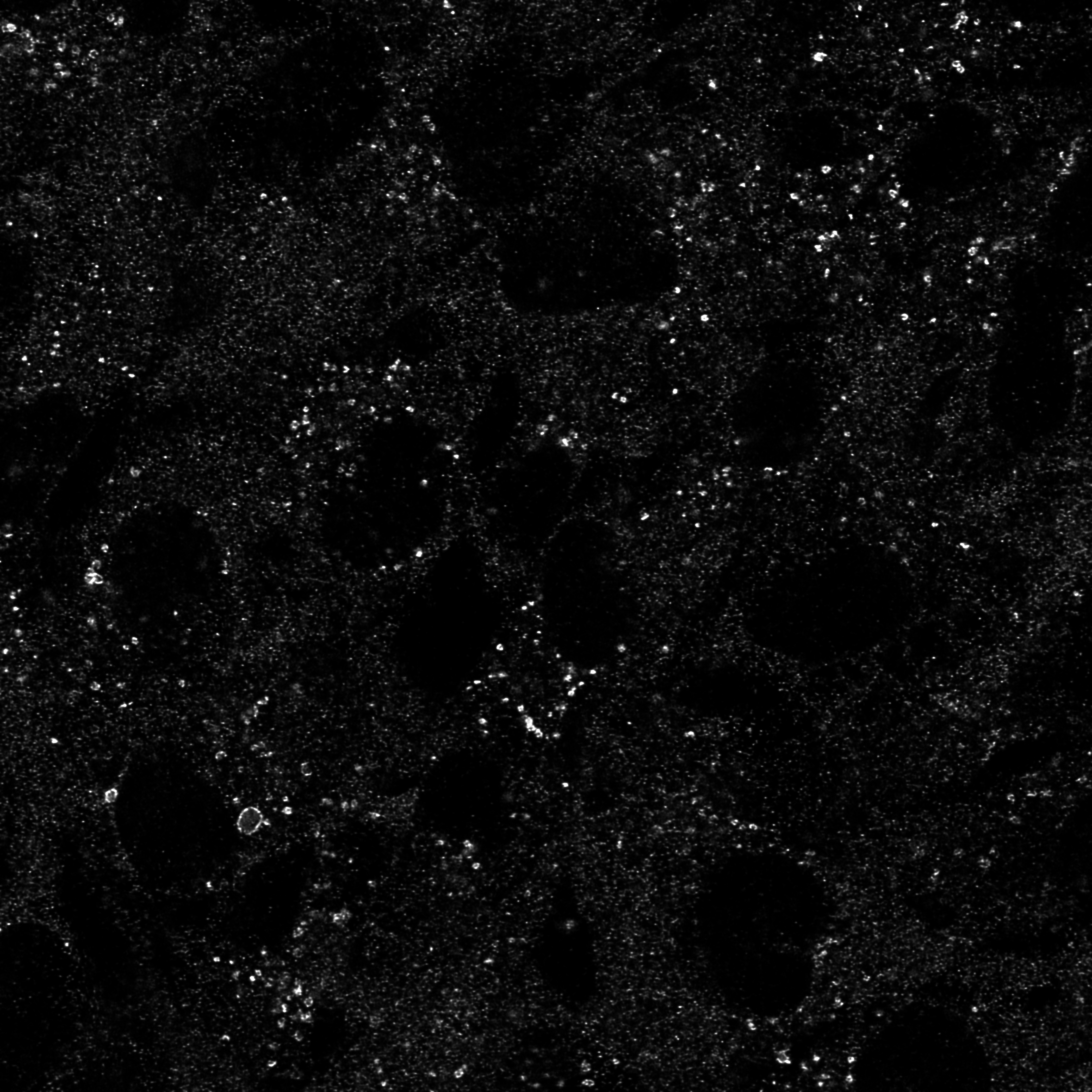

Supplement: Supplementary file 10 — Source data Fig. 5 [file 44319_2026_773_MOESM10_ESM.zip › Figure 5/Figure 5A/IF WT mTOR +AA.tif]

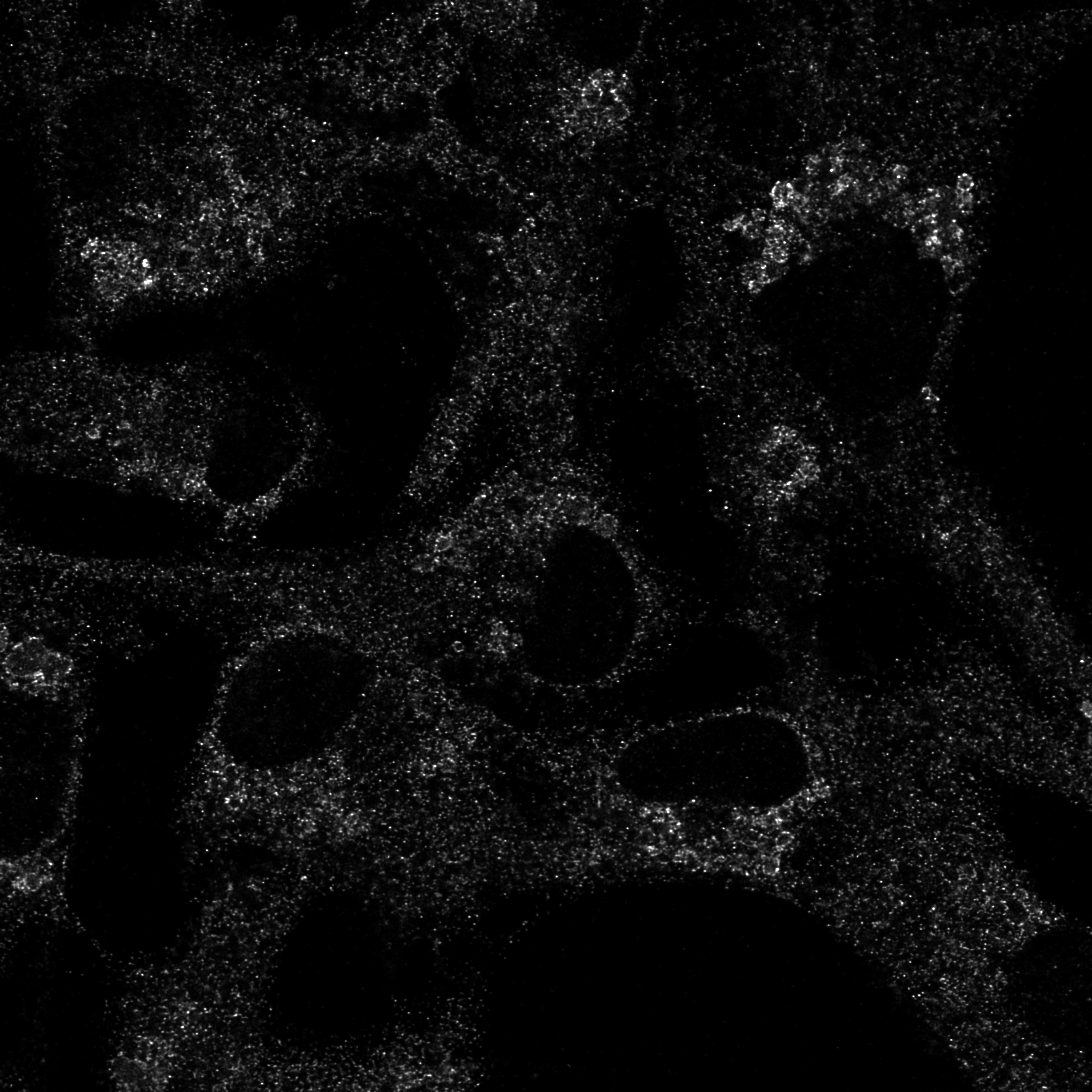

Supplement: Supplementary file 10 — Source data Fig. 5 [file 44319_2026_773_MOESM10_ESM.zip › Figure 5/Figure 5A/IF GNPTABKO mTOR +AA .tif]

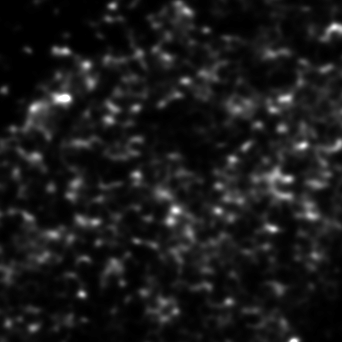

Supplement: Supplementary file 10 — Source data Fig. 5 [file 44319_2026_773_MOESM10_ESM.zip › Figure 5/Figure 5A/IF GNPTABKO mTOR +AA inset.tif]

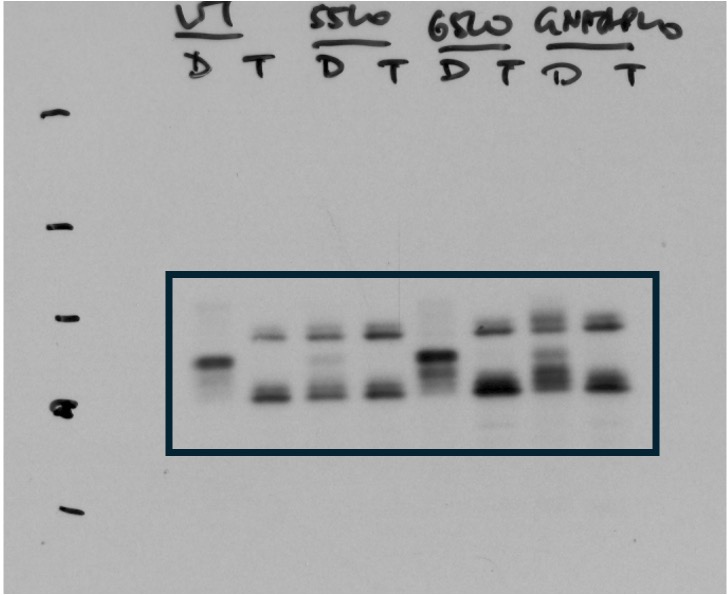

Supplement: Supplementary file 10 — Source data Fig. 5 [file 44319_2026_773_MOESM10_ESM.zip › Figure 5/Figure 5C/Western TFE3.tif]

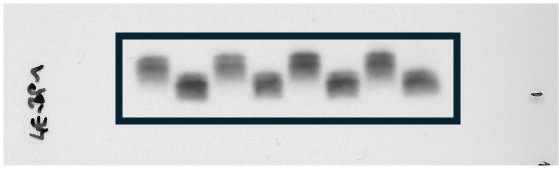

Supplement: Supplementary file 10 — Source data Fig. 5 [file 44319_2026_773_MOESM10_ESM.zip › Figure 5/Figure 5C/Western 4E-BP1.tif]
