# Supplementary figures and images for: GRASP55 maintains lysosome function by controlling sorting of lysosomal enzymes at the Golgi (part 3 of 5)
Source: EMBO Rep. 2026 Apr 16;27(11):2947–72. doi: 10.1038/s44319-026-00773-w (PMC13261057; doi:10.1038/s44319-026-00773-w)

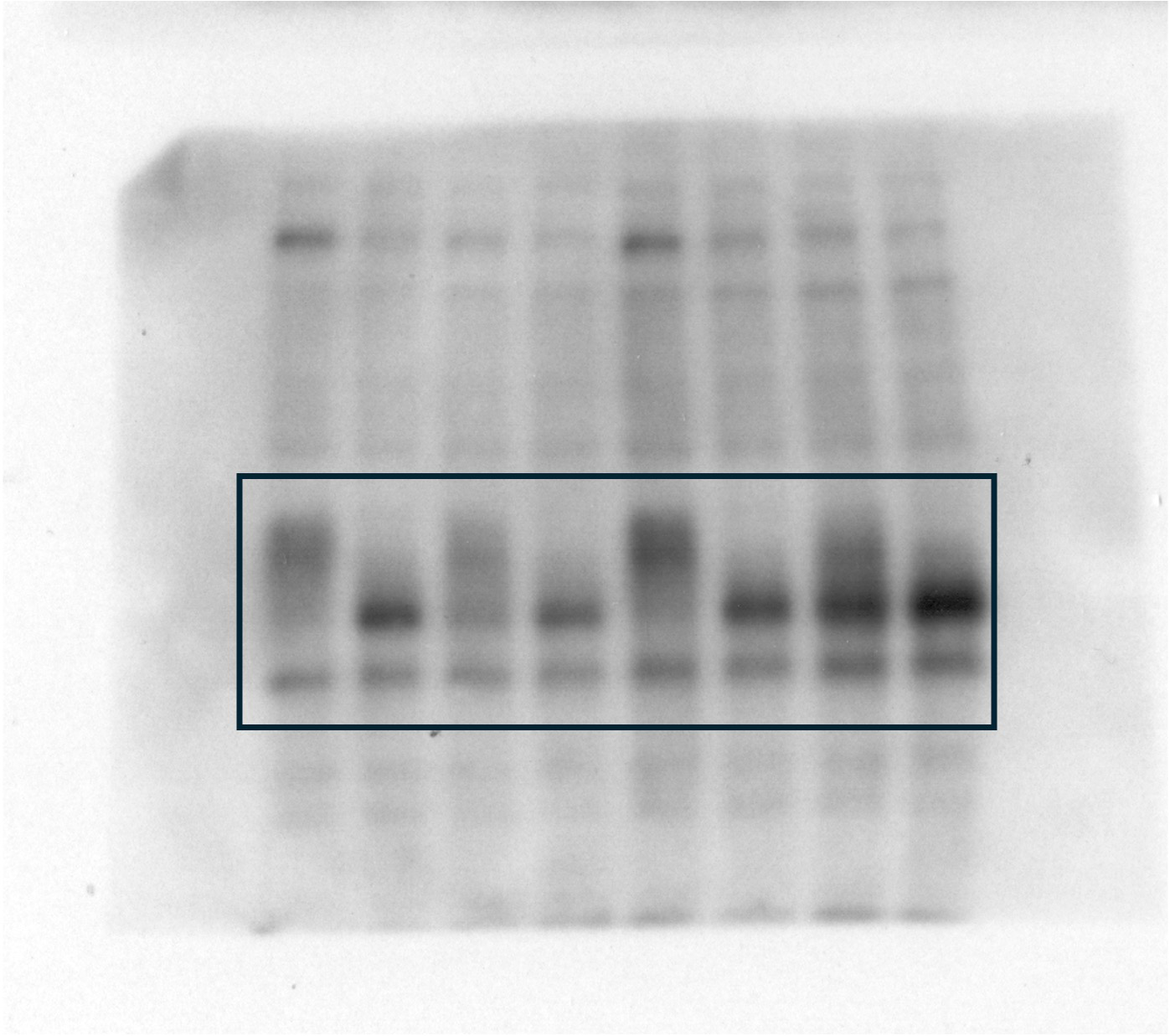

Supplement: Supplementary file 10 — Source data Fig. 5 [file 44319_2026_773_MOESM10_ESM.zip › Figure 5/Figure 5C/Western TFEB.tif]

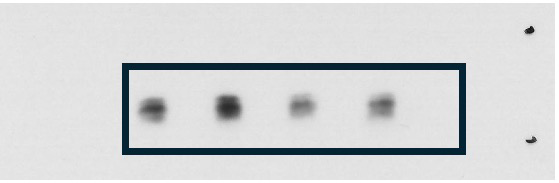

Supplement: Supplementary file 10 — Source data Fig. 5 [file 44319_2026_773_MOESM10_ESM.zip › Figure 5/Figure 5C/Western phospho-4E-BP1.tif]

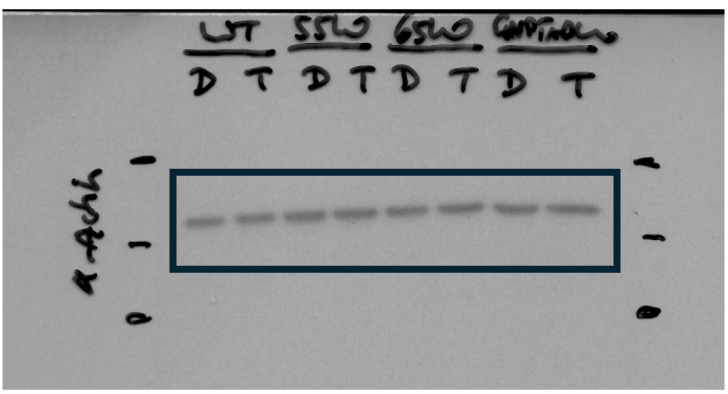

Supplement: Supplementary file 10 — Source data Fig. 5 [file 44319_2026_773_MOESM10_ESM.zip › Figure 5/Figure 5C/Western ACTIN.tif]

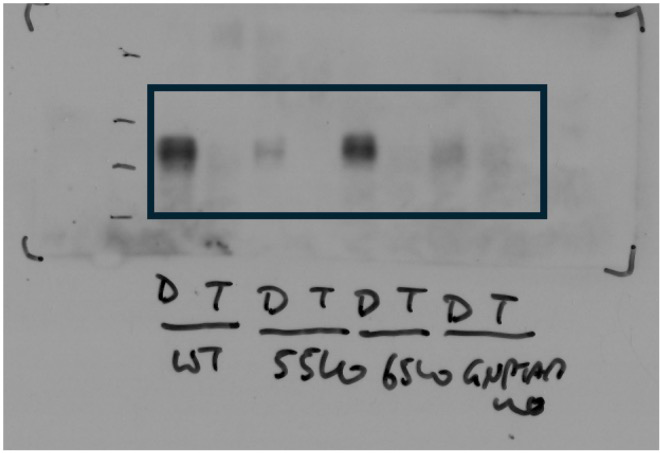

Supplement: Supplementary file 10 — Source data Fig. 5 [file 44319_2026_773_MOESM10_ESM.zip › Figure 5/Figure 5C/Western phospho-TFEB.tif]

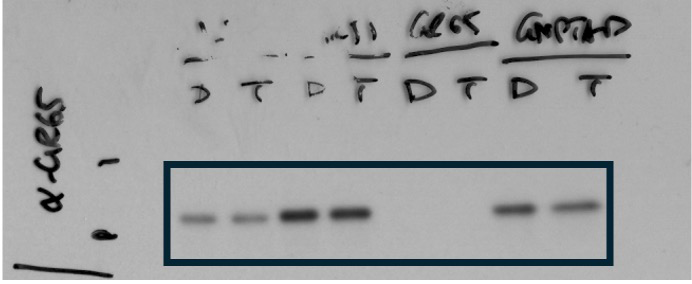

Supplement: Supplementary file 10 — Source data Fig. 5 [file 44319_2026_773_MOESM10_ESM.zip › Figure 5/Figure 5C/Western GRASP65.tif]

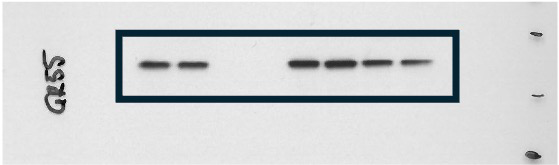

Supplement: Supplementary file 10 — Source data Fig. 5 [file 44319_2026_773_MOESM10_ESM.zip › Figure 5/Figure 5C/Western GRASP55.tif]

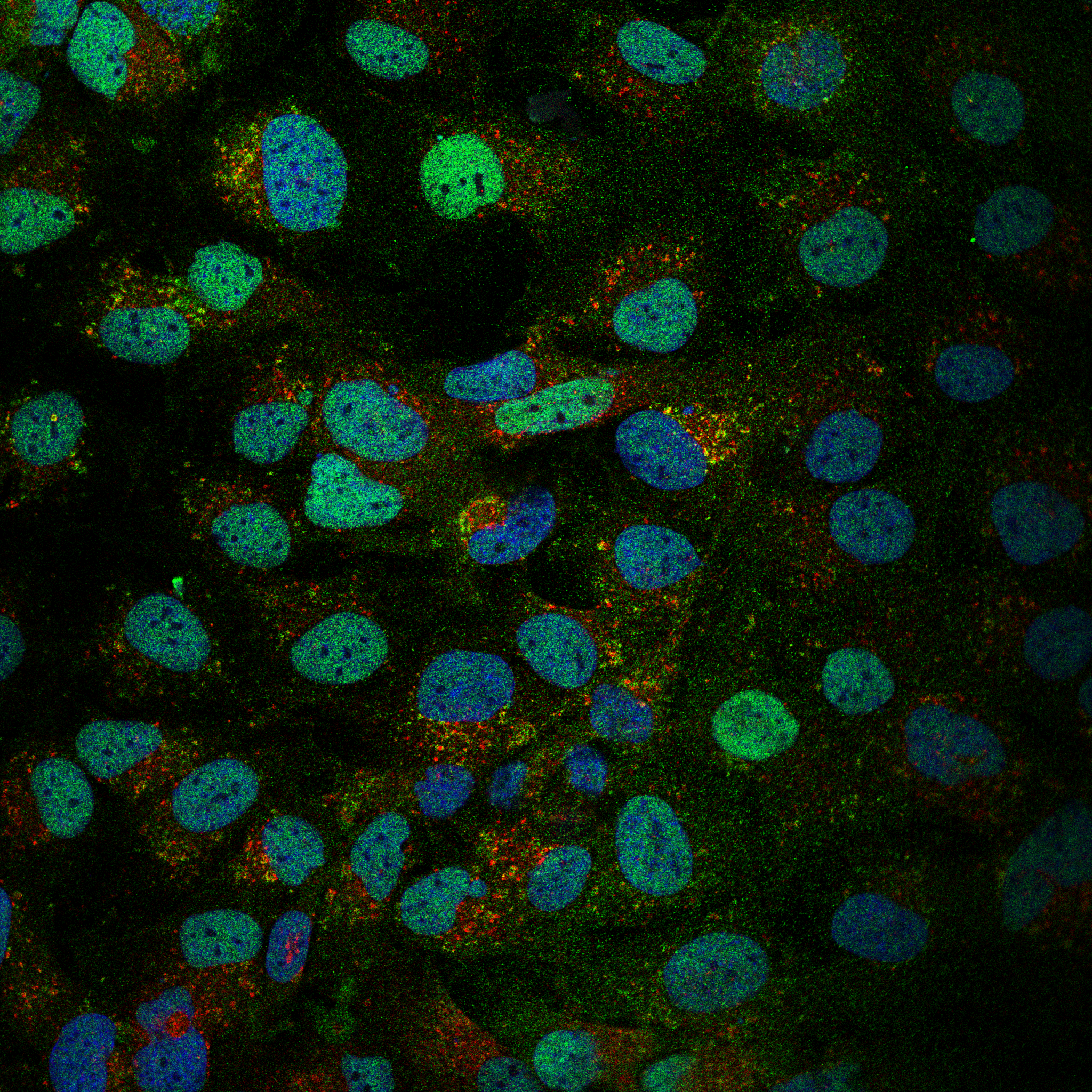

Supplement: Supplementary file 10 — Source data Fig. 5 [file 44319_2026_773_MOESM10_ESM.zip › Figure 5/Figure 5E/IF GRASP55KO Torin TFE3_LAMP2 MERGE.tif]

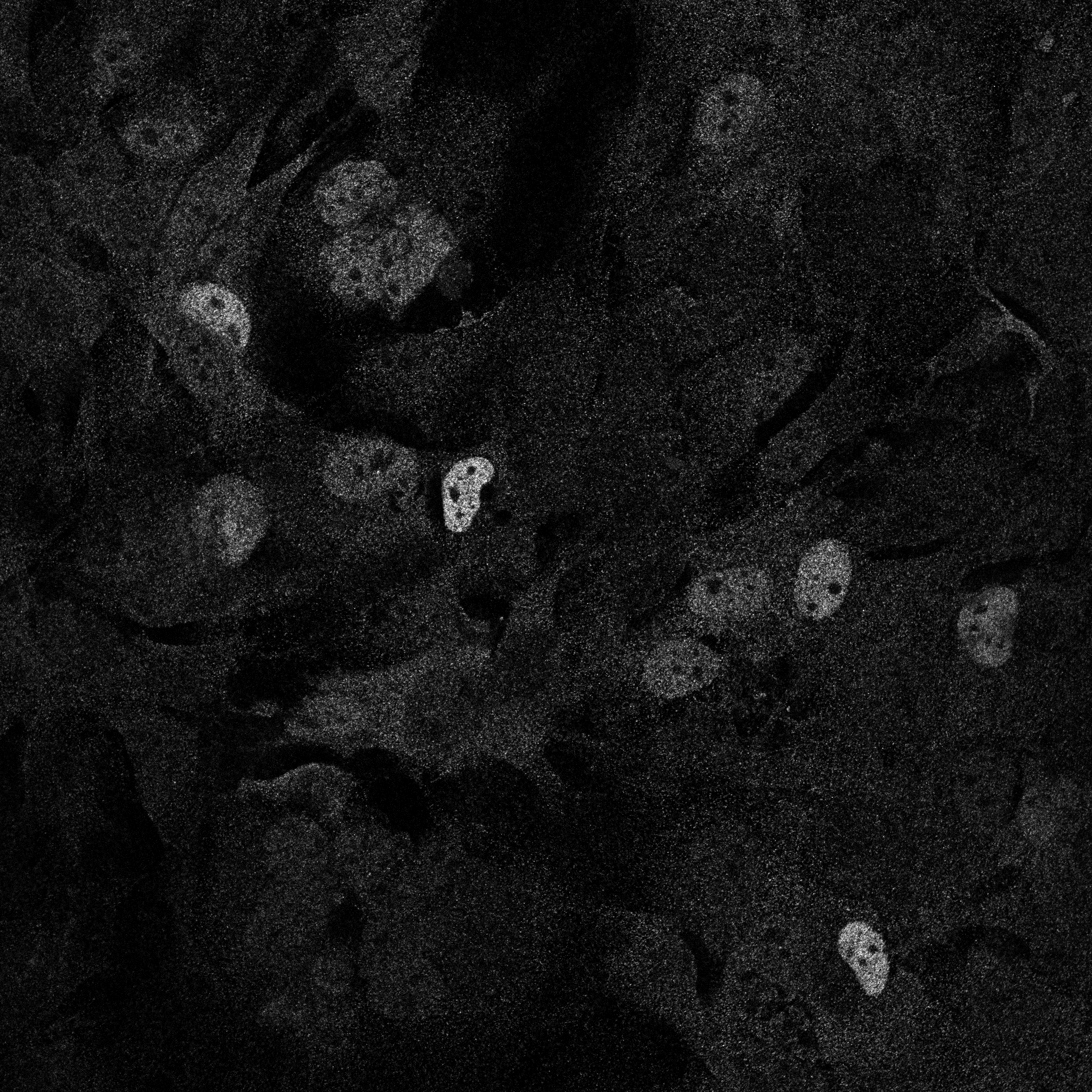

Supplement: Supplementary file 10 — Source data Fig. 5 [file 44319_2026_773_MOESM10_ESM.zip › Figure 5/Figure 5E/IF GRASP55KO DMSO TFE3.tif]

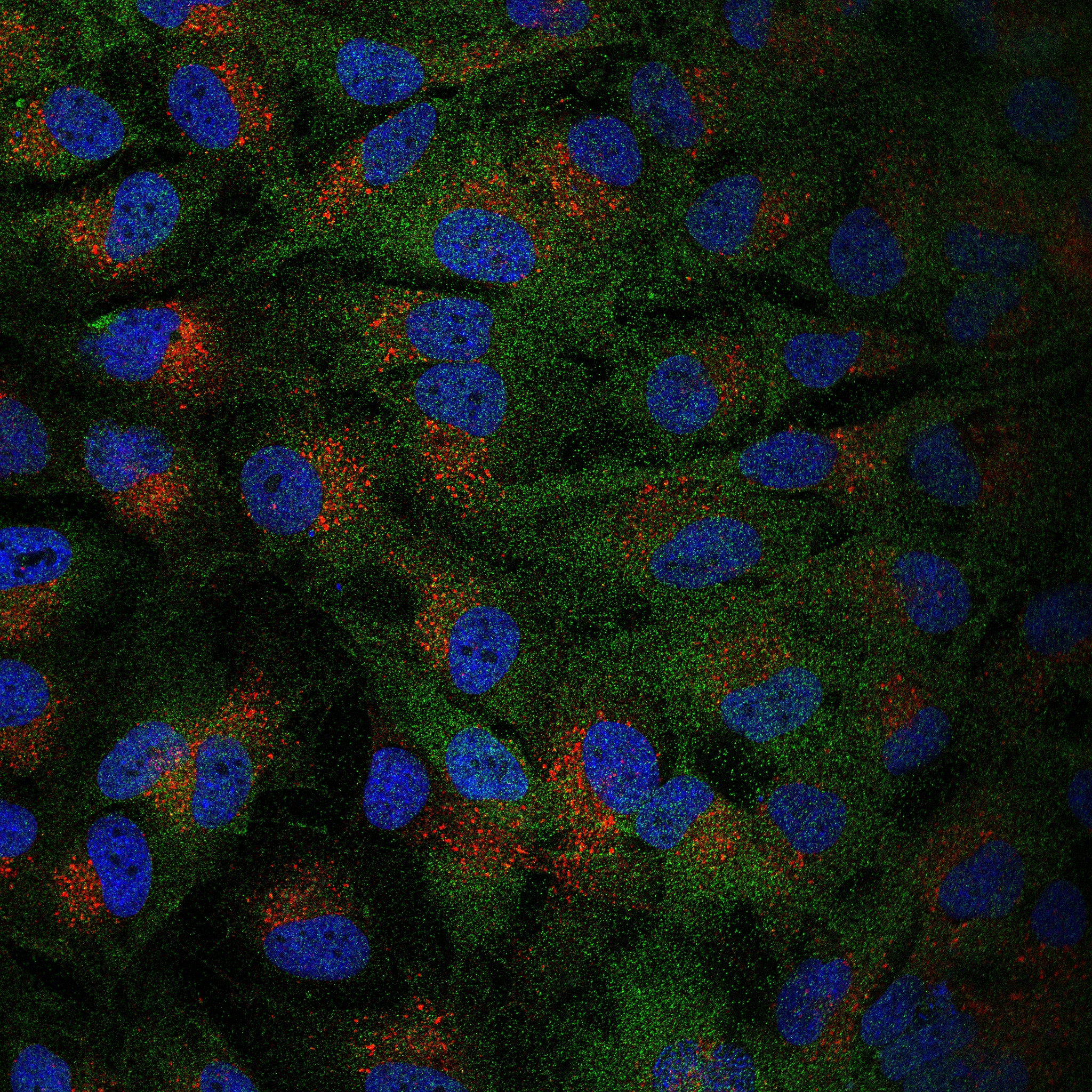

Supplement: Supplementary file 10 — Source data Fig. 5 [file 44319_2026_773_MOESM10_ESM.zip › Figure 5/Figure 5E/IF WT DMSO TFE3_LAMP2 MERGE.tif]

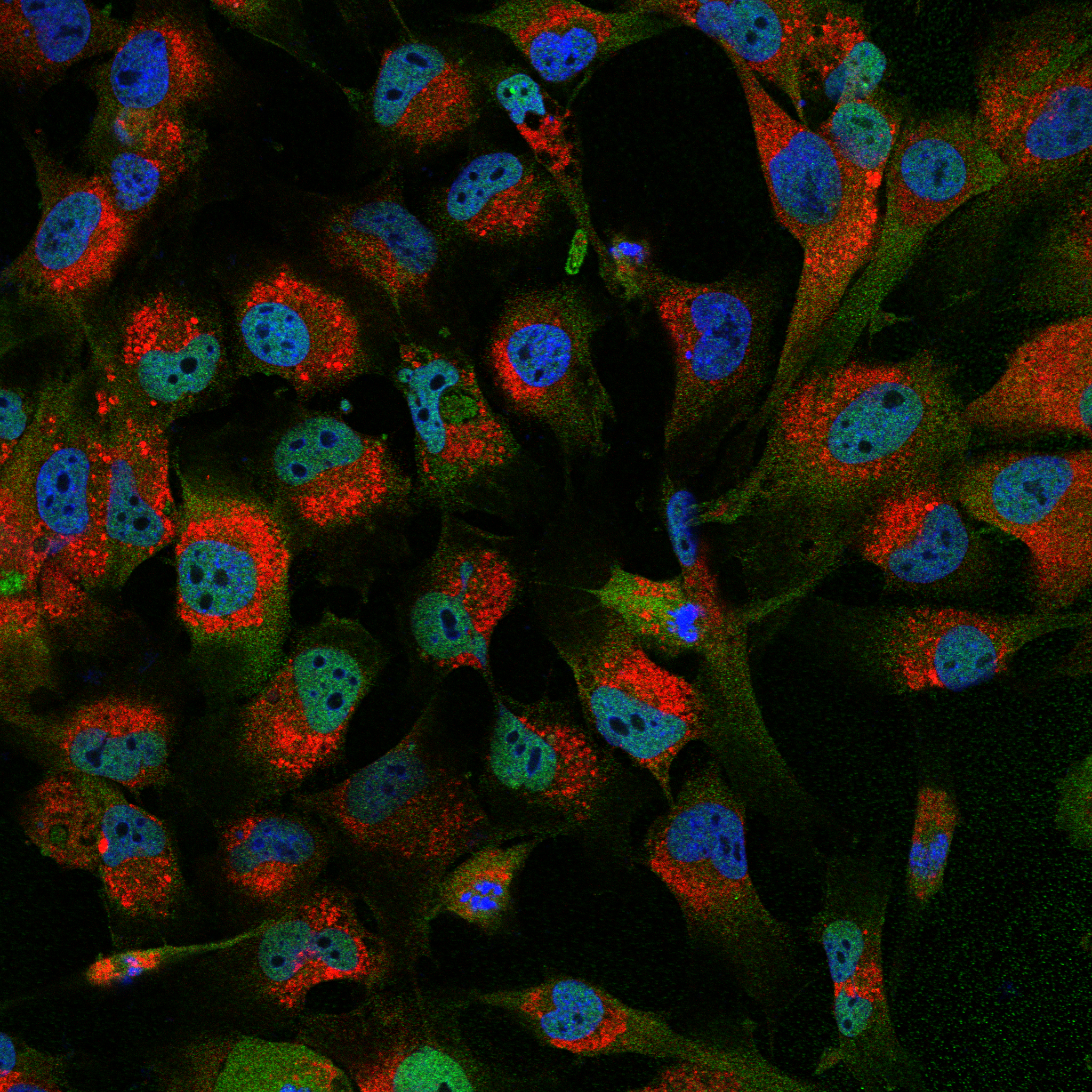

Supplement: Supplementary file 10 — Source data Fig. 5 [file 44319_2026_773_MOESM10_ESM.zip › Figure 5/Figure 5E/IF GNPTABKO DMSO TFE3_LAMP2 MERGE.tif]

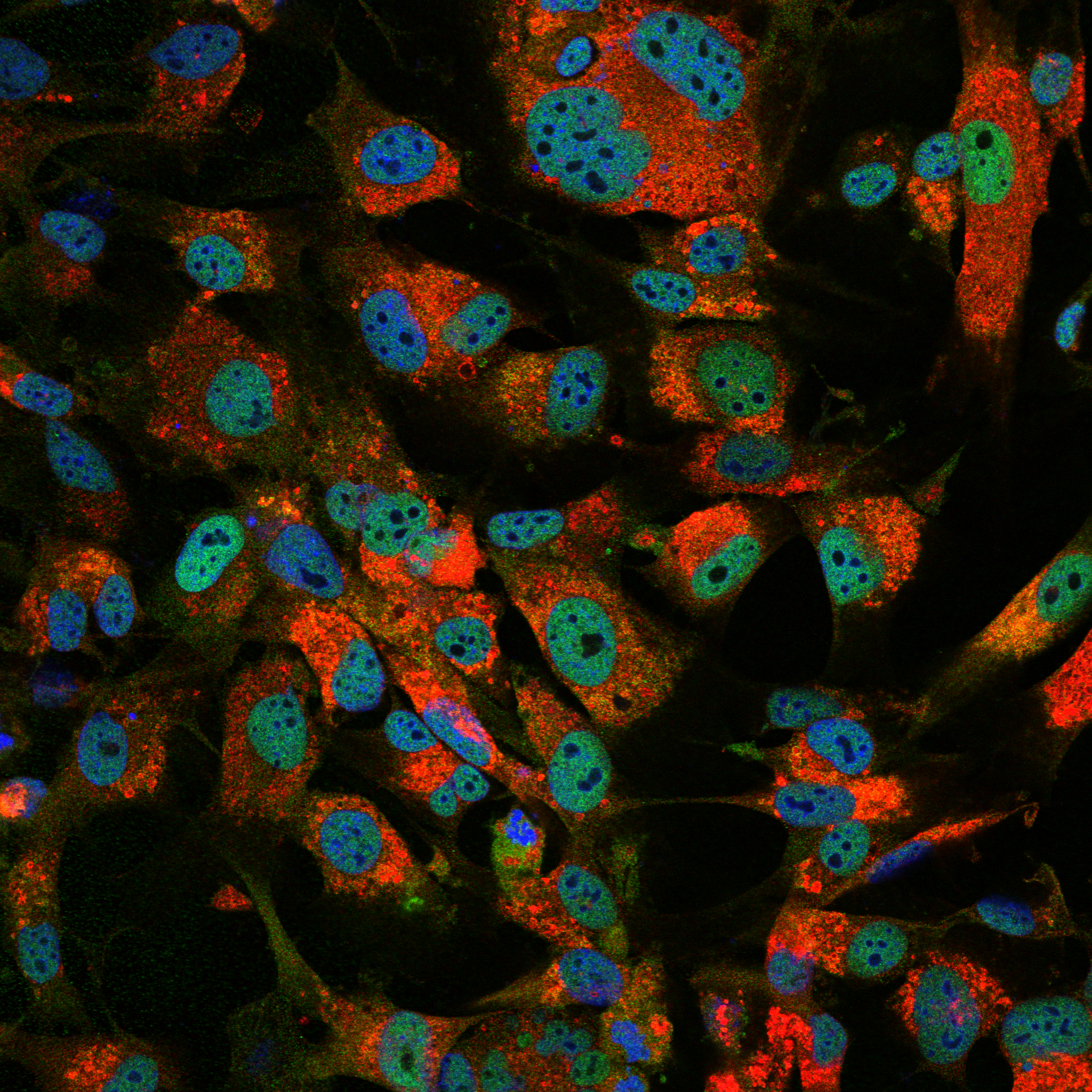

Supplement: Supplementary file 10 — Source data Fig. 5 [file 44319_2026_773_MOESM10_ESM.zip › Figure 5/Figure 5E/IF GNPTABKO Torin TFE3_LAMP2 MERGE.tif]

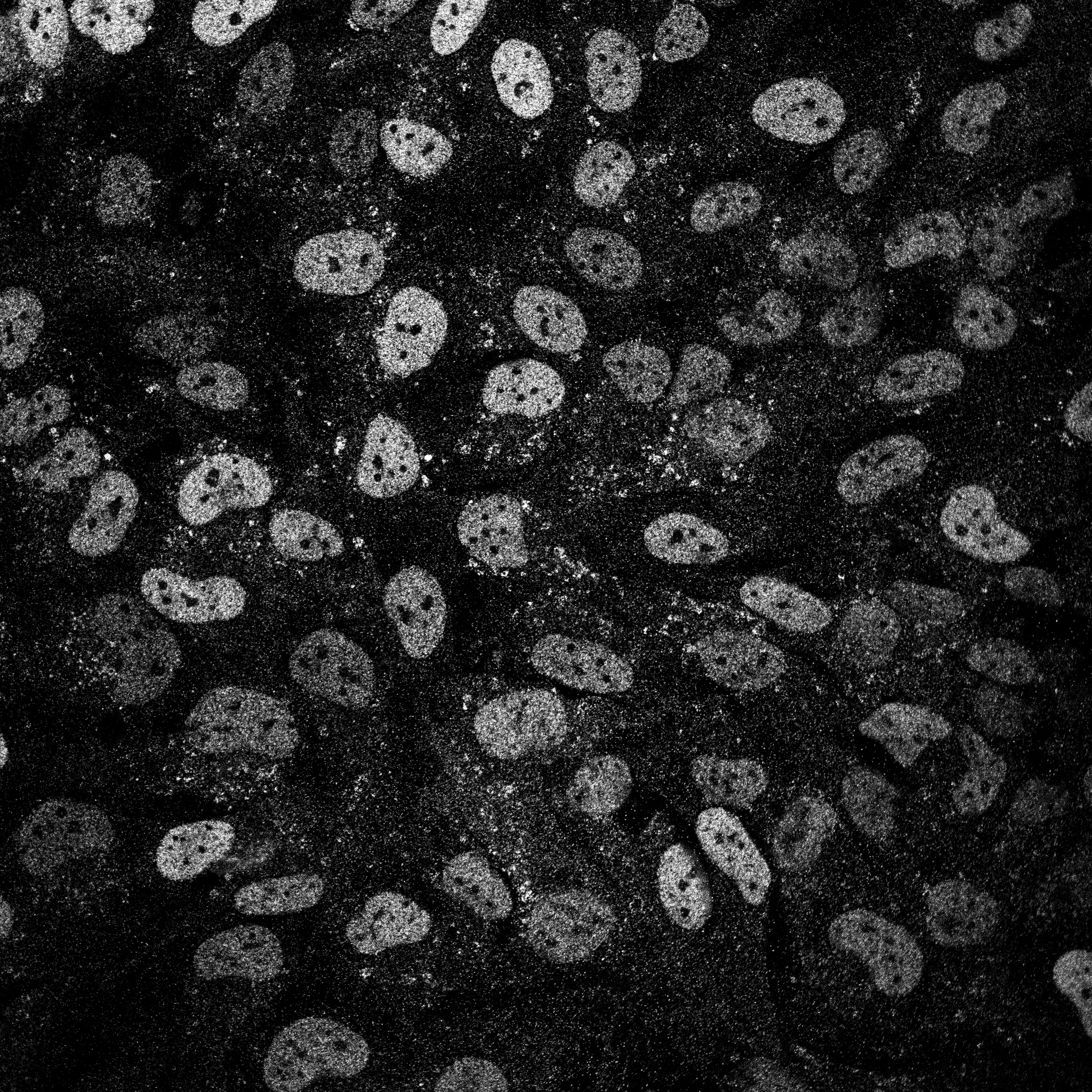

Supplement: Supplementary file 10 — Source data Fig. 5 [file 44319_2026_773_MOESM10_ESM.zip › Figure 5/Figure 5E/IF WT Torin TFE3.tif]

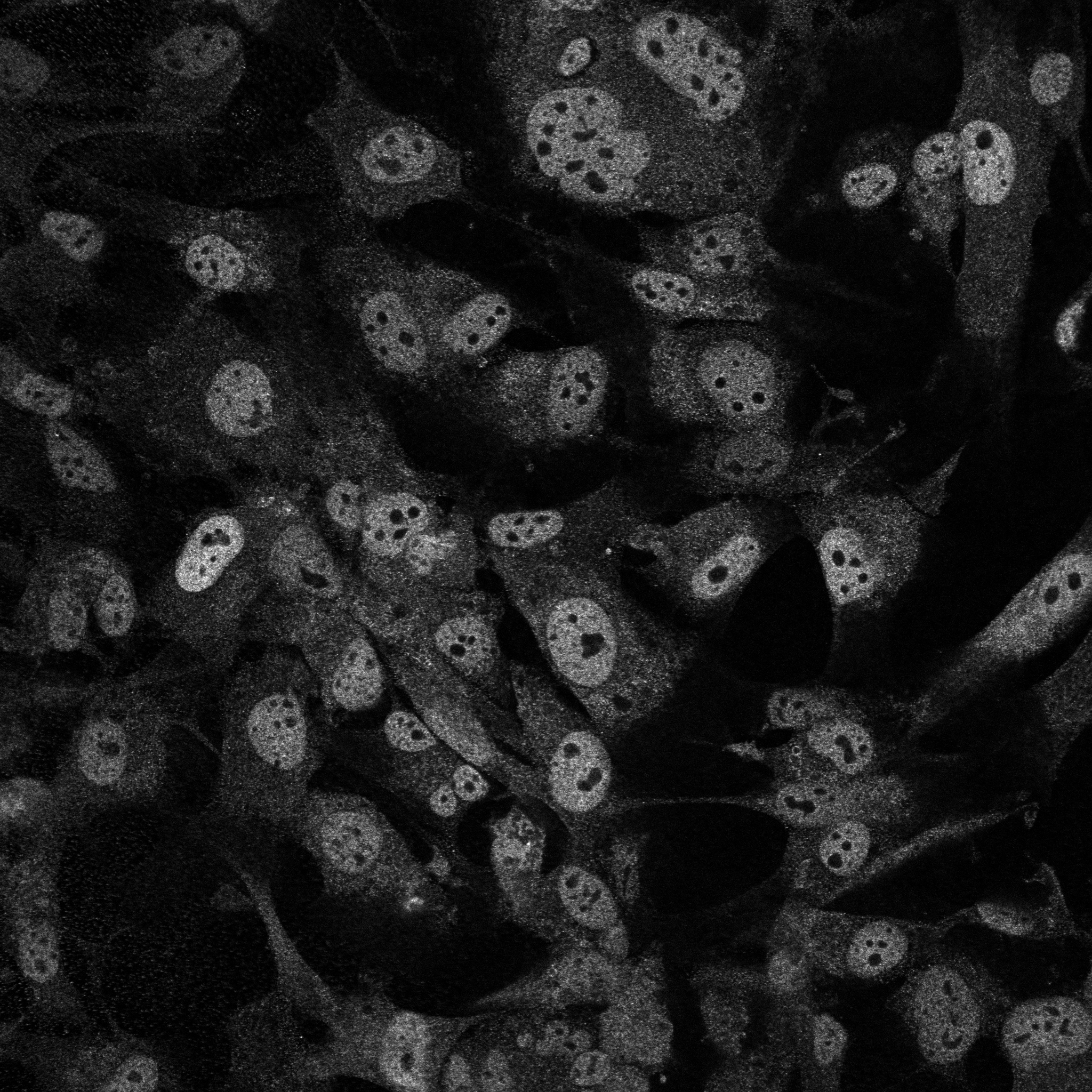

Supplement: Supplementary file 10 — Source data Fig. 5 [file 44319_2026_773_MOESM10_ESM.zip › Figure 5/Figure 5E/IF GNPTABKO Torin TFE3.tif]

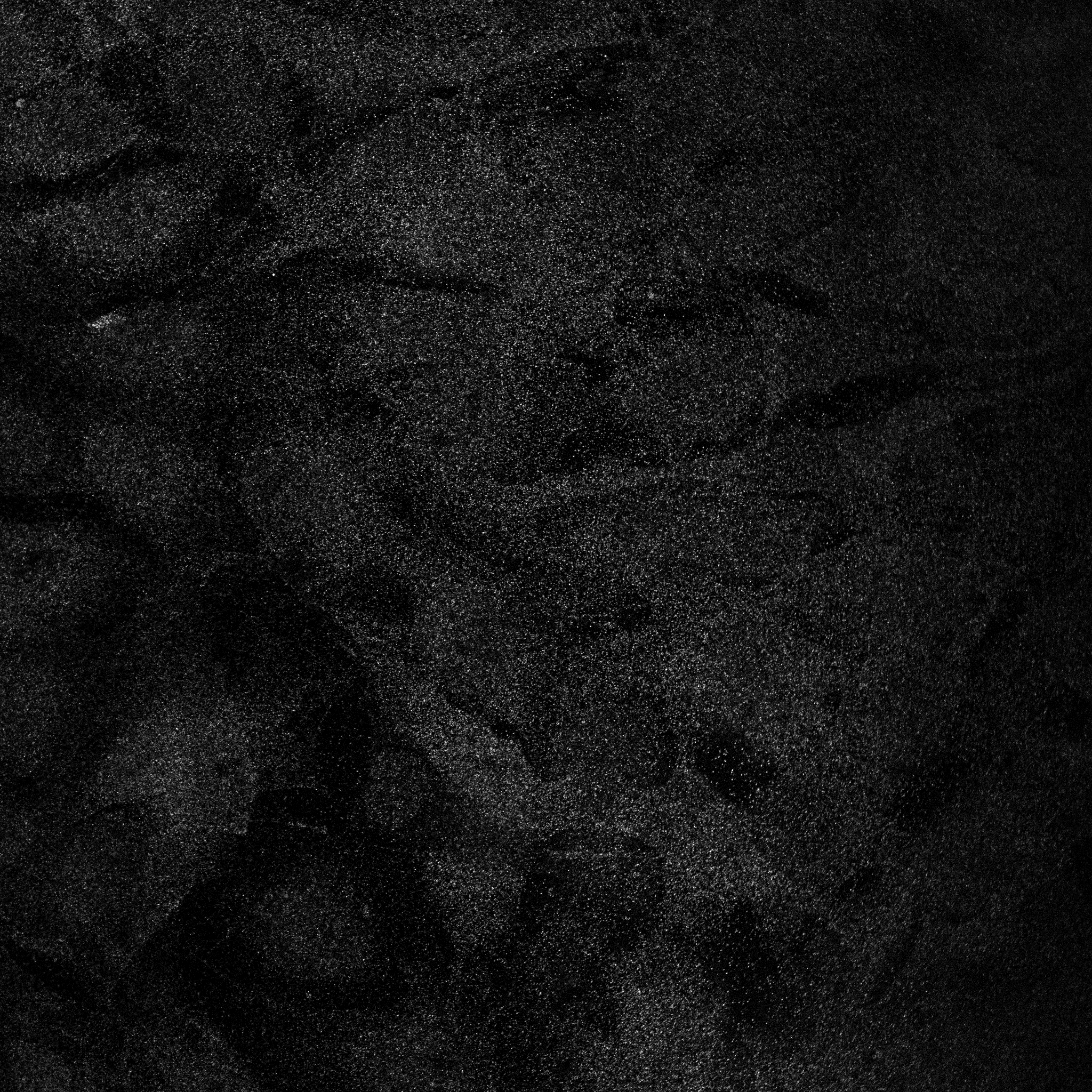

Supplement: Supplementary file 10 — Source data Fig. 5 [file 44319_2026_773_MOESM10_ESM.zip › Figure 5/Figure 5E/IF WT DMSO TFE3.tif]

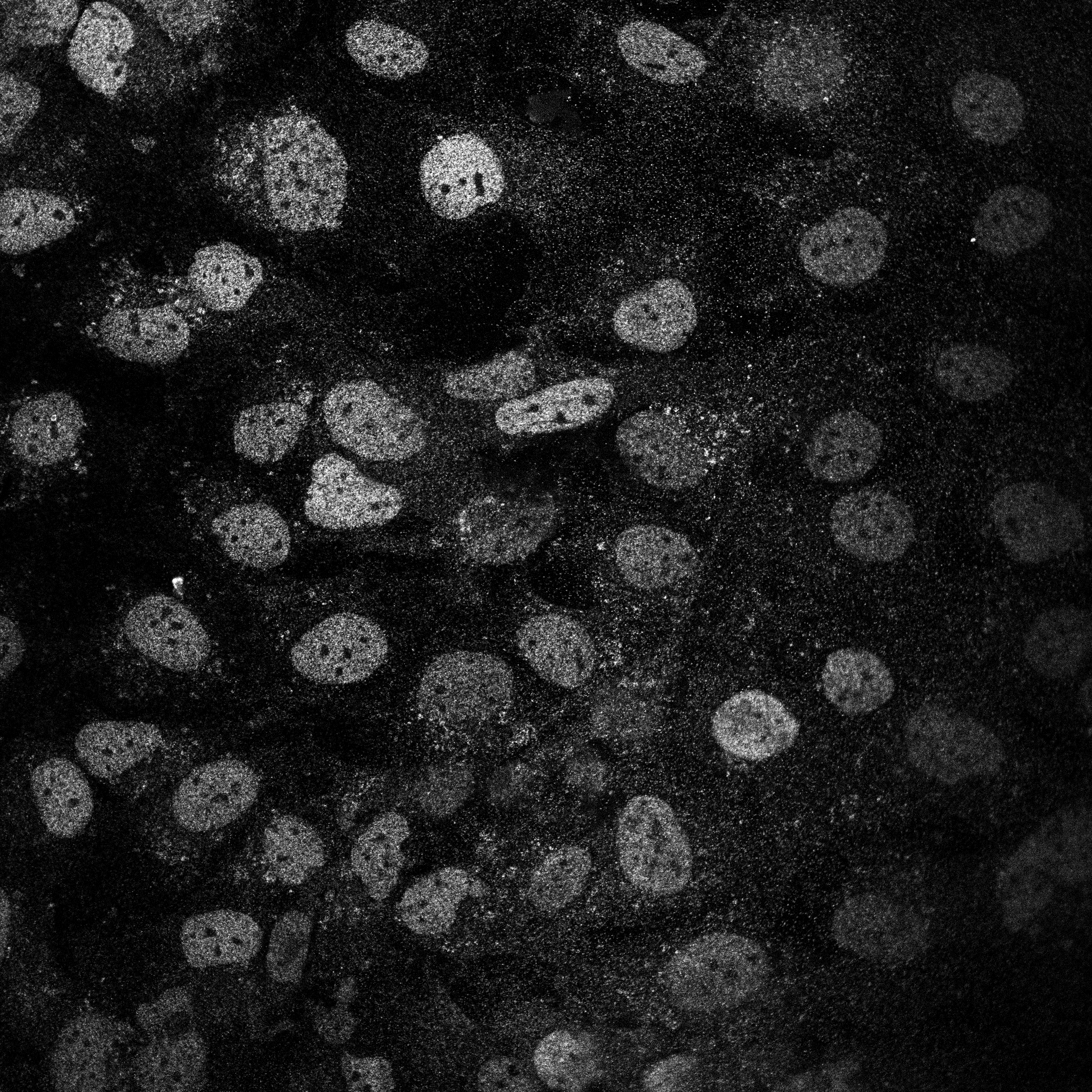

Supplement: Supplementary file 10 — Source data Fig. 5 [file 44319_2026_773_MOESM10_ESM.zip › Figure 5/Figure 5E/IF GRASP55KO Torin TFE3.tif]

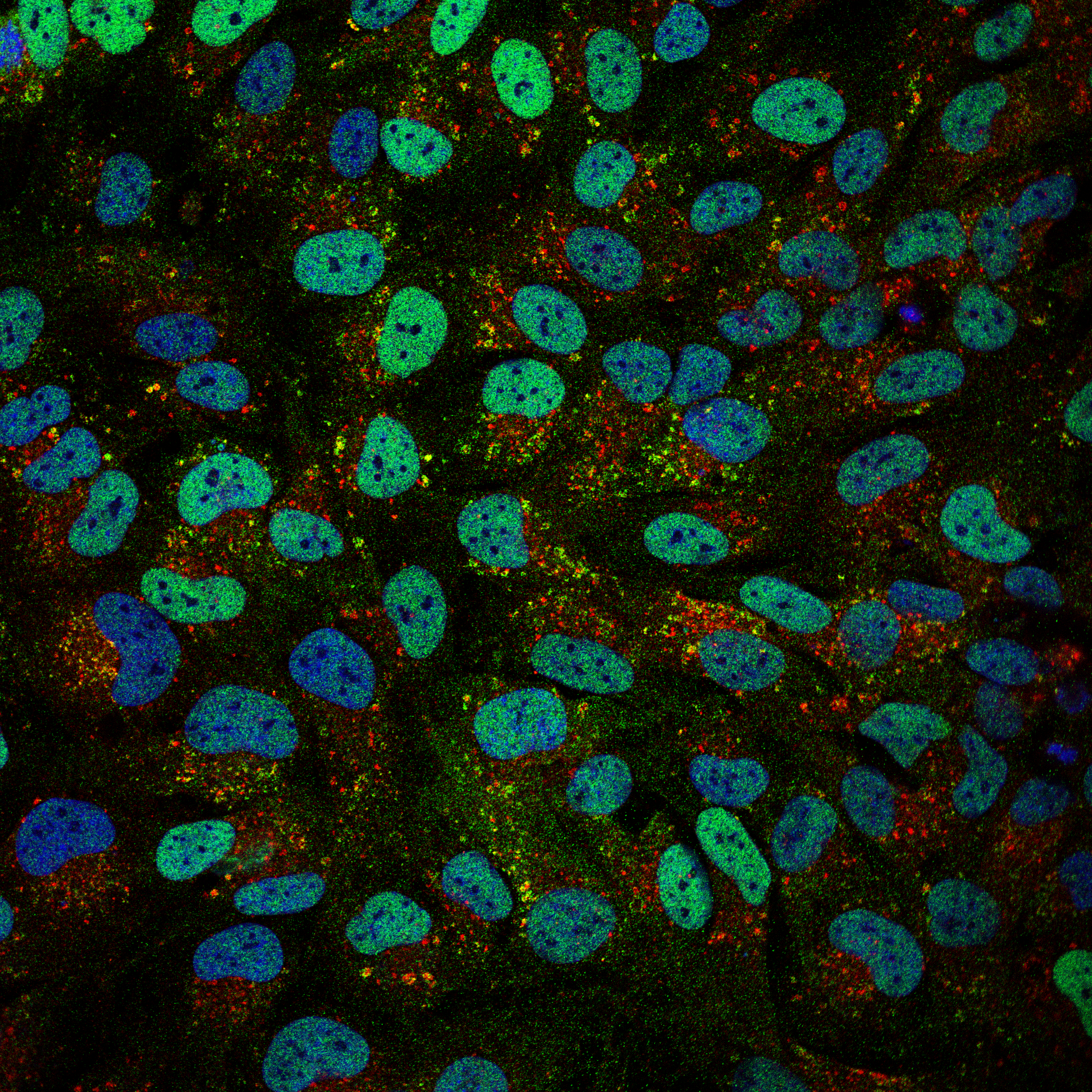

Supplement: Supplementary file 10 — Source data Fig. 5 [file 44319_2026_773_MOESM10_ESM.zip › Figure 5/Figure 5E/IF WT Torin TFE3_LAMP2 MERGE.tif]

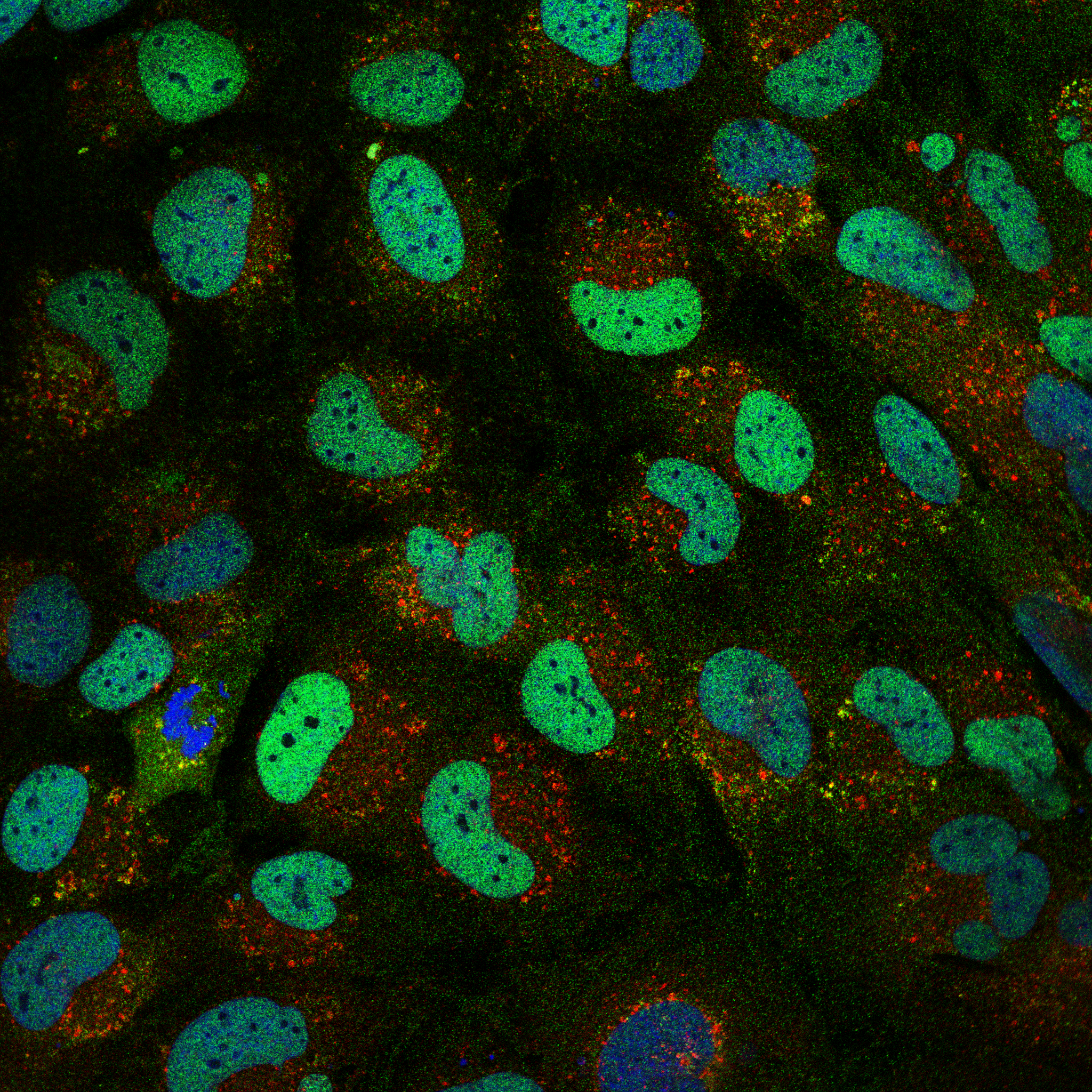

Supplement: Supplementary file 10 — Source data Fig. 5 [file 44319_2026_773_MOESM10_ESM.zip › Figure 5/Figure 5E/IF GRASP65KO Torin TFE3_LAMP2 MERGE.tif]

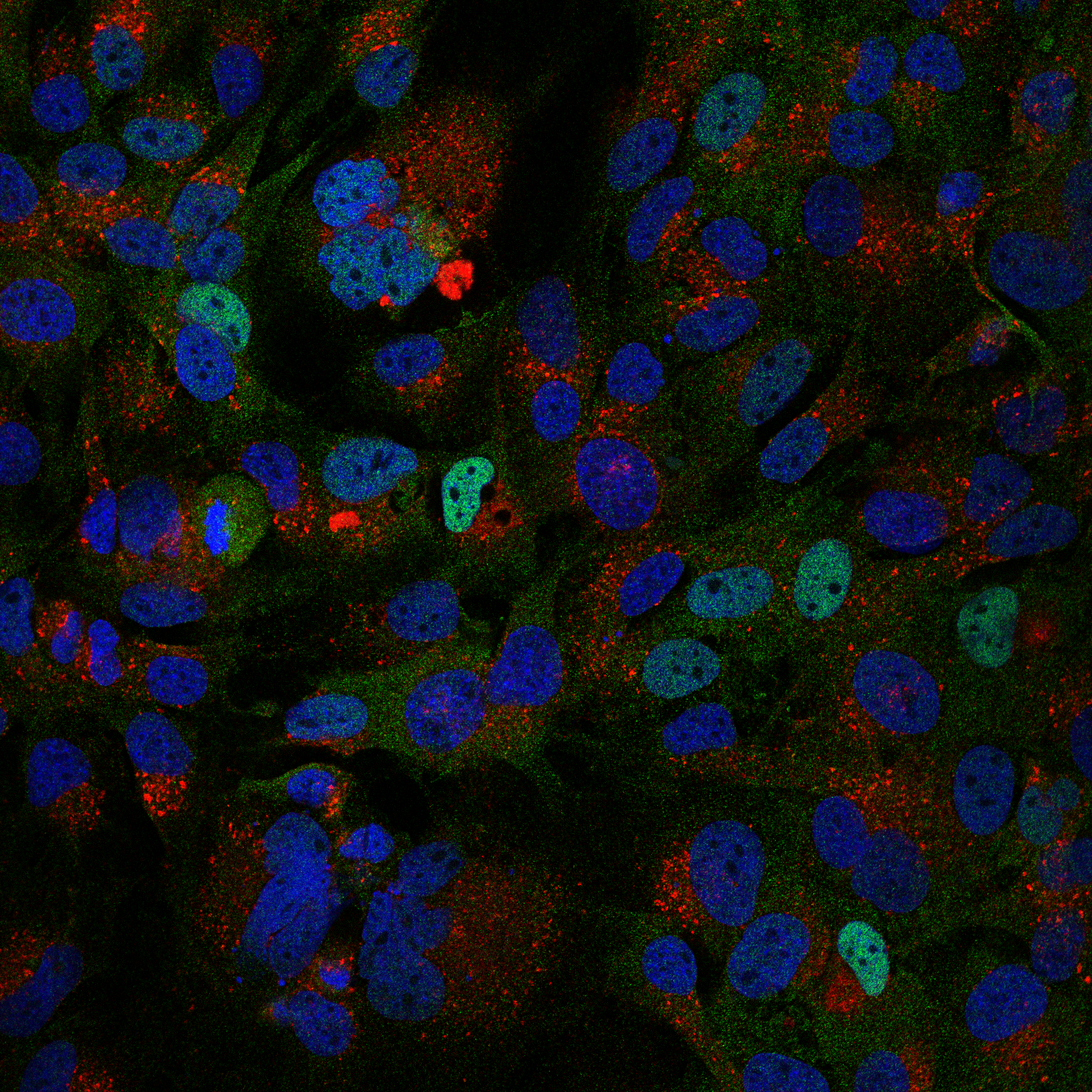

Supplement: Supplementary file 10 — Source data Fig. 5 [file 44319_2026_773_MOESM10_ESM.zip › Figure 5/Figure 5E/IF GRASP55KO DMSO TFE3_LAMP2 MERGE.tif]

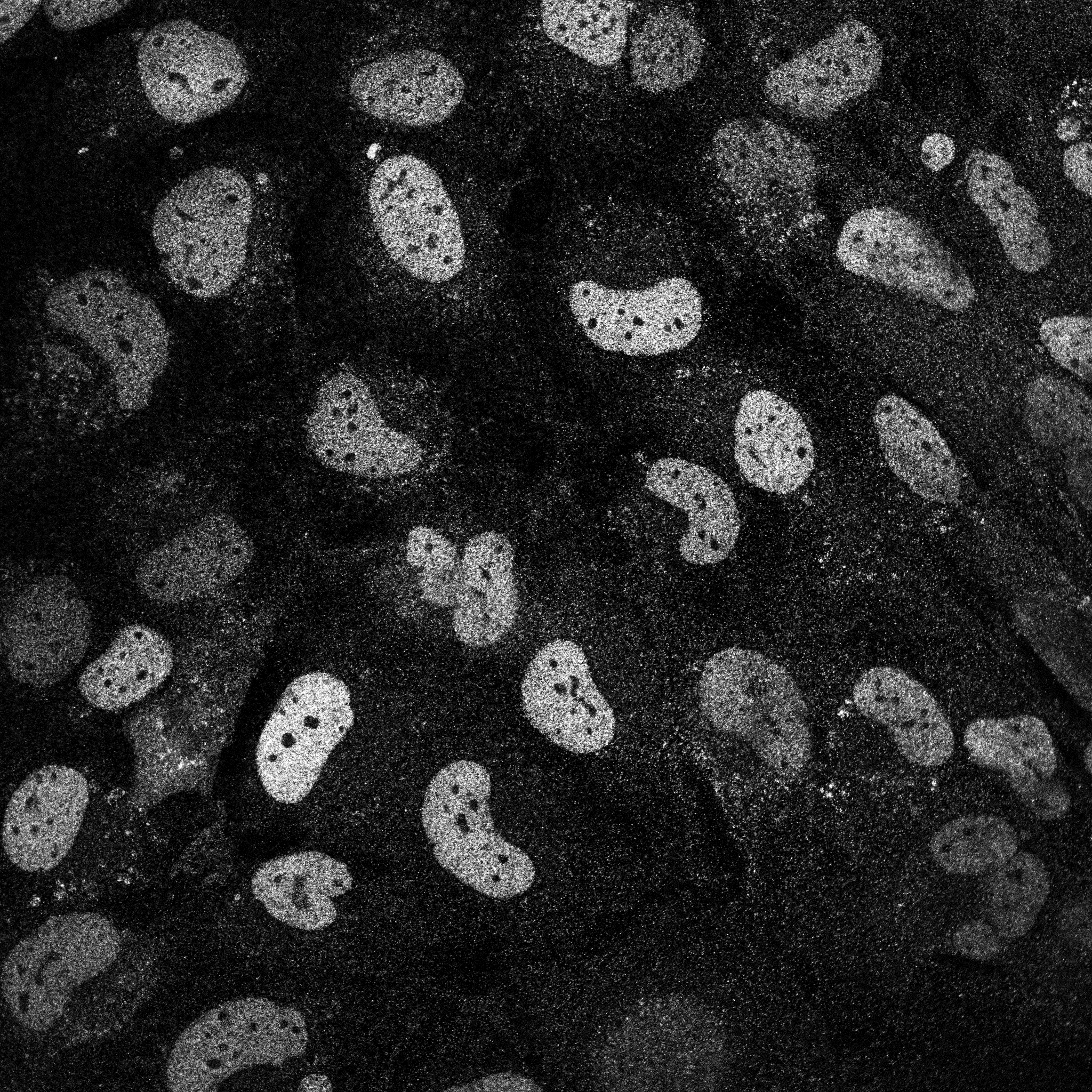

Supplement: Supplementary file 10 — Source data Fig. 5 [file 44319_2026_773_MOESM10_ESM.zip › Figure 5/Figure 5E/IF GRASP65KO Torin TFE3.tif]

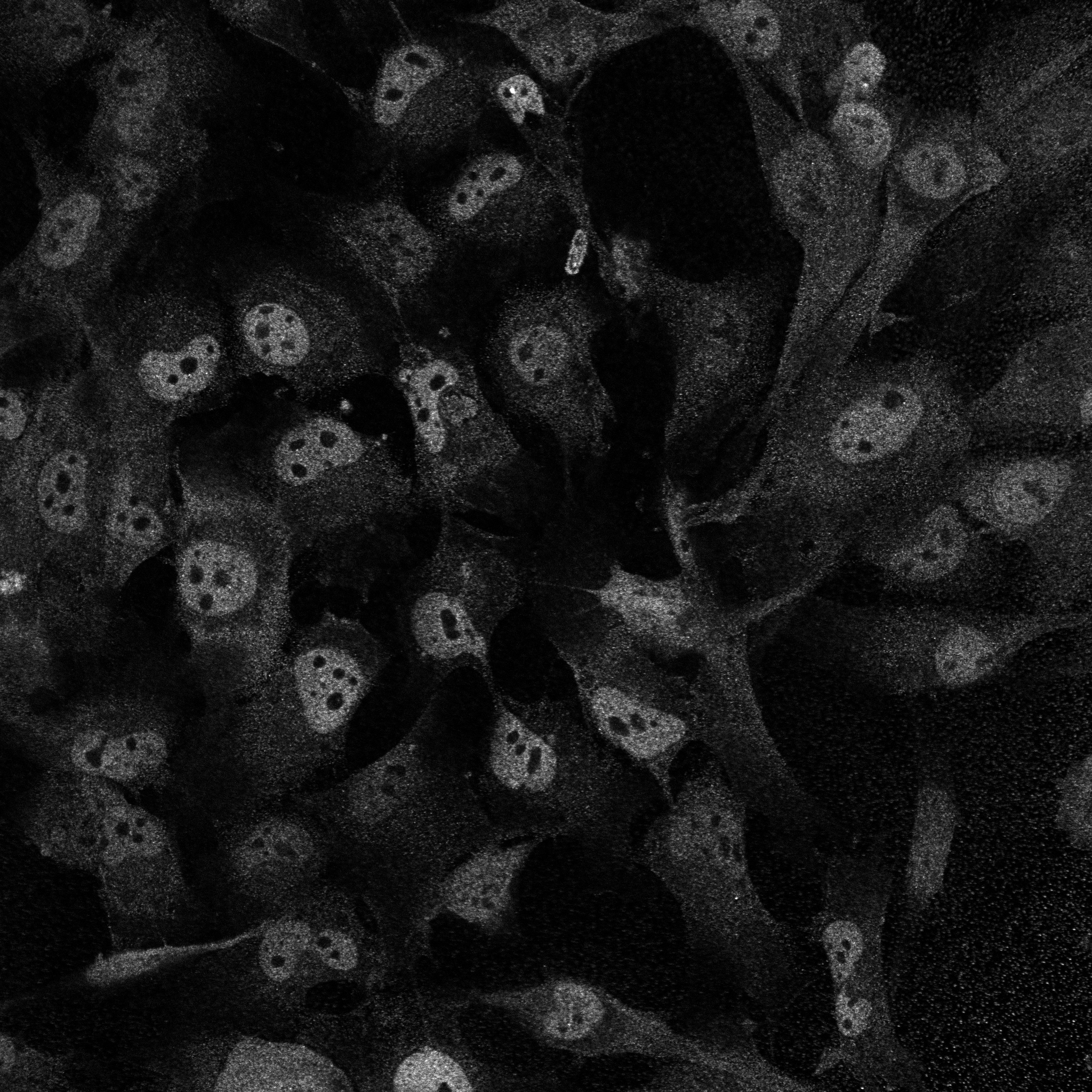

Supplement: Supplementary file 10 — Source data Fig. 5 [file 44319_2026_773_MOESM10_ESM.zip › Figure 5/Figure 5E/IF GNPTABKO DMSO TFE3.tif]

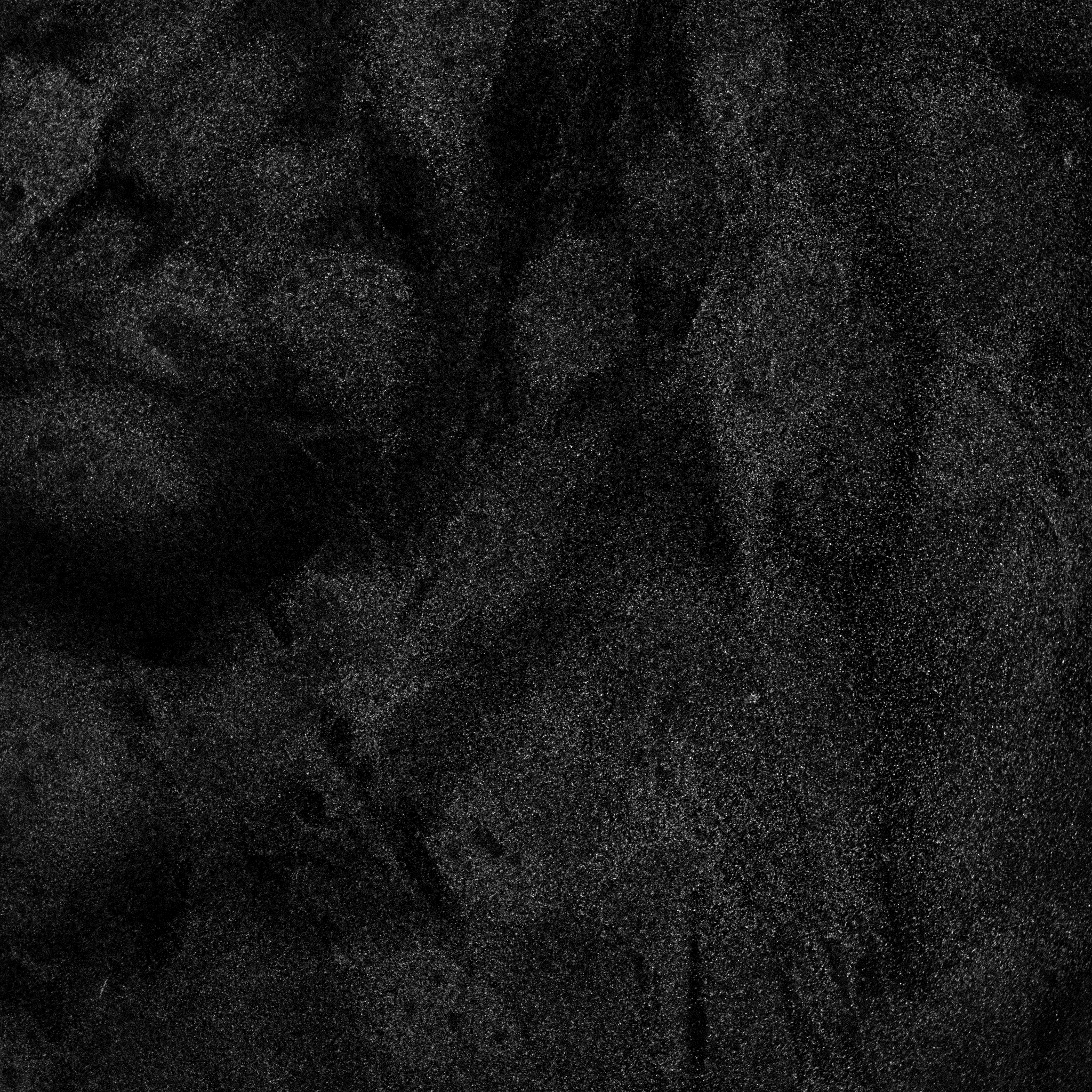

Supplement: Supplementary file 10 — Source data Fig. 5 [file 44319_2026_773_MOESM10_ESM.zip › Figure 5/Figure 5E/IF GRASP65KO DMSO TFE3.tif]

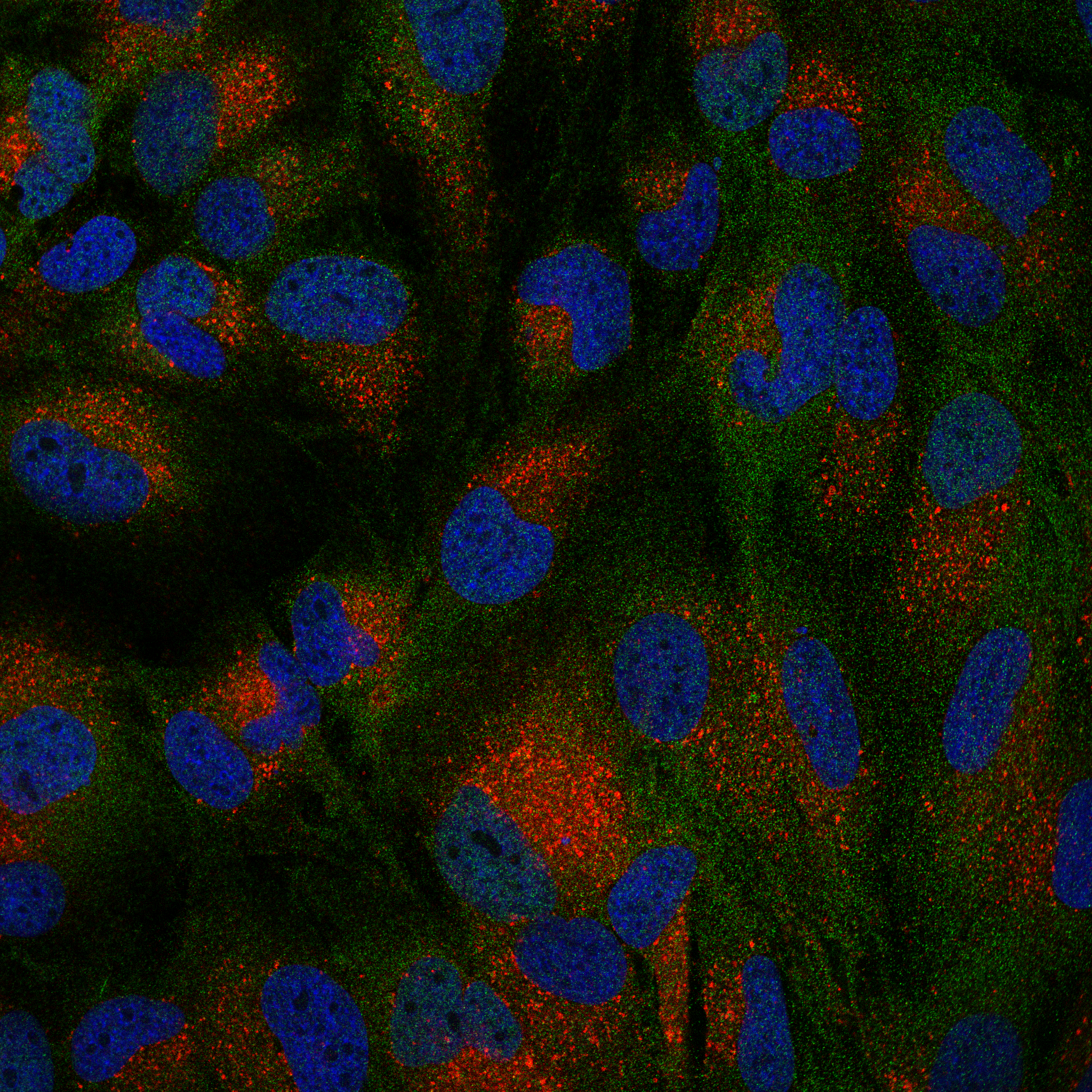

Supplement: Supplementary file 10 — Source data Fig. 5 [file 44319_2026_773_MOESM10_ESM.zip › Figure 5/Figure 5E/IF GRASP65KO DMSO TFE3_LAMP2 MERGE.tif]

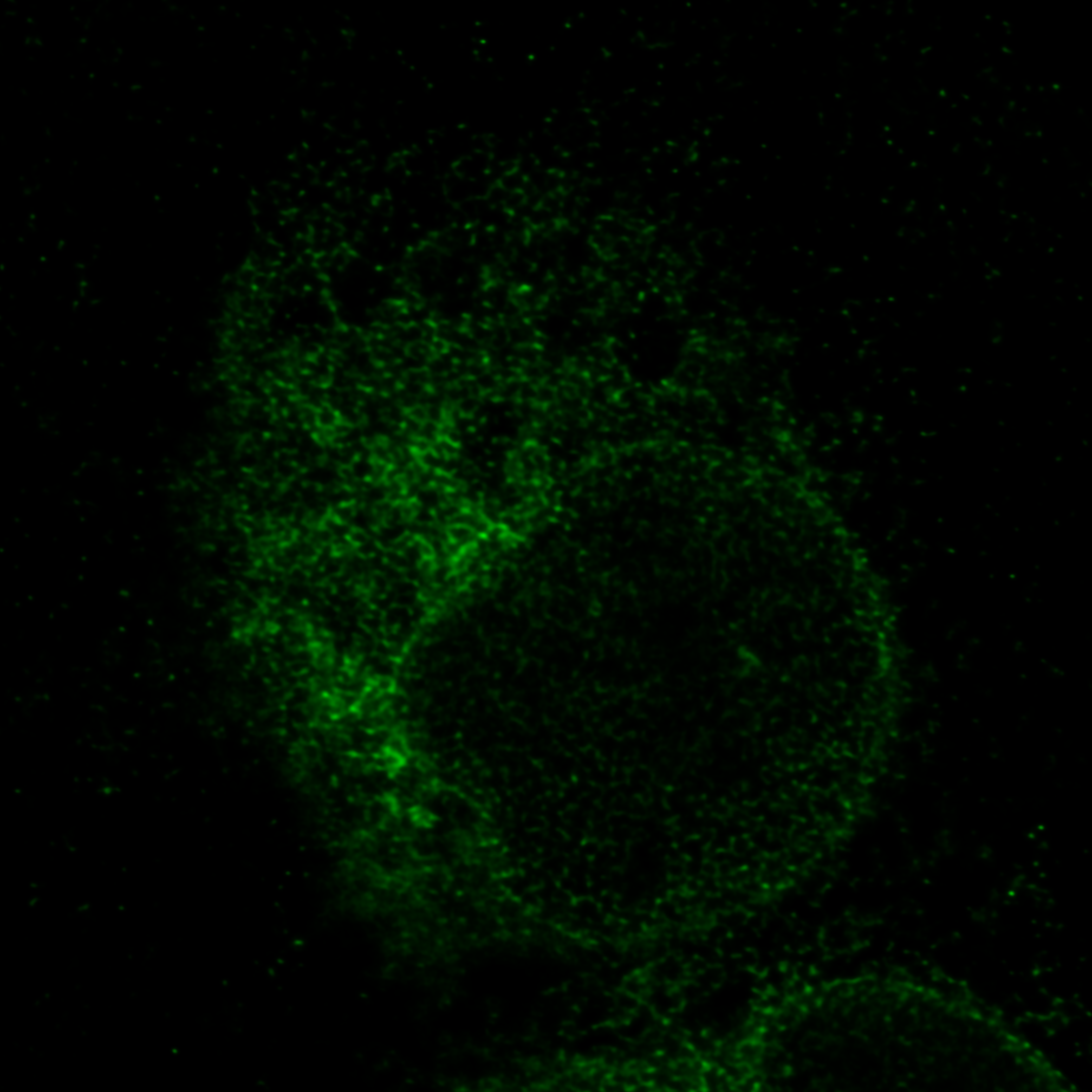

Supplement: Supplementary file 11 — Source data Fig. 6 [file 44319_2026_773_MOESM11_ESM.zip › Figure 6/Figure 6A/IF KO GNPTAB-MYC.tif]

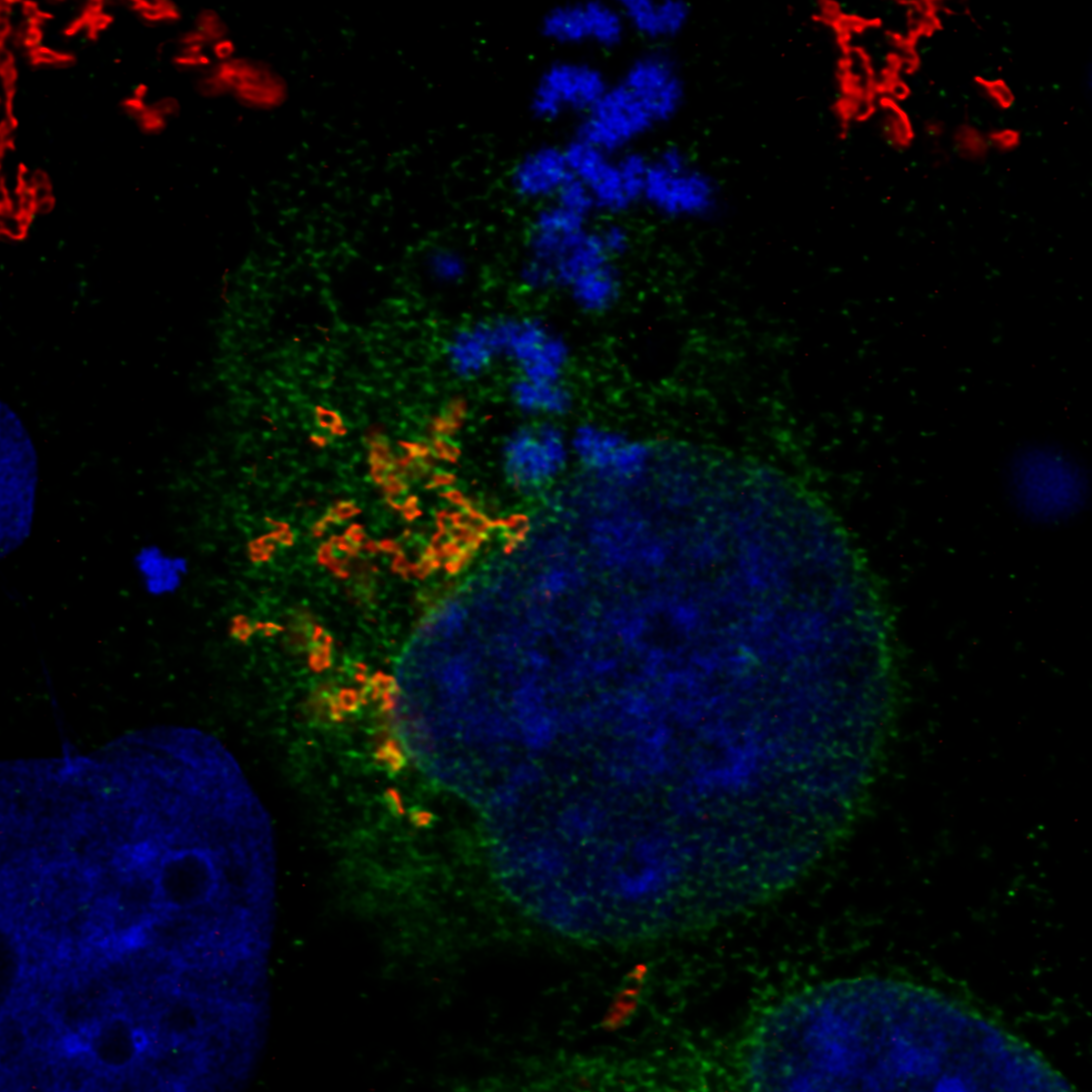

Supplement: Supplementary file 11 — Source data Fig. 6 [file 44319_2026_773_MOESM11_ESM.zip › Figure 6/Figure 6A/KO IF WT GNPTAB-MYC GM130.tif]

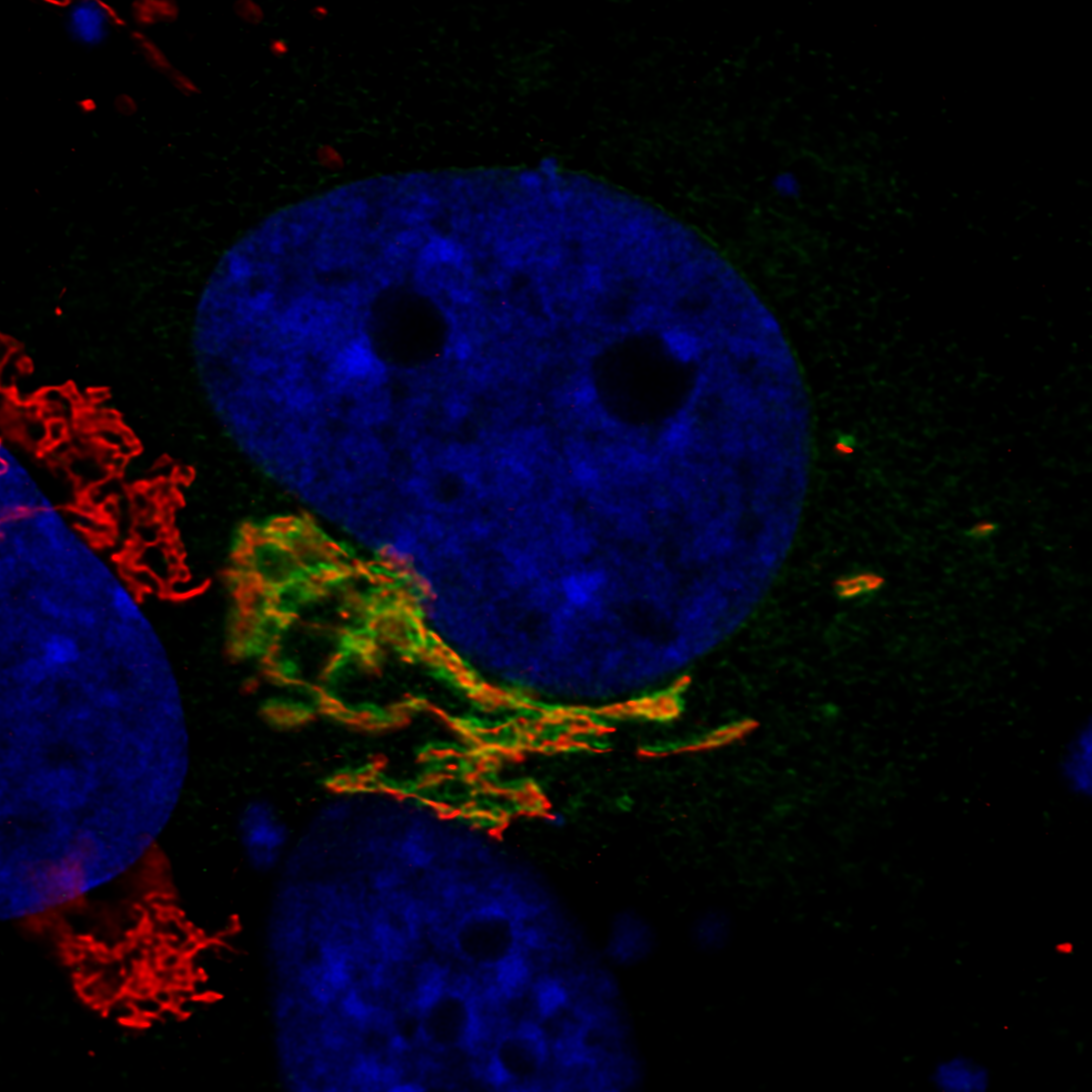

Supplement: Supplementary file 11 — Source data Fig. 6 [file 44319_2026_773_MOESM11_ESM.zip › Figure 6/Figure 6A/IF WT GNPTAB-MYC GM130 merge.tif]

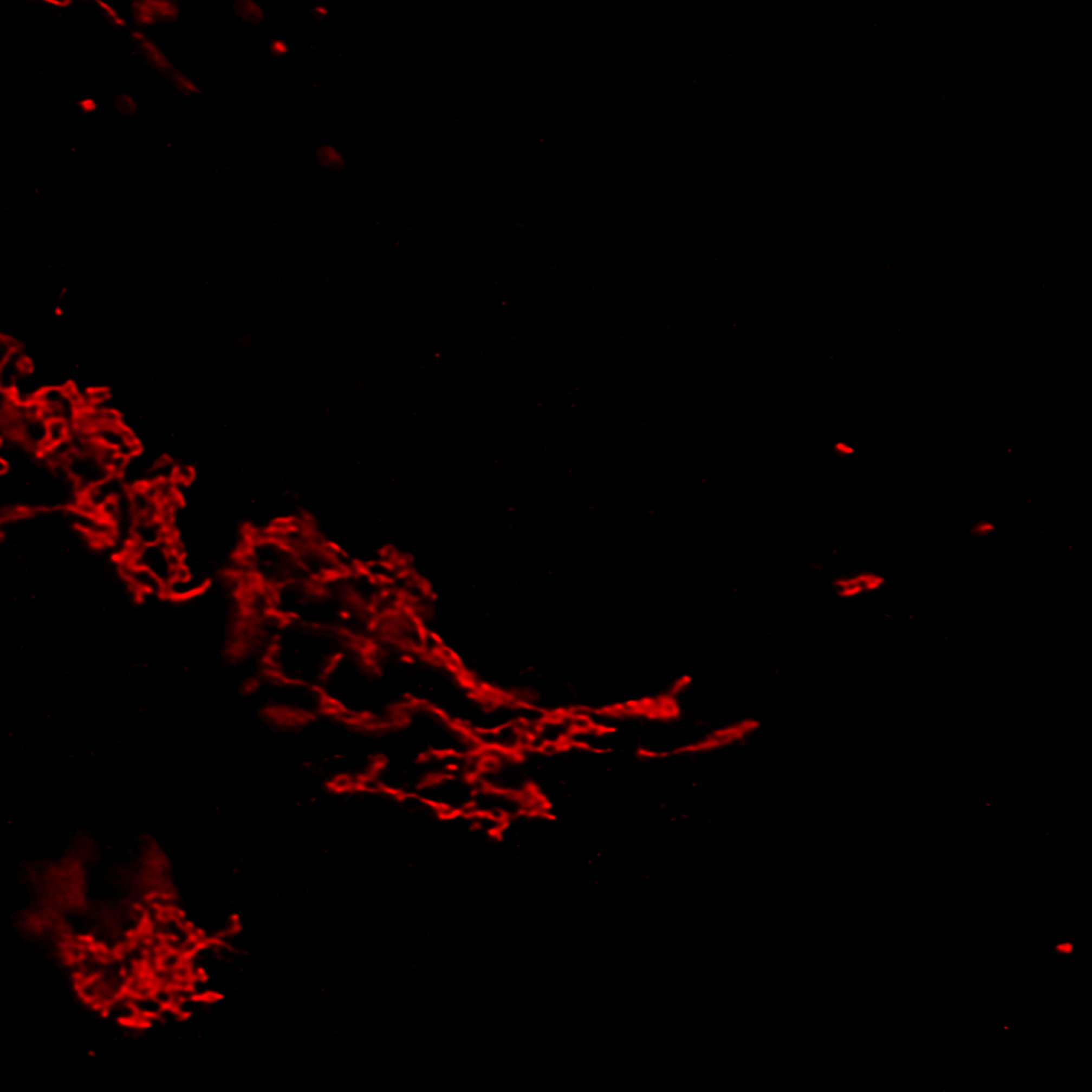

Supplement: Supplementary file 11 — Source data Fig. 6 [file 44319_2026_773_MOESM11_ESM.zip › Figure 6/Figure 6A/IF WT GM130.tif]

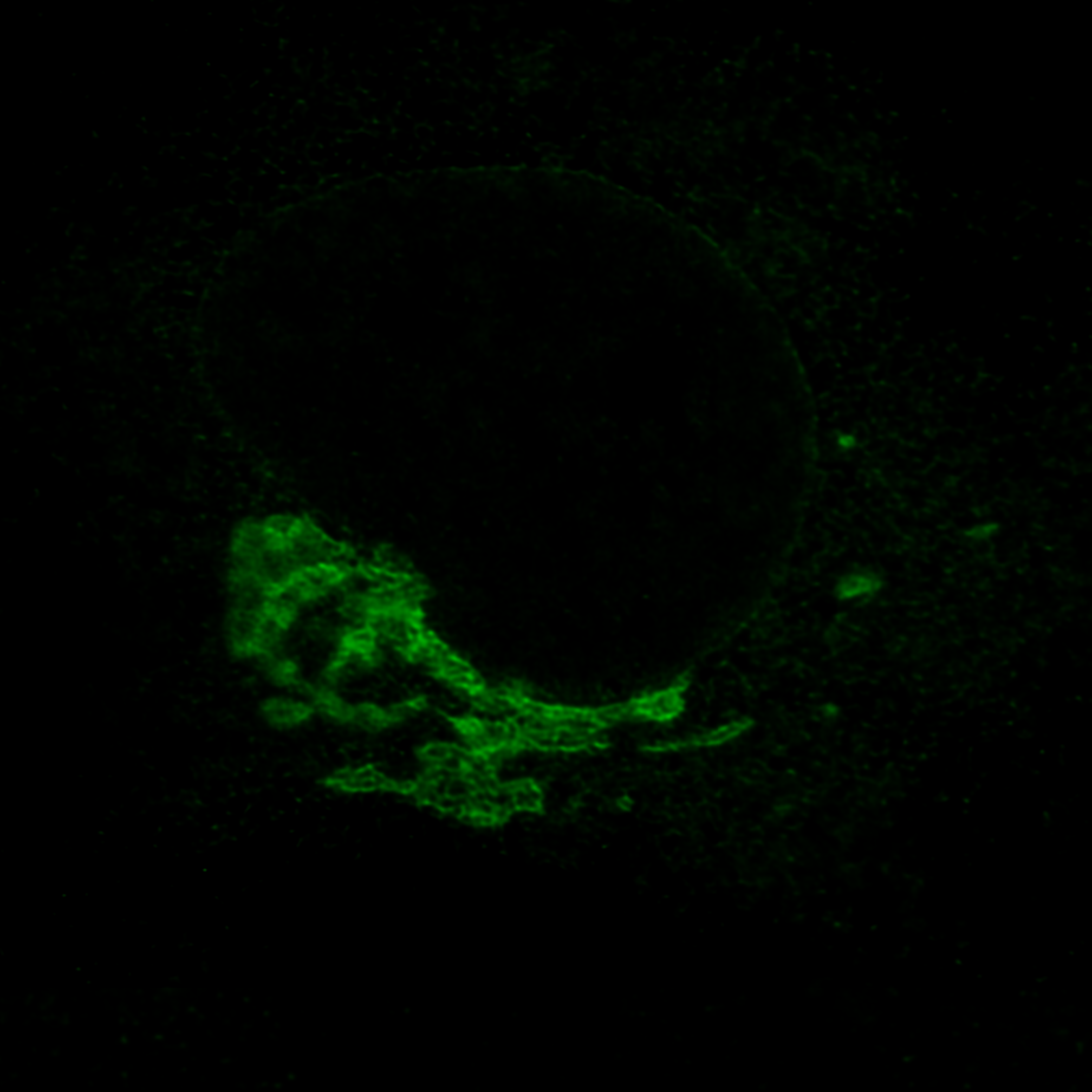

Supplement: Supplementary file 11 — Source data Fig. 6 [file 44319_2026_773_MOESM11_ESM.zip › Figure 6/Figure 6A/IF WT GNPTAB-MYC.tif]

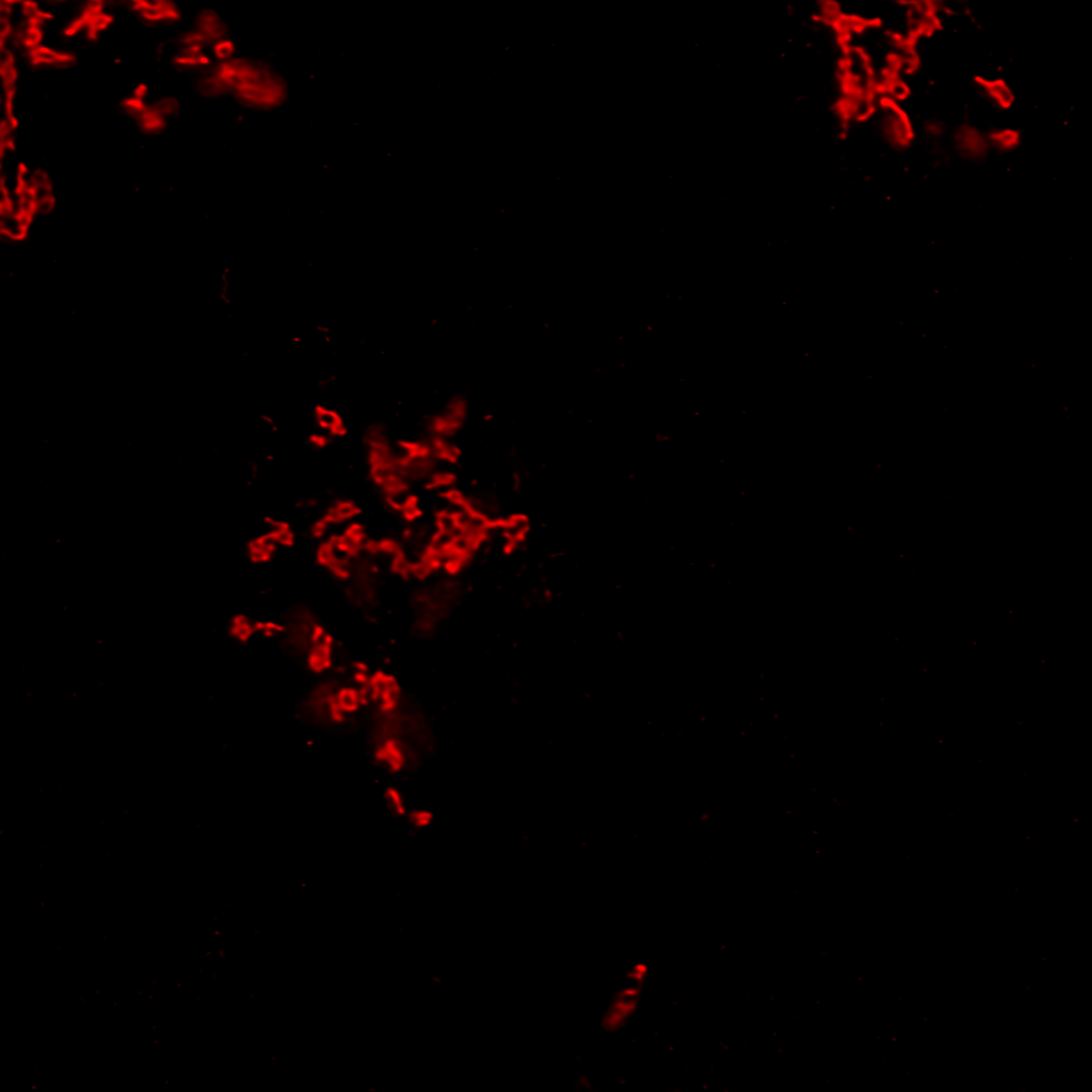

Supplement: Supplementary file 11 — Source data Fig. 6 [file 44319_2026_773_MOESM11_ESM.zip › Figure 6/Figure 6A/IF KO GM130.tif]

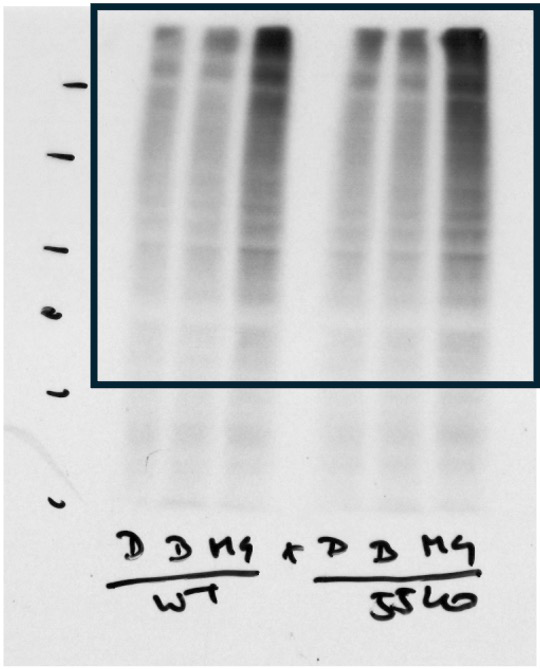

Supplement: Supplementary file 11 — Source data Fig. 6 [file 44319_2026_773_MOESM11_ESM.zip › Figure 6/Figure 6E/Western Ubiquitin.tif]

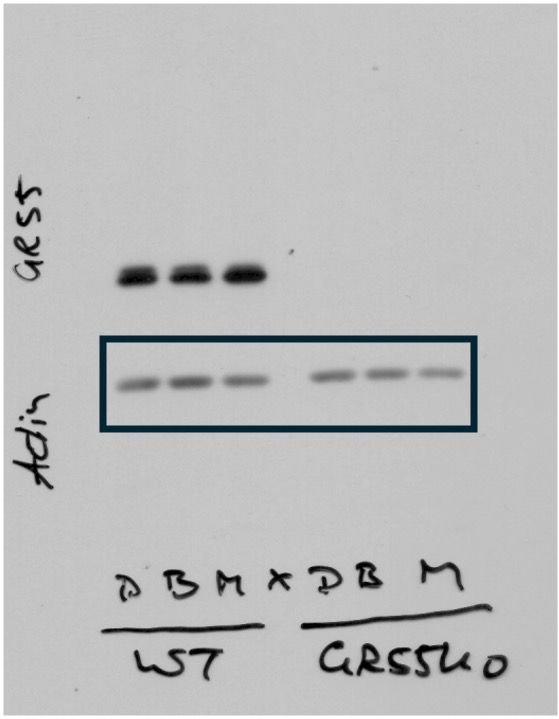

Supplement: Supplementary file 11 — Source data Fig. 6 [file 44319_2026_773_MOESM11_ESM.zip › Figure 6/Figure 6E/Western ACTIN.tif]

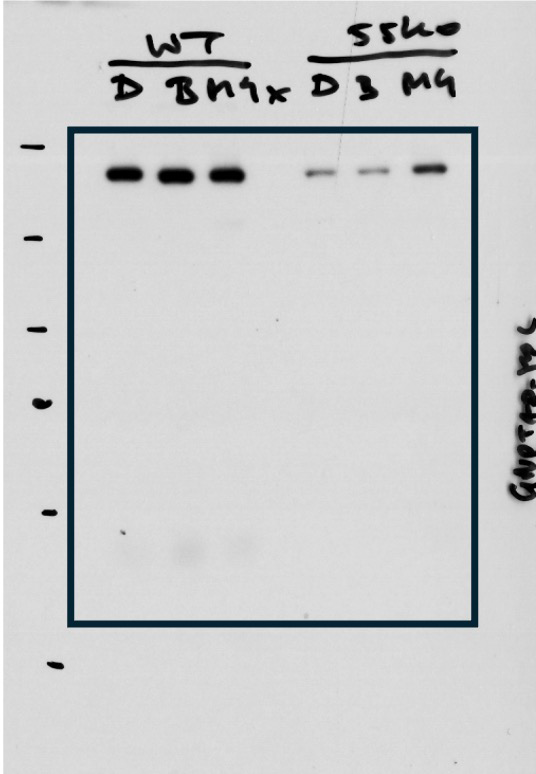

Supplement: Supplementary file 11 — Source data Fig. 6 [file 44319_2026_773_MOESM11_ESM.zip › Figure 6/Figure 6E/Western GNPTAB-Myc short.tif]

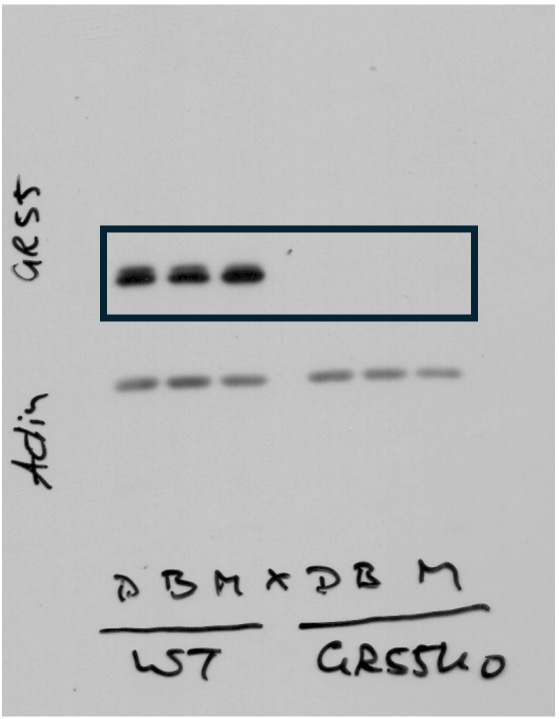

Supplement: Supplementary file 11 — Source data Fig. 6 [file 44319_2026_773_MOESM11_ESM.zip › Figure 6/Figure 6E/Western GRASP55.tif]

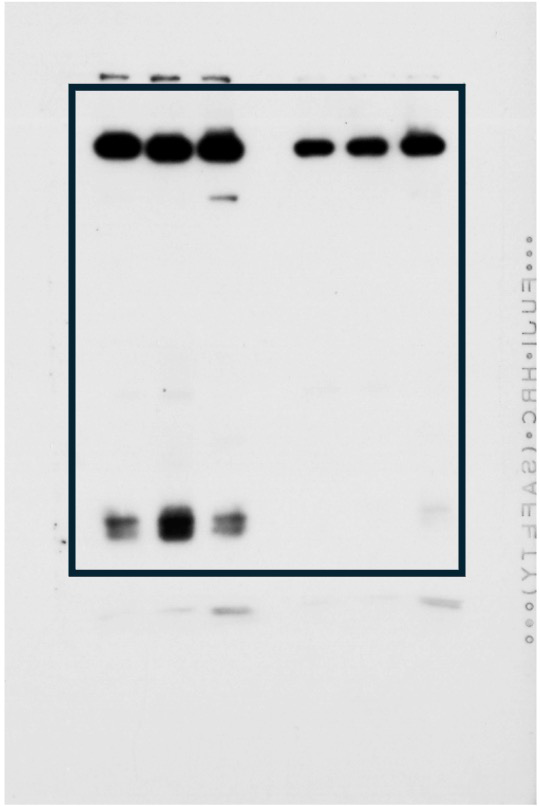

Supplement: Supplementary file 11 — Source data Fig. 6 [file 44319_2026_773_MOESM11_ESM.zip › Figure 6/Figure 6E/Western GNPTAB-Myc long.tif]

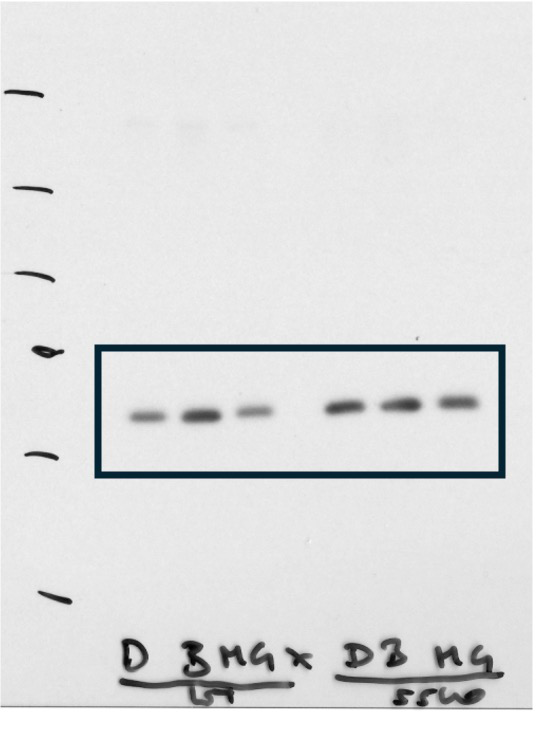

Supplement: Supplementary file 11 — Source data Fig. 6 [file 44319_2026_773_MOESM11_ESM.zip › Figure 6/Figure 6E/Western p62.tif]

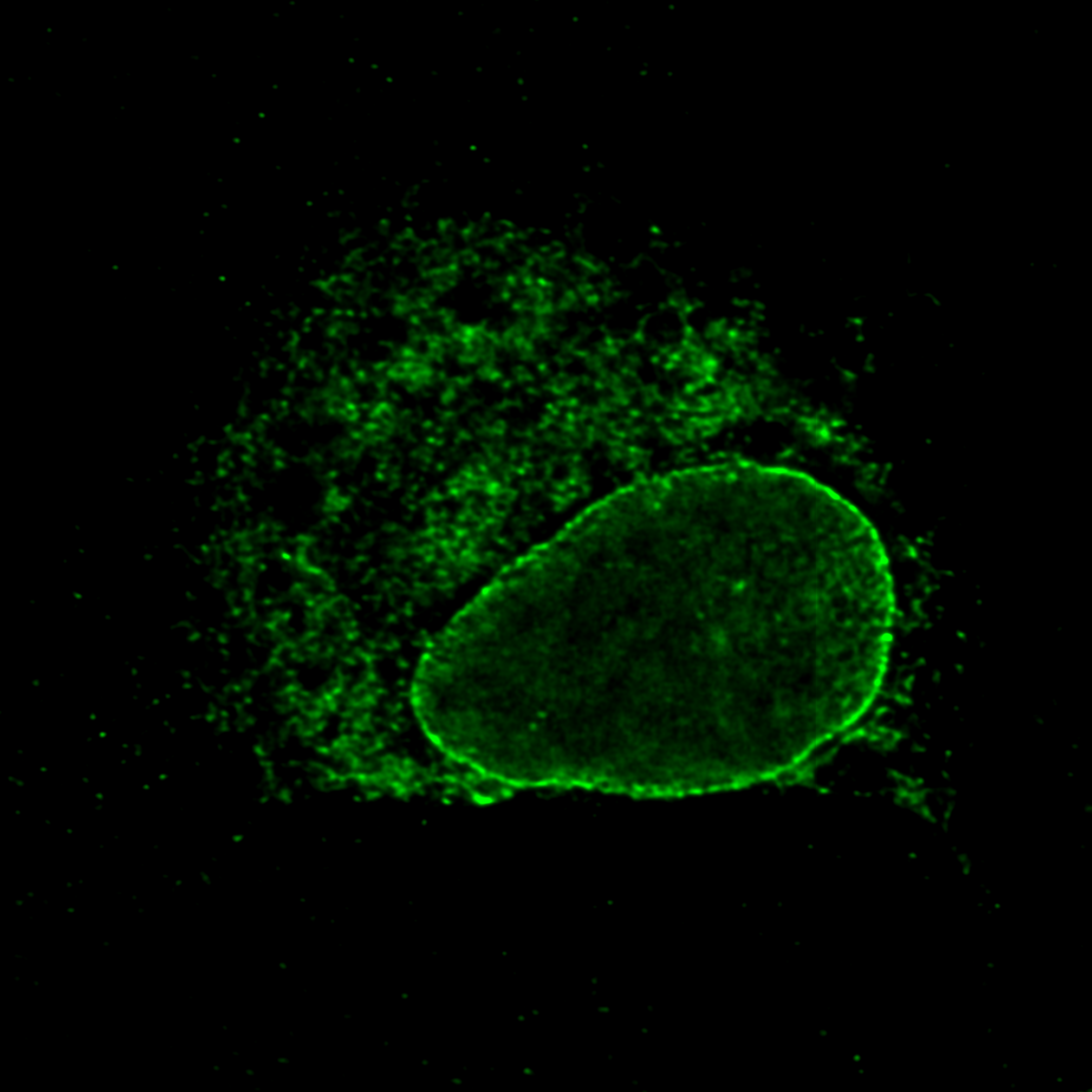

Supplement: Supplementary file 11 — Source data Fig. 6 [file 44319_2026_773_MOESM11_ESM.zip › Figure 6/Figure 6C/IF KO GNPTAB-Myc.tif]

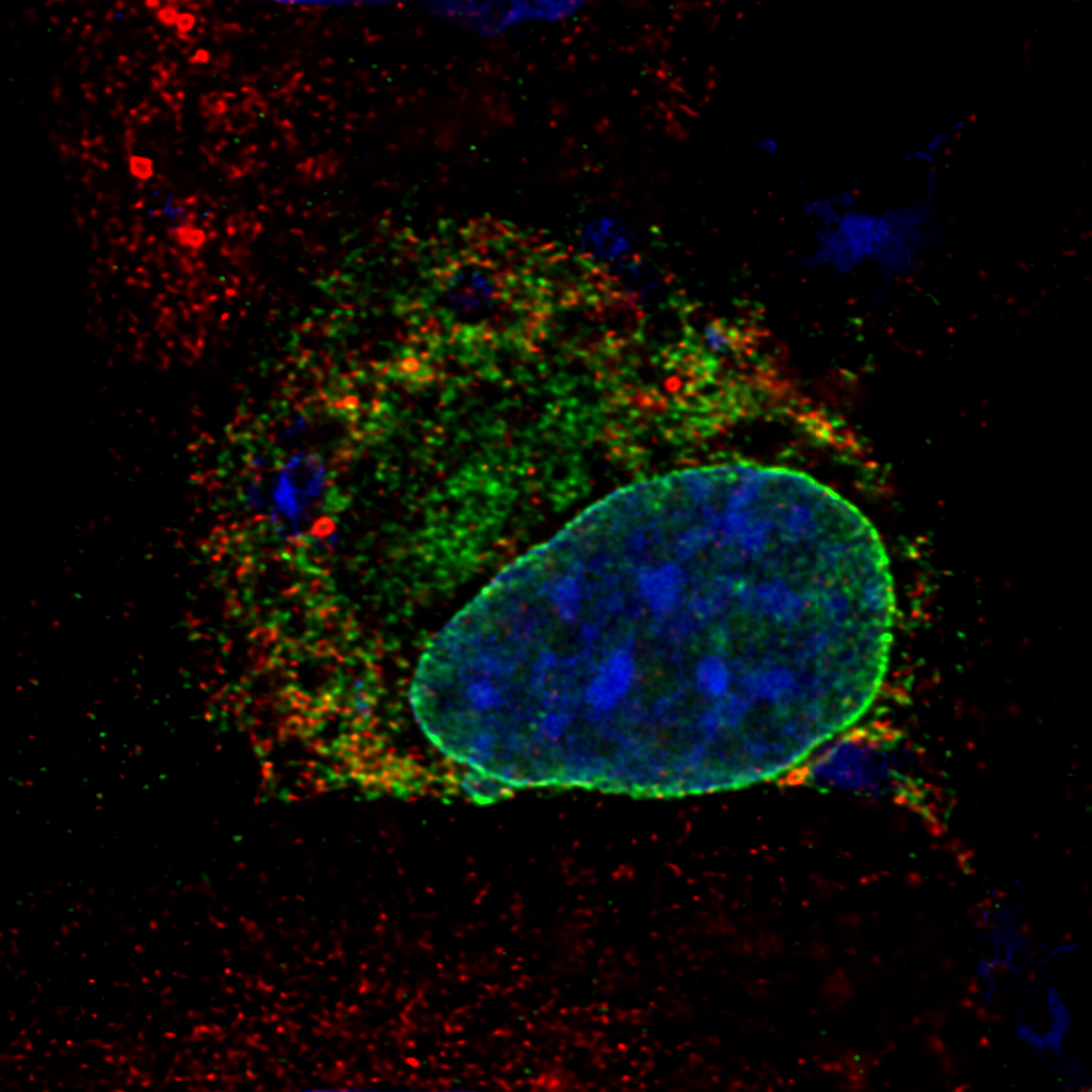

Supplement: Supplementary file 11 — Source data Fig. 6 [file 44319_2026_773_MOESM11_ESM.zip › Figure 6/Figure 6C/IF KO GNPTAB-Myc PDI MERGE.tif]

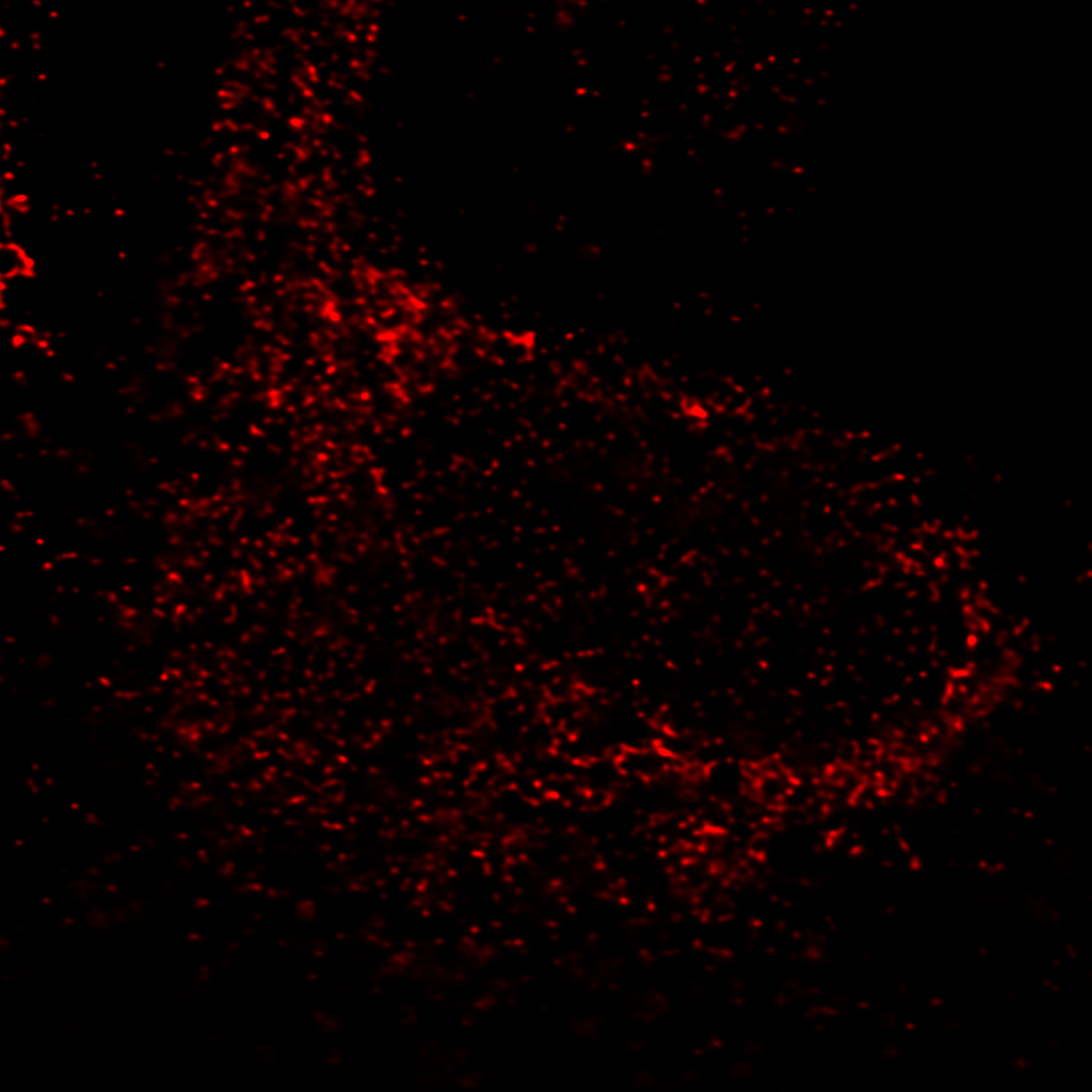

Supplement: Supplementary file 11 — Source data Fig. 6 [file 44319_2026_773_MOESM11_ESM.zip › Figure 6/Figure 6C/IF WT PDI .tif]

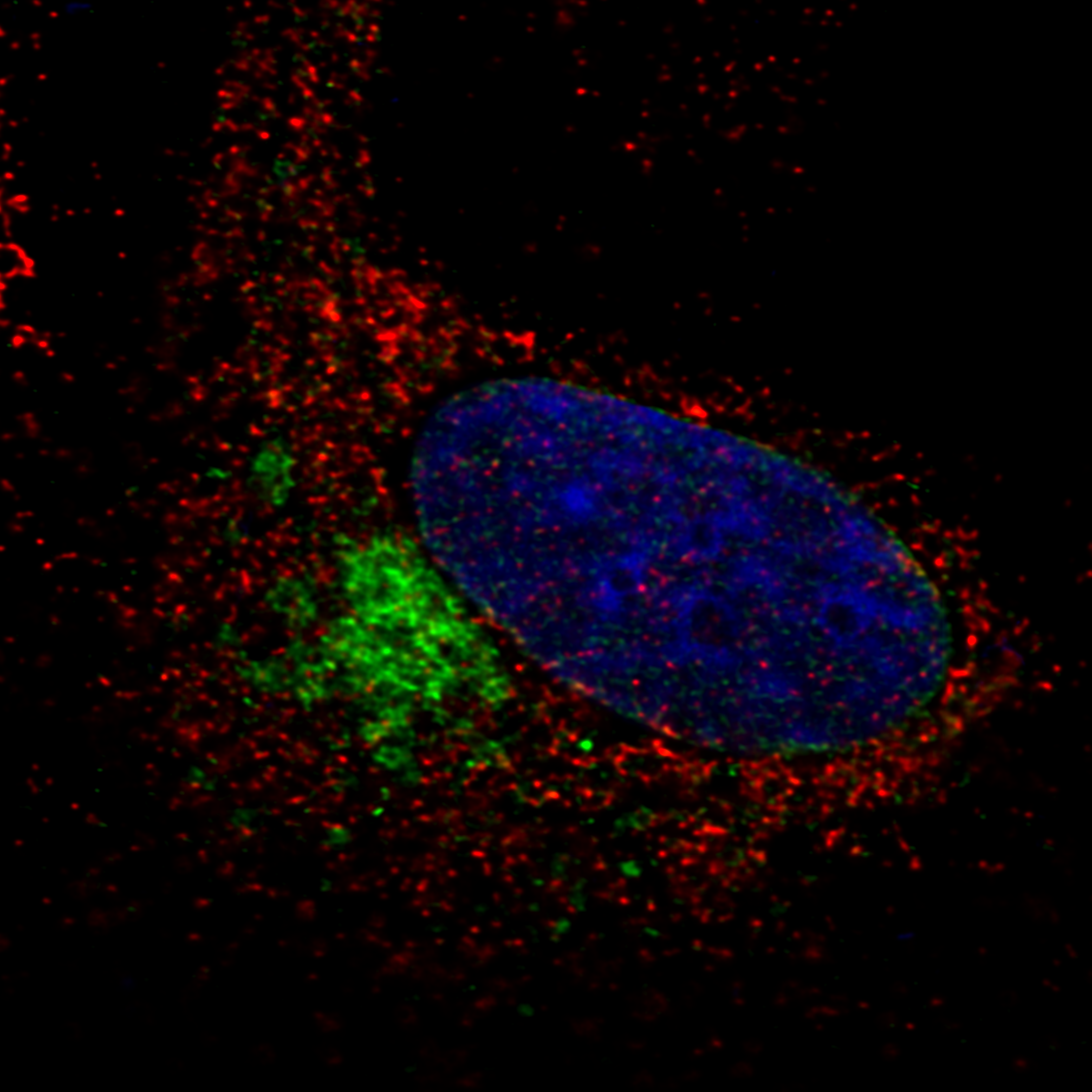

Supplement: Supplementary file 11 — Source data Fig. 6 [file 44319_2026_773_MOESM11_ESM.zip › Figure 6/Figure 6C/IF WT GNPTAB-Myc PDI MERGE.tif]

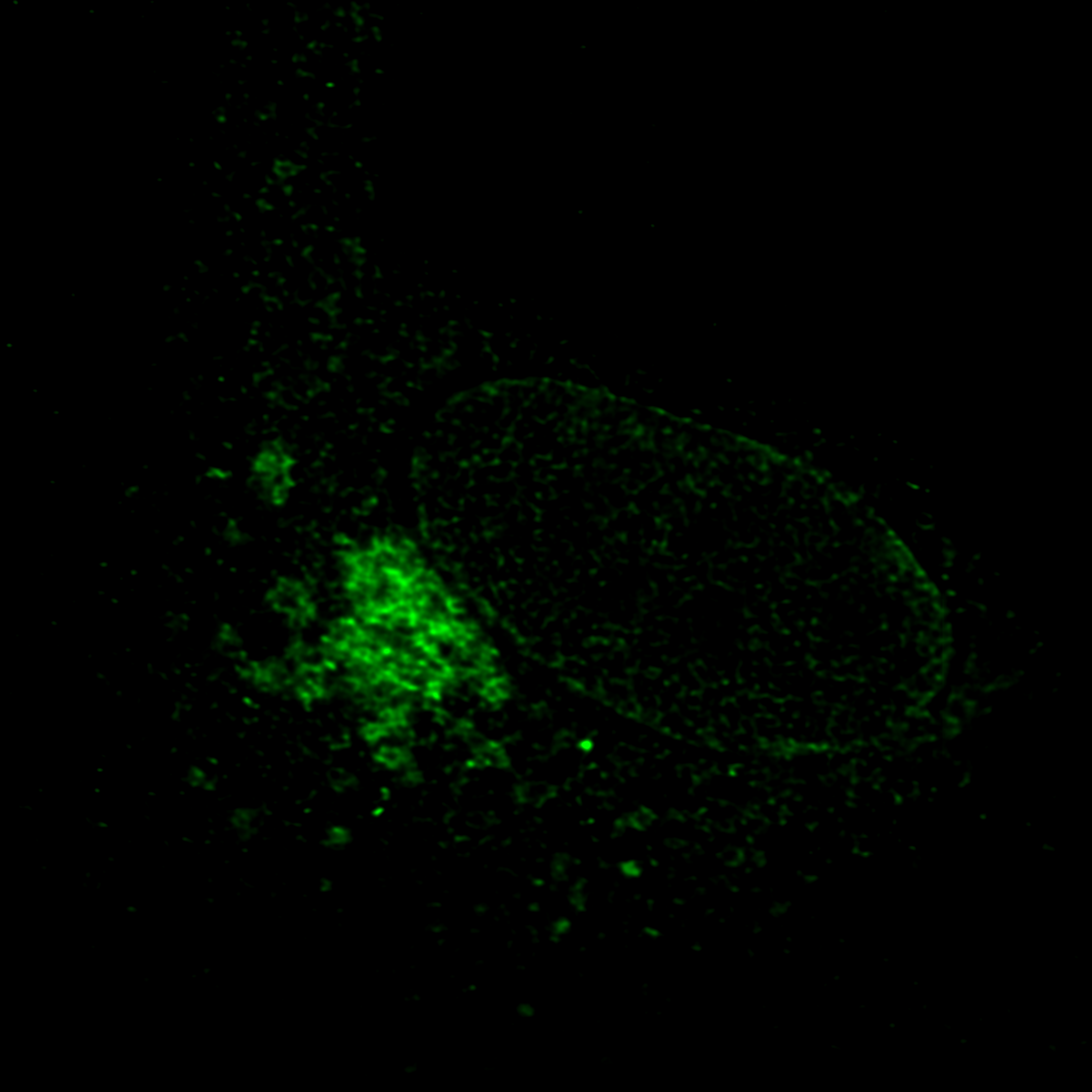

Supplement: Supplementary file 11 — Source data Fig. 6 [file 44319_2026_773_MOESM11_ESM.zip › Figure 6/Figure 6C/IF WT GNPTAB-Myc.tif]

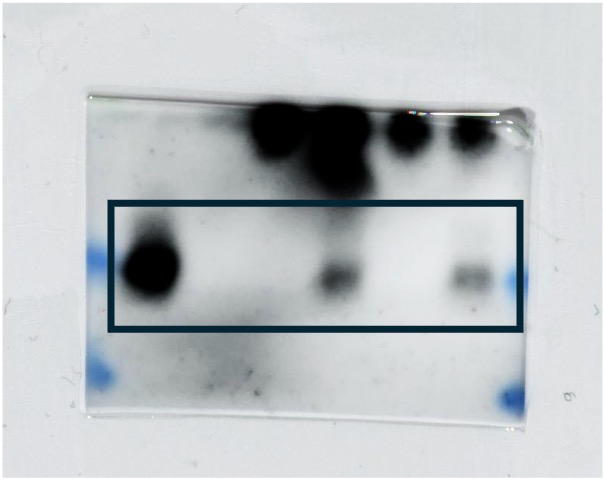

Supplement: Supplementary file 12 — Source data Fig. 7 [file 44319_2026_773_MOESM12_ESM.zip › Figure 7/Figure 7D/Western LYSET.tif]

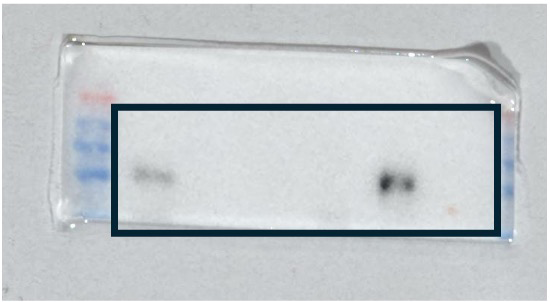

Supplement: Supplementary file 12 — Source data Fig. 7 [file 44319_2026_773_MOESM12_ESM.zip › Figure 7/Figure 7D/Western GM130.tif]

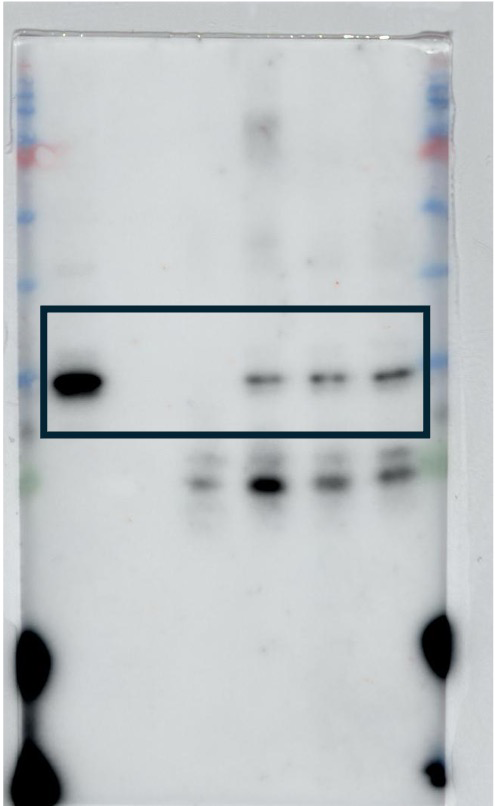

Supplement: Supplementary file 12 — Source data Fig. 7 [file 44319_2026_773_MOESM12_ESM.zip › Figure 7/Figure 7D/Western GOLPH3.tif]

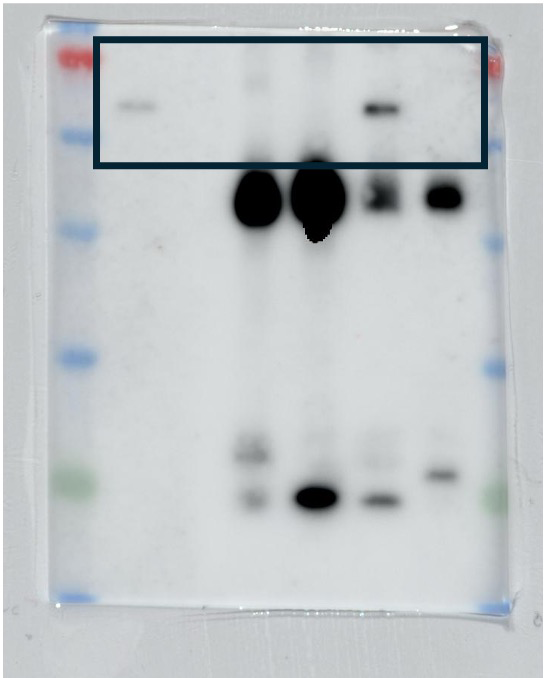

Supplement: Supplementary file 12 — Source data Fig. 7 [file 44319_2026_773_MOESM12_ESM.zip › Figure 7/Figure 7D/Western GRASP65.tif]

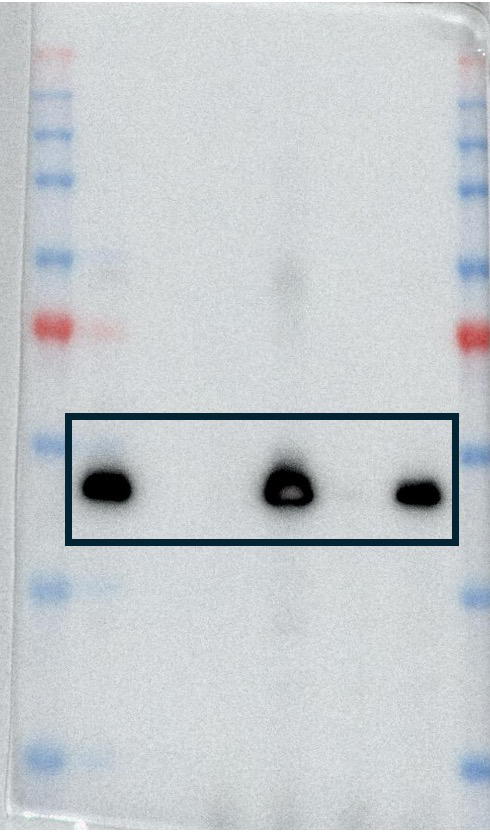

Supplement: Supplementary file 12 — Source data Fig. 7 [file 44319_2026_773_MOESM12_ESM.zip › Figure 7/Figure 7D/Western GRASP55.tif]

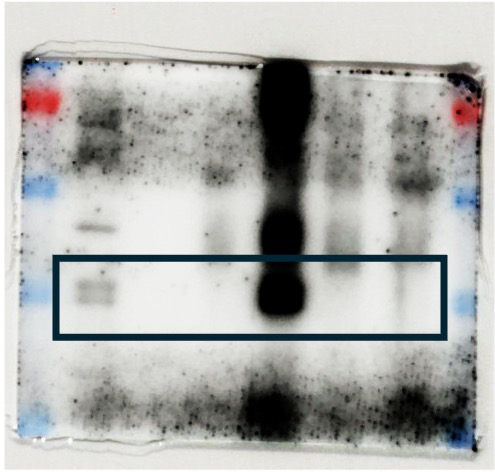

Supplement: Supplementary file 12 — Source data Fig. 7 [file 44319_2026_773_MOESM12_ESM.zip › Figure 7/Figure 7D/Western GOLGIN-45.tif]

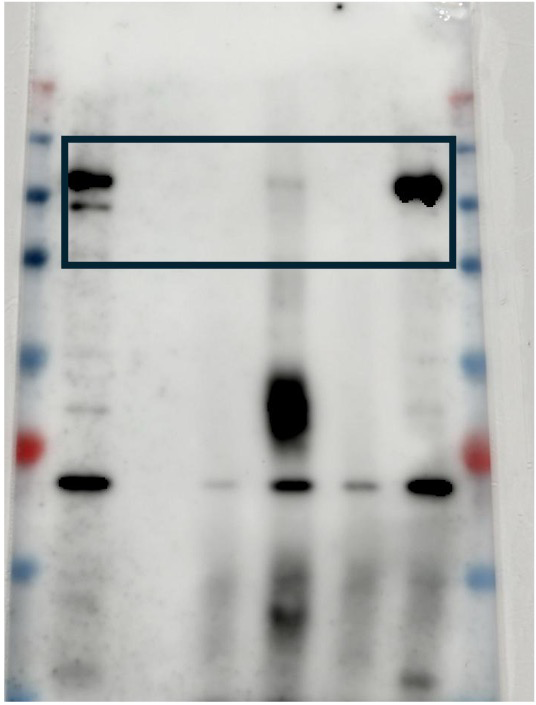

Supplement: Supplementary file 12 — Source data Fig. 7 [file 44319_2026_773_MOESM12_ESM.zip › Figure 7/Figure 7D/Western Myc.tif]

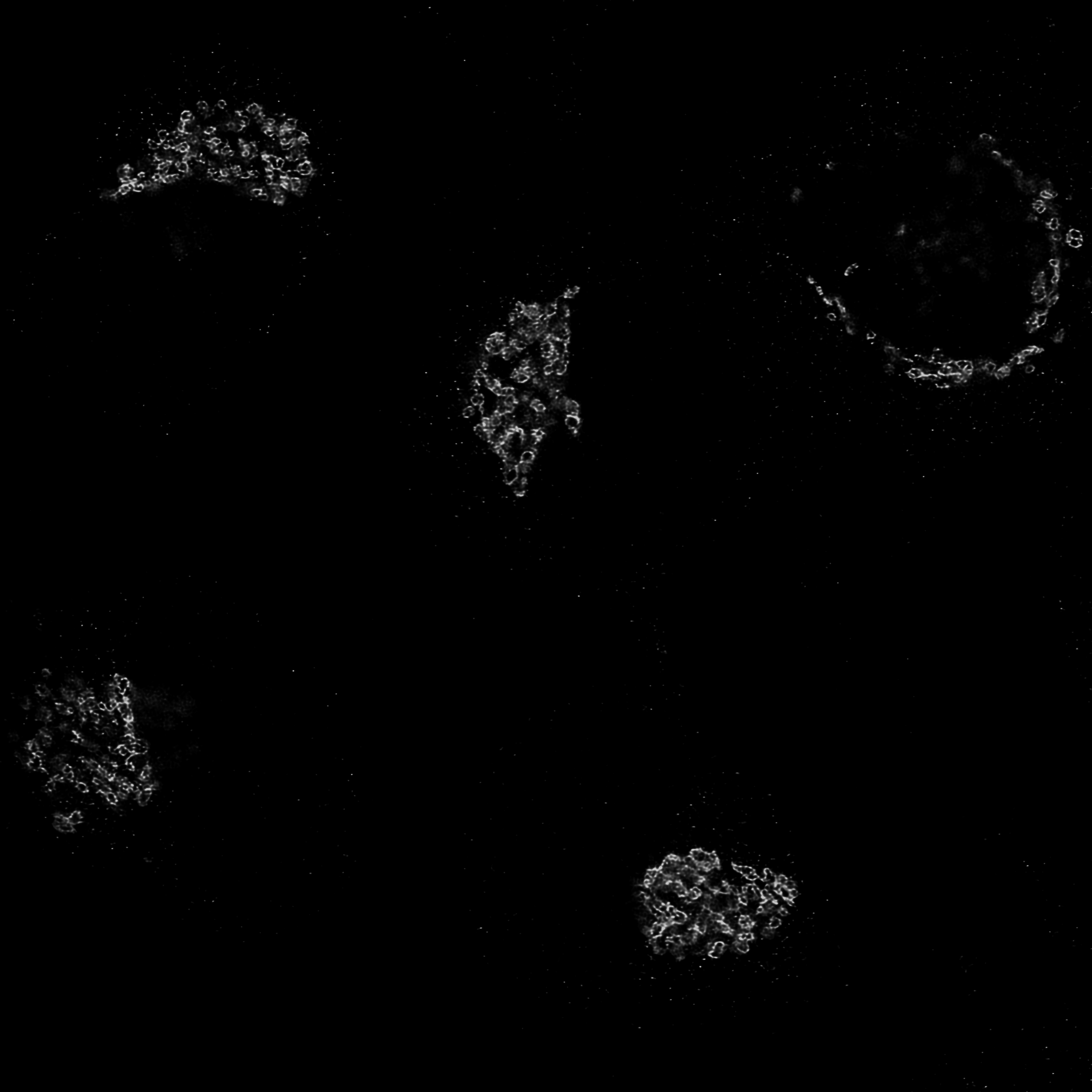

Supplement: Supplementary file 12 — Source data Fig. 7 [file 44319_2026_773_MOESM12_ESM.zip › Figure 7/Figure 7C/IF GRASP65KO GIANTIN.tif]

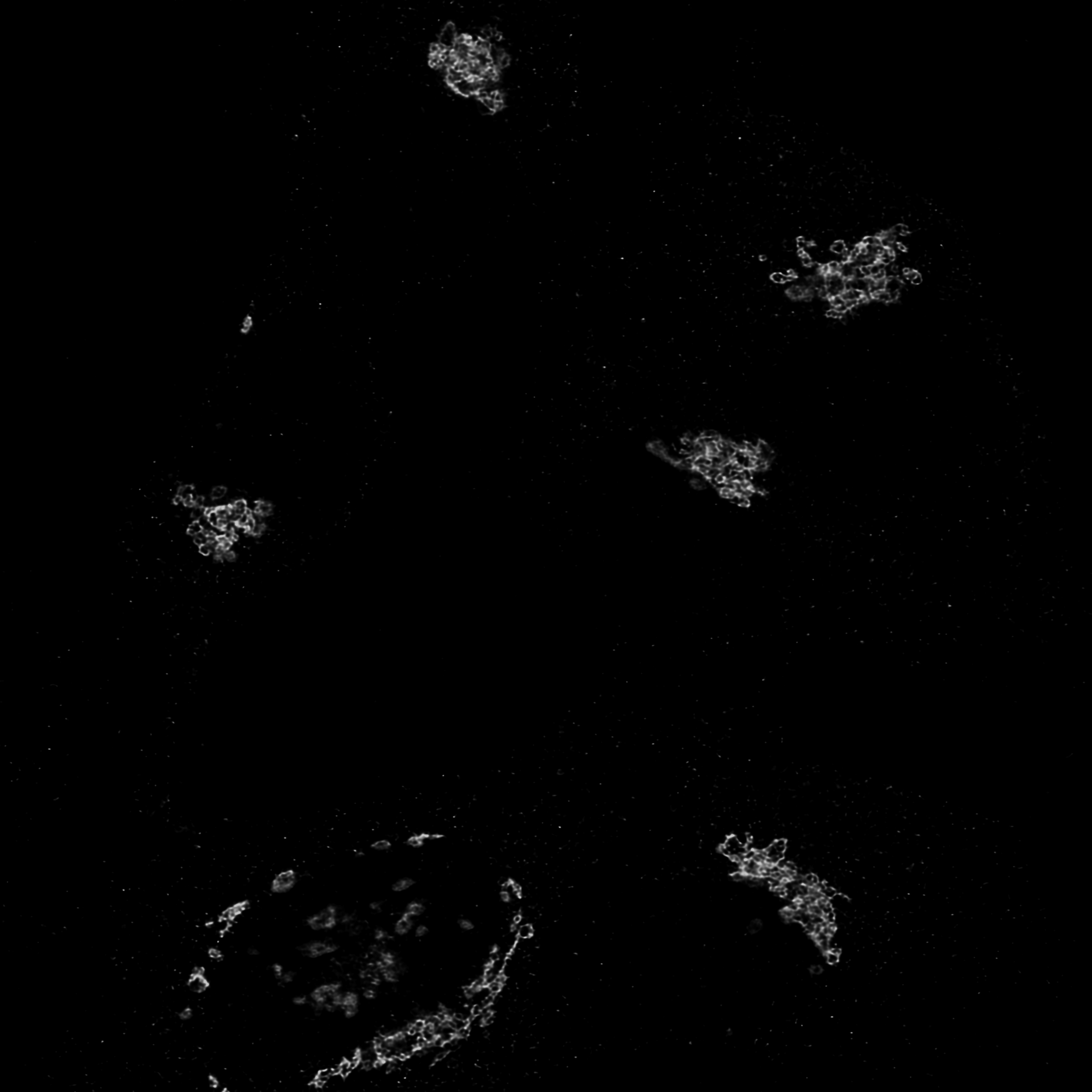

Supplement: Supplementary file 12 — Source data Fig. 7 [file 44319_2026_773_MOESM12_ESM.zip › Figure 7/Figure 7C/IF WT GIANTIN.tif]

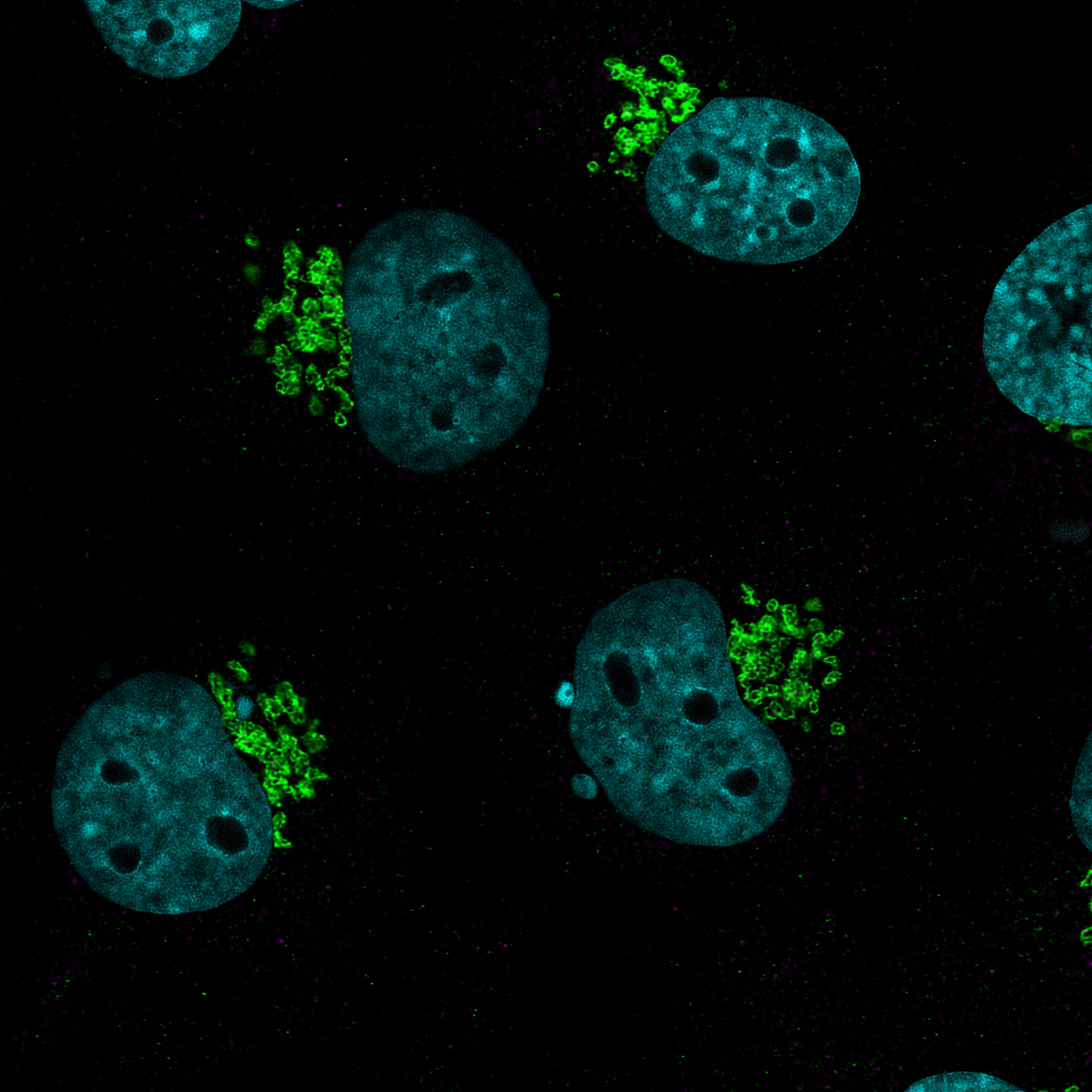

Supplement: Supplementary file 12 — Source data Fig. 7 [file 44319_2026_773_MOESM12_ESM.zip › Figure 7/Figure 7C/IF GRASP55KO LYSET_GIANTIN MERGE.tif]

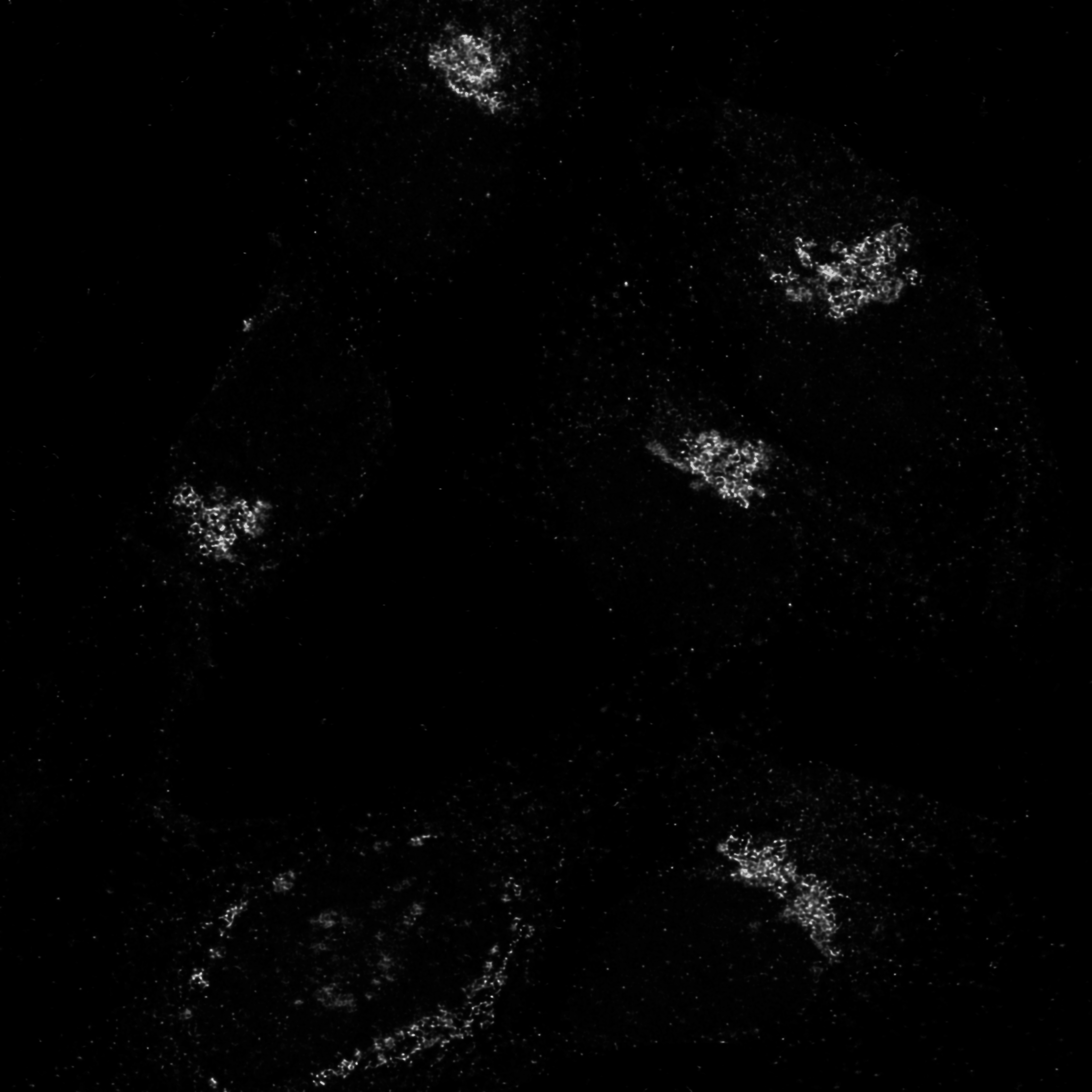

Supplement: Supplementary file 12 — Source data Fig. 7 [file 44319_2026_773_MOESM12_ESM.zip › Figure 7/Figure 7C/IF WT LYSET.tif]

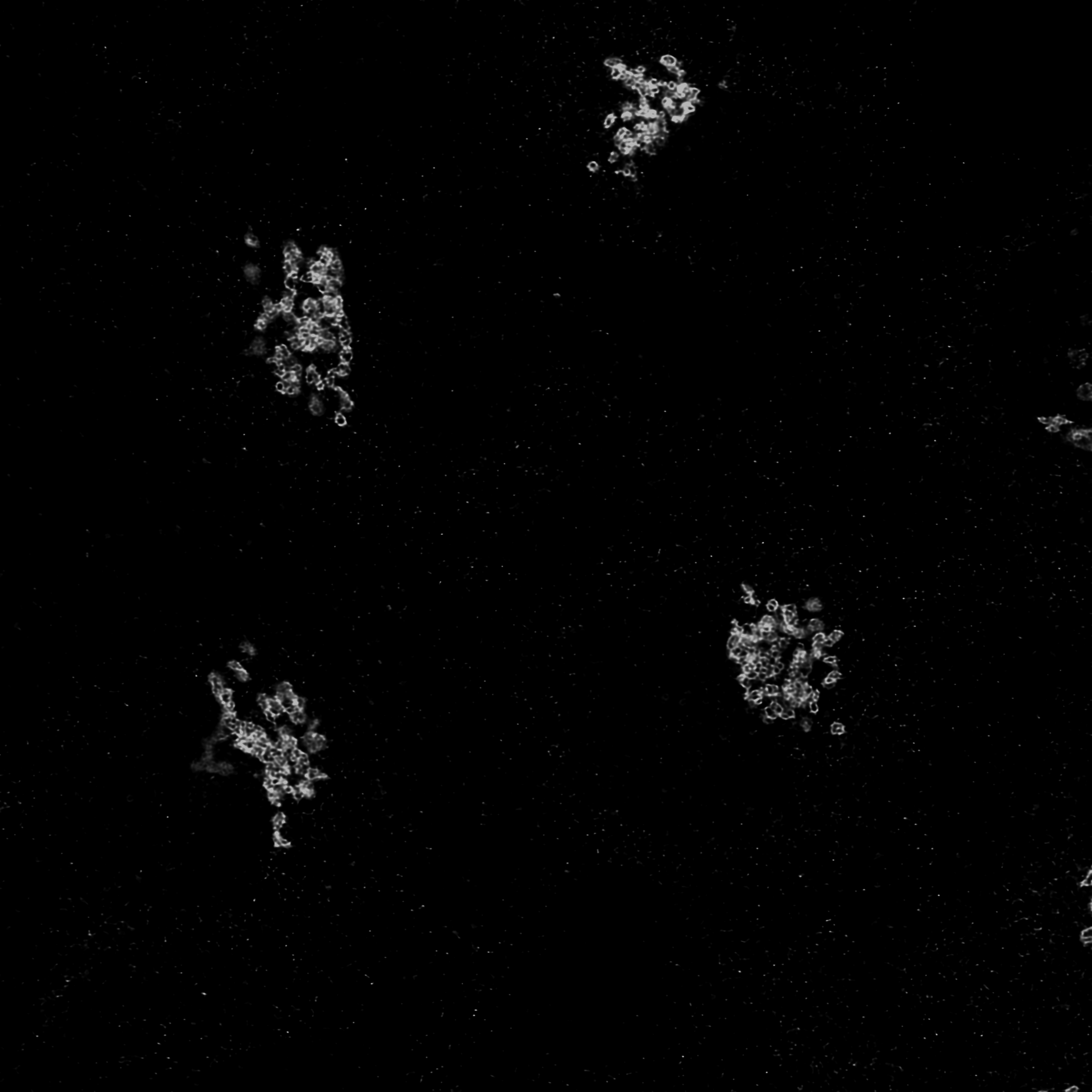

Supplement: Supplementary file 12 — Source data Fig. 7 [file 44319_2026_773_MOESM12_ESM.zip › Figure 7/Figure 7C/IF GRASP55KO GIANTIN.tif]

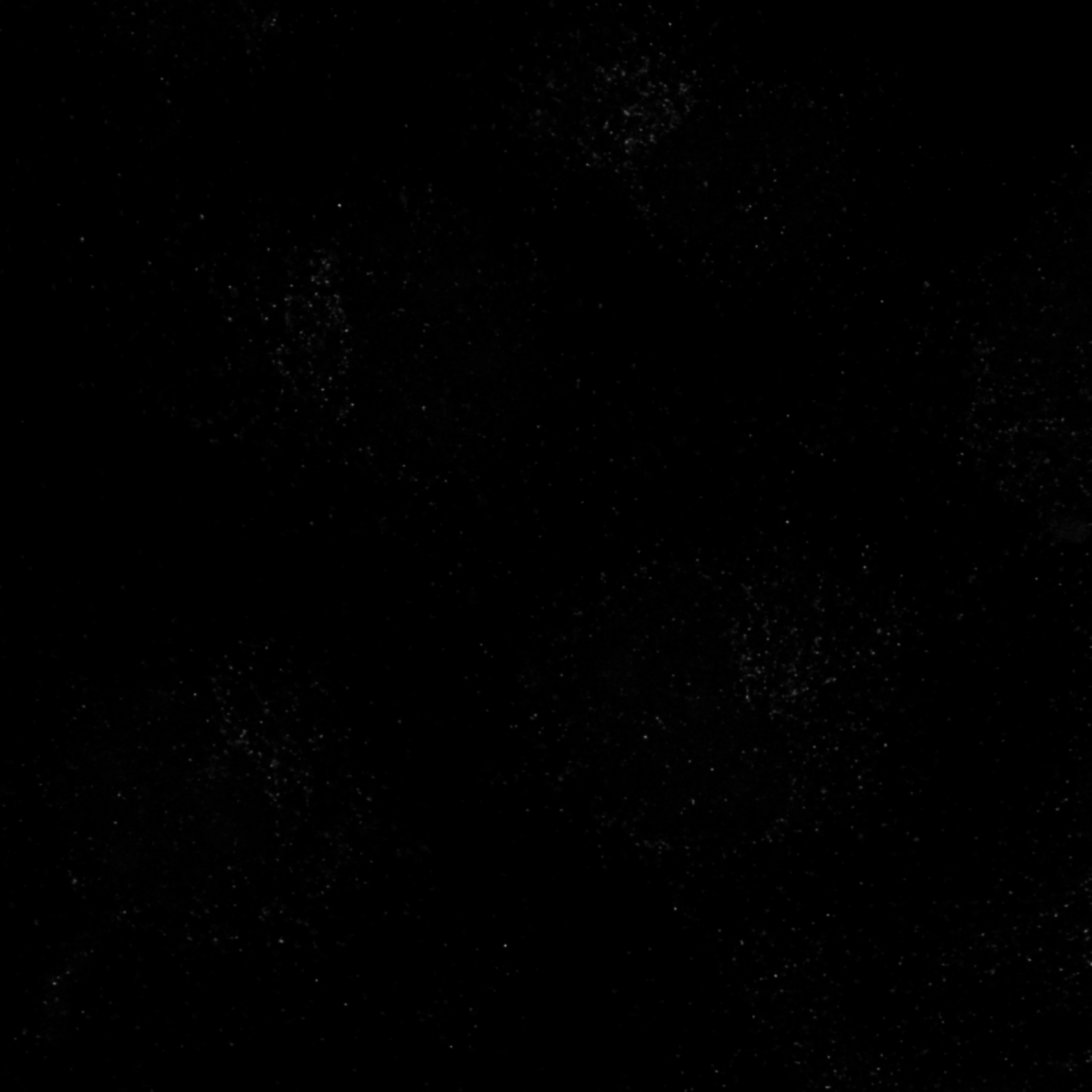

Supplement: Supplementary file 12 — Source data Fig. 7 [file 44319_2026_773_MOESM12_ESM.zip › Figure 7/Figure 7C/IF GRASP55KO LYSET.tif]

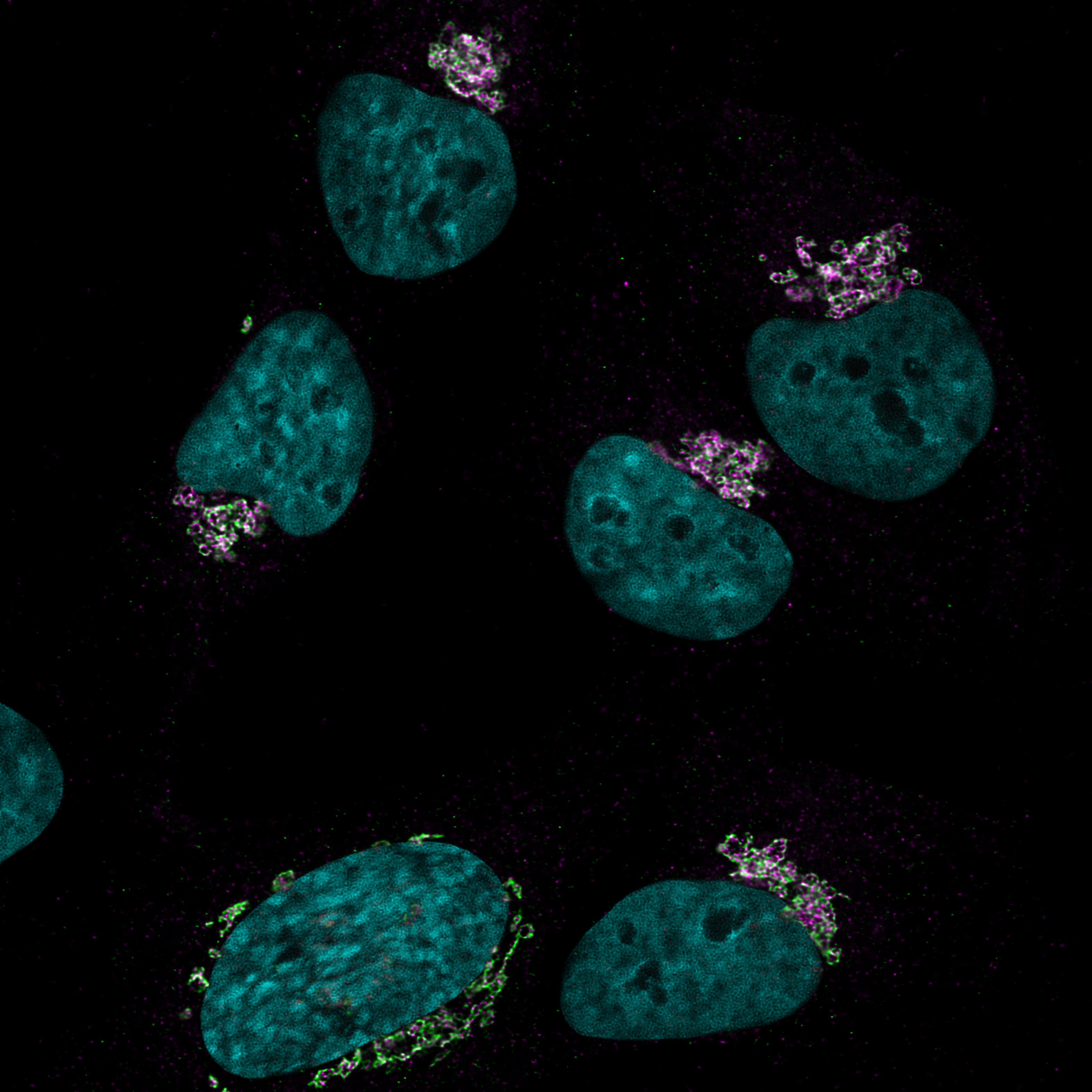

Supplement: Supplementary file 12 — Source data Fig. 7 [file 44319_2026_773_MOESM12_ESM.zip › Figure 7/Figure 7C/IF WT LYSET_GIANTIN MERGE.tif]

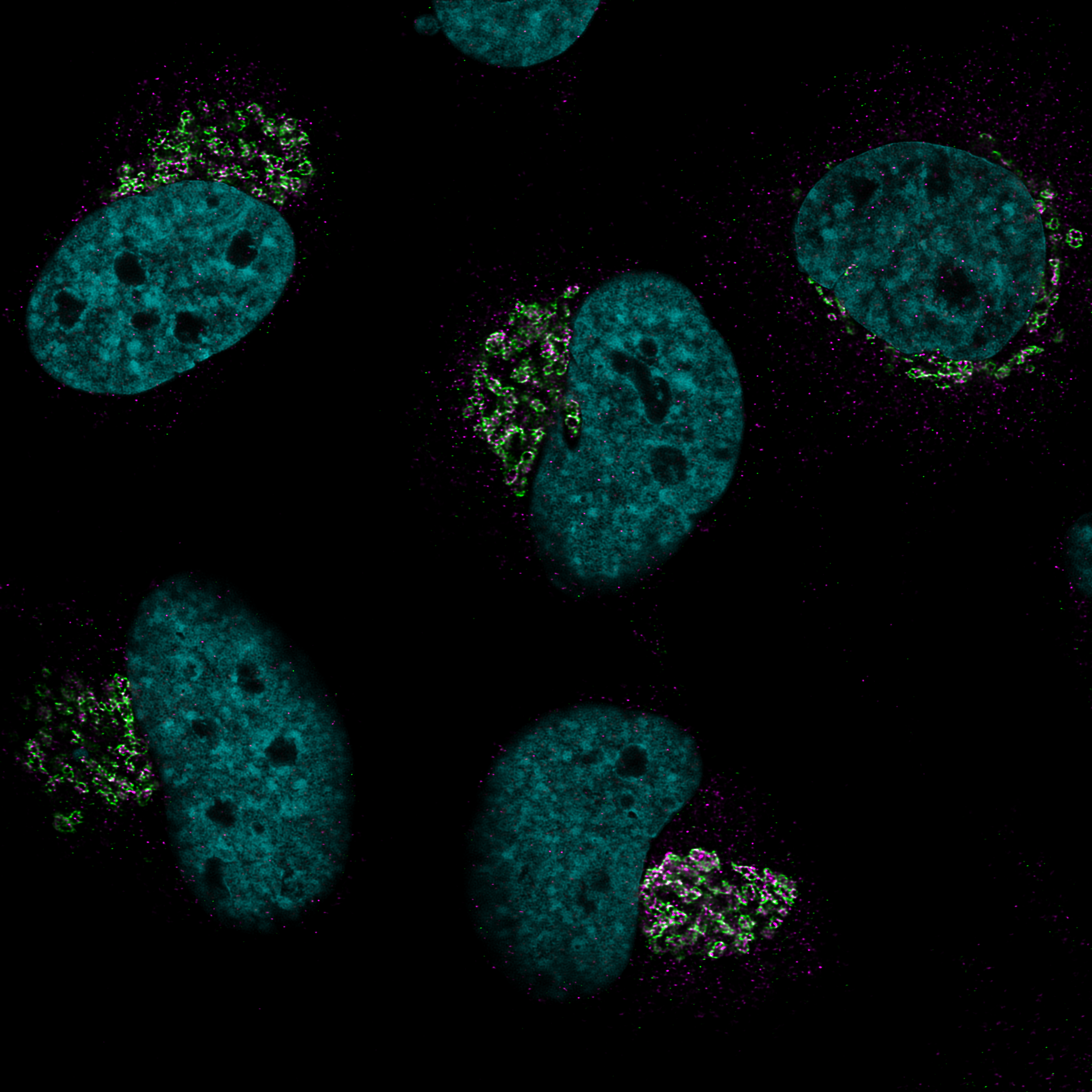

Supplement: Supplementary file 12 — Source data Fig. 7 [file 44319_2026_773_MOESM12_ESM.zip › Figure 7/Figure 7C/IF GRASP65KO LYSET_GIANTIN MERGE.tif]

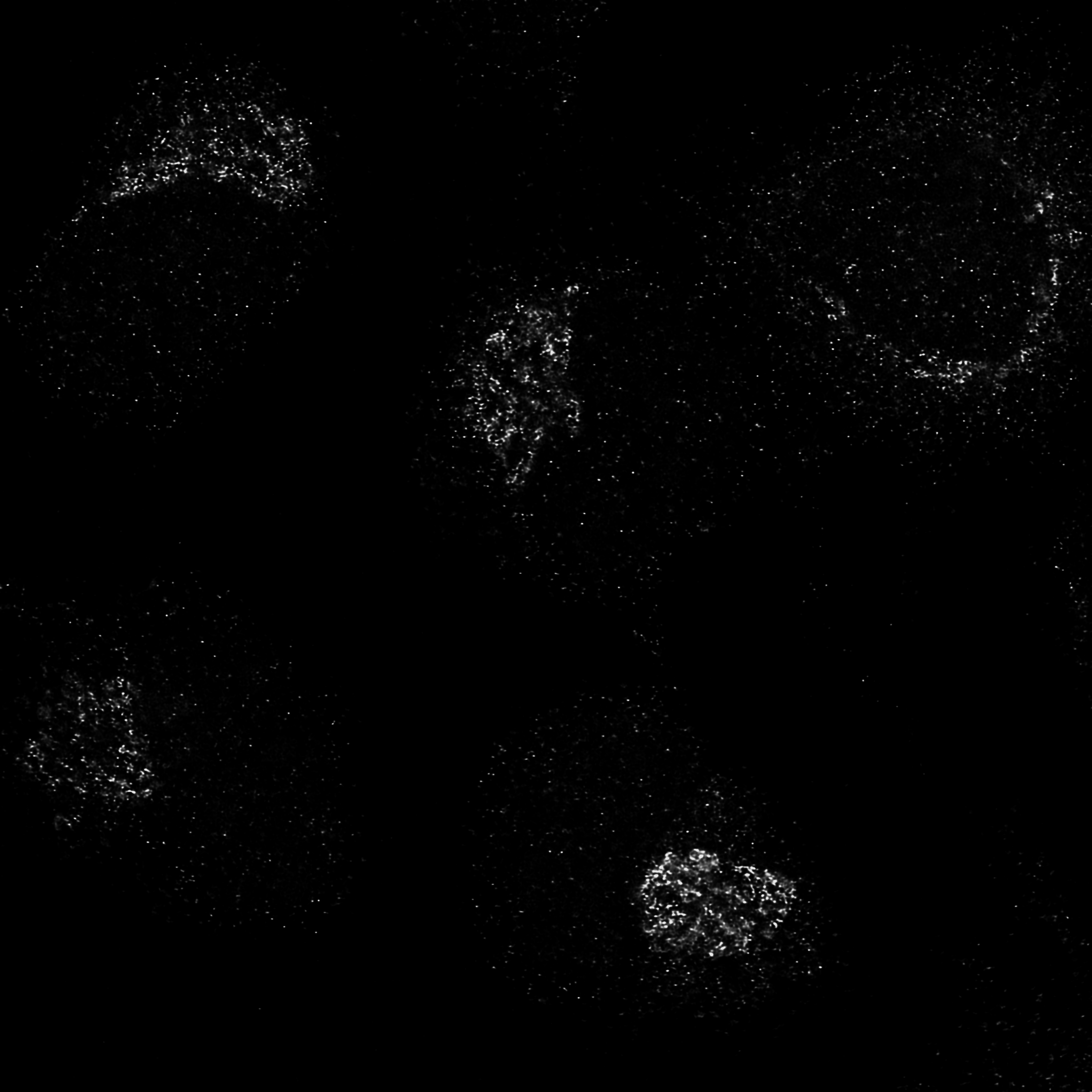

Supplement: Supplementary file 12 — Source data Fig. 7 [file 44319_2026_773_MOESM12_ESM.zip › Figure 7/Figure 7C/IF GRASP65KO LYSET.tif]

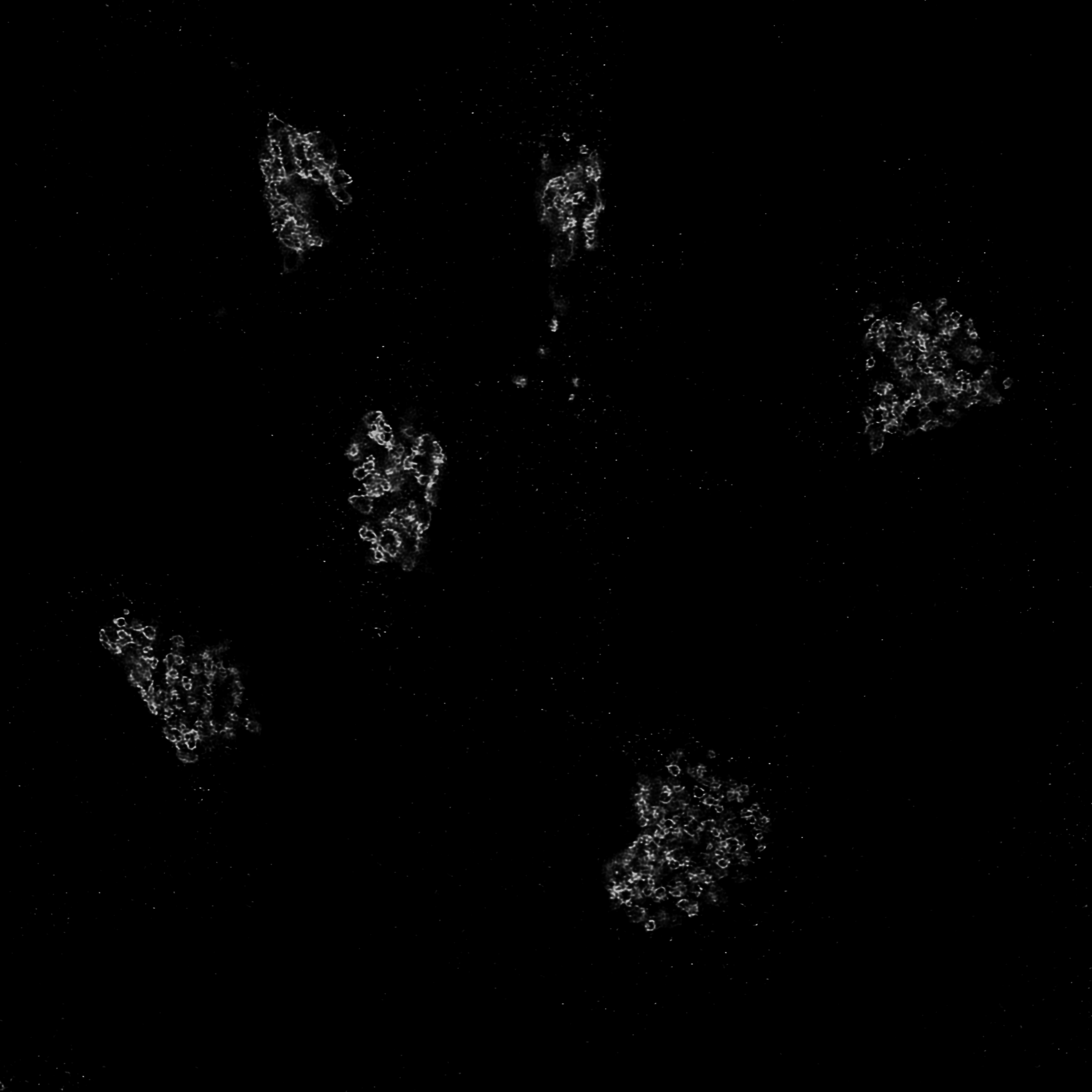

Supplement: Supplementary file 12 — Source data Fig. 7 [file 44319_2026_773_MOESM12_ESM.zip › Figure 7/Figure 7B/IF GRASP65KO GIANTIN.tif]

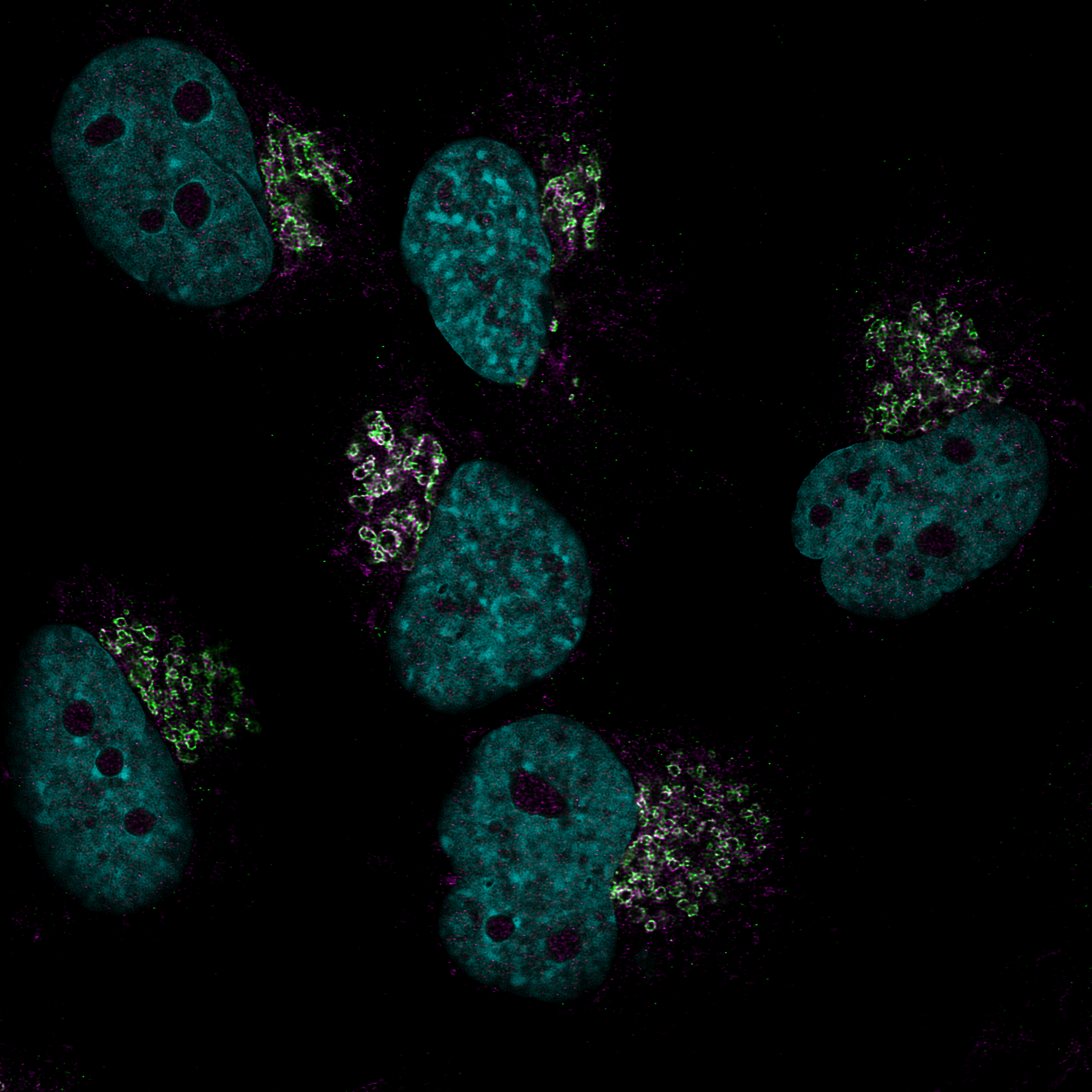

Supplement: Supplementary file 12 — Source data Fig. 7 [file 44319_2026_773_MOESM12_ESM.zip › Figure 7/Figure 7B/IF GRASP65KO GOLPH3_GIANTIN MERGE.tif]

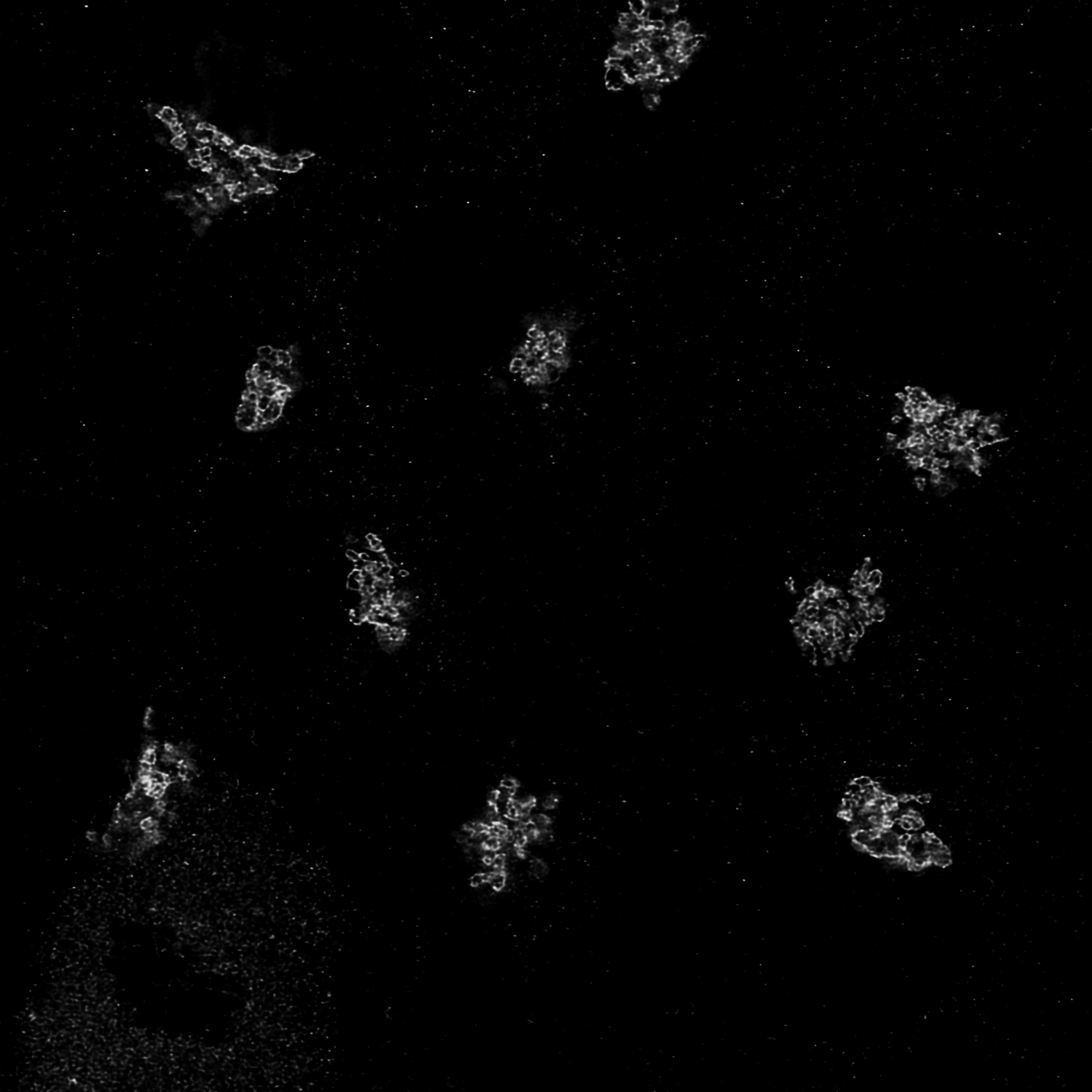

Supplement: Supplementary file 12 — Source data Fig. 7 [file 44319_2026_773_MOESM12_ESM.zip › Figure 7/Figure 7B/IF WT GIANTIN.tif]

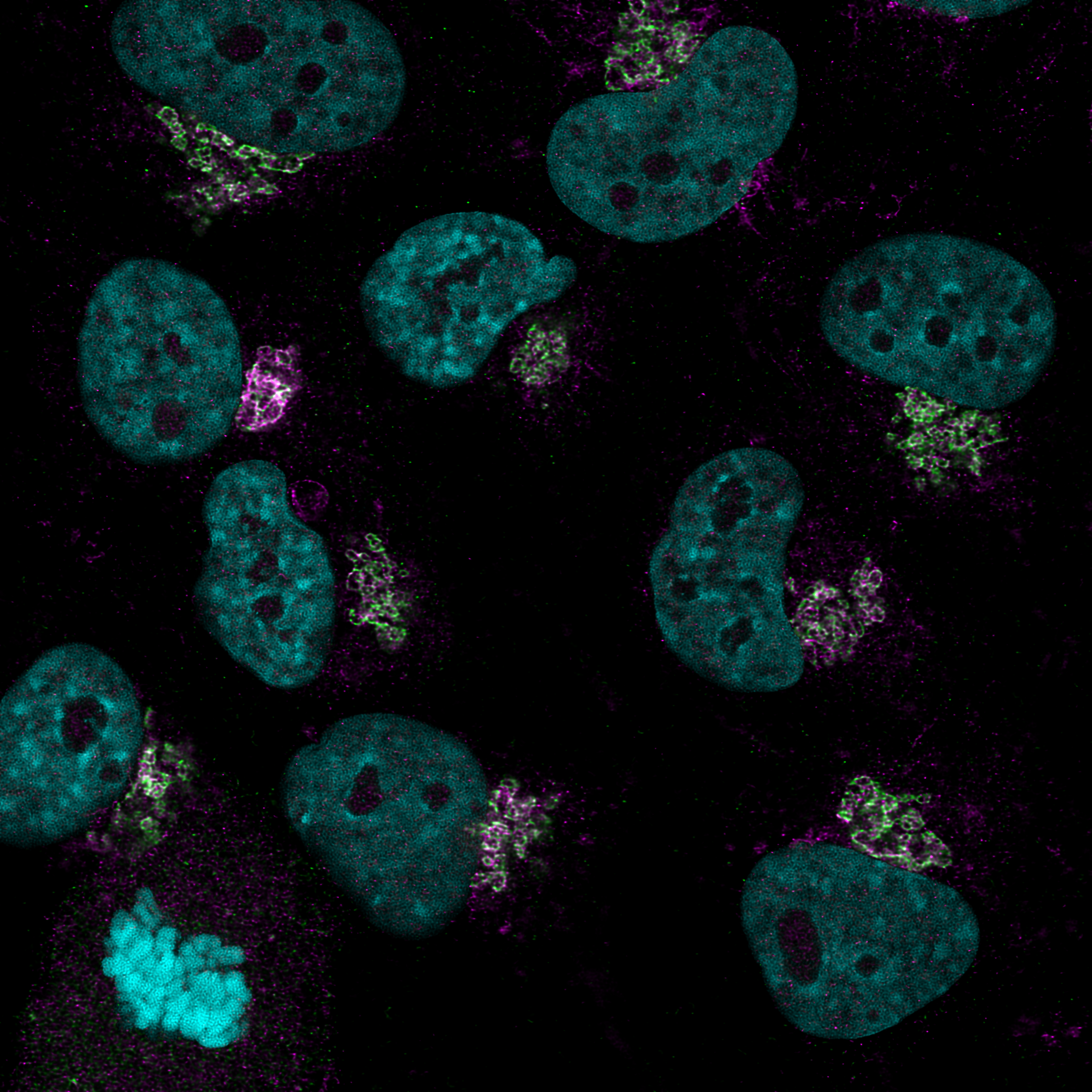

Supplement: Supplementary file 12 — Source data Fig. 7 [file 44319_2026_773_MOESM12_ESM.zip › Figure 7/Figure 7B/IF WT GOLPH3_GIANTIN MERGE.tif]

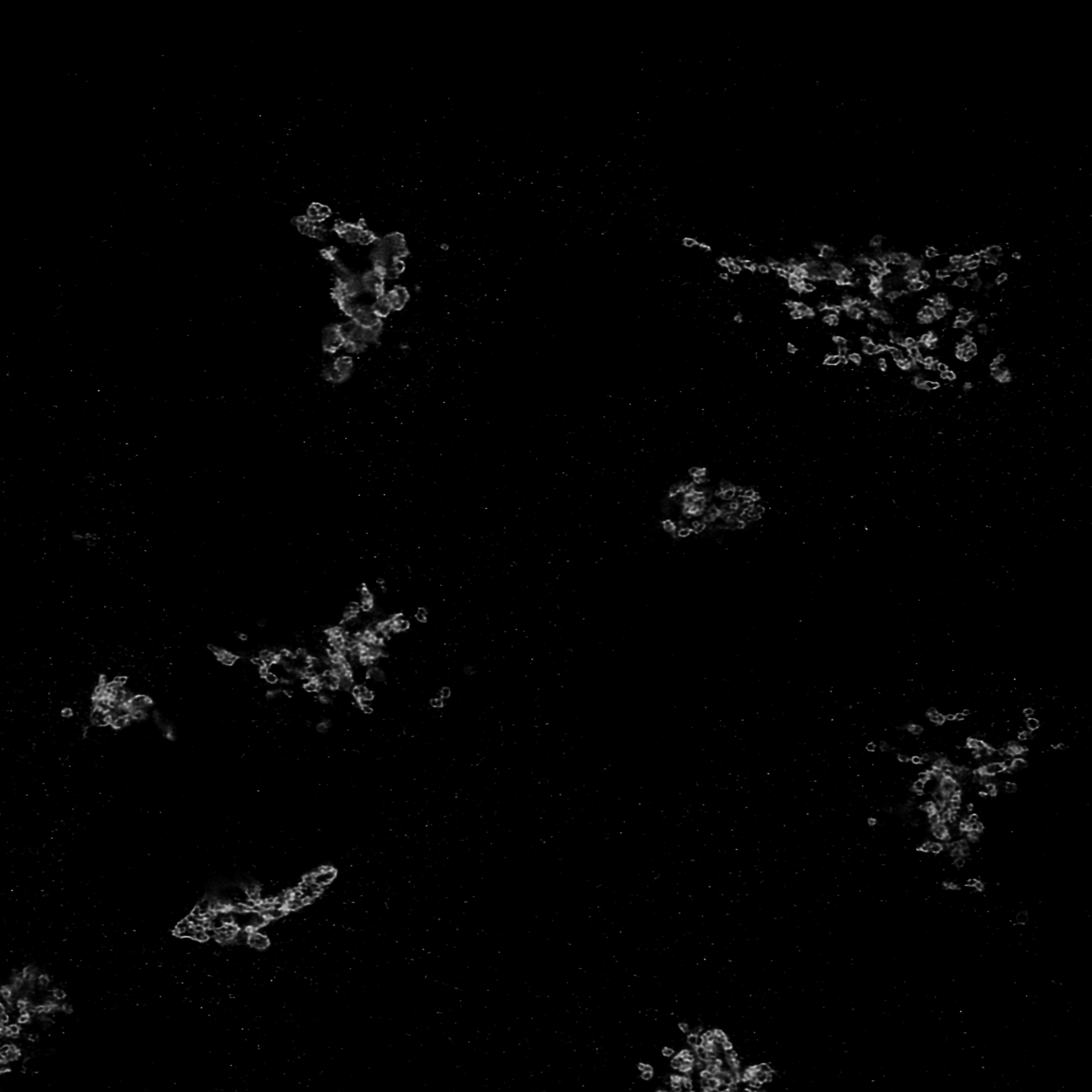

Supplement: Supplementary file 12 — Source data Fig. 7 [file 44319_2026_773_MOESM12_ESM.zip › Figure 7/Figure 7B/IF GRASP55KO GIANTIN.tif]

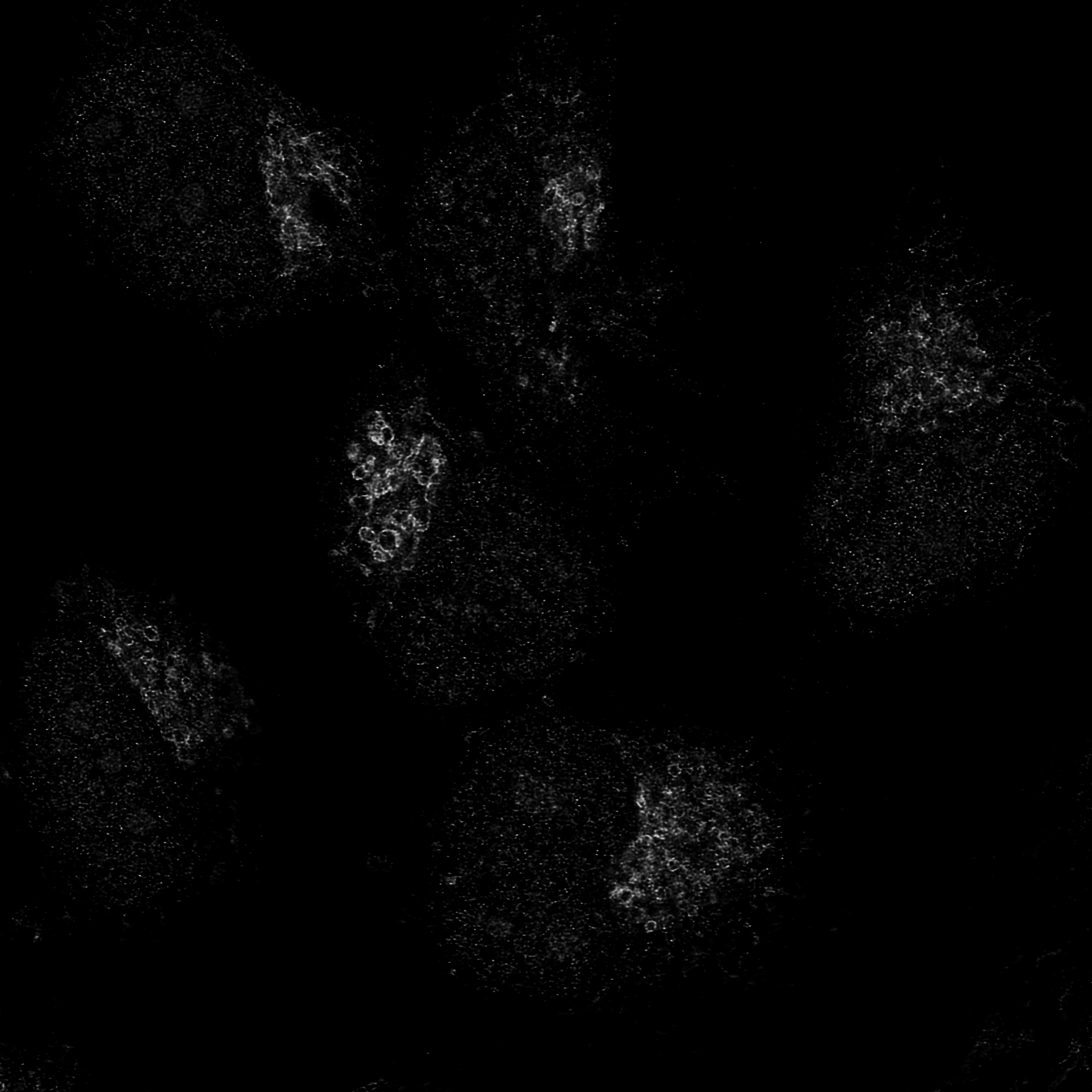

Supplement: Supplementary file 12 — Source data Fig. 7 [file 44319_2026_773_MOESM12_ESM.zip › Figure 7/Figure 7B/IF GRASP65KO GOLPH3tif.tif]

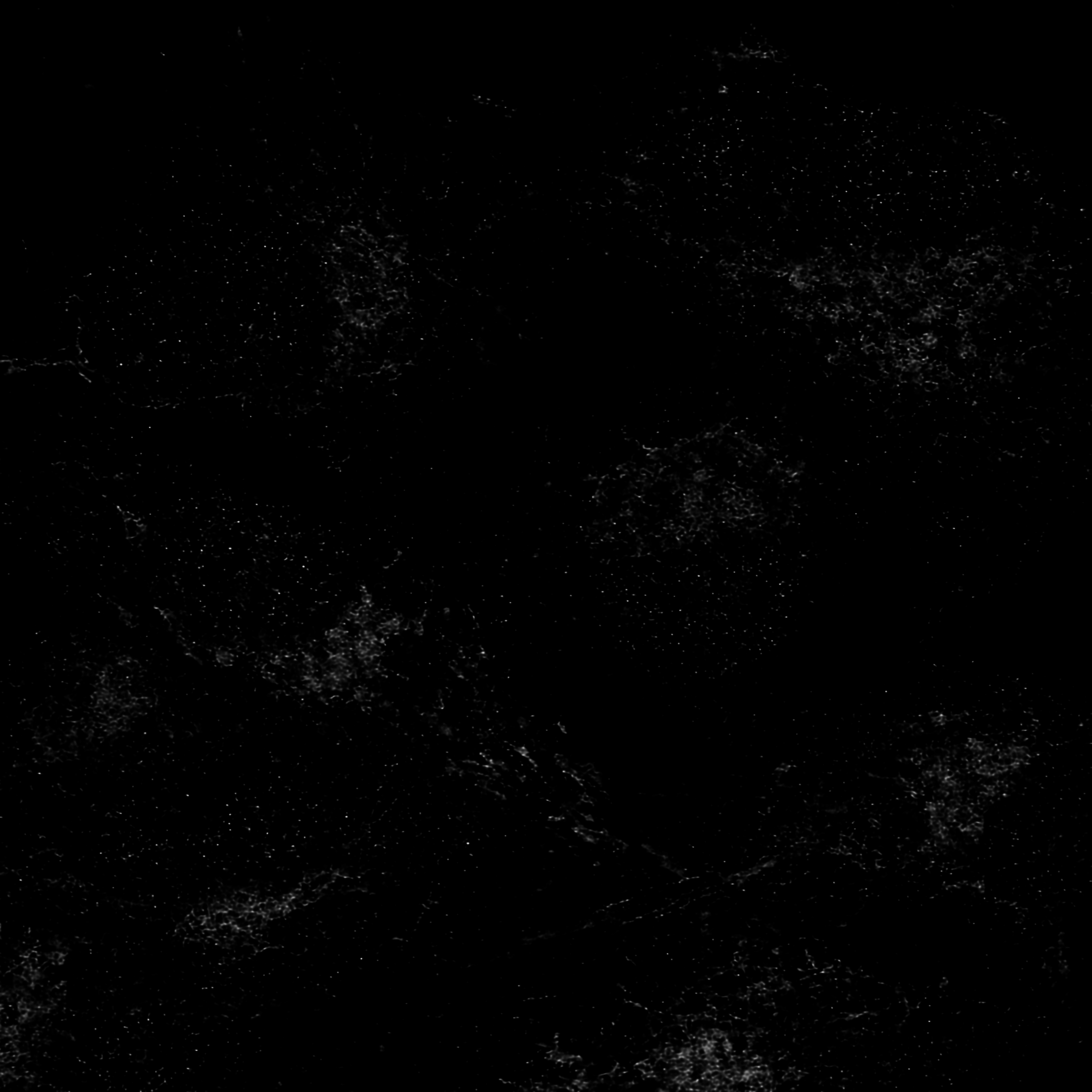

Supplement: Supplementary file 12 — Source data Fig. 7 [file 44319_2026_773_MOESM12_ESM.zip › Figure 7/Figure 7B/IF GRASP55KO GOLPH3.tif]

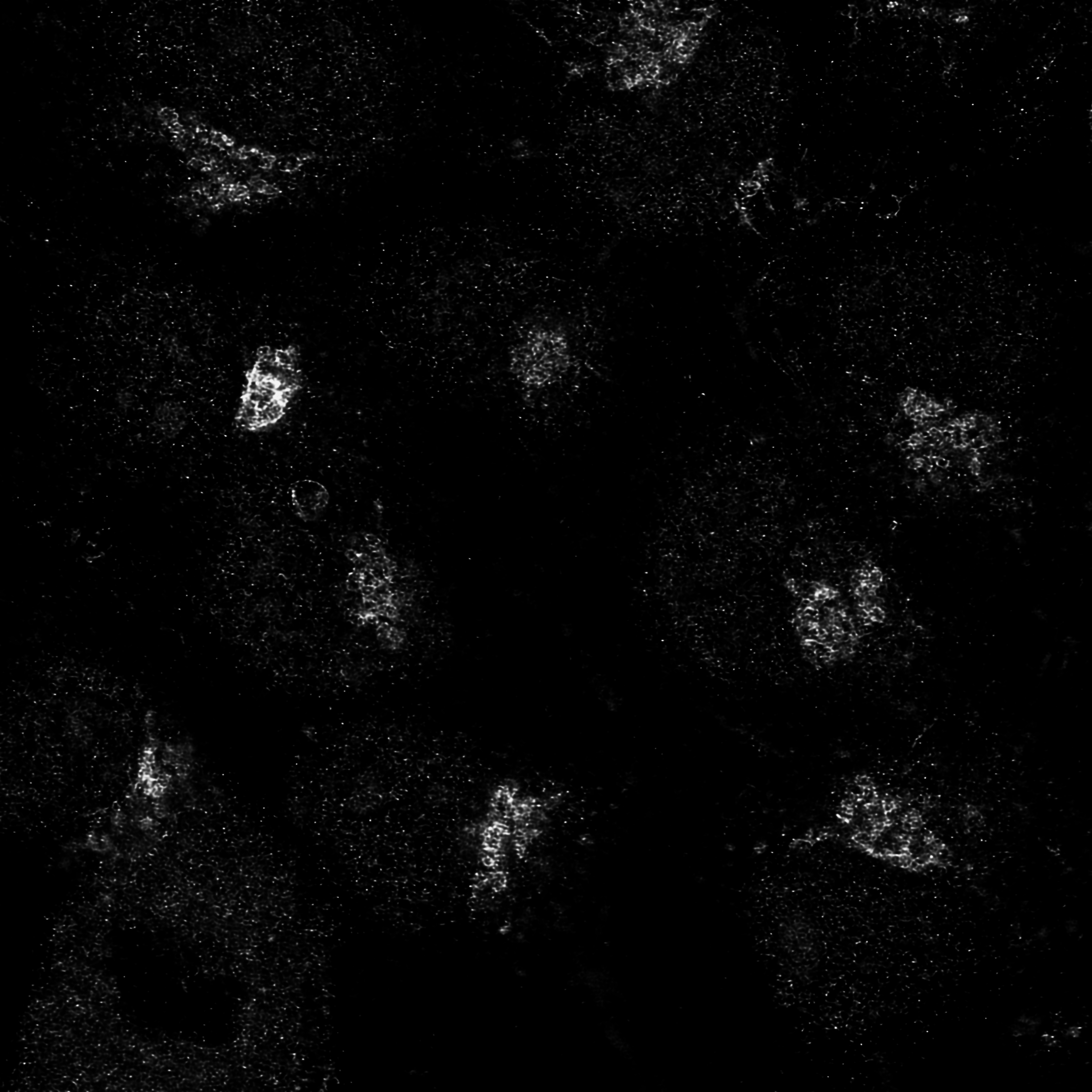

Supplement: Supplementary file 12 — Source data Fig. 7 [file 44319_2026_773_MOESM12_ESM.zip › Figure 7/Figure 7B/IF WT GOLPH3.tif]

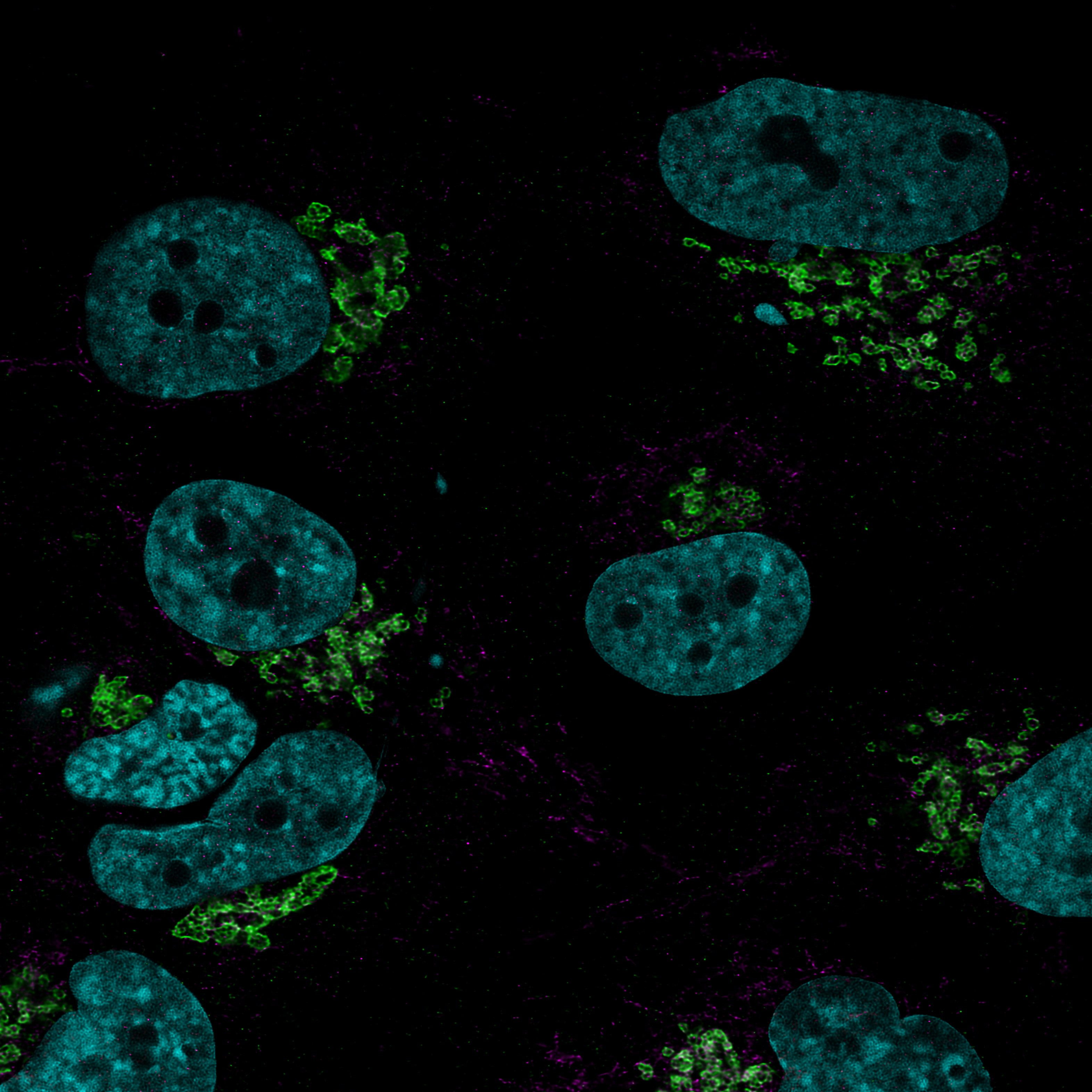

Supplement: Supplementary file 12 — Source data Fig. 7 [file 44319_2026_773_MOESM12_ESM.zip › Figure 7/Figure 7B/IF GRASP55KO GOLPH3_GIANTIN MERGE.tif]

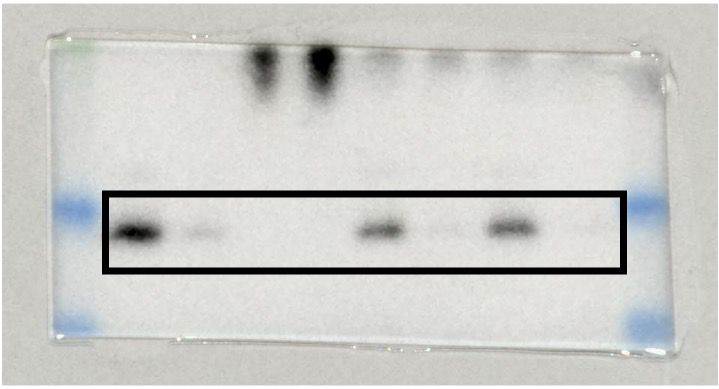

Supplement: Supplementary file 12 — Source data Fig. 7 [file 44319_2026_773_MOESM12_ESM.zip › Figure 7/Figure 7E/Western LYSET.tif]

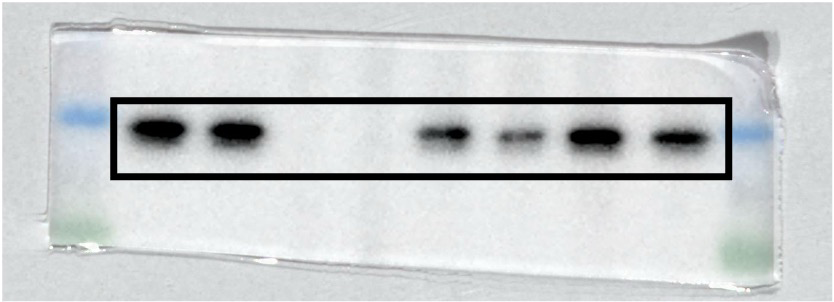

Supplement: Supplementary file 12 — Source data Fig. 7 [file 44319_2026_773_MOESM12_ESM.zip › Figure 7/Figure 7E/Western GOLPH3.tif]

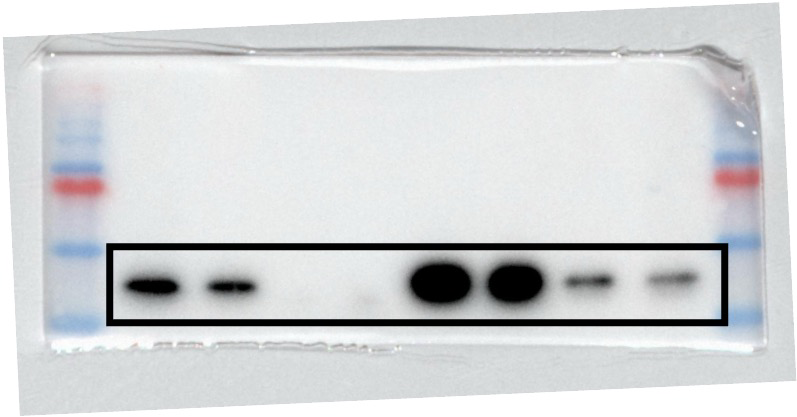

Supplement: Supplementary file 12 — Source data Fig. 7 [file 44319_2026_773_MOESM12_ESM.zip › Figure 7/Figure 7E/Western GRASP55.tif]

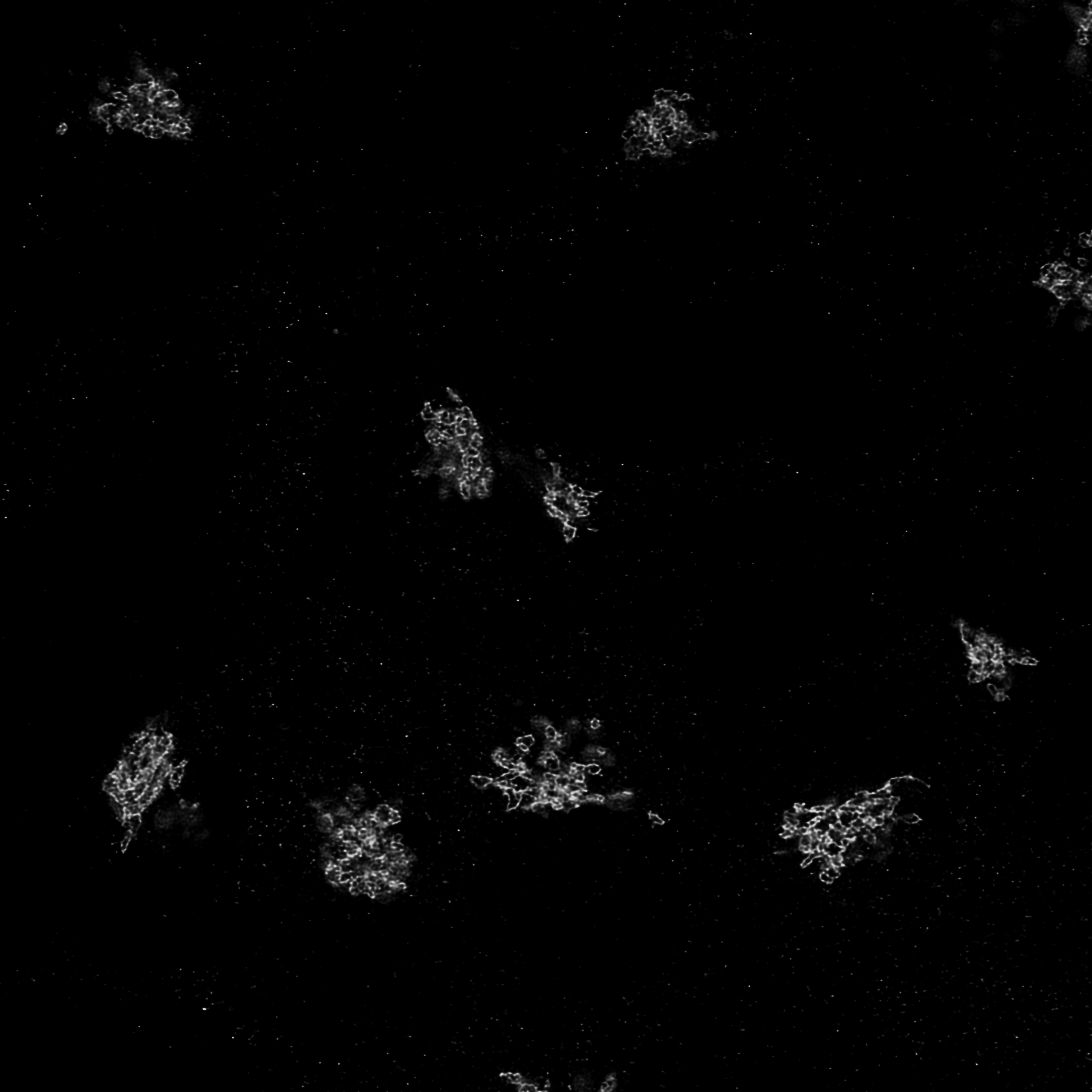

Supplement: Supplementary file 12 — Source data Fig. 7 [file 44319_2026_773_MOESM12_ESM.zip › Figure 7/Figure 7I/IF WT GIANTIN.tif]

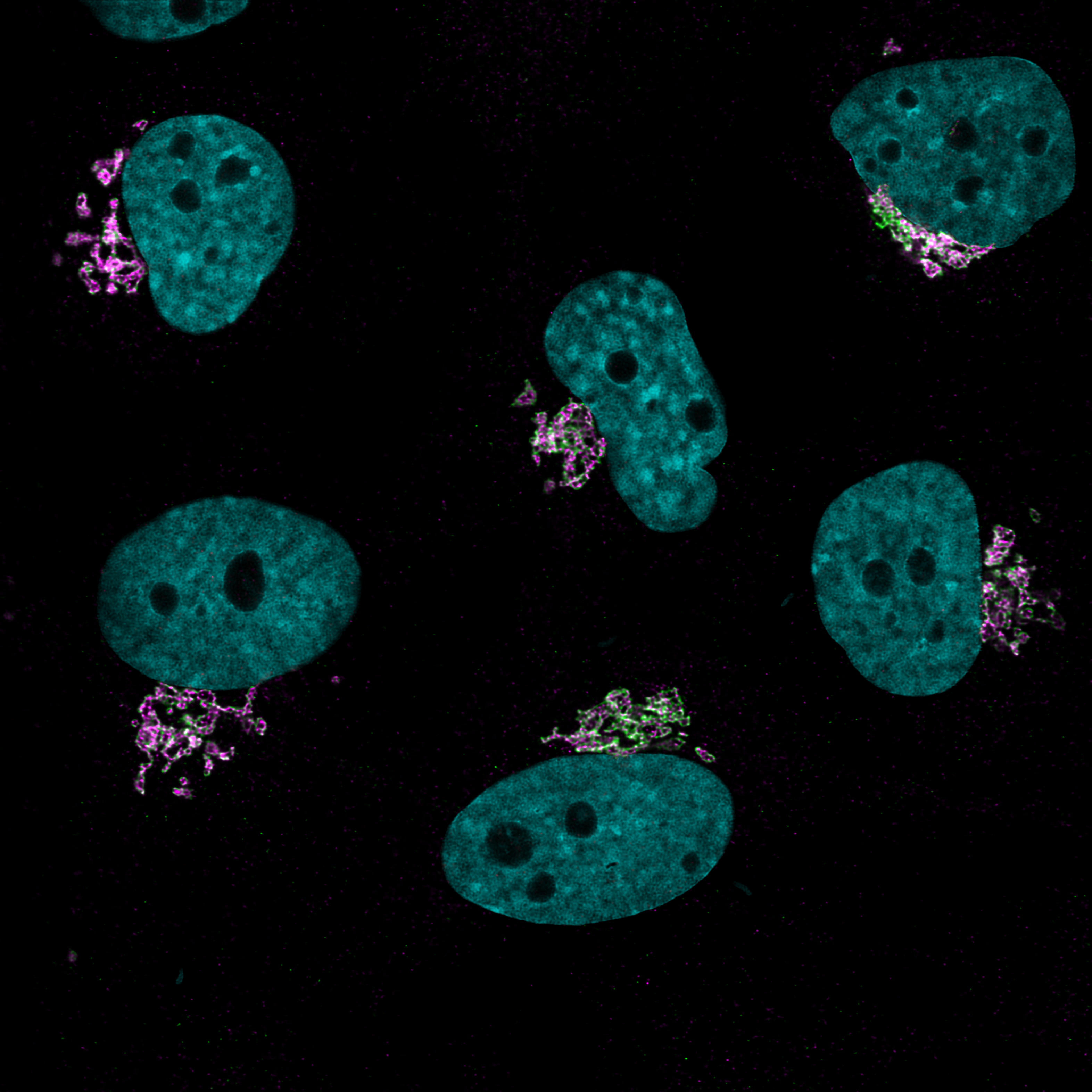

Supplement: Supplementary file 12 — Source data Fig. 7 [file 44319_2026_773_MOESM12_ESM.zip › Figure 7/Figure 7I/IF GRASP55KO+WT LYSET_GIANTIN MERGE.tif]

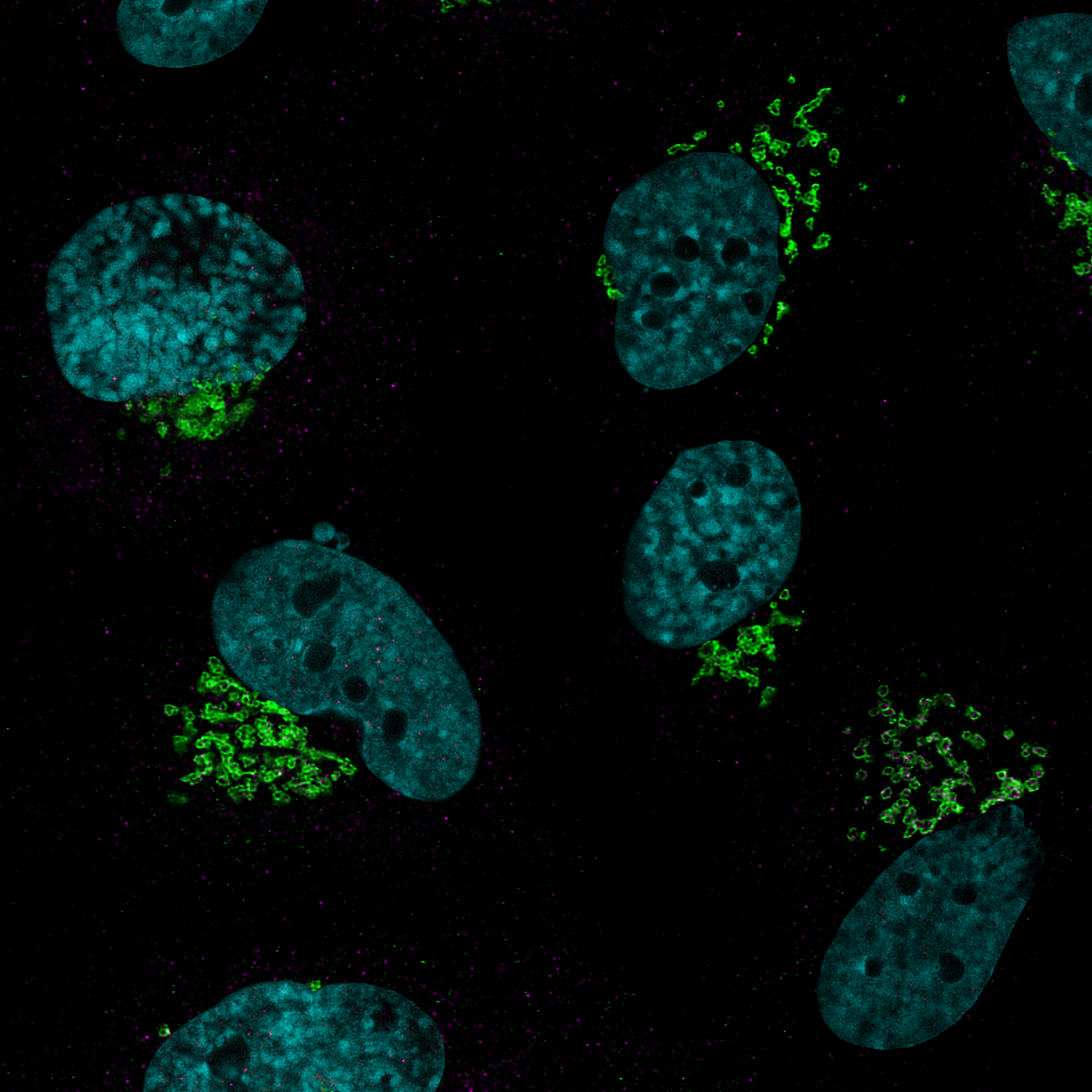

Supplement: Supplementary file 12 — Source data Fig. 7 [file 44319_2026_773_MOESM12_ESM.zip › Figure 7/Figure 7I/IF GRASP55KO LYSET_GIANTIN MERGE.tif]

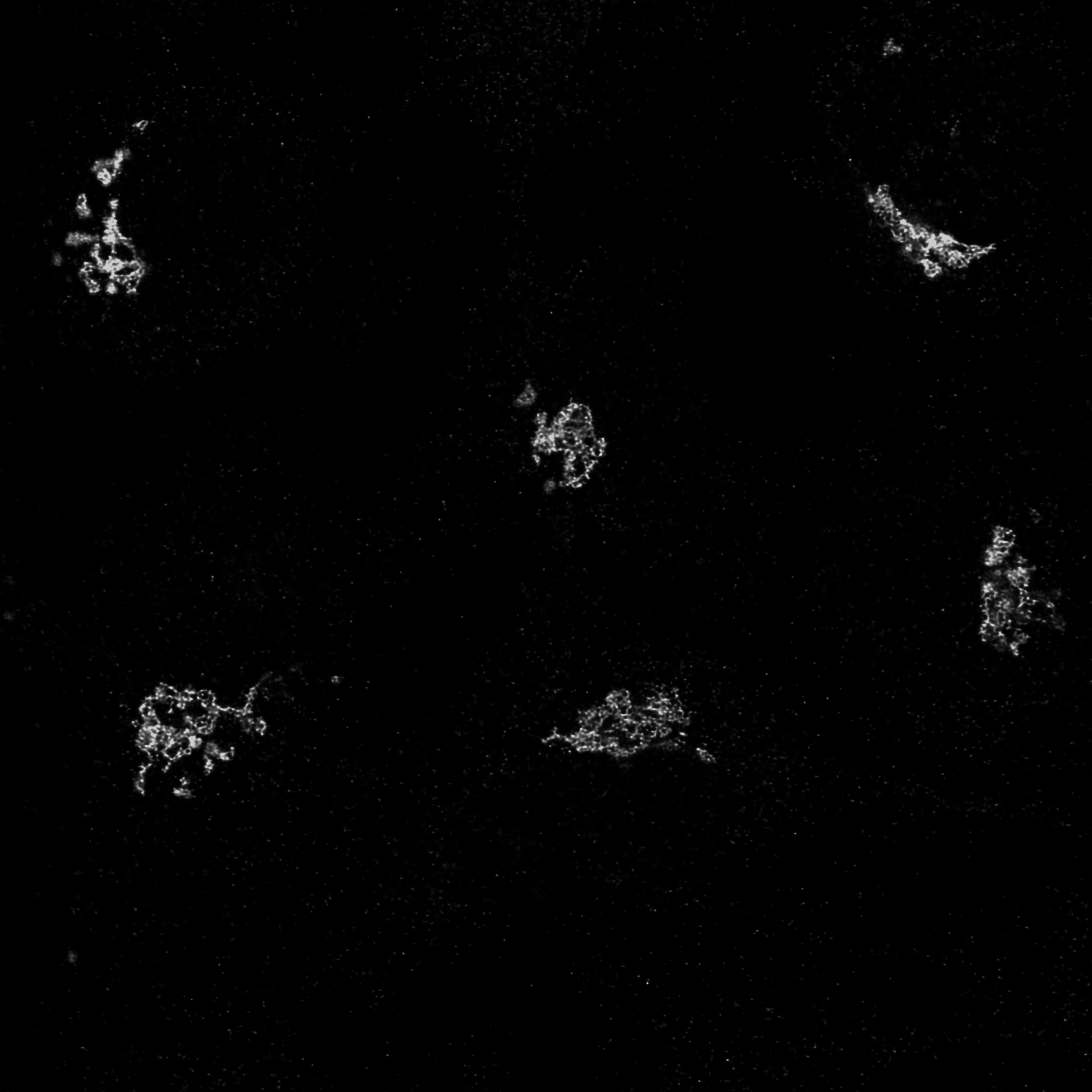

Supplement: Supplementary file 12 — Source data Fig. 7 [file 44319_2026_773_MOESM12_ESM.zip › Figure 7/Figure 7I/IF GRASP55KO+WT LYSET.tif]

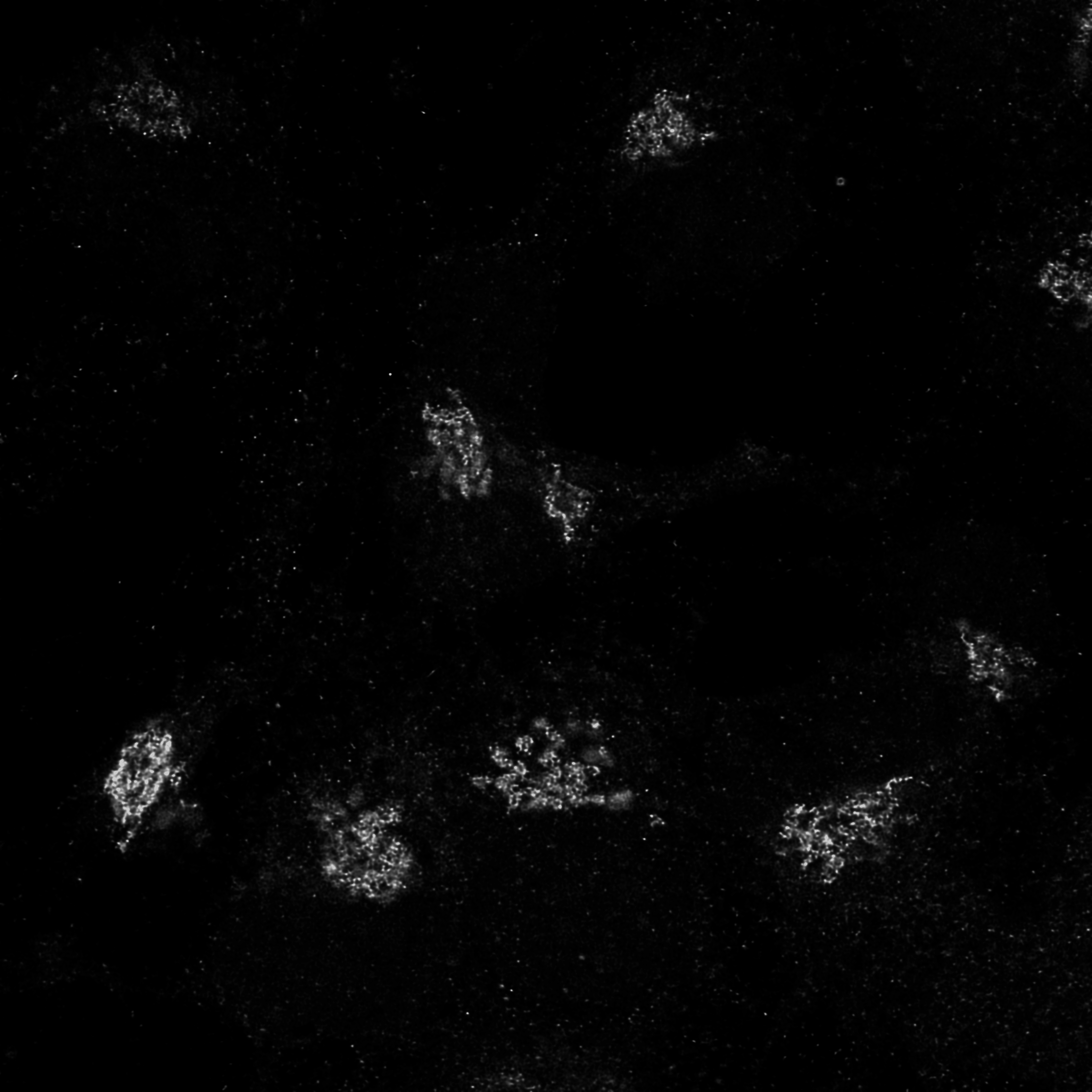

Supplement: Supplementary file 12 — Source data Fig. 7 [file 44319_2026_773_MOESM12_ESM.zip › Figure 7/Figure 7I/IF WT LYSET.tif]

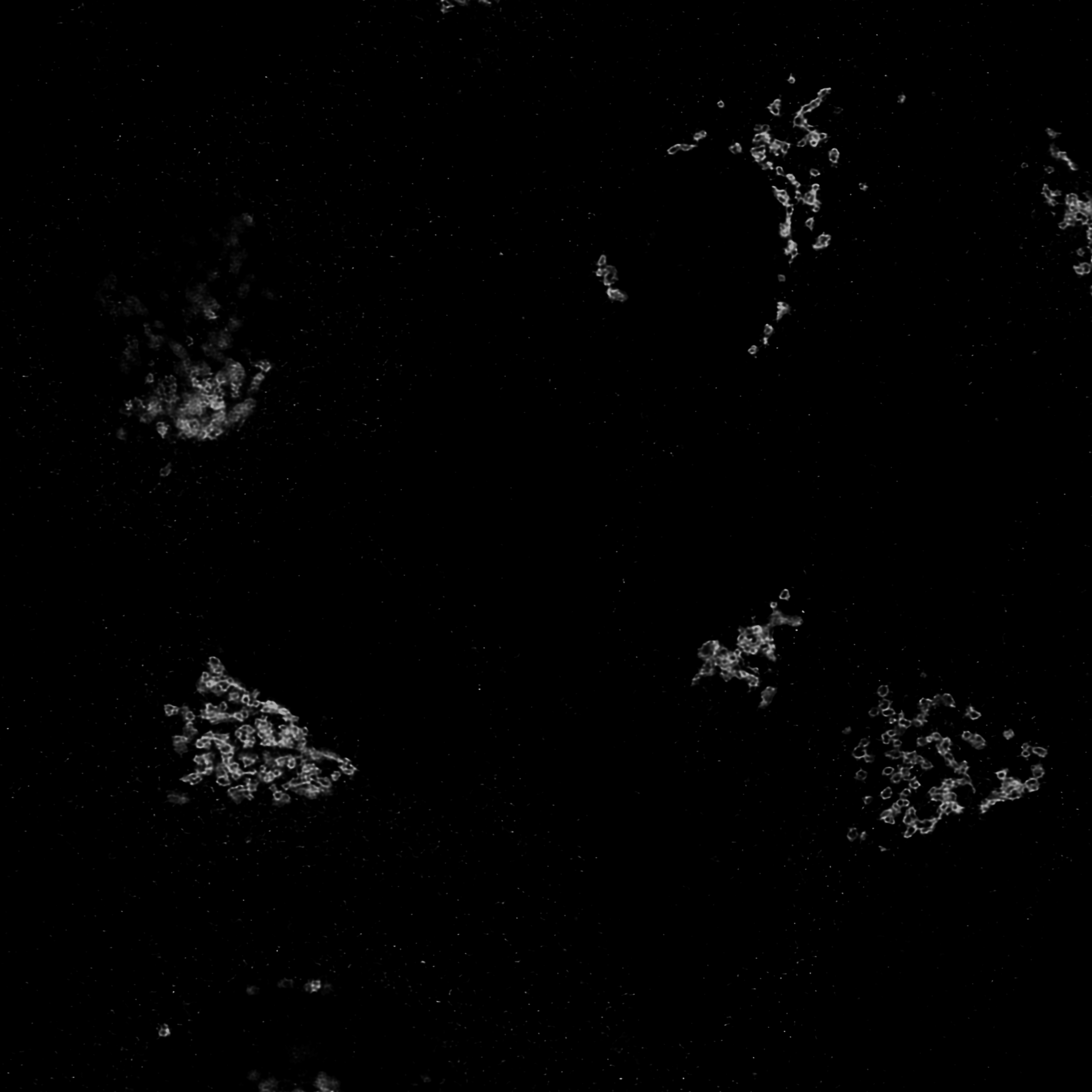

Supplement: Supplementary file 12 — Source data Fig. 7 [file 44319_2026_773_MOESM12_ESM.zip › Figure 7/Figure 7I/IF GRASP55KO GIANTIN.tif]

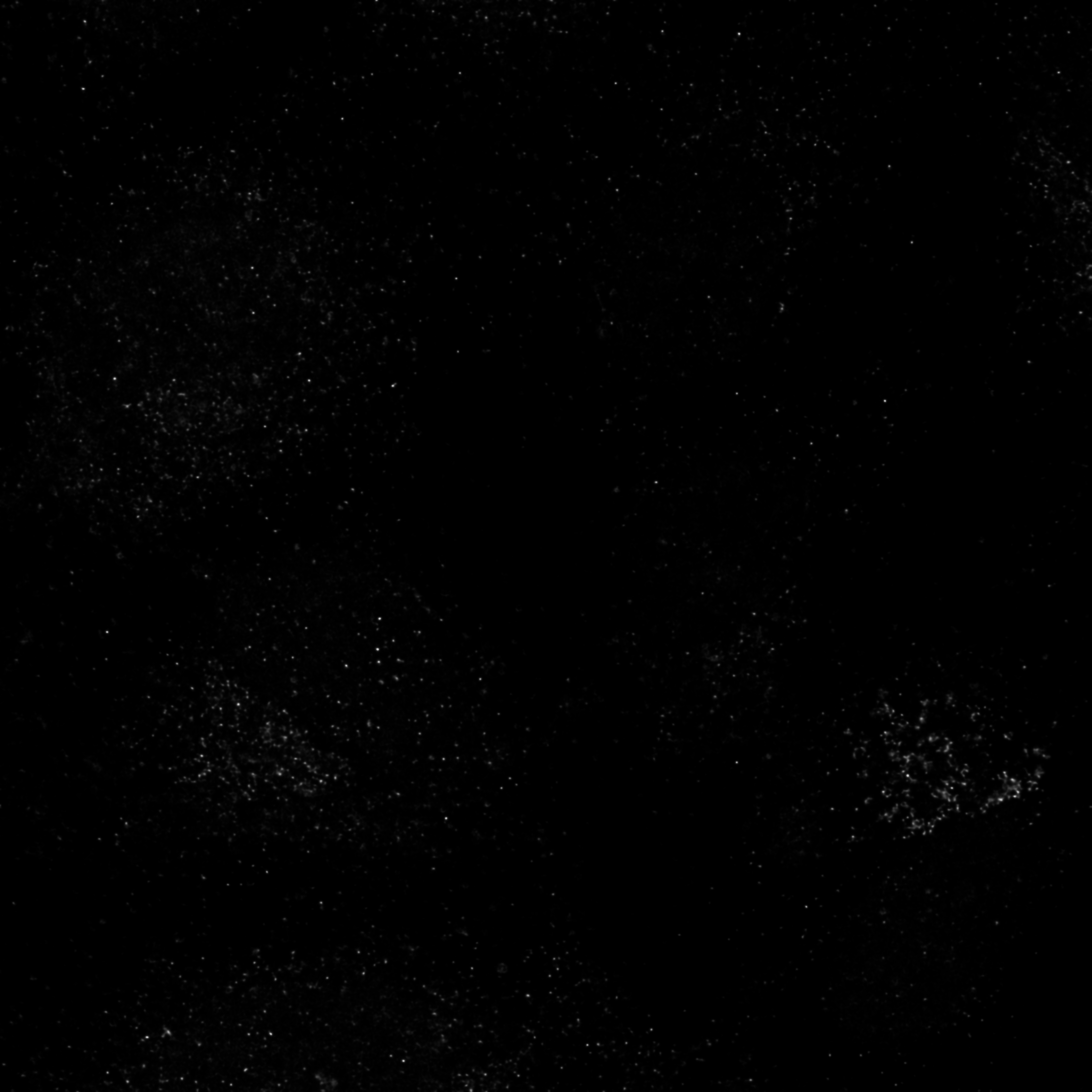

Supplement: Supplementary file 12 — Source data Fig. 7 [file 44319_2026_773_MOESM12_ESM.zip › Figure 7/Figure 7I/IF GRASP55KO LYSET.tif]

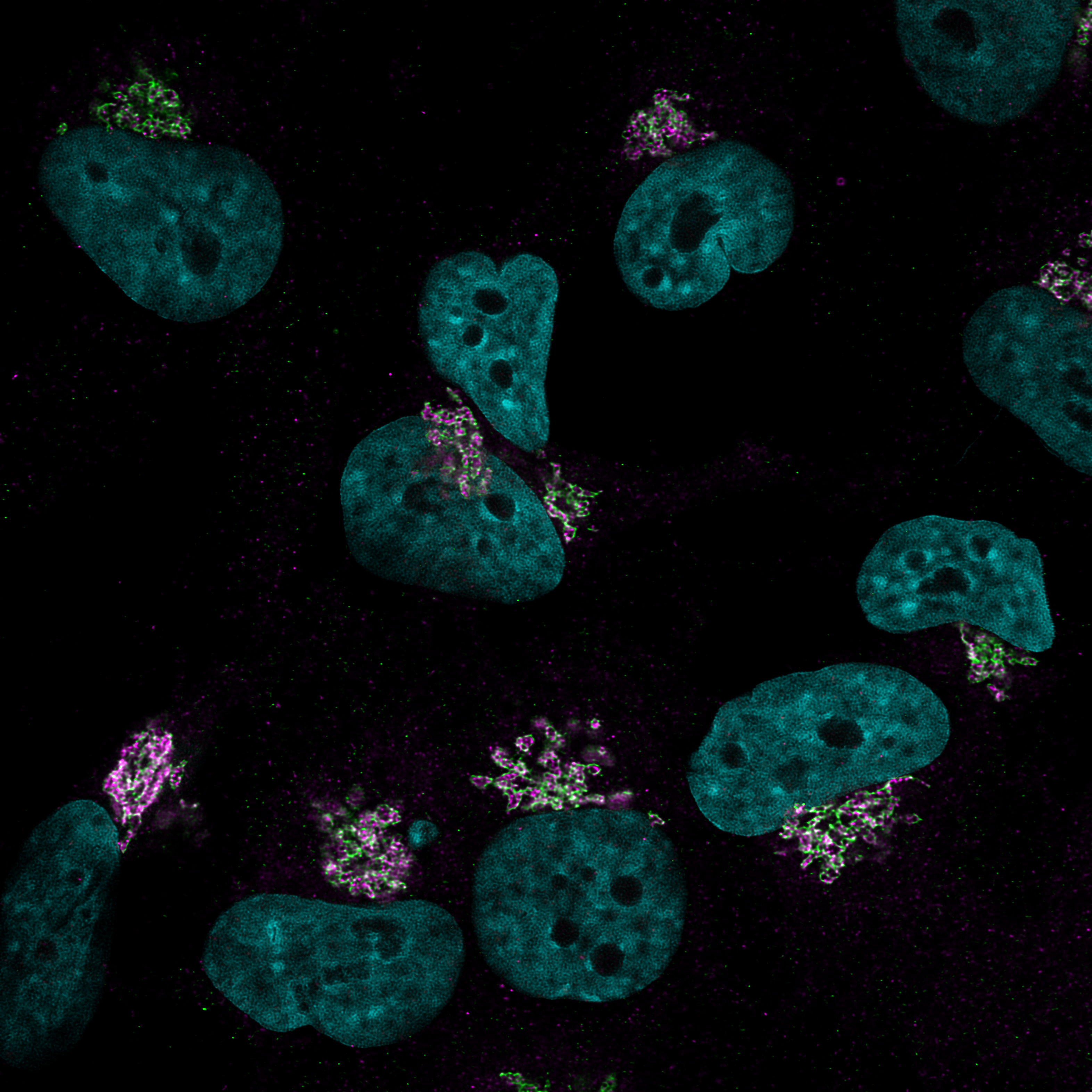

Supplement: Supplementary file 12 — Source data Fig. 7 [file 44319_2026_773_MOESM12_ESM.zip › Figure 7/Figure 7I/IF WT LYSET_GIANTIN MERGE.tif]

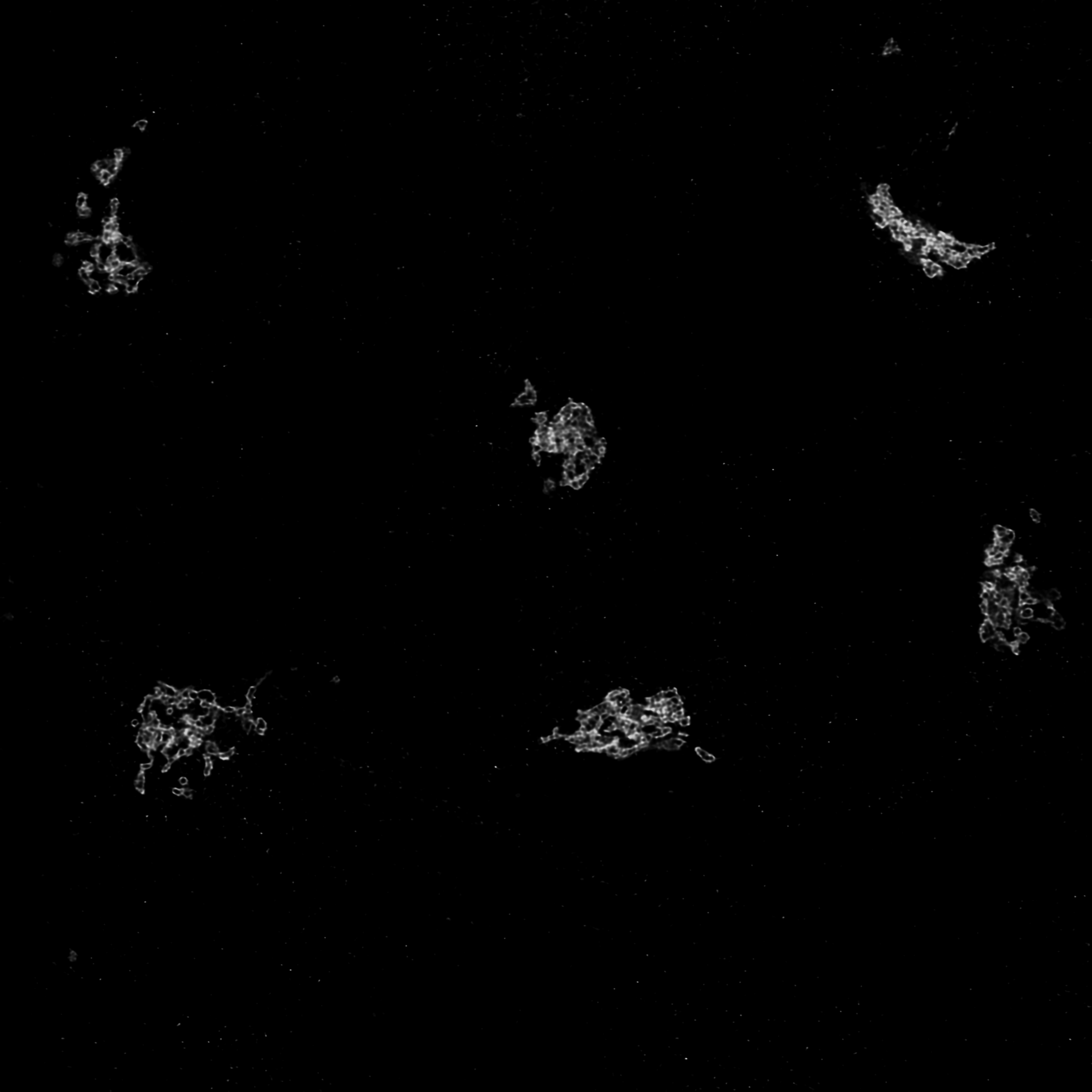

Supplement: Supplementary file 12 — Source data Fig. 7 [file 44319_2026_773_MOESM12_ESM.zip › Figure 7/Figure 7I/IF GRASP55KO+WT GIANTIN.tif]

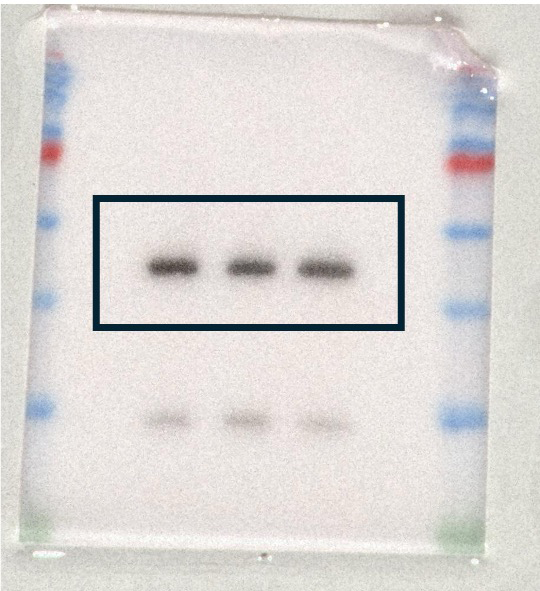

Supplement: Supplementary file 12 — Source data Fig. 7 [file 44319_2026_773_MOESM12_ESM.zip › Figure 7/Figure 7G/Western Tubulin.tif]

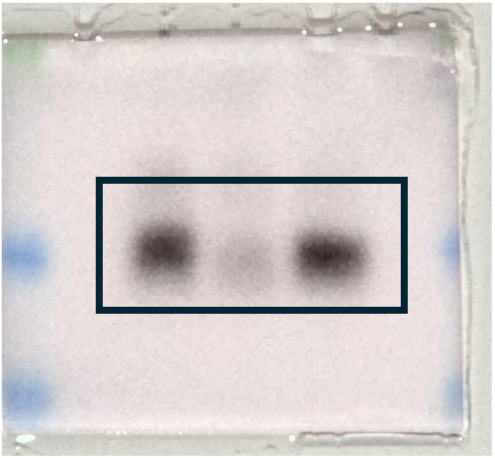

Supplement: Supplementary file 12 — Source data Fig. 7 [file 44319_2026_773_MOESM12_ESM.zip › Figure 7/Figure 7G/Western LYSET.tif]

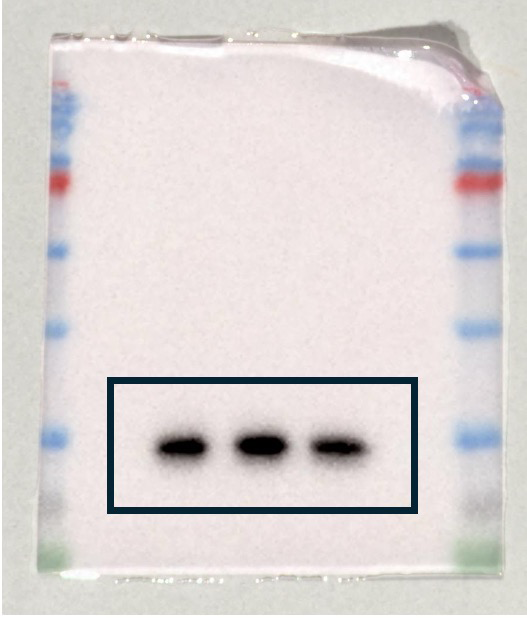

Supplement: Supplementary file 12 — Source data Fig. 7 [file 44319_2026_773_MOESM12_ESM.zip › Figure 7/Figure 7G/Western GOLPH3.tif]

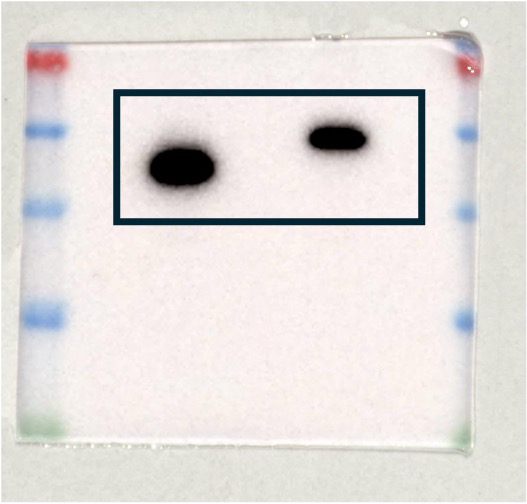

Supplement: Supplementary file 12 — Source data Fig. 7 [file 44319_2026_773_MOESM12_ESM.zip › Figure 7/Figure 7G/Western GRASP55.tif]

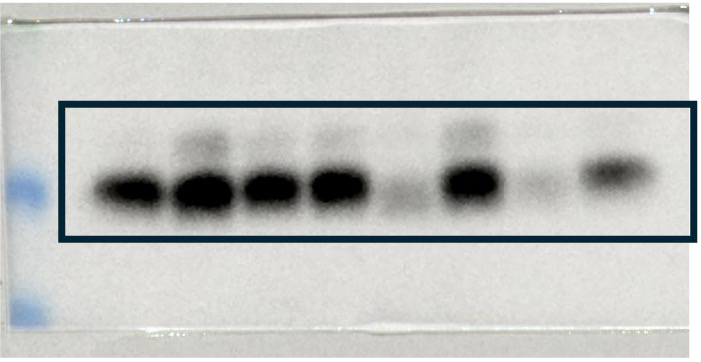

Supplement: Supplementary file 12 — Source data Fig. 7 [file 44319_2026_773_MOESM12_ESM.zip › Figure 7/Figure 7F/Western LYSET.tif]

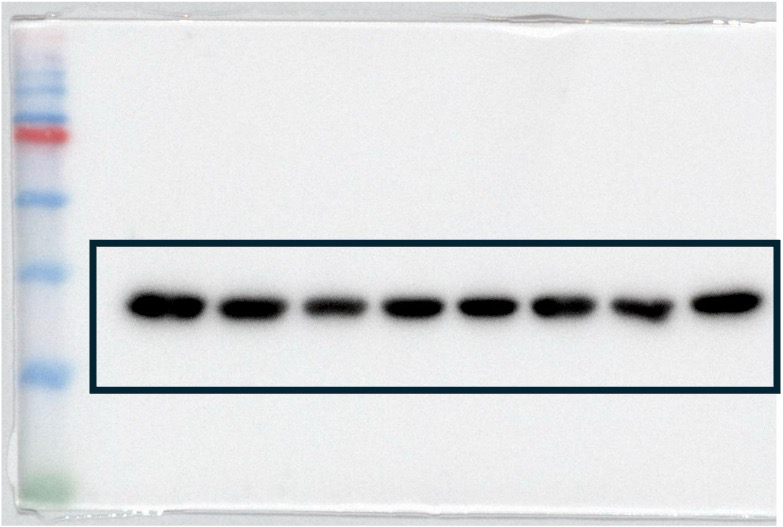

Supplement: Supplementary file 12 — Source data Fig. 7 [file 44319_2026_773_MOESM12_ESM.zip › Figure 7/Figure 7F/Western ACTIN.tif]

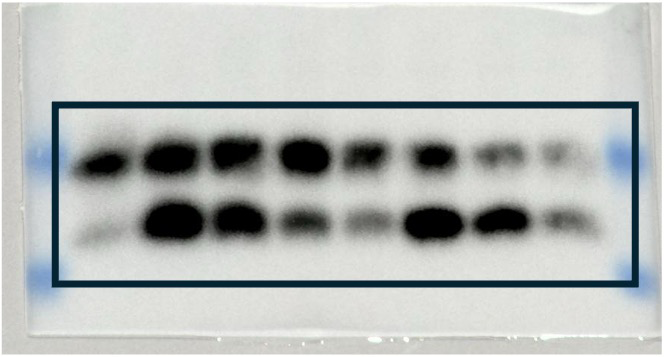

Supplement: Supplementary file 12 — Source data Fig. 7 [file 44319_2026_773_MOESM12_ESM.zip › Figure 7/Figure 7F/Western LC3B.tif]

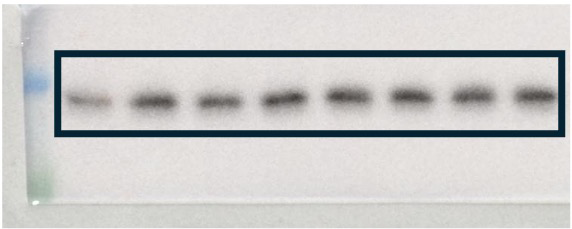

Supplement: Supplementary file 12 — Source data Fig. 7 [file 44319_2026_773_MOESM12_ESM.zip › Figure 7/Figure 7F/Western GOLPH3.tif]

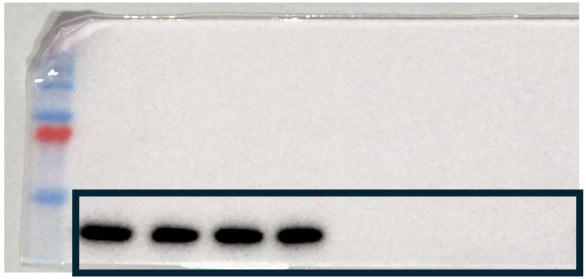

Supplement: Supplementary file 12 — Source data Fig. 7 [file 44319_2026_773_MOESM12_ESM.zip › Figure 7/Figure 7F/Western GRASP55.tif]

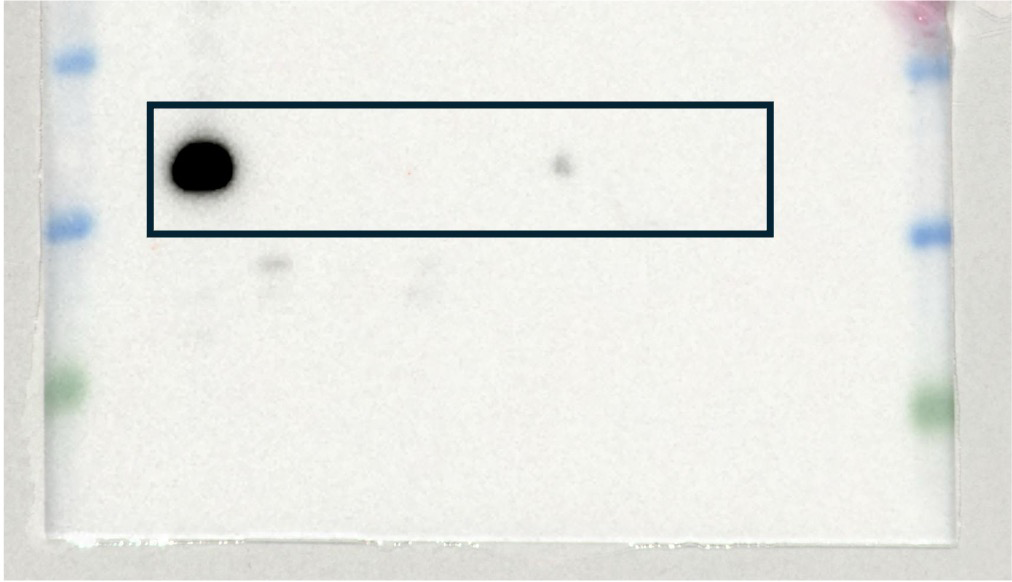

Supplement: Supplementary file 12 — Source data Fig. 7 [file 44319_2026_773_MOESM12_ESM.zip › Figure 7/Figure 7A/Western FLAG.tif]

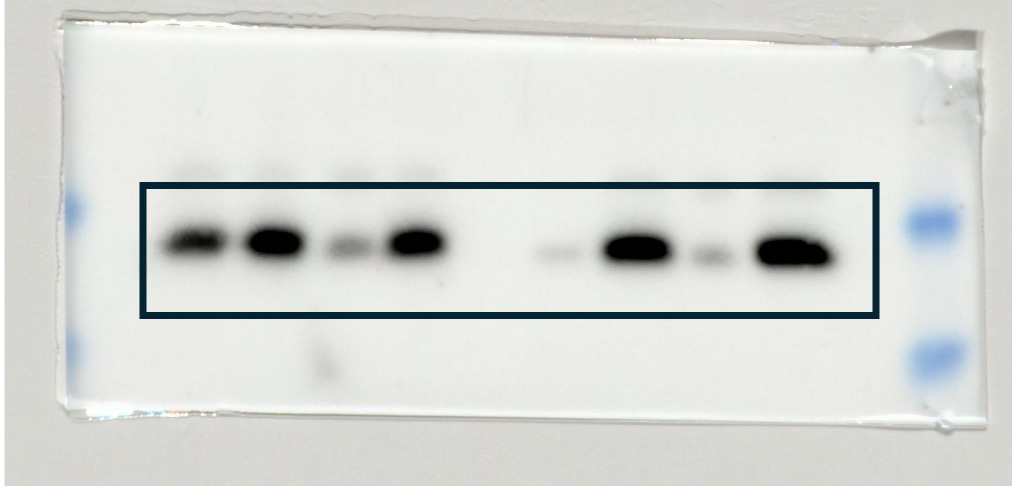

Supplement: Supplementary file 12 — Source data Fig. 7 [file 44319_2026_773_MOESM12_ESM.zip › Figure 7/Figure 7A/Western LYSET.tif]

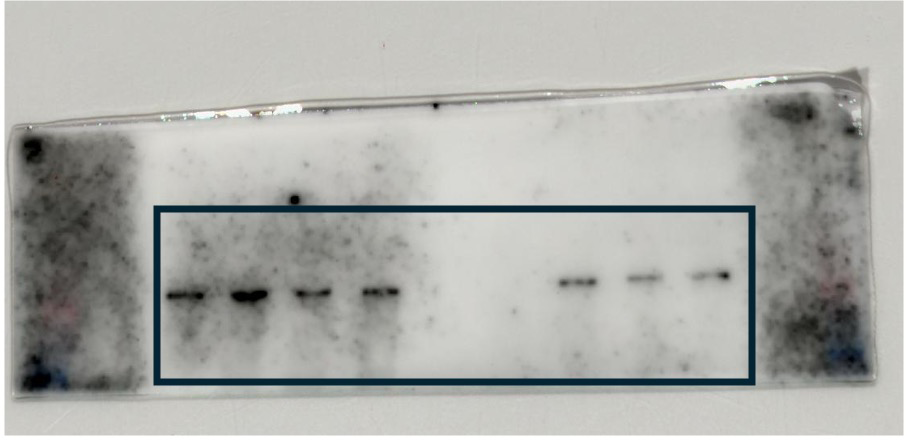

Supplement: Supplementary file 12 — Source data Fig. 7 [file 44319_2026_773_MOESM12_ESM.zip › Figure 7/Figure 7A/Western GIANTIN.tif]

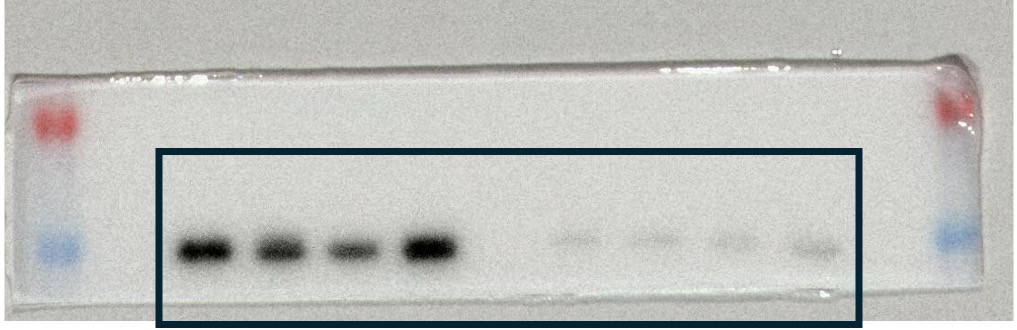

Supplement: Supplementary file 12 — Source data Fig. 7 [file 44319_2026_773_MOESM12_ESM.zip › Figure 7/Figure 7A/Western HSP60.tif]

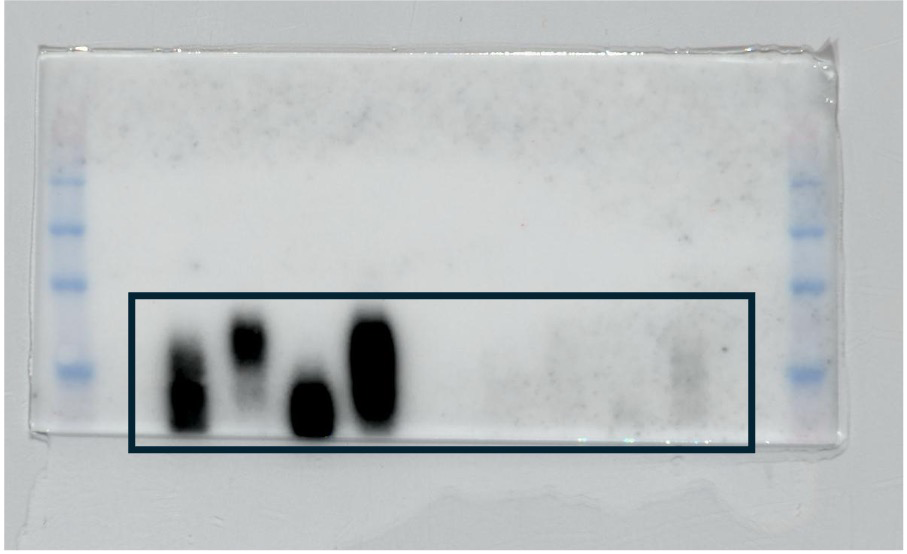

Supplement: Supplementary file 12 — Source data Fig. 7 [file 44319_2026_773_MOESM12_ESM.zip › Figure 7/Figure 7A/Western LAMP2.tif]

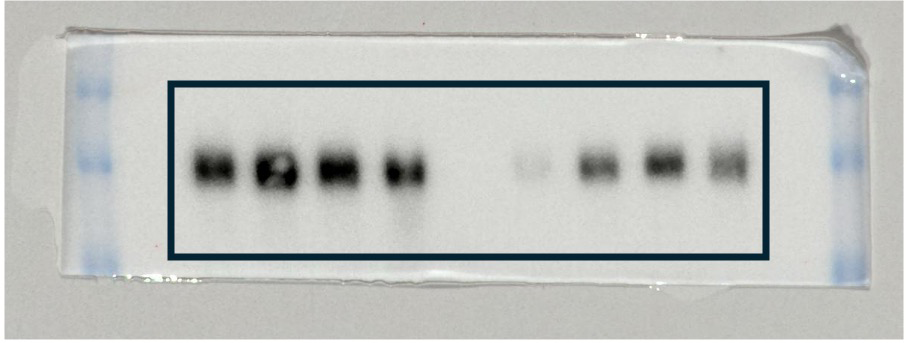

Supplement: Supplementary file 12 — Source data Fig. 7 [file 44319_2026_773_MOESM12_ESM.zip › Figure 7/Figure 7A/Western GM130.tif]

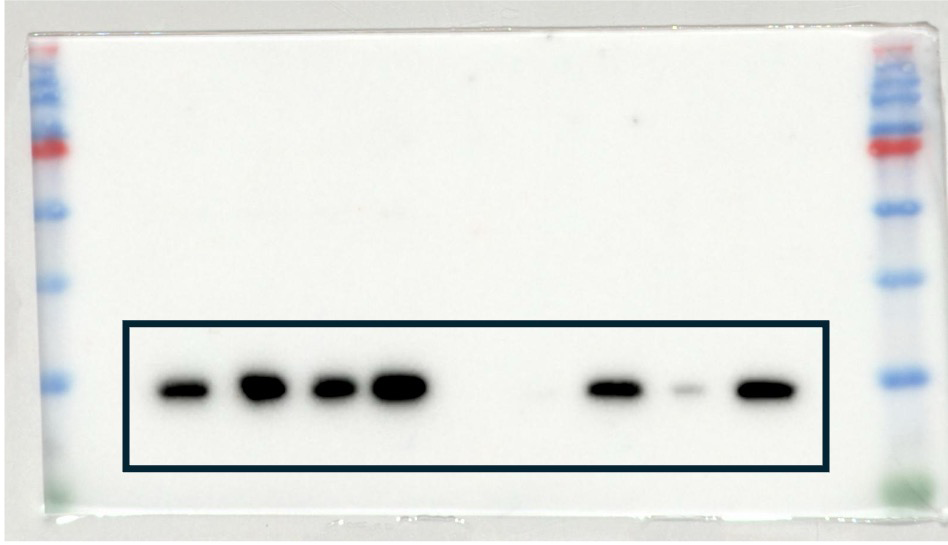

Supplement: Supplementary file 12 — Source data Fig. 7 [file 44319_2026_773_MOESM12_ESM.zip › Figure 7/Figure 7A/Western GOLPH3.tif]

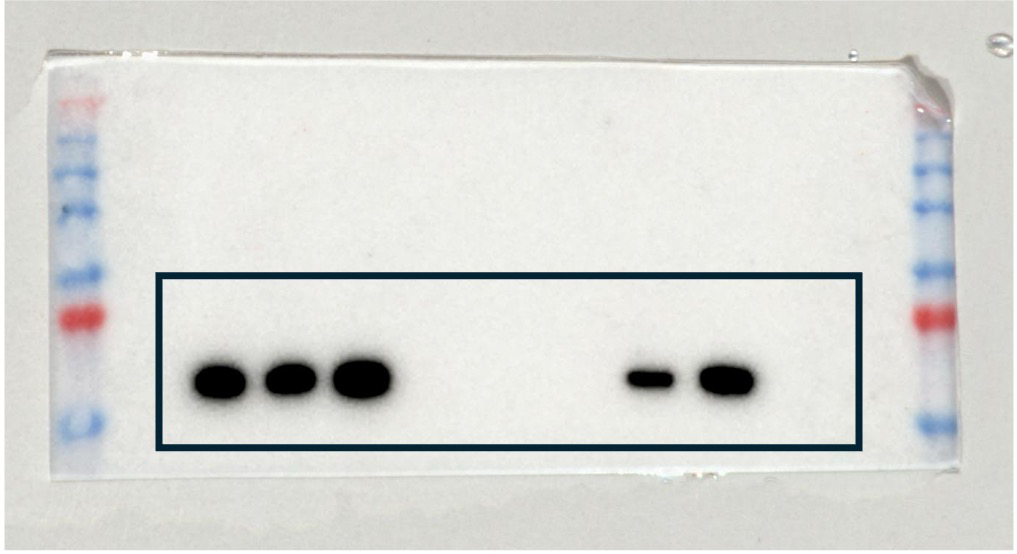

Supplement: Supplementary file 12 — Source data Fig. 7 [file 44319_2026_773_MOESM12_ESM.zip › Figure 7/Figure 7A/Western GRASP65.tif]

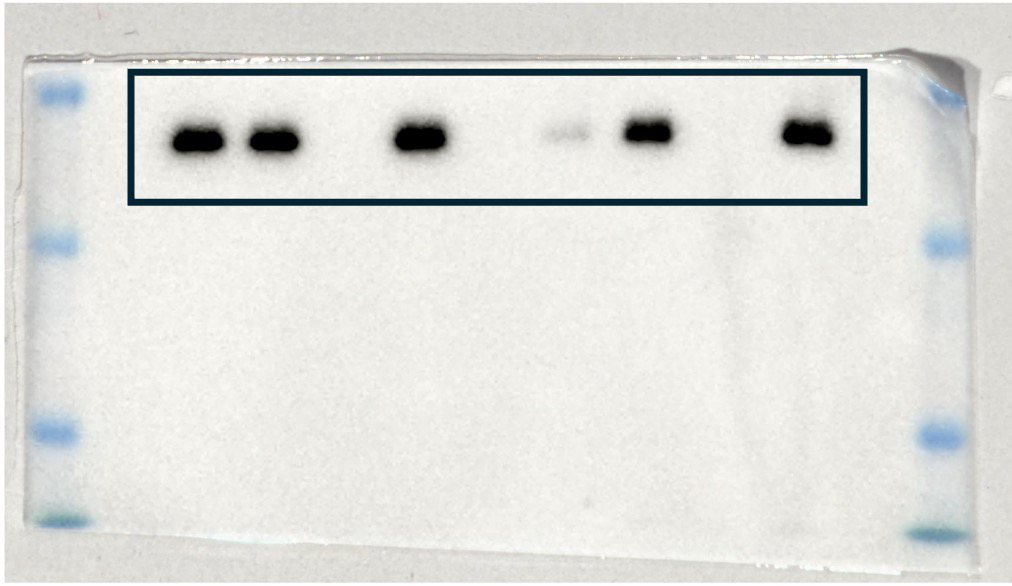

Supplement: Supplementary file 12 — Source data Fig. 7 [file 44319_2026_773_MOESM12_ESM.zip › Figure 7/Figure 7A/Western GRASP55.tif]

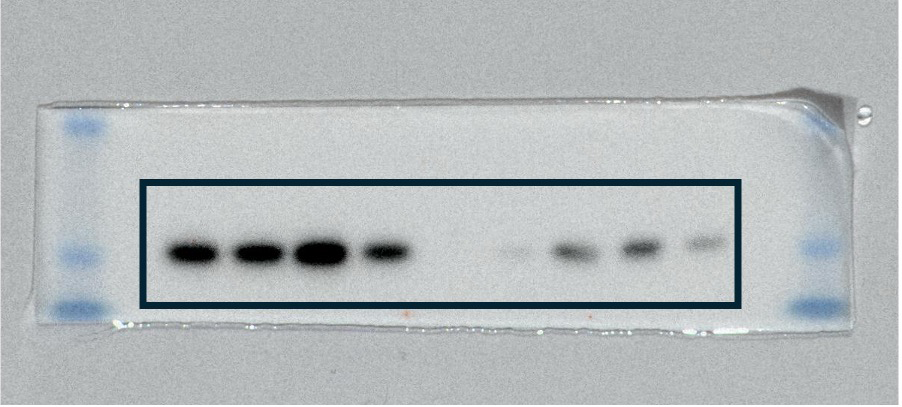

Supplement: Supplementary file 12 — Source data Fig. 7 [file 44319_2026_773_MOESM12_ESM.zip › Figure 7/Figure 7A/Western HSP47.tif]

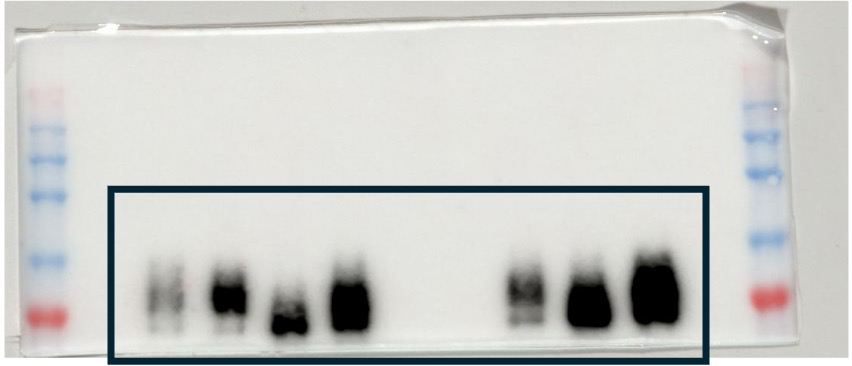

Supplement: Supplementary file 12 — Source data Fig. 7 [file 44319_2026_773_MOESM12_ESM.zip › Figure 7/Figure 7A/Western TGN46.tif]

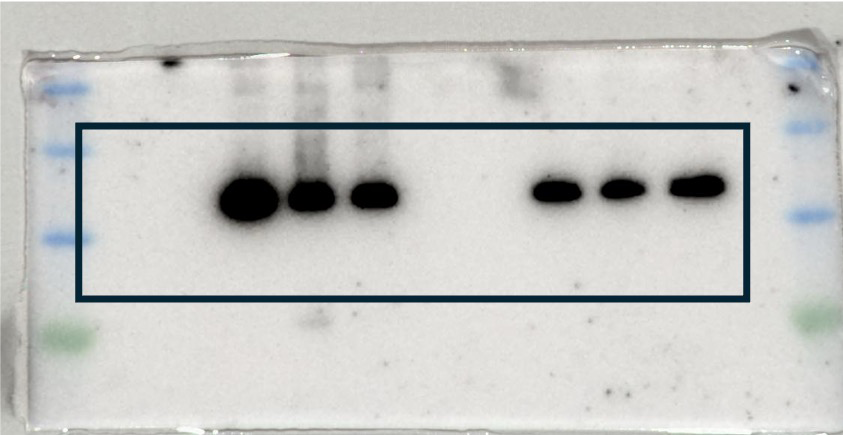

Supplement: Supplementary file 12 — Source data Fig. 7 [file 44319_2026_773_MOESM12_ESM.zip › Figure 7/Figure 7A/Western HA.tif]

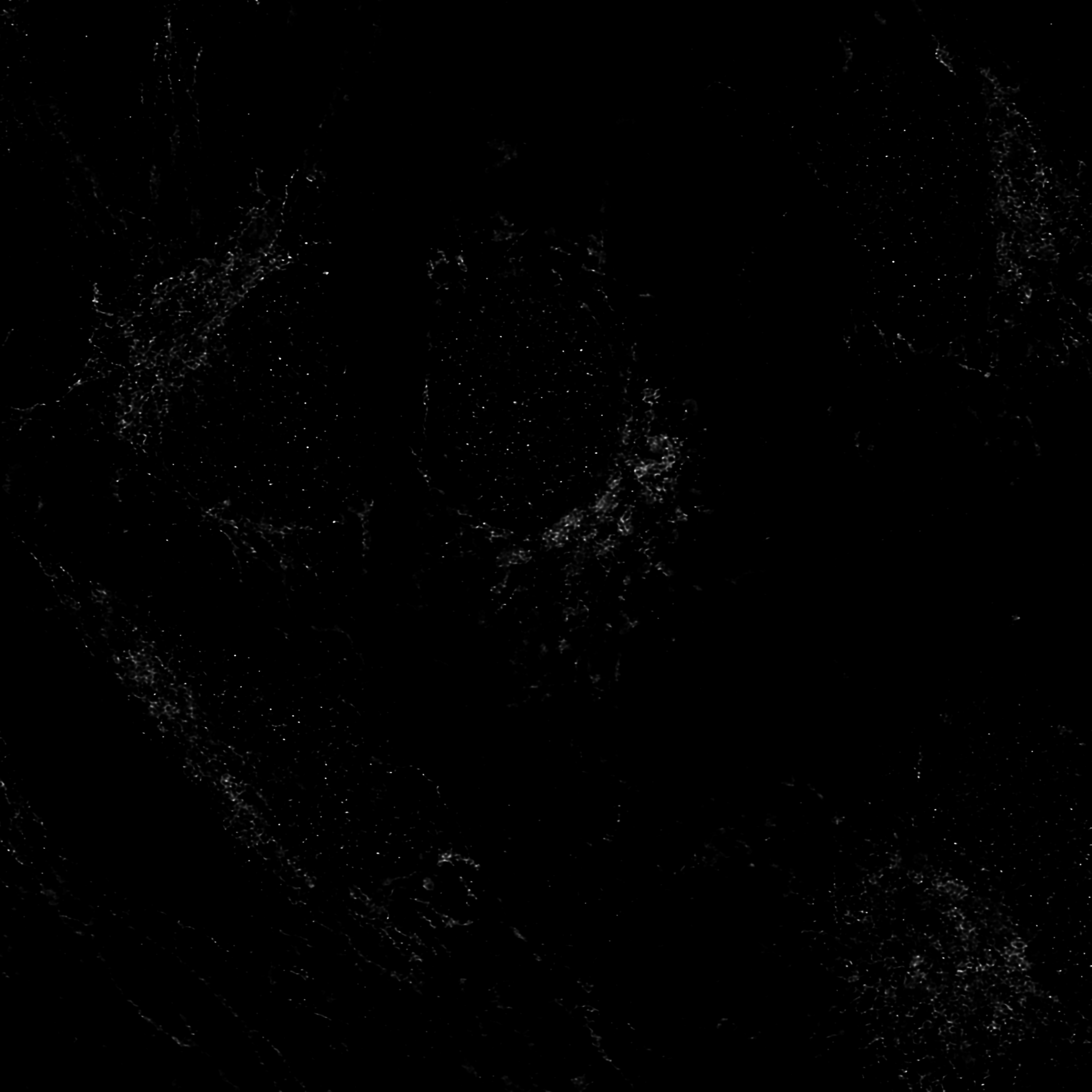

Supplement: Supplementary file 12 — Source data Fig. 7 [file 44319_2026_773_MOESM12_ESM.zip › Figure 7/Figure 7H/IF GRASP55KO GOLPH3 .tif]

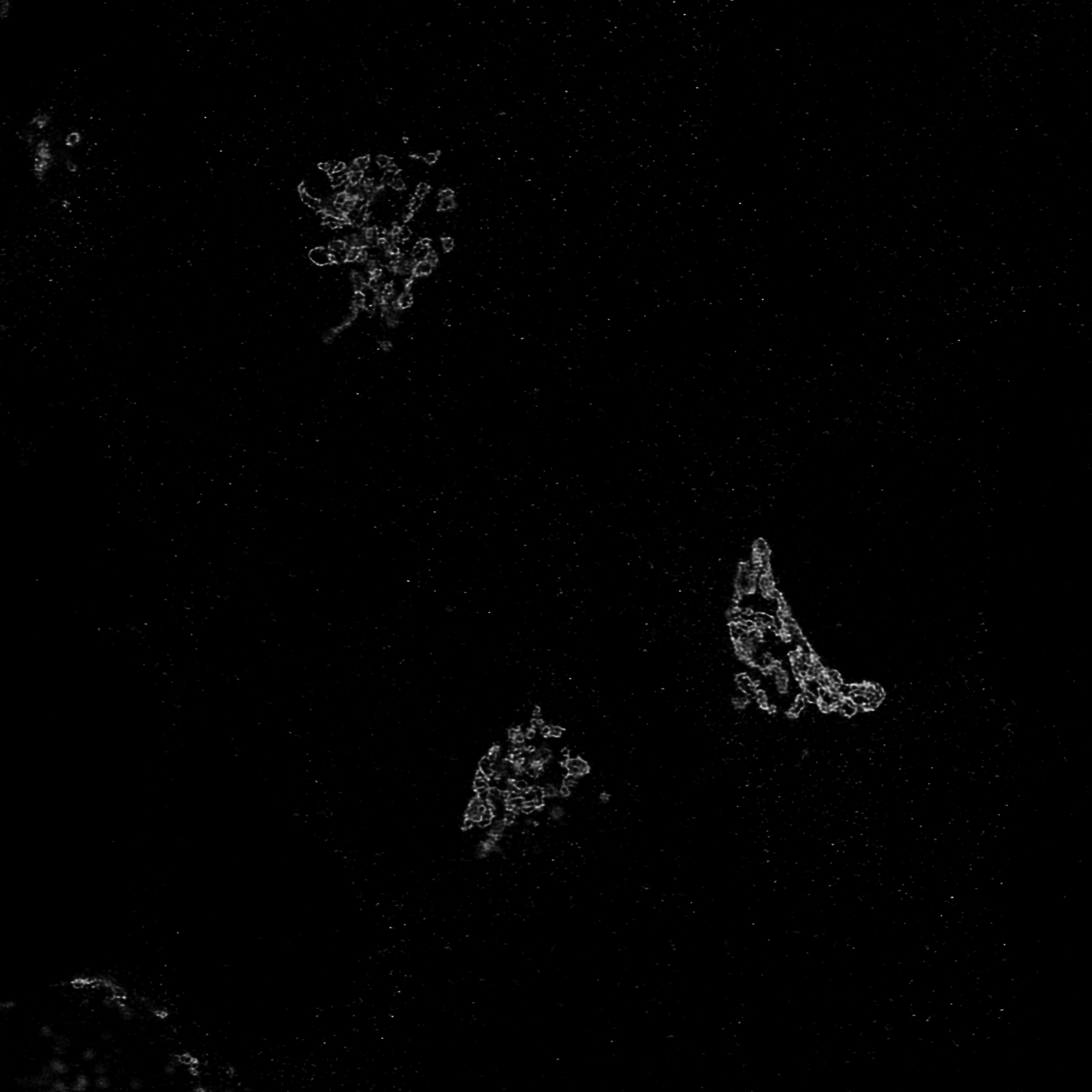

Supplement: Supplementary file 12 — Source data Fig. 7 [file 44319_2026_773_MOESM12_ESM.zip › Figure 7/Figure 7H/IF WT GIANTIN.tif]

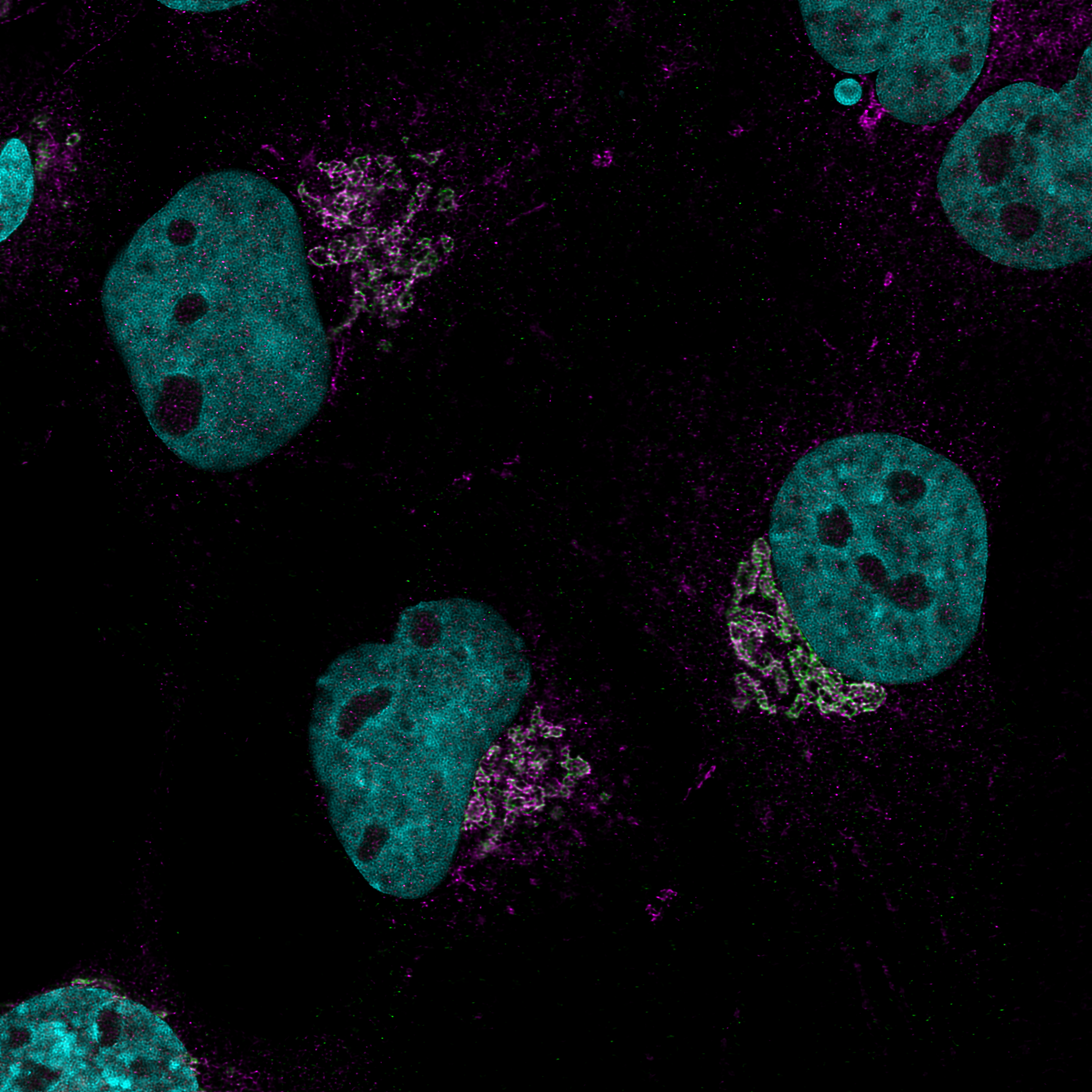

Supplement: Supplementary file 12 — Source data Fig. 7 [file 44319_2026_773_MOESM12_ESM.zip › Figure 7/Figure 7H/IF WT GOLPH3_GIANTIN MERGE.tif]
