# Supplementary figures and images for: GRASP55 maintains lysosome function by controlling sorting of lysosomal enzymes at the Golgi (part 4 of 5)
Source: EMBO Rep. 2026 Apr 16;27(11):2947–72. doi: 10.1038/s44319-026-00773-w (PMC13261057; doi:10.1038/s44319-026-00773-w)

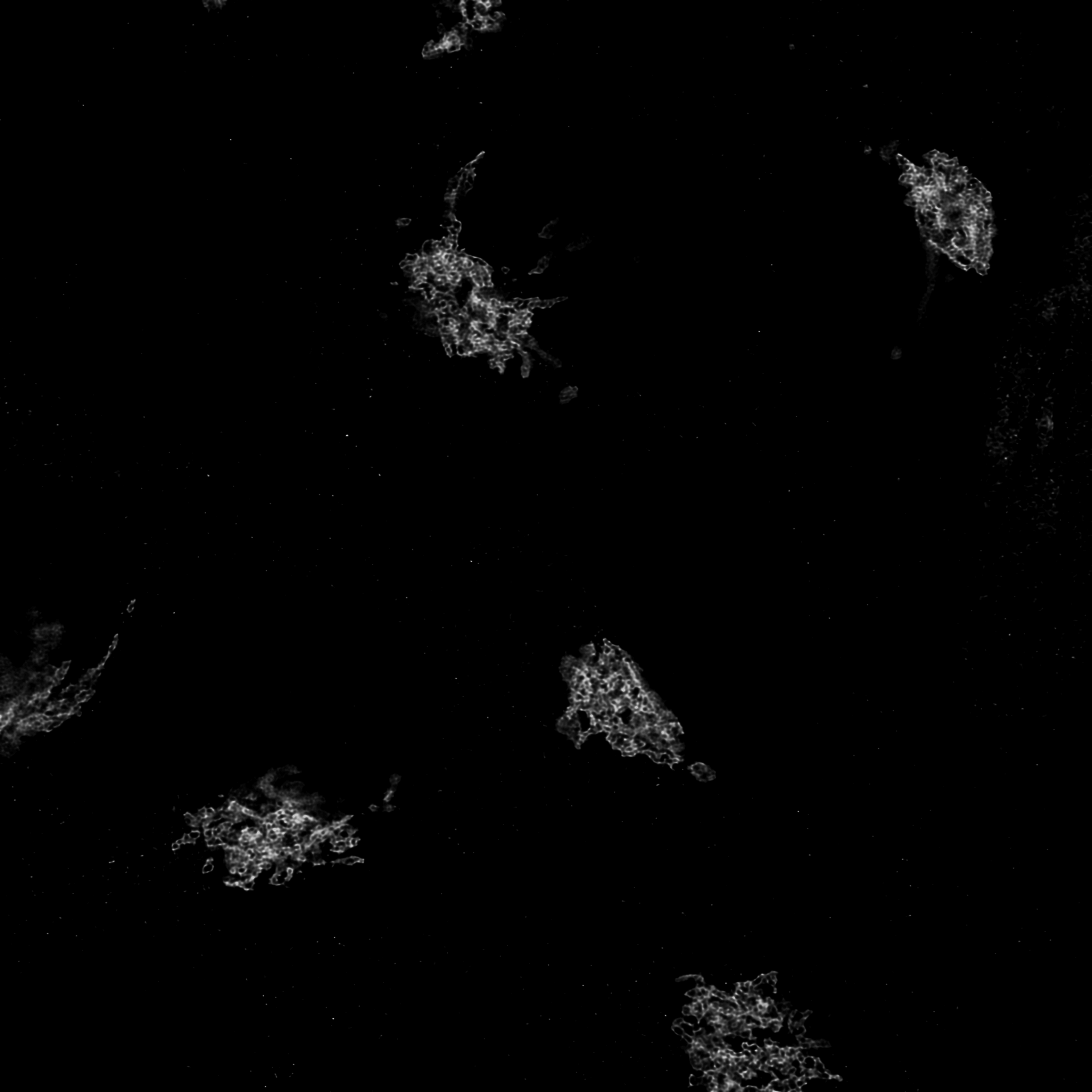

Supplement: Supplementary file 12 — Source data Fig. 7 [file 44319_2026_773_MOESM12_ESM.zip › Figure 7/Figure 7H/IF GRASP55KO+WT GIANTIN .tif]

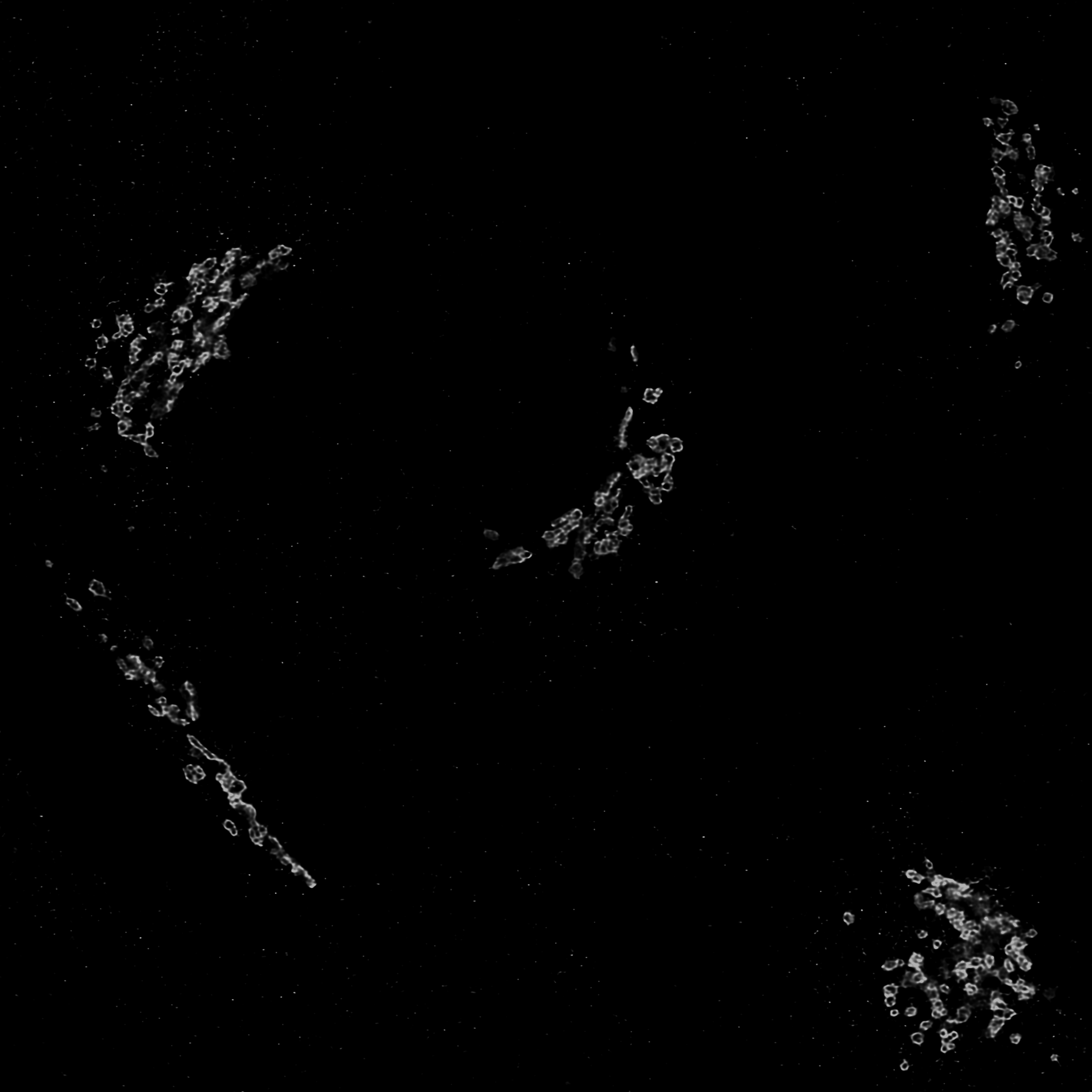

Supplement: Supplementary file 12 — Source data Fig. 7 [file 44319_2026_773_MOESM12_ESM.zip › Figure 7/Figure 7H/IF GRASP55KO GIANTIN.tif]

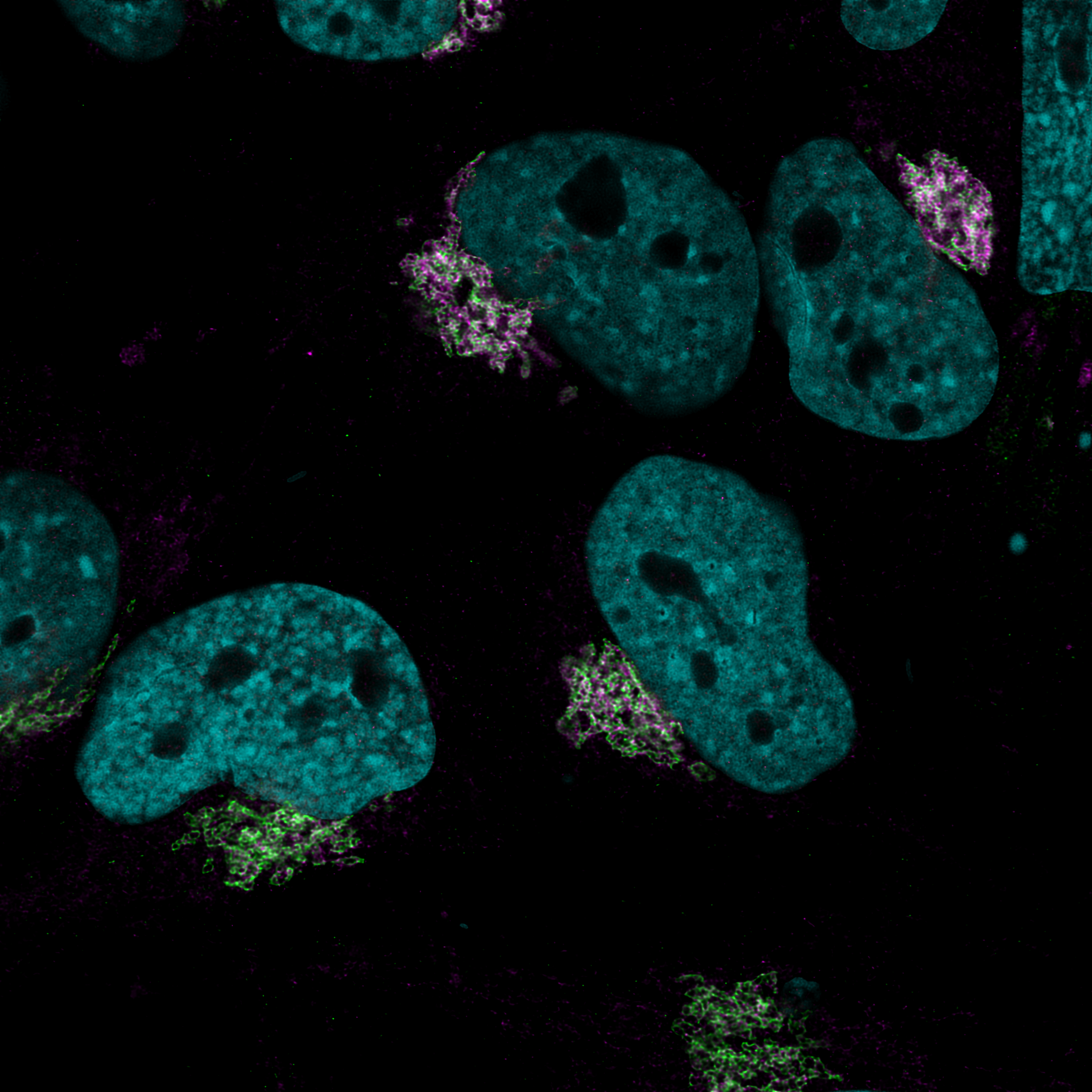

Supplement: Supplementary file 12 — Source data Fig. 7 [file 44319_2026_773_MOESM12_ESM.zip › Figure 7/Figure 7H/IF GRASP55KO+WT GOLPH3_GIANTIN MERGE.tif]

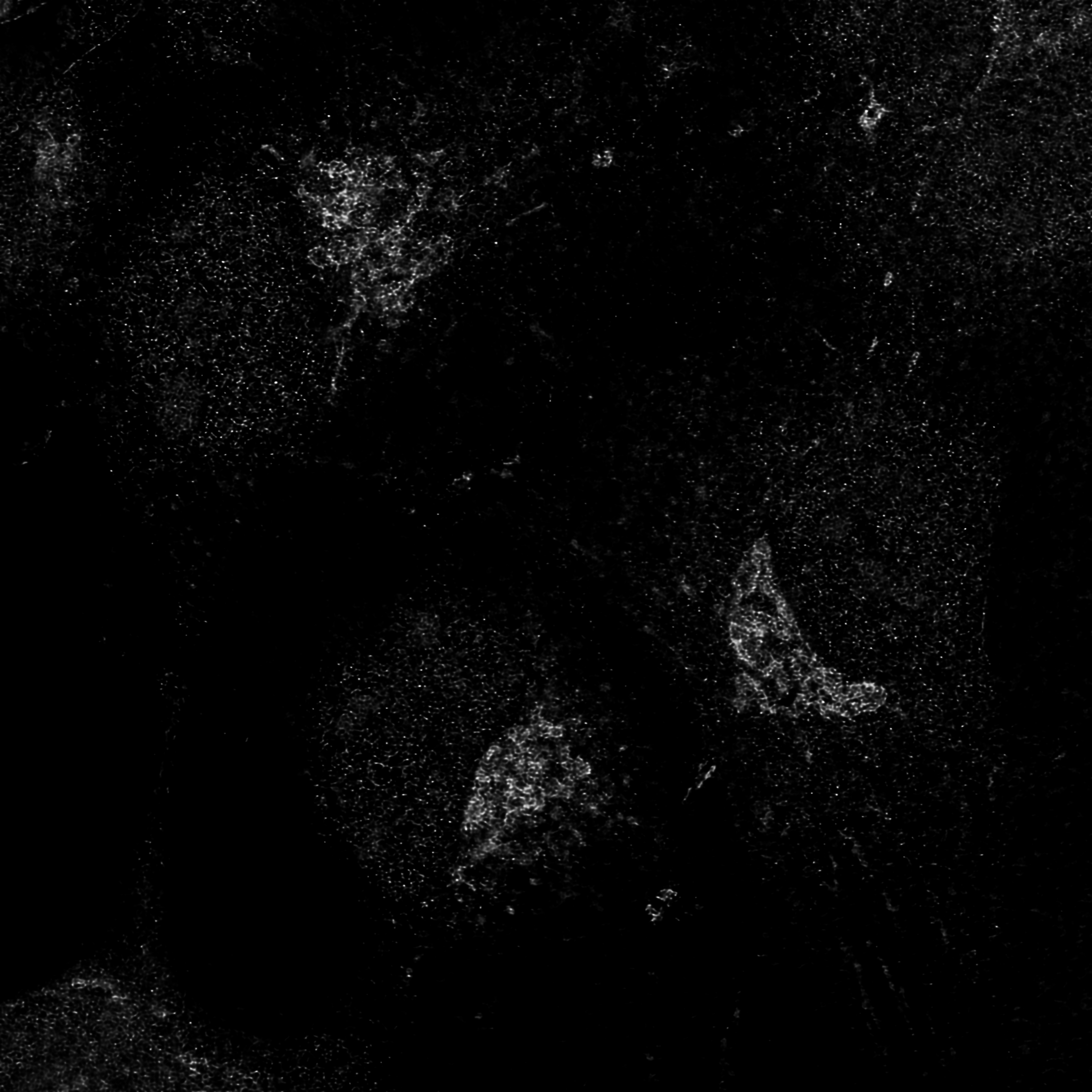

Supplement: Supplementary file 12 — Source data Fig. 7 [file 44319_2026_773_MOESM12_ESM.zip › Figure 7/Figure 7H/IF WT GOLPH3.tif]

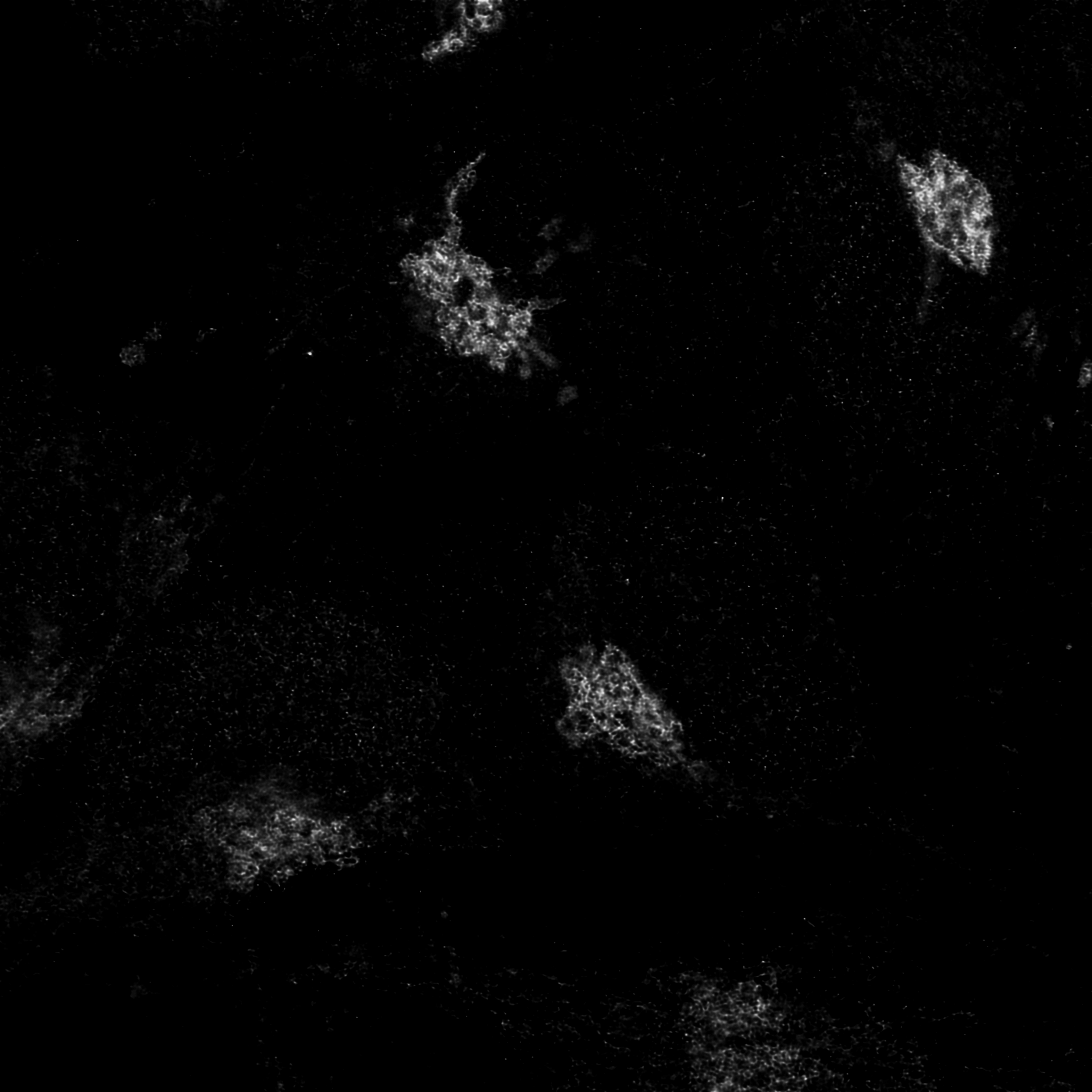

Supplement: Supplementary file 12 — Source data Fig. 7 [file 44319_2026_773_MOESM12_ESM.zip › Figure 7/Figure 7H/IF GRASP55KO+WT GOLPH3.tif]

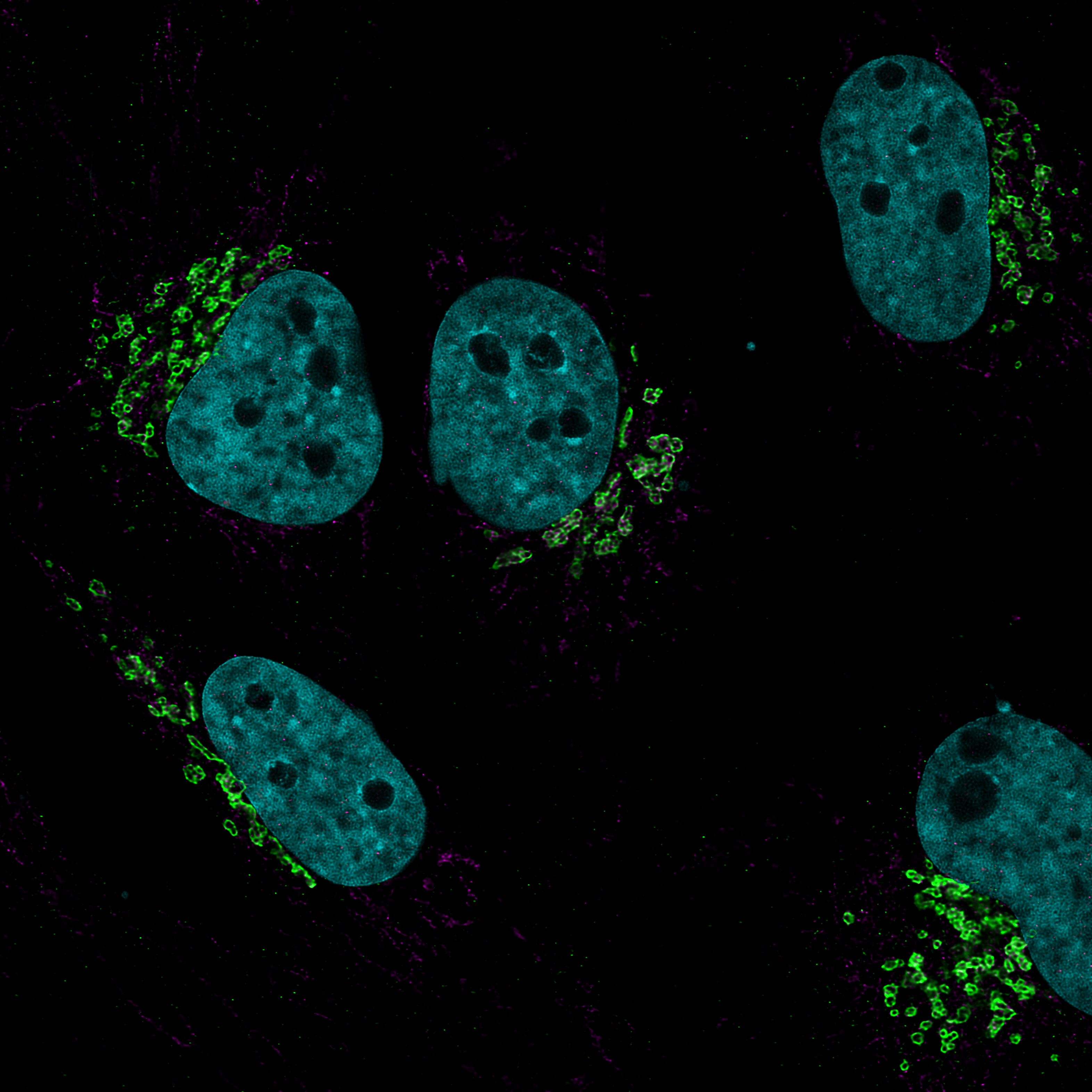

Supplement: Supplementary file 12 — Source data Fig. 7 [file 44319_2026_773_MOESM12_ESM.zip › Figure 7/Figure 7H/IF GRASP55KO GOLPH3_GIANTIN MERGE.tif]

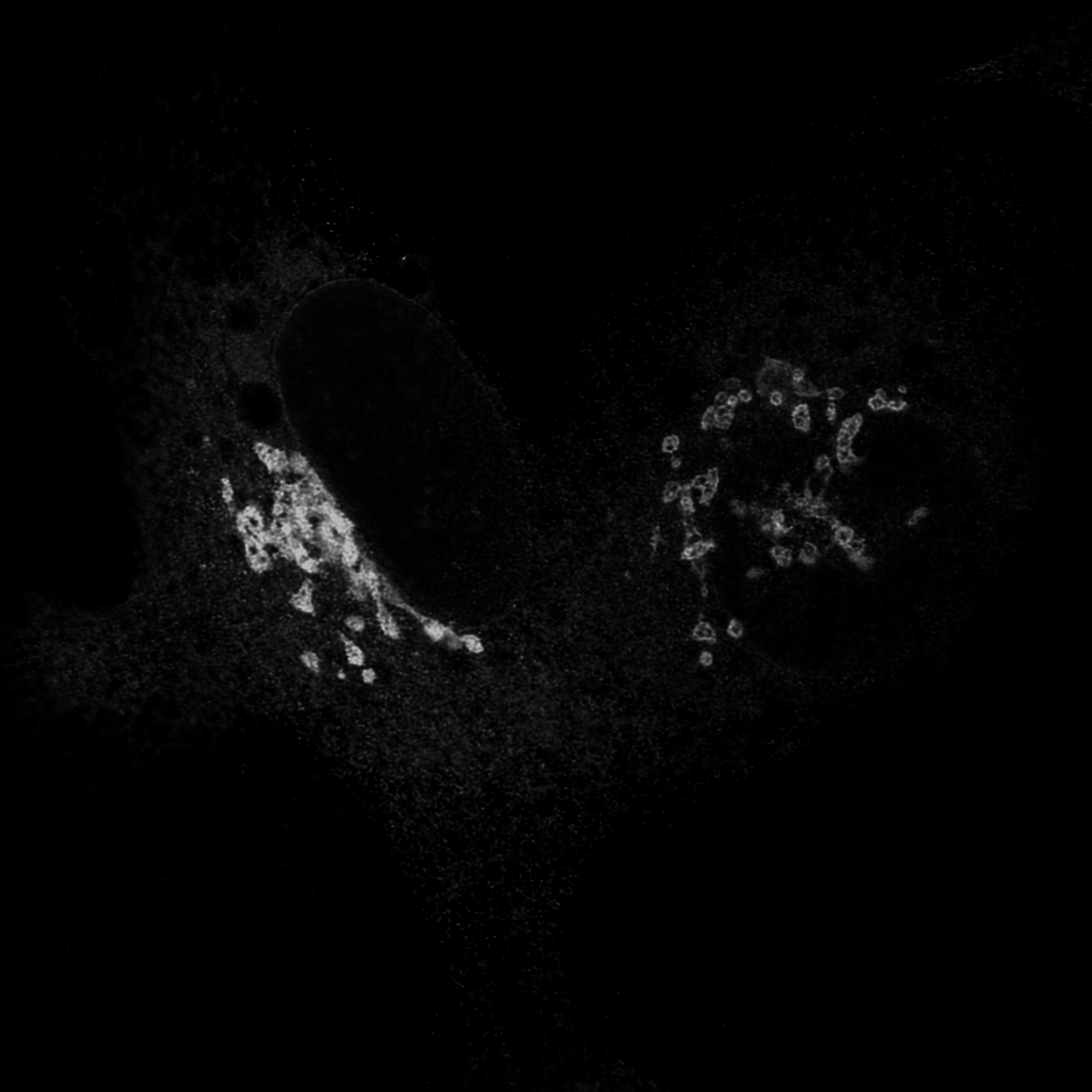

Supplement: Supplementary file 13 — Source data Fig. 8 [file 44319_2026_773_MOESM13_ESM.zip › Figure 8/Figure 8A/IF WT GNPTAB WT Myc.tif]

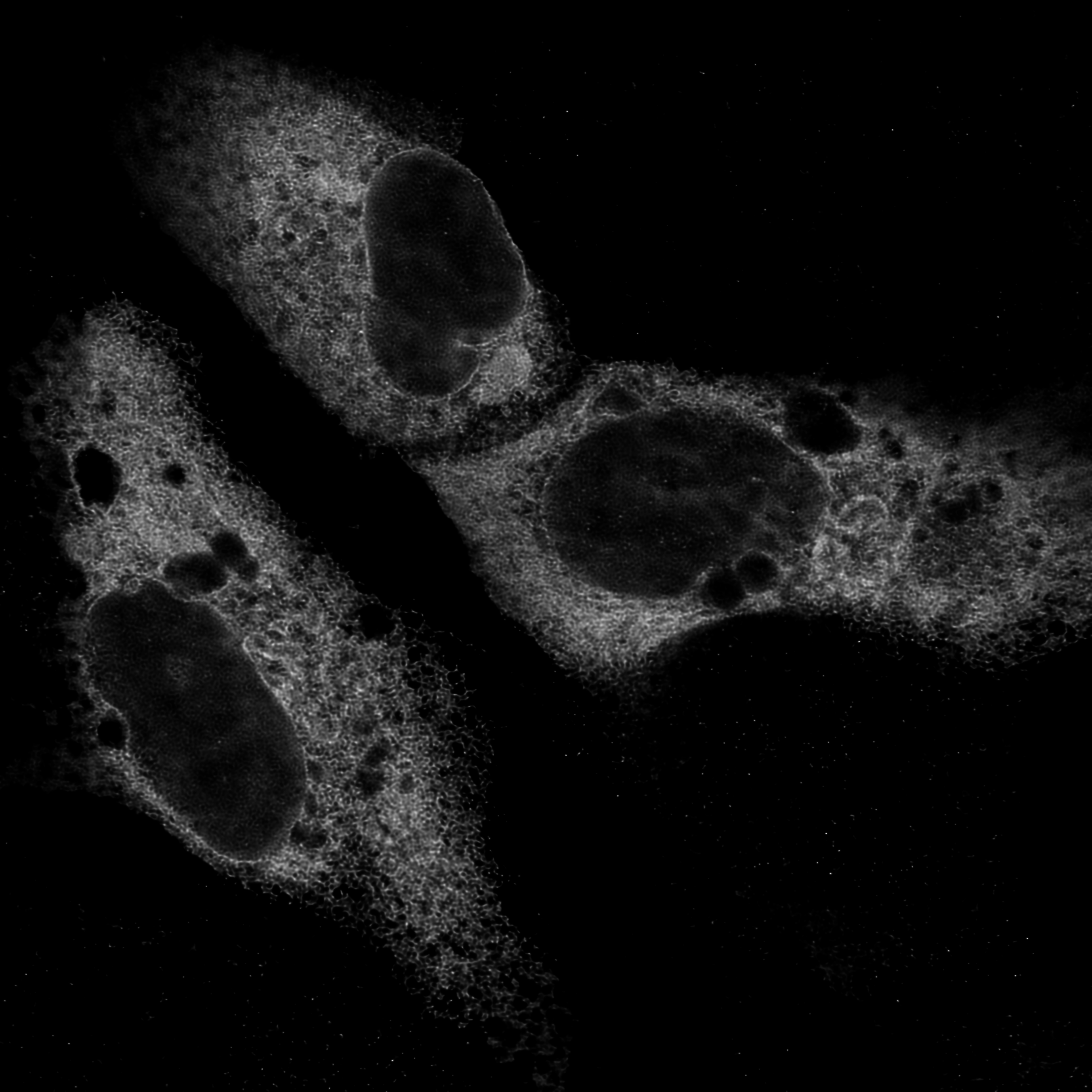

Supplement: Supplementary file 13 — Source data Fig. 8 [file 44319_2026_773_MOESM13_ESM.zip › Figure 8/Figure 8A/IF GRASP55KO GNPTAB WT Myc.tif]

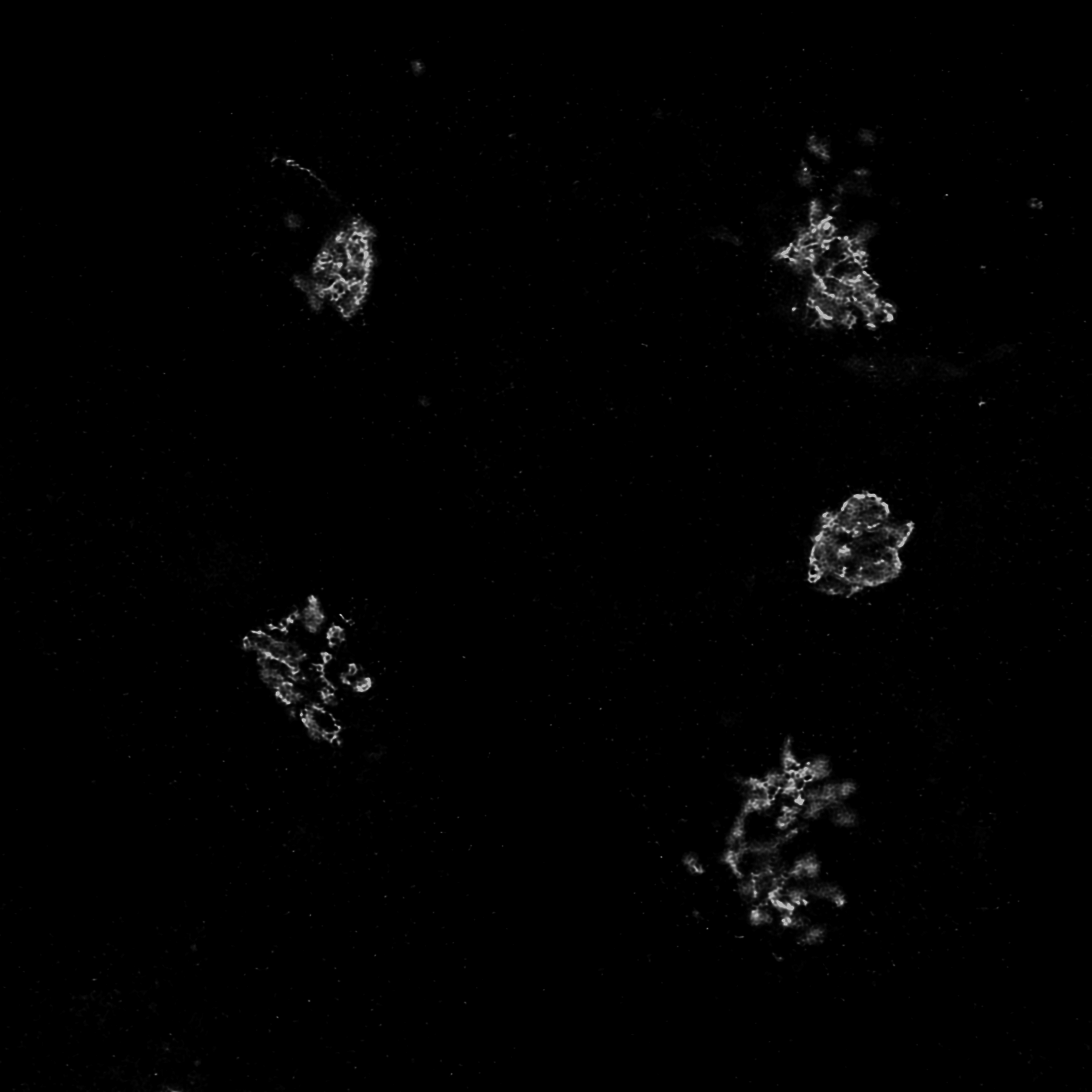

Supplement: Supplementary file 13 — Source data Fig. 8 [file 44319_2026_773_MOESM13_ESM.zip › Figure 8/Figure 8A/IF GRASP55KO GNPTAB WT GM130.tif]

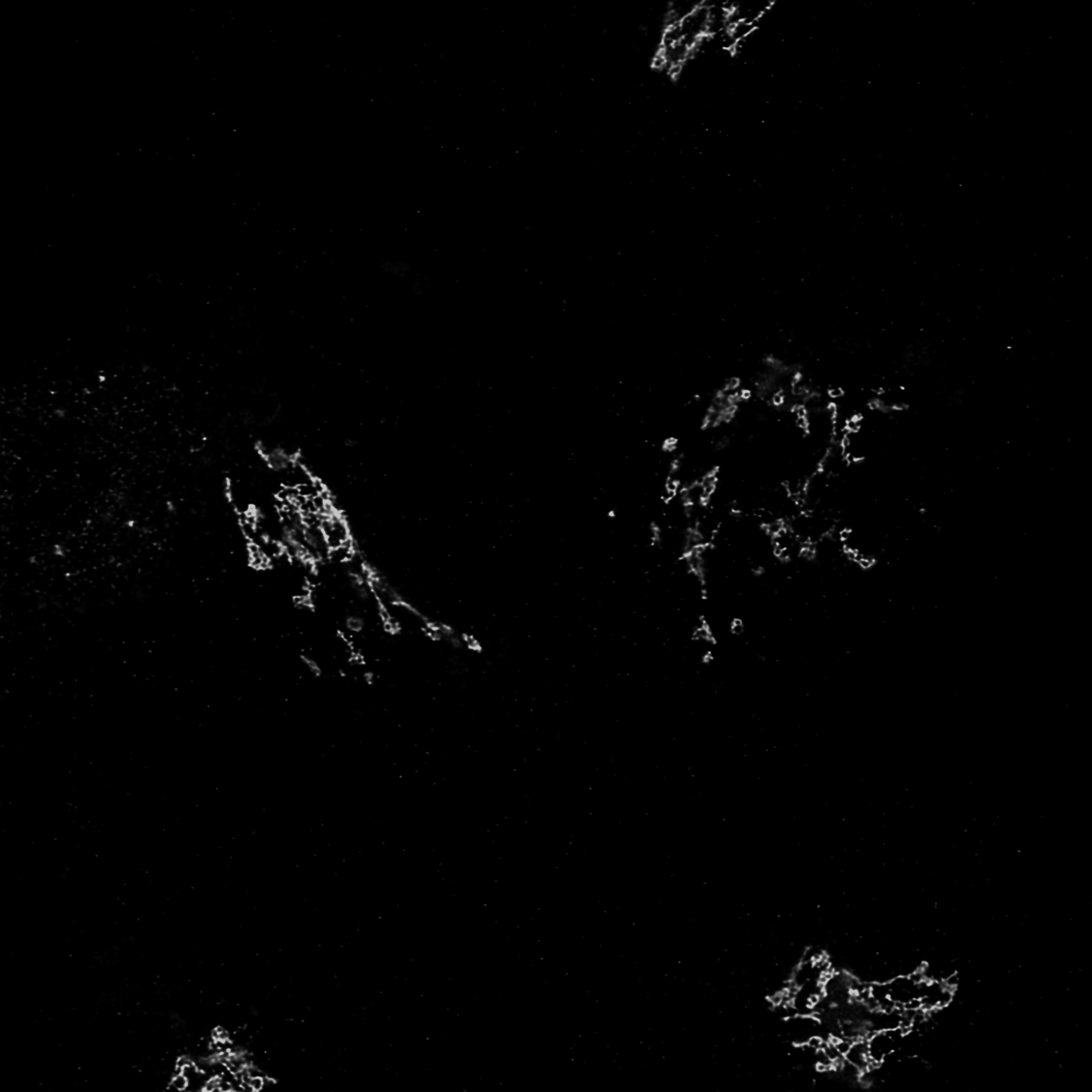

Supplement: Supplementary file 13 — Source data Fig. 8 [file 44319_2026_773_MOESM13_ESM.zip › Figure 8/Figure 8A/IF WT GNPTAB WT GM130.tif]

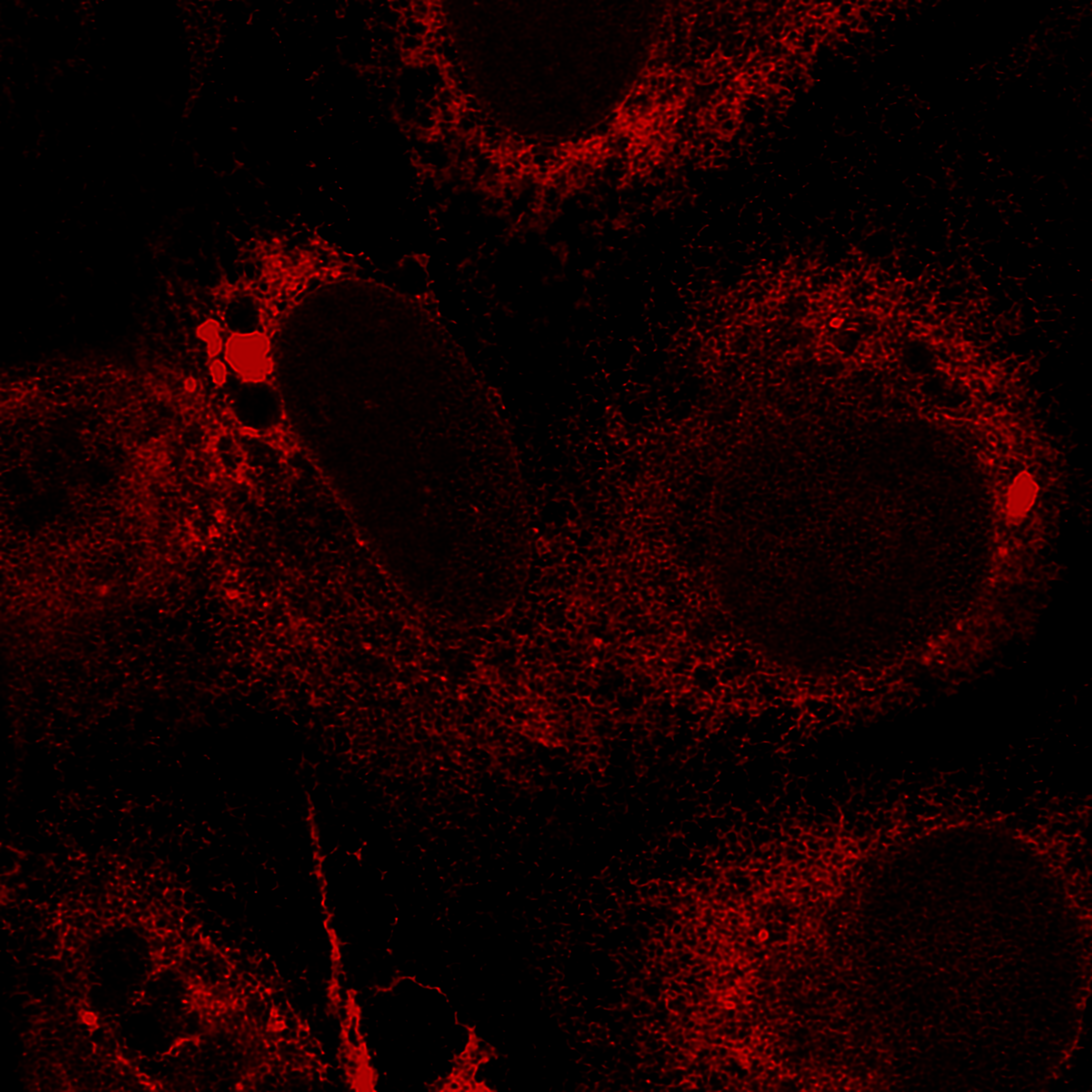

Supplement: Supplementary file 13 — Source data Fig. 8 [file 44319_2026_773_MOESM13_ESM.zip › Figure 8/Figure 8A/IF WT GNPTAB WT HSP47 MERGE.tif]

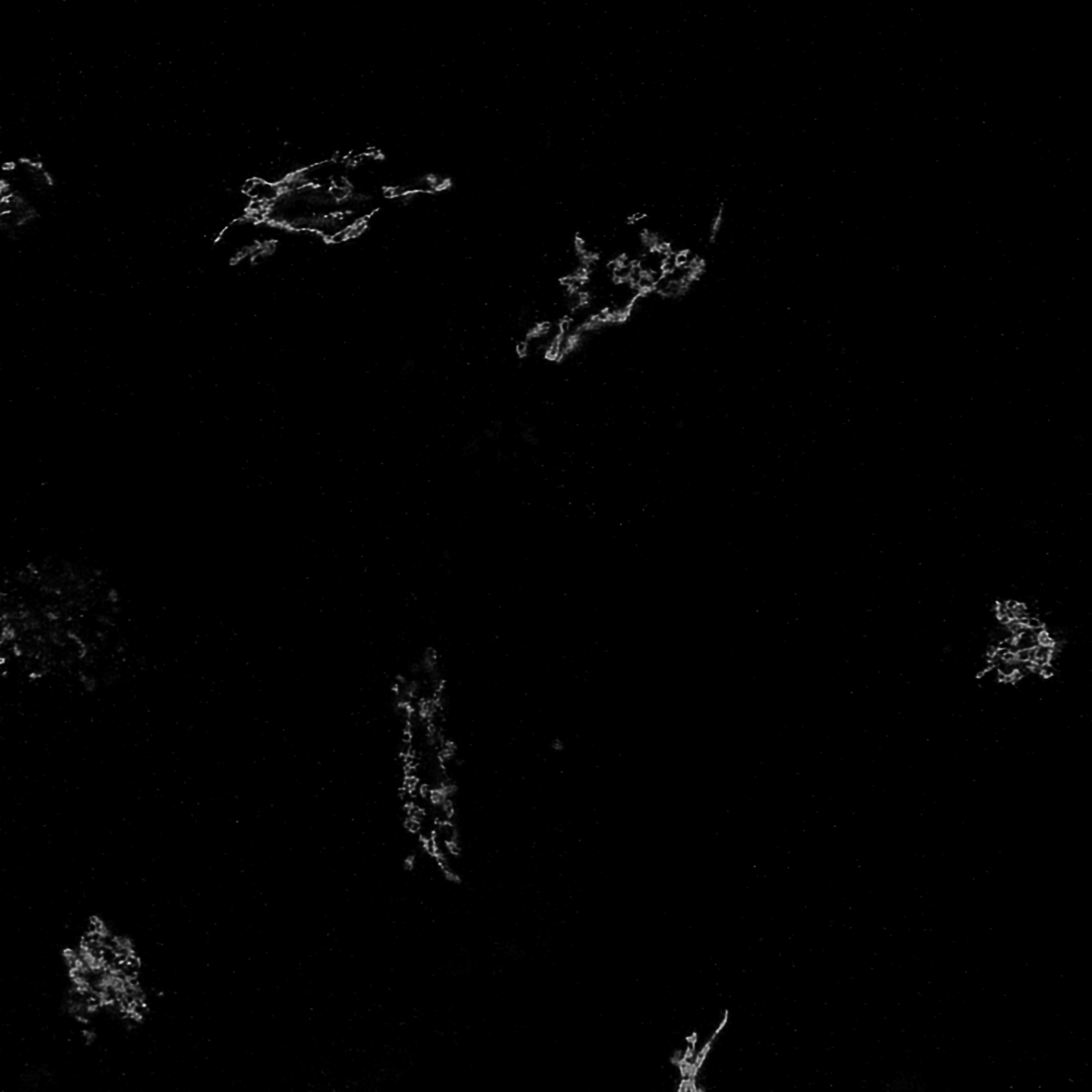

Supplement: Supplementary file 13 — Source data Fig. 8 [file 44319_2026_773_MOESM13_ESM.zip › Figure 8/Figure 8A/IF WT GNPTAB QELL GM130.tif]

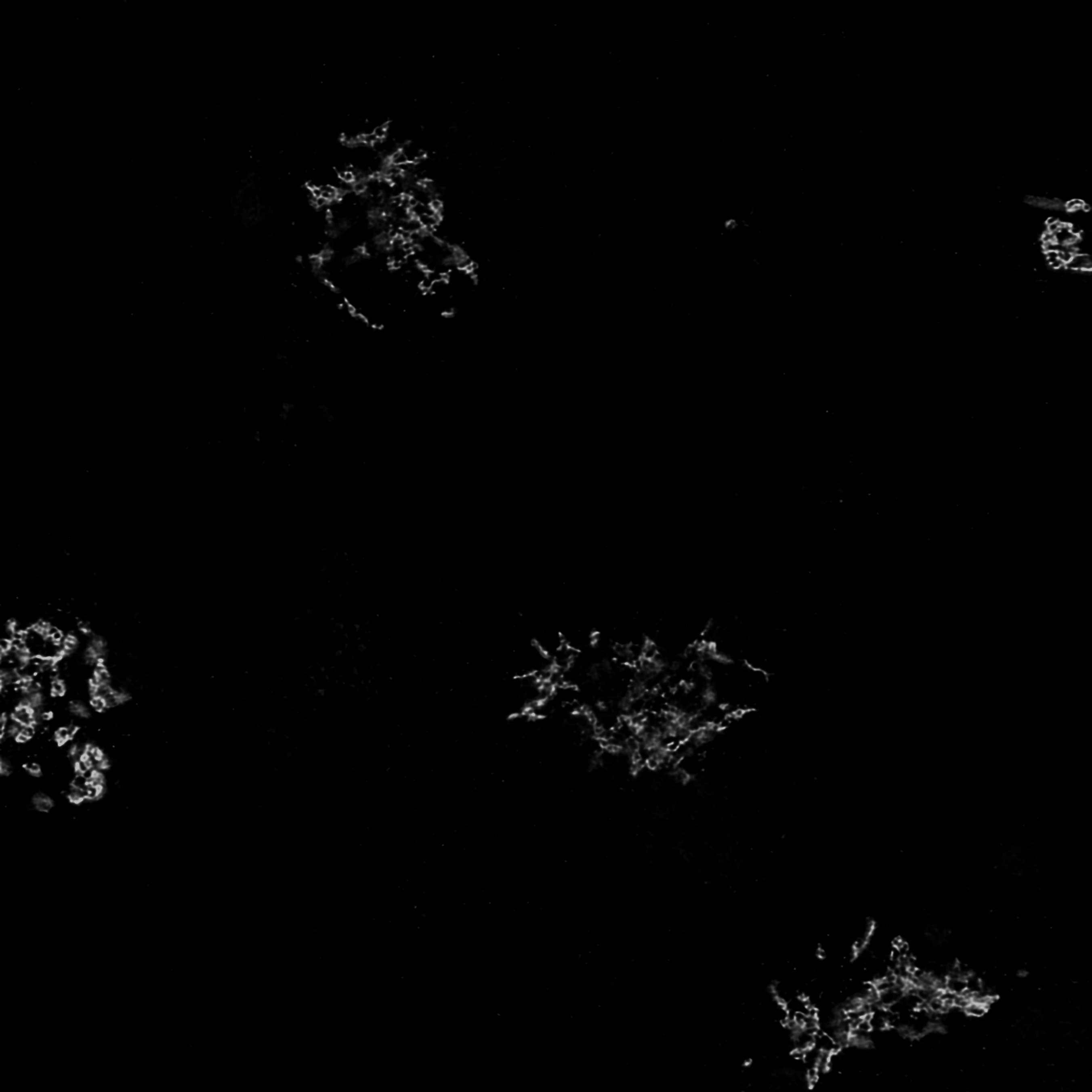

Supplement: Supplementary file 13 — Source data Fig. 8 [file 44319_2026_773_MOESM13_ESM.zip › Figure 8/Figure 8A/IF GRASP55KO GNPTAB QELL GM130.tif]

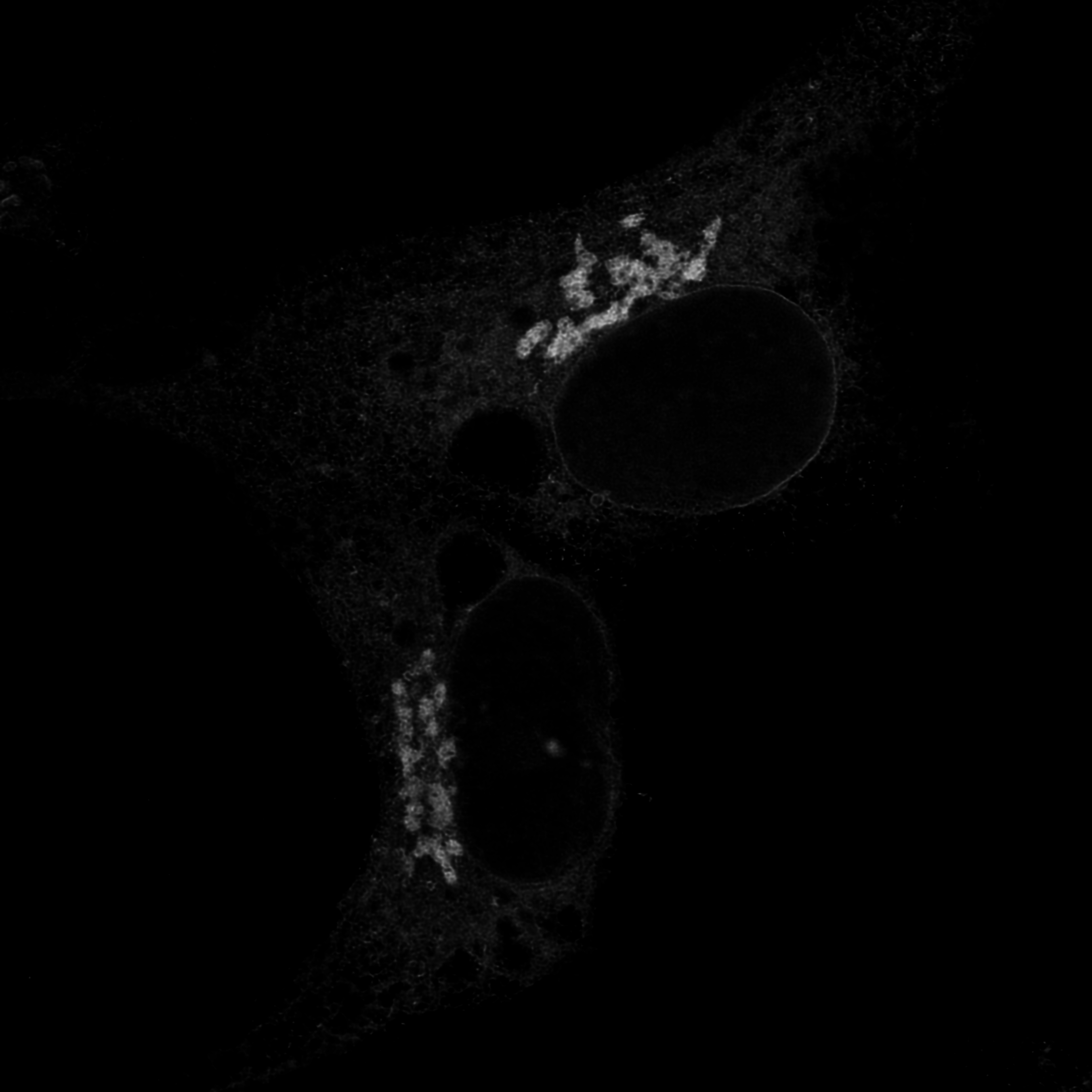

Supplement: Supplementary file 13 — Source data Fig. 8 [file 44319_2026_773_MOESM13_ESM.zip › Figure 8/Figure 8A/IF WT GNPTAB QELL Myc.tif]

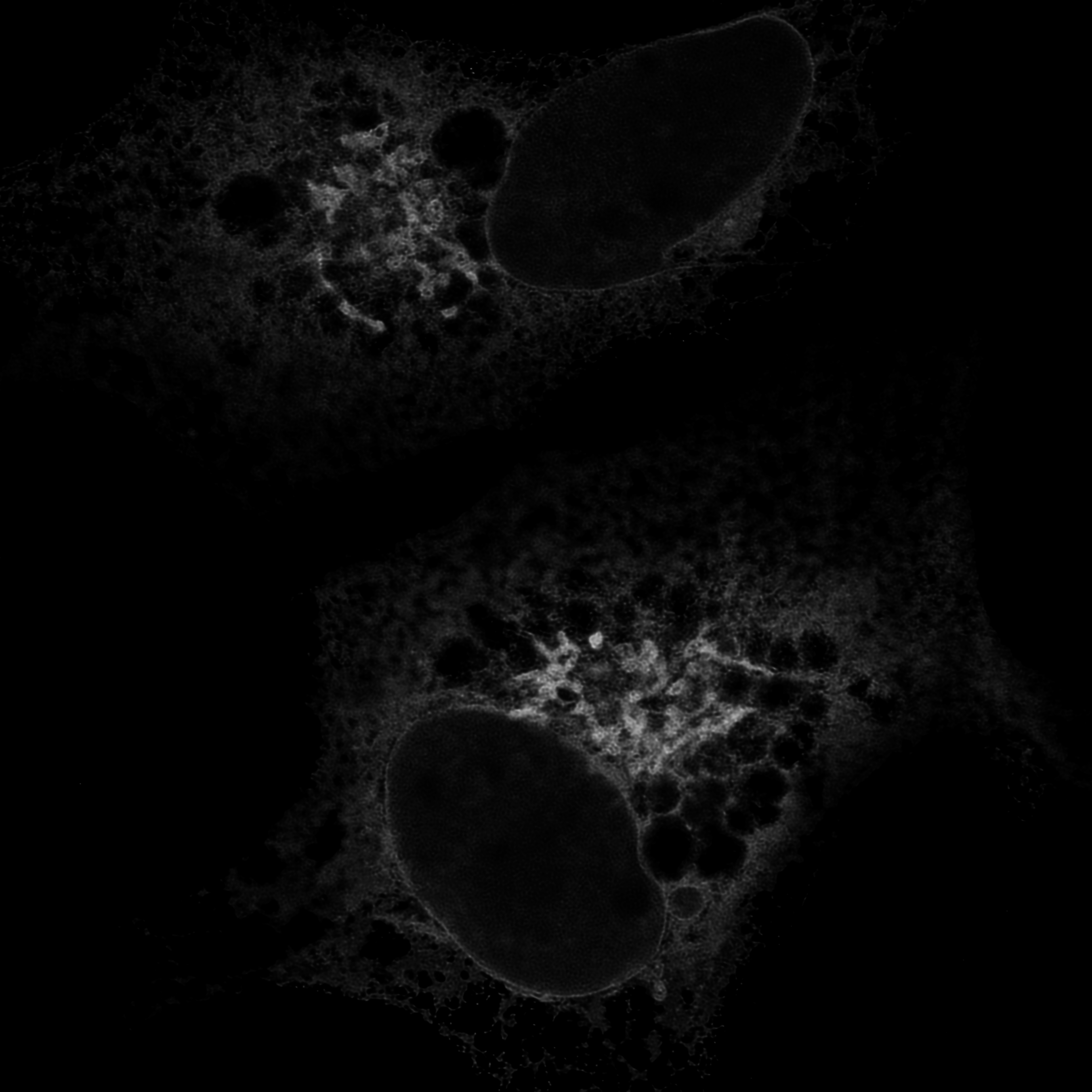

Supplement: Supplementary file 13 — Source data Fig. 8 [file 44319_2026_773_MOESM13_ESM.zip › Figure 8/Figure 8A/IF GRASP55KO GNPTAB QELL Myc.tif]

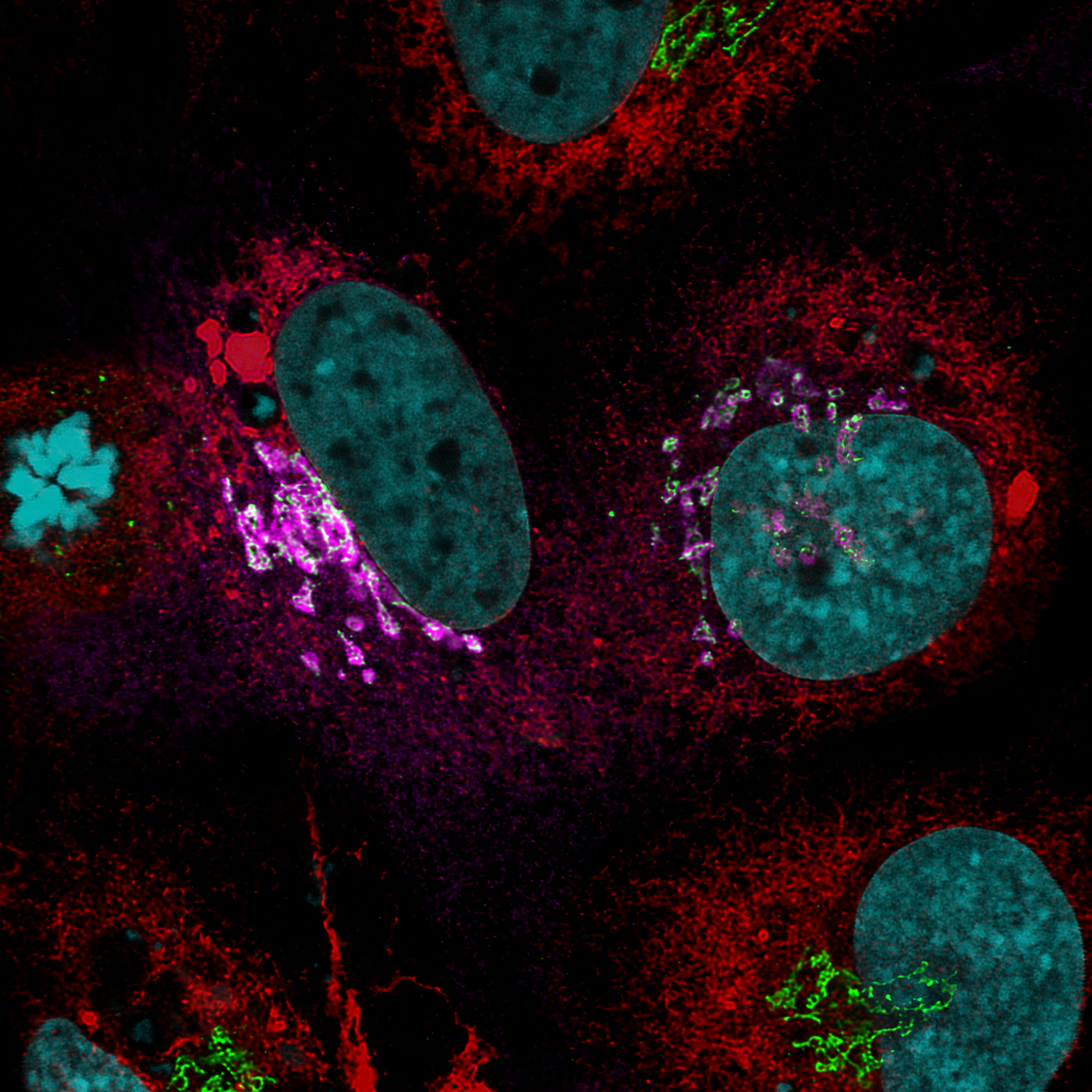

Supplement: Supplementary file 13 — Source data Fig. 8 [file 44319_2026_773_MOESM13_ESM.zip › Figure 8/Figure 8A/IF WT GNPTAB WT HSP47_Myc_GM130 MERGE.tif]

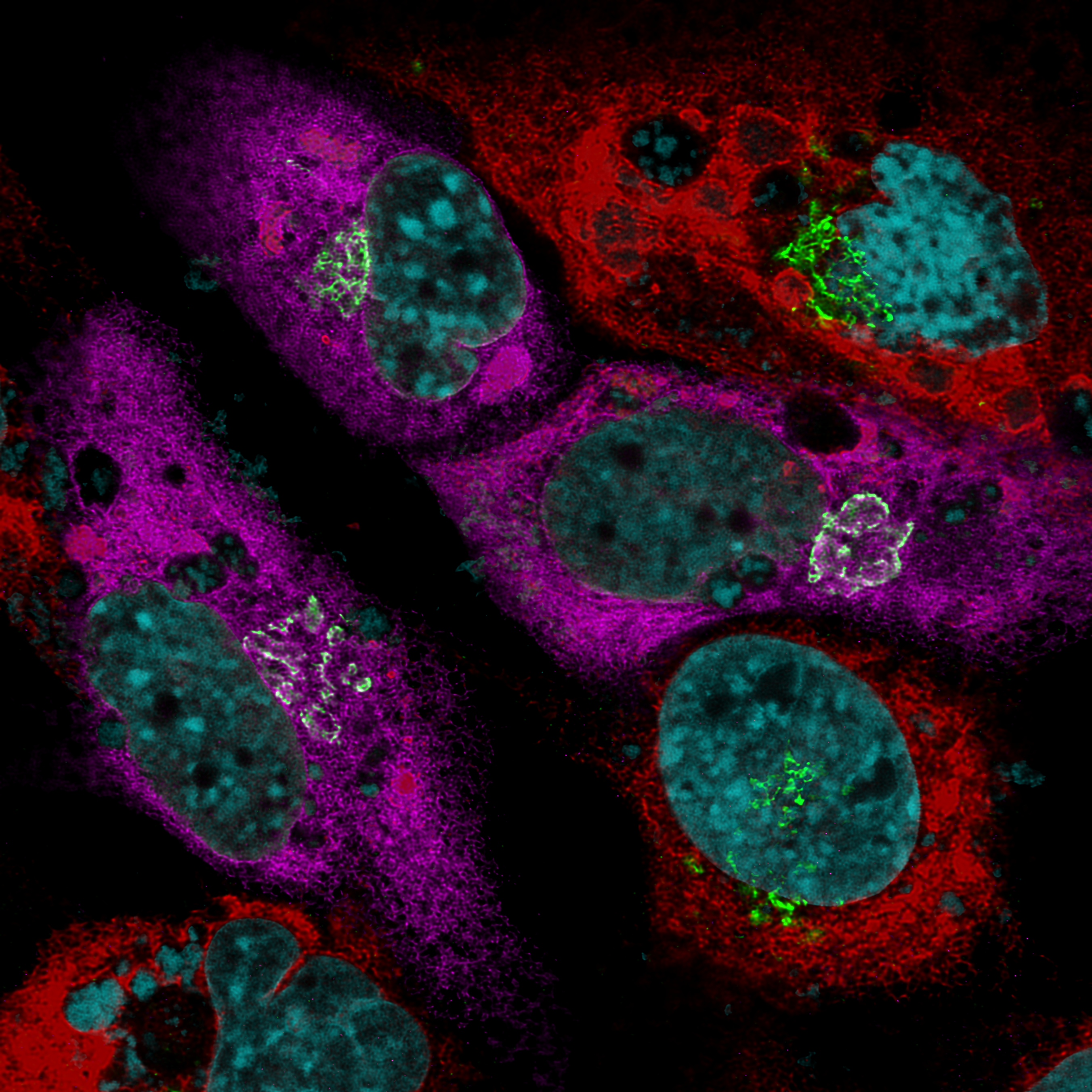

Supplement: Supplementary file 13 — Source data Fig. 8 [file 44319_2026_773_MOESM13_ESM.zip › Figure 8/Figure 8A/IF GRASP55KO GNPTAB WT HSP47_Myc_GM130 MERGE.tif]

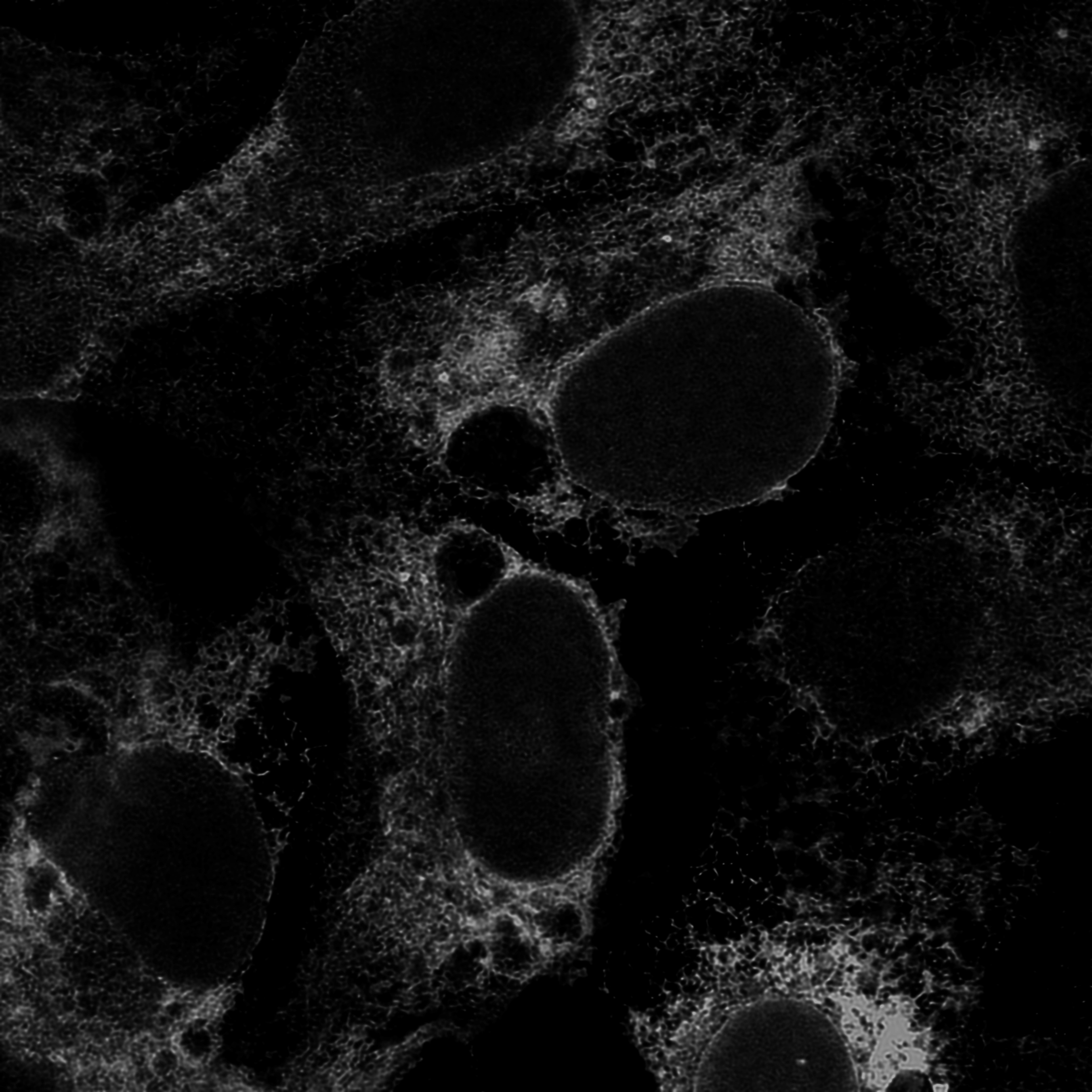

Supplement: Supplementary file 13 — Source data Fig. 8 [file 44319_2026_773_MOESM13_ESM.zip › Figure 8/Figure 8A/IF WT GNPTAB QELL HSP47.tif]

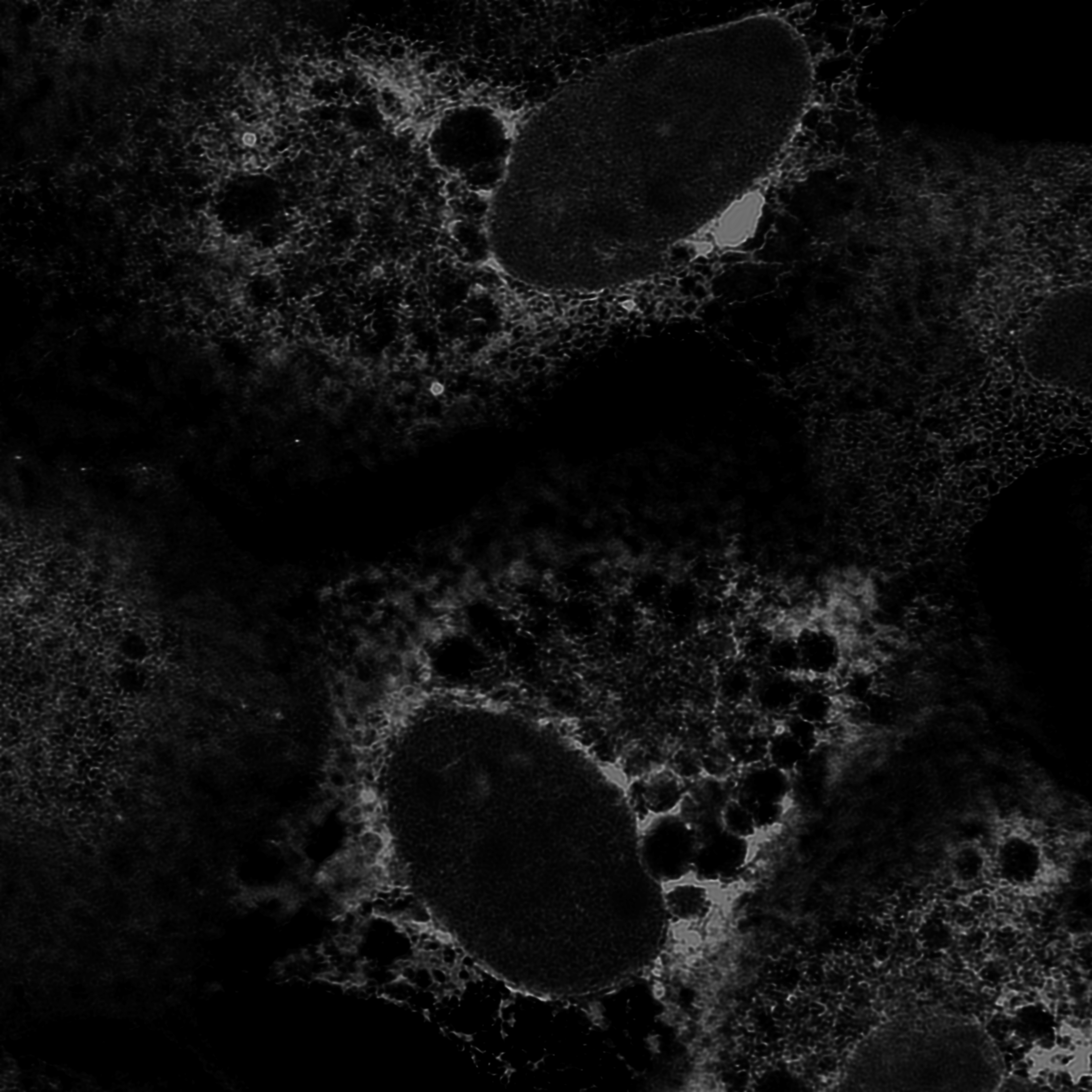

Supplement: Supplementary file 13 — Source data Fig. 8 [file 44319_2026_773_MOESM13_ESM.zip › Figure 8/Figure 8A/IF GRASP55KO GNPTAB QELL HSP47.tif]

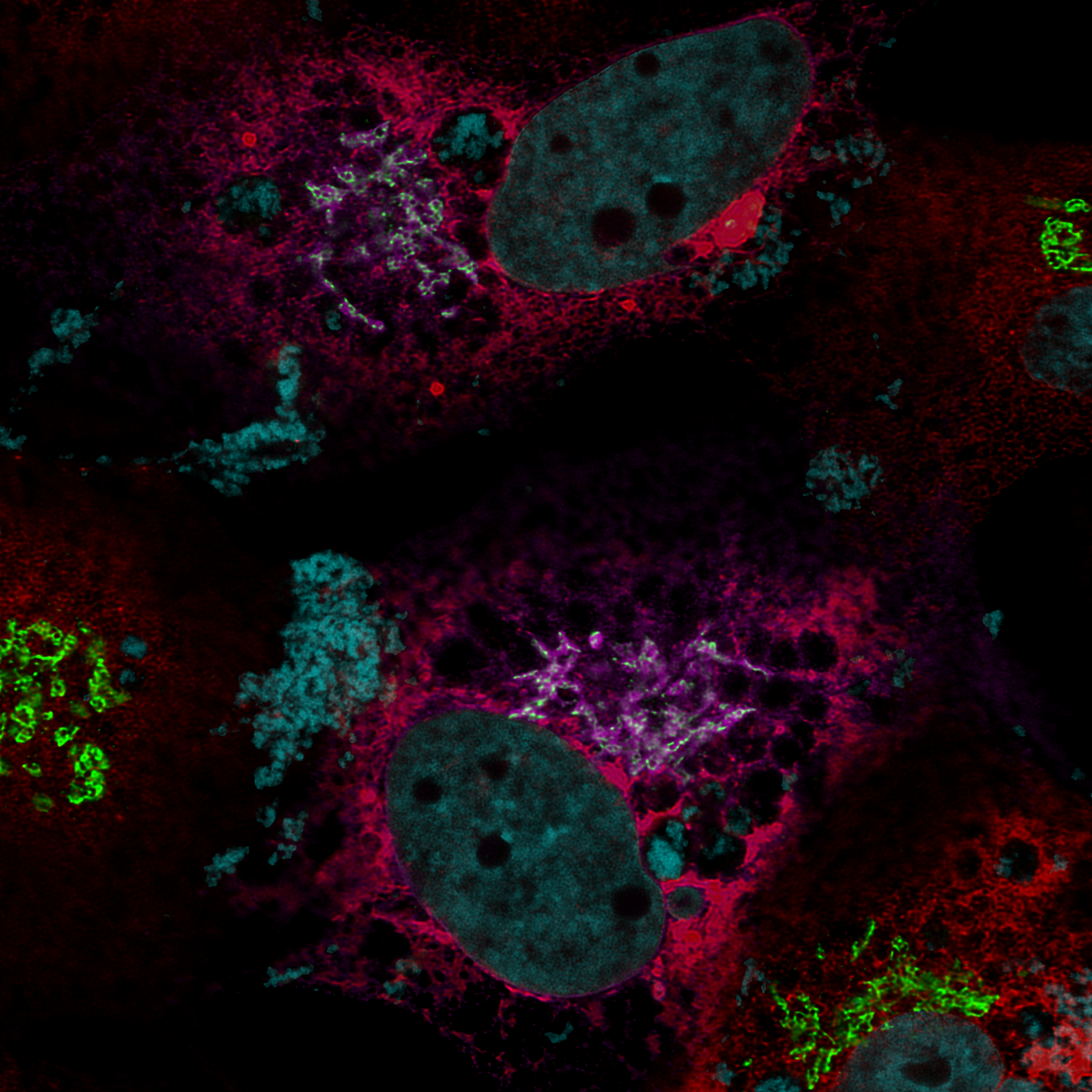

Supplement: Supplementary file 13 — Source data Fig. 8 [file 44319_2026_773_MOESM13_ESM.zip › Figure 8/Figure 8A/IF GRASP55KO GNPTAB QELL HSP47_Myc_GM130 MERGE.tif]

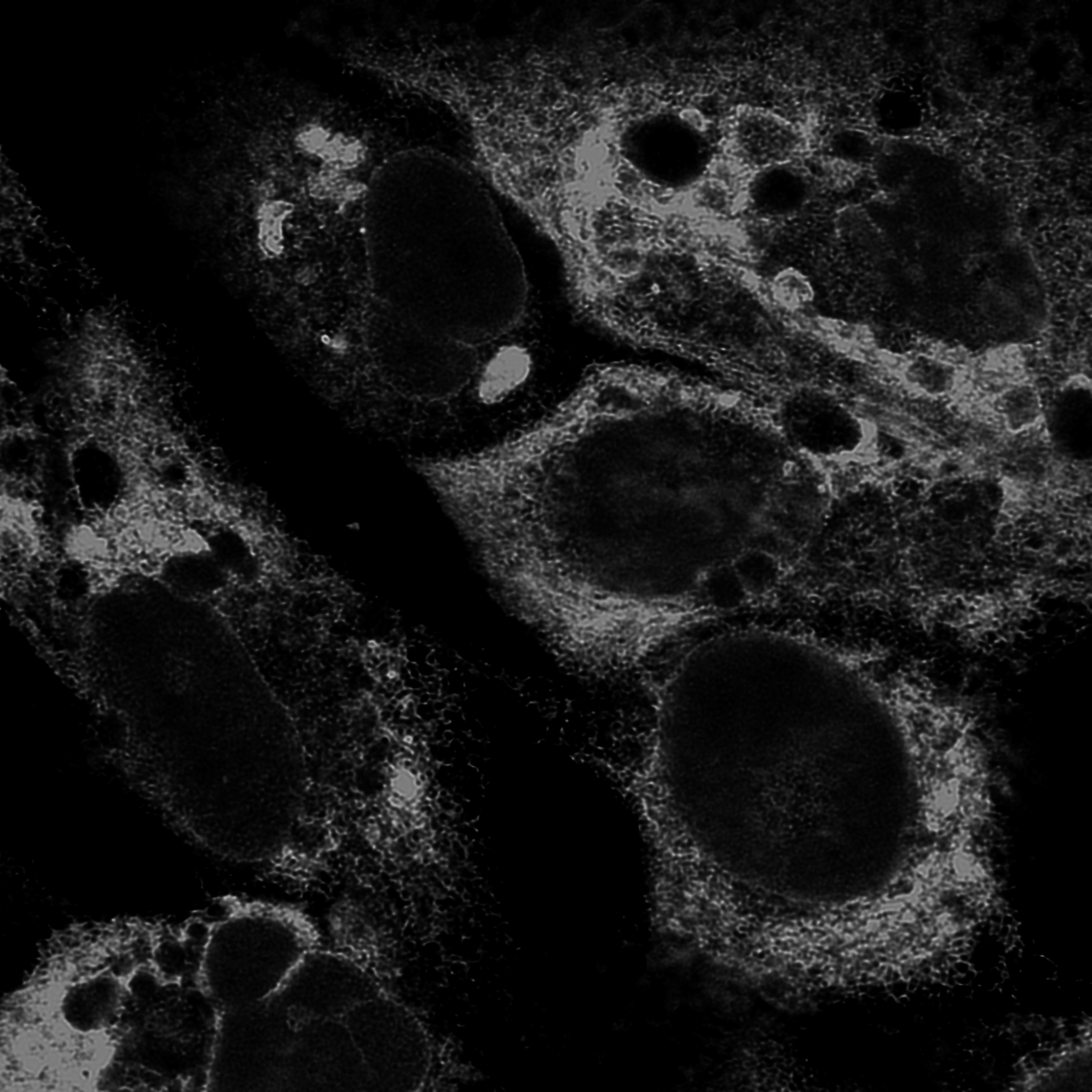

Supplement: Supplementary file 13 — Source data Fig. 8 [file 44319_2026_773_MOESM13_ESM.zip › Figure 8/Figure 8A/IF GRASP55KO GNPTAB WT HSP47.tif]

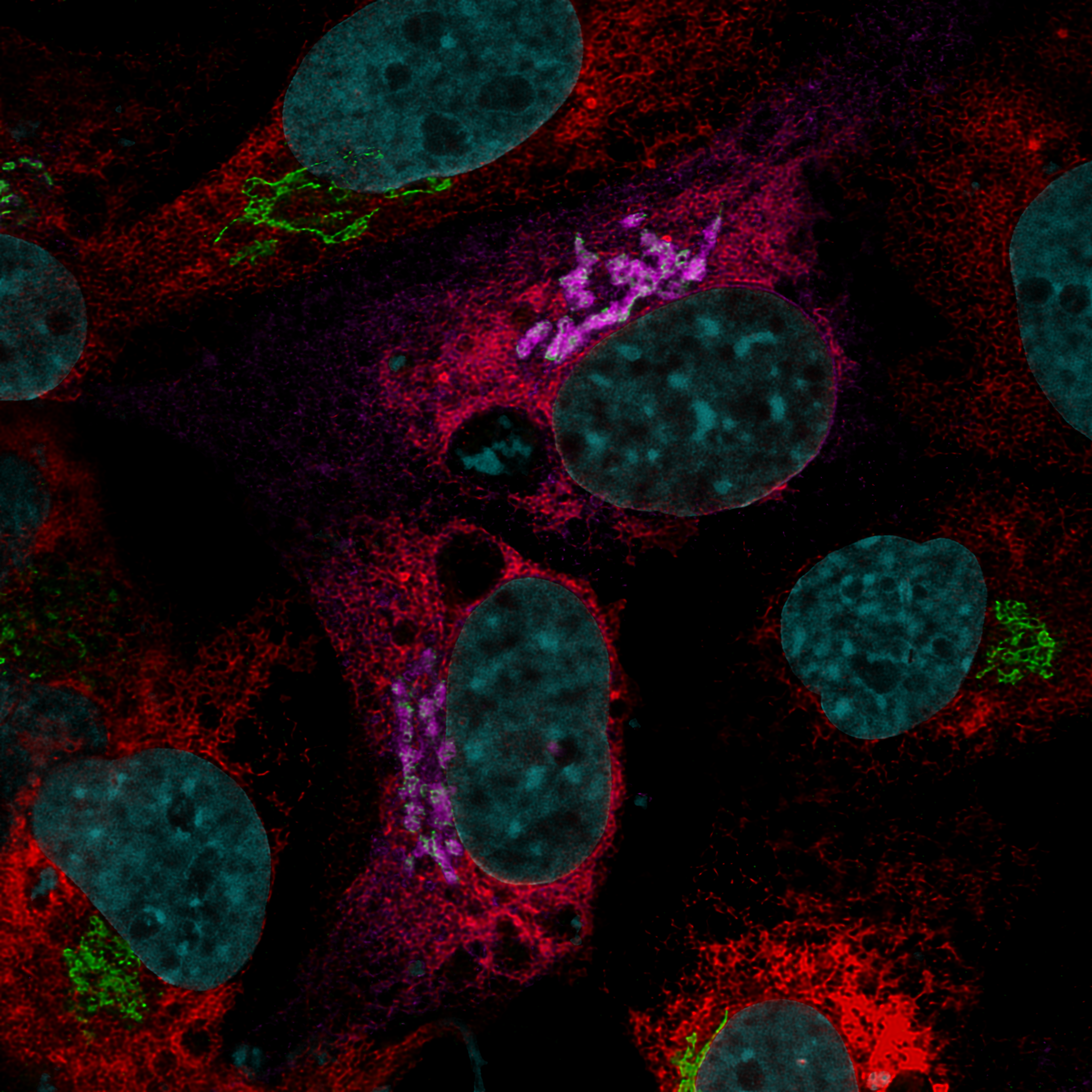

Supplement: Supplementary file 13 — Source data Fig. 8 [file 44319_2026_773_MOESM13_ESM.zip › Figure 8/Figure 8A/IF WT GNPTAB QELL HSP47_Myc_GM130 MERGE.tif]

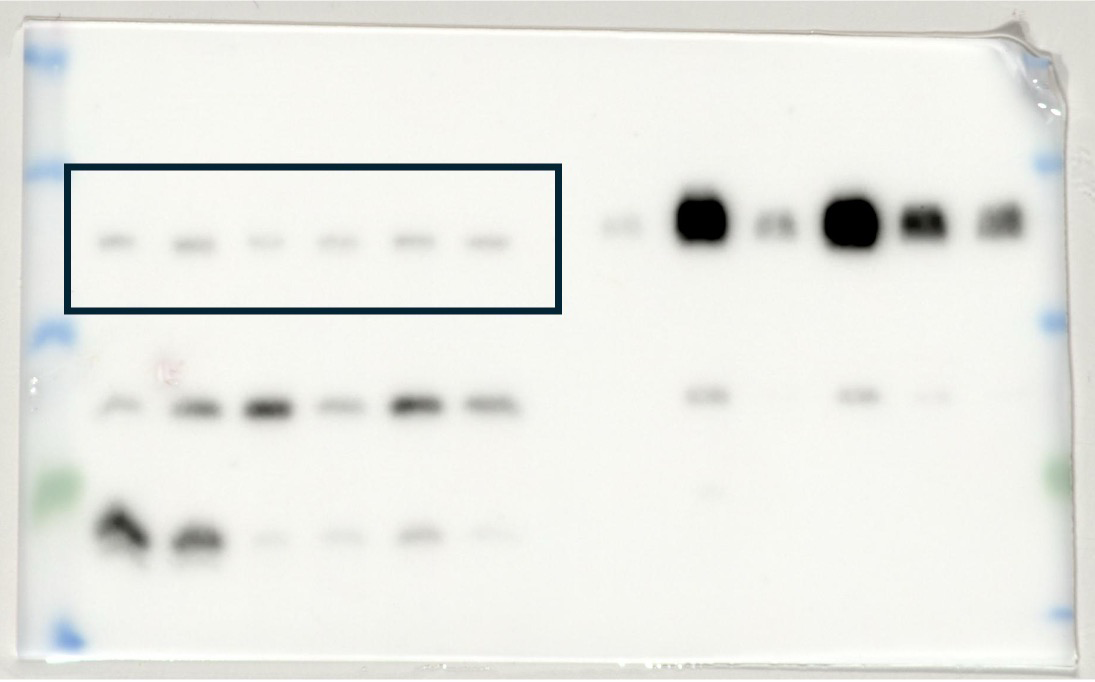

Supplement: Supplementary file 13 — Source data Fig. 8 [file 44319_2026_773_MOESM13_ESM.zip › Figure 8/Figure 8C/Western CTSB lysate.tif]

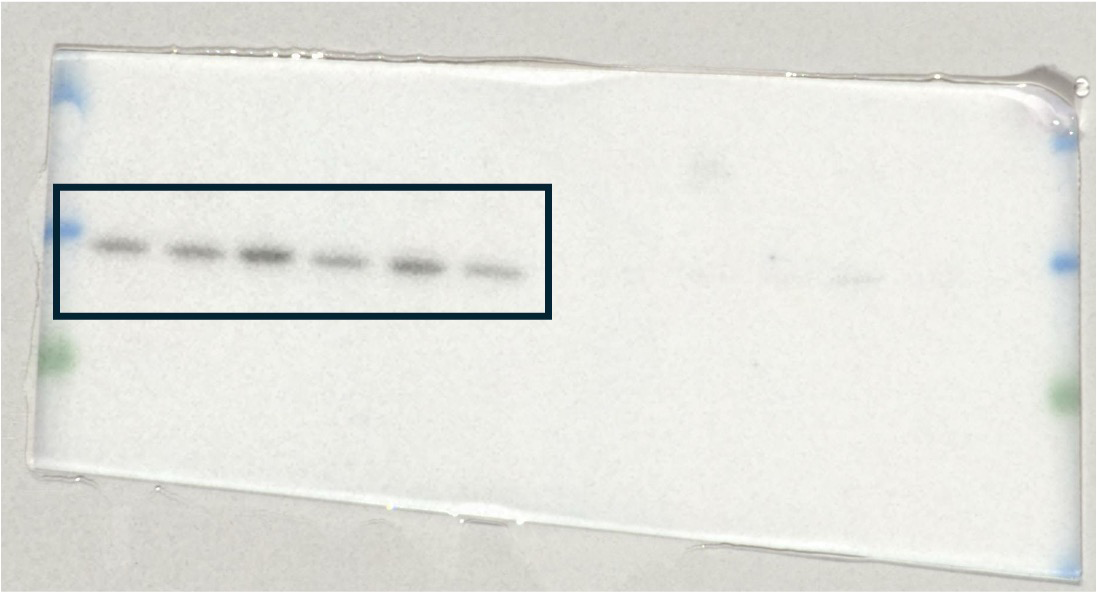

Supplement: Supplementary file 13 — Source data Fig. 8 [file 44319_2026_773_MOESM13_ESM.zip › Figure 8/Figure 8C/Western GNPTG lysate.tif]

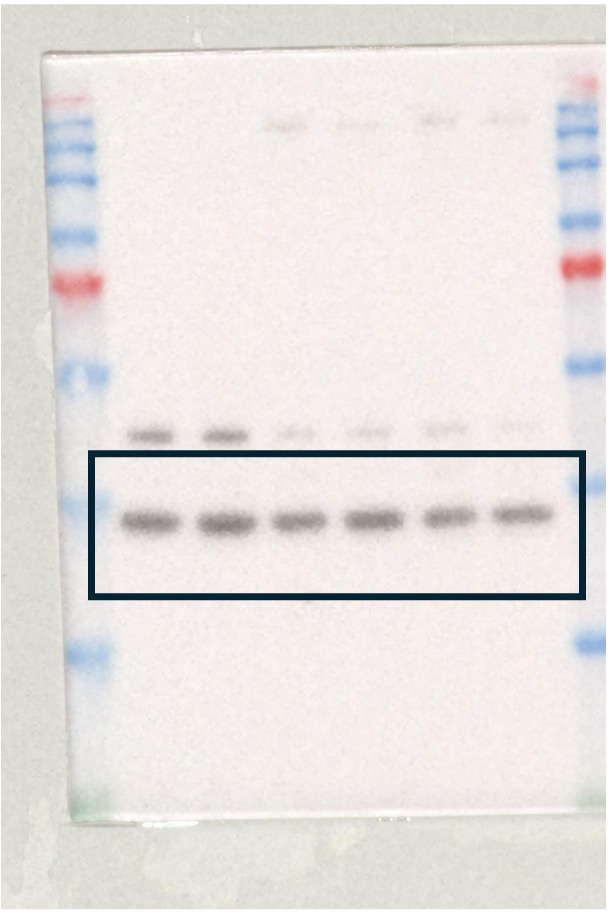

Supplement: Supplementary file 13 — Source data Fig. 8 [file 44319_2026_773_MOESM13_ESM.zip › Figure 8/Figure 8C/Western ACTIN lysate.tif]

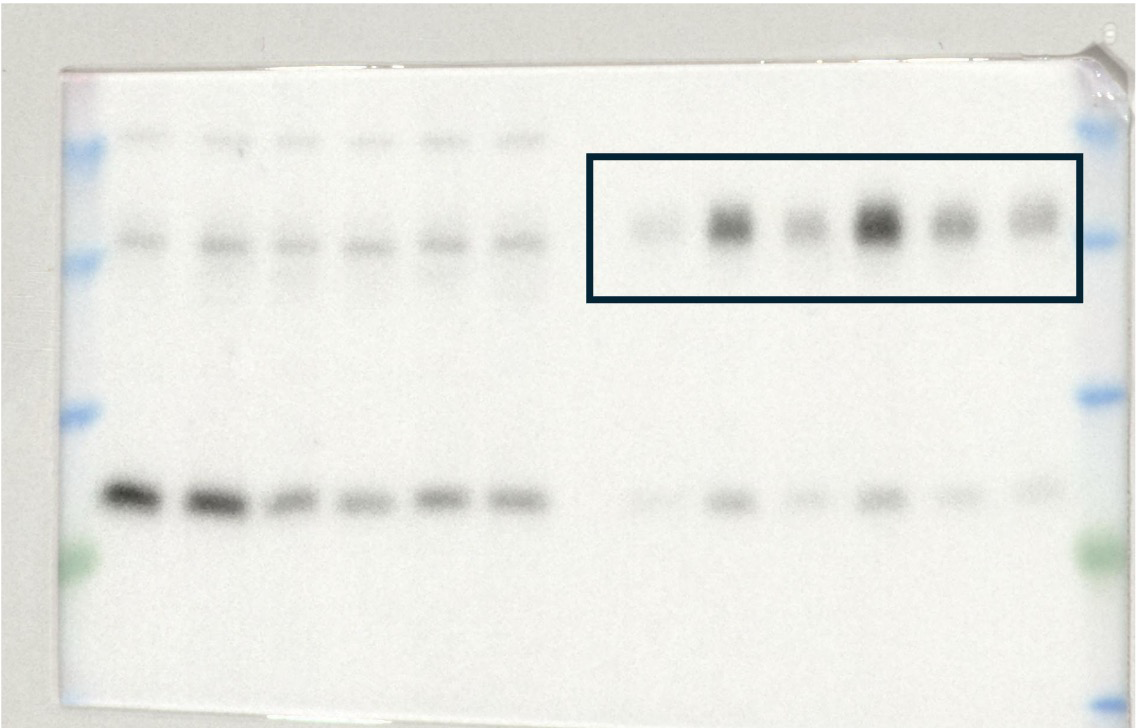

Supplement: Supplementary file 13 — Source data Fig. 8 [file 44319_2026_773_MOESM13_ESM.zip › Figure 8/Figure 8C/Western CTSD supernatant.tif]

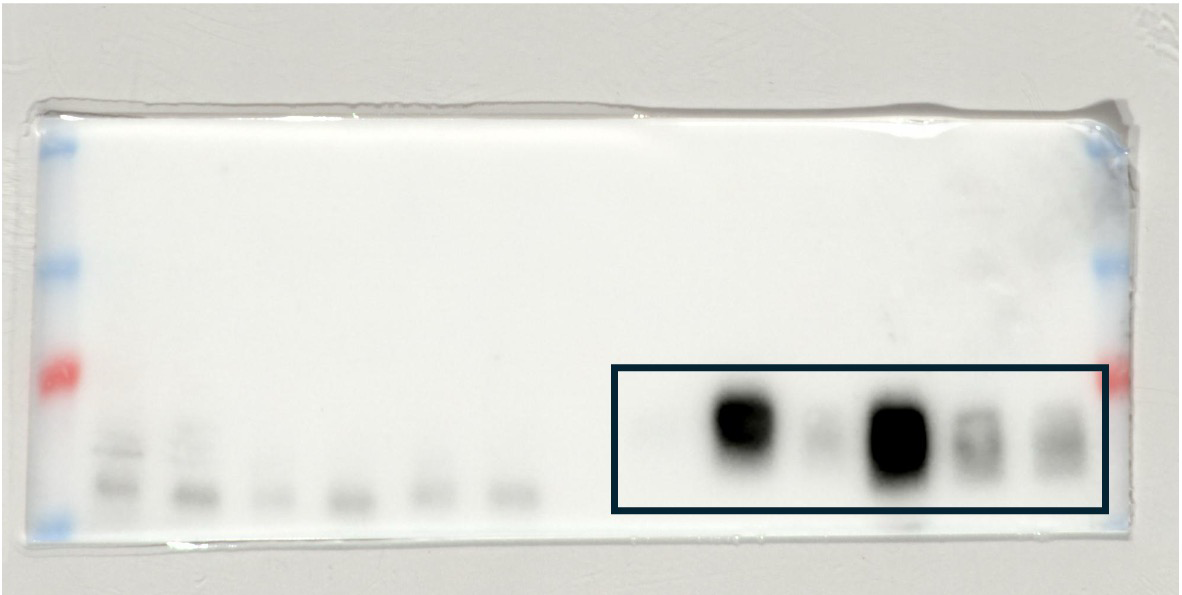

Supplement: Supplementary file 13 — Source data Fig. 8 [file 44319_2026_773_MOESM13_ESM.zip › Figure 8/Figure 8C/Western PSAP supernatant.tif]

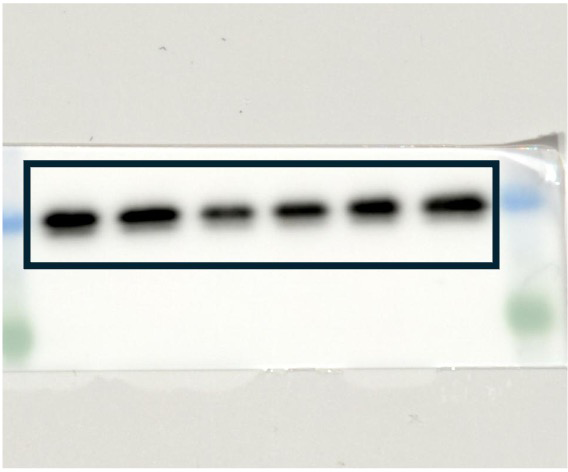

Supplement: Supplementary file 13 — Source data Fig. 8 [file 44319_2026_773_MOESM13_ESM.zip › Figure 8/Figure 8C/Western GOLPH3 lysate.tif]

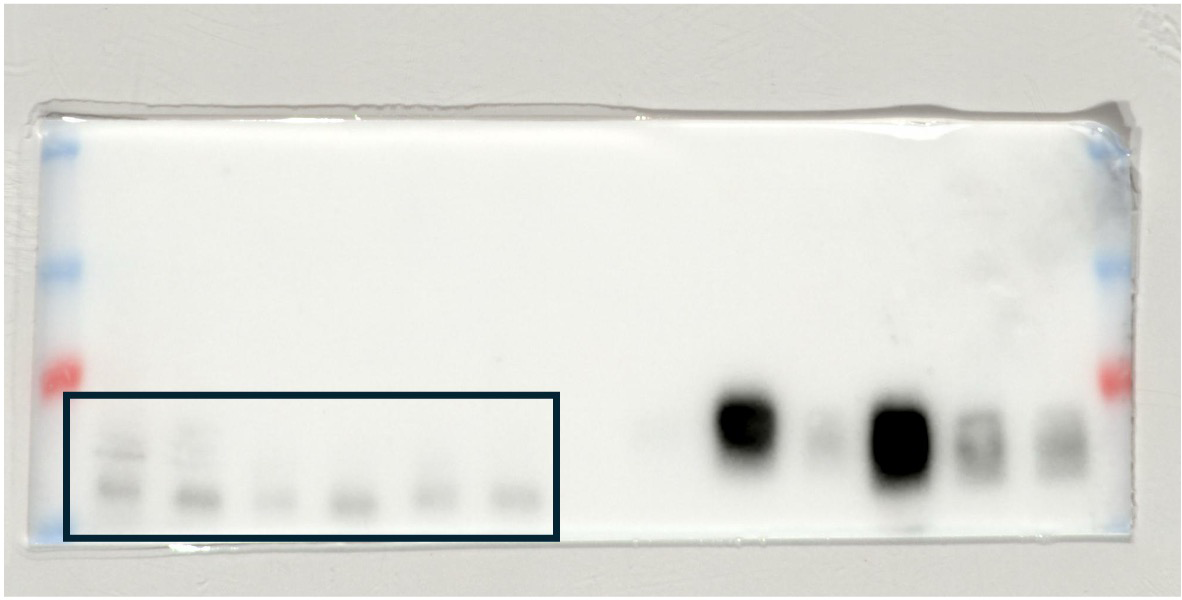

Supplement: Supplementary file 13 — Source data Fig. 8 [file 44319_2026_773_MOESM13_ESM.zip › Figure 8/Figure 8C/Western PSAP lysate.tif]

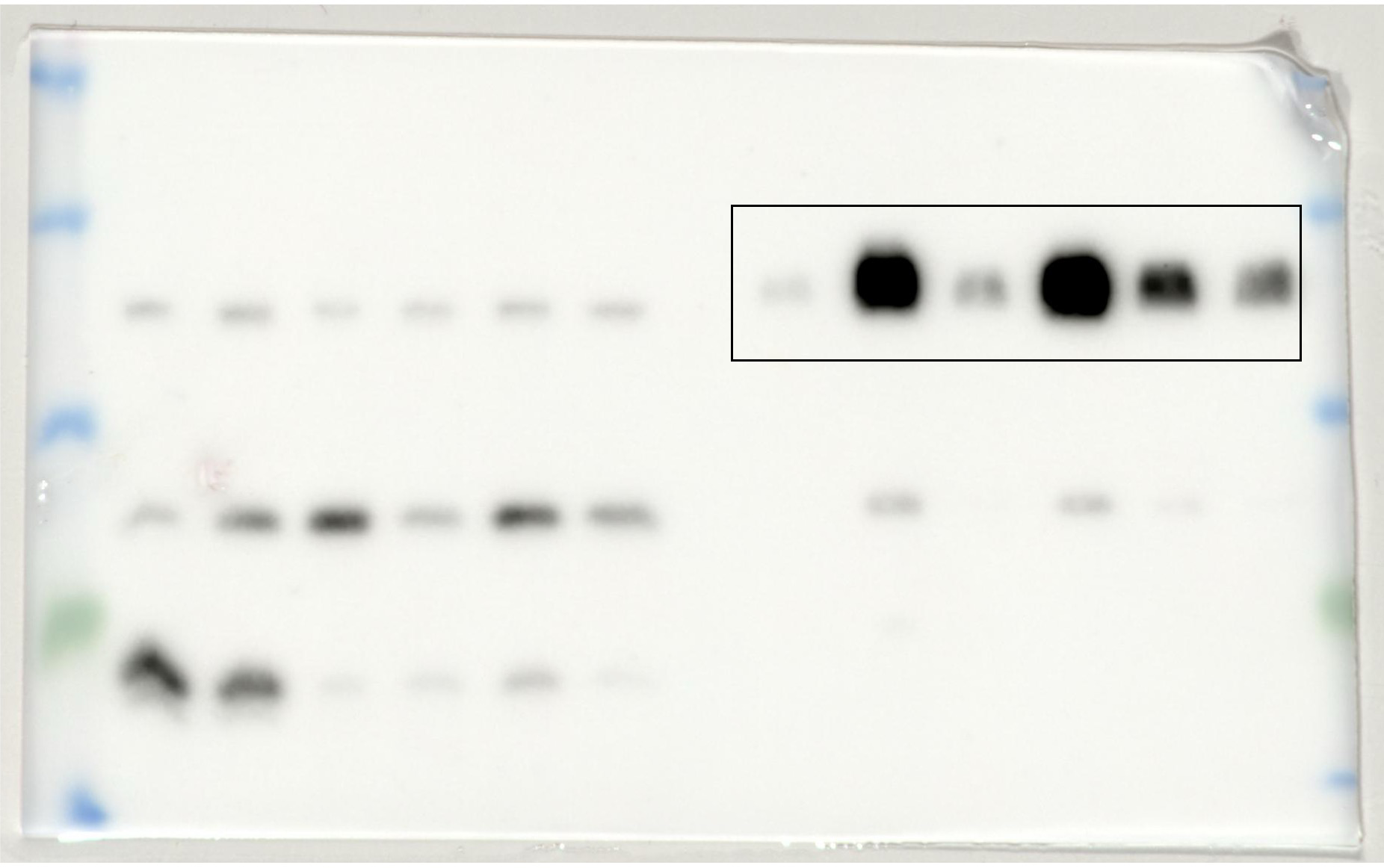

Supplement: Supplementary file 13 — Source data Fig. 8 [file 44319_2026_773_MOESM13_ESM.zip › Figure 8/Figure 8C/Western CTSB supernatant.tif]

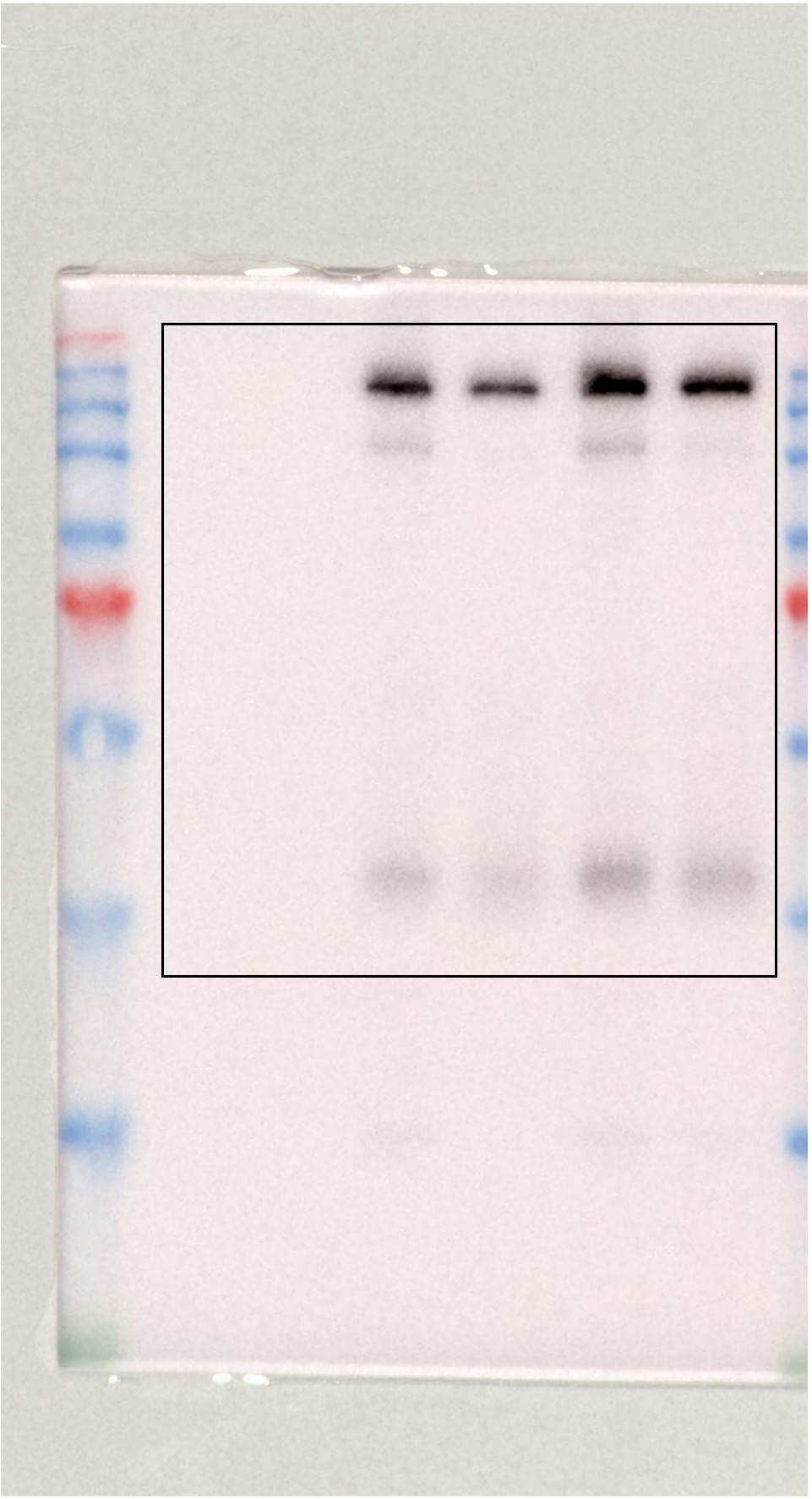

Supplement: Supplementary file 13 — Source data Fig. 8 [file 44319_2026_773_MOESM13_ESM.zip › Figure 8/Figure 8C/Western Myc lysate.tif]

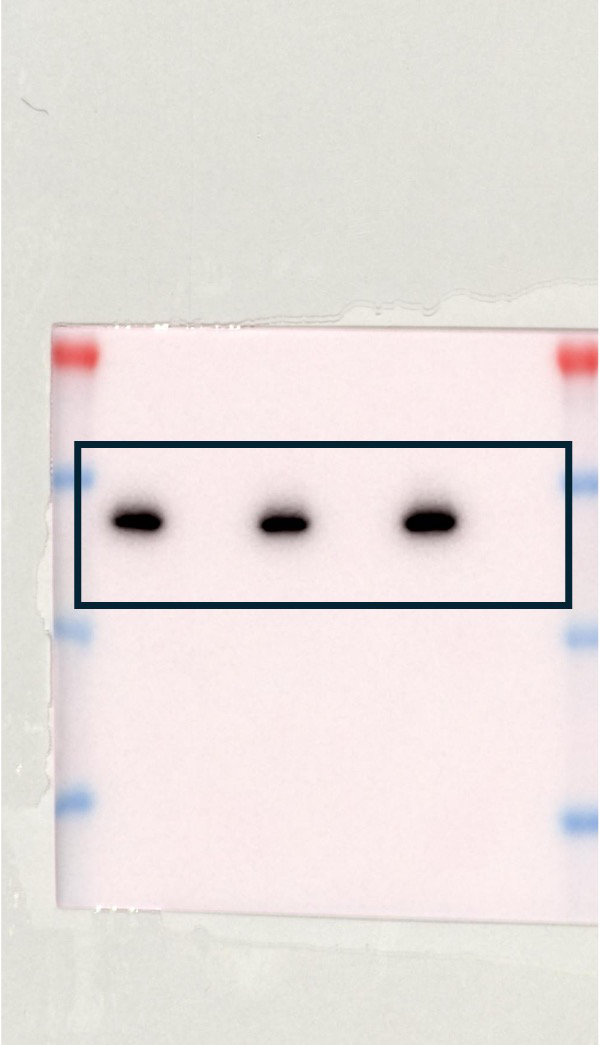

Supplement: Supplementary file 13 — Source data Fig. 8 [file 44319_2026_773_MOESM13_ESM.zip › Figure 8/Figure 8C/Western GRASP55 lysate.tif]

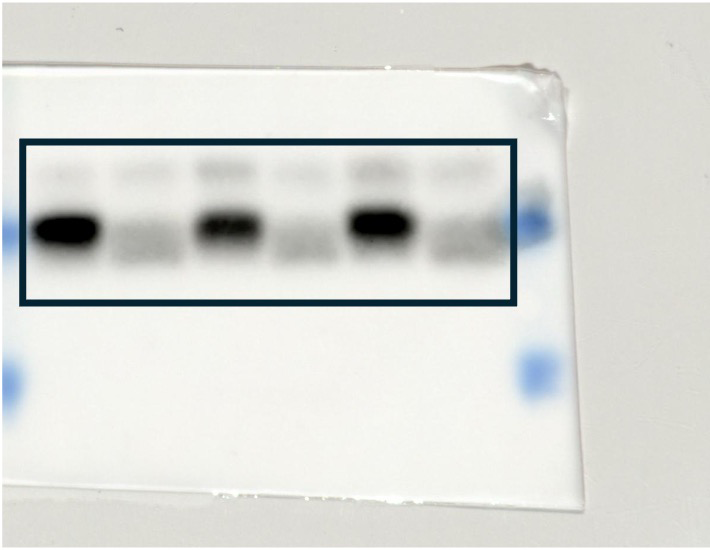

Supplement: Supplementary file 13 — Source data Fig. 8 [file 44319_2026_773_MOESM13_ESM.zip › Figure 8/Figure 8C/Western LYSET lysate.tif]

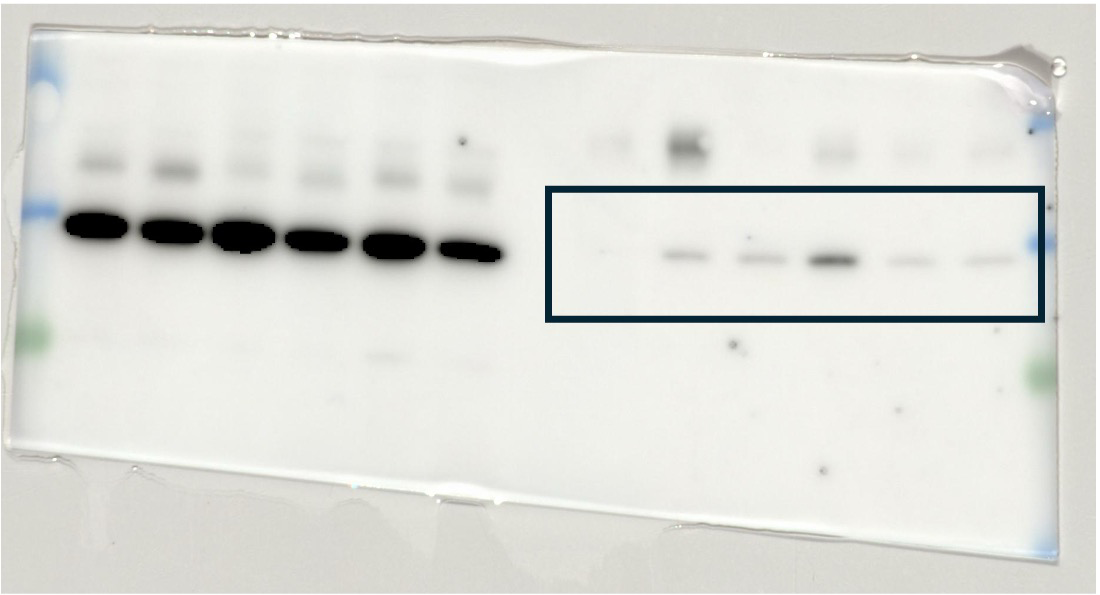

Supplement: Supplementary file 13 — Source data Fig. 8 [file 44319_2026_773_MOESM13_ESM.zip › Figure 8/Figure 8C/Western GNPTG medium.tif]

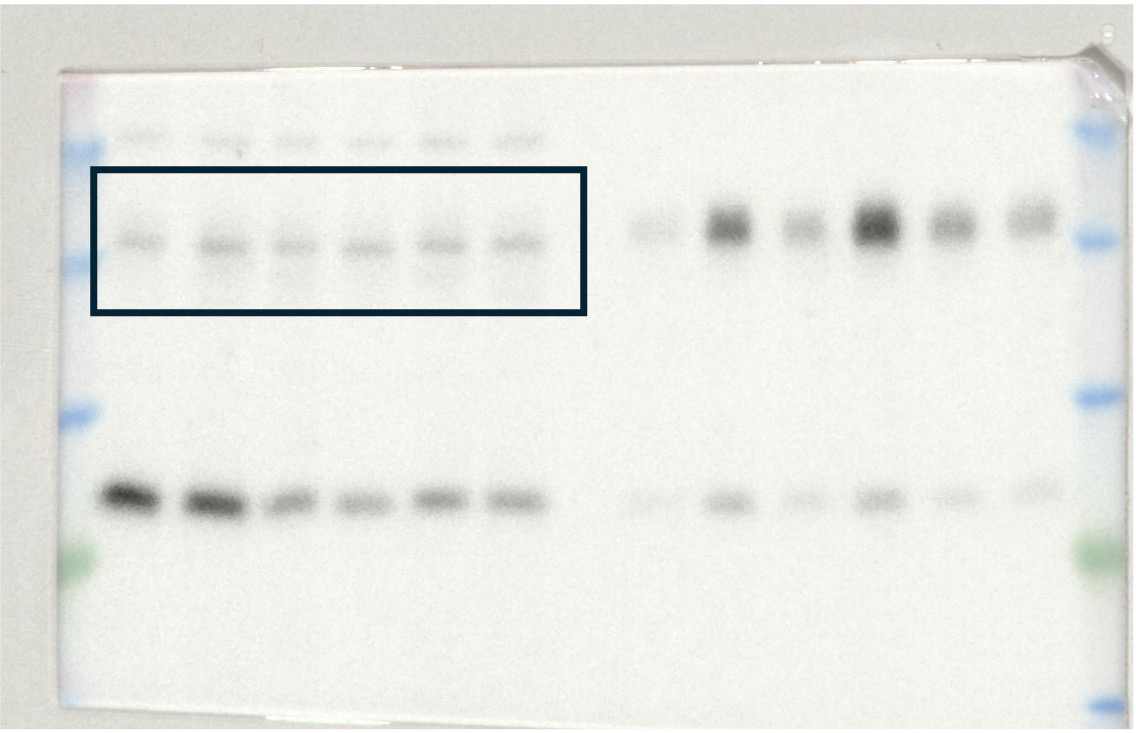

Supplement: Supplementary file 13 — Source data Fig. 8 [file 44319_2026_773_MOESM13_ESM.zip › Figure 8/Figure 8C/Western CTSD lysate.tif]

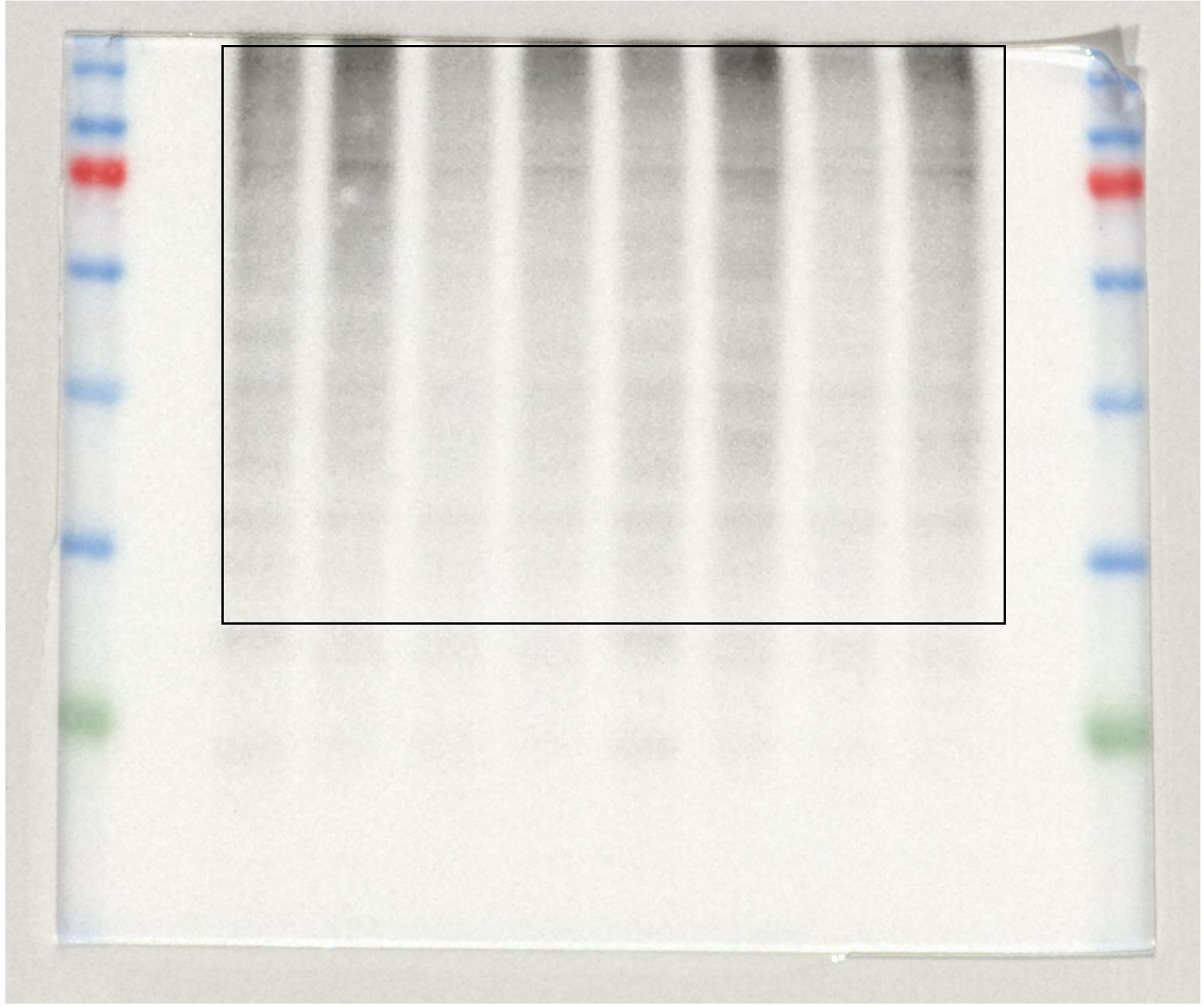

Supplement: Supplementary file 13 — Source data Fig. 8 [file 44319_2026_773_MOESM13_ESM.zip › Figure 8/Figure 8B/Western Ubiquitin.tif]

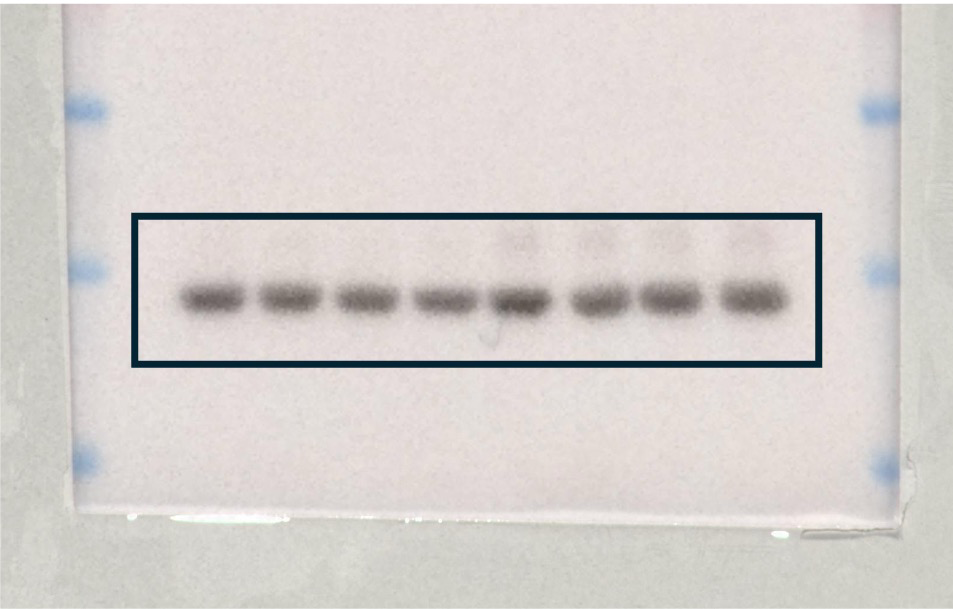

Supplement: Supplementary file 13 — Source data Fig. 8 [file 44319_2026_773_MOESM13_ESM.zip › Figure 8/Figure 8B/Western ACTIN.tif]

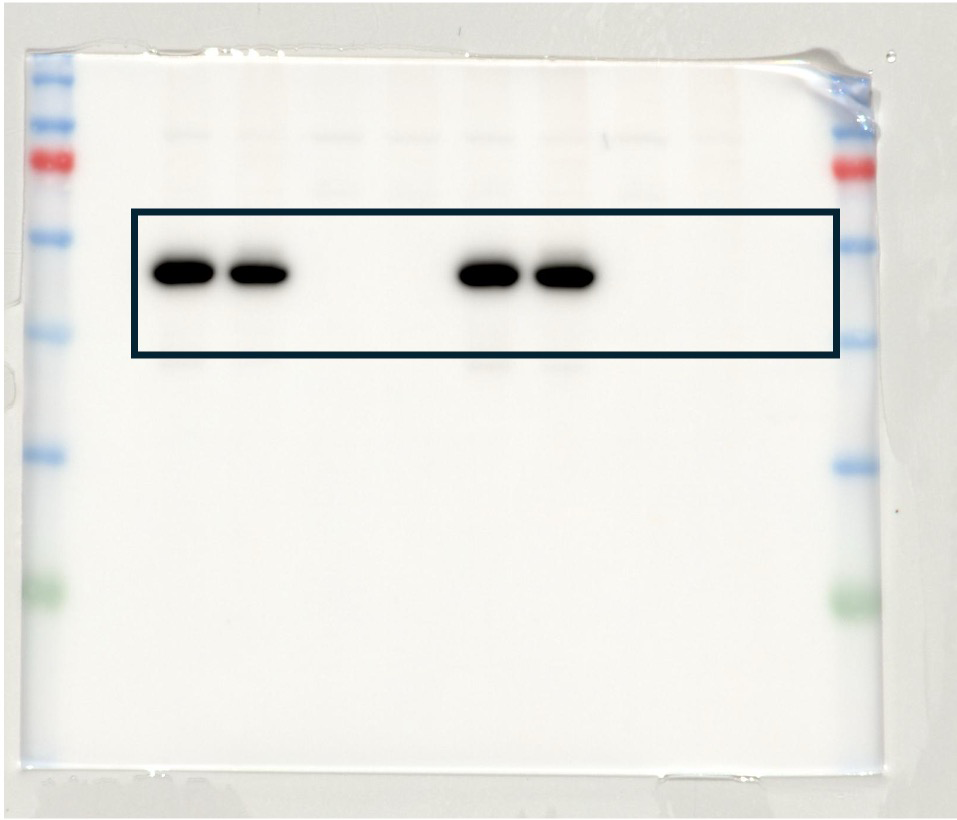

Supplement: Supplementary file 13 — Source data Fig. 8 [file 44319_2026_773_MOESM13_ESM.zip › Figure 8/Figure 8B/Western GRASP55.tif]

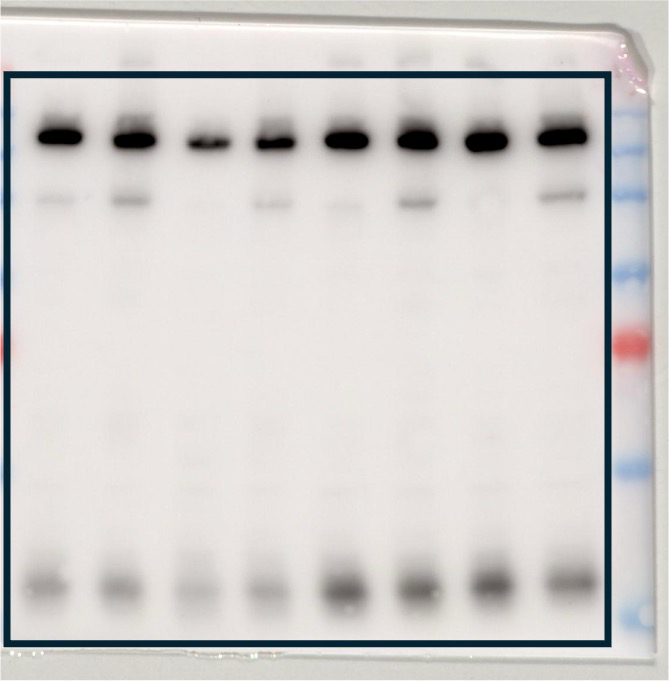

Supplement: Supplementary file 13 — Source data Fig. 8 [file 44319_2026_773_MOESM13_ESM.zip › Figure 8/Figure 8B/Western MYC.tif]

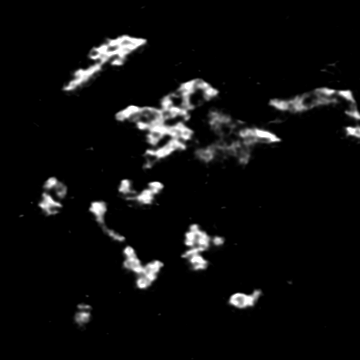

Supplement: Supplementary file 14 — Figure EV1 Source Data [file 44319_2026_773_MOESM14_ESM.zip › Figure EV1/Figure EV 1A/IF WT 1h RUSH GM130 inset.tif]

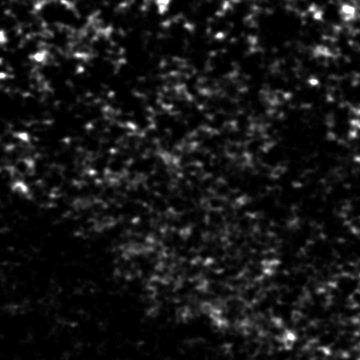

Supplement: Supplementary file 14 — Figure EV1 Source Data [file 44319_2026_773_MOESM14_ESM.zip › Figure EV1/Figure EV 1A/IF GR55KO UT RUSH PSAP-SBP inset.tif]

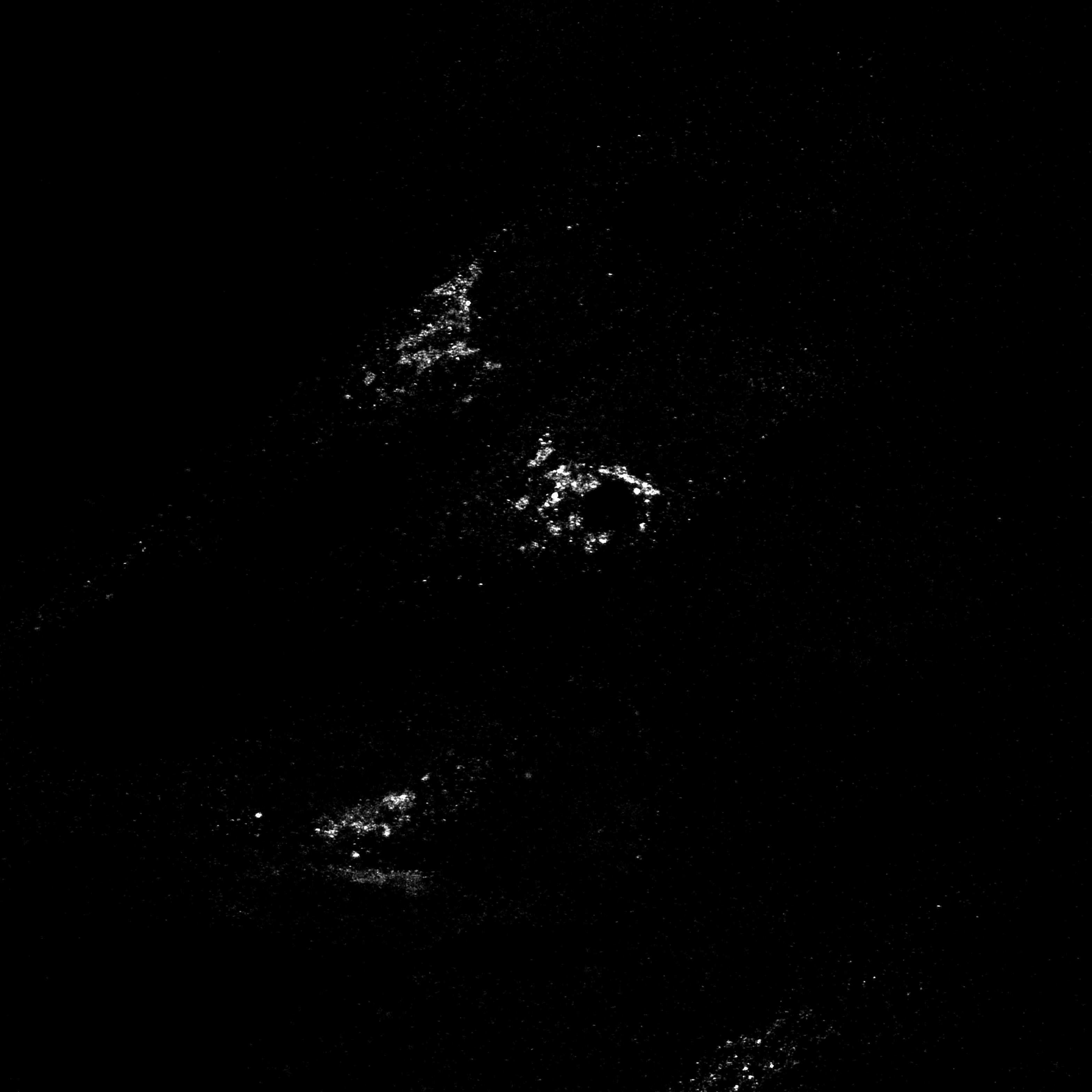

Supplement: Supplementary file 14 — Figure EV1 Source Data [file 44319_2026_773_MOESM14_ESM.zip › Figure EV1/Figure EV 1A/IF WT 1h RUSH PSAP-SBP..tif]

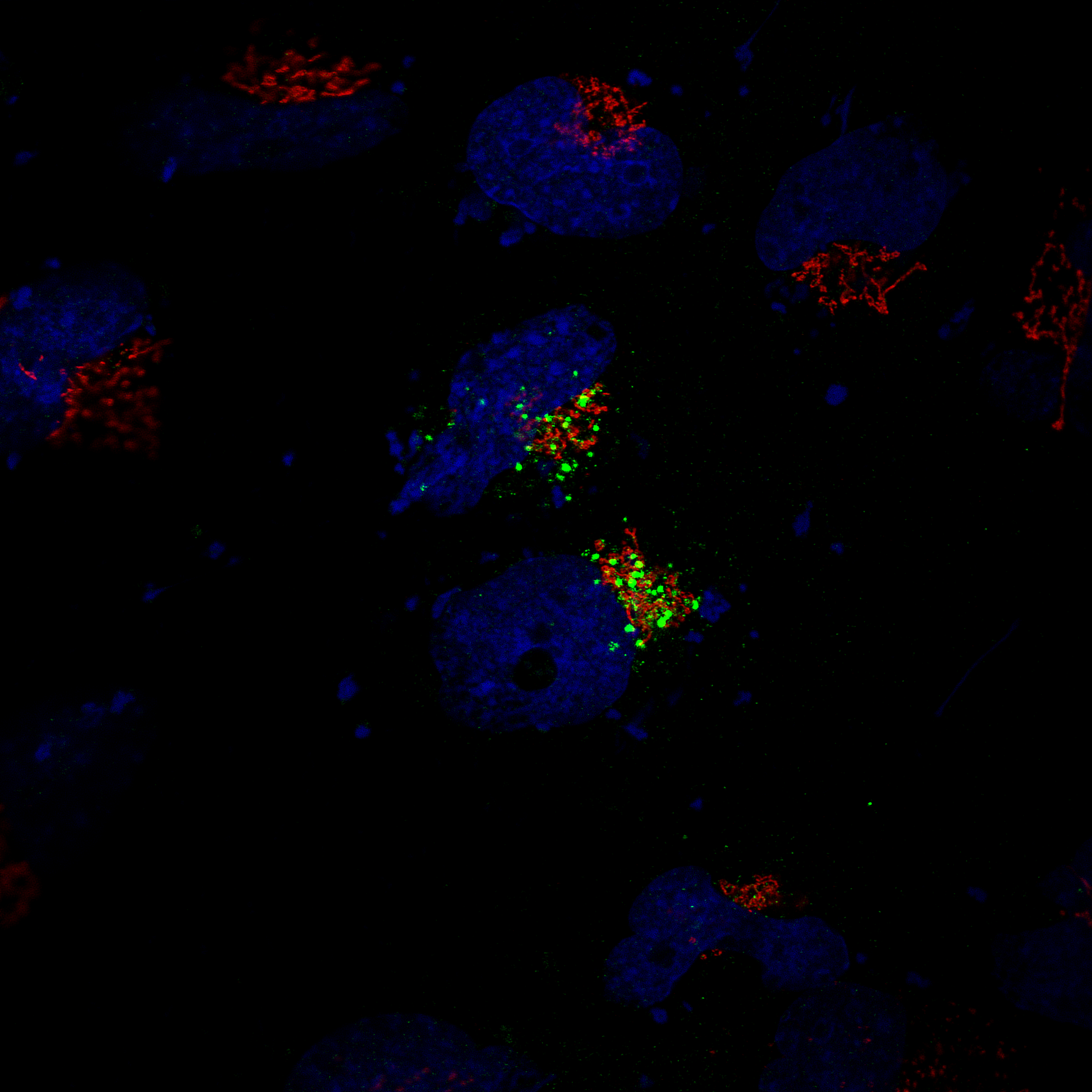

Supplement: Supplementary file 14 — Figure EV1 Source Data [file 44319_2026_773_MOESM14_ESM.zip › Figure EV1/Figure EV 1A/IF WT 2h RUSH PSAP-SBP_GM130 MERGE.tif]

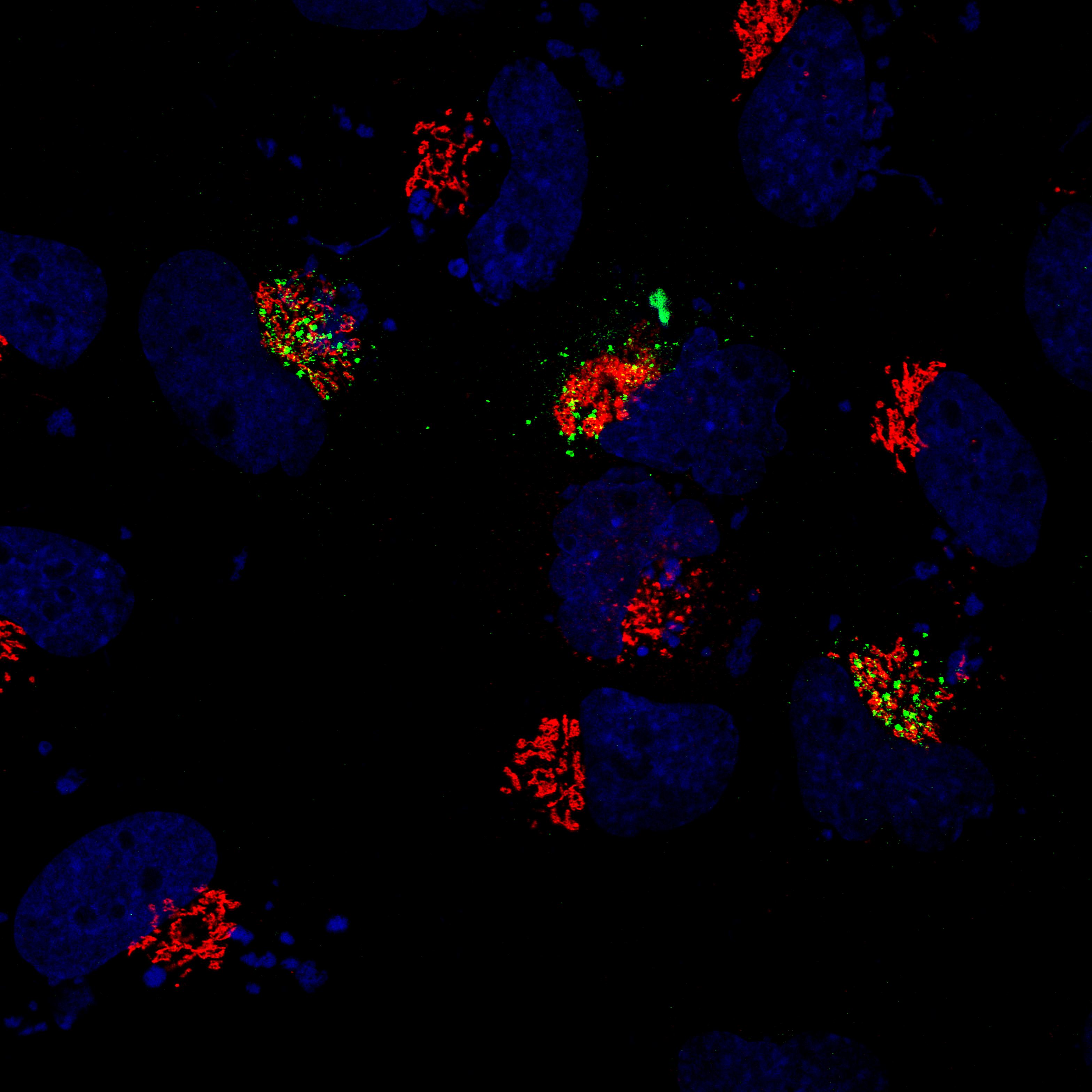

Supplement: Supplementary file 14 — Figure EV1 Source Data [file 44319_2026_773_MOESM14_ESM.zip › Figure EV1/Figure EV 1A/IF WT 4h RUSH PSAP-SBP_GM130 MERGE.tif]

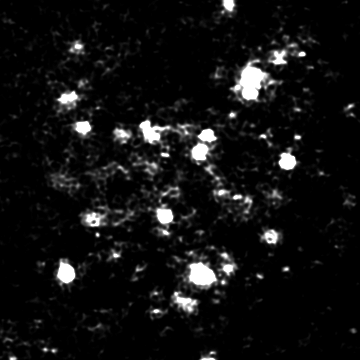

Supplement: Supplementary file 14 — Figure EV1 Source Data [file 44319_2026_773_MOESM14_ESM.zip › Figure EV1/Figure EV 1A/IF WT 2h RUSH PSAP-SBP inset.tif]

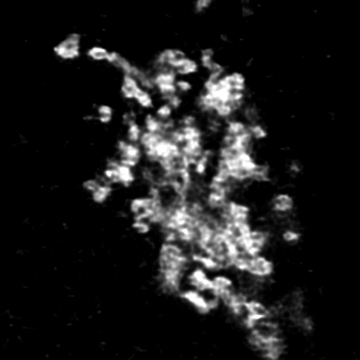

Supplement: Supplementary file 14 — Figure EV1 Source Data [file 44319_2026_773_MOESM14_ESM.zip › Figure EV1/Figure EV 1A/IF GR55KO UT RUSH GM130 inset.tif]

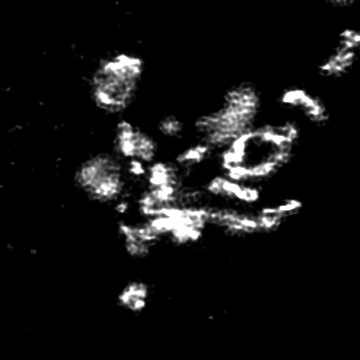

Supplement: Supplementary file 14 — Figure EV1 Source Data [file 44319_2026_773_MOESM14_ESM.zip › Figure EV1/Figure EV 1A/IF GR55KO 2h RUSH GM130 inset.tif]

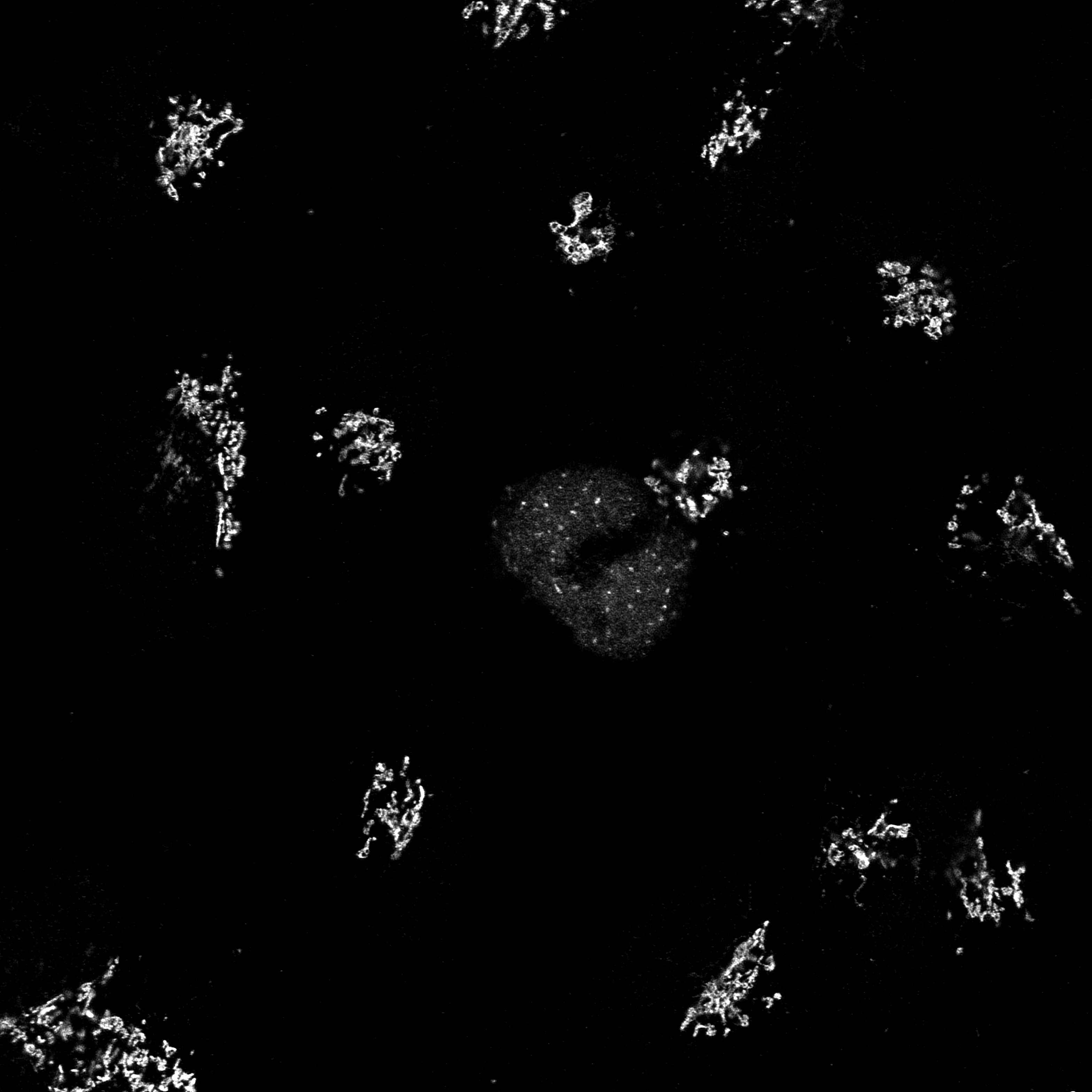

Supplement: Supplementary file 14 — Figure EV1 Source Data [file 44319_2026_773_MOESM14_ESM.zip › Figure EV1/Figure EV 1A/IF GR55KO 4h RUSH GM130.tif]

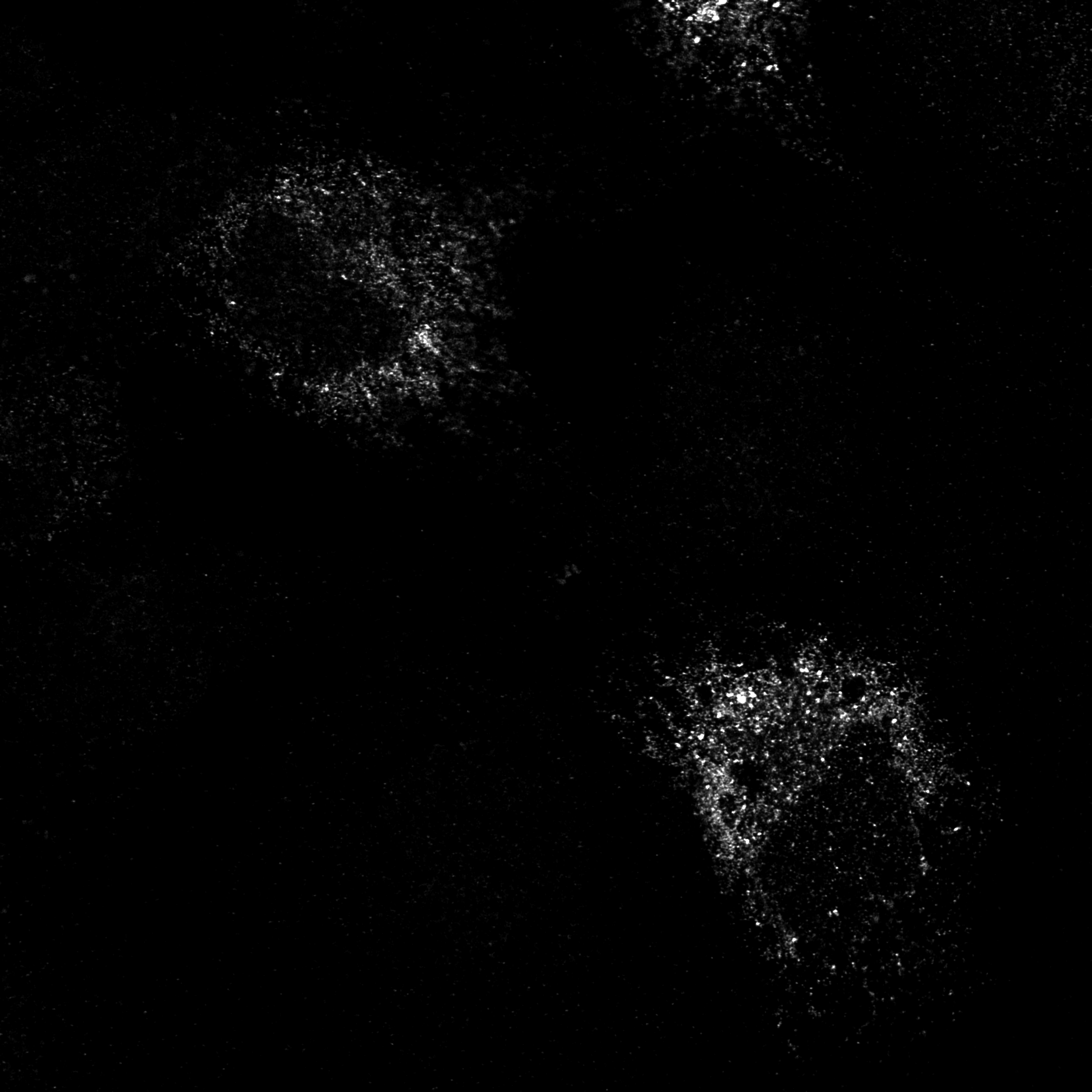

Supplement: Supplementary file 14 — Figure EV1 Source Data [file 44319_2026_773_MOESM14_ESM.zip › Figure EV1/Figure EV 1A/IF WT UT RUSH PSAP-SBP.tif]

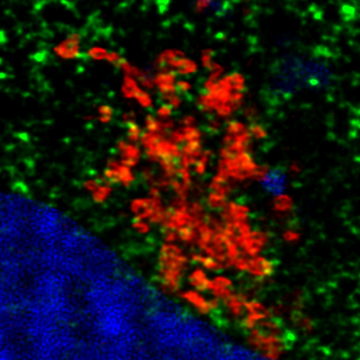

Supplement: Supplementary file 14 — Figure EV1 Source Data [file 44319_2026_773_MOESM14_ESM.zip › Figure EV1/Figure EV 1A/IF GR55KO UT RUSH PSAP-SBP_GM130 MERGE inset.tif]

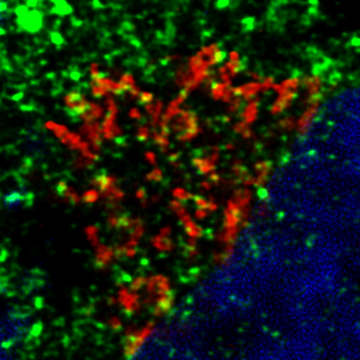

Supplement: Supplementary file 14 — Figure EV1 Source Data [file 44319_2026_773_MOESM14_ESM.zip › Figure EV1/Figure EV 1A/IF WT UT RUSH PSAP-SBP_GM130 MERGE inset.tif]

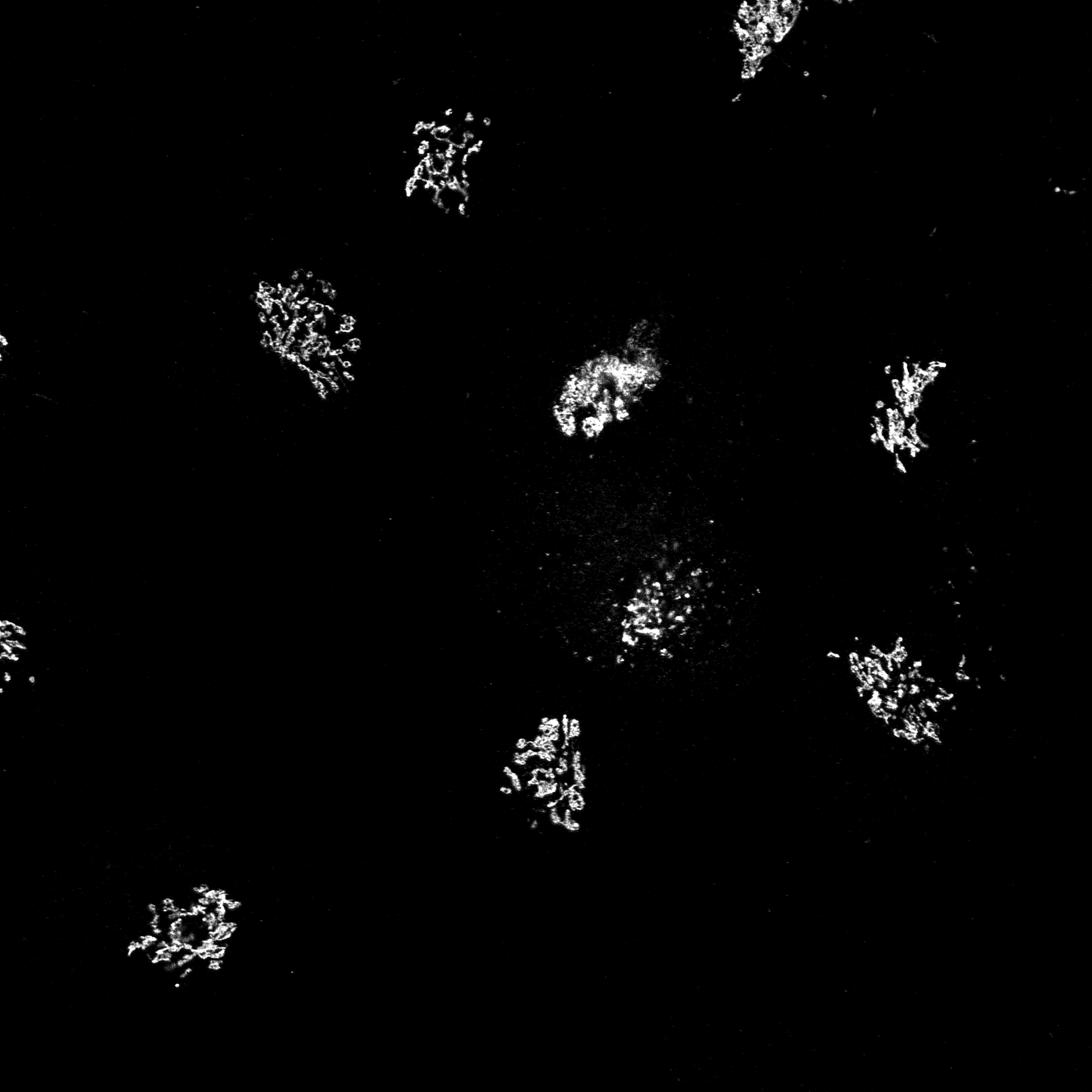

Supplement: Supplementary file 14 — Figure EV1 Source Data [file 44319_2026_773_MOESM14_ESM.zip › Figure EV1/Figure EV 1A/IF WT 4h RUSH GM130.tif]

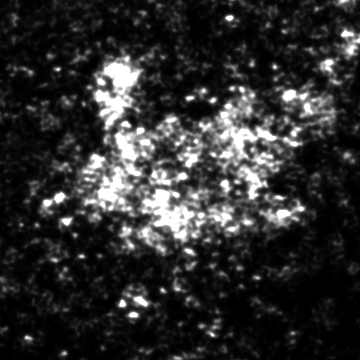

Supplement: Supplementary file 14 — Figure EV1 Source Data [file 44319_2026_773_MOESM14_ESM.zip › Figure EV1/Figure EV 1A/IF GR55KO 2h RUSH PSAP-SBP inset.tif]

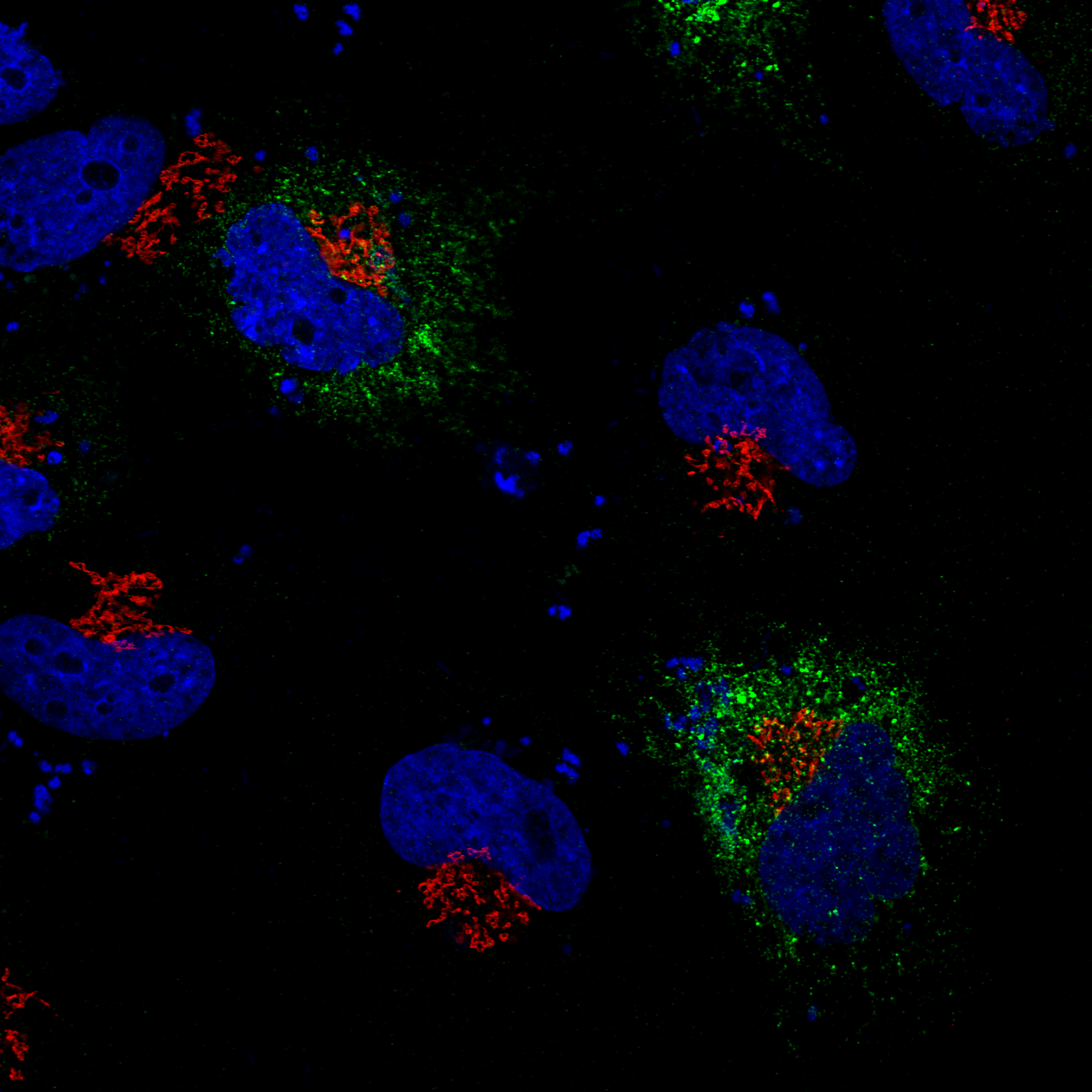

Supplement: Supplementary file 14 — Figure EV1 Source Data [file 44319_2026_773_MOESM14_ESM.zip › Figure EV1/Figure EV 1A/IF WT UT RUSH PSAP-SBP_GM130 MERGE.tif]

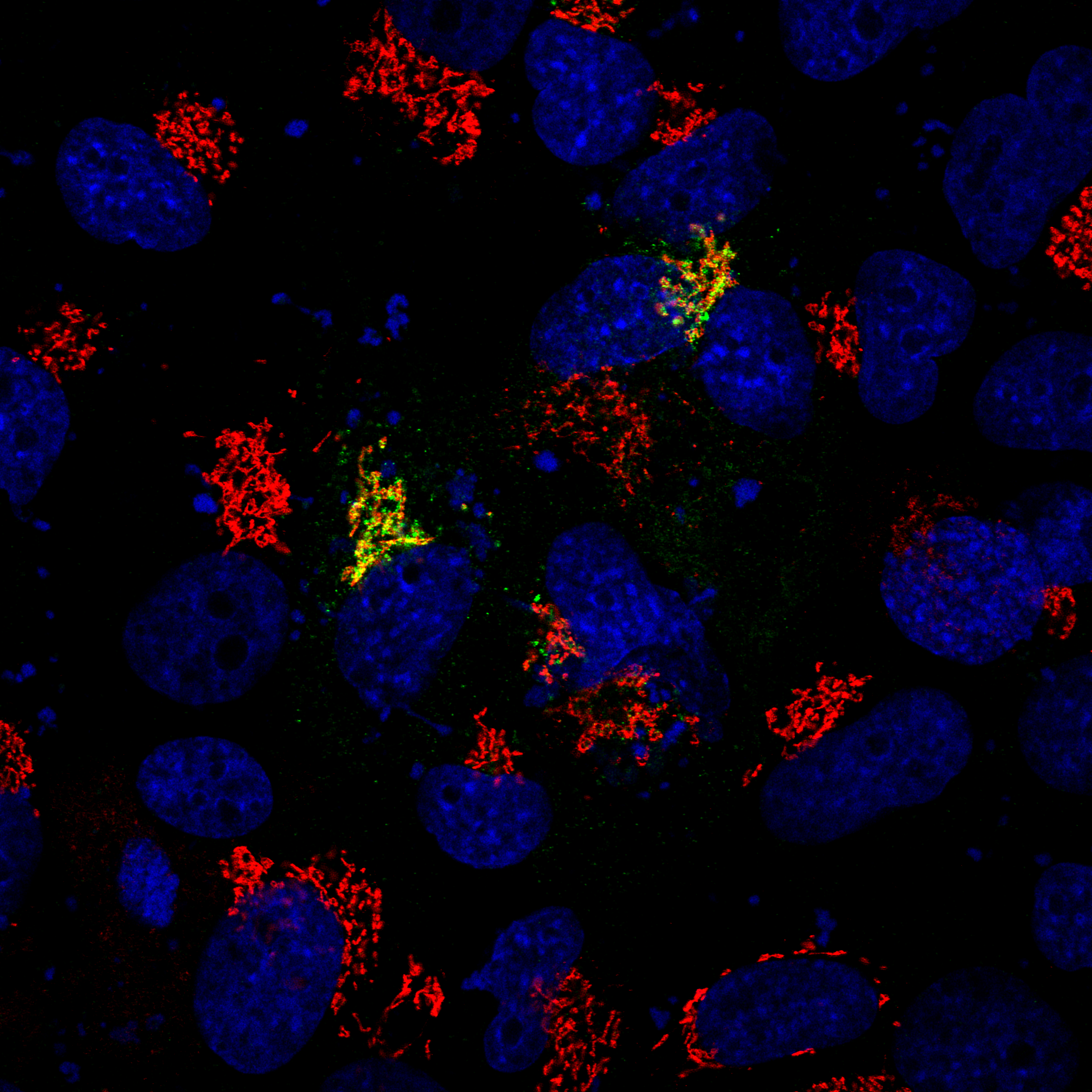

Supplement: Supplementary file 14 — Figure EV1 Source Data [file 44319_2026_773_MOESM14_ESM.zip › Figure EV1/Figure EV 1A/IF GR55KO 1h RUSH PSAP-SBP_GM130 MERGE.tif]

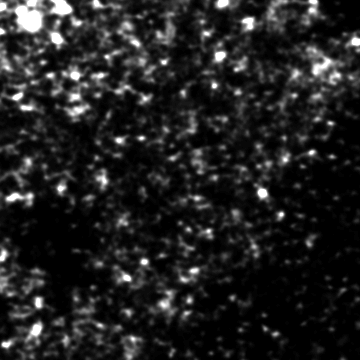

Supplement: Supplementary file 14 — Figure EV1 Source Data [file 44319_2026_773_MOESM14_ESM.zip › Figure EV1/Figure EV 1A/IF WT UT RUSH PSAP-SBP inset.tif]

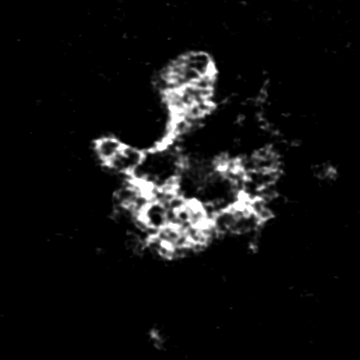

Supplement: Supplementary file 14 — Figure EV1 Source Data [file 44319_2026_773_MOESM14_ESM.zip › Figure EV1/Figure EV 1A/IF GR55KO 4h RUSH GM130 inset.tif]

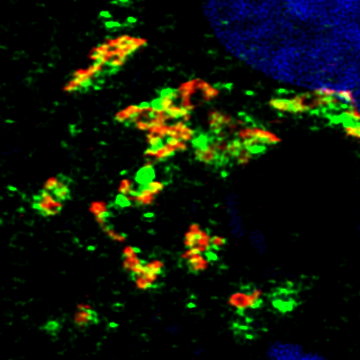

Supplement: Supplementary file 14 — Figure EV1 Source Data [file 44319_2026_773_MOESM14_ESM.zip › Figure EV1/Figure EV 1A/IF WT 1h RUSH PSAP-SBP_GM130 MERGE inset.tif]

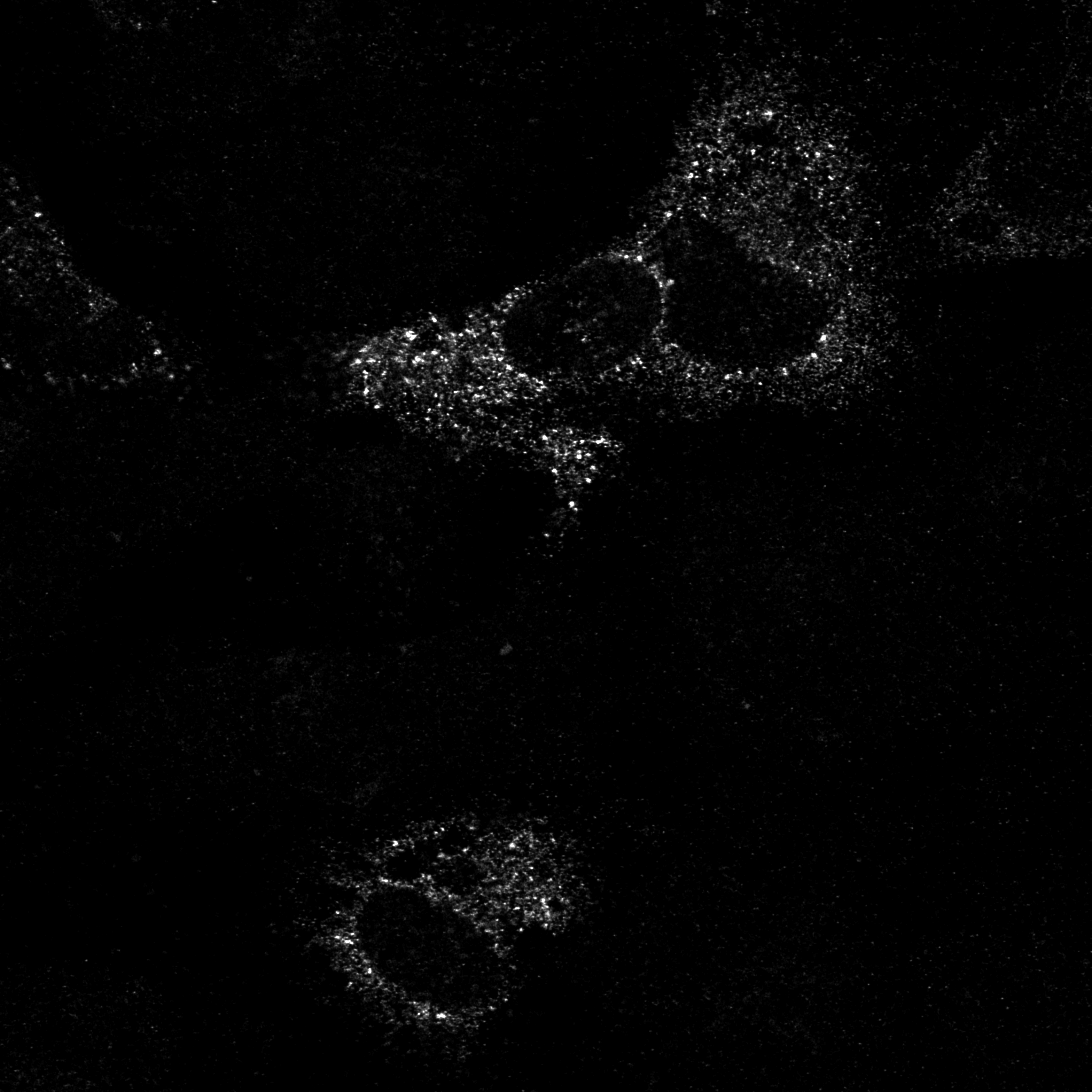

Supplement: Supplementary file 14 — Figure EV1 Source Data [file 44319_2026_773_MOESM14_ESM.zip › Figure EV1/Figure EV 1A/IF GR55KO UT RUSH PSAP-SBP.tif]

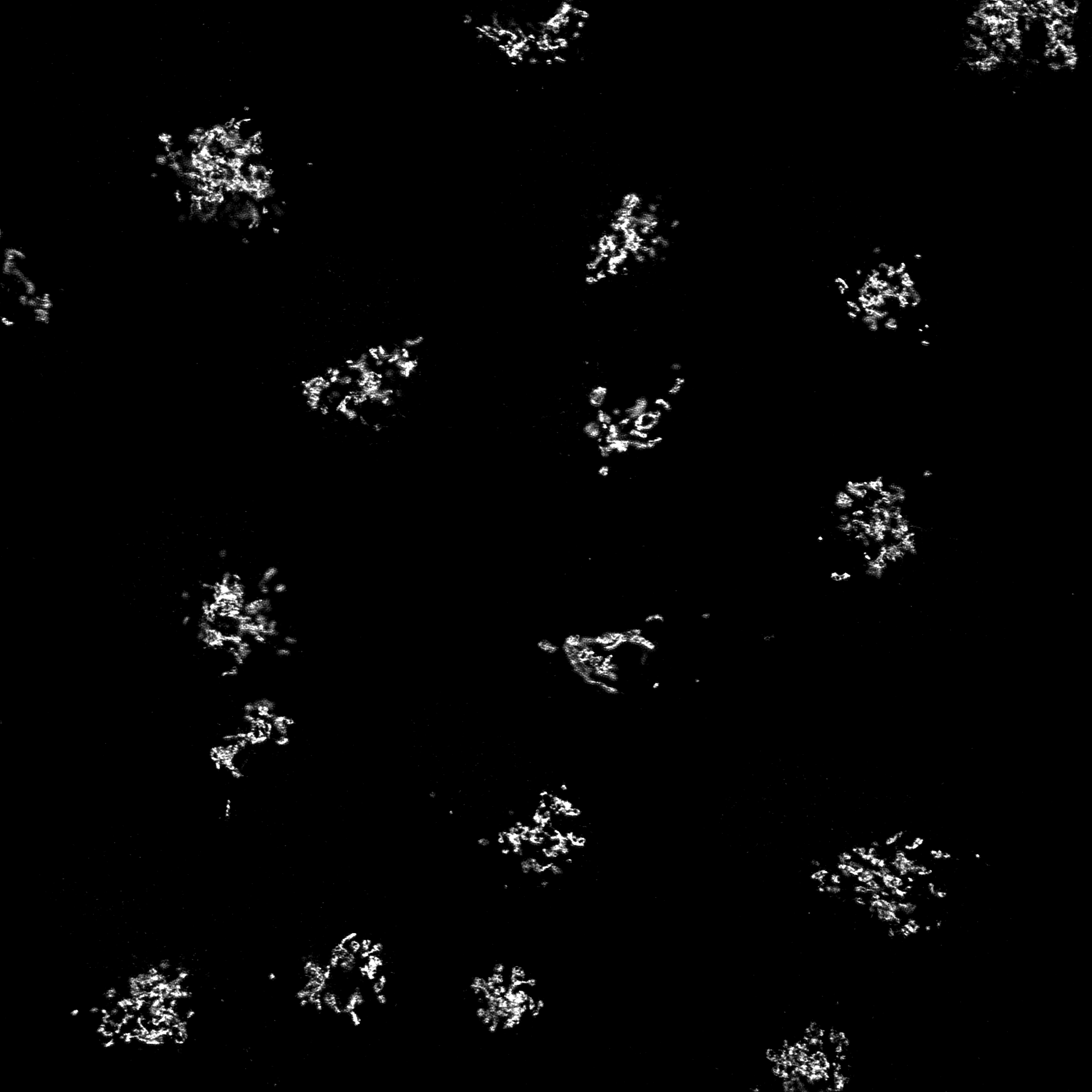

Supplement: Supplementary file 14 — Figure EV1 Source Data [file 44319_2026_773_MOESM14_ESM.zip › Figure EV1/Figure EV 1A/IF GR55KO 2h RUSH GM130.tif]

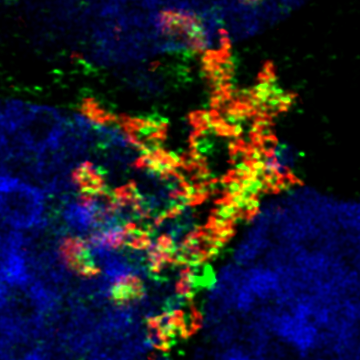

Supplement: Supplementary file 14 — Figure EV1 Source Data [file 44319_2026_773_MOESM14_ESM.zip › Figure EV1/Figure EV 1A/IF GR55KO 1h RUSH PSAP-SBP_GM130 MERGE inset.tif]

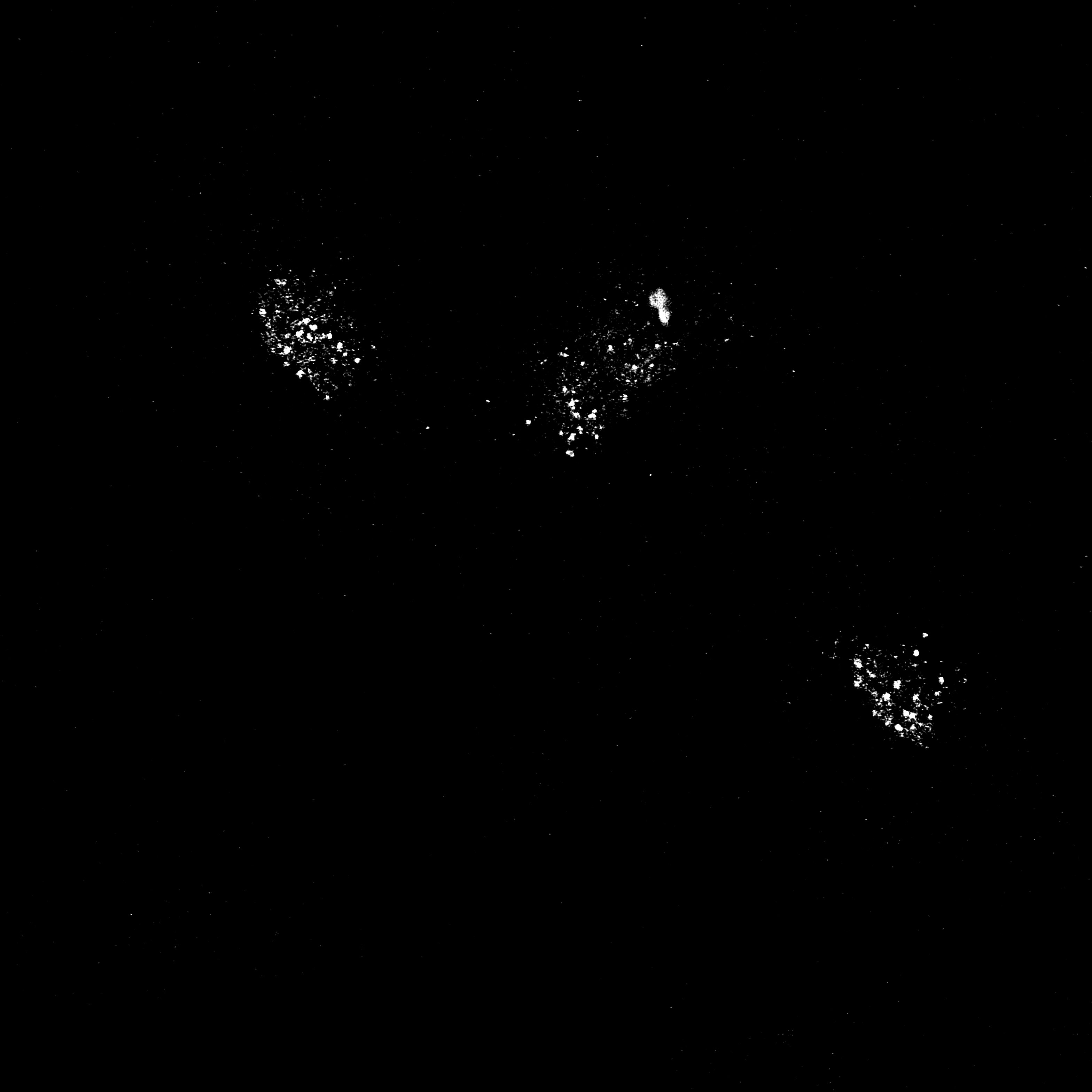

Supplement: Supplementary file 14 — Figure EV1 Source Data [file 44319_2026_773_MOESM14_ESM.zip › Figure EV1/Figure EV 1A/IF WT 4h RUSH PSAP-SBP.tif]

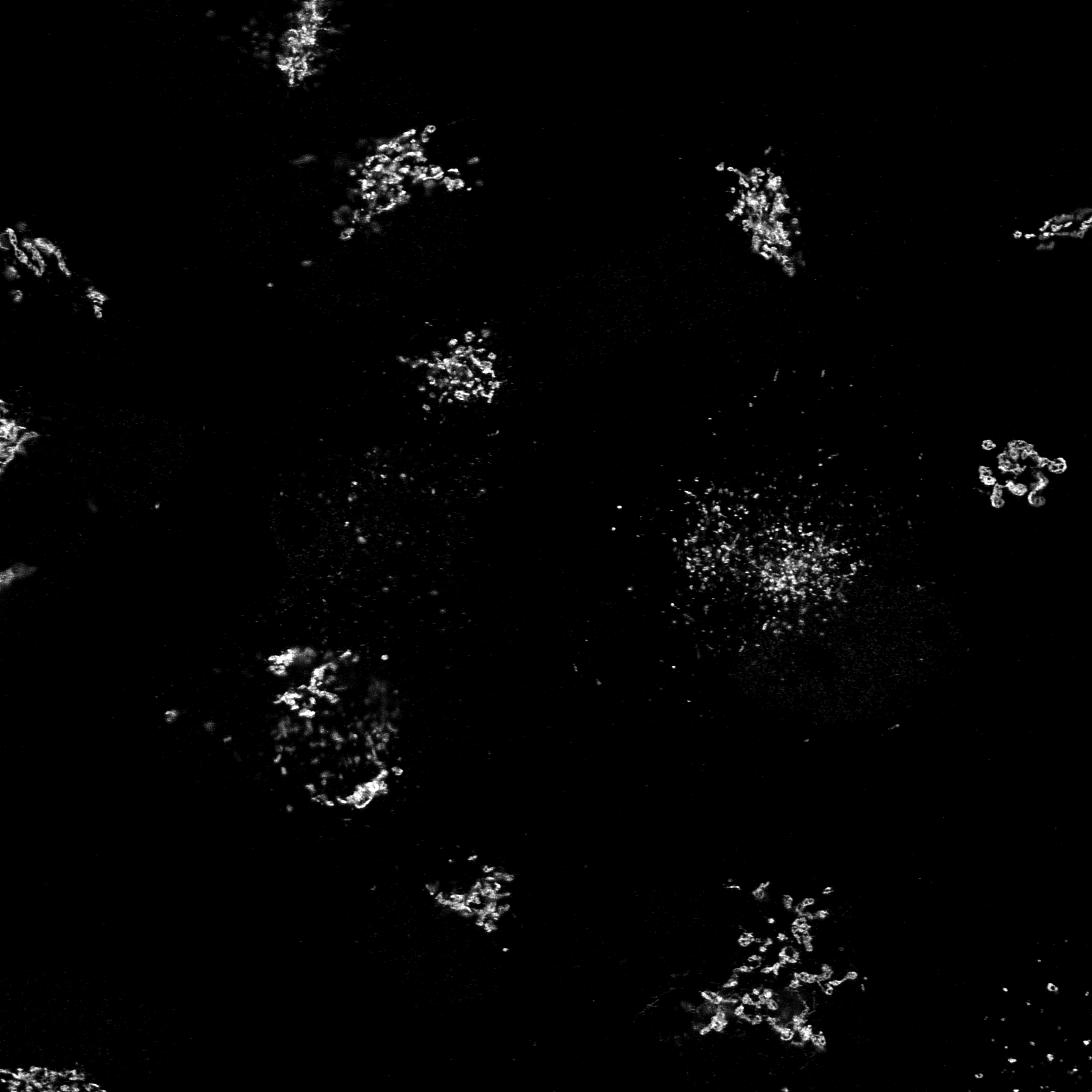

Supplement: Supplementary file 14 — Figure EV1 Source Data [file 44319_2026_773_MOESM14_ESM.zip › Figure EV1/Figure EV 1A/IF GR55KO UT RUSH GM130.tif]

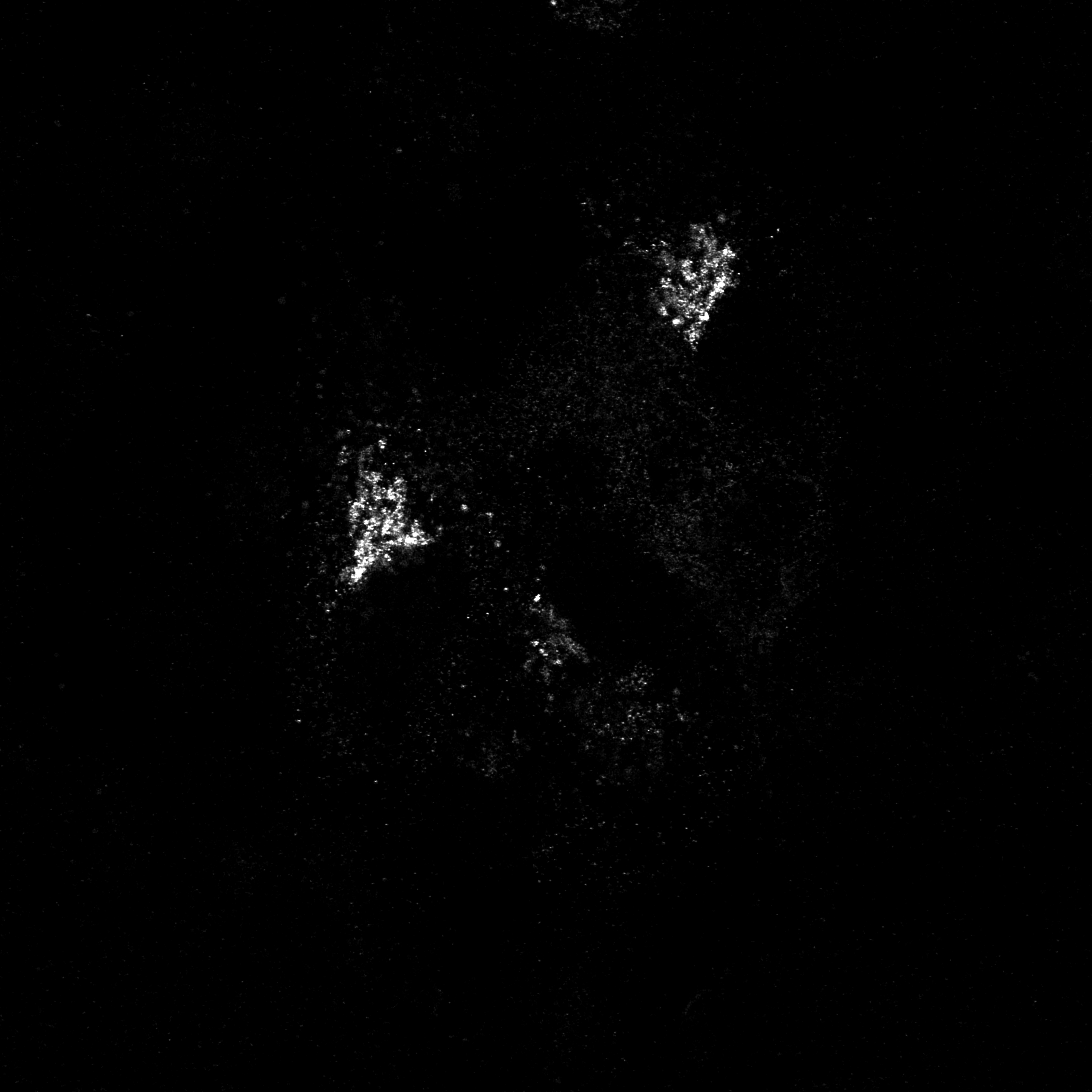

Supplement: Supplementary file 14 — Figure EV1 Source Data [file 44319_2026_773_MOESM14_ESM.zip › Figure EV1/Figure EV 1A/IF GR55KO 1h RUSH PSAP-SBP.tif]

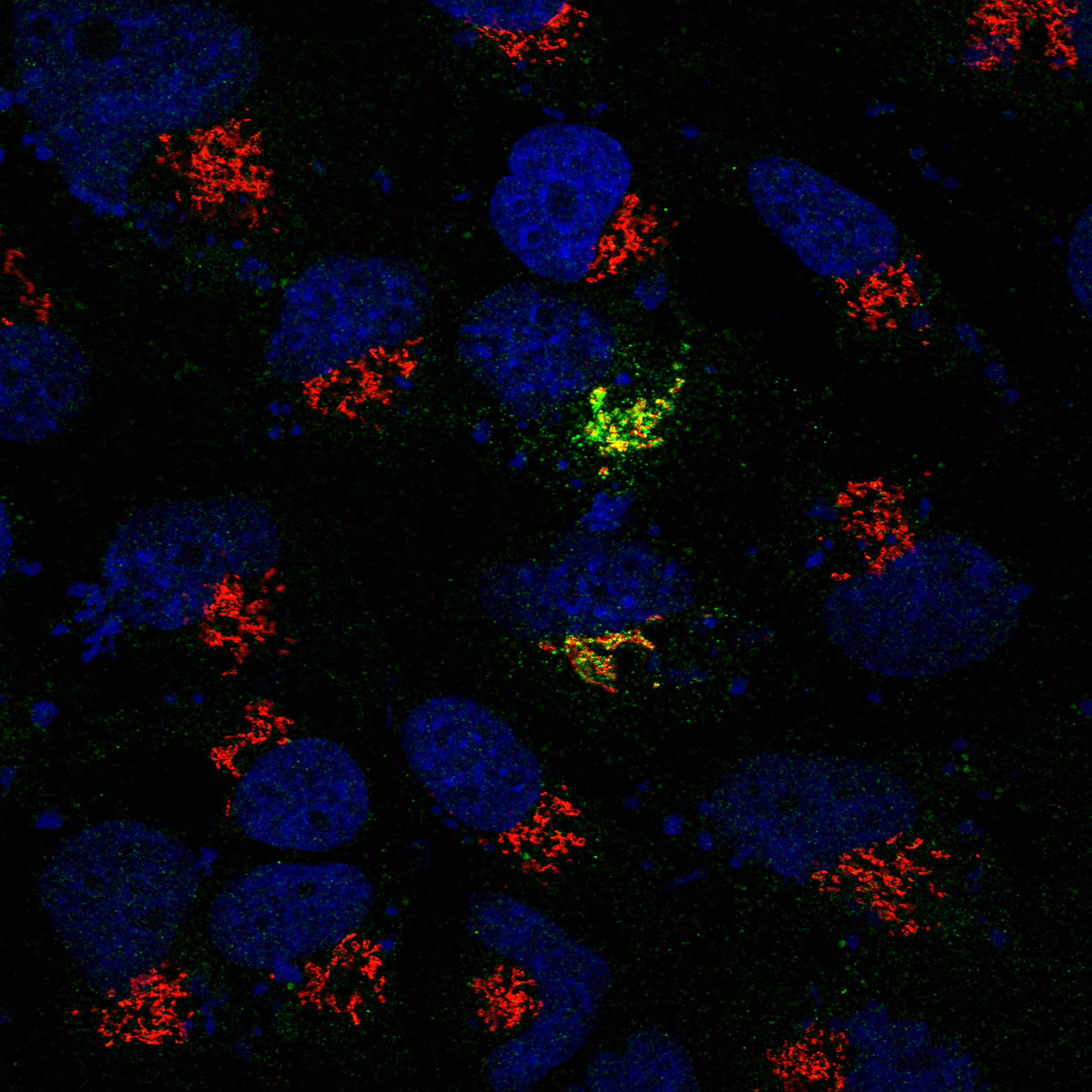

Supplement: Supplementary file 14 — Figure EV1 Source Data [file 44319_2026_773_MOESM14_ESM.zip › Figure EV1/Figure EV 1A/IF GR55KO 2h RUSH PSAP-SBP_GM130 MERGE.tif]

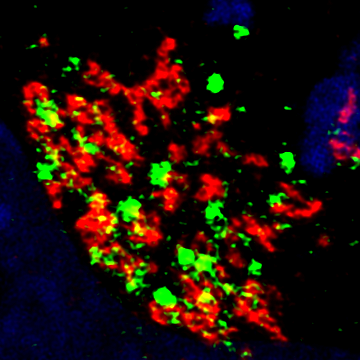

Supplement: Supplementary file 14 — Figure EV1 Source Data [file 44319_2026_773_MOESM14_ESM.zip › Figure EV1/Figure EV 1A/IF WT 4h RUSH PSAP-SBP_GM130 MERGE inset.tif]

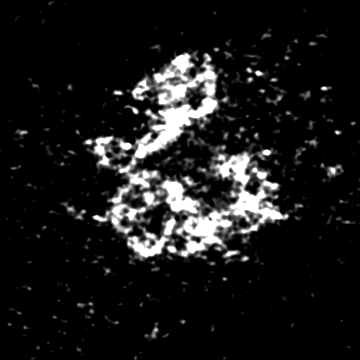

Supplement: Supplementary file 14 — Figure EV1 Source Data [file 44319_2026_773_MOESM14_ESM.zip › Figure EV1/Figure EV 1A/IF GR55KO 4h RUSH PSAP-SBP inset.tif]

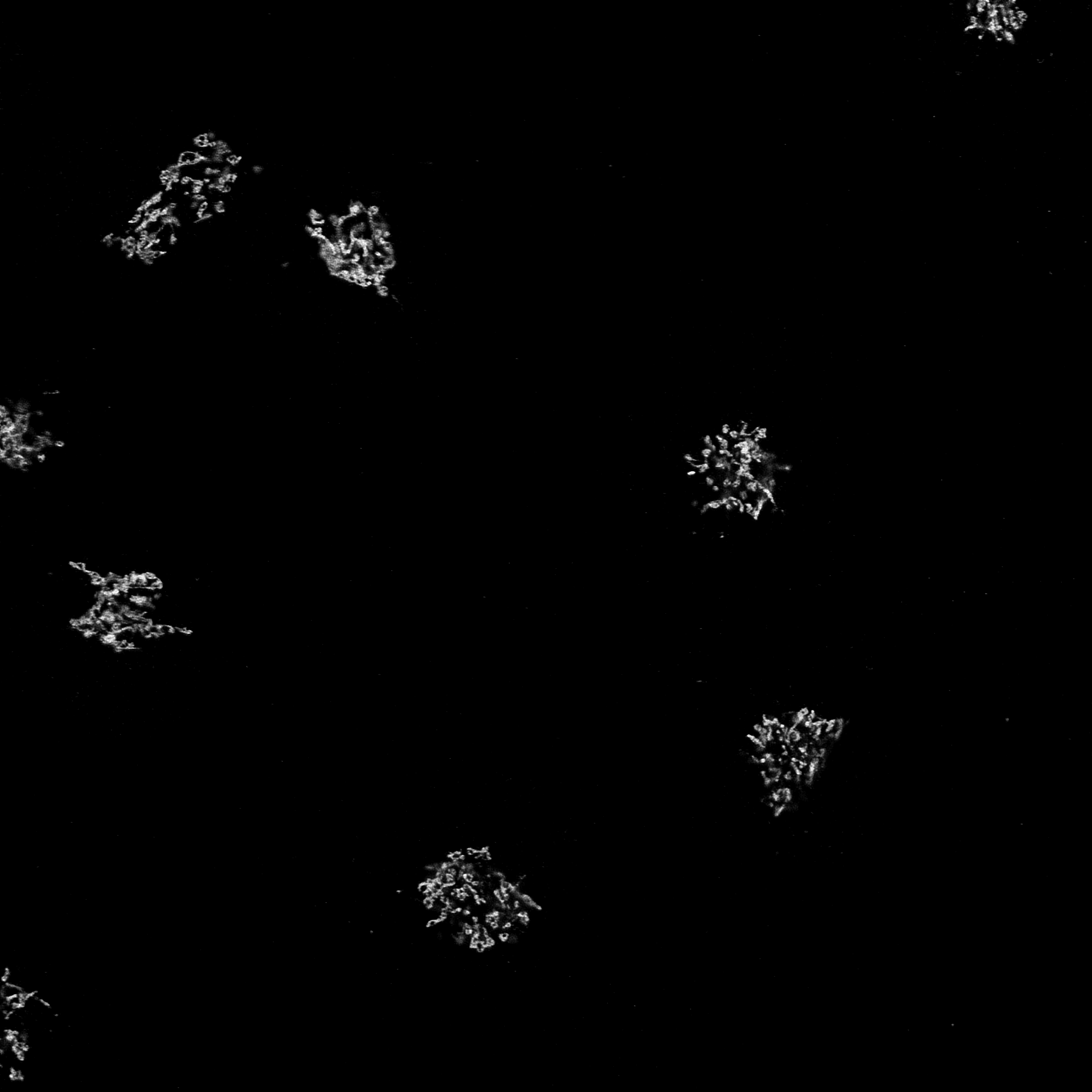

Supplement: Supplementary file 14 — Figure EV1 Source Data [file 44319_2026_773_MOESM14_ESM.zip › Figure EV1/Figure EV 1A/IF WT UT RUSH GM130.tif]

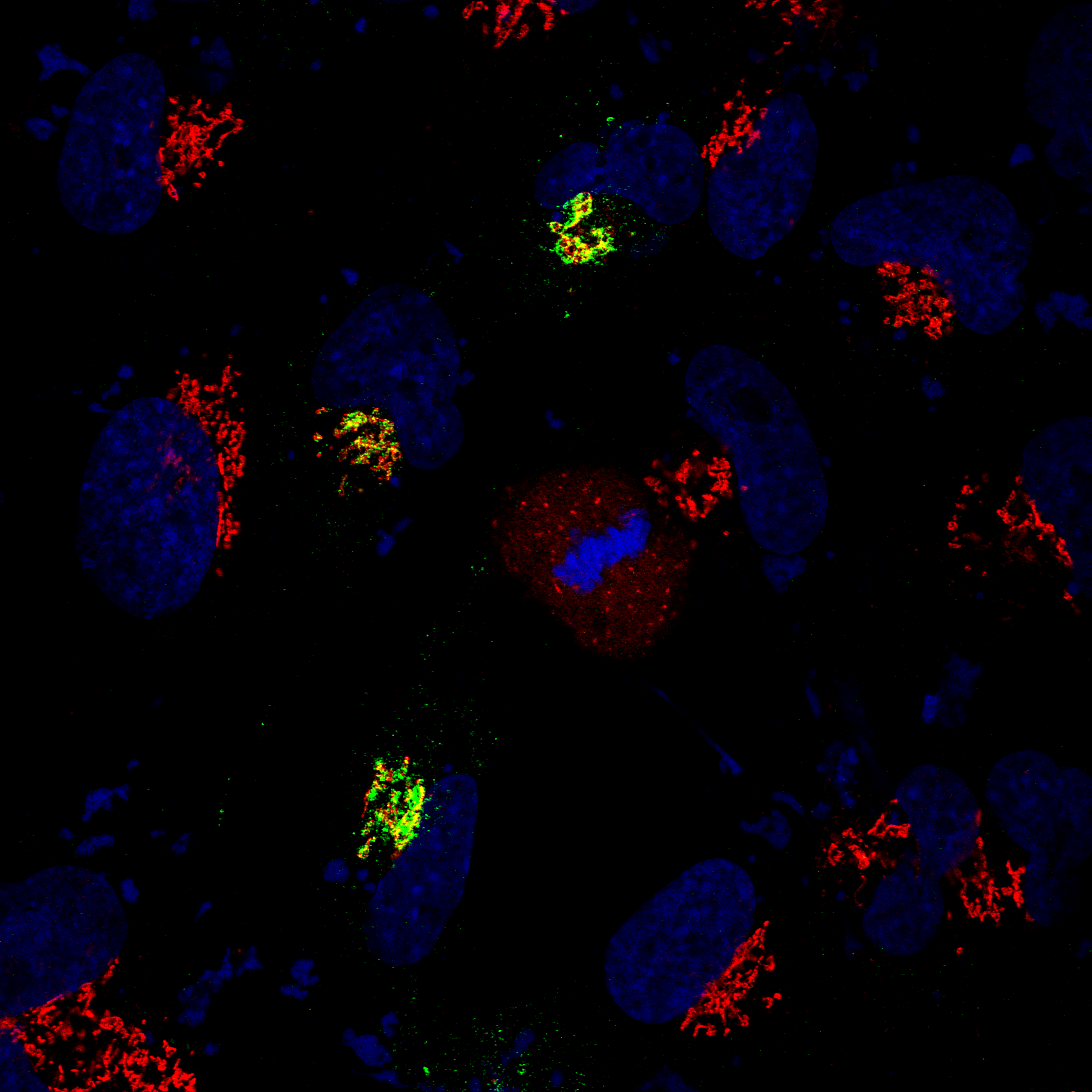

Supplement: Supplementary file 14 — Figure EV1 Source Data [file 44319_2026_773_MOESM14_ESM.zip › Figure EV1/Figure EV 1A/IF GR55KO 4h RUSH PSAP-SBP_GM130 MERGE.tif]

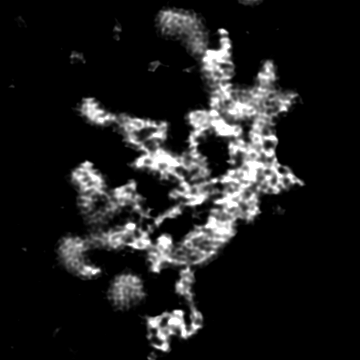

Supplement: Supplementary file 14 — Figure EV1 Source Data [file 44319_2026_773_MOESM14_ESM.zip › Figure EV1/Figure EV 1A/IF GR55KO 1h RUSH GM130 inset.tif]

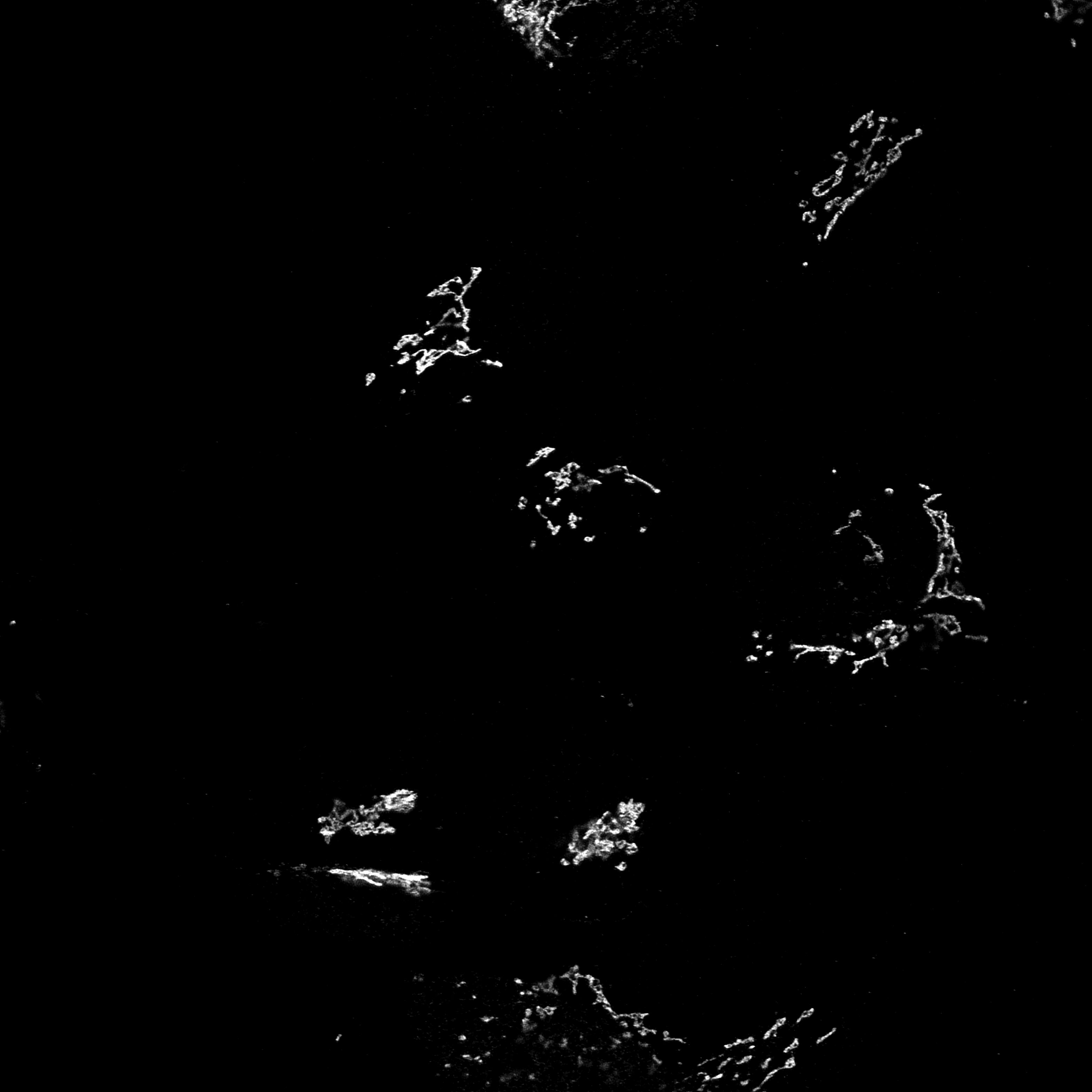

Supplement: Supplementary file 14 — Figure EV1 Source Data [file 44319_2026_773_MOESM14_ESM.zip › Figure EV1/Figure EV 1A/IF WT 1h RUSH GM130..tif]

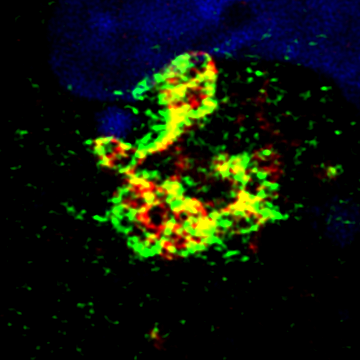

Supplement: Supplementary file 14 — Figure EV1 Source Data [file 44319_2026_773_MOESM14_ESM.zip › Figure EV1/Figure EV 1A/IF GR55KO 4h RUSH PSAP-SBP_GM130 MERGE inset.tif]

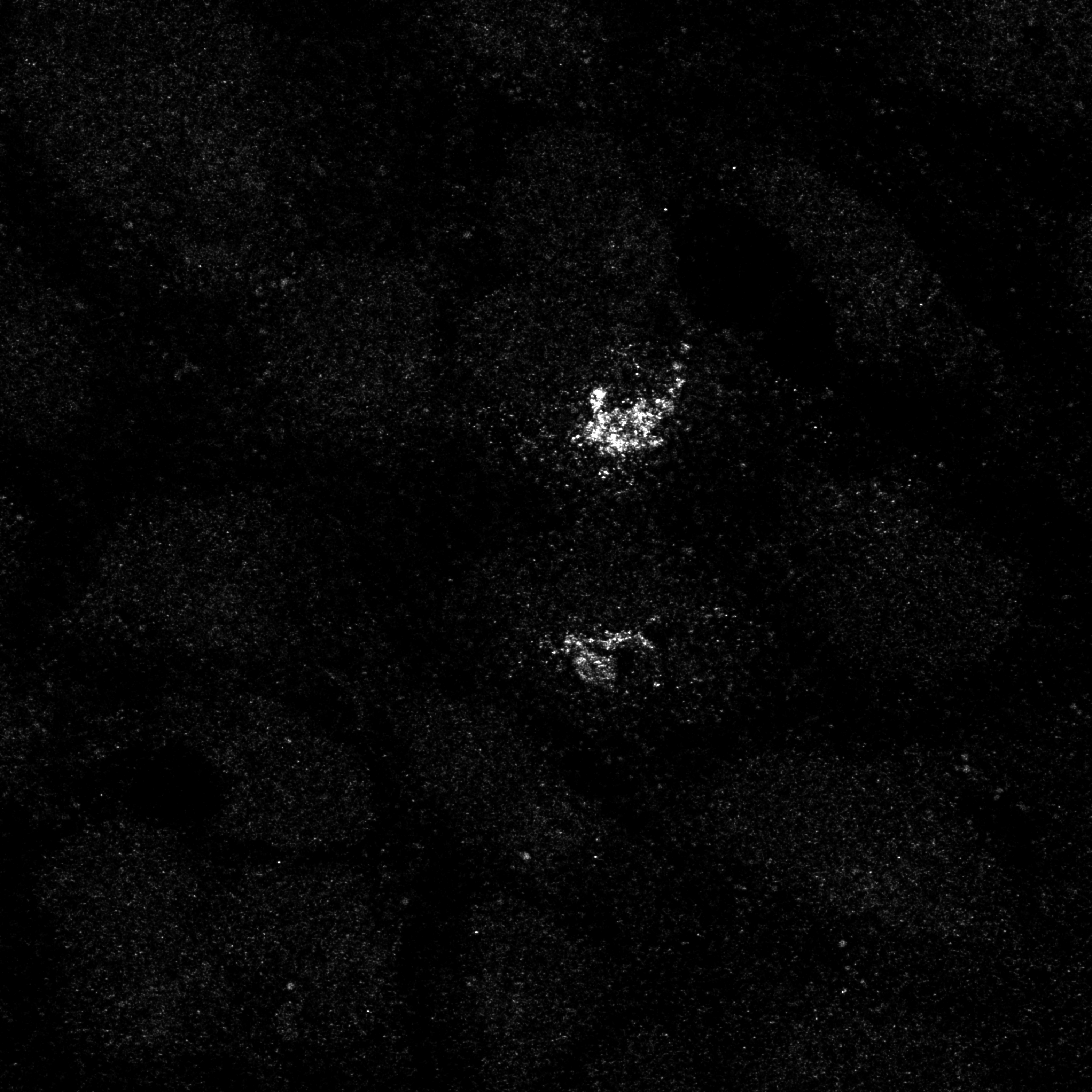

Supplement: Supplementary file 14 — Figure EV1 Source Data [file 44319_2026_773_MOESM14_ESM.zip › Figure EV1/Figure EV 1A/IF GR55KO 2h RUSH PSAP-SBP.tif]

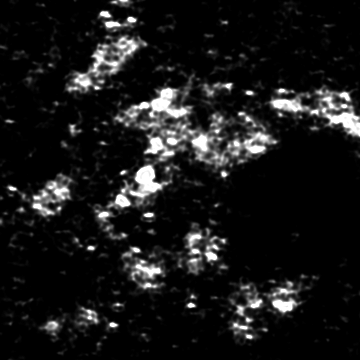

Supplement: Supplementary file 14 — Figure EV1 Source Data [file 44319_2026_773_MOESM14_ESM.zip › Figure EV1/Figure EV 1A/IF WT 1h RUSH PSAP-SBP inset.tif]

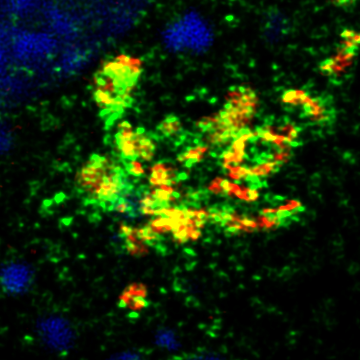

Supplement: Supplementary file 14 — Figure EV1 Source Data [file 44319_2026_773_MOESM14_ESM.zip › Figure EV1/Figure EV 1A/IF GR55KO 2h RUSH PSAP-SBP_GM130 MERGE inset.tif]

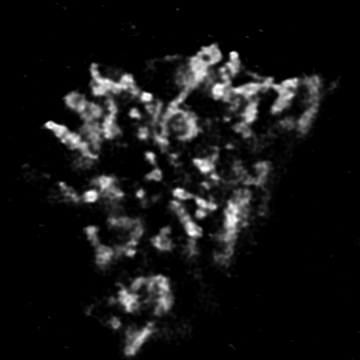

Supplement: Supplementary file 14 — Figure EV1 Source Data [file 44319_2026_773_MOESM14_ESM.zip › Figure EV1/Figure EV 1A/IF WT UT RUSH GM130 inset.tif]

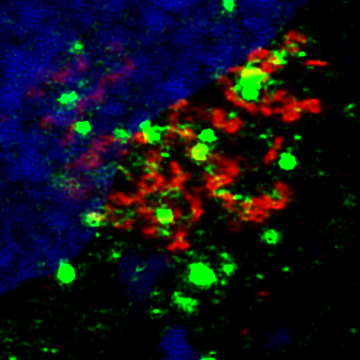

Supplement: Supplementary file 14 — Figure EV1 Source Data [file 44319_2026_773_MOESM14_ESM.zip › Figure EV1/Figure EV 1A/IF WT 2h RUSH PSAP-SBP_GM130 MERGE inset.tif]

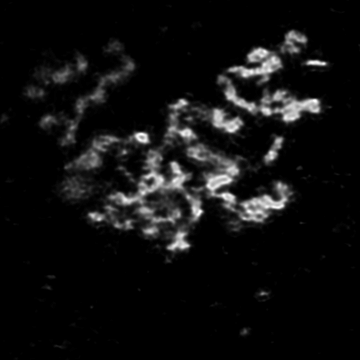

Supplement: Supplementary file 14 — Figure EV1 Source Data [file 44319_2026_773_MOESM14_ESM.zip › Figure EV1/Figure EV 1A/IF WT 2h RUSH GM130 inset.tif]

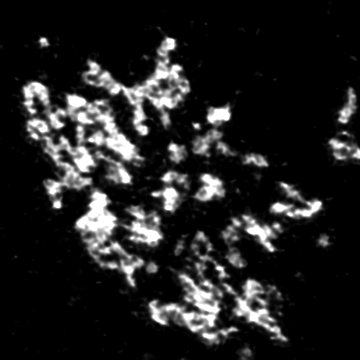

Supplement: Supplementary file 14 — Figure EV1 Source Data [file 44319_2026_773_MOESM14_ESM.zip › Figure EV1/Figure EV 1A/IF WT 4h RUSH GM130 inset.tif]

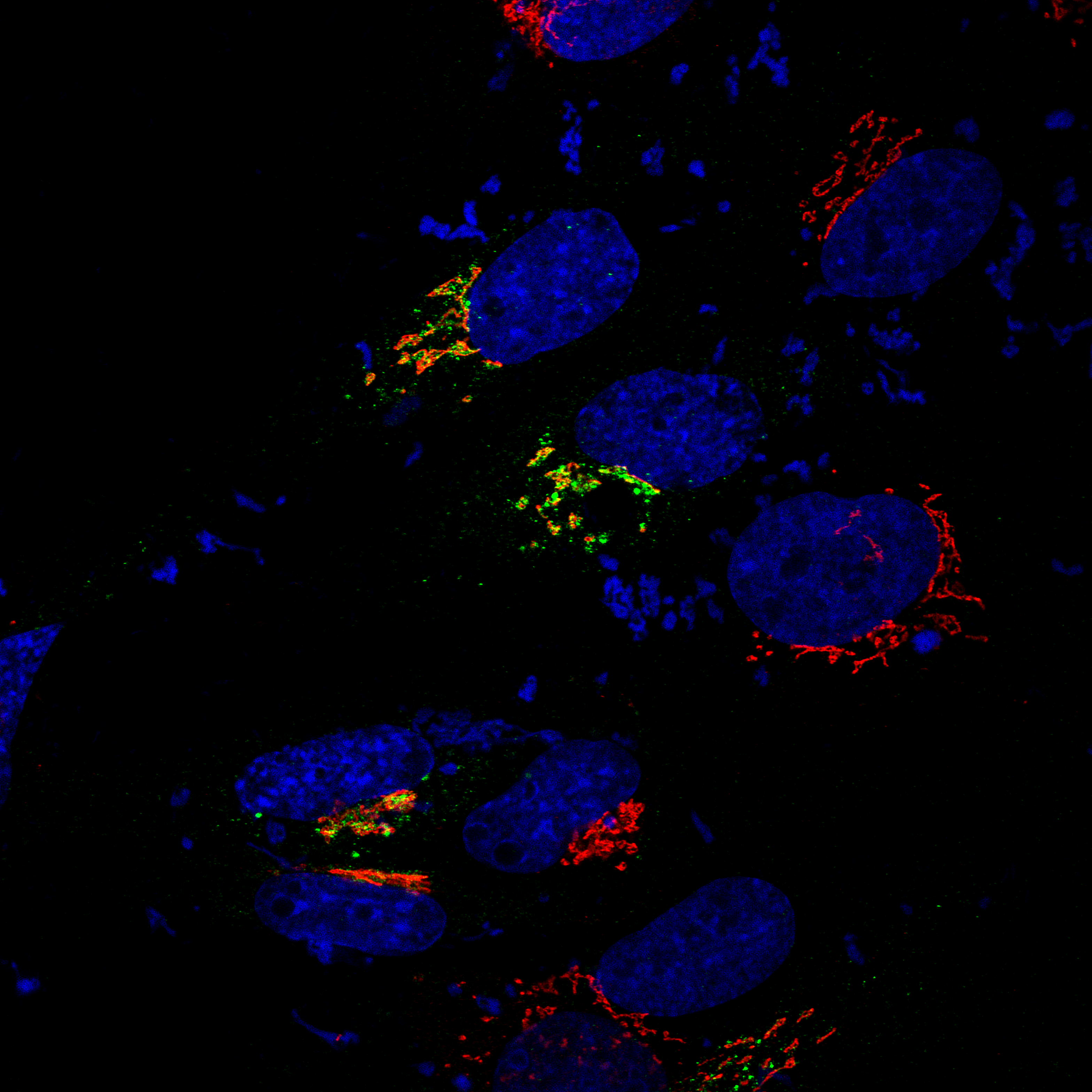

Supplement: Supplementary file 14 — Figure EV1 Source Data [file 44319_2026_773_MOESM14_ESM.zip › Figure EV1/Figure EV 1A/IF WT 1h RUSH PSAP-SBP_GM130 MERGE.tif]

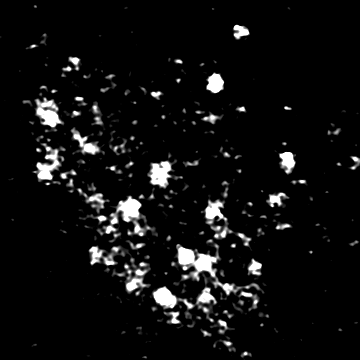

Supplement: Supplementary file 14 — Figure EV1 Source Data [file 44319_2026_773_MOESM14_ESM.zip › Figure EV1/Figure EV 1A/IF WT 4h RUSH PSAP-SBP inset.tif]

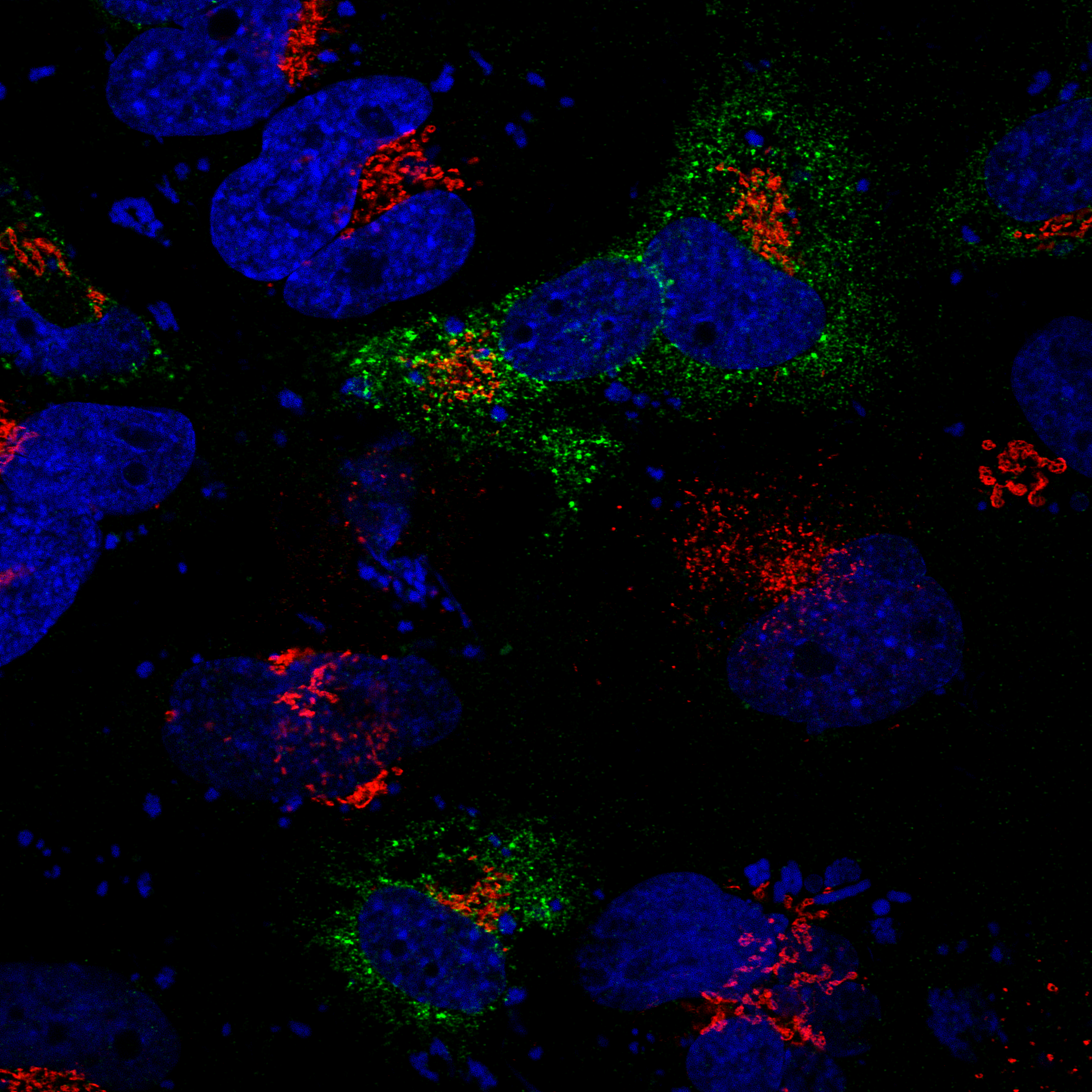

Supplement: Supplementary file 14 — Figure EV1 Source Data [file 44319_2026_773_MOESM14_ESM.zip › Figure EV1/Figure EV 1A/IF GR55KO UT RUSH PSAP-SBP_GM130 MERGE.tif]

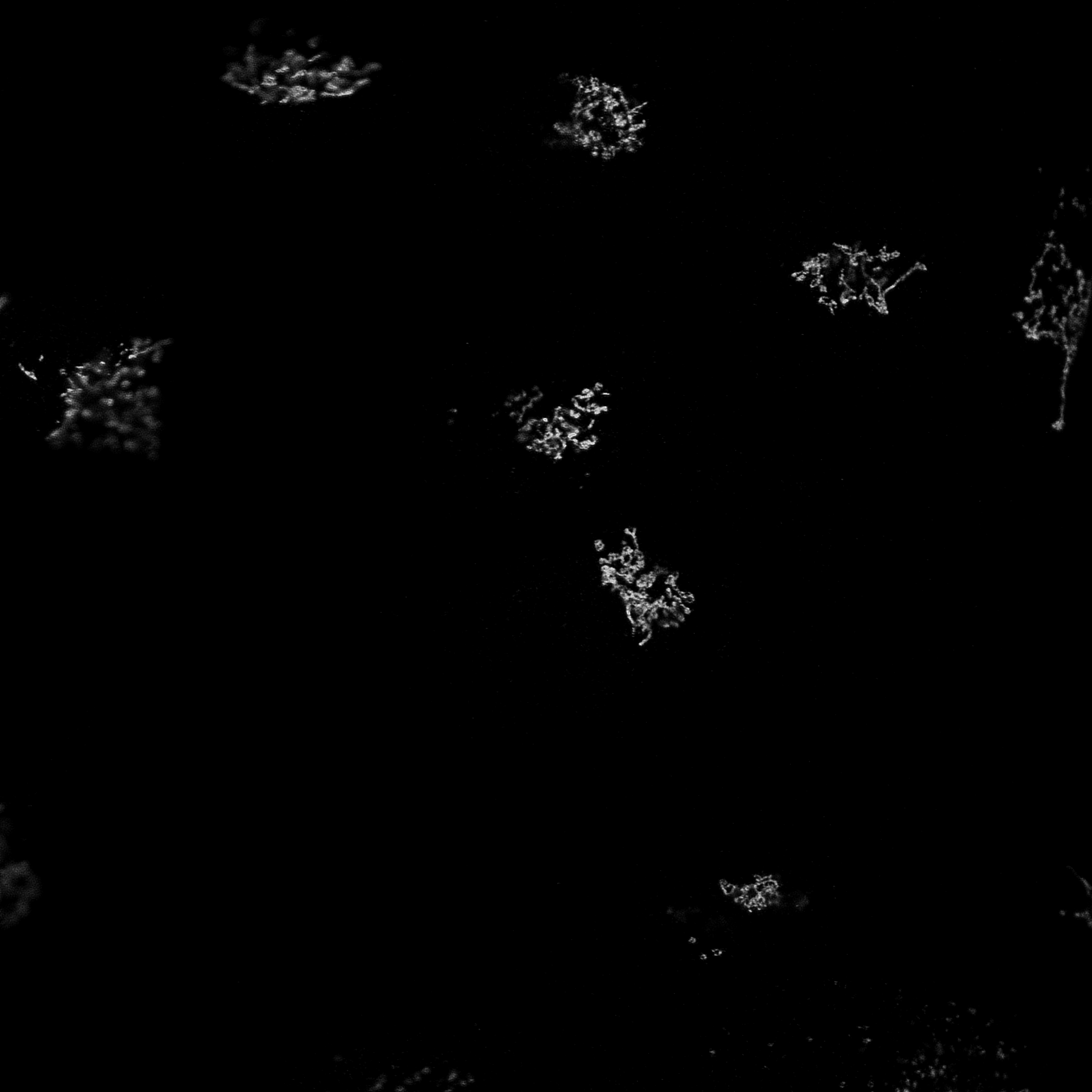

Supplement: Supplementary file 14 — Figure EV1 Source Data [file 44319_2026_773_MOESM14_ESM.zip › Figure EV1/Figure EV 1A/IF WT 2h RUSH GM130tif.tif]

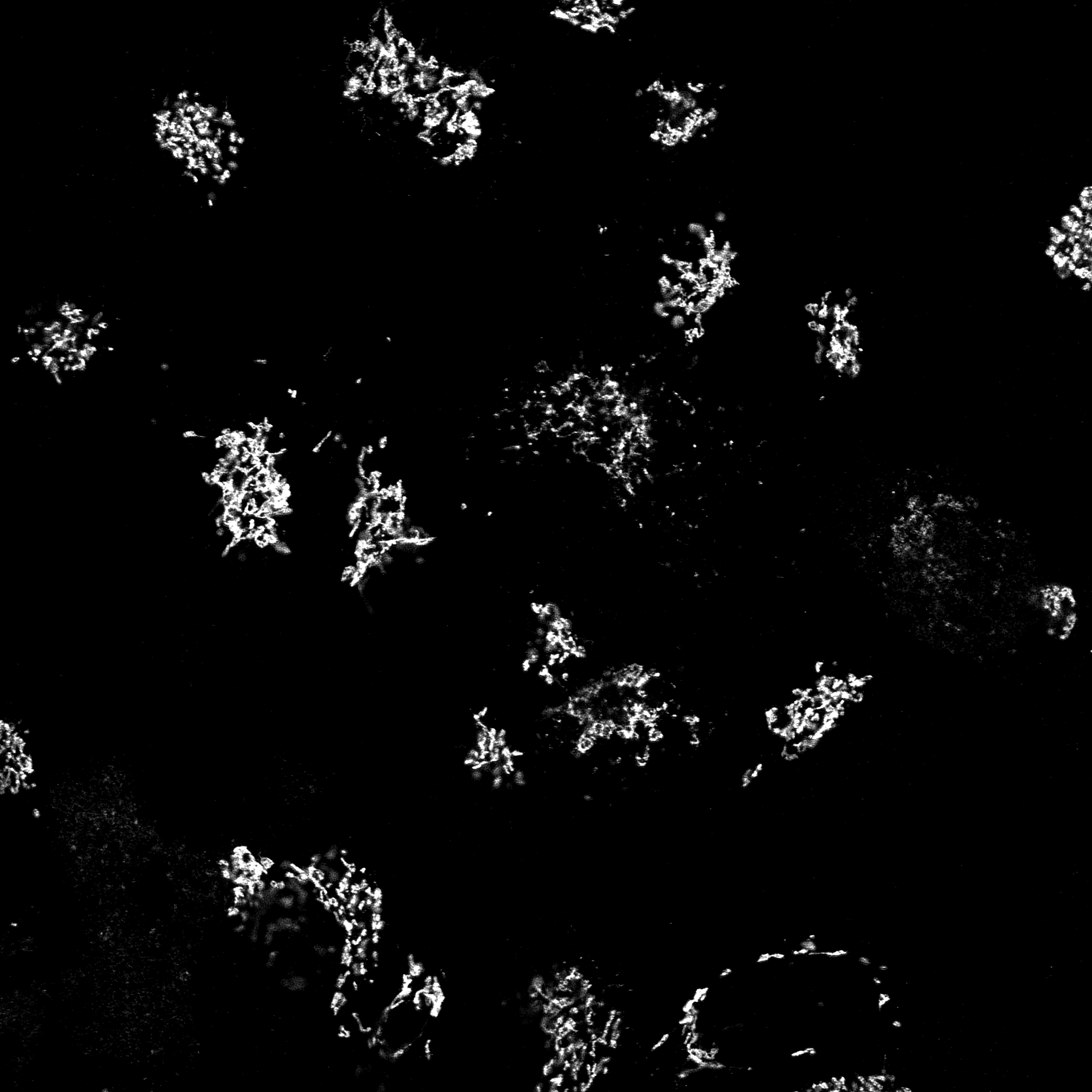

Supplement: Supplementary file 14 — Figure EV1 Source Data [file 44319_2026_773_MOESM14_ESM.zip › Figure EV1/Figure EV 1A/IF GR55KO 1h RUSH GM130.tif]

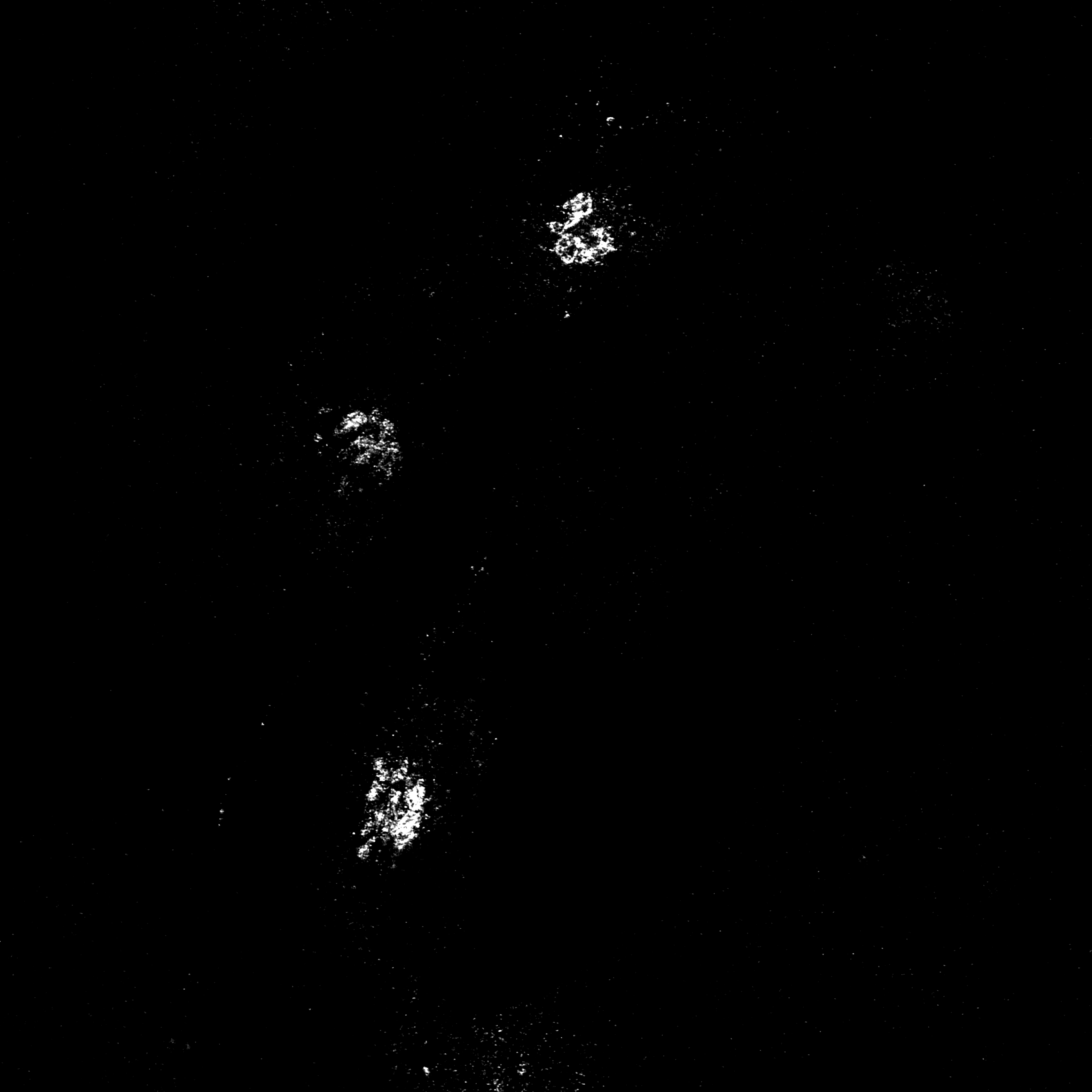

Supplement: Supplementary file 14 — Figure EV1 Source Data [file 44319_2026_773_MOESM14_ESM.zip › Figure EV1/Figure EV 1A/IF GR55KO 4h RUSH PSAP-SBP.tif]

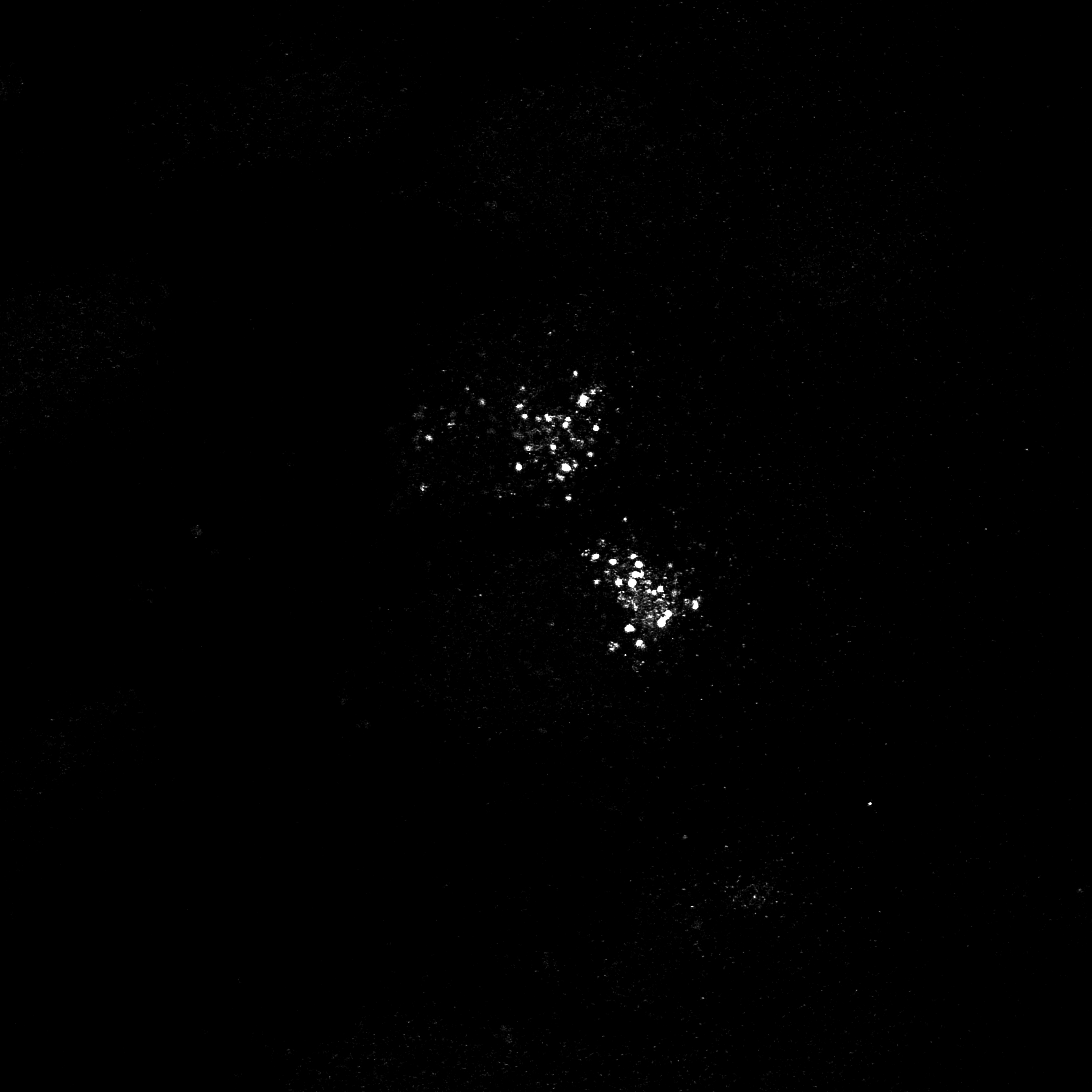

Supplement: Supplementary file 14 — Figure EV1 Source Data [file 44319_2026_773_MOESM14_ESM.zip › Figure EV1/Figure EV 1A/IF WT 2h RUSH PSAP-SBP.tif]

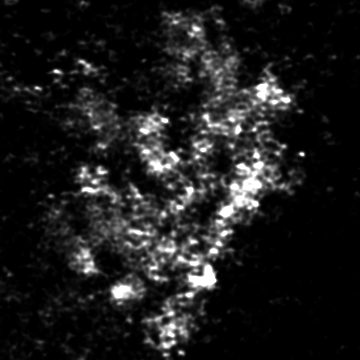

Supplement: Supplementary file 14 — Figure EV1 Source Data [file 44319_2026_773_MOESM14_ESM.zip › Figure EV1/Figure EV 1A/IF GR55KO 1h RUSH PSAP-SBP inset.tif]

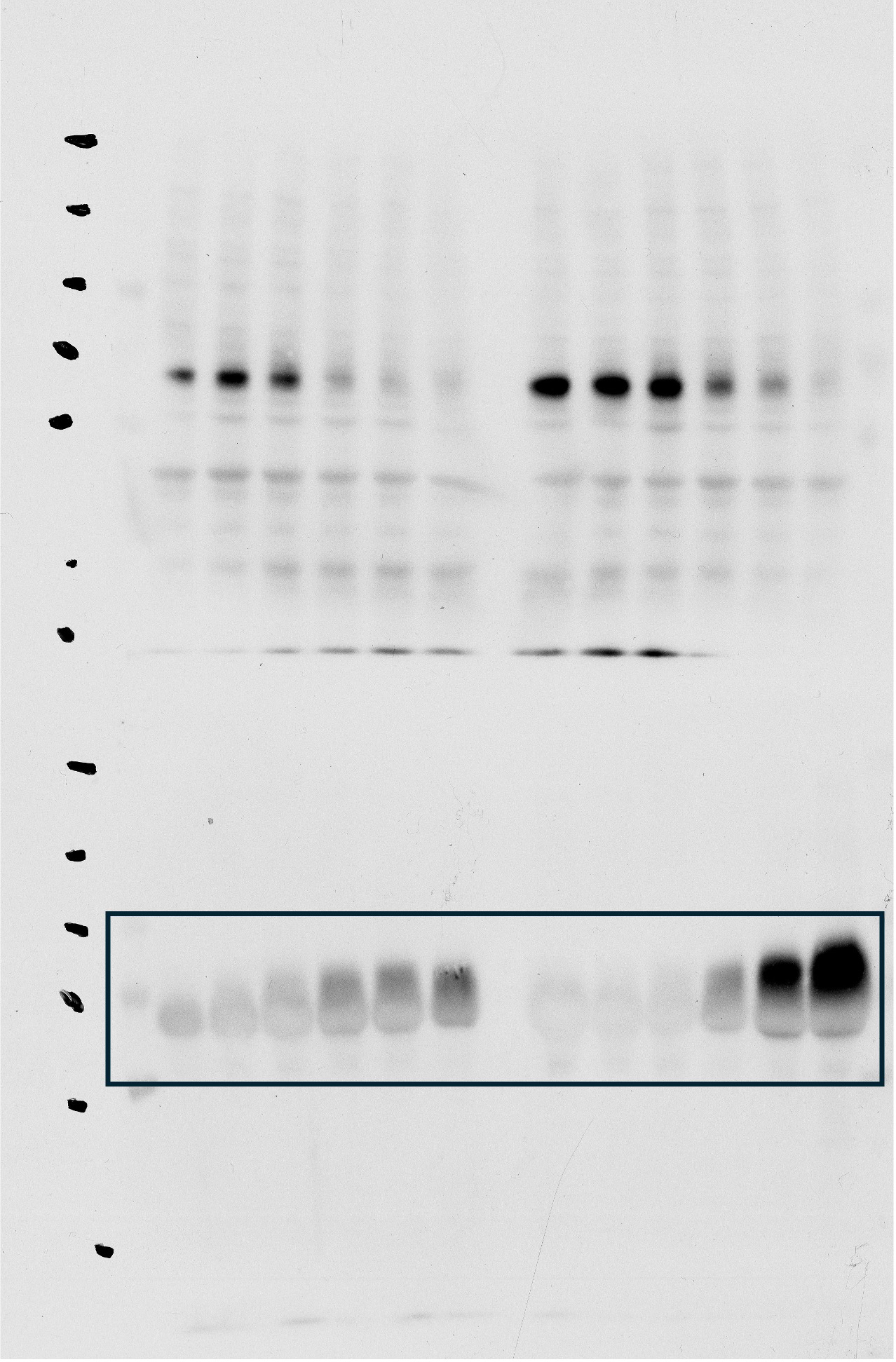

Supplement: Supplementary file 14 — Figure EV1 Source Data [file 44319_2026_773_MOESM14_ESM.zip › Figure EV1/Figure EV 1B/Western PSAP medium long exposure.tif]

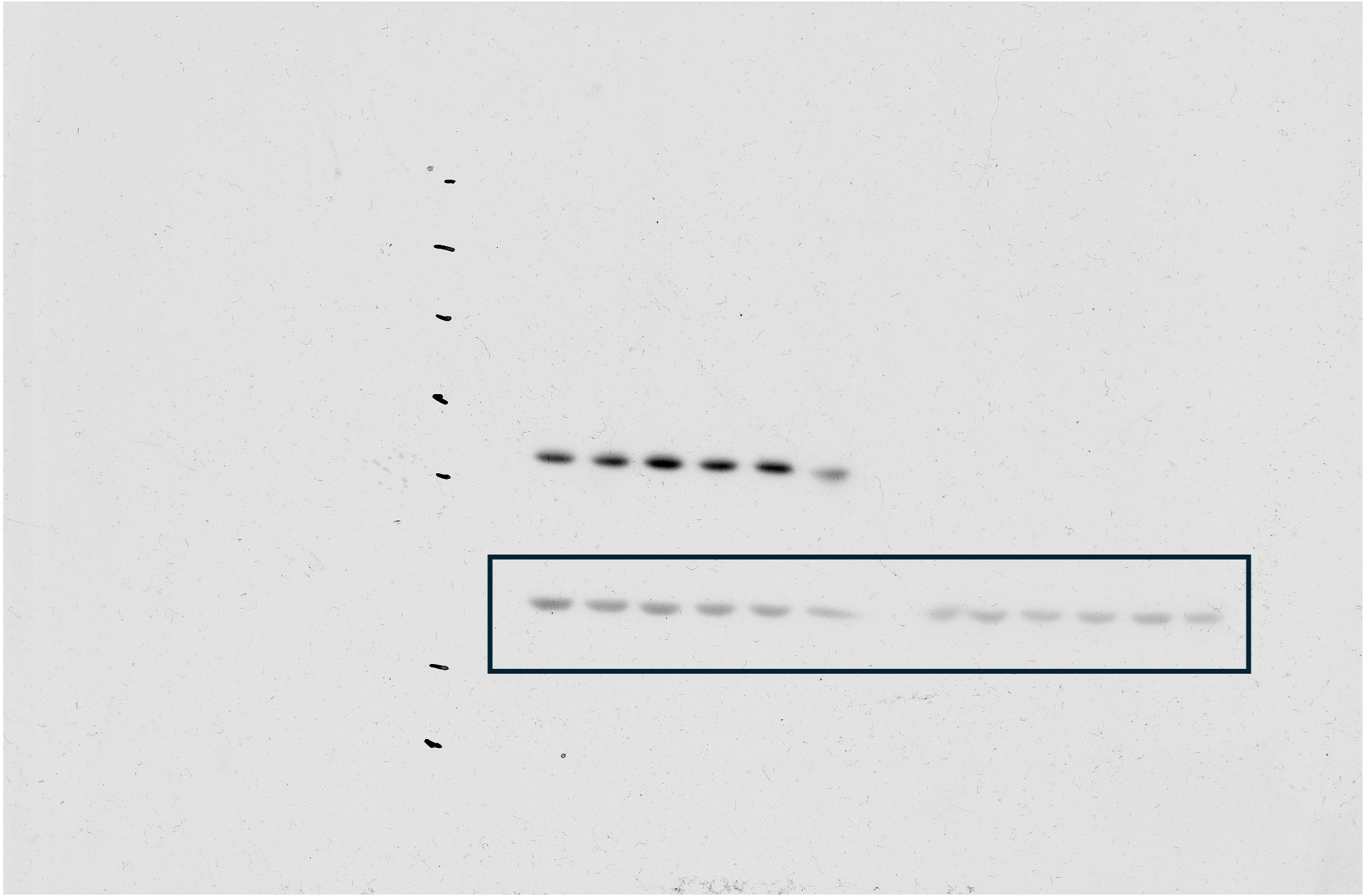

Supplement: Supplementary file 14 — Figure EV1 Source Data [file 44319_2026_773_MOESM14_ESM.zip › Figure EV1/Figure EV 1B/Western ACTIN.tif]

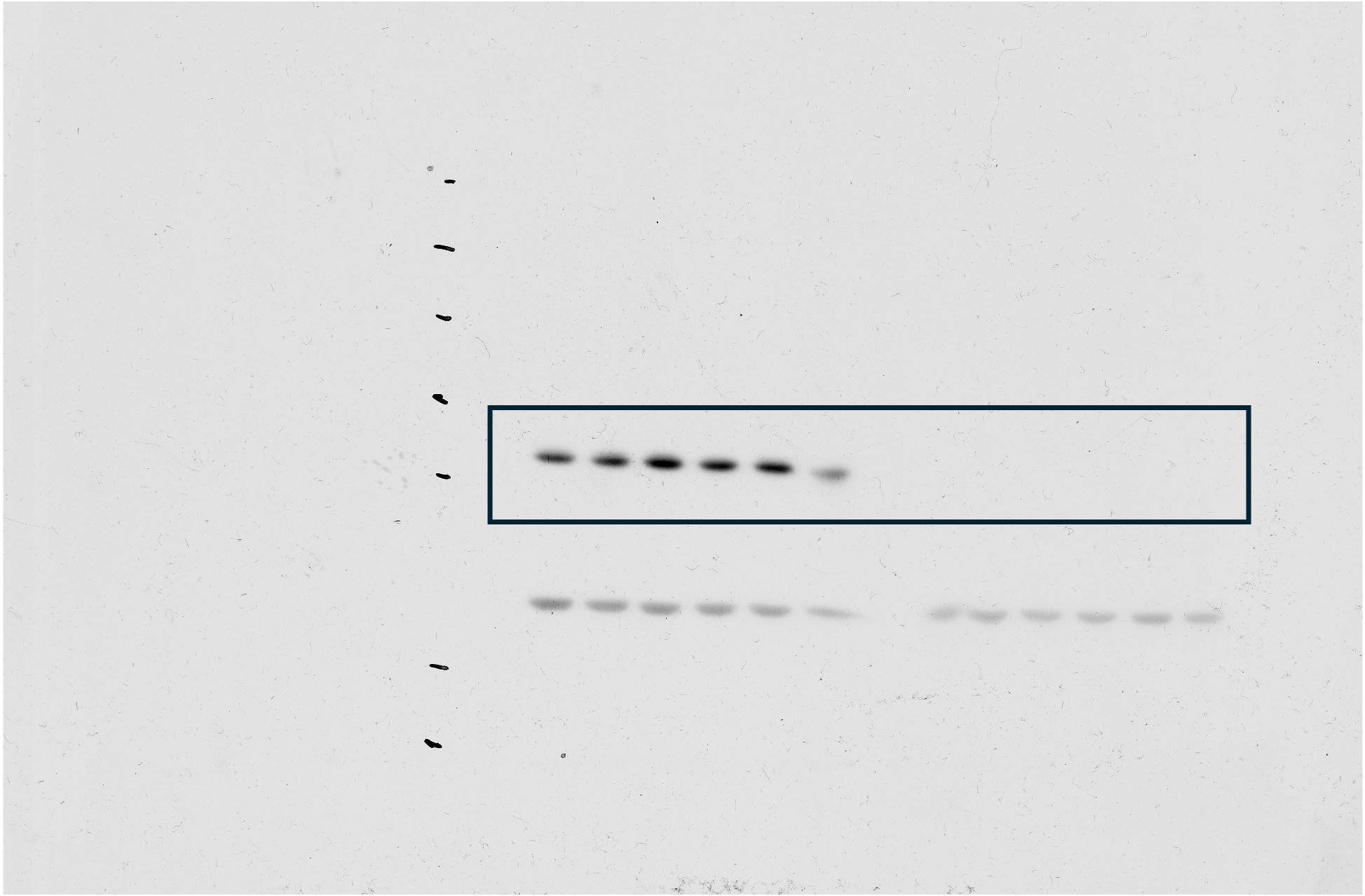

Supplement: Supplementary file 14 — Figure EV1 Source Data [file 44319_2026_773_MOESM14_ESM.zip › Figure EV1/Figure EV 1B/Western GRASP55.tif]

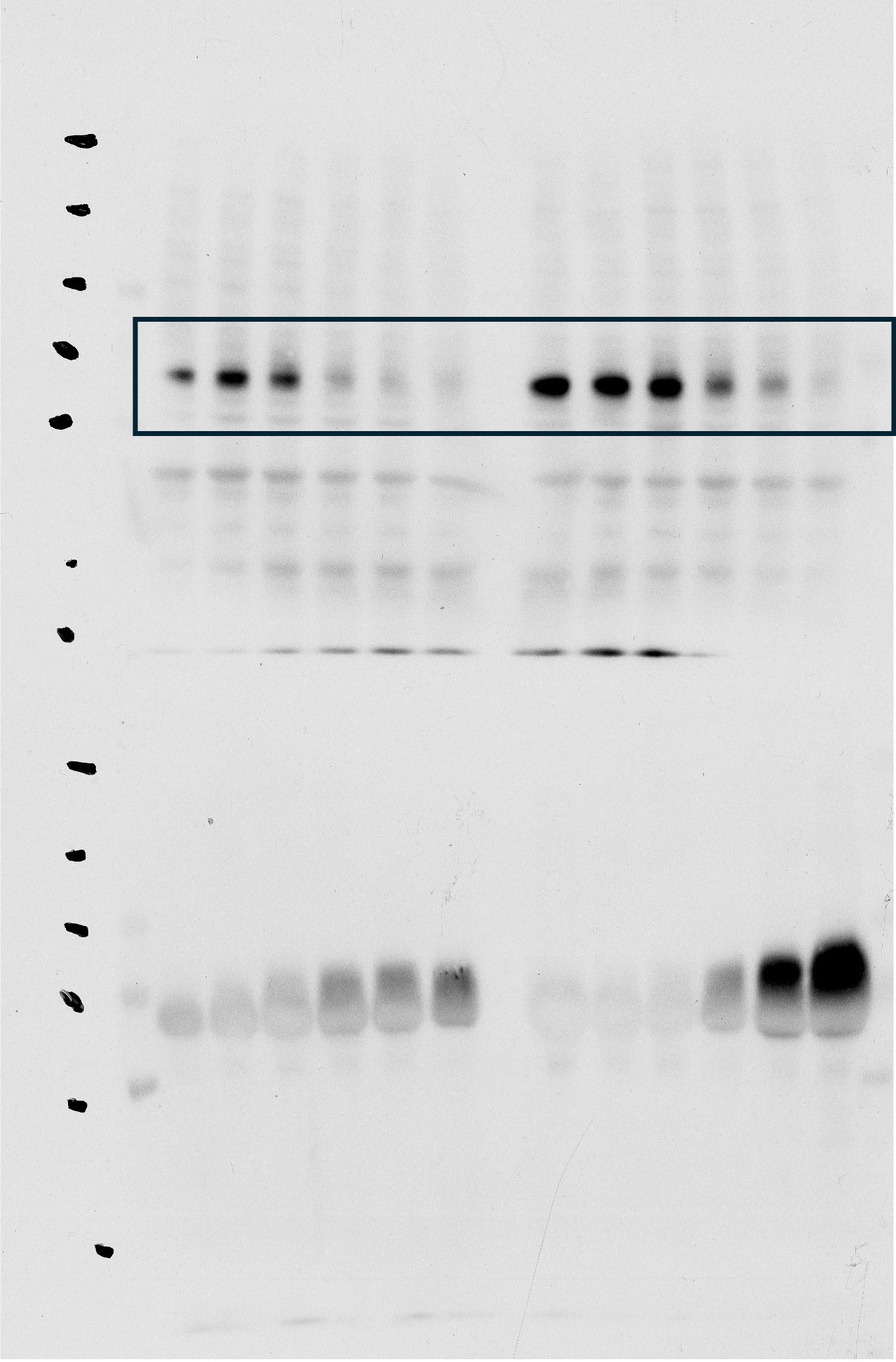

Supplement: Supplementary file 14 — Figure EV1 Source Data [file 44319_2026_773_MOESM14_ESM.zip › Figure EV1/Figure EV 1B/Western PSAP Lysate.tif]

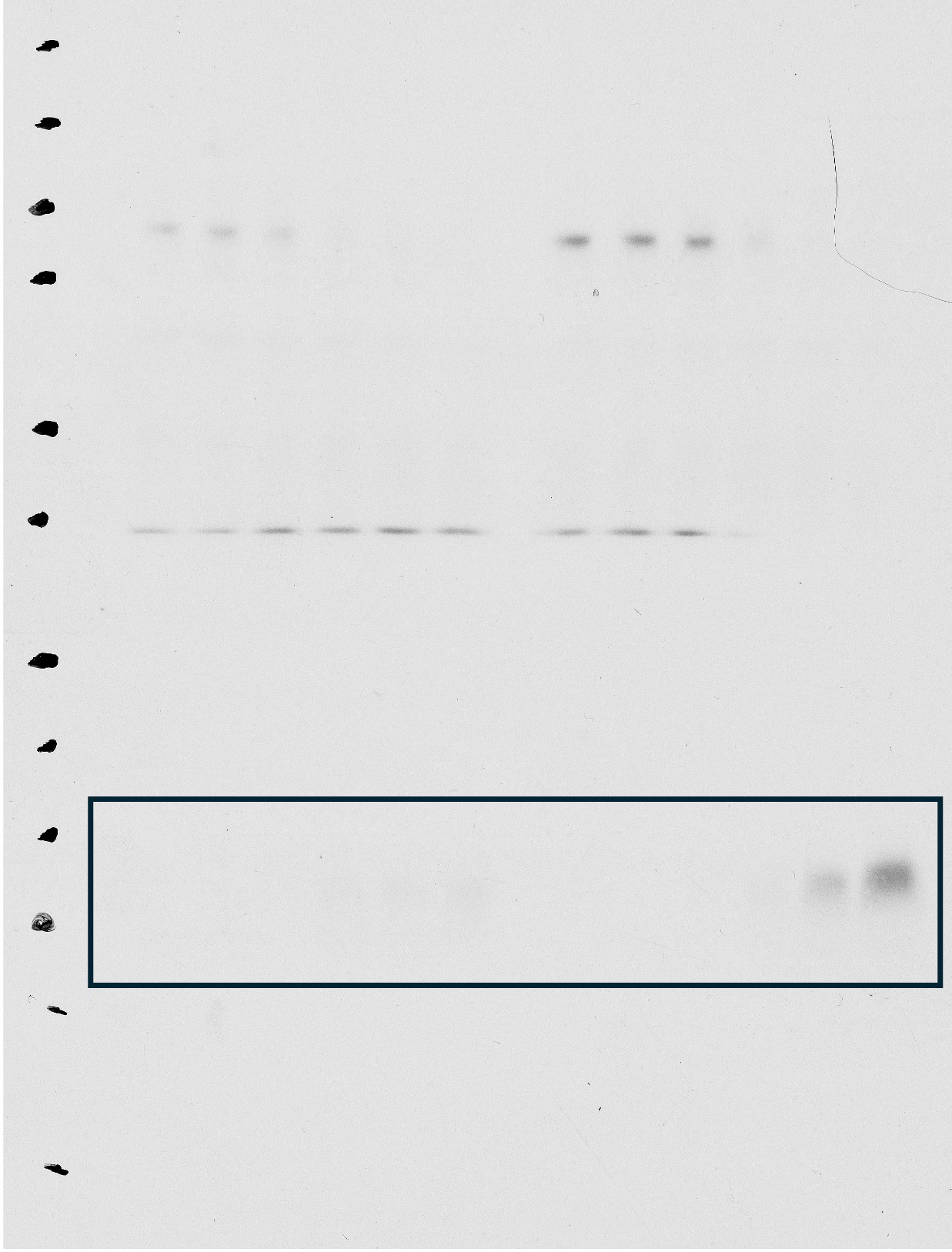

Supplement: Supplementary file 14 — Figure EV1 Source Data [file 44319_2026_773_MOESM14_ESM.zip › Figure EV1/Figure EV 1B/Western PSAP medium short exposure.tif]

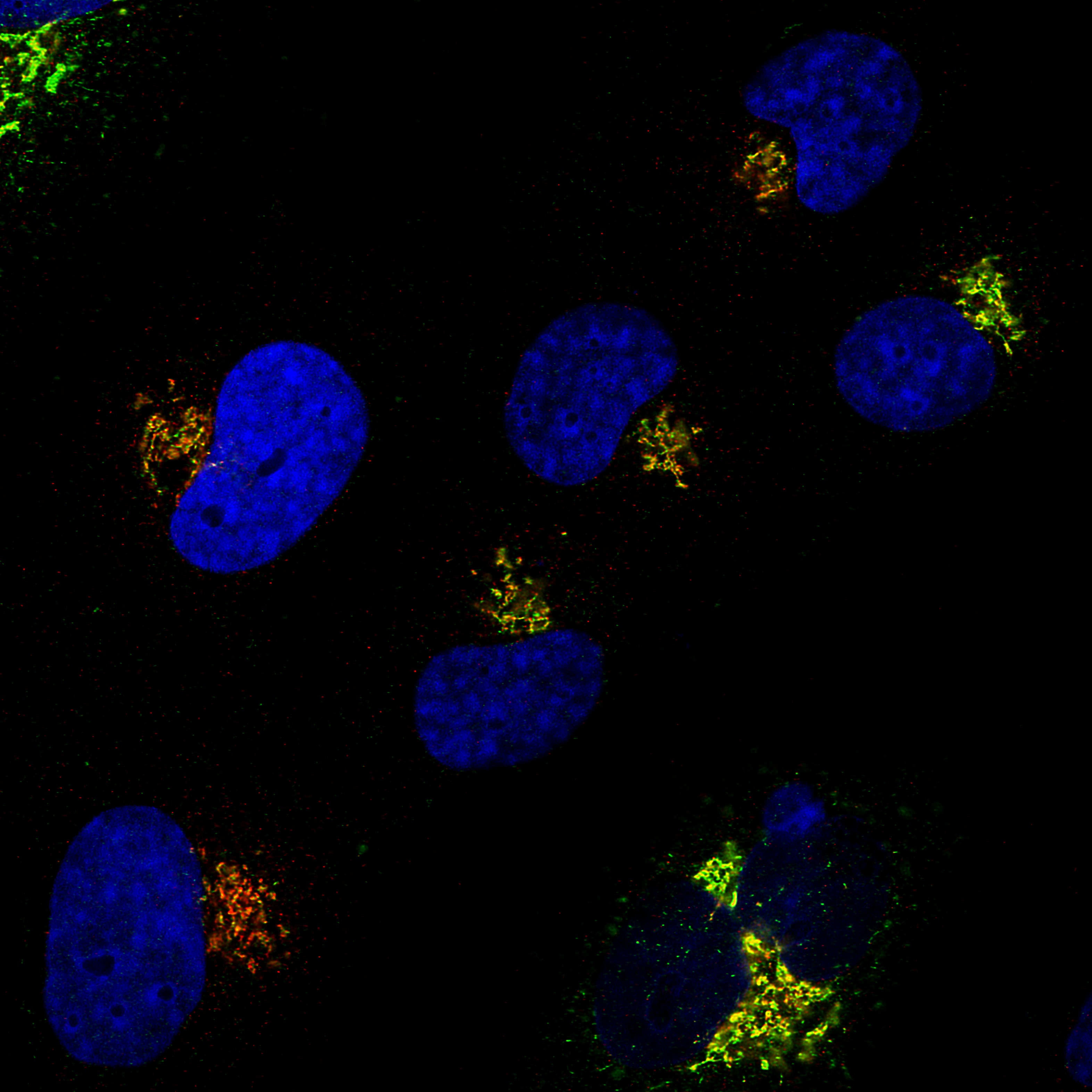

Supplement: Supplementary file 16 — Figure EV3 Source Data [file 44319_2026_773_MOESM16_ESM.zip › Figure EV3/Figure EV 3A/IF GRASP55KO+WT GRASP55_GM130 MERGE.tif]

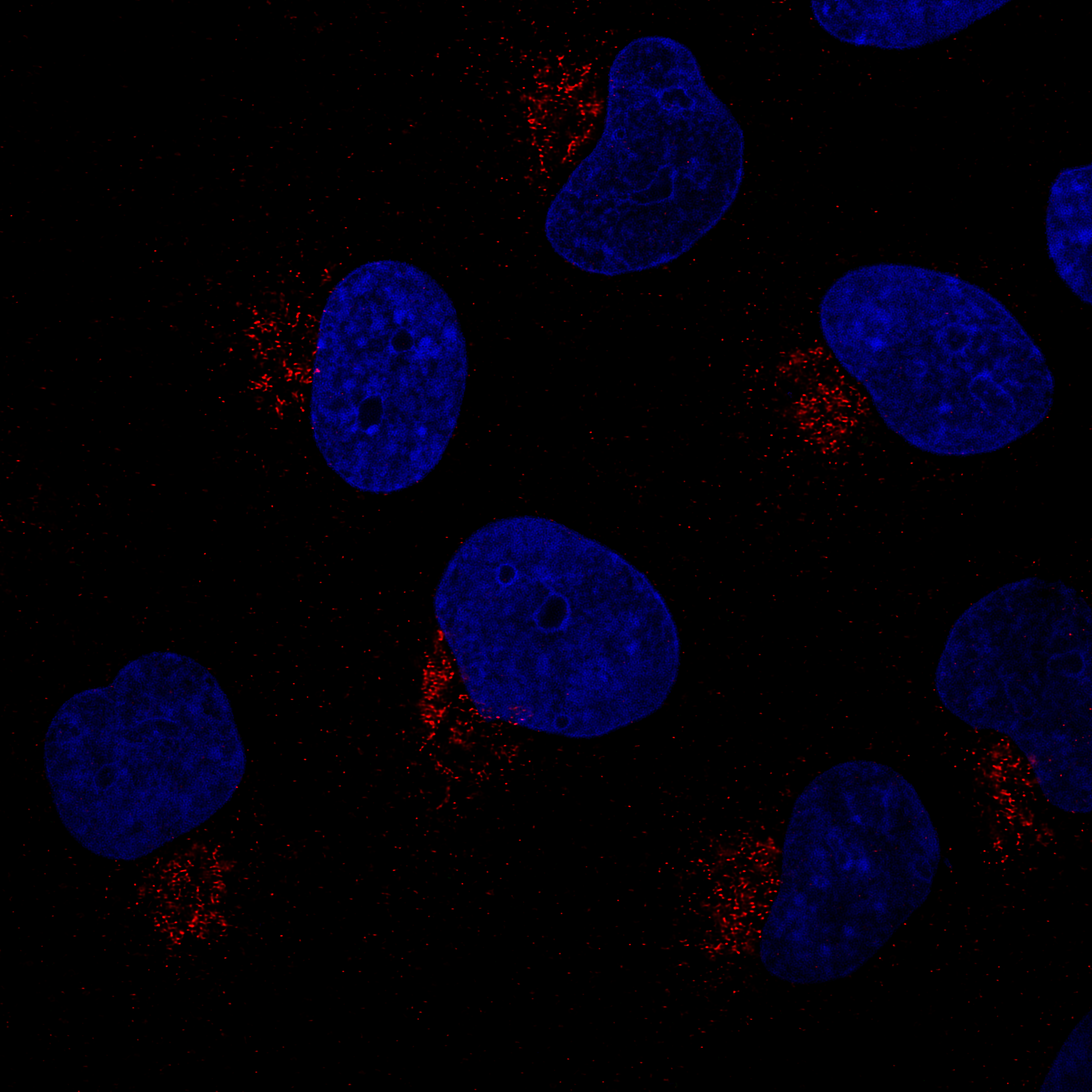

Supplement: Supplementary file 16 — Figure EV3 Source Data [file 44319_2026_773_MOESM16_ESM.zip › Figure EV3/Figure EV 3A/IF GRASP55KO GRASP55_GM130 MERGE.tif]

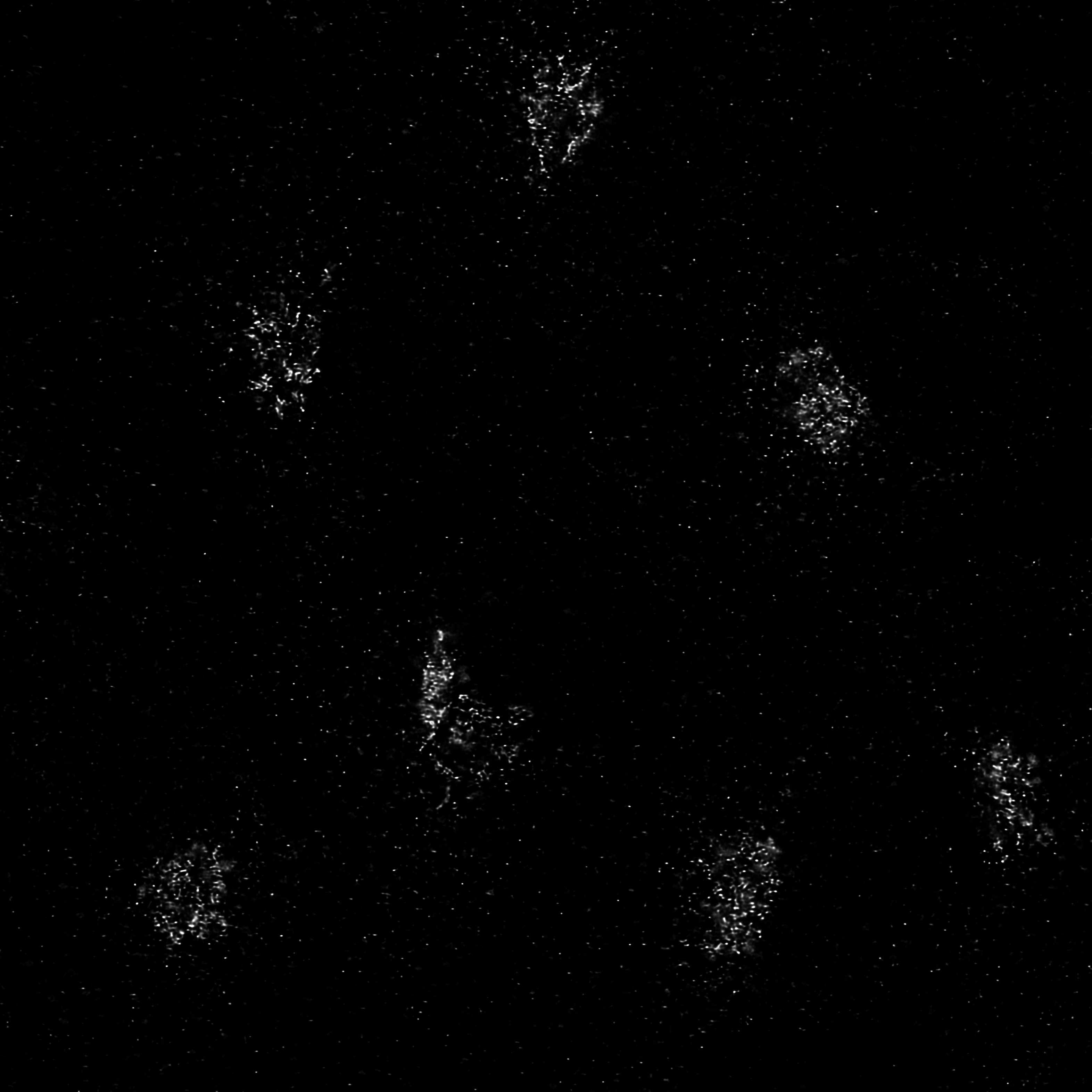

Supplement: Supplementary file 16 — Figure EV3 Source Data [file 44319_2026_773_MOESM16_ESM.zip › Figure EV3/Figure EV 3A/IF GRASP55KO GM130.tif]

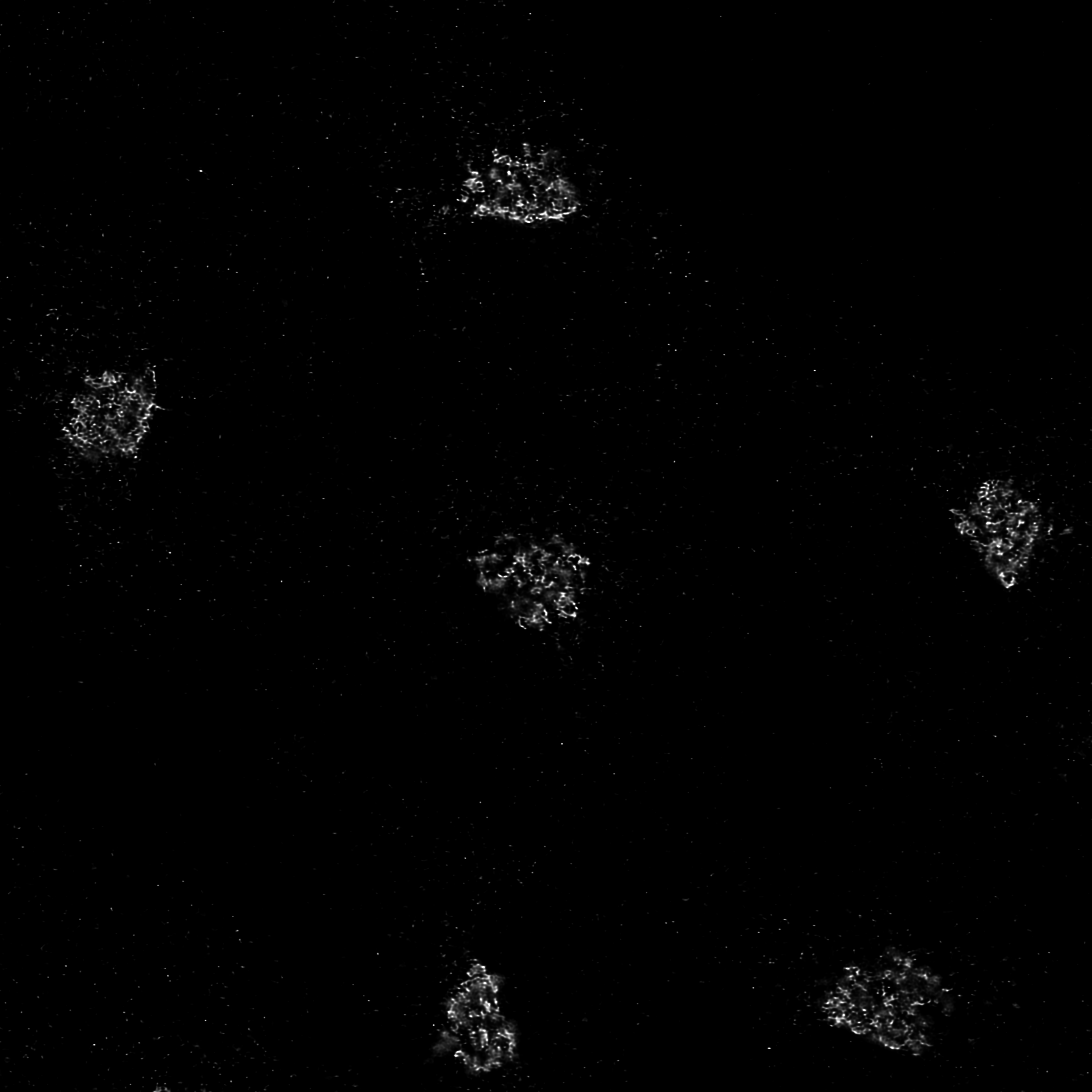

Supplement: Supplementary file 16 — Figure EV3 Source Data [file 44319_2026_773_MOESM16_ESM.zip › Figure EV3/Figure EV 3A/IF WT GM130.tif]

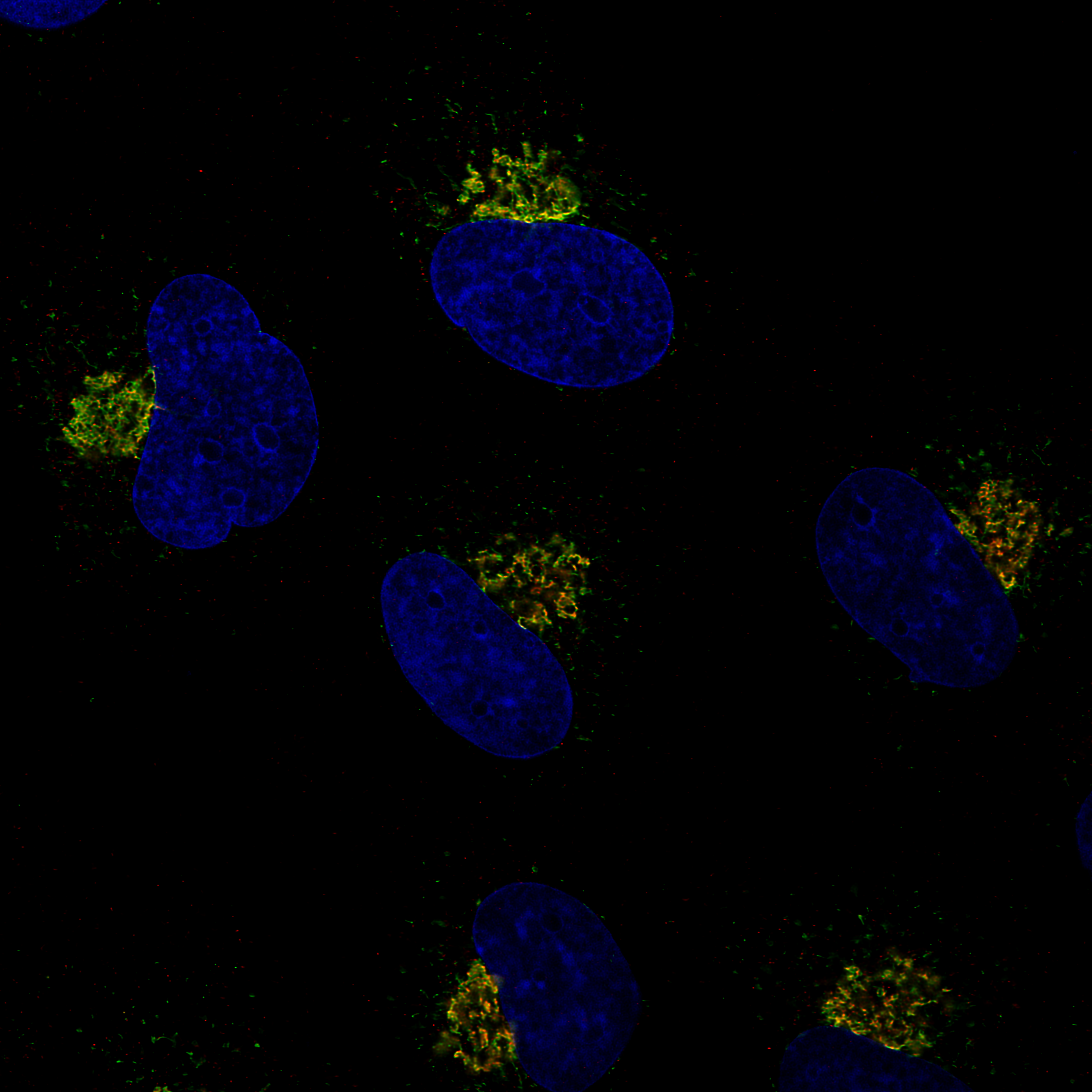

Supplement: Supplementary file 16 — Figure EV3 Source Data [file 44319_2026_773_MOESM16_ESM.zip › Figure EV3/Figure EV 3A/IF WT GRASP55_GM130 MERGE.tif]

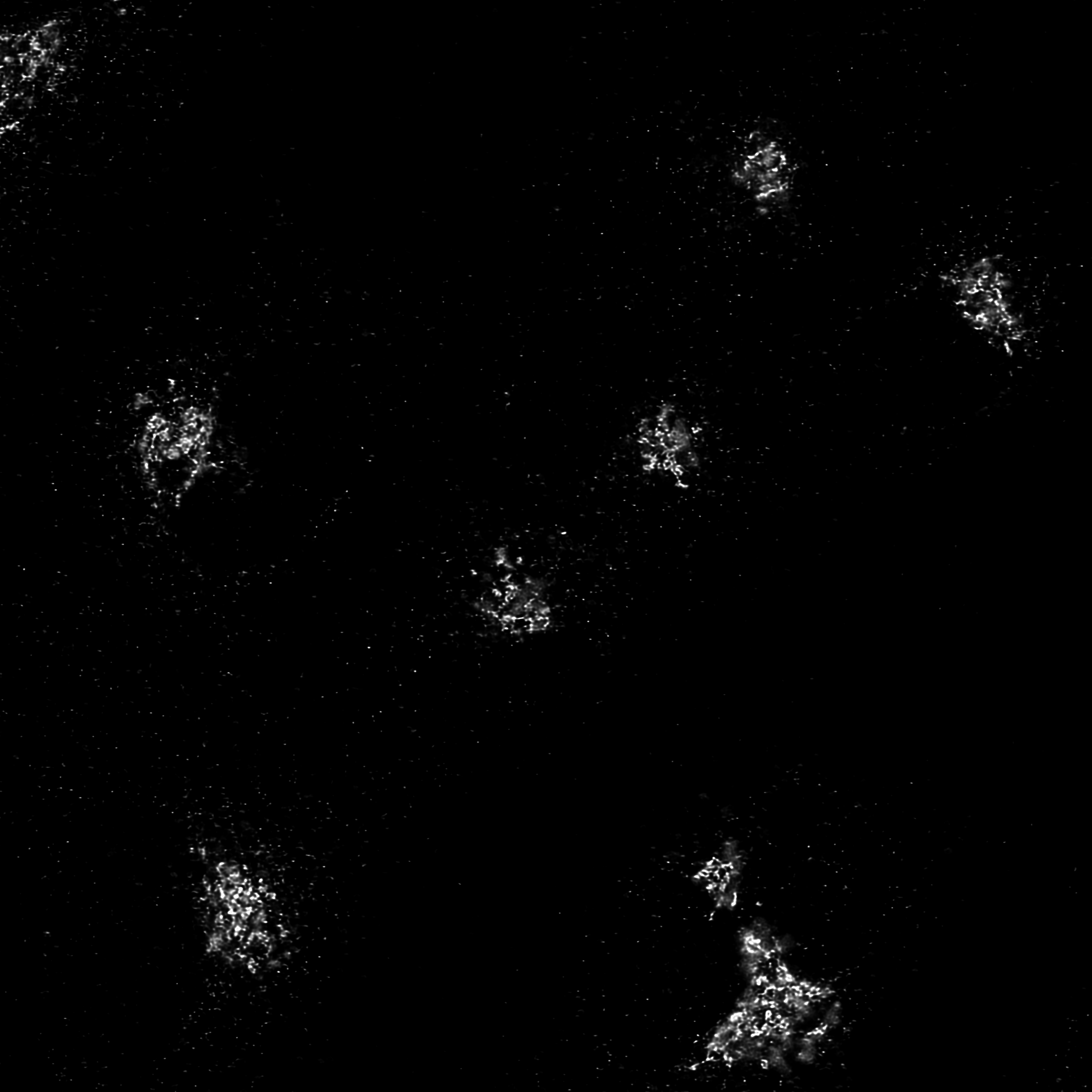

Supplement: Supplementary file 16 — Figure EV3 Source Data [file 44319_2026_773_MOESM16_ESM.zip › Figure EV3/Figure EV 3A/IF GRASP55KO+WT GM130.tif]

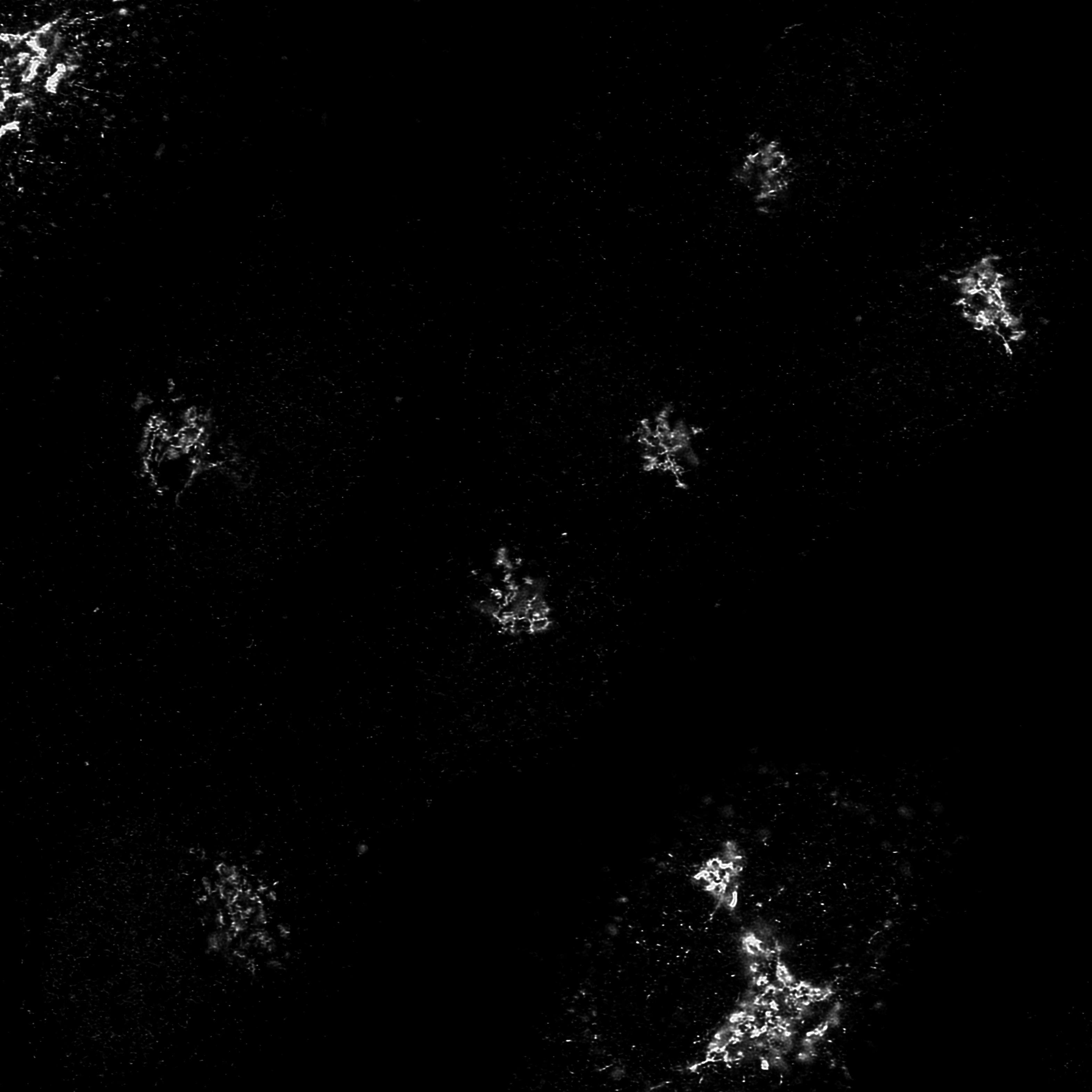

Supplement: Supplementary file 16 — Figure EV3 Source Data [file 44319_2026_773_MOESM16_ESM.zip › Figure EV3/Figure EV 3A/IF GRASP55KO+WT GRASP55.tif]

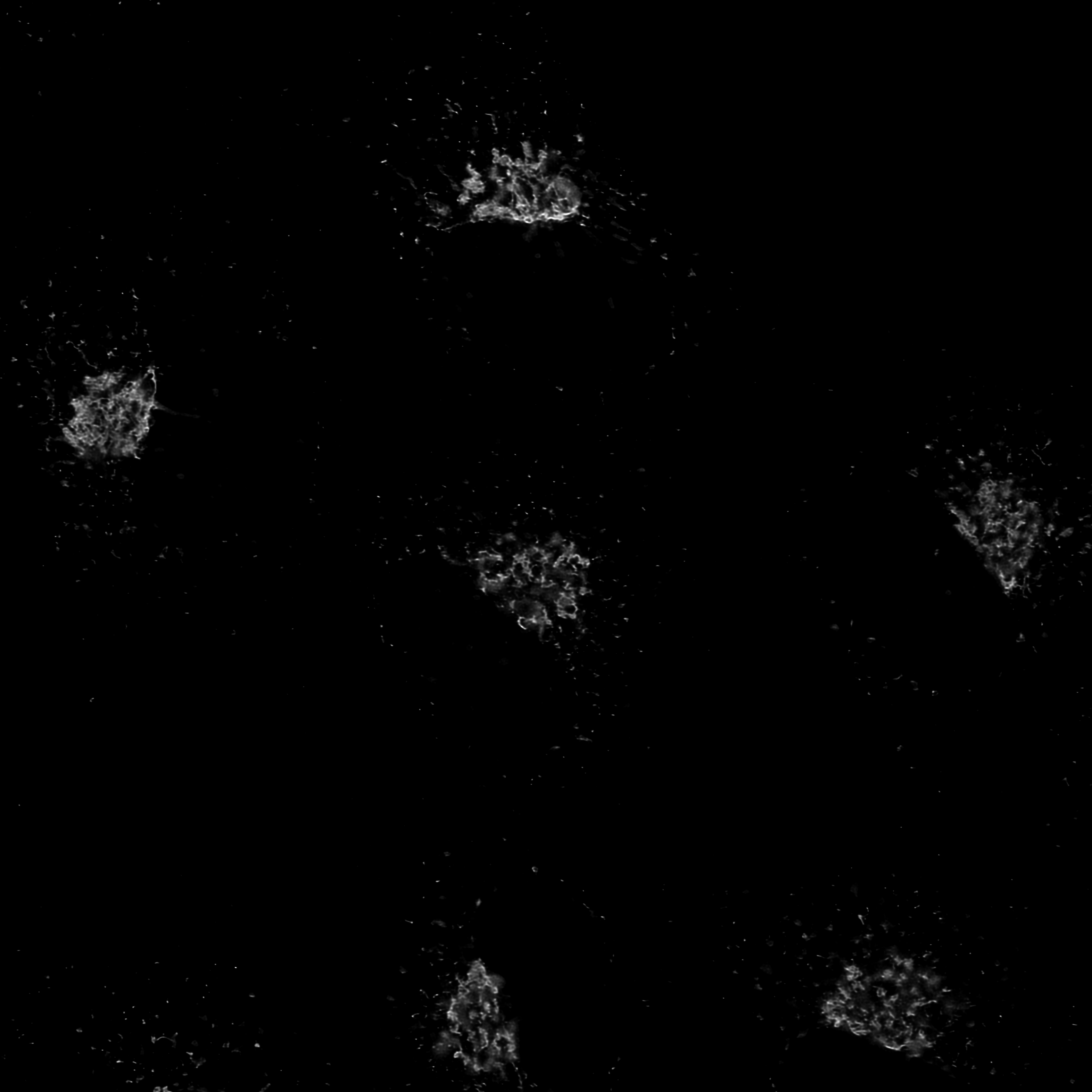

Supplement: Supplementary file 16 — Figure EV3 Source Data [file 44319_2026_773_MOESM16_ESM.zip › Figure EV3/Figure EV 3A/IF WT GRASP55.tif]
