# Supplementary figures and images for: GRASP55 maintains lysosome function by controlling sorting of lysosomal enzymes at the Golgi (part 5 of 5)
Source: EMBO Rep. 2026 Apr 16;27(11):2947–72. doi: 10.1038/s44319-026-00773-w (PMC13261057; doi:10.1038/s44319-026-00773-w)

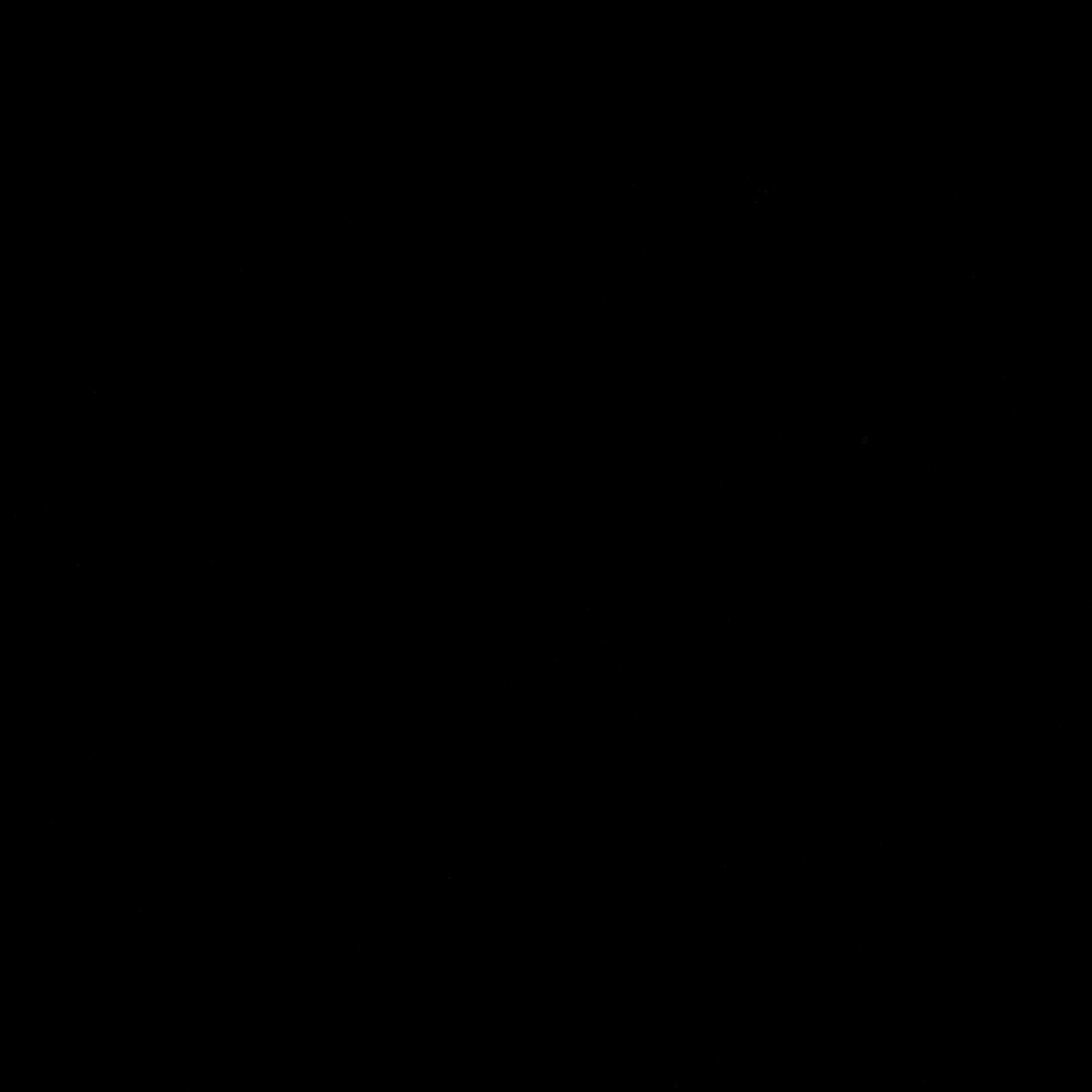

Supplement: Supplementary file 16 — Figure EV3 Source Data [file 44319_2026_773_MOESM16_ESM.zip › Figure EV3/Figure EV 3A/IF GRASP55KO GRASP55.tif]
