# Supplementary material for: GRASP55 maintains lysosome function by controlling sorting of lysosomal enzymes at the Golgi
Source: EMBO Rep. 2026 Apr 16;27(11):2947–72. doi: 10.1038/s44319-026-00773-w (PMC13261057; doi:10.1038/s44319-026-00773-w)
Supplement: Supplementary file 17 — Expanded View Figures [file 44319_2026_773_MOESM17_ESM.pdf]

Expanded View Figures

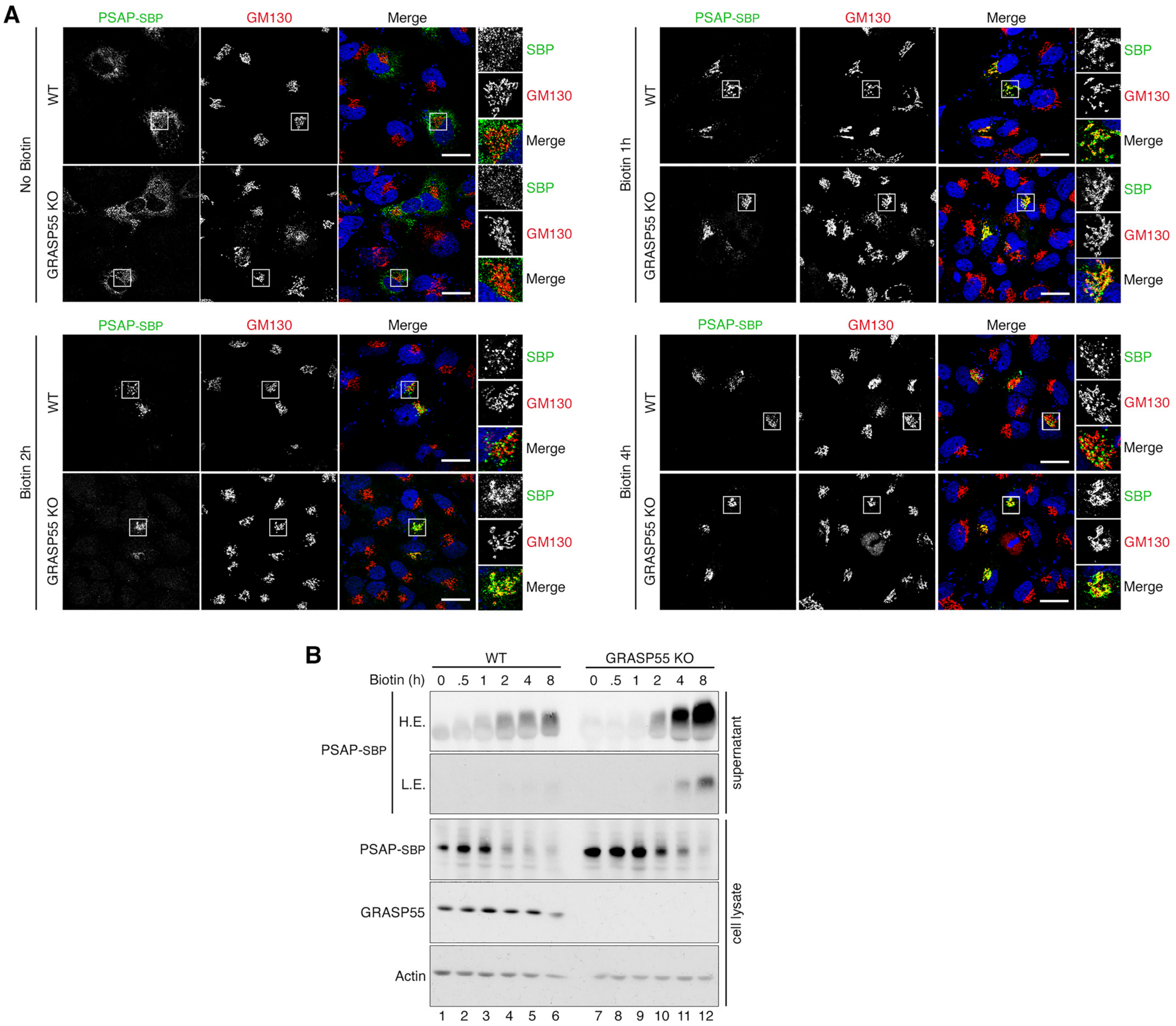

Figure EV1. Missorting of exogenous SBP-tagged PSAP GRASP55 KO cells, detected using a RUSH assay.

Related to Fig. 1. (**A**) Immunofluorescence analysis of WT or GRASP55 KO WI-26 cells transiently expressing streptavidin-binding peptide (SBP)-tagged prosaposin (PSAP) at the indicated time points following biotin addition. GM130 used as a Golgi marker. Nuclei stained with DAPI. Scale bars, 10 µm. $n = 3$ independent experiments. (**B**) Representative immunoblot analysis of cell lysates and supernatants from WT or GRASP55 KO WI-26 cells transiently expressing SBP-tagged PSAP at the indicated time points following biotin addition. GRASP55 and Actin immunoblots were used as controls. $n = 2$ independent experiments Source data are available online for this figure.

© The Author(s)

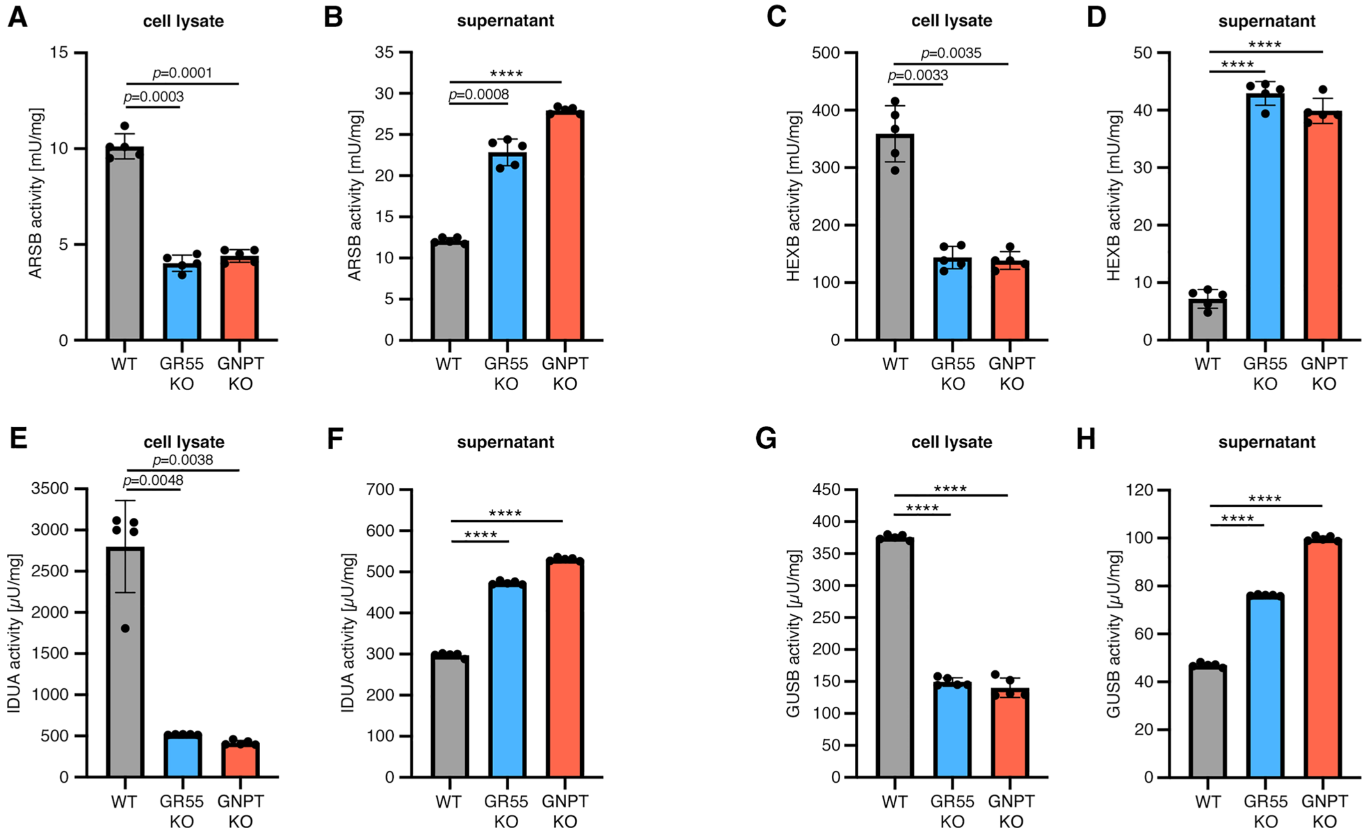

Figure EV2. GRASP55 KO cells exhibit decreased intracellular and elevated extracellular lysosomal enzyme activity, similar to GNPTAB KO cells.

Related to Fig. 3. (**A**, **B**) The enzymatic activity of arylsulfatase B (ARSB) was assayed in cell lysates (**A**) or supernatants (**B**) of wild-type (WT), GRASP55 KO (GR55 KO), or GNPTAB KO (GNPT KO) WI-26 cells (as a cellular model of mucolipidosis type II). (**C**, **D**) As in (**A**, **B**), but for β-hexosaminidase (HEXB) activity. (**E**, **F**) As in (**A**, **B**), but for α-L-iduronidase (IDUA) activity. (**G**, **H**) As in (**A**, **B**), but for β-glucuronidase (GUSB) activity. For all panels, $n = 5$ independent measurements from four biological replicates. Data in graphs shown as mean ± SD. ****$P < 0.0001$; P values ≥ 0.0001 are shown directly in the figures (one-way ANOVA) Source data are available online for this figure.

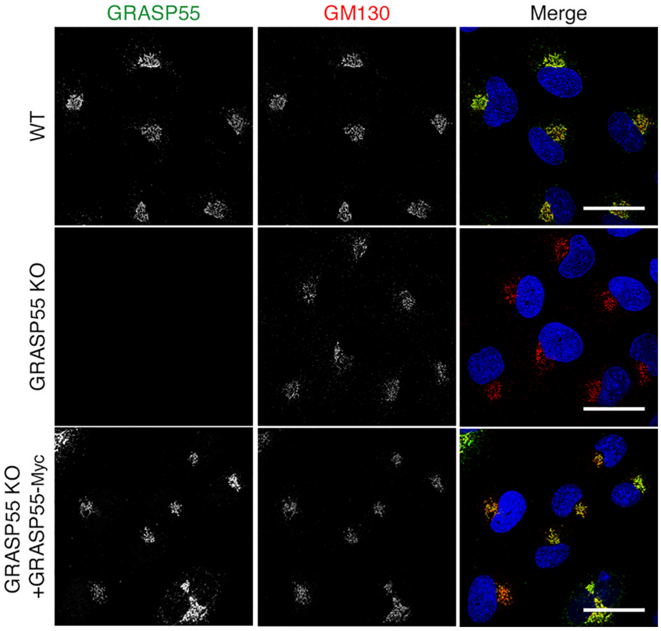

Figure EV3. Stably-expressed Myc-tagged GRASP55 localizes at the Golgi, resembling endogenous GRASP55 localization.

Related to Fig. 4. Immunofluorescence analysis of WT or GRASP55 KO WI-26 cells, or GRASP55 KO cells stably re-expressing Myc-tagged GRASP55 at near-endogenous levels. GM130 used as a Golgi marker. Nuclei stained with DAPI (blue). Scale bars, 10 µm. $n = 2$ independent experiments Source data are available online for this figure.

© The Author(s)
