# Supplementary material for: GRASP55 maintains lysosome function by controlling sorting of lysosomal enzymes at the Golgi
Source: EMBO Rep. 2026 Apr 16;27(11):2947–72. doi: 10.1038/s44319-026-00773-w (PMC13261057; doi:10.1038/s44319-026-00773-w)
Supplement: Supplementary file 3 — Peer Review File [file 44319_2026_773_MOESM3_ESM.pdf]

EMBO reports

GRASP55 maintains lysosome function by controlling sorting of lysosomal enzymes at the Golgi

Julian Nuechel, Maryam Omidi, Stephanie Fernandes, Marina Tauber, Sandra Pohl, Markus Plomann, and Constantinos Demetriades

Corresponding author(s): Constantinos Demetriades (Demetriades@age.mpg.de) , Julian Nuechel (nuechel.julian@uni-koeln.de)

Review Timeline:

Transfer Date:	19th May 25
Editorial Decision:	6th Jun 25
Revision Received:	4th Jan 26
Editorial Decision:	23rd Feb 26
Revision Received:	13th Mar 26
Accepted:	23rd Mar 26

Editor: Martina Rembold

Transaction Report: This manuscript was transferred to EMBO reports following peer review at The EMBO Journal.

(Note: With the exception of the correction of typographical or spelling errors that could be a source of ambiguity, letters and reports are not edited. Depending on transfer agreements, referee reports obtained elsewhere may or may not be included in this compilation. Referee reports are anonymous unless the Referee chooses to sign their reports.)

Referee #1:

Lysosomal hydrolases are critical for cellular function. They are sorted to the Golgi by means of a mannose 6-phosphate (M6P) signal that is attached to their glycans in the Golgi apparatus and then recognized by M6P receptors (MPRs) that direct the hydrolases to late endosomes for delivery to the lysosome. GRASP55 is a PDZ-domain containing Golgi protein that is recruited to the cytosolic surface of Golgi membranes by an N-terminal lipid anchor. It has been reported to have several possible functions including organising the structure of the Golgi, helping direct specific proteins through the Golgi, and even a role in unconventional protein secretion that bypasses the Golgi.

The authors have previously reported an examination of the role of GRASP55 in unconventional protein secretion which included determining the secretome of cells lacking GRASP55 (Nuschel et al. 2021). In this study they report that reanalyzing this secretome data revealed that removal of GRASP55 resulted in substantial secretion of lysosomal hydrolases from the cell. They confirm this using a variety of assays, including blotting for individual enzymes, following the traffic of an exogenously expressed enzyme, measuring lysosomal hydrolase activity inside cells, and examining the location of mTOR complex which they have previously shown to be released from the surface of lysosomes when hydrolase sorting is defective. All of this extensively confirms that lysosomal hydrolase are released from GRASP55 KO cells, and the data is carefully quantified and clearly presented.

To investigate mechanism, they follow up the fact that the GNPTAB enzyme that adds the M6P group is also released from cells lacking GRASP55. They express a tagged version of this Golgi membrane protein and find that when GRASP55 is missing the protein is partly mislocalized to the ER and degraded. They present immunoprecipitation data to argue that GRASP55 binds directly to GNPTAB. Finally, they use proximity biotinylation to determine a GRASP55 "proximome" and find amongst various Golgi proteins subunits of the COG complex that has a role in vesicle transport in the Golgi and is known to be required for efficient glycan modification in the Golgi. They present evidence that GRASP55 also binds to COG, and that cells lacking subunits of the COG complex show elevated secretion of lysosomal hydrolases.
There is quite a lot of interest in the mechanism by which lysosomal hydrolases are modified and sorted to the vacuole. In addition, the roles of GRASP55, and its paralog GRASP65 (which does not have a role here), are unresolved and so clear data on their function would be valuable. The first five figures of the paper are of a high standard, but all make essentially the same point albeit with different assays. The more mechanistic

aspects of the work are less robust, and there is a slight issue of novelty which could have been better handled. These points are explained fully below, but taken together they mean that the work would require substantial improvement and revision before publication in a general interest journal like the EMBO Journal.

A) In October 2024 a paper was posted in bioRxiv reporting that deletion of GRASP55 results in secretion of lysosomal hydrolases correlating with a perturbation in levels of GNPTAB, and this study has now appeared in print in Molecular Biology of the Cell (Akaaboune et al.; PubMed ID 39841559). Of course, it is likely that the work described in this submission was started ahead of the bioRxiv post, and the characterization here is certainly more extensive, but the authors really should have added a note to the effect that another study has reported similar conclusions whilst they were preparing theirs for submission.

B) The various experiments confirming that in the GRASP55 KO cells lysosomal hydrolases are secreted rather than intracellular are of a high standard and convincing. However, the data to support the proposed mechanism behind this effect needs more depth and better assays. The authors use immunoprecipitation of tagged GNPTAB from whole cell lysates to argue that GNPTAB binds to GRASP55. However, such an experiment does not demonstrate direct binding, and moreover the immunoprecipitation seems rather odd with myc-tagged GNPTAB not being highly enriched in the anti-myc IP, and the amount of GRASP55 that is coprecipitated being rather low (Figure 6E). GRASP55-GFP was analysed in the OpenCell large scale PPI screen where it was tagged in the genome and GNPTAB was not among the proteins that co-precipitated with it (PubMed ID 35271311). In addition, it is known that GNPTAB forms a complex with a small membrane protein called LYSET which binds a COPI interactor called GOLPH3, and so even if the co-ip is correct then the interaction could be directly with GNPTAB or indirectly through LYSET, GOLPH3 or even some other factor. Thus, this binding data would be more convincing if the cytoplasmic domains of these various proteins were tested with recombinant GRASP55 to identify and define a direct interaction.

C) The authors use an APEX fusion and proximity biotinylation to identify proteins near to GRASP55 and then test the COG complex which they suggest also co-immunoprecipitates. Such proximity biotinylation does not measure an "interactome" as the authors state, especially as APEX has a large radius of action compared to BirA, but it will simply label proteins that happen to be in the same location in the cell. Many Golgi proteins are enriched as would be expected, and it is not clear why GRASP55 should interact with the COG complex, especially as this interaction has not been previously observed in large scale PPI screens that have found known GRASP55 interactors. The authors show

lysosomal hydrolase missorting in cells lacking COG subunits, but it is known that such lines have strong defects in glycan processing in the Golgi due to defects in vesicle recycling and given that M6P is added to glycans in the Golgi then the effect on hydrolases could be a consequence of a general defect in glycans rather than a specific effect via GRASP55 or GNPTAB. Indeed, the general perturbation of glycan processing in the COG subunit KO lines can be seen in the increased mobility of some of the hydrolases tested here (Figure 7F).

D) The authors should have cited and briefly discussed the fact that mice lacking GRASP55 have male fertility defects but the females are viable and fertile which suggests that GRASP55 has a rather limited role that it is unique and instead there is greater redundancy with GRASP65 (PubMed ID 28617811).

Overall, the data in Figures 1-6 (without Figure 6E) would make a good quality descriptive paper in a specialized journal on the requirement for GRASP55 for efficient sorting of lysosomal hydrolases. However, if the authors wish to add mechanistic insight and hence raise the work to the standard expected for a broad interest journal such as the EMBO Journal then substantial new data would be required to make robust mechanistic conclusions.

Referee #2:

This manuscript describes a role for the Golgi resident protein GRASP55 in the correct trafficking of lysosomal enzymes to the lysosome. GRASP55 functions in this process by maintaining the enzyme that adds the key mannose 6-phosphate tag to the enzymes, GNPTAB, in the Golgi. In the absence of GRASP55, GNPTAB is localized to the ER instead of the Golgi, and the lysosomal enzymes are missorted and secreted upon Golgi exit instead of being delivered to the lysosomes. This results in swollen dysfunctional lysosomes that have defective mTOR signaling. Interaction data show that GRASP55 can bind to GNPTAB, and that it can also interact with the Golgi vesicle tethering complex COG.

This is an interesting paper that is very nicely done. The data are clear and support the authors' conclusions. However, while I liked this study, I think it suffers from a lack of mechanistic information, and the novelty of the major findings is also diminished by previous work. I elaborate on the points below.

Major comments:

1.) While it is shown that GRASP55 can bind to GNPTAB and to the COG complex, how it maintains GNPTAB in the Golgi remains unclear. Does it bind to GNPTAB to anchor or retain it in the Golgi? Does it help GNPTAB get sorted into retrograde COPI vesicles to retain it there? Does it promote ER-Golgi traffic of GNPTAB or prevent its recycling to the ER? It remains unclear. Recent papers have shown that LYSET also keeps GNPTAB in the Golgi, acting through the GOLPH proteins for sorting into COPI vesicles. How does GRASP55 function compare to that of LYSET? The interaction with COG might suggest a role in vesicle traffic but it is not explored.

2.) The experiments with COG do not inform us of GRASP55 function. Although there is some degree of phenotypic overlap in the GRASP55 and COG KO cells, it is likely that the COG is acting independently considering that it is required for proper localization of numerous Golgi enzymes, resulting in major defects in glycan addition and processing, as opposed to just the GNPTAB enzyme. Also, it was shown recently that stable KO of COG results in a myriad of trafficking phenotypes, many likely indirect, greatly complicating the interpretation of the experiments here.

3.) In terms of novelty, previous studies have shown that GRASP55 retains Golgi enzymes involved in sphingolipid synthesis in the Golgi, but even more relevant for this study is a recent paper showing that GRASP55 is required for GNPTAB stability and correct lysosomal hydrolase sorting. The potential additional novelty in this paper might therefore come from the analysis of mTOR signaling, but it was also shown recently by the same authors that lysosomal dysfunction affects this process. My feeling is that without additional exploration of GRASP55 mechanism regarding GNPTAB function, or the regulation of GRASP55 by mTOR in the context of lysosomal sorting, the paper lacks some novelty.

Additional comments:

1.) In Fig S1 (the RUSH experiment) the expression of PSAP looks to be higher in the KO cells. The apparent increased secretion is a little misleading considering the different expression level. The rate of exit from the cells looks similar in WT and KO.

2.) In Fig 2, the lysosomal hydrolases look to be in spotty structures in the GRASP55 KO (CTSB, CTSD, PSAP). If these structures are not lysosomes, what are they? If the enzymes are missorted it would be good to know where they go.

3.) In Fig 6, the altered processing of GNPTAB is shown. This could be a consequence of its altered localization, but equally it could be due to altered localization of the S1P protease, which cycles between the ER and Golgi. Can the authors localize S1P to exclude a defect in its own trafficking?

4.) Text, line 162, states "Focusing on the proteins whose secretion is blunted in GRASP55 KO cells, we observed a robust enrichment of extracellular matrix (ECM)- and cell

adhesion-related factors, indicating that GRASP55 controls such cellular processes through the regulation of UPS". The first part of this sentence is confusing and oxymoronic- secretion that is blunted but enriched? The second part is not justified- ECM is mainly secreted by conventional secretion so if ECM proteins are altered is not an indication of UPS regulation.

5.) Text, line 318, states "reduced expression of Myc-tagged GNPTAB". It only indicates reduced abundance not expression per se.

6.) Text, line 473, states ER to Golgi transport of enzymes is affected by GRASP55 KO. The RUSH data in Fig S1 suggest this step is not affected by GRASP55 KO.

Referee #3:

Nuchel et al. report a novel function for GRASP55 in lysosomal enzyme trafficking. Through reanalysis of their previously published secretome of GRASP55 KO cells, the authors discover increased secretion of various lysosomal enzymes in their immature form. By further investigating the phenotypes of GRASP55 KO cells, the authors demonstrate decreased cellular levels and activity of lysosomal enzymes, which is accompanied by accumulation of enlarged lysosomes and perturbed mTORC1 signaling. Mechanistically, the authors link these phenotypes to defects in the trafficking and localization of GNPTAB in the absence of GRASP55. This study identifies interesting and striking phenotype of GRASP55 KO cells, which is timely in light of the emerging realization that Golgi processes are critical for lysosomal biogenesis and hence function. The data are presented in well-structured figures, although the manuscript is somewhat digressive and the authors tend to overinterpret their results. A large part of the manuscript is dedicated to documenting the lysosomal defects of GRASP55 KO, but the mechanistic part concerning the role of GRASP55 in GNPTAB trafficking and localization is immature and the proposed mechanism speculative. While this manuscript is of potential interest, substantial additional experiments are required to achieve a mechanistic depth appropriate for publication in the EMBO Journal.

Major points:

1. The authors report decreased protein levels and ER relocalization of ectopically expressed GNPTAB in GRASP55 KO cells (Fig. 5), but how GRASP55 controls GNPTAB localization and stability remains a mystery. Does GNPTAB localize to the ER in GRASP55 KO cells due to decreased anterograde trafficking to or retrograde trafficking from the

Golgi? How, mechanistically, does the Golgi-resident GRASP55 affect this process? Time course studies of GNPTAB trafficking and processing as well as manipulation of the underlying trafficking pathways might help resolve these questions. The authors further suggest that GRASP55 controls intra-Golgi trafficking of GNPTAB (lines 362-364) - are there any data supporting this claim?

2. Significant progress has been made over the past several years in understanding how correct Golgi localization of GNPTAB is controlled, including demonstrations of GNPTAB Golgi-retention as a COPI-dependent process mediated by GOLPH3/GOLPH3L and LYSET and membrane stabilization of GNPTAB by LYSET (PMID: 36074821, 36074822, 36096887, 39587297, 39937677). It is curious that the authors do not, at least to some extent, attempt to place their findings into this mechanistic framework. Does GRASP55 KO affect LYSET localization or levels? How is GRASP55-mediated Golgi trafficking / retention of GNPTAB related to the COPI/GOLPH3-dependent process?

3. Experiments concerning GNPTAB are based on ectopic overexpression, which does not necessarily reflect the localization, abundance and processing of the endogenous protein or recapitulate the stoichiometry of the heteromeric phosphotransferase complex. Indeed, over-expressed GNPTAB-myc leads to much higher levels of the precursor protein than the processed beta-subunit, which in addition is degraded by the proteasome to a similar extent in WT and GRASP55 KO cells (Fig. 6F). It would be important to demonstrate key results at endogenous protein level.

4. Some of the phenotypes in GRASP55 KO cells are inconsistent, especially with relation to GNPTAB (supposed to be similar) and GRASP65 (supposed to be different). In particular, a reduction of M6P levels is hard to discern (Fig. 1D, 7F) and the levels are indistinguishable from of those in GRASP65 cells. This contrasts to the hypersecretion of enzymes in GRASP55 KO but not GRASP65 KO cells, raising the question whether a reduction of M6P levels is responsible for this phenotype.

5. The entire manuscript appears to be based on a single cell line and a single knockout clone for each of the genes under investigation. To demonstrate robustness of the findings, one would expect key findings to be validated in multiple cell lines / with multiple sgRNAs.

6. The manuscript concludes with the finding that GRASP55 interacts with the COG complex, whose knockout also leads to lysosomal enzyme secretion and lysosomal bloating (Fig. 7). These results, while potentially interesting, appear like an afterthought - except that the lysosomal defects of COG KO cells are much stronger than those of

GRASP55 KO cells. This suggests that the COG complex either regulates enzyme trafficking through GRASP55-dependent and independent pathways (no data for GNPTAB are provided in this context) or has a pleiotropic effect on Golgi trafficking / function. It would be helpful if the authors could provide some perspective on this.

7. The text would benefit from revision to more accurately reflect and discuss results. Examples are given below:

The title states that GRASP55 safeguards lysosomal function. I am not aware of the term safeguard being used when referring to the M6P pathway, and it would help if the authors could clarify its meaning.

In the abstract, the authors refer to GRASP55 as a key regulator of lysosome function - what is being regulated how and in response to what input remains elusive.

Line 145, 291: "cytoplasmic targets, like 4E-BP1". No cytoplasmic targets other than 4E-BP1 are examined.
The discussion has a lengthy paragraph termed "GRASP55 lies at the center of mTORC1 signaling", which is very strange considering that the manuscript reports perturbed mTORC1 signaling as an indirect consequence due to lysosomal dysfunction resulting from Golgi trafficking defects in GRASP55 KO cells. Please clarify the precise role of GRASP55 (i.e. not its absence) in mTORC1 signaling; alternatively consider removing this paragraph and focus on key points that are relevant to the present study.

Throughout the study, the authors claim to provide new insights into the molecular basis of LSD-related pathologies and suggest GRASP55 to act as a new LSD susceptibility gene. Has GRASP55 been associated with genetic diseases and do the patients present LSD-like phenotypes? This would indeed be very interesting and relevant. If such evidence is absent, then this could be an interesting but speculative idea that should be discussed in a more balanced way and removed from the abstract.

Minor points:

1. Review the references cited in lines 50 - 57. Are these really the most suitable to discuss the work over the last 15 years characterizing the lysosomal regulation of mTORC1 and TSC?

2. Since the authors previously identified GRASP55 to be phosphorylated and regulated by mTORC1, it would be very interesting to know whether this affects GNPTAB localization and lysosomal enzyme trafficking, which would point to a regulatory role of GRASP55 in this process.

1st Editorial Decision **6th Jun 2025**

Dear Costas,

Thank you for the transfer of your manuscript from our sister journal The EMBO Journal to EMBO Reports. Thank you also for the discussion about a potential revision. From our side, we noted that all three referees considered the data preliminary in the absence of mechanistic insight. All three referees considered it essential to provide further insight into the mechanism by which GRAP55 retains GNPTAB in the Golgi, whether the interaction is direct, and whether it involves pathways that have previously been described for GNPTAB retention in the Golgi, i.e., the COP I dependent pathway via GOLPH3/3L and LYSET. The referees further questioned the specificity of the COG deficiency phenotype, that seemed rather reflect the pleiotropic role of the COG complex.

We further noted that a recent paper reported similar findings on the role of GRASP55 in lysosomal enzyme sorting and GNPTAB function, which compromised the novelty of your findings. Please note that our scooping protection formally only applies to manuscripts that are under review and actively under revision. Taking all these concerns and considerations into account, we would require at least some mechanistic insight into the GNPTAB trafficking role and its function in the context of GOLPH3/3L and LYSET for publication in EMBO Reports.

You have meanwhile sent an outline of how you plan to experimentally address the referee's concerns, in particular the concerns regarding the role of GRASP55 in the context of the known GNPTAB regulators GOLPH3 and LYSET, which will potentially provide the required mechanistic insights. As discussed, I would like to invite you to revise your manuscript along the lines you suggested in your proposal. I would also advice to follow the referee suggestion to remove the data on the COG complex, which might form the basis of future studies.

Once you have submitted the revised manuscript, I will walk the referees through our review process outlining the discussed requirements from our side (as listed above). Acceptance of the manuscript will depend on a positive outcome of a second round of review.

I am always happy to discuss this further on a video chat.

We realize that it is difficult to revise to a specific deadline. In the interest of protecting the conceptual advance provided by the work, we recommend a revision within 3 months (September 6). Please discuss the revision progress ahead of this time with the editor if you require more time to complete the revisions.

You can either publish the study as a short report or as a full article. For short reports, the revised manuscript should not exceed 27,000 characters (including spaces but excluding materials & methods and references) and 5 main plus 5 expanded view figures. The results and discussion sections must further be combined, which will help to shorten the manuscript text by eliminating some redundancy that is inevitable when discussing the same experiments twice. For a normal article there are no length limitations, but it should have more than 5 main figures and the results and discussion sections must be separate. In both cases, the entire materials and methods must be included in the main manuscript file.

================
IMPORTANT NOTE:

We perform an initial quality control of all revised manuscripts before re-review. Your manuscript will FAIL this control and the handling will be delayed IN CASE the following APPLIES:

1) A data availability section providing access to data deposited in public databases is missing. If you have not deposited any data, please add a sentence to the data availability section that explains that.

2) Your manuscript contains statistics and error bars based on n=2. Please use scatter blots in these cases. No statistics should be calculated if n=2.

When submitting your revised manuscript, please carefully review the instructions that follow below. Failure to include requested items will delay the evaluation of your revision.

================

When submitting your revised manuscript, we will require:

1) a .docx formatted version of the manuscript text (including legends for main figures, EV figures and tables). Please make sure that the changes are highlighted to be clearly visible.

2) individual production quality figure files as .eps, .tif, .jpg (one file per figure).
Please download our Figure Preparation Guidelines (figure preparation pdf) from our Author Guidelines pages https://www.embopress.org/page/journal/14693178/authorguide for more info on how to prepare your figures.

3) a .docx formatted letter INCLUDING the reviewers' reports and your detailed point-by-point responses to their comments. As part of the EMBO Press transparent editorial process, the point-by-point response is part of the Review Process File (RPF), which will be published alongside your paper.

4) a complete author checklist, which you can download from our author guidelines (<https://www.embopress.org/page/journal/14693178/authorguide>). Please insert information in the checklist that is also reflected in the manuscript. The completed author checklist will also be part of the RPF.

5) Please note that all corresponding authors are required to supply an ORCID ID for their name upon submission of a revised manuscript (<https://orcid.org/>). Please find instructions on how to link your ORCID ID to your account in our manuscript tracking system in our Author guidelines (<https://www.embopress.org/page/journal/14693178/authorguide#authorshipguidelines>)

6) We replaced Supplementary Information with Expanded View (EV) Figures and Tables that are collapsible/expandable online. A maximum of 5 EV Figures can be typeset. EV Figures should be cited as 'Figure EV1, Figure EV2" etc... in the text and their respective legends should be included in the main text after the legends of regular figures.

- For the figures that you do NOT wish to display as Expanded View figures, they should be bundled together with their legends in a single PDF file called *Appendix*, which should start with a short Table of Content. Appendix figures should be referred to in the main text as: "Appendix Figure S1, Appendix Figure S2" etc. See detailed instructions regarding expanded view here: <https://www.embopress.org/page/journal/14693178/authorguide#expandedview>

- Additional Tables/Datasets should be labeled and referred to as Table EV1, Dataset EV1, etc. Legends have to be provided in a separate tab in case of .xls files. Alternatively, the legend can be supplied as a separate text file (README) and zipped together with the Table/Dataset file.

7) The "Data Availability" section at the end of the Methods should be formatted as follows: "The [structural coordinates | microarray | mass spectrometry] data from this publication have been deposited to the [name of the database] database [URL] and assigned the identifier [accession | permalink | hashtag]."). Should this not apply, this should still be stated as "This study includes no data deposited in external repositories."

8) At EMBO Press we ask authors to provide source data for the main manuscript figures. You will receive a separate email with instructions for providing source data with your revised manuscript, including how to upload and organize the files.

Additional information on source data and instruction on how to label the files are available <https://www.embopress.org/page/journal/14693178/authorguide#sourcedata>

9) The journal requires a statement specifying whether or not authors have competing interests (defined as all potential or actual interests that could be perceived to influence the presentation or interpretation of an article). In case of competing interests, this must be specified in your disclosure statement. Further information: https://www.embopress.org/competing-interests

10) Figure legends and data quantification:
The following points must be specified in each figure legend:

- the name of the statistical test used to generate error bars and P values,
- the EXACT p-values,
- the number (n) of independent experiments (please specify technical or biological replicates) underlying each data point,
- the nature of the bars and error bars (s.d., s.e.m.)

- If the data are obtained from n {less than or equal to} 5, show the individual data points in addition to the SD or SEM.
- If the data are obtained from n {less than or equal to} 2, use scatter blots showing the individual data points.

Discussion of statistical methodology can be reported in the materials and methods section, but figure legends should contain a basic description of n, P and the test applied.

See also the guidelines for figure legend preparation:
https://www.embopress.org/page/journal/14693178/authorguide#figureformat

- Please also include scale bars in all microscopy images and define their size in the legend, not the image.

11) Our journal encourages inclusion of *data citations in the reference list* to directly cite datasets that were re-used and obtained from public databases. Data citations in the article text are distinct from normal bibliographical citations and should directly link to the database records from which the data can be accessed. In the main text, data citations are formatted as follows: "Data ref: Smith et al, 2001" or "Data ref: NCBI Sequence Read Archive PRJNA342805, 2017". In the Reference list, data citations must be labeled with "[DATASET]". A data reference must provide the database name, accession number/identifiers and a resolvable link to the landing page from which the data can be accessed at the end of the reference. Further instructions are available at <https://www.embopress.org/page/journal/14693178/authorguide#referencesformat>.

12) All Materials and Methods need to be described in the main text using our 'Structured Methods' format. According to this format, the Methods section includes a Reagents and Tools Table (listing key reagents, experimental models, software and relevant equipment and including their sources and relevant identifiers) followed by a Methods and Protocols section describing the methods, ideally using a step-by-step protocol format. The aim is to facilitate adoption of the methodologies across labs. Please download and fill our Reagents and Tools Table template (.docx), which you can find in our author guidelines: https://www.embopress.org/page/journal/14693178/authorguide#structuredmethods.
When submitting your revised manuscript, please do not include the Reagents and Tools Table in the Methods section of the manuscript but upload it as a separate file choosing the file type "Reagent Table".
An example of a Method paper with Structured Methods can be found here: https://www.embopress.org/doi/10.15252/msb.20178071.

13) As part of the EMBO publication's Transparent Editorial Process, EMBO Reports publishes online a Review Process File to accompany accepted manuscripts. This File will be published in conjunction with your paper and will include the referee reports, your point-by-point response and all pertinent correspondence relating to the manuscript.

You are able to opt out of this by letting the editorial office know (emboreports@embo.org). If you do opt out, the Review Process File link will point to the following statement: "No Review Process File is available with this article, as the authors have chosen not to make the review process public in this case."

We would also welcome the submission of cover suggestions, or motifs to be used by our Graphics Illustrator in designing a cover.

I look forward to seeing a revised form of your manuscript when it is ready and please let me know if you have questions or comments regarding the revision.

Kind regards,

Martina

Martina Rembold, PhD
Senior Editor
EMBO reports

Response to Referees

EMBOR-2025-61967-T (former EMBOJ-2025-120203R-Q)

GRASP55 Safeguards Proper Lysosome Function by Controlling Sorting of Lysosomal Enzymes at the Golgi

Nüchel et al.

We sincerely thank the Reviewers for the careful evaluation of our manuscript and for the constructive comments. We acknowledge the Reviewers' view that the manuscript would benefit from further mechanistic insight into how GRASP55 controls GNPTAB localization. Therefore, we have now revised the manuscript based on their previous comments (for *EMBO J*) and our discussions with the new handling editor at *EMBO Reports*. In the revised manuscript, we have incorporated a large amount of new experiments and analyses to substantially strengthen the mechanistic depth of our study and improve clarity. In particular, we have now added data (**new Figs. 7 and 8**) and discussion strengthening the following points:

- We provide a larger part of the molecular mechanism of how GRASP55 controls GNPTAB localization and stability to ensure correct sorting and trafficking of lysosomal enzymes and hence proper lysosome function.
- We combined microscopy-based and biochemical (Golgi-IP, co-IPs) methods to explore the role of GRASP55 (using GRASP65 as a negative control) in the regulation of GNPTAB in the context of the previously described GOLPH3-LYSET machinery (**new Fig. 7**). In a nutshell, our new data show that GRASP55 is required for the Golgi localization of the complete GOLPH3-LYSET-GNPTAB complex. Not only GNPTAB, but also GOLPH3 and LYSET mislocalize away from the Golgi in GRASP55-deficient cells. In addition, we detect interactions between GRASP55 and all proteins of the GOLPH3-LYSET-GNPTAB complex, with the GRASP55-GOLPH3 interaction being unaffected by loss of LYSET, which suggests that the loss of GOLPH3 from the Golgi is likely the first step in a cascade of events downstream of GRASP55 loss. In other words, by GRASP55 ensuring correct localization of GOLPH3, it indirectly controls the localization of LYSET and GNPTAB. Importantly, this places GRASP55 upstream of the GOLPH3-LYSET machinery that was previously shown to control GNPTAB localization and lysosomal enzyme sorting; and highlights it as novel key factor in Golgi-lysosome communication.
- We find that, in GRASP55-deficient cells, LYSET missorting is accompanied by its lysosome-dependent degradation, confirming previous findings by the Voss group

(Brauer et al. 2024 EMBO J) describing GOLPH3 (and GOLPH3L) as a regulator of LYSET-GNPTAB.

- Again, not only the lysosomal enzyme secretion and the lysosomal deficiency phenotypes, but also the effects on GOLPH3, LYSET and GNPTAB localization and stability are specific for GRASP55, as loss of the closely related GRASP65 does not recapitulate these effects. This also shows these are not generic effects of perturbed Golgi architecture, as both GRASP55 and 65 are known to play an important role and work together in this process.
- Using a GNPTAB mutant that was previously described to not require LYSET for its localization at the Golgi, we show that it localizes properly, it is stable, and can rescue the lysosomal enzyme secretion phenotype, even in GRASP55-deficient cells (**new Fig. 8**). These data further support a model wherein GRASP55 controls GNPTAB through LYSET.
- Following the reviewers' suggestion, we have removed the—more exploratory—data on the COG complex from the revised manuscript.
- We have rearranged the figures to improve clarity and incorporate the new data.
- We have expanded our Discussion to include the new findings and to acknowledge the remaining limitations of our study.
- We have adapted our working model (new Fig. 9) accordingly to reflect the new findings better.

We believe that these changes/additions make the revised manuscript very solid and we hope that the Reviewers will find our revised manuscript suitable for publication in *EMBO Reports*.

Regarding the Reviewers' concerns about the novelty of our work, we wish to note that our manuscript was publicly available as a preprint (DOI: 10.1101/2024.12.10.627846v1) since December 2024, prior to the publication of the related study by Akaaboune et al. in *MBoC*, and we were offered scooping protection by the *EMBO J* accordingly. Furthermore, we hope the Reviewers agree that our study includes a more extensive phenotypic characterization of GRASP55 knockout (KO) and reconstituted cell line models, multiple independent lines of functional validation, integration with mTORC1 signaling, and—now—a deeper mechanistic understanding of how GRASP55 controls GNPTAB and lysosomal enzyme sorting, which collectively offer a more detailed and in-depth analysis compared to the competing work.

Please see below for our responses to the specific Reviewer comments (in blue).

Referee #1

Lysosomal hydrolases are critical for cellular function. They are sorted to the Golgi by means of a mannose 6-phosphate (M6P) signal that is attached to their glycans in the Golgi apparatus and then recognized by M6P receptors (MPRs) that direct the hydrolases to late endosomes for delivery to the lysosome. GRASP55 is a PDZ-domain containing Golgi protein that is recruited to the cytosolic surface of Golgi membranes by an N-terminal lipid anchor. It has been reported to have several possible functions including organising the structure of the Golgi, helping direct specific proteins through the Golgi, and even a role in unconventional protein secretion that bypasses the Golgi.

The authors have previously reported an examination of the role of GRASP55 in unconventional protein secretion which included determining the secretome of cells lacking GRASP55 (Nuschel et al. 2021). In this study they report that reanalyzing this secretome data revealed that removal of GRASP55 resulted in substantial secretion of lysosomal hydrolases from the cell. They confirm this using a variety of assays, including blotting for individual enzymes, following the traffic of an exogenously expressed enzyme, measuring lysosomal hydrolase activity inside cells, and examining the location of mTOR complex which they have previously shown to be released from the surface of lysosomes when hydrolase sorting is defective. All of this extensively confirms that lysosomal hydrolase are released from GRASP55 KO cells, and the data is carefully quantified and clearly presented.

To investigate mechanism, they follow up the fact that the GNPTAB enzyme that adds the M6P group is also released from cells lacking GRASP55. They express a tagged version of this Golgi membrane protein and find that when GRASP55 is missing the protein is partly mislocalized to the ER and degraded. They present immunoprecipitation data to argue that GRASP55 binds directly to GNPTAB. Finally, they use proximity biotinylation to determine a GRASP55 "proximome" and find amongst various Golgi proteins subunits of the COG complex that has a role in vesicle transport in the Golgi and is known to be required for efficient glycan modification in the Golgi. They present evidence that GRASP55 also binds to COG, and that cells lacking subunits of the COG complex show elevated secretion of lysosomal hydrolases. There is quite a lot of interest in the mechanism by which lysosomal hydrolases are modified and sorted to the vacuole. In addition, the roles of GRASP55, and its paralog GRASP65 (which does not have a role here), are unresolved and so clear data on their function would be valuable. The first five figures of the paper are of a high standard, but all make essentially the same point albeit with different assays. The more mechanistic aspects of the work are less

robust, and there is a slight issue of novelty which could have been better handled. These points are explained fully below, but taken together they mean that the work would require substantial improvement and revision before publication in a general interest journal like the EMBO Journal.

We thank the Reviewer for their overall positive assessment of our manuscript, and for finding our data on the characterization of the GRASP55 loss-of-function models clear, solid and conclusive. Please see below for our responses to the specific comments regarding novelty and on expanding the mechanistic depth of our study.

A) In October 2024 a paper was posted in bioRxiv reporting that deletion of GRASP55 results in secretion of lysosomal hydrolases correlating with a perturbation in levels of GNPTAB, and this study has now appeared in print in Molecular Biology of the Cell (Akaaboune et al.; PubMed ID 39841559). Of course, it is likely that the work described in this submission was started ahead of the bioRxiv post, and the characterization here is certainly more extensive, but the authors really should have added a note to the effect that another study has reported similar conclusions whilst they were preparing theirs for submission.

We thank the Reviewer for raising this point and for acknowledging the more extensive characterization presented in our study.

As stated in the original version of our manuscript, this work was initiated based on a reanalysis of our GRASP55-dependent secretome data, originally published in our 2021 paper (Nüchel et al. 2021 Mol Cell, PMID: 34245671). Building on these findings, we subsequently undertook a comprehensive functional characterization of GRASP55 knockout and reconstituted cell line models, including analyses of lysosomal enzyme trafficking, lysosomal function, mTORC1 signaling, and GNPTAB localization and stability. Thus, as the Reviewer correctly surmised, this study was initiated several years prior to the appearance of the related MBoC paper; or the associated preprint.

Importantly, our revised manuscript now provides substantial new mechanistic insight that extends well beyond the initial observation of reduced GNPTAB levels. Specifically, our new data demonstrate that GRASP55 is required for Golgi recruitment of GOLPH3 and LYSET, which in turn stabilize GNPTAB at the cis-Golgi (**new Figs. 7 and 8**). These findings define a previously unrecognized GRASP55-GOLPH3-LYSET pathway linking Golgi organization to

M6P-dependent lysosomal enzyme sorting, thereby substantially strengthening the mechanistic framework and distinguishing our study from Akaaboune et al.

We also note that our manuscript was made publicly available as a preprint in December 2024 (DOI: 10.1101/2024.12.10.627846v1), prior to submission to *EMBO J*, and that we were offered scooping protection in accordance with the journal's policy, which has been extended upon transfer to *EMBO Reports*. We apologize for not explicitly citing Akaaboune et al. in the original submission, as the work had not yet appeared in print at that time, and we have now included a reference to the Akaaboune et al. paper in the revised version of our manuscript.

We hope this addresses the Reviewer's concern and clarifies both the timeline and the distinct contributions of our work.

B) The various experiments confirming that in the GRASP55 KO cells lysosomal hydrolases are secreted rather than intracellular are of a high standard and convincing. However, the data to support the proposed mechanism behind this effect needs more depth and better assays. The authors use immunoprecipitation of tagged GNPTAB from whole cell lysates to argue that GNPTAB binds to GRASP55. However, such an experiment does not demonstrate direct binding, and moreover the immunoprecipitation seems rather odd with myc-tagged GNPTAB not being highly enriched in the anti-myc IP, and the amount of GRASP55 that is coprecipitated being rather low (Figure 6E). GRASP55-GFP was analysed in the OpenCell large scale PPI screen where it was tagged in the genome and GNPTAB was not among the proteins that co-precipitated with it (PubMed ID 35271311). In addition, it is known that GNPTAB forms a complex with a small membrane protein called LYSET which binds a COPI interactor called GOLPH3, and so even if the co-ip is correct then the interaction could be directly with GNPTAB or indirectly through LYSET, GOLPH3 or even some other factor. Thus, this binding data would be more convincing if the cytoplasmic domains of these various proteins were tested with recombinant GRASP55 to identify and define a direct interaction.

We thank the Reviewer for the careful evaluation of our mechanistic data and for the constructive suggestions to strengthen this aspect of the study. We fully agree that the original manuscript did not provide sufficient depth to support the proposed mechanism linking GRASP55 to GNPTAB, and we have therefore substantially revised this part of the work.

First, in response to the Reviewer's concerns regarding the GNPTAB-GRASP55 co-immunoprecipitation experiment, we wish to clarify that we do not interpret these data as evidence for a direct interaction between the two proteins. Indeed, as the Reviewer correctly points out, co-immunoprecipitation from whole-cell lysates cannot distinguish between direct and indirect associations, and the relatively low recovery of GRASP55 in this assay is consistent with a transient and/or indirect interaction. To avoid overinterpretation, we have revised the text to explicitly state that the interaction detected by co-IP is likely indirect and does not demonstrate direct binding.

Second, prompted by the Reviewer's comment and informed by the established literature on GNPTAB regulation, we have now extended our mechanistic analysis to include the previously described GOLPH3-LYSET machinery. As shown in the revised manuscript (**new Fig. 7**), we find that loss of GRASP55 results not only in mislocalization of GNPTAB, but also in the displacement of GOLPH3 and LYSET from the Golgi. Moreover, we detect interactions between GRASP55 and each component of the GOLPH3-LYSET-GNPTAB complex, with the GRASP55-GOLPH3 interaction remaining intact upon LYSET depletion. This epistasis suggests that GOLPH3 is the primary GRASP55-dependent factor, placing GRASP55 upstream of the GOLPH3-LYSET pathway that has been shown to control GNPTAB localization and stability.

Consistent with this model, we further show that LYSET undergoes lysosome-dependent degradation in GRASP55-deficient cells, in agreement with recent findings identifying GOLPH3 as a key regulator of LYSET and GNPTAB (Brauer et al. 2024 *EMBO J*). Importantly, using a GNPTAB mutant that was previously shown to localize to the Golgi independently of LYSET, we demonstrate that this mutant remains stable and functionally rescues lysosomal enzyme sorting even in the absence of GRASP55 (**new Fig. 8**). Together, these data strongly support an indirect mechanism whereby GRASP55 controls GNPTAB through GOLPH3 and LYSET, rather than via direct binding.

Finally, we fully agree with the Reviewer that identifying direct interactions using recombinant proteins and defined domains would be an important future direction. However, given the size, topology, and post-translational regulation of these proteins, as well as the substantial new in vivo mechanistic framework now provided, we believe that such biochemical reconstitution studies fall beyond the scope of the current work. We have therefore explicitly acknowledged

this limitation in the Discussion and now clearly state that the directness and molecular interfaces of the GRASP55-GOLPH3-LYSET interactions remain to be determined.

We hope that the additional data, revised interpretation, and clearer positioning of GRASP55 upstream of the GOLPH3-LYSET machinery adequately address the Reviewer's concerns and provide a robust mechanistic explanation for how GRASP55 controls lysosomal enzyme sorting.

C) The authors use an APEX fusion and proximity biotinylation to identify proteins near to GRASP55 and then test the COG complex which they suggest also co-immunoprecipitates. Such proximity biotinylation does not measure an "interactome" as the authors state, especially as APEX has a large radius of action compared to BirA, but it will simply label proteins that happen to be in the same location in the cell. Many Golgi proteins are enriched as would be expected, and it is not clear why GRASP55 should interact with the COG complex, especially as this interaction has not been previously observed in large scale PPI screens that have found known GRASP55 interactors. The authors show lysosomal hydrolase missorting in cells lacking COG subunits, but it is known that such lines have strong defects in glycan processing in the Golgi due to defects in vesicle recycling and given that M6P is added to glycans in the Golgi then the effect on hydrolases could be a consequence of a general defect in glycans rather than a specific effect via GRASP55 or GNPTAB. Indeed, the general perturbation of glycan processing in the COG subunit KO lines can be seen in the increased mobility of some of the hydrolases tested here (Figure 7F).

We appreciate the Rewiewer's concern about the GRASP55-COG interaction data being preliminary and the functional effects in the COG KO lines being presumably indirect. Following the Reviewer's suggestion and following discussions with the handling editor, we have therefore decided to remove the APEX/COG data from the revised manuscript. This has allowed us to sharpen the focus of the manuscript on the GRASP55-GOLPH3-LYSET-GNPTAB pathway, for which we now provide substantially strengthened mechanistic evidence.

We believe that this revision improves the clarity and conceptual coherence of the manuscript and fully addresses the Reviewer's concerns.

D) The authors should have cited and briefly discussed the fact that mice lacking GRASP55 have male fertility defects but the females are viable and fertile which suggests that GRASP55 has a rather limited role that it is unique and instead there is greater redundancy with GRASP65 (PubMed ID 28617811).

We agree with the Reviewer that there is some redundancy between GRASP55 and GRASP65 at the organismal level; or in cells when it comes to their role in Golgi morphology and structure (which is the most relevant function for spermatogenesis). However, as we demonstrated previously (Nüchel et al. 2021 *Mol Cell*, PMID: 34245671), this is not the case in the regulation of unconventional protein secretion (UPS), where GRASP65 is not under the regulation of nutrient/stress/mTORC1 signaling and also not involved in controlling UPS. The same is true for lysosomal enzyme sorting, as all data presented in this manuscript clearly show: while loss of GRASP55 causes milocalization of GOLPH3-LYSET, destabilization of LYSET and GNPTAB, lysosomal enzyme missorting, lysosomal dysfunction, and blunts lysosomal mTORC1 signaling, GRASP65 KO cells show none of the above phenotypes.

We therefore believe that the organismal phenotype of GRASP55-deficient mice, while interesting, is not directly informative for the Golgi-based molecular mechanism addressed in this study. To keep the Discussion concise and focused on the pathways and processes directly relevant to lysosomal enzyme sorting and Golgi–lysosome communication, we have chosen not to expand this aspect further in the manuscript.

Overall, the data in Figures 1-6 (without Figure 6E) would make a good quality descriptive paper in a specialized journal on the requirement for GRASP55 for efficient sorting of lysosomal hydrolases. However, if the authors wish to add mechanistic insight and hence raise the work to the standard expected for a broad interest journal such as the EMBO Journal then substantial new data would be required to make robust mechanistic conclusions.

We thank again the Reviewer for the careful evaluation of our work and for the constructive suggestions regarding both the scope and mechanistic depth of the manuscript. We appreciate his/her assessment that the core findings represent a solid and high-quality body of work.

In response to these comments, we have taked a two-fold approach: (i) we have substantially expanded our mechanistic analyses in the revised manuscript, providing new data that

position GRASP55 upstream of the GOLPH3-LYSET-GNPTAB machinery and clarify how GRASP55 controls lysosomal enzyme sorting and lysosomal function; (ii) in line with the Reviewer's assessment, we have accepted the handling editor's offer to transfer the manuscript to *EMBO Reports*, which we believe is an appropriate venue, as its scope explicitly encourages the publication of timely and robust biological observations that offer new conceptual insight, even when the precise molecular mechanisms are not yet fully defined.

Referee #2

This manuscript describes a role for the Golgi resident protein GRASP55 in the correct trafficking of lysosomal enzymes to the lysosome. GRASP55 functions in this process by maintaining the enzyme that adds the key mannose 6-phosphate tag to the enzymes, GNPTAB, in the Golgi. In the absence of GRASP55, GNPTAB is localized to the ER instead of the Golgi, and the lysosomal enzymes are missorted and secreted upon Golgi exit instead of being delivered to the lysosomes. This results in swollen dysfunctional lysosomes that have defective mTOR signaling. Interaction data show that GRASP55 can bind to GNPTAB, and that it can also interact with the Golgi vesicle tethering complex COG.

This is an interesting paper that is very nicely done. The data are clear and support the authors' conclusions. However, while I liked this study, I think it suffers from a lack of mechanistic information, and the novelty of the major findings is also diminished by previous work. I elaborate on the points below.

We thank the Reviewer for finding our manuscript interesting and our data clear and conclusive. Please see below for our responses to the specific comments regarding novelty and the mechanistic depth of our study.

Major comments:

1) While it is shown that GRASP55 can bind to GNPTAB and to the COG complex, how it maintains GNPTAB in the Golgi remains unclear. Does it bind to GNPTAB to anchor or retain it in the Golgi? Does it help GNPTAB get sorted into retrograde COPI vesicles to retain it there? Does it promote ER-Golgi traffic of GNPTAB or prevent its recycling to the ER? It remains unclear. Recent papers have shown that LYSET also keeps GNPTAB in the Golgi, acting through the GOLPH proteins for sorting into COPI vesicles. How does GRASP55 function compare to that of LYSET? The interaction with COG might suggest a role in vesicle traffic but it is not explored.

We agree that the original version of the manuscript did not sufficiently clarify how GRASP55 maintains GNPTAB at the Golgi, and we have therefore substantially revised this aspect of the study.

In the revised manuscript, we show that GRASP55 does not act by directly anchoring GNPTAB at the Golgi. Instead, our new data place GRASP55 upstream of the previously

described GOLPH3-LYSET pathway that controls GNPTAB localization via COPI-dependent retention. Specifically, we demonstrate that loss of GRASP55 results in mislocalization of GOLPH3 and LYSET from the Golgi, leading to destabilization and mislocalization of GNPTAB (**new Fig. 7**). Conversely, a GNPTAB mutant that localizes to the Golgi independently of LYSET remains properly localized and functional in GRASP55-deficient cells (**new Fig. 8**). These findings support a model in which GRASP55 maintains GNPTAB indirectly, by ensuring Golgi localization of GOLPH3 and LYSET rather than by directly regulating GNPTAB trafficking.

Based on this revised framework, GRASP55 functions upstream of LYSET, rather than in parallel to it, and its role is to maintain the Golgi residence of the GOLPH3-LYSET machinery required for COPI-mediated retention of GNPTAB. While our data do not resolve whether GRASP55 directly influences COPI vesicle formation or ER-Golgi recycling, they establish its position in the pathway and provide a mechanistic link between GRASP55 and GNPTAB localization.

Finally, following reviewer feedback and discussions with the handling editor, we have removed the COG-related data from the revised manuscript, as these experiments were preliminary and did not contribute directly to the clarified mechanism.

2) The experiments with COG do not inform us of GRASP55 function. Although there is some degree of phenotypic overlap in the GRASP55 and COG KO cells, it is likely that the COG is acting independently considering that it is required for proper localization of numerous Golgi enzymes, resulting in major defects in glycan addition and processing, as opposed to just the GNPTAB enzyme. Also, it was shown recently that stable KO of COG results in a myriad of trafficking phenotypes, many likely indirect, greatly complicating the interpretation of the experiments here.

We appreciate the Rewiewer's concern about the GRASP55-COG interaction data being preliminary and the functional effects in the COG KO lines being presumably indirect. Following the Reviewer's suggestion and upon discussions with the handling editor, we have therefore decided to remove the COG-related data from the revised manuscript. This has allowed us to sharpen the focus of the manuscript on the GRASP55-GOLPH3-LYSET-GNPTAB pathway, for which we now provide substantially strengthened mechanistic evidence.

3) In terms of novelty, previous studies have shown that GRASP55 retains Golgi enzymes involved in sphingolipid synthesis in the Golgi, but even more relevant for this study is a recent paper showing that GRASP55 is required for GNPTAB stability and correct lysosomal hydrolase sorting. The potential additional novelty in this paper might therefore come from the analysis of mTOR signaling, but it was also shown recently by the same authors that lysosomal dysfunction affects this process. My feeling is that without additional exploration of GRASP55 mechanism regarding GNPTAB function, or the regulation of GRASP55 by mTOR in the context of lysosomal sorting, the paper lacks some novelty.

As stated in the original version of our manuscript, this work was initiated based on a reanalysis of our GRASP55-dependent secretome data, originally published in our 2021 paper (Nüchel et al. 2021 Mol Cell, PMID: 34245671). Building on these findings, we subsequently undertook a comprehensive functional characterization of GRASP55 knockout and reconstituted cell line models, including analyses of lysosomal enzyme trafficking, lysosomal function, mTORC1 signaling, and GNPTAB localization and stability. Thus, as the Reviewer correctly surmised, this study was initiated several years prior to the appearance of the related MBoC paper; or the associated preprint.

Importantly, our revised manuscript now provides substantial new mechanistic insight that extends well beyond the initial observation of reduced GNPTAB levels. Specifically, our new data demonstrate that GRASP55 is required for Golgi recruitment of GOLPH3 and LYSET, which in turn stabilize GNPTAB at the cis-Golgi (**new Figs. 7 and 8**). These findings define a previously unrecognized GRASP55-GOLPH3-LYSET pathway linking Golgi organization to M6P-dependent lysosomal enzyme sorting, thereby substantially strengthening the mechanistic framework and distinguishing our study from Akaaboune et al.

We also note that our manuscript was made publicly available as a preprint in December 2024 (DOI: 10.1101/2024.12.10.627846v1), prior to submission to *EMBO J*, and that we were offered scooping protection in accordance with the journal's policy, which has been extended upon transfer to *EMBO Reports*. We hope this addresses the Reviewer's concern and clarifies both the timeline and the distinct contributions of our work.

Additional comments:

1) In Fig S1 (the RUSH experiment) the expression of PSAP looks to be higher in the KO cells. The apparent increased secretion is a little misleading considering the different expression level. The rate of exit from the cells looks similar in WT and KO.

We kindly disagree with the Reviewer's point, as the secretion of PSAP-SBP in the RUSH experiments is clearly enhanced in the GRASP55 KO cells, compared to controls. These differences can be appreciated better in the lower exposure of the PSAB blots. Concerning PSAP-SBP expression levels, we have not observed consistent differences between the two genotypes. One posibility for the visual impression that PSAP-SBP levels may be slightly higher in GRASP55 KO cells is that PSAP is proteolytically cleaved in WT cells, therefore the unprocessed pre-form appears less abundant.

Furthermore, in this figure, we complement the immunoblotting data (**Fig. EV1B**) with microscopy images (**Fig. EV1A**) showing the different kinetics of PSAP which, unlike in the WT control cells, it does not end up in vesicular structures in GRASP55 KO cells even following prolonged biotin addition. Together with the experiments looking at lysosomal enzyme secretion (**Fig. 1**), activity in cells and in media (**Fig. 3** and **Fig. EV2**), and colocalization with a lysosomal marker (**Fig. 2**), our RUSH data indicate that lysosomal enzymes are missorted in GRASP55 KO cells.

2) In Fig 2, the lysosomal hydrolases look to be in spotty structures in the GRASP55 KO (CTSB, CTSD, PSAP). If these structures are not lysosomes, what are they? If the enzymes are missorted it would be good to know where they go.

The Reviewer raises a very interesting point. Although the nature of these vesicles is unclear to us, most of these puncta do not stain positive for the lysosomal marker LAMP2, therefore we conclude these are not lysosomes. This is also indicated by the quantitative colocalization analysis that shows strongly decreased overlap between the lysosomal hydrolase and lysosomal marker signals in the GRASP55 KO cells. One posibility is that these are Golgi-derived vesicles that deliver the missorted lysosomal enzymes to the extracellular space, however additional work (e.g., co-staining with different Rab protein markers) that is beyond the scope of this manuscript, would be necessary to identify the secretory pathways that are involved in this process.

3) In Fig 6, the altered processing of GNPTAB is shown. This could be a consequence of its altered localization, but equally it could be due to altered localization of the S1P protease, which cycles between the ER and Golgi. Can the authors localize S1P to exclude a defect in its own trafficking?

We agree that altered GNPTAB processing could, in principle, result from defects in the localization or trafficking of the S1P protease. However, our data argue against this possibility. Specifically, we show that the $GNPTAB^{QELL}$ mutant, which localizes to the Golgi independently of LYSET (and independently of GRASP55), remains correctly processed and functionally active in GRASP55-deficient cells (**new Fig. 8**). This occurs despite the absence of GRASP55, indicating that S1P-mediated cleavage is intact under these conditions. Thus, the defective processing of wild-type GNPTAB in GRASP55 KO cells is best explained by its mislocalization away from the Golgi rather than by altered S1P trafficking or activity.

4) Text, line 162, states "Focusing on the proteins whose secretion is blunted in GRASP55 KO cells, we observed a robust enrichment of extracellular matrix (ECM)- and cell adhesion-related factors, indicating that GRASP55 controls such cellular processes through the regulation of UPS". The first part of this sentence is confusing and oxymoronic- secretion that is blunted but enriched? The second part is not justified- ECM is mainly secreted by conventional secretion so if ECM proteins are altered is not an indication of UPS regulation.

We apologize for the confusion caused by this sentence, and we are happy to clarify. In fact, <u>this sentence refers to our previous, published work</u> that is described in the Nüchel et al. 2021 *Mol Cell* paper.

Regarding the first part, what we mean is that there is an enrichment of ECM- and cell adhesion-related factors (and of the associated GO terms) when looking at the group of proteins whose secretion is blunted in the GRASP55 KO cells. We hope this is clearer now.

Regarding the second part, we would like to kindly point out that GRASP55 is well known as a master regulator of unconventional protein secretion (UPS), a process that is activated <u>in response to starvation</u>. The Reviewer is correct that most ECM proteins are secreted via conventional secretory routes, however here we refer to the <u>reshaping of the ECM that takes place upon starvation and/or stress</u>, which involves the GRASP55/UPS-dependent secretion of <u>distinct</u> and <u>different</u> ECM and ECM-modifying proteins.

5) Text, line 318, states "reduced expression of Myc-tagged GNPTAB". It only indicates reduced abundance not expression per se.

We thank the Reviewer for pointing this out, and we rephrased this part to *“reduced protein levels of Myc-tagged GNPTAB”*.

6) Text, line 473, states ER to Golgi transport of enzymes is affected by GRASP55 KO. The RUSH data in Fig S1 suggest this step is not affected by GRASP55 KO.

We have removed this statement in the revised manuscript to align the text with the updated working model and mechanistic framework. Specifically, the revised model focuses on GRASP55’s role upstream of the GOLPH3-LYSET-GNPTAB pathway at the Golgi, rather than on ER-to-Golgi transport of GNPTAB. This change is also consistent with the removal of the COG-related data, which were part of the original exploratory analysis.

Referee #3

Nuchel et al. report a novel function for GRASP55 in lysosomal enzyme trafficking. Through reanalysis of their previously published secretome of GRASP55 KO cells, the authors discover increased secretion of various lysosomal enzymes in their immature form. By further investigating the phenotypes of GRASP55 KO cells, the authors demonstrate decreased cellular levels and activity of lysosomal enzymes, which is accompanied by accumulation of enlarged lysosomes and perturbed mTORC1 signaling. Mechanistically, the authors link these phenotypes to defects in the trafficking and localization of GNPTAB in the absence of GRASP55. This study identifies interesting and striking phenotype of GRASP55 KO cells, which is timely in light of the emerging realization that Golgi processes are critical for lysosomal biogenesis and hence function. The data are presented in well-structured figures, although the manuscript is somewhat digressive and the authors tend to overinterpret their results. A large part of the manuscript is dedicated to documenting the lysosomal defects of GRASP55 KO, but the mechanistic part concerning the role of GRASP55 in GNPTAB trafficking and localization is immature and the proposed mechanism speculative. While this manuscript is of potential interest, substantial additional experiments are required to achieve a mechanistic depth appropriate for publication in the EMBO Journal.

We would like to thank the Reviewer for their positive comments on our manuscript, and for finding our study interesting and timely. Please see below for our responses to the specific comments regarding the mechanistic depth of our study and the interpretation of our results.

Major points:

1. The authors report decreased protein levels and ER relocalization of ectopically expressed GNPTAB in GRASP55 KO cells (Fig. 5), but how GRASP55 controls GNPTAB localization and stability remains a mystery. Does GNPTAB localize to the ER in GRASP55 KO cells due to decreased anterograde trafficking to or retrograde trafficking from the Golgi? How, mechanistically, does the Golgi-resident GRASP55 affect this process? Time course studies of GNPTAB trafficking and processing as well as manipulation of the underlying trafficking pathways might help resolve these questions. The authors further suggest that GRASP55 controls intra-Golgi trafficking of GNPTAB (lines 362-364) - are there any data supporting this claim?

Prompted by the Reviewer's suggestion, we have now expanded the mechanistic analysis of how GRASP55 controls GNPTAB localization and stability. In the revised manuscript, we show that GRASP55 acts indirectly by maintaining the Golgi residence of the GOLPH3-LYSET machinery. Loss of GRASP55 causes mislocalization of GOLPH3 and LYSET from the Golgi, which in turn destabilizes GNPTAB and leads to its redistribution away from the Golgi (**new Fig. 7**). Importantly, a $GNPTAB^{QELL}$ mutant, that localizes to the Golgi independently of LYSET, remains properly localized and stable, and can rescue lysosomal enzyme sorting even in GRASP55-deficient cells (**new Fig. 8**). These findings indicate that GRASP55 functions upstream of GOLPH3-LYSET rather than acting directly on GNPTAB.

Furthermore, to better align with the updated mechanistic model, we have also removed the previous statement regarding the control of intra-Golgi trafficking of GNPTAB by GRASP55-COG.

2. Significant progress has been made over the past several years in understanding how correct Golgi localization of GNPTAB is controlled, including demonstrations of GNPTAB Golgi-retention as a COPI-dependent process mediated by GOLPH3/GOLPH3L and LYSET and membrane stabilization of GNPTAB by LYSET (PMID: 36074821, 36074822, 36096887, 39587297, 39937677). It is curious that the authors do not, at least to some extent, attempt to place their findings into this mechanistic framework. Does GRASP55 KO affect LYSET localization or levels? How is GRASP55-mediated Golgi trafficking / retention of GNPTAB related to the COPI/GOLPH3-dependent process?

We thank the Reviewer for the valuable suggestions. As mentioned above we have now integrated our findings into the broader mechanistic framework established by excellent recent work on GNPTAB retention at the Golgi. More specifically, we now provide data showing that GRASP55 is required for proper Golgi localization of both GOLPH3 and LYSET, explaining how it indirectly maintains GNPTAB at the Golgi and supports COPI-dependent retention (**new Figs. 7 and 8**).

3. Experiments concerning GNPTAB are based on ectopic overexpression, which does not necessarily reflect the localization, abundance and processing of the endogenous protein or recapitulate the stoichiometry of the heteromeric phosphotransferase complex. Indeed, over-expressed GNPTAB-myc leads to much higher levels of the precursor protein than the processed beta-subunit, which in addition is degraded by the proteasome to a similar extent

in WT and GRASP55 KO cells (Fig. 6F). It would be important to demonstrate key results at endogenous protein level.

We fully acknowledge the limitations associated with the use of exogenously expressed GNPTAB. We have made multiple efforts to detect the endogenous protein, including testing four (4) different commercially available antibodies; however, none yielded a specific signal in either immunoblotting or microscopy, as determined using GNPTAB KO cells as a negative control. Unfortunately, we were also denied access to a previously validated anti-GNPTAB antibody (used in some of the recently published LYSET papers) due to a prior conflict between the generating laboratory and one of our co-authors.

Despite these constraints, we would like to point out that, in our experiments, GNPTAB-myc localized properly to the Golgi in control cells (**Figs. 6A and 8A**), supporting its physiological relevance. Finally, regarding Fig. 6F (now **Fig. 6E** in the revised manuscript) and the Reviewer's statement that *"GNPTAB-myc… is degraded by the proteasome to a similar extent in WT and GRASP55 KO cells"*, we would like to clarify that full-length GNPTAB is actually not degraded in control cells (see lanes 1 vs. 3). Instead, we find that GNPTAB levels are lower in GRASP55 KO cells compared to controls (see lanes 1 vs. 4), with partial stabilization by MG132 treatment (see lanes 4 vs. 6), indicating proteasome-mediated degradation specifically in the absence of GRASP55.

4. Some of the phenotypes in GRASP55 KO cells are inconsistent, especially with relation to GNPTAB (supposed to be similar) and GRASP65 (supposed to be different). In particular, a reduction of M6P levels is hard to discern (Fig. 1D, 7F) and the levels are indistinguishable from of those in GRASP65 cells. This contrasts to the hypersecretion of enzymes in GRASP55 KO but not GRASP65 KO cells, raising the question whether a reduction of M6P levels is responsible for this phenotype.

We respectfully disagree with the Reviewer's assessment of our data shown in **Fig. 1D** and former Fig. 7F (this was now removed together with all COG-related data, as mentioned above). To explain the presentation of our data in these experiments better, several bands are present in the anti-M6P blots, many of which are seemingly non-specific, as they do not diappear in GNPTAB KO cells. In fact, in both panels, we had used arrowheads to clearly mark the bands that disappear in the GNPTAB KO samples compared to WT controls, indicating they represent M6P-modified proteins. Importantly, the intensity of these bands

decreases in the samples from GRASP55 KO, but not GRASP65 KO cells. Finaly, please note that the weaker effect in lowering M6P protein modification observed in GRASP55 KO cells, compared to GNPTAB KOs, is also consistent with the weaker effects in lysosomal enzyme secretion, lysosomal bloating and nearly every single phenotype tested in our study. This is, actually, not unexpected as GNPTAB is completely absent in the respective KOs, while some expression of GNPTAB that partially localizes to the Golgi is detected in GRASP55 KOs.

5. The entire manuscript appears to be based on a single cell line and a single knockout clone for each of the genes under investigation. To demonstrate robustness of the findings, one would expect key findings to be validated in multiple cell lines / with multiple sgRNAs.

As described in our previous paper on the role of the mTORC1-GRASP55 signaling axis in unconventional secretion (Nüchel et al. 2021 *Mol Cell*): *"For the quantitative analysis of the GRASP55-dependent secretome, SILAC experiments were performed with four independent biological replicates, including two independent GRASP55 KO clones and swapping labeling of WT and KO cells with heavy and medium isotopes (or vice versa) to control for labeling bias"*. Because of the high reproducibility in the data obtained from the two independent clones, merged data were shown in the corresponding volcano plots (as the one shown in **Fig. 1B**).

Importantly, we also show that re-expression of Myc-tagged GRASP55 in the GRASP55 KO fibroblasts is sufficient to fully rescue the lysosomal bloating, the lysosomal enzyme secretion, the LYSET stability, and the GOLPH3-LYSET localization phenotypes (**Fig. 4C-E**, **Fig. EV3**, and **new Fig. 7G-I**), showing that the effects are indeed due to loss of GRASP55 expression in our KOs. Finally, similar phenotypes were independently reported in a related study using GRASP55 KO HeLa and HEK293T cell lines (Akaaboune et al. 2025 *MBoC*, PMID: 39464054), further confirming the validity of the key findings described in our manuscript.

6. The manuscript concludes with the finding that GRASP55 interacts with the COG complex, whose knockout also leads to lysosomal enzyme secretion and lysosomal bloating (Fig. 7). These results, while potentially interesting, appear like an afterthought - except that the lysosomal defects of COG KO cells are much stronger than those of GRASP55 KO cells. This suggests that the COG complex either regulates enzyme trafficking through GRASP55-dependent and independent pathways (no data for GNPTAB are provided in this context) or

has a pleiotropic effect on Golgi trafficking / function. It would be helpful if the authors could provide some perspective on this.

The Reviewer is correct about the COG proteins having additional roles beyond the regulation of lysosomal enzyme sorting at the Golgi, the most relevant of which for this study being their involvement in glycan addition and processing. Therefore, following the Reviewer's suggestion and upon discussions with the handling editor, we have therefore decided to remove the COG-related data from the revised manuscript. This has allowed us to sharpen the focus of the manuscript on the GRASP55-GOLPH3-LYSET-GNPTAB pathway, for which we now provide substantially strengthened mechanistic evidence.

7. The text would benefit from revision to more accurately reflect and discuss results. Examples are given below:

The title states that GRASP55 safeguards lysosomal function. I am not aware of the term safeguard being used when referring to the M6P pathway, and it would help if the authors could clarify its meaning.

We would like to clarify that the term 'safeguard' used in our title does not refer to any particular pathway. According to various dictionaries, to safeguard means to protect someone or something or to prevent something undesirable; to protect from harm or damage with an appropriate measure; to shield; to preserve. In our study, we show that the presence of GRASP55 is necessary for proper lysosomal enzyme sorting, lysosome function, lysosomal mTORC1 signaling, localization of key enzymes, etc., while its absence causes the dysregulation of all aforementioned cellular functions. Based on the above, we propose that GRASP55 safeguards lysosomal function.

In the abstract, the authors refer to GRASP55 as a key regulator of lysosome function - what is being regulated how and in response to what input remains elusive.

In our revised manuscript, we reveal a large part of the molecular mechanism via which GRASP55 controls GNPTAB localization at the Golgi, acting through the regulation of GOLPH3 and LYSET localization and/or stability. Although we have not yet identified the upstream signals that impinge on GRASP55 to modify its function, or the molecular principles of how it stabilizes GOLPH3 at the Golgi, our data clearly show that GRASP55 is necessary

for proper lysosomal enzyme sorting and lysosome function via the GOLPH3-LYSET-GNPTAB axis, therefore it is reasonable to state that it regulates these processes, even if this refers to indirect regulation.

Line 145, 291: "cytoplasmic targets, like 4E-BP1". No cytoplasmic targets other than 4E-BP1 are examined.

We have rephrased this sentence in the revised version of our manuscript which now reads: “towards a cytoplasmic target such as 4E-BP1”. Of note, we recently showed that the same mode of regulation is true for additional cytoplasmic mTORC1 substrates, at least for GNPTAB KO cell lines (Fernandes et al. 2024 *Nat Cell Biol*, PMID: 39385049).

The discussion has a lengthy paragraph termed "GRASP55 lies at the center of mTORC1 signaling", which is very strange considering that the manuscript reports perturbed mTORC1 signaling as an indirect consequence due to lysosomal dysfunction resulting from Golgi trafficking defects in GRASP55 KO cells. Please clarify the precise role of GRASP55 (i.e. not its absence) in mTORC1 signaling; alternatively consider removing this paragraph and focus on key points that are relevant to the present study.

To clarify, we never claim that GRASP55 plays a <u>direct</u> role in the regulation of mTORC1 activity. However, because in its absence lysosomal enzyme sorting is perturbed, its presence is necessary for proper lysosome function and therefore lysosomal mTORC1 activity. A similar example is the lysosomal v-ATPase that is generally considered, by most people in the mTOR field, a central component in lysosomal mTORC1 signaling: while its inhibition is necessary for the phosphorylation of lysosomal mTORC1 substrates and for the acute reactivation of mTORC1 following starvation, this effect is only due to its role in lysosomal acidification, and thus lysosomal protease activity and amino acid efflux from lysosomes; and not because it directly controls mTORC1 activity.

Throughout the study, the authors claim to provide new insights into the molecular basis of LSD-related pathologies and suggest GRASP55 to act as a new LSD susceptibility gene. Has GRASP55 been associated with genetic diseases and do the patients present LSD-like phenotypes? This would indeed be very interesting and relevant. If such evidence is absent, then this could be an interesting but speculative idea that should be discussed in a more balanced way and removed from the abstract.

We would like to clarify that we refer to the potential impact of our work for improving our understanding on the molecular etiology of certain lysosomal enzyme trafficking disorders. No disease-linked GRASP55 variants have been identified so far, however one should consider that these are ultra-rare disorders for each individual LSD-linked gene, therefore it would not be expected to easily detect GRASP55 mutations at high prevalence in human populations. In fact, in collaboration with an international network of physicians, we are currently actively searching for potential disease-relevant GRASP55 variants in patients in the LSD spectrum.

Minor points:

1. Review the references cited in lines 50 - 57. Are these really the most suitable to discuss the work over the last 15 years characterizing the lysosomal regulation of mTORC1 and TSC?

Although we try to be as inclusive as possible when citing previous work, we apologize for any oversight in referring to additional relevant studies in this part of our manuscript, and we would welcome specific suggestions by the Reviewer.

2. Since the authors previously identified GRASP55 to be phosphorylated and regulated by mTORC1, it would be very interesting to know whether this affects GNPTAB localization and lysosomal enzyme trafficking, which would point to a regulatory role of GRASP55 in this process.

This is a great point and we are currently performing experiments to explore this posibility. However, we would prefer to pursue this additional work in the context of a follow-up study, mainly due to time constraints. We hope the Reviewer will agree that the mechanistic depth of our study is sufficient to justify publication in *EMBO Reports*.

Dear Costas,

Thank you for your patience while I completed the editorial checks on your manuscript. As you know, all three referees support publication of your manuscript pending a few minor changes.

From the editorial side, I need you to address the points listed below, before we can proceed with the official acceptance of your manuscript.

1) „Data & Reagent availability" should be renamed to "Data Availability". This section should only refer to data deposited in public repositories, there please remove the statement "All unique plasmids and cell lines generated in this study are available from the corresponding authors with a completed material transfer agreement."
If the proteomics data (PXD020331) have been published before and reanalyzed here, then please remove that accession information from the Data availability section and refer to the dataset in a "Data citation" instead, wherever relevant (e.g., in the methods section, in the text, or in the figure legend)

Such a Data citation includes the published paper as a normal reference, plus the dataset.

In the text: (Nüchel et al, 2021; Data ref: Nüchel et al, 2021)

In the reference list:
Nüchel J, Tauber M, Nolte JL, Mörgelin M, Türk C, Eckes B, Demetriades C, Plomann M (2021) An mTORC1-GRASP55 signaling axis controls unconventional secretion to reshape the extracellular proteome upon stress. Mol Cell. 81(16):3275-3293.e12

Nüchel J, Tauber M, Nolte JL, Mörgelin M, Türk C, Eckes B, Demetriades C, Plomann M. (2021) PRoteomics IDEntifications Database PXD020331(www.ebi.ac.uk/pride/archive/projects/PXD020331) [DATASET]

2) "Declaration of Interests" needs to be renamed to "Disclosure and Competing Interests Statement".

3) Regarding the Author Contributions, we now use CRediT to specify the contributions of each author in the journal submission system. Therefore, please remove the Author Contributions from the manuscript file and make sure that the author contributions in our online manuscript tracking system are correct and up-to-date. The information you specified in the system will be automatically retrieved and typeset into the article. You can enter additional information in the free text box provided, if you wish. See also our guide to authors https://www.embopress.org/page/journal/14693178/authorguide#authorshipguidelines.

4) Table EV1 is a dataset and should be updated to Dataset EV1 in all places. Please provide a legend in a separate tab of the .xls file.
The other two EV tables need to be renamed to Table EV1 and Table EV2. the legends need to be removed from the manuscript file.

5) I recommend adding the accession number for the dataset you re-analyse in Figure 1B, C for easy reference.

6) The manuscript sections should be in the following order: Title page - Abstract & Keywords - Introduction - Results - Discussion - Methods - Data Availability - Acknowledgments - Disclosure Statement & Competing Interests - References - Figure Legends - (Main Tables with legends if applicable) - Expanded View Figure Legends.

7) Summary should be Abstract

8) Materials and Methods should be Methods

9) BioRender should be acknowledged at the end of the Methods section (not in the Acknowledgments) in the following way:
Graphics:
(some of the... OR Figure #... OR synopsis) Graphics were created with BioRender.com.

10) During our routine image checks, we noticed that the resolution of the submitted figures is too low for blot and microscopy images. This reduction in resolution is commonly caused by converting original 16-bit TIFF files to RGB format for publication. While this is not inherently problematic, it can raise concerns about image integrity for critical readers.

To avoid any misunderstanding and to meet EMBO Press publishing standards, we kindly ask that you:

* Resubmit the complete figure set at the captured original data resolution using 16-bit TIFF.

* Apply the same resolution standards to the blot source data files, which are also currently below the required quality threshold. Please upload the blot source data files using 16-bit TIFF.

11) We perform a routine check on all quantifications. Doing so, I noticed blocks of numbers that are repeated in the source data .xls file you supplied for Figure 3B. Could you please check the attached color-coded file and the respective raw data whether these duplications are OK? (compare e.g., the orange blocks to each other).

12) Please add MW markers for the Western blots in the figures.

13) Please address the following points in the figure legends:
- Please provide the exact p values in the legends of figures 2E, F, G, H; 3B, 4B, D; 5B, D, F; 6B, D; EV2 A-H (unless p<0.0001).
- Please indicate the statistical test used for data analysis in the legend of figure 1B
- Please provide information related to n in the legend of figure 1B

14) As a standard procedure we modify title and abstract. Please find my suggestions below my signature. Note also, that the abstract is limited to 174 words.

15) Finally, EMBO Reports papers are accompanied online by
A) a short (1-2 sentences) summary of the findings and their significance,
B) 2-3 bullet points highlighting key results and
C) a schematic summary figure that provides a sketch of the major findings (not a data image).
Please provide the summary figure as a separate file in PNG or JPG format at a size of 550x300-600 pixels (width x height). Please note that the size is rather small and that text needs to be readable at the final size. Please send us this information along with the revised manuscript.

We look forward to seeing a final version of your manuscript as soon as possible.

With kind regards,

Martina

Martina Rembold, PhD
Senior Editor
EMBO reports

==================

Referee #1:

This is a revised version of paper that examines the role of the Golgi PDZ domain protein GRASP55 in the sorting of lysosomal hydrolases at the Golgi. My review of the initial version of this paper stated that the first five figures of the paper were of a high quality that made a clear and well proven case that GRASP55 is important for correct sorting of lysosomal proteins, but that the remaining part of the paper on a possible link with the COG complex was less well developed and that better mechanistic insight would be valuable.

In revising the paper that authors have added large amount of new data to address the effect of removing GRASP55 on the Golgi components known to be required for correct sorting of lysosomal enzymes, and in particular the GNPTAB enzyme that adds the N-acetylglucosamine-1-phosphate to form the lysosomal sorting signal, as well as LYSET and GOLPH3, the adaptors that link GNPTAB to COPI-coated vesicles and hence ensure that GNPTAB is retained in the Golgi. This new data is based on Golgi purification, immunoprecipitation and immunofluorescence and clearly shows that GRASP55 is required to maintain both LYSET and GOLPH3 on the Golgi, due to an interaction with the latter (or something that binds the latter). This is a striking finding, and it provides a clear explanation for the effects of removing GRASP55. The observation that deletion of GRASP55 causes loss of GOLPH3 from the Golgi is particularly striking and indeed unexpected, and is likely to be of interest to quite a lot of labs studying the retention of Golgi resident proteins.

The authors have also addressed the more minor points I raised and indeed done a particularly good job of engaging thoughtfully with my comments. For what it is worth, I also feel that they have successfully addressed the issues raised by the other two referees. Given this, I am happy to recommend that the paper is accepted for publication in EMBO Reports. There are

a couple of minor further revisions that could improve the presentation and impact:

a) In the immunofluorescence panels in Figures 2-6 the authors show the single channels as greyscale with the merged images in colour. This is a good idea as greyscale is much easier to see and has a better dynamic range for our eyes. It would thus be good to do the same for the new immunofluorescence panels in Figures 7 and 8.

b) The authors examine a form of GNPTAB that is not dependent on LYSET for Golgi retention (QELL) and show that it restores lysosomal enzyme sorting when GRASP55 is absent. Did the authors check if this restored the Golgi localisation of GOLPH3? This would seem a simply experiment and would add mechanistic insight irrespective of the outcome.

Referee #2:

In the revised manuscript, Nuechel et al. have added new data that clarify the relationship between GRASP55 and other proteins that are required for correct Golgi localization of GNPTAB and, consequently, trafficking of lysosomal hydrolases. The authors also have streamlined the results section to focus on the mechanistic and functional aspects of GRASP55. Overall, this is a substantial improvement of the manuscript that is, in principle, suitable for publication in EMBO Reports. The discussion and interpretation of the data still have several issues that should be addressed in the manuscript.

1. Figure 1D: I appreciate the authors' explanation of nonspecific bands for the M6P antibody. However, I remain unconvinced by the GRASP55 KO effects in this blot. GNPTAB KO leads to a near-complete loss of multiple bands, whereas the effect of GRASP55 KO is much more modest and overall comparable to that of GRASP65 KO. To demonstrate a convincing effect, a quantification of band intensities across multiple independent experiments might help. Alternatively, the authors could consider removing this blot and limiting the results to the clear effects of GRASP55 KO on GNPTAB localization and enzyme mistrafficking (which point to an M6P pathway defect).

2. As the authors correctly point out, their data demonstrate that GRASP55 is necessary for proper lysosomal enzyme sorting and lysosome function. However, necessity alone does not imply regulation. In biological terms, a regulator is a factor that modulates a process, adjusting its activity or output, rather than simply being required for it to occur. If the authors want to make the claim that GRASP55 is a key regulator of lysosomal function, especially in the abstract, they should demonstrate that it actively tunes lysosomal enzyme sorting or function. Alternatively, they might want to change their wording to something along the lines of 'necessary for lysosomal function'.

3. In the absence of any evidence that link GRASP55 to lysosomal storage disorders or related disorders, this is an interesting point that is appropriate for the discussion. Please remove any references to LSDs from the summary and the results summary of the introduction.

4. The authors point out that they never claim a direct role for GRASP55 in the regulation of mTORC1. They dedicate a large part of the discussion to a chapter entitled 'GRASP55 lies at the center of mTORC1 signaling' - which is a much stronger claim than merely a direct role! This is an interesting paper and to do it justice, the authors may want to consider that a concise discussion which comes across as thoughtful and balanced would be received more favorably.

Referee #3:

The revised version of the manuscript is significantly improved over the original version previously submitted to EMBO J. It provides new mechanistic information as to how GRASP55 functions in lysosomal enzyme modification. This is shown to be via GOLPH3 and LYSET to retain the GNPTAB enzyme within the Golgi. The new data are clear and support the authors' conclusions. The paper is suitable for EMBOR in my opinion.

==================

GRASP55 maintains lysosome function by controlling sorting of lysosomal enzymes at the Golgi

Lysosomes are multifunctional organelles that play important roles in cellular recycling, signaling, and homeostasis, relying on precise trafficking and activation of lysosomal enzymes. While the Golgi apparatus plays a central role in lysosomal enzyme sorting, the mechanisms linking Golgi function to lysosomal activity remain incompletely understood. Here, we identify the Golgi-resident protein GRASP55, but not its paralog GRASP65, as necessary for lysosome function. Loss of GRASP55 expression leads to missorting and secretion of lysosomal enzymes, lysosomal dysfunction and bloating. GRASP55 deficiency also disrupts

lysosomal mTORC1 signaling, reducing the phosphorylation of its lysosomal substrates TFEB/TFE3, while sparing its non-lysosomal targets. Mechanistically, GRASP55 binds and maintains the COPI adaptor GOLPH3 protein at the Golgi, thereby controlling the Golgi localization and stability of LYSET and GNPTAB that are required for mannose 6-phosphate (M6P) tagging of lysosomal enzymes. These findings reveal an essential role for GRASP55 in Golgi-lysosome communication and lysosomal enzyme trafficking and underscore the importance of Golgi-mediated protein sorting in lysosome function and lysosomal mTORC1 signaling.

Response to Referees

EMBOR-2025-61967V2

GRASP55 maintains lysosome function by controlling sorting of lysosomal enzymes at the Golgi

Nüchel et al.

Referee #1

This is a revised version of paper that examines the role of the Golgi PDZ domain protein GRASP55 in the sorting of lysosomal hydrolases at the Golgi. My review of the initial version of this paper stated that the first five figures of the paper were of a high quality that made a clear and well proven case that GRASP55 is important for correct sorting of lysosomal proteins, but that the remaining part of the paper on a possible link with the COG complex was less well developed and that better mechanistic insight would be valuable.

In revising the paper that authors have added large amount of new data to address the effect of removing GRASP55 on the Golgi components known to be required for correct sorting of lysosomal enzymes, and in particular the GNPTAB enzyme that adds the N-acetylglucosamine-1-phosphate to form the lysosomal sorting signal, as well as LYSET and GOLPH3, the adaptors that link GNPTAB to COPI-coated vesicles and hence ensure that GNPTAB is retained in the Golgi. This new data is based on Golgi purification, immunoprecipitation and immunofluorescence and clearly shows that GRASP55 is required to maintain both LYSET and GOLPH3 on the Golgi, due to an interaction with the latter (or something that binds the latter). This is a striking finding, and it provides a clear explanation for the effects of removing GRASP55. The observation that deletion of GRASP55 causes loss of GOLPH3 from the Golgi is particularly striking and indeed unexpected, and is likely to be of interest to quite a lot of labs studying the retention of Golgi resident proteins.

The authors have also addressed the more minor points I raised and indeed done a particularly good job of engaging thoughtfully with my comments. For what it is worth, I also feel that they have successfully addressed the issues raised by the other two referees. Given this, I am happy to recommend that the paper is accepted for publication in EMBO Reports. There are a couple of minor further revisions that could improve the presentation and impact:

We sincerely thank the Reviewer for the positive assessment of our work and for acknowledging our efforts during the revision process. We particularly appreciate the recognition of the impact of the new data and the reviewer's thoughtful engagement with our responses. We are grateful for the reviewer's support and for considering the revised manuscript suitable for publication.

a) In the immunofluorescence panels in Figures 2-6 the authors show the single channels as greyscale with the merged images in colour. This is a good idea as greyscale is much easier to see and has a better dynamic range for our eyes. It would thus be good to do the same for the new immunofluorescence panels in Figures 7 and 8.

We agree and have implemented the Referee's suggestion. The immunofluorescence panels in Figures 7 and 8 now follow the same format, with single channels shown in greyscale and merged images in color.

b) The authors examine a form of GNPTAB that is not dependent on LYSET for Golgi retention (QELL) and show that it restores lysosomal enzyme sorting when GRASP55 is absent. Did the authors check if this restored the Golgi localisation of GOLPH3? This would seem a simply experiment and would add mechanistic insight irrespective of the outcome.

As we and others have shown, GNPTAB functions downstream of the LYSET-GOLPH3 machinery that mediates its COPI-dependent Golgi retention. As GNPTAB is a client of this pathway rather than an upstream regulator, it is not intuitive that expression of a LYSET-independent GNPTAB mutant would, in turn, influence GOLPH3 localization. For this reason, and given the time constraints, we did not pursue this experiment and hope the editor agrees that it is not essential for the conclusions of the study.

Referee #2

In the revised manuscript, Nuechel et al. have added new data that clarify the relationship between GRASP55 and other proteins that are required for correct Golgi localization of GNPTAB and, consequently, trafficking of lysosomal hydrolases. The authors also have streamlined the results section to focus on the mechanistic and functional aspects of GRASP55. Overall, this is a substantial improvement of the manuscript that is, in principle,

suitable for publication in EMBO Reports. The discussion and interpretation of the data still have several issues that should be addressed in the manuscript.

1. Figure 1D: I appreciate the authors' explanation of nonspecific bands for the M6P antibody. However, I remain unconvinced by the GRASP55 KO effects in this blot. GNPTAB KO leads to a near-complete loss of multiple bands, whereas the effect of GRASP55 KO is much more modest and overall comparable to that of GRASP65 KO. To demonstrate a convincing effect, a quantification of band intensities across multiple independent experiments might help. Alternatively, the authors could consider removing this blot and limiting the results to the clear effects of GRASP55 KO on GNPTAB localization and enzyme mistrafficking (which point to an M6P pathway defect).

We respectfully disagree with the reviewer's assessment and would prefer to retain this experiment. As indicated in the figure, several bands detected by the anti-M6P antibody are nonspecific, as they remain present in GNPTAB KO cells. In the figure, arrowheads mark the bands that disappear in GNPTAB KO samples and therefore represent bona fide M6P-modified proteins. The intensity of these bands is reduced in GRASP55 KO cells, but not in GRASP65 KO cells.

Importantly, the more modest reduction observed in GRASP55 KO cells compared to GNPTAB KO cells is consistent with the overall phenotypes described in the manuscript. In contrast to GNPTAB KO cells, which completely lack the enzyme, GRASP55 KO cells still express GNPTAB and retain partial Golgi localization, leading to a partial reduction rather than a complete loss of M6P modification.

We also note that the other two Reviewers did not raise concerns regarding this experiment. Given that the relevant bands are clearly indicated and the data are consistent with the overall phenotypic strength observed throughout the study, we consider this an important control demonstrating functional consequences of GRASP55 loss on lysosomal enzyme modification and therefore prefer to retain the experiment in the manuscript.

2. As the authors correctly point out, their data demonstrate that GRASP55 is necessary for proper lysosomal enzyme sorting and lysosome function. However, necessity alone does not imply regulation. In biological terms, a regulator is a factor that modulates a process, adjusting its activity or output, rather than simply being required for it to occur. If the authors want to

make the claim that GRASP55 is a key regulator of lysosomal function, especially in the abstract, they should demonstrate that it actively tunes lysosomal enzyme sorting or function. Alternatively, they might want to change their wording to something along the lines of 'necessary for lysosomal function'.

We have now updated our abstract as per the Editor's suggestions, which we believe fully addresses this point.

3. In the absence of any evidence that link GRASP55 to lysosomal storage disorders or related disorders, this is an interesting point that is appropriate for the discussion. Please remove any references to LSDs from the summary and the results summary of the introduction.

We have now updated our abstract as per the editor's suggestions, which we believe fully addresses this point.

4. The authors point out that they never claim a direct role for GRASP55 in the regulation of mTORC1. They dedicate a large part of the discussion to a chapter entitled 'GRASP55 lies at the center of mTORC1 signaling' - which is a much stronger claim than merely a direct role! This is an interesting paper and to do it justice, the authors may want to consider that a concise discussion which comes across as thoughtful and balanced would be received more favorably.

This part in the Discussion highlights that loss of GRASP55 disrupts lysosomal enzyme sorting, which in turn impairs lysosome function and lysosomal mTORC1 signaling. This effect is fully supported by our data showing altered hydrolase localization, lysosomal bloating, and lysosomal mTORC1 substrate phosphorylation. This is conceptually analogous to the lysosomal v-ATPase, which is widely considered central to lysosomal mTORC1 signaling due to its role in maintaining lysosomal acidification and protease activity, not because it directly regulates mTORC1. Furthermore, we previously showed that GRASP55 is a direct, Golgi-based substrate of mTORC1 (Nüchel et al. 2021 *Mol Cell*), thus underscoring the bidirectional nature of the relationship between mTORC1 and GRASP55. We believe this framing is accurate, logically grounded in the data, and provides important context for understanding the functional consequences of GRASP55 loss, and therefore we prefer to retain it in the manuscript as it currently is.

Referee #3

The revised version of the manuscript is significantly improved over the original version previously submitted to EMBO J. It provides new mechanistic information as to how GRASP55 functions in lysosomal enzyme modification. This is shown to be via GOLPH3 and LYSET to retain the GNPTAB enzyme within the Golgi. The new data are clear and support the authors' conclusions. The paper is suitable for EMBOR in my opinion.

Thank you!

Dr. Constantinos Demetriades
MPI for Biology of Ageing
AG Demetriades
Joseph Stelzmann Str 9B
Cologne 50931
Germany

Dear Costas,

I am very pleased to accept your manuscript for publication in the next available issue of EMBO reports. Thank you for your contribution to our journal.

Your manuscript will be processed for publication by EMBO Press. It will be copy edited and you will receive page proofs prior to publication. Please note that you will be contacted by Springer Nature Author Services to complete licensing and payment information.

You may qualify for financial assistance for your publication charges - either via a Springer Nature fully open access agreement or an EMBO initiative. Check your eligibility: https://link.springer.com/journal/44319/how-to-publish-with-us

Should you be planning a Press Release on your article, please get in contact with embo_production@springernature.com as early as possible in order to coordinate publication and release dates.

If you have any questions, please do not hesitate to contact the Editorial Office. Thank you for your contribution to EMBO Reports.

Best regards,

Martina

Martina Rembold, PhD
Senior Editor
EMBO reports

>>> Please note that it is EMBO Reports policy for the transcript of the editorial process (containing referee reports and your response letter) to be published as an online supplement to each paper. If you do NOT want this, you will need to inform the Editorial Office via email immediately. More information is available here: https://link.springer.com/partners/embo-press/editorial-policies#Peer%20review

EMBO Press Author Checklist

Corresponding Author Name: Constantinos Demetriades	
Journal Submitted to: EMBO Reports	
Manuscript Number: EMBOR-2025-61967V2	

USEFUL LINKS FOR COMPLETING THIS FORM
The EMBO Journal - Author Guidelines
EMBO Reports - Author Guidelines
Molecular Systems Biology - Author Guidelines
EMBO Molecular Medicine - Author Guidelines

Reporting Checklist for Life Science Articles (updated January
This checklist is adapted from Materials Design Analysis Reporting (MDAR) Checklist for Authors. MDAR establishes a minimum set of requirements in transparent reporting in the life sciences (see Statement of Task: 10.31222/osf.io/9sm4x). Please follow the journal's guidelines in preparing your
Please note that a copy of this checklist will be published alongside your article.

Abridged guidelines for figures
1. Data
The data shown in figures should satisfy the following conditions:
- the data were obtained and processed according to the field's best practice and are presented to reflect the results of the experiments in an accurate and unbiased manner.
- ideally, figure panels should include only measurements that are directly comparable to each other and obtained with the same assay.
- plots include clearly labeled error bars for independent experiments and sample sizes. Unless justified, error bars should not be shown for technical
- if n<5, the individual data points from each experiment should be plotted. Any statistical test employed should be justified.
- Source Data should be included to report the data underlying figures according to the guidelines set out in the authorship guidelines on Data

2. Captions
Each figure caption should contain the following information, for each panel where they are relevant:
- a specification of the experimental system investigated (eg cell line, species name).
- the assay(s) and method(s) used to carry out the reported observations and measurements.
- an explicit mention of the biological and chemical entity(ies) that are being measured.
- an explicit mention of the biological and chemical entity(ies) that are altered/varied/perturbed in a controlled manner.
- the exact sample size (n) for each experimental group/condition, given as a number, not a range;
- a description of the sample collection allowing the reader to understand whether the samples represent technical or biological replicates (including how many animals, litters, cultures, etc.).
- a statement of how many times the experiment shown was independently replicated in the laboratory.
- definitions of statistical methods and measures:
  - common tests, such as t-test (please specify whether paired vs. unpaired), simple χ2 tests, Wilcoxon and Mann-Whitney tests, can be unambiguously identified by name only, but more complex techniques should be described in the methods section;
  - are tests one-sided or two-sided?
  - are there adjustments for multiple comparisons?
  - exact statistical test results, e.g., P values = x but not P values < x;
  - definition of 'center values' as median or average;
  - definition of error bars as s.d. or s.e.m.

Please complete ALL of the questions below.
Select "Not Applicable" only when the requested information is not relevant for your study.

Materials

Newly Created Materials	Information included in the manuscript?	In which section is the information available? (Reagents and Tools Table, Materials and Methods, Figures, Data Availability Section)
New materials and reagents need to be available; do any restrictions apply?	Yes	All unique plasmids and cell lines generated in this study are available from the corresponding authors with a completed material transfer agreement

Antibodies	Information included in the manuscript?	In which section is the information available? (Reagents and Tools Table, Materials and Methods, Figures, Data Availability Section)
For **antibodies** provide the following information:<br>- Commercial antibodies: RRID (if possible) or supplier name, catalogue number and or/clone number<br>- Non-commercial: RRID or citation	Yes	Reagents and Tools Table; Table EV1

DNA and RNA sequences	Information included in the manuscript?	In which section is the information available? (Reagents and Tools Table, Materials and Methods, Figures, Data Availability Section)
**Short novel DNA or RNA including primers, probes**: provide the sequences.	Yes	Table EV2

Cell materials	Information included in the manuscript?	In which section is the information available? (Reagents and Tools Table, Materials and Methods, Figures, Data Availability Section)
**Cell lines:** Provide species information, strain. Provide accession number in repository **OR** supplier name, catalog number, clone number, and/**OR** RRID.	Yes	Materials and Methods; Reagents and Tools Table
**Primary cultures:** Provide species, strain, sex of origin, genetic modification status.	Not Applicable	
Report if the cell lines were recently **authenticated** (e.g., by STR profiling) and tested for mycoplasma contamination.	Yes	Materials and Methods

Experimental animals	Information included in the manuscript?	In which section is the information available? (Reagents and Tools Table, Materials and Methods, Figures, Data Availability Section)
**Laboratory animals or Model organisms:** Provide species, strain, sex, age, genetic modification status. Provide accession number in repository **OR** supplier name, catalog number, clone number, **OR** RRID.	Not Applicable	
**Animal observed in or captured from the field:** Provide species, sex, and age where possible.	Not Applicable	
Please detail **housing and husbandry conditions**.	Not Applicable	

Plants and microbes	Information included in the manuscript?	In which section is the information available? (Reagents and Tools Table, Materials and Methods, Figures, Data Availability Section)
**Plants:** provide species and strain, ecotype and cultivar where relevant, unique accession number if available, and source (including location for collected wild specimens).	Not Applicable	
**Microbes:** provide species and strain, unique accession number if available, and source.	Not Applicable	

Human research participants	Information included in the manuscript?	In which section is the information available? (Reagents and Tools Table, Materials and Methods, Figures, Data Availability Section)
If collected and within the bounds of privacy constraints report on age, sex and gender or ethnicity for all study participants.	Not Applicable	

Core facilities	Information included in the manuscript?	In which section is the information available? (Reagents and Tools Table, Materials and Methods, Figures, Data Availability Section)
If your work benefited from core facilities, was their service mentioned in the acknowledgments section?	Yes	Acknowledgements

Design

Study protocol	Information included in the manuscript?	In which section is the information available? (Reagents and Tools Table, Materials and Methods, Figures, Data Availability Section)
If study protocol has been **pre-registered, provide DOI in the manuscript**. For clinical trials, provide the trial registration number **OR** cite DOI.	Not Applicable	
Report the **clinical trial registration number** (at ClinicalTrials.gov or equivalent), where applicable.	Not Applicable	

Laboratory protocol	Information included in the manuscript?	In which section is the information available? (Reagents and Tools Table, Materials and Methods, Figures, Data Availability Section)
Provide DOI OR other citation details if **external detailed step-by-step protocols** are available.	Not Applicable	

Experimental study design and statistics	Information included in the manuscript?	In which section is the information available? (Reagents and Tools Table, Materials and Methods, Figures, Data Availability Section)
Include a statement about **sample size** estimate even if no statistical methods were used.	Yes	Materials and Methods
Were any steps taken to minimize the effects of subjective bias when allocating animals/samples to treatment (e.g. **randomization procedure**)? If yes, have they been described?	Yes	Materials and Methods
Include a statement about **blinding** even if no blinding was done.	Yes	Materials and Methods
Describe **inclusion/exclusion criteria** if samples or animals were excluded from the analysis. Were the criteria pre-established?<br><br>If sample or data points were omitted from analysis, report if this was due to attrition or intentional exclusion and provide justification.	Yes	Materials and Methods
For every figure, are **statistical tests** justified as appropriate? Do the data meet the assumptions of the tests (e.g., normal distribution)? Describe any methods used to assess it. Is there an estimate of variation within each group of data? Is the variance similar between the groups that are being statistically compared?	Yes	Materials and Methods

Sample definition and in-laboratory replication	Information included in the manuscript?	In which section is the information available? (Reagents and Tools Table, Materials and Methods, Figures, Data Availability Section)
In the figure legends: state number of times the experiment was **replicated** in laboratory.	Yes	Figure legends
In the figure legends: define whether data describe **technical or biological replicates**.	Yes	Figure legends

Ethics

Ethics	Information included in the manuscript?	In which section is the information available? (Reagents and Tools Table, Materials and Methods, Figures, Data Availability Section)
Studies involving **human participants**: State details of **authority granting ethics approval** (IRB or equivalent committee(s), provide reference number for approval.	Not Applicable	
Studies involving **human participants**: Include a statement confirming that **informed consent** was obtained from all subjects and that the experiments conformed to the principles set out in the WMA Declaration of Helsinki and the Department of Health and Human Services Belmont Report.	Not Applicable	
Studies involving **human participants:** For publication of **patient photos**, include a statement confirming that consent to publish was obtained.	Not Applicable	
Studies involving experimental **animals**: State details of **authority granting ethics approval** (IRB or equivalent committee(s), provide reference number for approval. Include a statement of compliance with ethical regulations.	Not Applicable	
Studies involving **specimen and field samples:** State if relevant **permits** obtained, provide details of authority approving study; if none were required, explain why.	Not Applicable	

Dual Use Research of Concern (DURC)	Information included in the manuscript?	In which section is the information available? (Reagents and Tools Table, Materials and Methods, Figures, Data Availability Section)
Could your study fall under dual use research restrictions? Please check biosecurity documents and list of **select agents and toxins** (CDC): https://www.selectagents.gov/sat/list.htm	Not Applicable	
If you used a select agent, is the security level of the lab appropriate and reported in the manuscript?	Not Applicable	
If a study is subject to dual use research of concern regulations, is the name of the **authority granting approval and reference number** for the regulatory approval provided in the manuscript?	Not Applicable	

Reporting

The MDAR framework recommends adoption of discipline-specific guidelines, established and endorsed through community initiatives. Journals have their own policy about requiring specific guidelines and recommendations to complement MDAR.

Adherence to community standards	Information included in the manuscript?	In which section is the information available? (Reagents and Tools Table, Materials and Methods, Figures, Data Availability Section)
State if relevant guidelines or checklists (e.g., **ICMJE, MIBBI, ARRIVE, PRISMA**) have been followed or provided.	Not Applicable	
For **tumor marker prognostic studies,** we recommend that you follow the **REMARK** reporting guidelines (see link list at top right). See author guidelines, under 'Reporting Guidelines'. Please confirm you have followed these guidelines.	Not Applicable	
For **phase II and III randomized controlled trials**, please refer to the **CONSORT** flow diagram (see link list at top right) and submit the CONSORT checklist (see link list at top right) with your submission. See author guidelines, under 'Reporting Guidelines'. Please confirm you have submitted this list.	Not Applicable	

Data Availability

Data availability	Information included in the manuscript?	In which section is the information available? (Reagents and Tools Table, Materials and Methods, Figures, Data Availability Section)
Have **primary datasets** been deposited according to the journal's guidelines (see 'Data Deposition' section) and the respective accession numbers provided in the Data Availability Section?	Not Applicable	
Were **human clinical and genomic datasets** deposited in a public access-controlled repository in accordance to ethical obligations to the patients and to the applicable consent agreement?	Not Applicable	
Are **computational models** that are central and integral to a study available without restrictions in a machine-readable form? Were the relevant accession numbers or links provided?	Not Applicable	
If publicly available data were reused, provide the respective **data citations in the reference list**.	Yes	References: Nuchel et. al 2021 Mol Cell
